# Formally Stereoretentive S_N_1 Reactions of Homoallylic Tertiary Alcohols Via Nonclassical Carbocation

**DOI:** 10.1021/jacs.5c05680

**Published:** 2025-05-27

**Authors:** Kaushalendra Patel, Leonie Wilczek, Francesco Calogero, Ilan Marek

**Affiliations:** Schulich Faculty of Chemistry and the Resnick Sustainability Center for Catalysis, 26747 Technion−Israel Institute of Technology, Haifa, 3200009, Israel

## Abstract

We present a stereoretentive nucleophilic substitution of homoallylic tertiary alcohols via the formation of a nonclassical cyclopropyl carbinyl (CPC) carbocation intermediate. This strategy enables the creation of highly congested tertiary centers with preserved stereocontrol, addressing the typical challenges of carbocation instability and reactivity in S_N_1 mechanisms. The stabilization of the CPC intermediate is crucial for achieving precise regio- and stereoselectivity, significantly enhancing the utility of S_N_1-type mechanisms in complex molecule synthesis.

## Graphical abstract

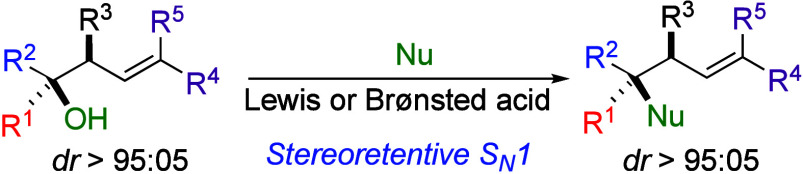

Carbocation chemistry has constantly attracted significant attention serving as a cornerstone in fundamental organic transformations and various industrial processes.^1^ However, starting from enantiopure alkyl halides, achieving precise stereocontrol in carbocation reactions, particularly at the level of intimate ion pairs^2^ or solvent-separated ion pairs,^3^ remains an inherently challenging task.^4^ Nevertheless, stereochemical control at a tertiary center has been reported in the literature, including examples of enantioconvergent S_N_1 transformations (Scheme 1a).^5^ On the other hand, nonclassical carbocations are stabilized by charge delocalization through neighboring C–C or C–H bonds, forming bridged intermediates with penta-coordinated carbon atoms, best described by three-center, two-electron bonds (Scheme 1b illustrates the formation of nonclassical carbocations starting from cyclopropylmethyl, cyclobutyl, or homoallyl cations).^6^ Unlike classical planar trivalent cations, nonclassical cations contain stereochemical information embedded within their tetracoordinated structure, and this unique feature presents a potential pathway for stereocontrol. Notably, the nonclassical cyclopropyl carbinyl cations (CPCs), supported by low-temperature NMR studies^7^ and computational analysis,^8^ inherently have one face shielded by its carbon skeleton (Scheme 1c), and when suitably substituted, it may undergo a selective nucleophilic attack (at either C_3_ or C_4_) from the unshielded face.^9^ This stereospecific nature of nonclassical carbocations opens exciting possibilities for developing stereoselective transformations as elegantly demonstrated in the Lewis acid-catalyzed rearrangement of cycloheptenyl bromide by Feringa^10^ or through catalytic enantiocontrol additions on achiral 2-exonorbornyl trichloroacetamide by Schreiner and List.^11^ In this context, our recent efforts have focused on the chemistry of nonclassical carbocations formed via the dissociation of cyclopropyl carbinol (Scheme 1d).^12,13^ Upon treatment of cyclopropyl carbinol derivative with 10 mol % of tris­(pentafluorophenyl)­borane in water, a nonclassical cyclopropyl carbinyl carbocation intermediate **A** (CPC) is initially formed. This intermediate reacts with water to give the corresponding homoallyl alcohol **1** with excellent diastereomeric ratio and yield.^13^ Controlling reactivity at the CPC intermediate stage allowed nucleophilic substitution at a quaternary carbon stereocenter with complete inversion of configuration, even with weak nucleophiles such as water, among others (Scheme 1d).^13,14^ This transformation addresses a critical gap in organic chemistry where crowded quaternary carbon stereocenters undergo stereospecific nucleophilic substitution involving C–C bond cleavage.^15,16^

**Scheme 1:**
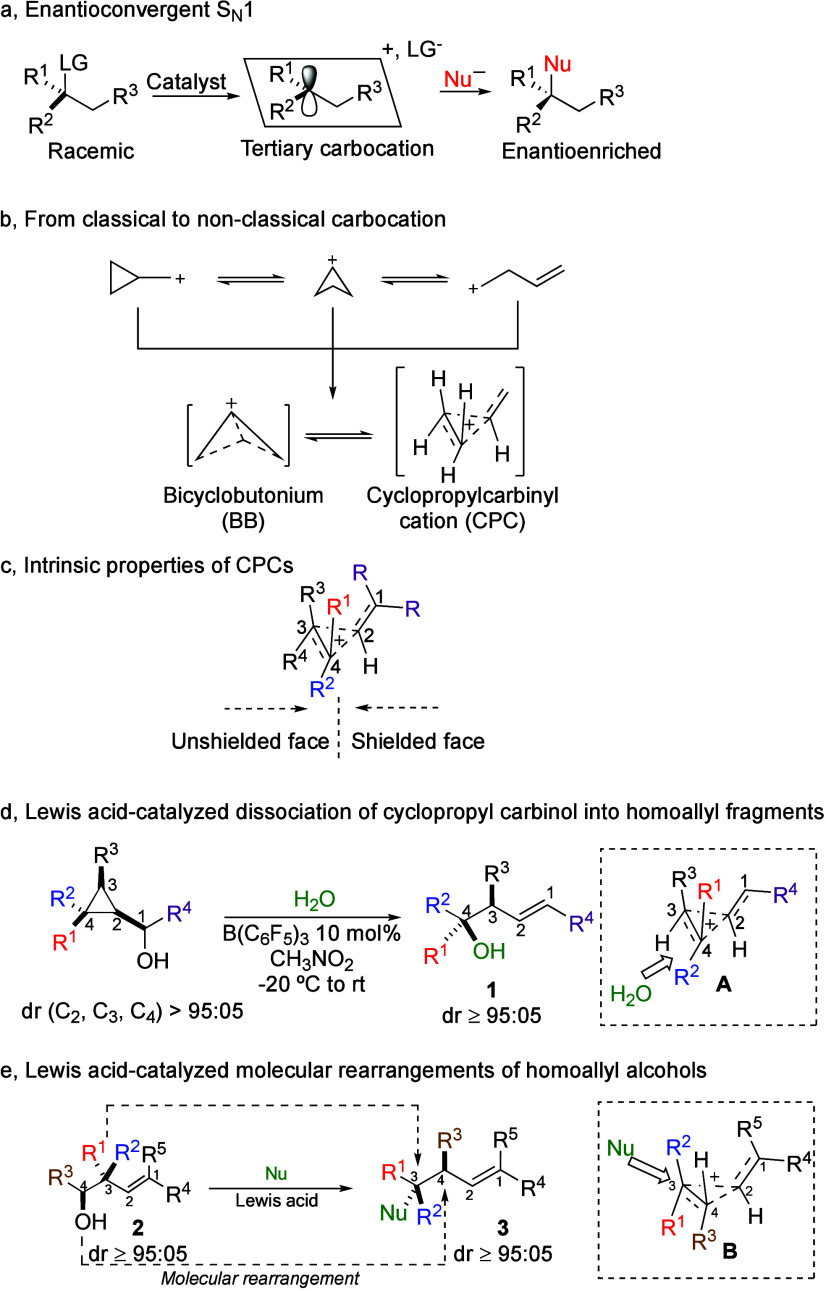
Stereochemistry and Carbocations

Building on our success in controlling the reactivity of nucleophilic substitution involving cyclopropyl carbinyl cation **A** in stereoselective synthesis, we have expanded our approach to the stereospecific molecular rearrangement of homoallyl alcohols^17^ **2** (Scheme 1e).^18^ Upon activation by a Lewis acid and π-participation, the cyclopropyl carbinyl cation **B** is formed, and although the initially generated carbocation is located at the secondary carbon center (C_4_), the presence of a quaternary carbon stereocenter at C_3_ results in a slightly elongated C_2_–C_3_ bond compared to the C_2_–C_4_ bond in **B**.^8^ This structural feature directs the regioselective nucleophile attack at C_3_, occurring anti to the C_3_–C_2_ bond, providing **3** with an excellent diastereomeric ratio. This complete molecular rearrangement shifts the initial secondary carbon center (C_4_), originally bearing the hydroxy group, to the middle of the molecular chain of **3**. The overall process results in the cleavage of a quaternary C–C bond (C_3_) substituted with a nucleophile. This molecular rearrangement proceeds with a double inversion of configuration at both C_3_ and C_4_.^18^

Based on these results, we next wondered whether homoallyl alcohol derivatives **1** might now serve as a new platform to provide complete *stereoretentive* nucleophilic substitution as described in Scheme 2. Our proposed strategy would rely on the peculiar reactivity of **1**, possessing a tertiary alcohol adjacent to an allylic tertiary carbon stereocenter. Upon activation with a Lewis or Brønsted acid, **1** should provide the cyclopropyl carbinyl cation intermediate **C**. In this intermediate, the positive charge in **C** is expected to be more localized at the quaternary carbon stereocenter C_4_.^7,8^ Consequently, the nucleophile would preferentially react at C_4_ to provide **4** with an excellent diastereomeric ratio. Considering the respective stereochemistry of **1** and **4**, the nucleophilic substitution would proceed with an overall pure retention of the configuration through π-bond participation. Although stereoretentive nucleophilic substitution reactions have been reported in the literature though an anchimeric effect,^19^ to our knowledge, it never received attention via the formation of a nonclassical carbocation such as cyclopropyl carbinyl cation **C**.

**Scheme 2:**
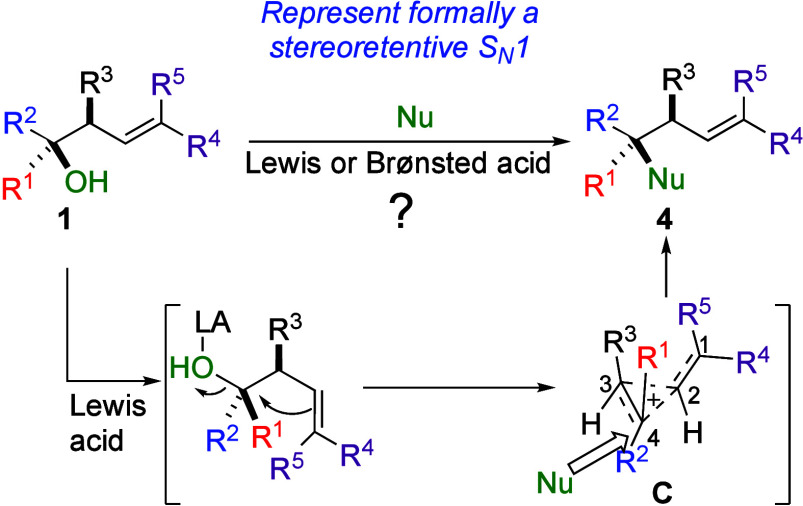
Proposed Transformation with Mechanistic Blueprint

With convenient access to **1** (Scheme 1d),^13^ we started our investigation by treating **1a** with DAST (Et_2_NSF_3_)^20^ at low temperature in CH_2_Cl_2_ and were delighted to obtain the corresponding tertiary alkyl fluoride **4a** in excellent yield and with a high diastereomeric ratio (*dr* 95:05, with a stereospecificity *sp* > 98:02, Scheme 3).^21^ The determination of the relative configuration of **4a** ^14a^ revealed that the *S*_*N*_ *1 reaction proceeded indeed with a complete overall retention of configuration*. Attempts to perform the same transformation with other fluorinating reagents, such as HBF_4_ (50% aqueous) and HBF_4_ (50% in diethyl ether), led to no reaction in the former case and only elimination products in the latter.^22^ Building on this promising initial result, we broadened the scope of the transformation to various structural motifs (Scheme 3). For instance, the reaction is stereospecific, as the two diastereomers at the tertiary alkyl fluoride carbon center, **4b** and **4c**, could be obtained with similar yields and selectivities by simply permuting the stereochemistry of the two substituents at the tertiary alcohol center in the corresponding starting materials **1b** (R^1^ = Me, R^2^ = Bu) and **1c** (R^1^ = Bu, R^2^ = Me), respectively. The relative configurations were determined by chemical correlation, as was the case for all other products described in this manuscript, with full details provided in the Supporting Information. Various aromatic substituents with different electronic properties, such as electron-donating or electron-withdrawing groups, could equally participate in the reaction, providing excellent yields and selectivities (**4b**–**4f**, Scheme 3). Furthermore, starting materials featuring an electron-donating methoxy group at the ortho, meta, or para positions were successfully converted into the corresponding products with similar yields and selectivities (**4i**–**4k**, Scheme 3). It is worth noting that the presence of R^3^ = CO_2_Et is not essential for the reaction to proceed selectively, as it also proceeded smoothly with other substituents, such as CH_2_OMe, allyl, propyl, and benzyl (**4f**–**4x**, Scheme 3). Remarkably, replacing an aryl group at the R^4^ position with a diene did not significantly impact the reaction outcome (**4m**, Scheme 3). Likewise, the incorporation of aliphatic groups at the R^4^ position resulted in the desired product, without notable changes (**4n**–**4p**, Scheme 3).

**Scheme 3:**
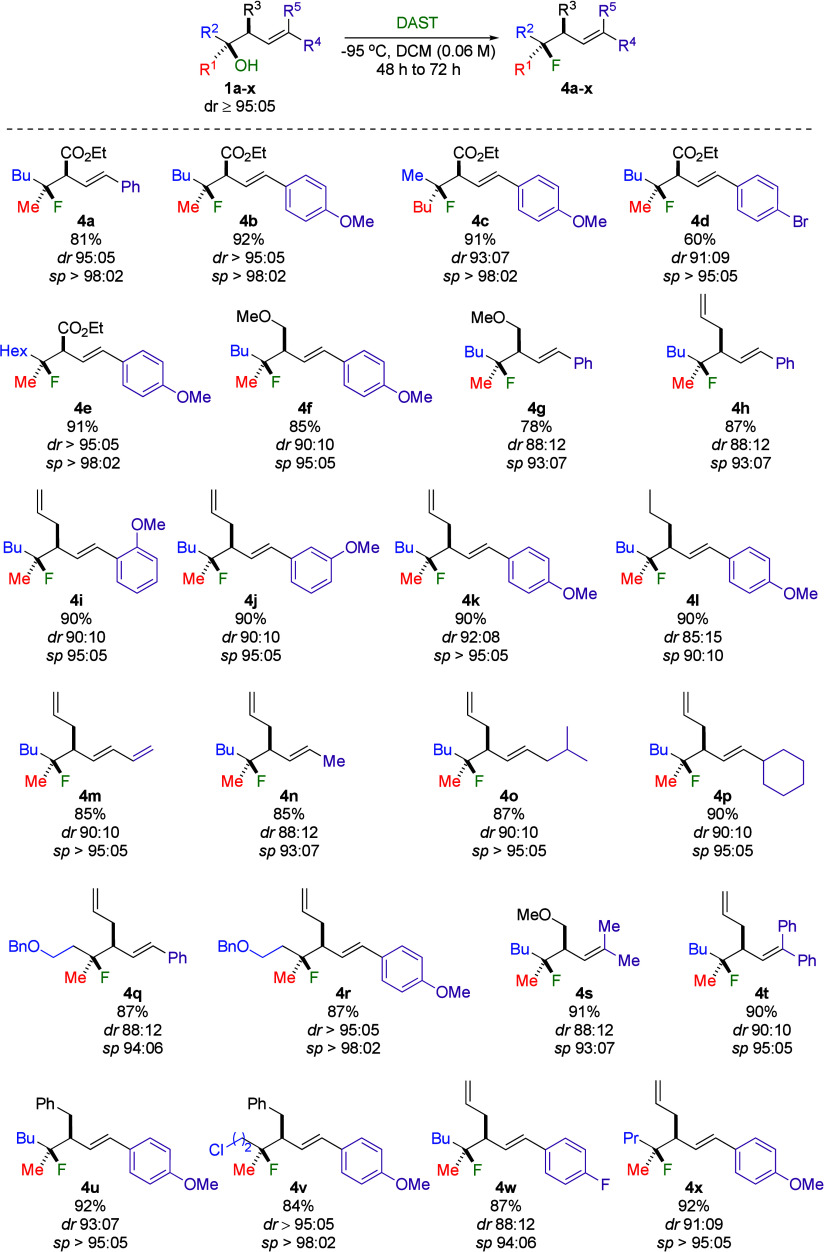
Stereoretentive S_N_1 Fluorination Reaction

When R^5^ ≠ H (**1s** and **1t**, see Supporting Information), the reaction proceeds smoothly yielding trisubstituted alkenes **4s** and **4t** (Scheme 3) with excellent yields and selectivities. Importantly, the reaction tolerates functional groups such as OMe, OBn, and Cl, underscoring selective fluorination of the tertiary alcohol even in the presence of functional groups (**4f**, **4g**, **4q**, **4r**, **4s**, and **4v**, Scheme 3). Although a stereoretentive S_N_i-type mechanism involving an intimate ion pair could also be considered, performing the same reaction on the reduced product (lacking the double bond) predominantly yielded elimination products. NMR analysis of the crude reaction mixture revealed a diastereomeric ratio of approximately 60:40, underscoring the crucial role of the double bond in enabling the desired reactivity via the formation of a cyclopropylcarbinyl cation.

Following the successful stereoretentive nucleophilic fluorination of tertiary homoallylic alcohols, we switched our attention to different types of nucleophiles and, in particular, bromination, chlorination, and azidation reactions (Scheme 4a-b). The use of 5 mol % of Fe­(OTf)_3_ in DCM (0.06 M) at −45 °C to −40 °C with TMSBr (2 equiv) afforded the tertiary homoallylic bromide **4y** in excellent yield (92%) and diastereoselectivity (*dr* 95:05, *sp* > 98:02, Scheme 4a).

**Scheme 4:**
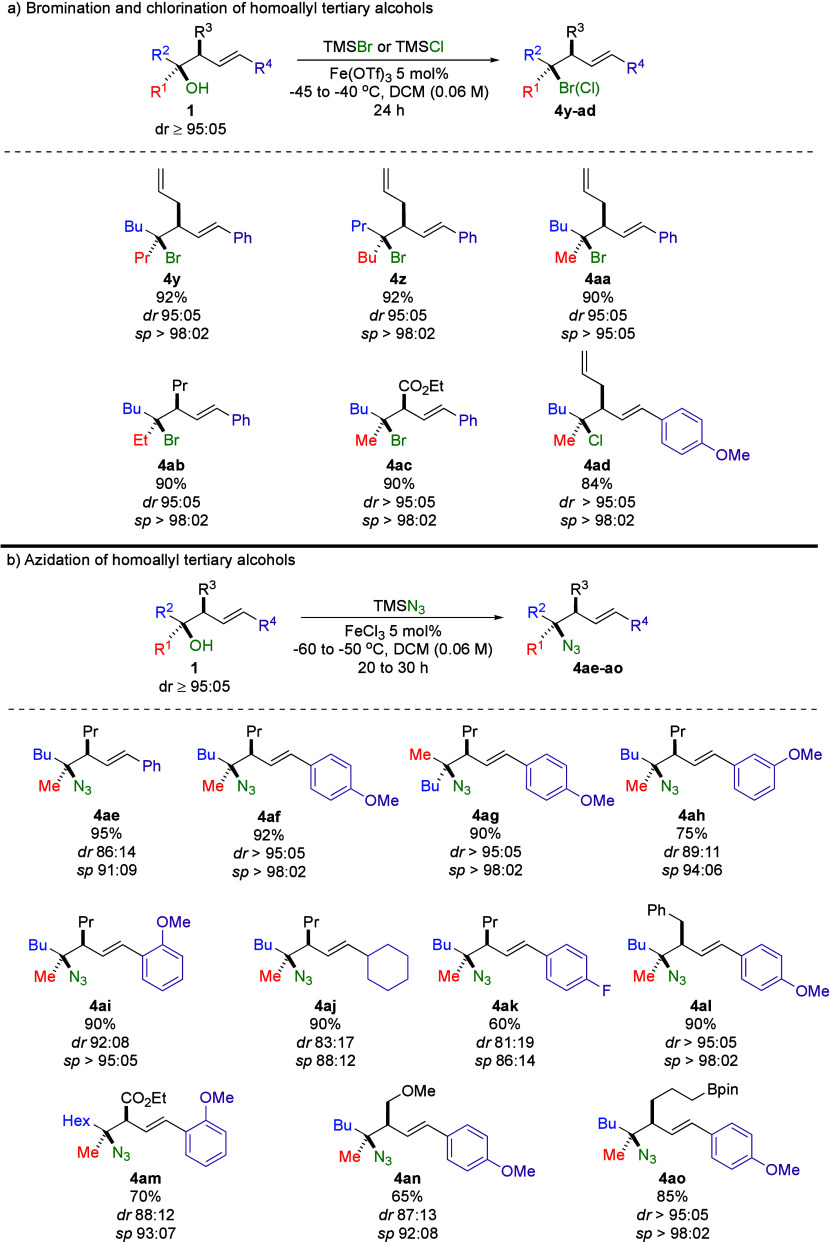
Fe-Catalyzed Stereoretentive S_N_1 Bromination, Chlorination, and Azidation Reactions

This transformation was also diastereospecific, as **4y** and **4z** could be obtained with identical stereoisomeric purity by simply permuting the stereochemistry of the two substituents at the tertiary alcohol center in the starting materials. Furthermore, modifying the R^1^ substituent from allyl to propyl or ester did not alter the outcome of the transformation (**4ab** and **4ac**, Scheme 4a). Under the same optimized conditions, substituting the nucleophile TMSBr with TMSCl, provided the tertiary homoallylic chloride (**4ad**, Scheme 4a) with excellent yields (84%) and exceptional diastereoselectivity (*dr* > 95:05, *sp* > 98:02).

Under optimized conditions (details available in Supporting Information), the FeCl_3_-catalyzed addition of TMSN_3_ enabled, at low temperatures, the formation of the desired tertiary homoallylic azides (**4ae**–**4ao**, Scheme 4b) with excellent yields and selectivities for a range of substituted substrates, providing a potential entry to β-amino acid with an alpha-tertiary amine through oxidative cleavage of the remaining double bond.

Notably, when the R^4^ substituent is a para-substituted anisole, the resulting azide (**4af**, Scheme 4b) was obtained with an outstanding yield and diastereoselectivity (92%, *dr* > 95:05, *sp* > 98:02), highlighting the significant role of the electron-donating OMe group in stabilizing the cyclopropyl carbinyl intermediate **C**. The reaction proved diastereospecific, as both **4af** and **4ag** could be prepared with stereoisomeric purity identical to that of their respective diastereomeric tertiary homoallyl alcohol precursors. Moreover, substituting the R^4^ group from phenyl to cyclohexyl (**4aj**, Scheme 4b) or introducing an electron-withdrawing fluoro group (**4ak**, Scheme 4b) led to a modest decrease in the diastereomeric ratio. Importantly, the transformation is not restricted to the presence of a propyl group at R^1^, and various substituents such as benzyl, CO_2_Et, and CH_2_OMe are also compatible. Additionally, the reaction tolerates functional groups like Bpin, as demonstrated in the synthesis of **4ao**, (Scheme 4b), underscoring its broad functional group compatibility.

After successfully establishing stereoretentive nucleophilic halogenation and azidation of homoallylic tertiary alcohols, we turned our attention to exploring their thiocyanation. Simply substituting the nucleophile TMSN_3_ with TMSNCS under the same reaction conditions but at room temperature resulted in the desired products in good yield and diastereoselectivity. However, we soon observed that the outcomes were highly substrate-dependent. To improve the diastereoselectivity, we transformed the tertiary alcohols with a more effective leaving group, such as phosphates **5** (Scheme 5).

**Scheme 5:**
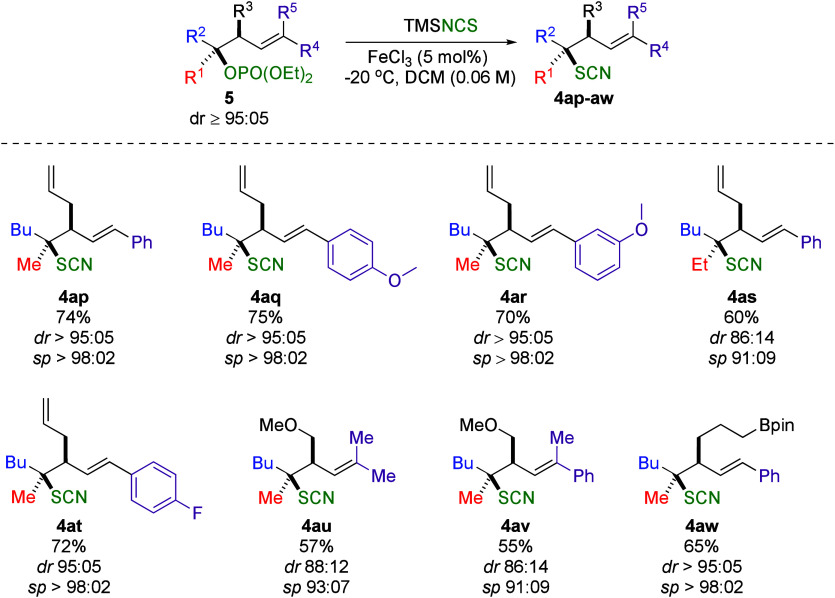
Fe-Catalyzed Stereoretentive S_N_1 Thiocyanation

This modification allowed the reaction to proceed at −20 °C, consistently delivering high specificity (Scheme 5). When R^4^ = R^5^ = CH_3_ (**4au**, Scheme 5) or R^4^ = Ph, R^5^ = CH_3_ (**4av**, Scheme 5), the reaction still proceeded but with lower yields and diastereoselectivity.

In a noteworthy transformation, thiocyanate **4ap** was converted into tertiary thiol **6a** upon treatment with lithium aluminum hydride (LAH) in THF (0.1 M) at room temperature.^23^ This transformation enables the overall stereoretentive conversion of tertiary alcohol derivatives into tertiary thiols (Scheme 6).^14e^

**Scheme 6:**
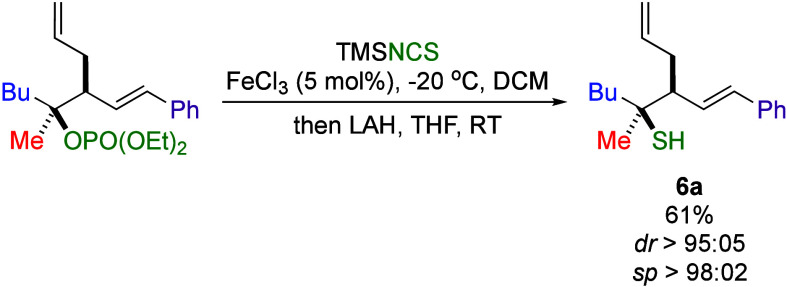
From Tertiary Alcohols to Tertiary Thiol

In conclusion, stereoretentive nucleophilic substitution at tertiary homoallylic alcohols in acyclic systems is achieved with full stereochemical control via an S_N_1-type mechanism. The key to this transformation is the selective formation and reactivity of the cyclopropyl carbinyl cation intermediate, which facilitates the targeted cleavage of C–OH bonds and the formation of new C-X bonds. This methodology has been successfully applied to generate homoallylic tertiary fluorides, chlorides, bromides, azides, and thiocyanates, demonstrating its versatility in accessing diverse functional groups with exceptional diastereoselectivity.

## Supplementary Material



## Data Availability

The data underlying this study are available in the published article and its Supporting Information.

## Notes

General procedures, product characterization, copies of NMR spectra (PDF)
