# Supplementary material for: Formally Stereoretentive SN1 Reactions of Homoallylic Tertiary Alcohols Via Nonclassical Carbocation
Source: J Am Chem Soc. 2025 May 27;147(23):19478–84. doi: 10.1021/jacs.5c05680 (PMC12164358; doi:10.1021/jacs.5c05680)
Supplement: Supplementary file 1 [file ja5c05680_si_001.pdf]

Supporting Information

Stereoretentive S_N1 Reactions of Homoallylic Tertiary Alcohols via Non-classical Carbocation

Kaushalendra Patel, Leonie Wilczek, Francesco Calogero, Ilan Marek*

Schulich Faculty of Chemistry and the Resnick Sustainability Center for Catalysis, Technion - Israel Institute of Technology, Technion City, Haifa 3200009, Israel

Table of contents

1	General experimental details	p. 3
2	Synthesis of starting materials	p. 4
3	Optimization Table	p. 5
4	Experimental procedures	p. 6
5	Characterization data for starting materials	p. 8
6	Determination of relative stereochemistry	p. 17
7	Characterization data for products	p. 17
8	Characterization spectra for starting materials	p. 33
9	Characterization spectra for products	p. 69
10	References	p. 133

1. General experimental details

Unless stated otherwise, reactions were conducted in flame-dried glassware under a positive pressure of argon. Ether and THF were dried from Pure-Solv® Purification System (Innovative Technology©). All other commercially obtained reagents were used as received. Dichloromethane was distilled from CaH_2. Copper iodide, DAST, $FeCl_3$, $B(C_6F_5)_3$, TMSX and other chemicals were purchased from Aldrich. All alkyl Grignard reagents were prepared from the corresponding alkyl bromides. Thin-layer chromatography (TLC) was conducted with Merck silica gel 60 F254 pre-coated plates (0.25 mm) and visualized by exposure to UV light (254 nm) or stained with anisaldehyde, phosphomolybdic acid, or potassium permanganate. Column chromatography was performed using Fluka silica gel 60Å (40-63mm, 230-400 mesh). ^{1}H-NMR, ^{13}C-NMR and other NMR spectra were recorded on a Bruker© spectrometers AVIII400 or AVIII500, using $CDCl_3$ as solvent. Chemical shifts are reported in parts per million (ppm) with respect to the residual solvent signal $CDCl_3$ (^{1}H NMR: δ = 7.26 ppm; ^{13}C NMR: δ = 77.00 ppm). Peak multiplicities are reported as follows: s = singlet, bs = broad singlet, d = doublet, t = triplet, dd = doublet of doublets, td = triplet of doublets, m = multiplet. High-resolution mass spectra (HRMS) were obtained by the mass spectrometry facility at the Technion-Israel Institute of Technology.

Synthesis of starting materials

The homoallyl tertiary alcohols were prepared according to the previous method developed by our group.[1a] Homoallylic alcohols 1a, 1b, 1d, 1f, 1g, 1h, 1n, 1s, 1t, 1y, 1z, 1aa, 1ab are known compounds, prepared according to the methods reported by our group.[1a]

Scheme S1: List of homoallyl tertiary alcohols as starting materials.

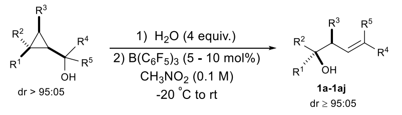

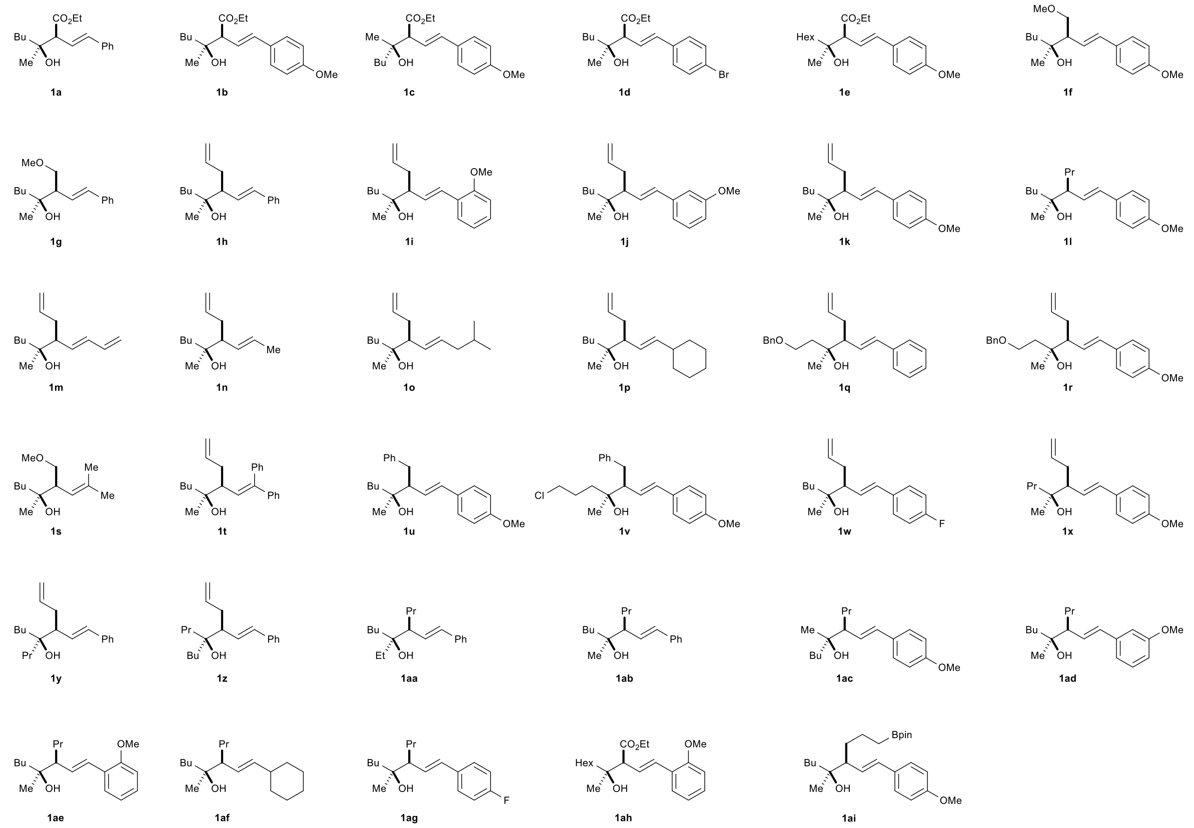

General procedure A: Synthesis of homoallylic tertiary alcohols[1a]

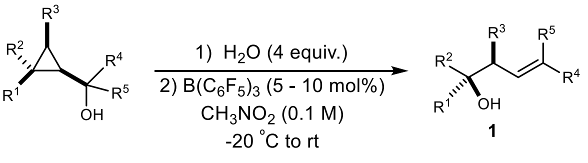

To a stirred solution of cyclopropyl carbinols (1.0 equiv., 0.4 mmol-0.6 mmol) in CH_3NO_2 (0.1M) at -20 °C under Argon atmosphere was added H_2O (4 equiv.) followed by addition of $B(C_6F_5)_3$ (5 - 10 mol%). The resulting mixture was then stirred at -20 °C to RT until the complete consumption of starting material, the reaction being monitored by TLC (stained with PMA) (typically 12 h). Then, the reaction mixture was quenched with Et_3N. The volatilities were removed under reduced pressure to give crudes **1** which were purified by flash column chromatography on silica gel (20% Et_2O/PE as eluent).

Note: For the cyclopropyl carbinols having R^3 = $-CO_2Et$ and -OMe the reactions were performed at RT.

General procedure B: Synthesis of homoallylic tertiary phosphates

Phosphorylation of homoallyl secondary alcohols was performed utilizing products derived via general procedures **A**.

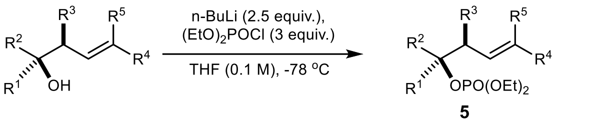

5
dr ≥ 95:05

To a stirred solution of homoallylic tertiary alcohols **1** (1 mmol) in dry THF (0.1 M) at -78 °C under argon atmosphere was added *n*-BuLi (2.5 mmol). The reaction mixture was stirred for around 1 hour while maintaining the temperature between -78 °C to -70 °C. Then to this solution was slowly added diethyl chlorophosphate (3 mmol) at -78 °C. The resulting mixture was then slowly warmed to 0 °C until complete consumption of the starting materials (typically 2 hours), monitored by TLC (stained with PMA). Further, an aqueous solution of NH_4Cl was added and the aqueous layer was extracted twice with Et_2O. Combined organic layers were washed with brine, dried on Na_2SO_4, filtered, and concentrated under vacuum to give the crude phosphates, which were purified by column chromatography on silica gel using 70-80% diethyl ether in petroleum ether as eluent.

Scheme S2: List of homoallyl tertiary phosphates as starting materials.

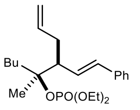

5a
90%

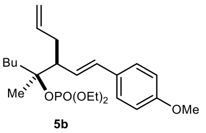

5b
92%

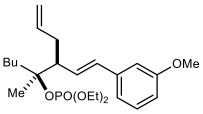

5c
88%

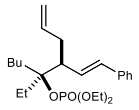

5d
86%

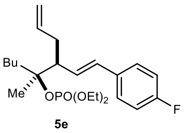

5e
82%

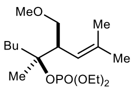

5f
82%

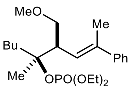

5g
85%

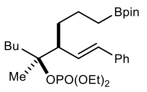

5h
90%

Optimization Table (i)

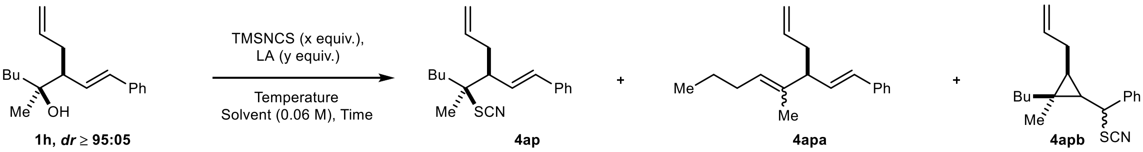

1h, ***dr*** **≥ 95:05** **4ap** **4apa** **4apb**

S. N.	Catalyst	(equ iv.)	Solvent	T	TMSNCS (equiv.)	Time (h)	1h + 4ap[a] (*dr*[b]) + 4apa + 4apb
1	$BF_3.OEt_2$	0.05	Toluene	0 °C - RT	5	16	Complex mixture
2	$InBr_3$	0.05	DCM	0 °C - RT	5	20	00 + 81 (88:12) + 15 + 4
3	$BF_3.OEt_2$	0.05	DCM	0 °C - RT	5	16	00 + 72 (92:08) + 08 + 20
4	$InBr_3$	0.05	DCM	0 °C - RT	5	07	00 + 74 (70:30) + 08 + 18
5	In $(OTf)_3$	0.05	DCM	RT	5	06	00 + 47 (> 95:05) + 42 + 11
6	$InBr_3$	0.05	DCM	-20 °C - RT	5	60	20 + 68 (82:18) + 6 + 6
7	$InBr_3$	0.05	DCE	-20 °C - RT	5	60	18 + 72 (80:20) + 3 + 7
8	$InBr_3$	0.05	Toluene	0 °C - RT	5	14	Complex mixture
9	$In(OTf)_3$	0.05	DCM	0 °C - RT	5	48	27 + 28 (> 95:05) + 37 + 08
10	$Fe(OTf)_3$	0.05	DCM	0 °C - RT	5	48	39 + 19 (> 95:05) + 42 + ND
11	$In(OTf)_3$	0.05	DCE	RT	5	05	00 + 42 (> 95:05) + 48 + 10

12	$FeCl_3$	0.05	DCM	RT	5	04	00 + 80 (70)^c (92:08) + 07 + 13
13	FeCl3	0.05	DCM	RT	3	06	00 + 72 (92:08) + 15 + 13
14	$In(OTf)_3$	0.05	DCE	0 °C - RT	5	22	45 + 35 (> 95:05) + 16 + 04
15	$In(OTf)_3$	0.1	DCE	0 °C - RT	5	22	05 + 53 (> 95:05) + 34 + 10
16	$FeBr_3$	0.05	DCM	RT	5	03	00 + 79 (92:08) + 05 + 16
17	$FeBr_2$	0.05	DCM	RT	5	24	30 + 60 (92:08) + 02 + 08
18	$FeCl_3$	0.05	DCM (0.1 M)	RT	5	12	10 + 76 (92:08) + 03 + 11
19	$FeCl_3$	0.05	DCM (0.03 M)	RT	5	12	13 + 69 (92:08) + 03 + 15

^{a} Yield determined by ^{1}H NMR using CH_2Br_2 as internal standard. ^{b} *dr* determined by ^{13}C NMR. ^{c} Yield of isolated products.

Optimization Table (ii)

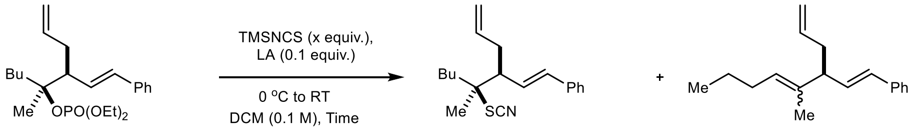

5a, *dr* ≥ 95:05 — 4ap — 4apa

S. N.	Catalyst	Solvent	T	TMSNCS (equiv.)	Time (h)	5a + 4ap^a (dr^b) + 4apa
1	$InBr_3$	DCM	- 20 °C	3	14	00 + 67 (> 95:05) + 33
2	$InBr_3$	DCM	-78 °C to RT	3	72	00 + 75 (> 95:05) + 22
3	$InBr_3$	DCM	-45 °C to RT	3	72	00 + 76 (> 95:05) + 24
4	$FeCl_3$	DCM	-30 °C to RT	3	30	00 + 76 (> 95:05) + 24
5	$FeCl_3$	DCM	-20 °C	3	16	00 + 82 (74)^c (> 95:05) + 18
6	$FeCl_3$	DCM	-20 °C	5	14	00 + 82 (> 95:05) + 18
7	$FeCl_3$	DCM	-20 °C	1	24	24 + 50 (> 95:05) + 26
8	$FeCl_3$	DCM	-20 °C	2	24	21 + 56 (> 95:05) + 23
9	$FeCl_3$ (0.1 Equiv.)	DCM	-20 °C	3	12	00 + 75 (> 95:05) + 25

^{a} Yield determined by ^{1}H NMR using CH_2Br_2 as internal standard. ^{b} *dr* determined by ^{13}C NMR. ^{c} Yield of isolated products.

Experimental procedures

General procedure C: Nucleophilic fluorination

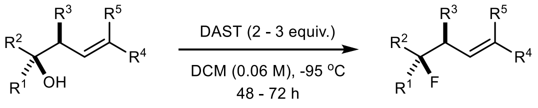

1 — 4a-x

To a stirred solution of homoallyl tertiary alcohols **1** (0.2 mmol to 0.4 mmol) in dry DCM (0.06 M) at -95 °C under argon, diethylaminosulfur trifluoride (2 equiv. to 3 equiv.) was added dropwise. The resulting mixture was then stirred at -95 °C until complete consumption of the starting materials, with progress monitored by TLC (stained with PMA). After completion of the reaction (typically 2 - 3 days), it was quenched with a saturated aqueous solution of $NaHCO_3$. The reaction mixture was then extracted twice with DCM (10 mL), dried over Na_2SO_4, filtered, and concentrated under vacuum to give the crude product. Further purification was achieved by column chromatography on silica gel using pure petroleum ether or diethyl ether in petroleum ether as the eluent.

Note: For the homoallylic tertiary alcohols having R^3 = -CO_2Et and -OMe the reaction temperature was raised to -80 °C after addition of DAST.

General procedure D: Nucleophilic bromination/chlorination

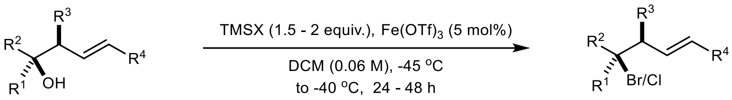

1 **4y-ad**

To a stirred solution of homoallyl alcohols **1** (0.2 mmol-0.4 mmol) in dry DCM (0.06 M) at -45 °C under argon, $Fe(OTf)_3$ (0.05 equiv.) and TMSBr or TMSCl (1.5 - 2 equiv.) were added sequentially. The resulting mixture was then stirred at -45 °C to -40 °C until complete consumption of the starting materials, with progress monitored by TLC (stained with PMA). After completion of the reaction (typically 24 to 48 h), it was quenched with a saturated aqueous solution of NH_4Cl. The reaction mixture was then extracted twice with DCM (10 mL), dried over Na_2SO_4, filtered, and concentrated under vacuum to give the crude product. Further purification was achieved by column chromatography on silica gel using pure petroleum ether or diethyl ether in petroleum ether as the eluent for chlorination (as homoallyl tertiary bromides are very sensitive towards silica, purification was performed for them only if needed). [2]

Note: Changing the Lewis acid from $Fe(OTf)_3$ to $FeCl_3$ does not significantly alter the transformation. For Bromination (**4ac,** R^3 = **-**CO_2Et), reaction was performed at room temperature with TMSBr (3 equiv.) over 4 h.

General procedure E: Nucleophilic azidation

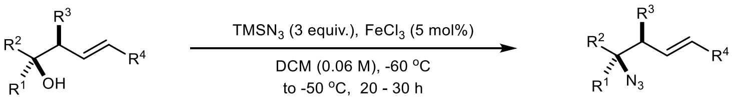

1 **4ae-ao**

To a stirred solution of homoallyl alcohols **1** (0.2 mmol-0.4 mmol) in dry DCM (0.06 M) at -60 °C under argon, $FeCl_3$ (0.05 equiv.) and $TMSN_3$ (3 equiv.) were added sequentially. The resulting mixture was then stirred at -60 °C to -50 °C until complete consumption of the starting materials, with progress monitored by TLC (stained with PMA). After completion of the reaction (typically 20 to 30 h), it was quenched with a saturated aqueous solution of NH_4Cl. The reaction mixture was then extracted twice with DCM (10 mL), dried over Na_2SO_4, filtered, and concentrated under vacuum to give the crude product. Further purification was achieved by column chromatography on silica gel using pure petroleum ether or diethyl ether in petroleum ether as the eluent.

Note: For azidation 4am (R^1 = -CO_2Et), and 4an (R^1 = -OMe), the reactions were performed at RT. For 4am (R^1 = -CO_2Et), reaction was performed with $InBr_3$ instead of $FeCl_3$ (With $FeCl_3$ it gives TMS protected alcohol).

General procedure F: Nucleophilic thiocyanation from phosphates

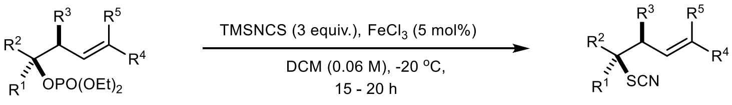

5 **4ap-aw**

To a stirred solution of homoallyl phosphates **5** (0.2 mmol-0.4 mmol) in dry DCM (0.06 M) at -20 °C under argon, TMSNCS (3 equiv.) and $FeCl_3$ (0.05 equiv.) were added sequentially. The resulting mixture was then stirred at -20 °C until complete consumption of the starting materials, with progress monitored by TLC (stained with PMA). After completion of the reaction (typically 15 to 20 h), it was quenched with a saturated aqueous solution of NH_4Cl. The reaction mixture was then extracted twice with DCM (10 mL), dried over Na_2SO_4, filtered, and

concentrated under vacuum to give the crude product. Further purification was achieved by column chromatography on silica gel using pure petroleum ether or diethyl ether in petroleum ether as the eluent.

Characterization data for starting materials

Ethyl (2*S,3*R**)-3-hydroxy-2-((*E*)-4-methoxystyryl)-3-methylheptanoate (1c)**

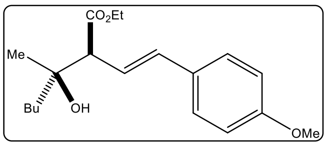

The title compound was prepared according to the general procedure A. The product was obtained as a colourless liquid (75%, 450 mg from 600 mg, *dr* = 94:06). R_f = 0.5 (petroleum ether /Et_2O = 6/4). ^{1}H NMR (400 MHz, Chloroform-*d*) δ 7.33 (d, *J* = 8.8 Hz, 2H), 6.85 (d, *J* = 8.7 Hz, 2H), 6.47 (d, *J* = 15.9 Hz, 1H), 6.23 (dd, *J* = 15.9, 9.7 Hz, 1H), 4.27 – 4.11 (m, 2H), 3.79 (s, 3H), 3.44 (s, 1H), 3.18 (d, *J* = 9.7 Hz, 1H), 1.54 – 1.41 (m, 2H), 1.28 (t, *J* = 7.1 Hz, 7H), 1.24 (s, 3H), 0.88 (t, *J* = 7.0 Hz, 3H); ^{13}C NMR (101 MHz, Chloroform-*d*) δ 174.12, 159.28, 133.91, 129.39, 127.58, 121.55, 113.90, 73.36, 60.86, 58.14, 55.21, 38.91, 25.57, 25.48, 23.09, 14.09, 14.01; **HRMS** (APCI) [M+H]$^+$, calculated for $C_{19}H_{29}O_4$; 321.2066; found 321.2085.

Ethyl (2*S,3*S**)-3-hydroxy-2-((*E*)-4-methoxystyryl)-3-methylnonanoate (1e)**

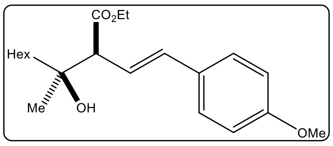

The title compound was prepared according to the general procedure A. The product was obtained as a colourless liquid (72%, 540 mg from 750 mg, *dr* > 95:05). R_f = 0.5 (petroleum ether /Et_2O = 6/4). ^{1}H NMR (400 MHz, Chloroform-*d*) δ 7.33 (d, *J* = 8.4 Hz, 2H), 6.85 (d, *J* = 8.4 Hz, 2H), 6.46 (d, *J* = 15.8 Hz, 1H), 6.22 (dd, *J* = 15.8, 9.7 Hz, 1H), 4.28 – 4.11 (m, 2H), 3.80 (s, 3H), 3.35 (s, 1H), 3.18 (d, *J* = 9.6 Hz, 1H), 1.50 (dd, *J* = 9.0, 6.8 Hz, 2H), 1.45 – 1.34 (m, 2H), 1.28 (q, *J* = 5.3, 3.4 Hz, 9H), 1.16 (s, 3H), 0.88 (t, *J* = 6.6 Hz, 3H); ^{13}C NMR (101 MHz, Chloroform-*d*) δ 174.21, 159.29, 133.94, 129.43, 127.61, 121.82, 113.93, 73.80, 60.91, 58.14, 55.27, 41.68, 31.74, 29.74, 23.77, 23.62, 22.55, 14.15, 14.04; **HRMS** (APCI) [M+H]$^+$, calculated for $C_{21}H_{33}O_4$; 349.2379; found 349.2387.

(4*S,5*S**)-4-((*E*)-2-methoxystyryl)-5-methylnon-1-en-5-ol (1i)**

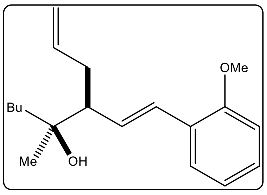

The title compound was prepared according to the general procedure A. The product was obtained as a colourless liquid (65%, 325 mg from 500 mg, *dr* = 95:05). R_f = 0.5 (petroleum ether /Et_2O = 7/3). ^{1}H NMR (400 MHz, Chloroform-*d*) δ 7.44 (dd, *J* = 7.7, 1.7 Hz, 0H), 7.33 – 7.17 (m, 0H), 6.93 (td, *J* = 7.5, 1.1 Hz, 0H), 6.87 (dd, *J* = 8.2, 1.1 Hz, 0H), 6.74 (d, *J* = 16.0 Hz, 0H), 6.02 (dd, *J* = 16.0, 9.6 Hz, 0H), 5.81 (ddt, *J* = 17.1, 10.1, 6.9 Hz, 0H), 5.28 – 4.70 (m, 1H), 3.84 (s, 1H), 2.51 (dddd, *J* = 13.8, 7.1, 3.3, 1.5 Hz, 0H), 2.33 (td, *J* = 10.1, 3.2 Hz, 0H), 2.17 (dddd, *J* = 13.3, 10.6, 5.3, 1.3 Hz, 0H), 1.61 – 1.39 (m, 1H), 1.38 – 1.23 (m, 1H), 1.20 (s, 1H); ^{13}C NMR (101 MHz, Chloroform-*d*) δ 156.43, 137.92, 130.74, 128.18, 127.53, 126.45, 126.41, 120.52, 115.42, 110.91, 74.33, 55.44, 53.92, 39.98, 34.00, 25.41, 24.19, 23.28, 14.12; **HRMS** (APCI) [M+H]$^+$, calculated for $C_{19}H_{29}O_2$; 289.2168; found 289.2149.

(4*S,5*S**)-4-((*E*)-3-methoxystyryl)-5-methylnon-1-en-5-ol (1j)**

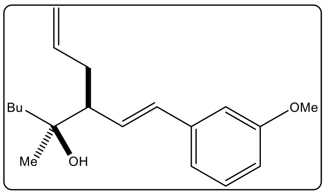

The title compound was prepared according to the general procedure A. The product was obtained as a colourless liquid (70%, 420 mg from 600 mg, *dr* = 95:05). R_f = 0.5 (petroleum ether /Et_2O = 7/3). ^{1}H NMR (400 MHz, Chloroform-*d*) δ 7.32 – 7.26 (m, 1H), 7.05 – 6.99 (m, 1H), 6.95 (dd, *J* = 2.6, 1.6 Hz, 1H), 6.83 (ddd, *J* = 8.2, 2.6, 0.9 Hz, 1H), 6.42 (d, *J* = 15.8 Hz, 1H), 6.07 (dd, *J* = 15.8, 9.6 Hz, 1H), 5.81 (ddt, *J* = 17.0, 10.0, 7.0 Hz, 1H), 5.13 – 4.99 (m, 2H), 3.87 (s, 3H), 2.56 (dddt, *J* = 13.7, 7.1, 3.0, 1.3 Hz, 1H), 2.33 (td, *J* = 10.1, 3.1 Hz, 1H), 2.23 – 2.13 (m, 1H), 1.60 – 1.44 (m, 4H), 1.39 – 1.30 (m, 3H), 1.23 (s, 3H), 0.96 (t, *J* = 7.0 Hz, 3H); ^{13}C NMR (101 MHz, Chloroform-*d*) δ 159.78, 138.77, 137.69, 132.68, 130.65, 129.49, 118.82, 115.67, 112.67, 111.64, 74.36, 55.22, 53.62, 40.19, 33.86, 25.45, 24.17, 23.27, 14.15; **HRMS** (APCI) $[M+H]^+$, calculated for $C_{19}H_{29}O_2$; 289.2168; found 289.2154.

(4*S,5*S**)-4-((*E*)-4-methoxystyryl)-5-methylnon-1-en-5-ol (1k)**

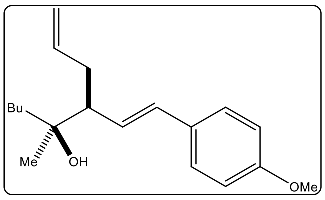

The title compound was prepared according to the general procedure A. The product was obtained as a colourless liquid (80%, 480 mg from 600 mg, *dr* > 95:05). R_f = 0.5 (petroleum ether /Et_2O = 7/3). ^{1}H NMR (400 MHz, Chloroform-*d*) δ 7.31 (d, *J* = 8.2 Hz, 2H), 6.86 (d, *J* = 8.2 Hz, 2H), 6.35 (d, *J* = 15.8 Hz, 1H), 5.88 (dd, *J* = 15.8, 9.5 Hz, 1H), 5.76 (dt, *J* = 11.6, 8.4 Hz, 1H), 5.03 (d, *J* = 17.1 Hz, 1H), 4.96 (d, *J* = 10.1 Hz, 1H), 3.81 (d, *J* = 1.7 Hz, 3H), 2.50 (dd, *J* = 14.4, 7.1 Hz, 1H), 2.31 – 2.21 (m, 1H), 2.13 (td, *J* = 12.1, 11.3, 6.9 Hz, 1H), 1.53 – 1.27 (m, 7H), 1.19 (s, 3H), 0.91 (t, *J* = 6.8 Hz, 3H); ^{13}C NMR (101 MHz, Chloroform-*d*) δ 158.96, 137.86, 132.26, 130.16, 128.04, 127.27, 115.51, 113.94, 74.38, 55.31, 53.76, 40.03, 34.00, 25.46, 24.17, 23.30, 14.14; **HRMS** (APCI) $[M-OH]^+$, calculated for $C_{19}H_{27}O$; 271.2056; found 271.2086.

(4*S,5*S**)-4-((*E*)-4-methoxystyryl)-5-methylnonan-5-ol (1l)**

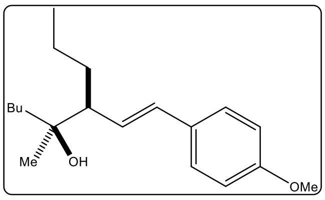

The title compound was prepared according to the general procedure A. The product was obtained as a colourless liquid (80%, 576 mg from 720 mg, *dr* = 95:05). R_f = 0.5 (petroleum ether /Et_2O = 7/3). ^{1}H NMR (400 MHz, Chloroform-*d*) δ 7.31 (d, *J* = 8.7 Hz, 2H), 6.86 (d, *J* = 8.7 Hz, 2H), 6.36 (d, *J* = 15.8 Hz, 1H), 5.86 (dd, *J* = 15.8, 9.8 Hz, 1H), 3.81 (s, 3H), 2.23 – 2.08 (m, 1H), 1.66 – 1.57 (m, 1H), 1.52 – 1.37 (m, 5H), 1.37 – 1.24 (m, 5H), 1.17 (s, 3H), 0.91 (td, *J* = 7.0, 3.0 Hz, 6H); ^{13}C NMR (101 MHz, Chloroform-*d*) δ 158.87, 132.13, 130.21, 128.79, 127.19, 113.92, 74.28, 55.28, 54.09, 39.71, 31.25, 25.47, 24.27, 23.33, 21.26, 14.15, 14.11; **HRMS** (APCI) $[M-OH]^+$, calculated for $C_{19}H_{29}O$: 273.2168; found 273.2213.

(5*S,6*S**,*E*)-6-allyl-5-methyldeca-7,9-dien-5-ol (1m)**

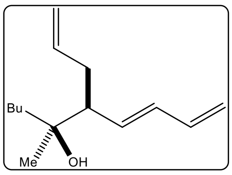

The title compound was prepared according to the general procedure A. The product was obtained as a colourless liquid (78%, 468 mg from 600 mg, *dr* = 95:05). R_f = 0.5 (petroleum ether /Et_2O = 7/3). ^1H NMR (400 MHz, Chloroform-*d*) δ 6.32 (dt, *J* = 16.9, 10.3 Hz, 1H), 6.06 (dd, *J* = 15.2, 10.4 Hz, 1H), 5.72 (ddt, *J* = 16.9, 10.1, 6.8 Hz, 1H), 5.50 (dd, *J* = 15.2, 9.5 Hz, 1H), 5.14 (dd, *J* = 17.0, 1.7 Hz, 1H), 5.06 – 4.79 (m, 3H), 2.60 – 2.36 (m, 1H), 2.15 (td, *J* = 10.0, 2.9 Hz, 1H), 2.04 (ddd, *J* = 13.5, 10.5, 6.6 Hz, 1H), 1.48 – 1.25 (m, 7H), 1.13 (s, 3H), 0.91 (t, *J* = 6.8 Hz, 3H); ^{13}C NMR (101 MHz, Chloroform-*d*) δ 137.74, 136.86, 134.51, 133.94, 115.86, 115.55, 74.29, 53.20, 40.06, 33.78, 25.40, 24.06, 23.27, 14.12; **HRMS** (APCI) $[M-H]^+$, calculated for $C_{14}H_{23}O$; 207.1743; found 207.1743.

(5*S,6*S**,*E*)-6-allyl-5,10-dimethylundec-7-en-5-ol (1o)**

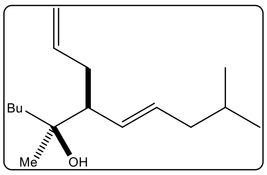

The title compound was prepared according to the general procedure A. The product was obtained as a colourless liquid (77%, 347 mg from 451 mg, *dr* = 95:05). R_f = 0.5 (petroleum ether /Et_2O = 7/3). ^1H NMR (400 MHz, Chloroform-*d*) δ 5.78 (ddt, *J* = 16.9, 10.1, 6.8 Hz, 1H), 5.54 – 5.41 (m, 1H), 5.22 (dd, *J* = 15.3, 9.1 Hz, 1H), 5.07 – 4.95 (m, 2H), 2.54 – 2.34 (m, 1H), 2.16 – 2.01 (m, 2H), 1.96 (t, *J* = 6.9 Hz, 2H), 1.68 – 1.62 (m, 1H), 1.52 – 1.39 (m, 4H), 1.39 – 1.31 (m, 3H), 1.16 (s, 3H), 0.94 (dd, *J* = 14.5, 6.7 Hz, 9H); ^{13}C NMR (101 MHz, Chloroform-*d*) δ 138.20, 133.04, 130.87, 115.18, 73.97, 53.44, 42.11, 39.82, 33.91, 28.45, 25.32, 24.01, 23.33, 22.33, 22.23, 14.12; **HRMS** (APCI) $[M-H]^+$, calculated for $C_{16}H_{29}O$; 237.2213; found 237.2220.

(4*S,5*S**)-4-((*E*)-2-cyclohexylvinyl)-5-methylnon-1-en-5-ol (1p)**

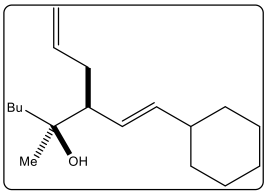

The title compound was prepared according to the general procedure A. The product was obtained as a colourless liquid (75%, 126 mg from 280 mg, *dr* = 95:05). R_f = 0.5 (petroleum ether /Et_2O = 7/3). ^1H NMR (400 MHz, Chloroform-*d*) δ 5.71 (ddt, *J* = 16.7, 10.3, 6.7 Hz, 1H), 5.40 (dd, *J* = 15.4, 6.8 Hz, 1H), 5.13 (dd, *J* = 15.5, 8.7 Hz, 1H), 5.02 – 4.86 (m, 2H), 2.47 – 2.30 (m, 1H), 1.99 (dtd, *J* = 16.9, 11.4, 6.9 Hz, 3H), 1.72 – 1.59 (m, 5H), 1.47 – 1.38 (m, 3H), 1.37 – 1.13 (m, 7H), 1.12 – 0.99 (m, 5H), 0.90 (t, *J* = 6.8 Hz, 3H); ^{13}C NMR (101 MHz, Chloroform-*d*) δ 140.43, 138.14, 126.96, 115.11, 73.92, 53.21, 40.88, 39.69, 33.92, 33.26, 33.16, 26.14, 25.97, 25.28, 24.03, 23.32, 14.13; **HRMS** (APCI) $[M-H]^+$, calculated for $C_{18}H_{31}O$; 263.2375; found 263.2348.

(3*S,4*S**)-1-(benzyloxy)-3-methyl-4-((*E*)-styryl)hept-6-en-3-ol (1q)**

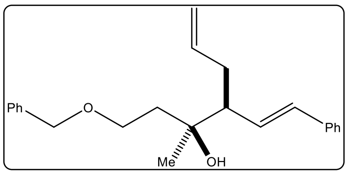

The title compound was prepared according to the general procedure A. The product was obtained as a colourless liquid (66%, 396 mg from 600 mg, *dr* = 95:05). R_f = 0.5 (petroleum ether /Et_2O = 7/3). ^1H NMR (400 MHz, Chloroform-*d*) δ 7.36 – 7.23 (m, 9H), 7.20 – 7.14 (m, 1H), 6.25 (d, *J* = 15.8 Hz, 1H), 5.92 (dd, *J* = 15.8, 9.7 Hz, 1H), 5.72 (ddt, *J* = 17.1, 10.1, 7.0 Hz, 1H), 5.00 (dd, *J* = 17.2, 2.0 Hz, 1H), 4.93 (dd, *J* = 10.1, 2.0 Hz, 1H), 4.58 – 4.37 (m, 2H), 3.69 (dddd, *J* = 25.9, 9.7, 6.6, 5.1 Hz, 2H), 3.25 (s, 1H), 2.72 – 2.55 (m, 1H), 2.26 (td, *J* = 10.3, 2.7 Hz, 1H), 2.12 – 1.98 (m, 1H), 1.89 – 1.72 (m, 2H), 1.16 (s, 3H); ^{13}C NMR

(101 MHz, Chloroform-*d*) δ 137.76, 137.53, 137.20, 132.51, 130.34, 128.40, 127.78, 127.08, 126.01, 115.44, 73.92, 73.36, 67.04, 53.80, 39.04, 33.20, 22.99; **HRMS** (APCI) $[M+H]^+$, calculated for $C_{23}H_{29}O_2$; 337.2162; found 337.2173.

(3*S,4*S**)-1-(benzyloxy)-4-((*E*)-4-methoxystyryl)-3-methylhept-6-en-3-ol (1r)**

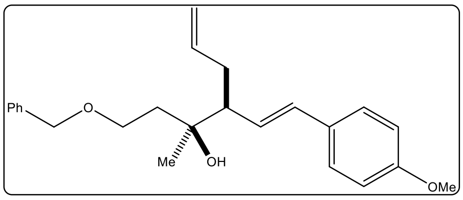

The title compound was prepared according to the general procedure A. The product was obtained as a colourless liquid (68%, 408 mg from 600 mg, *dr* = 95:05). R_f = 0.5 (petroleum ether /Et_2O = 7/3). ^1H NMR (400 MHz, Chloroform-*d*) δ 7.28 – 7.12 (m, 5H), 7.10 (d, *J* = 8.6 Hz, 2H), 6.68 (d, *J* = 8.6 Hz, 2H), 6.07 (d, *J* = 15.8 Hz, 1H), 5.70 – 5.51 (m, 2H), 4.86 (dd, *J* = 17.2, 2.0 Hz, 1H), 4.79 (dd, *J* = 10.0, 2.1 Hz, 1H), 4.48 – 4.22 (m, 2H), 3.62 (s, 3H), 3.55 (dddd, *J* = 16.3, 9.6, 5.9, 3.1 Hz, 2H), 3.16 (s, 1H), 2.56 – 2.45 (m, 1H), 2.10 (td, *J* = 10.3, 2.7 Hz, 1H), 1.97 – 1.84 (m, 1H), 1.75 – 1.61 (m, 2H), 1.03 (s, 3H); ^{13}C NMR (101 MHz, Chloroform-*d*) δ 158.81, 137.90, 137.57, 131.89, 130.03, 128.37, 128.09, 127.76, 127.74, 127.11, 115.32, 113.81, 73.93, 73.34, 67.08, 55.17, 53.84, 39.01, 33.28, 22.97; **HRMS** (APCI) $[M+H]^+$, calculated for $C_{24}H_{31}O_3$; 367.2268; found 367.2268.

(3*S,4*S**,*E*)-3-benzyl-1-(4-methoxyphenyl)-4-methyloct-1-en-4-ol (1u)**

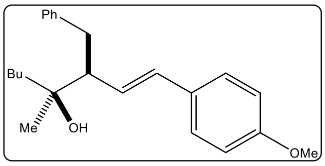

The title compound was prepared according to the general procedure A. The product was obtained as a colourless liquid (80%, 440 mg from 550 mg, *dr* > 95:05). R_f = 0.5 (petroleum ether /Et_2O = 7/3). ^1H NMR (400 MHz, Chloroform-*d*) δ 7.14 – 7.06 (m, 4H), 7.06 – 6.98 (m, 3H), 6.70 (d, *J* = 8.7 Hz, 2H), 5.92 (d, *J* = 15.8 Hz, 1H), 5.79 (dd, *J* = 15.8, 9.3 Hz, 1H), 3.65 (s, 3H), 3.04 (dd, *J* = 13.2, 2.6 Hz, 1H), 2.45 (dd, *J* = 13.2, 10.9 Hz, 1H), 2.32 (ddd, *J* = 10.9, 9.3, 2.6 Hz, 1H), 1.54 – 1.19 (m, 7H), 1.17 (s, 3H), 0.82 (t, *J* = 7.1 Hz, 3H); ^{13}C NMR (101 MHz, Chloroform-*d*) δ 158.76, 141.14, 132.47, 130.16, 129.22, 127.96, 127.54, 127.11, 125.54, 113.77, 74.35, 56.17, 55.15, 40.16, 35.97, 25.41, 24.10, 23.24, 14.13; **HRMS** (APCI) $[M-H]^+$, calculated for $C_{23}H_{29}O_2$; 337.2162; found 337.2193.

(3*S,4*S**,*E*)-3-benzyl-7-chloro-1-(4-methoxyphenyl)-4-methylhept-1-en-4-ol (1v)**

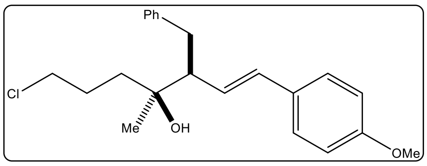

The title compound was prepared according to the general procedure A. The product was obtained as a colourless liquid (78%, 468 mg from 600 mg, *dr* > 95:05). R_f = 0.5 (petroleum ether /Et_2O = 7/3). ^1H NMR (400 MHz, Chloroform-*d*) δ 7.19 – 7.08 (m, 4H), 7.07 – 7.00 (m, 3H), 6.72 (d, *J* = 8.7 Hz, 2H), 5.97 (d, *J* = 15.8 Hz, 1H), 5.78 (dd, *J* = 15.8, 9.5 Hz, 1H), 3.68 (s, 3H), 3.47 (tt, *J* = 6.5, 3.7 Hz, 2H), 3.03 (dd, *J* = 13.2, 2.7 Hz, 1H), 2.47 (dd, *J* = 13.2, 10.8 Hz, 1H), 2.34 (td, *J* = 10.7, 10.2, 2.7 Hz, 1H), 1.94 – 1.77 (m, 2H), 1.67 – 1.50 (m, 3H), 1.20 (s, 3H); ^{13}C NMR (101 MHz, Chloroform-*d*) δ 158.90, 140.79, 133.05, 129.89, 129.18, 128.06, 127.21, 126.91, 125.69, 113.81, 74.02, 56.59, 55.21, 45.68, 37.42, 36.09, 26.69, 23.89; **HRMS** (APCI) $[M-H]^+$, calculated for $C_{22}H_{26}ClO_2$; 357.1616; found 357.1621.

(4*S,5*S**)-4-((*E*)-4-fluorostyryl)-5-methylnon-1-en-5-ol (1w)**

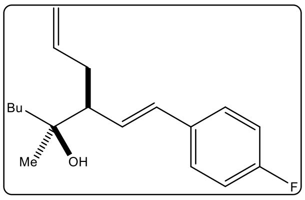

The title compound was prepared according to the general procedure A. The product was obtained as a colourless liquid (78%, 468 mg from 600 mg, *dr* = 94:05). R_f = 0.5 (petroleum ether /Et_2O = 7/3). ^{1}H NMR (400 MHz, Chloroform-*d*) δ 7.39 – 7.29 (m, 2H), 6.99 (t, *J* = 8.7 Hz, 2H), 6.36 (d, *J* = 15.8 Hz, 1H), 5.94 (dd, *J* = 15.9, 9.6 Hz, 1H), 5.76 (ddt, *J* = 17.1, 10.1, 7.0 Hz, 1H), 5.07 – 4.93 (m, 2H), 2.57 – 2.44 (m, 1H), 2.27 (td, *J* = 10.1, 3.1 Hz, 1H), 2.20 – 2.06 (m, 1H), 1.56 – 1.28 (m, 7H), 1.18 (s, 3H), 0.91 (t, *J* = 7.0 Hz, 3H); ^{13}C NMR (101 MHz, Chloroform-*d*) δ 162.08 (d, *J* = 246.2 Hz), 137.66, 133.45 (d, *J* = 3.4 Hz), 131.60, 130.03 (d, *J* = 2.2 Hz), 127.58 (d, *J* = 8.0 Hz), 115.58 (d, *J* = 23.8 Hz), 115.25, 74.37, 53.57, 40.17, 33.86, 25.46, 24.22, 23.27, 14.14; ^{19}F NMR (377 MHz, Chloroform-*d*) δ -115.07; **HRMS** (APCI) [M-OH]$^+$, calculated for $C_{18}H_{24}F$; 259.1857; found 259.1866.

(4*S,5*S**)-5-((*E*)-4-methoxystyryl)-4-methyloct-7-en-4-ol (1x)**

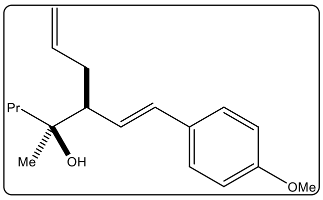

The title compound was prepared according to the general procedure A. The product was obtained as a colourless liquid (78%, 226 mg from 290 mg, *dr* = 95:05). R_f = 0.5 (petroleum ether /Et_2O = 7/3). ^{1}H NMR (400 MHz, Chloroform-*d*) δ 7.33 (d, *J* = 8.7 Hz, 2H), 6.87 (d, *J* = 8.7 Hz, 2H), 6.37 (d, *J* = 15.8 Hz, 1H), 5.90 (dd, *J* = 15.8, 9.5 Hz, 1H), 5.79 (ddt, *J* = 17.0, 10.1, 6.9 Hz, 1H), 5.05 (dq, *J* = 17.1, 1.6 Hz, 1H), 4.98 (ddt, *J* = 10.0, 2.3, 1.1 Hz, 1H), 3.81 (s, 3H), 2.52 (dddt, *J* = 13.2, 7.3, 2.9, 1.2 Hz, 1H), 2.26 (td, *J* = 10.1, 3.0 Hz, 1H), 2.20 – 2.07 (m, 1H), 1.62 (s, 1H), 1.58 – 1.35 (m, 4H), 1.20 (s, 3H), 0.94 (t, *J* = 6.6 Hz, 3H); ^{13}C NMR (101 MHz, Chloroform-*d*) δ 158.85, 137.79, 132.15, 130.06, 127.95, 127.19, 115.43, 113.85, 74.32, 55.19, 53.80, 42.61, 33.91, 23.93, 16.42, 14.63; **HRMS** (APCI) [M+H]$^+$, calculated for $C_{28}H_{27}O_2$; 275.2011; found 275.2000.

(4*S,5*R**)-4-((*E*)-2-(4-methoxyphenyl)ethenyl)-5-methylnonan-5-ol (1ac)**

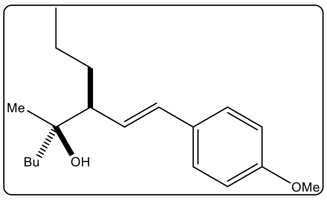

The title compound was prepared according to the general procedure A. The product was obtained as a colourless liquid (77%, 462 mg from 600 mg, *dr* = 95:05). R_f = 0.5 (petroleum ether /Et_2O = 7/3). ^{1}H NMR (400 MHz, $CDCl_3$) δ 7.41 – 7.28 (m, 2H), 6.86 (d, J = 8.7 Hz, 2H), 6.40 (d, J = 15.9 Hz, 1H), 5.90 (dd, J = 15.8, 9.8 Hz, 1H), 3.81 (s, 3H), 2.26 – 2.15 (m, 1H), 1.66 (s, 1H), 1.52 (m, 3H), 1.45 – 1.26 (m, 6H), 1.14 (s, 3H), 0.92 (dt, J = 10.0, 7.0 Hz, 6H); ^{13}C NMR (101 MHz, $CDCl_3$) δ 159.1, 133.0, 130.2, 128.6, 127.4, 114.1, 74.1, 55.4, 53.3, 39.7, 31.9, 25.5, 24.7, 23.5, 21.5, 14.3, 14.2; **HRMS** (APCI) [M-OH]$^+$, calculated for $C_{19}H_{29}O$: 273.2168; found 273.2213.

(4*S,5*S**)-4-((*E*)-2-(3-methoxyphenyl)ethenyl)-5-methylnonan-5-ol (1ad)**

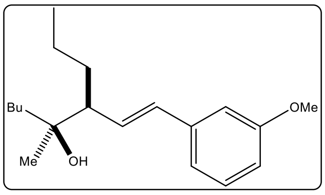

The title compound was prepared according to the general procedure A. The product was obtained as a colourless liquid (77%, 308 mg from 400 mg, *dr* = 95:05). R_f = 0.5 (petroleum ether /Et_2O = 7/3). ^{1}H NMR (400 MHz, $CDCl_3$) δ 7.14 (t, *J* = 7.9 Hz, 1H), 6.89 (d, *J* = 7.7 Hz, 1H), 6.83 (s, 1H), 6.69 (dd, *J* = 8.1, 2.4 Hz, 1H), 6.29 (d, *J* = 15.8 Hz, 1H), 5.92 (dd, *J* = 15.8, 9.8 Hz, 1H), 3.73 (s, 3H), 2.15 – 2.02 (m, 1H), 1.54 (dtd, *J* = 12.5, 6.8, 3.1 Hz, 1H), 1.42 (m, 2H), 1.39 – 1.29 (m, 5H), 1.23 (m, 3H), 1.08 (s, 3H), 0.86 – 0.77 (m, 6H); ^{13}C NMR (101 MHz, $CDCl_3$) δ 159.9, 139.0, 132.6, 131.6, 129.6, 118.9, 112.6, 111.7, 77.5, 76.8, 74.4, 55.3, 54.0, 40.1, 31.2, 25.6, 24.4, 23.4, 21.4, 14.3, 14.2; **HRMS** (APCI) $[M-H]^+$, calculated for $C_{19}H_{29}O_2$: 289.2186; found: 289.2178.

(4*S,5*S**)-4-((*E*)-2-(2-methoxyphenyl)ethenyl)-5-methylnonan-5-ol (1ae)**

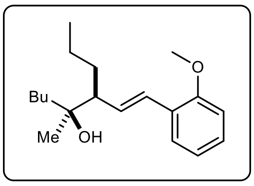

The title compound was prepared according to the general procedure A. The product was obtained as a colourless liquid (55%, 330 mg from 600 mg, *dr* = 95:05). R_f = 0.5 (petroleum ether /Et_2O = 7/3). ^{1}H NMR (400 MHz, $CDCl_3$) δ 7.46 (dd, *J* = 7.6, 1.7 Hz, 1H), 7.21 (td, *J* = 8.3, 1.7 Hz, 1H), 6.93 (t, *J* = 7.5 Hz, 1H), 6.87 (d, *J* = 8.2 Hz, 1H), 6.75 (d, *J* = 16.0 Hz, 1H), 5.99 (dd, *J* = 16.0, 9.9 Hz, 1H), 3.84 (s, 3H), 2.27 – 2.16 (m, 1H), 1.62 (dtd, *J* = 12.5, 6.8, 6.4, 3.1 Hz, 1H), 1.56 – 1.38 (m, 5H), 1.38 – 1.22 (m, 5H), 1.19 (s, 3H), 0.92 (t, *J* = 7.2 Hz, 6H);^{13}C NMR (101 MHz, $CDCl_3$) δ 156.6, 131.7, 128.3, 127.6, 126.5, 120.7, 111.1, 77.5, 76.8, 74.43, 55.60, 54.54, 39.87, 31.43, 25.6, 24.5, 23.5, 21.5, 14.3; **HRMS** (APCI) $[M-H]^+$, calculated for $C_{19}H_{29}O_2$: 289.2186; found: 289.2166.

(4*S,5*S**)-4-((*E*)-2-cyclohexylethenyl)-5-methylnonan-5-ol (1af)**

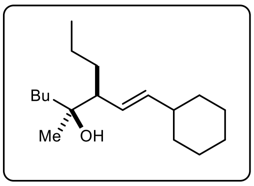

The title compound was prepared according to the general procedure A. The product was obtained as a colourless liquid (47%, 188 mg from 400 mg, *dr* = 95:05). R_f = 0.5 (petroleum ether /Et_2O = 7/3). ^{1}H NMR (400 MHz, $CDCl_3$) δ 5.41 (dd, *J* = 15.4, 6.8 Hz, 1H), 5.10 (dd, *J* = 15.4, 9.7 Hz, 1H), 2.08 – 1.84 (m, 2H), 1.69 (d, *J* = 10.0 Hz, 4H), 1.58 – 1.44 (m, 2H), 1.40 – 1.20 (m, 10H), 1.09 (m, 8H), 0.88 (dt, *J* = 11.1, 6.9 Hz, 6H); ^{13}C NMR (101 MHz, $CDCl_3$) δ 140.6, 127.7, 77.5, 77.2, 76.8, 53.8, 41.1, 39.4, 33.4, 31.3, 26.3, 26.1, 25.5, 24.3, 23.5, 21.3, 14.3, 14.3; **HRMS** (APCI) $[M-H]^+$, calculated for $C_{18}H_{33}O$: 265.2531; found: 265.2542.

(4*S,5*S**)-4-((*E*)-2-(4-fluorophenyl)ethenyl)-5-methylnonan-5-ol (1ag)**

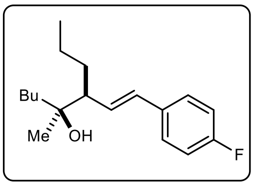

The title compound was prepared according to the general procedure A. The product was obtained as a colourless liquid (85%, 510 mg from 600 mg, *dr* = 95:05). R_f = 0.5 (petroleum ether /Et_2O = 7/3). ^{1}H NMR (400 MHz, $CDCl_3$) δ 7.33 (ddd, *J* = 8.6, 5.3, 2.6 Hz, 2H), 7.04 – 6.91 (m, 2H), 6.36 (d, *J* = 15.8 Hz, 1H), 5.92 (dd, *J* = 15.8, 9.8 Hz, 1H), 2.20 – 2.11 (m, 1H), 1.61 (dtd, *J* = 12.5, 6.8, 3.1 Hz, 1H), 1.50 (m, 2H), 1.47 – 1.36 (m, 3H), 1.36 – 1.21 (m, 5H), 1.17 (s, 3H), 0.90 (td, *J* = 7.0, 1.7 Hz, 6H); ^{13}C NMR (101 MHz, $CDCl_3$) δ 163.4, 161.0, 133.7, 131.6, 131.0, 127.7, 127.6, 115.6, 115.4, 77.5, 76.8, 74.4, 54.1, 40.1, 31.3,

25.7, 24.5, 23.5, 21.4, 14.3, 14.3; ^{19}F NMR (377 MHz, $CDCl_3$) δ -115.19; **HRMS** (APCI) $[M-OH]^+$, calculated for $C_{18}H_{26}F$: 261.2013; found: 261.2020.

Ethyl (2*S,3*S**)-3-hydroxy-2-((*E*)-2-methoxystyryl)-3-methylnonanoate (1ah)**

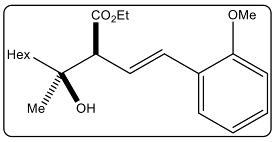

The title compound was prepared according to the general procedure A. The product was obtained as a colourless liquid (74%, 444 mg from 600 mg, *dr* = 95:05). R_f = 0.5 (petroleum ether /Et_2O = 6/4). ^{1}H NMR (400 MHz, Chloroform-*d*) δ 7.48 (dd, *J* = 7.6, 1.7 Hz, 1H), 7.22 (ddd, *J* = 8.2, 7.4, 1.7 Hz, 1H), 6.92 (td, *J* = 7.5, 1.1 Hz, 1H), 6.89 – 6.86 (m, 1H), 6.86 – 6.81 (m, 1H), 6.36 (dd, *J* = 16.0, 9.8 Hz, 1H), 4.28 – 4.12 (m, 2H), 3.84 (s, 3H), 3.35 (s, 1H), 3.25 (d, *J* = 9.7 Hz, 1H), 1.54 – 1.46 (m, 2H), 1.40 (tdd, *J* = 12.0, 6.4, 2.6 Hz, 2H), 1.31 – 1.26 (m, 8H), 1.17 (s, 3H), 0.95 – 0.80 (m, 4H); ^{13}C NMR (101 MHz, Chloroform-*d*) δ 174.22, 156.53, 129.15, 128.77, 126.70, 125.64, 124.45, 120.60, 110.85, 73.83, 60.88, 58.53, 55.44, 41.69, 31.76, 29.76, 23.76, 23.74, 22.56, 14.16, 14.05; **HRMS** (APCI) $[M+H]^+$, calculated for $C_{21}H_{33}O_4$; 349.2379; found 349.2384.

(4*S,5*S**)-4-((*E*)-4-methoxystyryl)-5-methyl-1-(4,4,5,5-tetramethyl-1,3,2-dioxaborolan-2-yl)nonan-5-ol (1ai)**

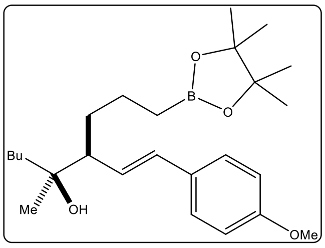

The title compound was prepared according to the general procedure A. The product was obtained as a colourless liquid (70%, 392 mg from 560 mg, *dr* = 95:05). R_f = 0.5 (petroleum ether /Et_2O = 5/5). ^{1}H NMR (400 MHz, Chloroform-*d*) δ 7.30 (d, *J* = 8.7 Hz, 2H), 6.84 (d, *J* = 8.6 Hz, 2H), 6.36 (d, *J* = 15.8 Hz, 1H), 5.85 (dd, *J* = 15.8, 9.7 Hz, 1H), 3.79 (s, 3H), 2.13 (td, *J* = 10.2, 2.4 Hz, 1H), 1.68 – 1.59 (m, 2H), 1.54 – 1.45 (m, 2H), 1.43 – 1.38 (m, 2H), 1.29 (td, *J* = 7.2, 2.9 Hz, 5H), 1.22 (s, 12H), 1.14 (s, 3H), 0.89 (t, *J* = 6.8 Hz, 3H), 0.82 – 0.73 (m, 2H); ^{13}C NMR (101 MHz, Chloroform-*d*) δ 158.78, 132.08, 130.24, 128.76, 127.17, 113.83, 82.81, 74.23, 55.21, 53.97, 39.62, 31.70, 25.47, 24.73, 24.22, 23.30, 22.43, 14.11; ^{11}B NMR (128 MHz, Chloroform-*d*) δ 34.08; **HRMS** (APCI) $[M-OH]^+$, calculated for $C_{25}H_{40}BO_3$; 399.3065; found 399.3096.

Diethyl ((4*S,5*S**)-5-methyl-4-((*E*)-styryl)non-1-en-5-yl) phosphate (5a)**

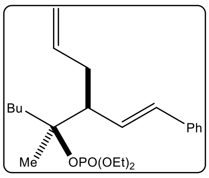

The title compound was prepared according to the general procedure B. The product was obtained as a colourless liquid (90%, 577 mg from 420 mg, *dr* > 95:05). R_f = 0.5 (petroleum ether /Et_2O = 2/8). ^{1}H NMR (400 MHz, Chloroform-*d*) δ 7.41 – 7.33 (m, 2H), 7.30 (dd, *J* = 8.5, 6.7 Hz, 2H), 7.25 – 7.19 (m, 1H), 6.39 (d, *J* = 15.8 Hz, 1H), 5.98 (dd, *J* = 15.8, 9.5 Hz, 1H), 5.73 (ddt, *J* = 17.0, 10.1, 7.0 Hz, 1H), 5.06 – 4.91 (m, 2H), 4.09 (pd, *J* = 7.1, 5.9 Hz, 4H), 2.66 – 2.45 (m, 2H), 2.20 – 2.08 (m, 1H), 1.94 – 1.76 (m, 2H), 1.51 (s, 3H), 1.44 – 1.27 (m, 10H), 0.91 (t, *J* = 7.1 Hz, 3H); ^{13}C NMR (101 MHz, Chloroform-*d*) δ 137.30, 137.15, 133.04, 129.04, 128.51, 127.25, 126.15, 115.89, 88.71 (d, *J* = 8.2 Hz), 63.24 (d, *J* = 5.9 Hz), 51.41 (d, *J* = 7.1 Hz), 38.82, 33.47, 25.59, 22.95, 22.80, 16.17 (d, *J* = 7.2 Hz), 14.08; ^{31}P NMR (162 MHz, Chloroform-*d*) δ -5.68; **HRMS** (APCI) $[M+H]^+$, calculated for $C_{22}H_{36}O_4P$; 395.2351; found 395.2352.

Diethyl ((4*S,5*S**)-4-((*E*)-4-methoxystyryl)-5-methylnon-1-en-5-yl) phosphate (5b)**

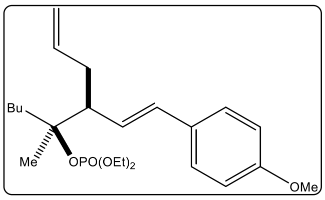

The title compound was prepared according to the general procedure B. The product was obtained as a colourless liquid (91%, 417 mg from 310 mg, *dr* > 95:05). R_f = 0.5 (petroleum ether /Et_2O = 2/8). ^1H NMR (400 MHz, Chloroform-*d*) δ 7.28 (d, *J* = 8.8 Hz, 2H), 6.90 – 6.80 (m, 2H), 6.33 (d, *J* = 15.8 Hz, 1H), 5.83 (dd, *J* = 15.8, 9.5 Hz, 1H), 5.72 (ddd, *J* = 17.1, 10.1, 7.0 Hz, 1H), 5.05 – 4.89 (m, 2H), 4.08 (pd, *J* = 7.1, 5.7 Hz, 4H), 3.80 (s, 3H), 2.59 – 2.44 (m, 2H), 2.18 – 2.10 (m, 1H), 1.93 – 1.75 (m, 2H), 1.50 (s, 3H), 1.41 – 1.27 (m, 10H), 0.90 (t, *J* = 7.1 Hz, 3H); ^{13}C NMR (101 MHz, Chloroform-*d*) δ 158.95, 137.30, 132.37, 130.15, 127.25, 126.81, 115.75, 113.91, 88.85 (d, *J* = 8.2 Hz), 63.21 (d, *J* = 6.0 Hz), 55.30, 51.36 (d, *J* = 7.3 Hz), 38.80, 33.53, 25.56, 22.94, 22.73 (d, *J* = 2.5 Hz), 16.17 (d, *J* = 7.1 Hz), 14.08; ^{31}P NMR (162 MHz, Chloroform-*d*) δ -5.69; **HRMS** (APCI) $[M+H]^+$, calculated for $C_{23}H_{38}O_5P$; 425.2457; found 425.2441.

Diethyl ((4*S,5*S**)-4-((*E*)-3-methoxystyryl)-5-methylnon-1-en-5-yl) phosphate (5c)**

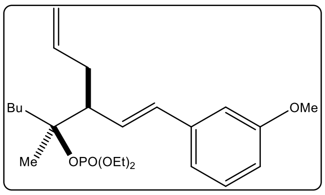

The title compound was prepared according to the general procedure B. The product was obtained as a colourless liquid (89%, 263 mg from 200 mg, *dr* > 95:05). R_f = 0.5 (petroleum ether /Et_2O = 2/8). ^1H NMR (400 MHz, Chloroform-*d*) δ 7.21 (t, *J* = 7.9 Hz, 1H), 6.95 (dt, *J* = 7.8, 1.2 Hz, 1H), 6.88 (t, *J* = 2.0 Hz, 1H), 6.77 (ddd, *J* = 8.2, 2.6, 0.9 Hz, 1H), 6.36 (d, *J* = 15.8 Hz, 1H), 5.97 (dd, *J* = 15.8, 9.5 Hz, 1H), 5.71 (ddt, *J* = 17.0, 10.1, 6.9 Hz, 1H), 5.08 – 4.89 (m, 2H), 4.08 (pd, *J* = 7.1, 5.8 Hz, 4H), 3.80 (s, 3H), 2.64 – 2.45 (m, 2H), 2.17 – 2.07 (m, 1H), 1.96 – 1.76 (m, 2H), 1.50 (s, 3H), 1.40 – 1.26 (m, 10H), 0.90 (t, *J* = 7.1 Hz, 3H); ^{13}C NMR (101 MHz, Chloroform-*d*) δ 159.72, 138.69, 137.03, 132.87, 129.41, 129.29, 118.71, 115.86, 112.58, 111.66, 88.60 (d, *J* = 8.1 Hz), 63.17 (d, *J* = 6.0 Hz), 55.12, 51.32 (d, *J* = 7.2 Hz), 38.72 (d, *J* = 1.9 Hz), 33.39, 25.51, 22.87, 22.71 (d, *J* = 2.5 Hz), 16.11 (d, *J* = 7.1 Hz), 14.02; ^{31}P NMR (162 MHz, Chloroform-*d*) δ -5.69; **HRMS** (APCI) $[M+H]^+$, calculated for $C_{23}H_{38}O_5P$; 425.2457; found 425.2468.

Diethyl ((4*S,5*S**)-5-ethyl-4-((*E*)-styryl)non-1-en-5-yl) phosphate (5d)**

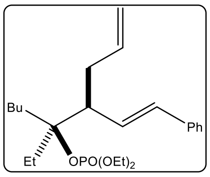

The title compound was prepared according to the general procedure B. The product was obtained as a colourless liquid (74%, 343 mg from 310 mg, *dr* = 95:05). R_f = 0.5 (petroleum ether /Et_2O = 2/8). ^1H NMR (500 MHz, Chloroform-*d*) δ 7.36 (d, *J* = 7.2 Hz, 2H), 7.30 (t, *J* = 7.6 Hz, 2H), 7.21 (t, *J* = 7.3 Hz, 1H), 6.34 (d, *J* = 15.8 Hz, 1H), 6.16 (dd, *J* = 15.8, 9.6 Hz, 1H), 5.73 (ddt, *J* = 17.2, 10.1, 6.9 Hz, 1H), 5.07 – 4.89 (m, 2H), 4.08 (p, *J* = 7.2 Hz, 4H), 2.60 – 2.41 (m, 2H), 2.22 (tdd, *J* = 10.5, 8.1, 4.2 Hz, 1H), 2.05 – 1.80 (m, 4H), 1.44 – 1.28 (m, 10H), 0.99 – 0.86 (m, 6H); ^{13}C NMR (126 MHz, Chloroform-*d*) δ 137.38, 137.29, 132.53, 129.42, 128.45, 127.09, 126.11, 115.74, 92.33 (d, *J* = 9.2 Hz), 63.22 (dd, *J* = 6.1, 2.0 Hz), 51.09 (d, *J* = 4.6 Hz), 34.88 (d, *J* = 2.7 Hz), 33.31, 28.92 (d, *J* = 3.4 Hz), 25.84, 23.09, 16.13 (d, *J* = 7.1 Hz), 14.02, 8.34; ^{31}P NMR (202 MHz, Chloroform-*d*) δ -6.32; **HRMS** (APCI) $[M+H]^+$, calculated for $C_{23}H_{38}O_4P$; 409.2508; found 409.2524.

Diethyl ((4*S**,5*S**)-4-((*E*)-4-fluorostyryl)-5-methylnon-1-en-5-yl) phosphate (5e)

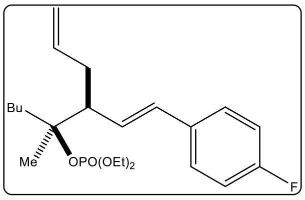

The title compound was prepared according to the general procedure B. The product was obtained as a colourless liquid (85%, 415 mg from 340 mg, *dr* = 95:05). R_f = 0.5 (petroleum ether /Et_2O = 2/8). ^{1}H NMR (400 MHz, Chloroform-*d*) δ 7.30 (dd, *J* = 8.6, 5.5 Hz, 2H), 6.97 (t, *J* = 8.7 Hz, 2H), 6.34 (d, *J* = 15.8 Hz, 1H), 5.89 (dd, *J* = 15.8, 9.4 Hz, 1H), 5.70 (ddt, *J* = 17.1, 10.1, 7.0 Hz, 1H), 5.04 – 4.88 (m, 2H), 4.07 (h, *J* = 7.2 Hz, 4H), 2.61 – 2.42 (m, 2H), 2.17 – 2.08 (m, 1H), 1.93 – 1.75 (m, 2H), 1.49 (s, 3H), 1.40 – 1.26 (m, 10H), 0.89 (t, *J* = 7.1 Hz, 3H); ^{13}C NMR (101 MHz, Chloroform-*d*) δ 162.04 (d, *J* = 246.3 Hz), 137.02, 133.38 (d, *J* = 3.3 Hz), 131.78, 128.75 (d, *J* = 2.2 Hz), 127.53 (d, *J* = 7.8 Hz), 115.88, 115.29 (d, *J* = 21.5 Hz), 88.59 (d, *J* = 8.1 Hz), 63.20 (d, *J* = 6.1 Hz), 51.34 (d, *J* = 7.0 Hz), 38.70 (d, *J* = 2.1 Hz), 33.40, 25.53, 22.88, 22.76 (d, *J* = 2.5 Hz), 16.10 (d, *J* = 7.0 Hz), 14.00; ^{31}P NMR (162 MHz, Chloroform-*d*) δ -5.69; ^{19}F NMR (377 MHz, Chloroform-*d*) δ -115.03; **HRMS** (APCI) $[M+H]^+$, calculated for $C_{22}H_{35}FO_4P$; 413.2257; found 413.2275.

Diethyl ((4*R**,5*S**)-4-(methoxymethyl)-2,5-dimethylnon-2-en-5-yl) phosphate (5f)

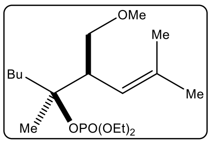

The title compound was prepared according to the general procedure B. The product was obtained as a colourless liquid (86%, 392 mg from 280 mg, *dr* = 95:05). R_f = 0.5 (petroleum ether /Et_2O = 2/8). ^{1}H NMR (400 MHz, Chloroform-*d*) δ 5.00 (dt, *J* = 10.3, 1.5 Hz, 1H), 4.02 (pd, *J* = 7.2, 2.6 Hz, 4H), 3.58 (dd, *J* = 9.4, 4.3 Hz, 1H), 3.29 (dd, *J* = 9.4, 7.6 Hz, 1H), 3.24 (s, 3H), 2.80 (tdd, *J* = 10.1, 4.3, 2.3 Hz, 1H), 1.86 – 1.71 (m, 2H), 1.70 (d, *J* = 1.4 Hz, 3H), 1.61 (d, *J* = 1.4 Hz, 3H), 1.38 (s, 3H), 1.27 (tt, *J* = 7.2, 1.3 Hz, 10H), 0.84 (t, *J* = 7.0 Hz, 3H); ^{13}C NMR (101 MHz, Chloroform-*d*) δ 134.69, 122.43, 89.05 (d, *J* = 8.3 Hz), 73.15, 63.03 (d, *J* = 5.9 Hz), 58.68, 45.69 (d, *J* = 7.3 Hz), 38.95 (d, *J* = 1.6 Hz), 26.06, 25.65, 22.84, 22.70 (d, *J* = 2.5 Hz), 18.41, 16.03 (d, *J* = 7.1 Hz), 13.90; ^{31}P NMR (162 MHz, Chloroform-*d*) δ -5.94; **HRMS** (APCI) $[M+H]^+$, calculated for $C_{17}H_{36}O_5P$; 351.2300; found 351.2285.

Diethyl ((4*R**,5*S**,*E*)-4-(methoxymethyl)-5-methyl-2-phenylnon-2-en-5-yl) phosphate (5g)

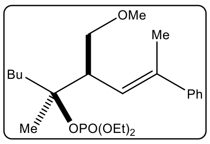

The title compound was prepared according to the general procedure B. The product was obtained as a colourless liquid (88%, 359 mg from 290 mg, *dr* = 95:05). R_f = 0.5 (petroleum ether /Et_2O = 2/8). ^{1}H NMR (400 MHz, Chloroform-*d*) δ 7.45 – 7.35 (m, 2H), 7.29 (dd, *J* = 8.4, 6.8 Hz, 2H), 7.24 – 7.17 (m, 1H), 5.70 (dt, *J* = 10.4, 1.4 Hz, 1H), 4.07 (h, *J* = 7.1 Hz, 4H), 3.73 (dd, *J* = 9.4, 4.4 Hz, 1H), 3.42 (dd, *J* = 9.5, 7.4 Hz, 1H), 3.30 (s, 3H), 3.06 (dddd, *J* = 10.0, 7.2, 4.4, 2.4 Hz, 1H), 2.09 (d, *J* = 1.4 Hz, 3H), 1.94 – 1.77 (m, 2H), 1.51 (s, 3H), 1.42 – 1.21 (m, 10H), 0.90 (t, *J* = 7.1 Hz, 3H); ^{13}C NMR (101 MHz, Chloroform-*d*) δ 143.69, 137.52, 128.02, 126.71, 126.07, 125.70, 88.74 (d, *J* = 8.1 Hz), 72.97, 63.10 (d, *J* = 6.1 Hz), 58.77, 46.48 (d, *J* = 7.1 Hz), 39.11 (d, *J* = 1.7 Hz), 25.77, 22.96 (d, *J* = 2.5 Hz), 22.85, 16.52, 16.02 (dd, *J* = 7.3, 2.2 Hz), 13.92; ^{31}P NMR (162 MHz, Chloroform-*d*) δ -5.75; **HRMS** (APCI) $[M+H]^+$, calculated for $C_{22}H_{38}O_5P$; 413.2457; found 413.2454.

Diethyl ((4*S**,5*S**)-5-methyl-4-((*E*)-styryl)-1-(4,4,5,5-tetramethyl-1,3,2-dioxaborolan-2-yl)nonan-5-yl) phosphate (5h)

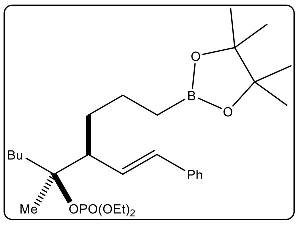

The title compound was prepared according to the literature procedure[3,4] from compound **3a**. The product was obtained as a colourless liquid (70%, 185 mg from 200 mg, *dr* > 95:05). R_f = 0.5 (petroleum ether /Et_2O = 2/8). ^{1}H NMR (400 MHz, Chloroform-*d*) δ 7.40 – 7.32 (m, 2H), 7.29 (dd, *J* = 8.4, 6.8 Hz, 2H), 7.23 – 7.17 (m, 1H), 6.40 (d, *J* = 15.8 Hz, 1H), 5.98 (dd, *J* = 15.9, 9.7 Hz, 1H), 4.05 (pd, *J* = 7.1, 6.0 Hz, 4H), 2.47 – 2.35 (m, 1H), 1.90 – 1.75 (m, 2H), 1.73 – 1.64 (m, 1H), 1.48 (s, 4H), 1.30 (dtd, *J* = 11.7, 7.1, 1.0 Hz, 12H), 1.21 (s, 12H), 0.89 (t, *J* = 7.0 Hz, 3H), 0.76 (td, *J* = 9.0, 6.6 Hz, 2H); ^{13}C NMR (101 MHz, Chloroform-*d*) δ 137.41, 132.54, 129.89, 128.42, 127.03, 126.04, 88.96 (d, *J* = 8.4 Hz), 82.82, 63.10 (d, *J* = 6.1 Hz), 51.20 (d, *J* = 7.3 Hz), 38.59 (d, *J* = 2.2 Hz), 31.20, 25.62, 24.73, 23.07 (d, *J* = 2.2 Hz), 22.95, 22.06, 16.11 (d, *J* = 7.1 Hz), 14.07; ^{31}P NMR (162 MHz, Chloroform-*d*) δ -5.70; ^{11}B NMR (128 MHz, Chloroform-*d*) δ 34.33; **HRMS** (APCI) $[M+H]^+$, calculated for $C_{28}H_{49}BO_6P$; 523.3354; found 523.3339.

Determination of relative stereochemistry through chemical correlation

Relative stereochemistry of each class of products was determined by analogy to our previous publications:

1. **Fluorination**: (i) Compound **4l** in this manuscript was compared with **9t** in our previous publication[1b]; (ii) Compound **4a** in this manuscript was compared with **2m** in our previous publication, for which the crystal structure was also reported (CCDC 1888232).[1c]
2. **Bromination**: Compound **4ac** in this manuscript was compared with **2a** in our previous publication.[1c]
3. **Chlorination**: Compound **4ad** in this manuscript was compared with **2j** in our previous publication.[1c]
4. **Azidation**: Compound **4ae** in this manuscript was compared with **6g** in our previous publication. [1d]
5. **Thiocyanation**: Compound **4ap** in this manuscript was compared with **2p** in our previous publication.[1e]

Characterization data for products

Ethyl (2*R,3*S**)-3-fluoro-3-methyl-2-((*E*)-styryl)heptanoate (4a)**

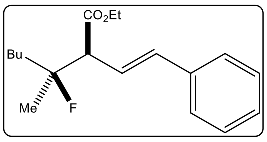

The title compound was prepared according to the general procedure C. The product was obtained as a colourless liquid (81%, 90 mg from 110 mg, *dr* = 95:05). R_f = 0.5 (petroleum ether /Et_2O = 8/2). ^{1}H NMR (400 MHz, Chloroform-*d*) δ 7.34 – 7.30 (m, 2H), 7.27 – 7.22 (m, 2H), 7.21 – 7.15 (m, 1H), 6.44 (d, *J* = 15.9 Hz, 1H), 6.21 (dd, *J* = 15.9, 9.8 Hz, 1H), 4.12 (qd, *J* = 7.1, 2.8 Hz, 2H), 3.40 (dd, *J* = 12.0, 9.8 Hz, 1H), 1.70 – 1.55 (m, 2H), 1.40 (s, 1H), 1.39 – 1.29 (m, 4H), 1.28 – 1.23 (m, 2H), 1.20 (d, *J* = 7.1 Hz, 3H), 0.84 (t, *J* = 7.3 Hz, 3H); ^{13}C NMR (101 MHz, Chloroform-*d*) δ 171.03 (d, *J* = 3.6 Hz), 136.43, 134.57, 128.58, 127.89, 126.47, 123.42 (d, *J* = 7.3 Hz), 97.31 (d, *J* = 177.6 Hz), 60.85, 58.18 (d, *J* = 23.8 Hz), 38.21 (d, *J* = 22.4 Hz), 25.18 (d, *J* = 4.0 Hz), 22.89, 21.62 (d, *J* = 24.6 Hz), 14.16, 13.99; ^{19}F NMR (377 MHz, Chloroform-*d*) δ -147.66; **HRMS** (ESI) $[M+Na]^+$, calculated for $C_{18}H_{25}FNaO_2$; 315.1731; found : 315.1748.

Ethyl (2*R,3*S**)-3-fluoro-2-((*E*)-4-methoxystyryl)-3-methylheptanoate (4b)**

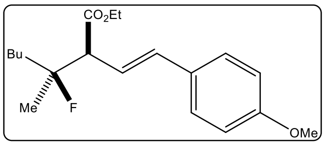

The title compound was prepared according to the general procedure C. The product was obtained as a colourless liquid (92%, 111 mg from 120 mg, *dr* > 95:05). R_f = 0.5 (petroleum ether /Et_2O = 8/2). ^{1}H NMR (400 MHz, Chloroform-*d*) δ 7.33 (d, *J* = 8.7 Hz, 2H), 6.86 (d, *J* = 8.8 Hz, 2H), 6.45 (d, *J* = 15.8 Hz, 1H), 6.13 (dd, *J* = 15.9, 9.8 Hz, 1H), 4.19 (qq, *J* = 6.9, 3.7 Hz, 2H), 3.81 (s, 3H), 3.45 (dd, *J* = 11.8, 9.8 Hz, 1H), 1.74 – 1.64 (m, 2H), 1.47 – 1.40 (m, 4H), 1.33 – 1.25 (m, 6H), 0.91 (t, *J* = 7.2 Hz, 3H); ^{13}C NMR (101 MHz, Chloroform-*d*) δ 171.24 (d, *J* = 3.6 Hz), 159.41, 133.97, 129.24, 127.67, 121.09 (d, *J* = 7.4 Hz), 113.96, 97.40 (d, *J* = 177.2 Hz), 60.79, 58.18 (d, *J* = 23.8 Hz), 55.28, 38.22 (d, *J* = 22.4 Hz), 25.17 (d, *J* = 4.2 Hz), 22.89, 21.54 (d, *J* = 24.6 Hz), 14.16, 14.00; ^{19}F NMR (377 MHz, Chloroform-*d*) δ -147.72; **HRMS** (APCI) $[M+H]^+$, calculated for $C_{19}H_{28}FO_3$; 323.2022; found 323.1993.

ethyl (2*R,3*R**)-3-fluoro-2-((*E*)-4-methoxystyryl)-3-methylheptanoate (4c)**

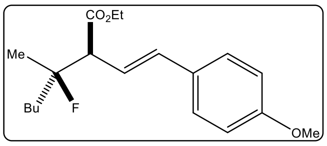

The title compound was prepared according to the general procedure C. The product was obtained as a colourless liquid (91%, 73 mg from 80 mg, *dr* = 93:07). R_f = 0.5 (petroleum ether /Et_2O = 8/2). ^{1}H NMR (400 MHz, Chloroform-*d*) δ 7.34 (d, *J* = 8.7 Hz, 2H), 6.86 (d, *J* = 8.7 Hz, 2H), 6.45 (d, *J* = 15.9 Hz, 1H), 6.20 (dd, *J* = 15.9, 9.4 Hz, 1H), 4.19 (qd, *J* = 7.1, 2.9 Hz, 2H), 3.80 (s, 3H), 3.43 (dd, *J* = 15.4, 9.4 Hz, 1H), 1.89 – 1.75 (m, 1H), 1.68 – 1.57 (m, 1H), 1.49 – 1.31 (m, 7H), 1.28 (t, *J* = 7.1 Hz, 3H), 0.91 (t, *J* = 7.2 Hz, 3H); ^{13}C NMR (101 MHz, Chloroform-*d*) δ 171.19 (d, *J* = 7.2 Hz), 159.35, 133.88, 129.40, 127.65, 120.97 (d, *J* = 4.9 Hz), 113.93, 96.99 (d, *J* = 177.4 Hz), 60.76, 58.27 (d, *J* = 23.9 Hz), 55.25, 37.53 (d, *J* = 22.5 Hz), 25.22 (d, *J* = 4.8 Hz), 22.91, 22.25 (d, *J* = 24.2 Hz), 14.05 (d, *J* = 17.8 Hz); ^{19}F NMR (377 MHz, Chloroform-*d*) δ -147.89; **HRMS** (APCI) $[M+H]^+$, calculated for $C_{19}H_{28}FO_3$; 323.2022; found 323.1993.

Ethyl (2*R,3*S**)-2-((*E*)-4-bromostyryl)-3-fluoro-3-methylheptanoate (4d)**

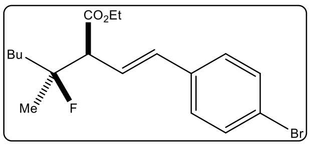

The title compound was prepared according to the general procedure C. The product was obtained as a colourless liquid (60%, 45 mg from 75 mg, *dr* = 91:09). R_f = 0.5 (petroleum ether /Et_2O = 8/2). ^{1}H NMR (400 MHz, Chloroform-*d*) δ 7.37 (d, *J* = 8.5 Hz, 2H), 7.18 (d, *J* = 8.1 Hz, 2H), 6.38 (d, *J* = 15.9 Hz, 1H), 6.21 (dd, *J* = 15.9, 9.7 Hz, 1H), 4.12 (qq, *J* = 7.3, 3.7 Hz, 2H), 3.38 (dd, *J* = 12.5, 9.7 Hz, 1H), 1.62 (dddd, *J* = 22.2, 11.5, 8.5, 3.0 Hz, 2H), 1.38 (s, 2H), 1.35 (d, *J* = 2.8 Hz, 1H), 1.33 (s, 2H), 1.27 (d, *J* = 7.3 Hz, 2H), 1.20 (d, *J* = 7.1 Hz, 3H), 0.84 (t, *J* = 7.2 Hz, 3H); ^{13}C NMR (101 MHz, Chloroform-*d*) δ 170.83 (d, *J* = 3.9 Hz), 135.34, 133.38, 131.67, 127.99, 124.26 (d, *J* = 7.0 Hz), 121.69, 97.17 (d, *J* = 177.7 Hz), 60.93, 58.17 (d, *J* = 23.8 Hz), 38.11 (d, *J* = 22.3 Hz), 25.19 (d, *J* = 4.2 Hz), 22.88, 21.73 (d, *J* = 24.6 Hz), 14.15, 13.98; ^{19}F NMR (377 MHz, Chloroform-*d*) δ -147.68. **HRMS** (APCI) $[M-H]^+$, calculated for $C_{18}H_{23}BrFO_2$; 369.0865; found 369.0876.

Ethyl (2*R,3*S**)-3-fluoro-2-((*E*)-4-methoxystyryl)-3-methylnonanoate (4e)**

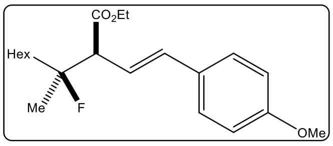

The title compound was prepared according to the general procedure C. The product was obtained as a colourless liquid (91%, 55 mg from 60 mg, *dr* > 95:05). R_f = 0.5 (petroleum ether /Et_2O = 8/2). ^1H NMR (400 MHz, Chloroform-*d*) δ 7.33 (d, *J* = 8.8 Hz, 1H), 6.86 (d, *J* = 8.8 Hz, 1H), 6.45 (d, *J* = 15.8 Hz, 1H), 6.13 (dd, *J* = 15.8, 9.8 Hz, 1H), 4.19 (qq, *J* = 6.9, 3.7 Hz, 1H), 3.81 (s, 2H), 3.45 (dd, *J* = 11.8, 9.8 Hz, 1H), 1.89 – 1.57 (m, 1H), 1.56 – 1.36 (m, 3H), 1.29 (h, *J* = 3.3 Hz, 7H), 0.88 (t, *J* = 6.5 Hz, 2H); ^{13}C NMR (101 MHz, Chloroform-*d*) δ 171.21 (d, *J* = 3.6 Hz), 159.40, 133.95, 129.23, 127.66, 121.08 (d, *J* = 7.5 Hz), 113.95, 97.39 (d, *J* = 177.1 Hz), 60.76, 58.15 (d, *J* = 23.8 Hz), 55.25, 38.51 (d, *J* = 22.3 Hz), 31.68, 29.45, 22.94 (d, *J* = 4.1 Hz), 22.51, 21.53 (d, *J* = 24.6 Hz), 14.14, 14.00; ^{19}F NMR (377 MHz, Chloroform-*d*) δ -147.66; **HRMS** (APCI) $[M+H]^+$, calculated for $C_{21}H_{32}FO_3$; 351.2305; found 351.2305.

1-((3*R,4*S**,*E*)-4-fluoro-3-(methoxymethyl)-4-methyloct-1-en-1-yl)-4-methoxybenzene (4f)**

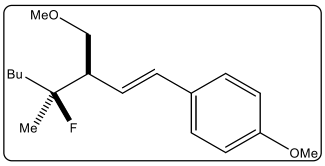

The title compound was prepared according to the general procedure C. The product was obtained as a colourless liquid (85%, 68 mg from 80 mg, *dr* = 90:10). R_f = 0.5 (petroleum ether /Et_2O = 8/2). ^1H NMR (400 MHz, Chloroform-*d*) δ 7.32 (d, *J* = 9.1 Hz, 2H), 6.85 (d, *J* = 8.9 Hz, 2H), 6.45 (d, *J* = 15.8 Hz, 1H), 5.94 (dd, *J* = 15.7, 9.2 Hz, 1H), 3.80 (s, 3H), 3.69 (dd, *J* = 9.4, 4.1 Hz, 1H), 3.53 (t, *J* = 8.6 Hz, 1H), 3.34 (s, 3H), 2.71 (dtd, *J* = 13.1, 8.6, 3.9 Hz, 1H), 1.76 – 1.59 (m, 2H), 1.43 – 1.26 (m, 7H), 0.91 (t, *J* = 7.1 Hz, 3H); ^{13}C NMR (101 MHz, Chloroform-*d*) δ 159.04, 132.65, 130.09, 127.42, 125.41 (d, *J* = 7.5 Hz), 113.89, 98.10 (d, *J* = 173.2 Hz), 72.30 (d, *J* = 5.2 Hz), 58.91, 55.29, 51.33 (d, *J* = 21.8 Hz), 38.76 (d, *J* = 22.6 Hz), 25.14 (d, *J* = 4.7 Hz), 22.99, 22.06 (d, *J* = 25.0 Hz), 14.03; ^{19}F NMR (377 MHz, Chloroform-*d*) δ -149.06. **HRMS** (APCI) $[M-F]^+$, calculated for $C_{18}H_{27}O_2$; 275.2006; found 275.2016.

((3*R,4*S**,*E*)-4-fluoro-3-(methoxymethyl)-4-methyloct-1-en-1-yl)benzene (4g)**

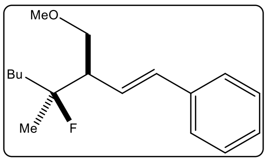

The title compound was prepared according to the general procedure C. The product was obtained as a colourless liquid (78%, 55 mg from 70 mg, *dr* = 88:12). R_f = 0.5 (petroleum ether /Et_2O = 8/2). ^1H NMR (400 MHz, Chloroform-*d*) δ 7.39 (d, *J* = 7.1 Hz, 2H), 7.31 (t, *J* = 7.6 Hz, 2H), 7.22 (t, *J* = 7.2 Hz, 1H), 6.51 (d, *J* = 15.9 Hz, 1H), 6.10 (dd, *J* = 15.9, 9.5 Hz, 1H), 3.71 (dd, *J* = 9.3, 4.1 Hz, 1H), 3.54 (t, *J* = 8.5 Hz, 1H), 3.35 (s, 3H), 2.75 (dtd, *J* = 13.3, 8.6, 4.0 Hz, 1H), 1.73 – 1.60 (m, 2H), 1.44 – 1.23 (m, 7H), 0.91 (t, *J* = 7.2 Hz, 3H); ^{13}C NMR (101 MHz, Chloroform-*d*) δ 137.20, 133.29, 128.46, 127.63 (d, *J* = 7.3 Hz), 127.33, 126.28, 98.00 (d, *J* = 173.3 Hz), 72.17 (d, *J* = 5.3 Hz), 58.92, 51.35 (d, *J* = 21.9 Hz), 38.75 (d, *J* = 22.6 Hz), 25.14 (d, *J* = 4.7 Hz), 22.98, 22.11 (d, *J* = 25.0 Hz), 14.03; ^{19}F NMR (377 MHz, Chloroform-*d*) δ -149.09; **HRMS** (APCI) $[M-F]^+$, calculated for $C_{17}H_{25}O$; 245.1900; found 245.1904.

((3*S,4*S**,*E*)-3-allyl-4-fluoro-4-methyloct-1-en-1-yl)benzene (4h)**

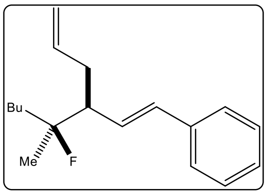

The title compound was prepared according to the general procedure C. The product was obtained as a colourless liquid (87%, 140 mg from 160 mg, *dr* = 88:12). R_f = 0.5 (petroleum ether /Et_2O = 9/1). ^1H NMR (400 MHz, Chloroform-*d*) δ 7.31 – 7.26 (m, 2H), 7.23 (dd, *J* = 8.5, 6.7 Hz, 2H), 7.18 – 7.11 (m, 1H), 6.32 (d, *J* = 15.8 Hz, 1H), 5.86 (dd, *J* = 15.8, 9.5 Hz, 1H), 5.67 (ddt, *J* = 17.0, 10.1, 6.9 Hz, 1H), 5.00 – 4.84 (m, 2H), 2.51 – 2.31 (m, 2H), 2.14 – 2.01 (m, 1H), 1.65 – 1.49 (m, 2H), 1.38 – 1.19 (m, 7H), 0.83 (t, *J* = 7.2 Hz, 3H); ^{13}C NMR (101 MHz, Chloroform-*d*) δ 137.24 & 137.12, 132.86, 129.10 (d, *J* = 7.9 Hz), 128.51, 127.28, 126.17, 115.88, 98.44 (d, *J* = 173.8 Hz), 51.56 (d, *J* = 22.5 Hz), 38.73 (d, *J* = 22.8 Hz), 33.28 (d, *J* = 4.2 Hz), 25.18 (d, *J* = 4.7 Hz), 23.04, 21.29 (d, *J* = 25.0 Hz), 14.06; ^{19}F NMR (377 MHz, Chloroform-*d*) δ -149.69; **HRMS** (APCI) $[M]^+$, calculated for $C_{18}H_{25}F$; 260.1946; found 260.1942.

1-((3*S,4*S**,*E*)-3-allyl-4-fluoro-4-methyloct-1-en-1-yl)-2-methoxybenzene (4i)**

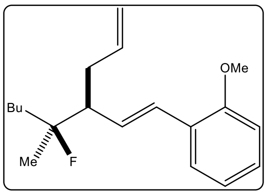

The title compound was prepared according to the general procedure C. The product was obtained as a colourless liquid (90%, 81 mg from 90 mg, *dr* = 90:10). R_f = 0.5 (petroleum ether /Et_2O = 9/1). ^1H NMR (400 MHz, Chloroform-*d*) δ 7.43 (dd, *J* = 7.6, 1.7 Hz, 1H), 7.22 (td, *J* = 7.8, 1.8 Hz, 1H), 6.93 (t, *J* = 7.3 Hz, 1H), 6.88 (dd, *J* = 8.3, 1.1 Hz, 1H), 6.74 (d, *J* = 16.0 Hz, 1H), 5.93 (dd, *J* = 16.0, 9.5 Hz, 1H), 5.79 (ddt, *J* = 17.0, 10.1, 6.9 Hz, 1H), 5.05 (dt, *J* = 17.0, 1.9 Hz, 1H), 4.98 (dd, *J* = 10.2, 2.0 Hz, 1H), 3.85 (s, 3H), 2.63 – 2.44 (m, 2H), 2.25 – 2.09 (m, 1H), 1.74 – 1.58 (m, 2H), 1.48 – 1.28 (m, 7H), 0.93 (t, *J* = 7.2 Hz, 3H); ^{13}C NMR (101 MHz, Chloroform-*d*) δ 156.49, 137.34, 129.55 (d, *J* = 7.8 Hz), 128.27, 127.51, 126.42, 120.55, 115.66, 110.95, 98.61 (d, *J* = 173.7 Hz), 55.48, 51.70 (d, *J* = 22.4 Hz), 38.80 (d, *J* = 22.7 Hz), 33.35 (d, *J* = 4.1 Hz), 25.15 (d, *J* = 4.5 Hz), 23.04, 21.27 (d, *J* = 25.0 Hz), 14.04; ^{19}F NMR (377 MHz, Chloroform-*d*) δ -149.72; **HRMS** (APCI) $[M-F]^+$, calculated for $C_{19}H_{27}O$; 271.2062; found 271.2033.

1-((3*S,4*S**,*E*)-3-allyl-4-fluoro-4-methyloct-1-en-1-yl)-3-methoxybenzene (4j)**

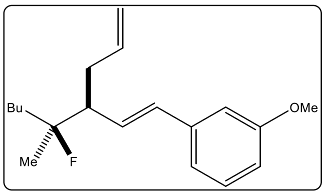

The title compound was prepared according to the general procedure C. The product was obtained as a colourless liquid (90%, 54 mg from 60 mg, *dr* = 90:10). R_f = 0.5 (petroleum ether /Et_2O = 9/1). ^1H NMR (400 MHz, Chloroform-*d*) δ 7.27 – 7.22 (m, 1H), 6.98 (dt, *J* = 7.7, 1.3 Hz, 1H), 6.91 (t, *J* = 2.1 Hz, 1H), 6.80 (ddd, *J* = 8.2, 2.6, 1.0 Hz, 1H), 6.38 (d, *J* = 15.8 Hz, 1H), 5.95 (dd, *J* = 15.8, 9.5 Hz, 1H), 5.76 (ddt, *J* = 17.0, 10.0, 6.9 Hz, 1H), 5.12 – 4.93 (m, 2H), 3.83 (s, 3H), 2.66 – 2.40 (m, 2H), 2.23 – 2.09 (m, 1H), 1.66 (dddd, *J* = 24.9, 14.0, 9.4, 5.4 Hz, 2H), 1.46 – 1.27 (m, 7H), 0.93 (t, *J* = 7.1 Hz, 3H); ^{13}C NMR (101 MHz, $CDCl_3$) δ 159.78, 138.68, 137.06, 132.74, 129.48, 129.47, 129.39, 118.81, 115.90, 112.73, 111.66, 99.26, 97.54, 77.32, 77.00, 76.68, 55.19, 51.64, 51.42, 38.82, 38.59, 33.27, 33.23, 25.20, 25.15, 23.02, 21.42, 21.17, 14.05; ^{19}F NMR (377 MHz, Chloroform-*d*) δ -149.71; **HRMS** (APCI) $[M-F]^+$, calculated for $C_{19}H_{27}O$; 271.2062; found 271.2055.

1-((3*S,4*S**,*E*)-3-allyl-4-fluoro-4-methyloct-1-en-1-yl)-4-methoxybenzene (4k)**

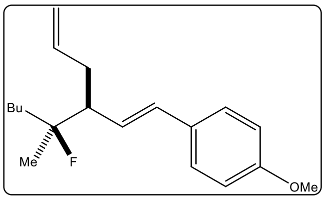

The title compound was prepared according to the general procedure C. The product was obtained as a colourless liquid (90%, 63 mg from 70 mg, *dr* = 92:08). R_f = 0.5 (petroleum ether /Et_2O = 9/1). ^{1}H NMR (400 MHz, Chloroform-*d*) δ 7.31 (d, *J* = 8.7 Hz, 2H), 6.89 – 6.82 (m, 2H), 6.34 (d, *J* = 15.8 Hz, 1H), 5.92 – 5.69 (m, 2H), 5.11 – 4.87 (m, 2H), 3.81 (s, 3H), 2.64 – 2.31 (m, 2H), 2.22 – 2.08 (m, 1H), 1.65 (dddd, *J* = 23.3, 11.7, 6.5, 4.6 Hz, 2H), 1.44 – 1.27 (m, 7H), 0.92 (t, *J* = 7.2 Hz, 3H); ^{13}C NMR (101 MHz, Chloroform-*d*) δ 158.99, 137.27, 132.19, 130.09, 127.28, 126.91 (d, *J* = 7.8 Hz), 115.74, 113.92, 98.55 (d, *J* = 173.6 Hz), 55.29, 51.54 (d, *J* = 22.4 Hz), 38.73 (d, *J* = 22.7 Hz), 33.34 (d, *J* = 4.2 Hz), 25.17 (d, *J* = 4.7 Hz), 23.03, 21.23 (d, *J* = 25.0 Hz), 14.05; ^{19}F NMR (377 MHz, Chloroform-*d*) δ -149.64; **HRMS** (APCI) $[M-F]^+$, calculated for $C_{19}H_{27}O$; 271.2062; found 271.2072.

1-((3*S,4*S**,*E*)-4-fluoro-4-methyl-3-propyloct-1-en-1-yl)-4-methoxybenzene (4l)**

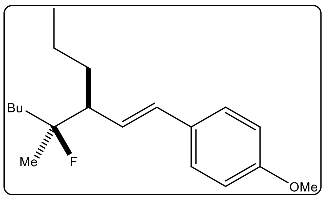

The title compound was prepared according to the general procedure C. The product was obtained as a colourless liquid (90%, 81 mg from 90 mg, *dr* = 85:15). R_f = 0.5 (petroleum ether /Et_2O = 9/1). ^{1}H NMR (400 MHz, Chloroform-*d*) δ 7.37 – 7.26 (m, 2H), 6.89 – 6.84 (m, 2H), 6.34 (d, *J* = 15.8 Hz, 1H), 5.78 (dd, *J* = 15.8, 9.7 Hz, 1H), 3.81 (s, 3H), 2.35 (dtd, *J* = 13.2, 10.4, 2.7 Hz, 1H), 1.70 – 1.58 (m, 3H), 1.41 – 1.18 (m, 10H), 0.91 (t, *J* = 7.2 Hz, 6H); ^{13}C NMR (101 MHz, Chloroform-*d*) δ 158.91, 131.77, 130.23, 127.86 (d, *J* = 7.8 Hz), 127.20, 113.93, 98.73 (d, *J* = 172.9 Hz), 55.30, 51.50 (d, *J* = 22.6 Hz), 38.73 (d, *J* = 22.9 Hz), 30.66 (d, *J* = 3.7 Hz), 25.20 (d, *J* = 4.6 Hz), 23.07, 21.35 (d, *J* = 25.1 Hz), 20.86, 14.08 (d, *J* = 2.3 Hz); ^{19}F NMR (377 MHz, Chloroform-*d*) δ -149.61; **HRMS** (APCI) $[M+H]^+$, calculated for $C_{19}H_{30}FO$; 293.2275; found 293.2265.

(5*S,6*S**,*E*)-5-allyl-6-fluoro-6-methyldeca-1,3-diene (4m)**

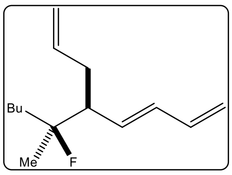

The title compound was prepared according to the general procedure C. The product was obtained as a colourless liquid (85%, 85 mg from 100 mg, *dr* = 90:10). R_f = 0.5 (petroleum ether /Et_2O = 9/1). ^{1}H NMR (400 MHz, Chloroform-*d*) δ 6.31 (dt, *J* = 16.7, 10.3 Hz, 1H), 6.06 (dd, *J* = 15.3, 10.4 Hz, 1H), 5.70 (ddt, *J* = 17.1, 10.1, 6.9 Hz, 1H), 5.42 (dd, *J* = 15.2, 9.6 Hz, 1H), 5.15 (d, *J* = 16.9 Hz, 1H), 5.06 – 4.94 (m, 3H), 2.48 (ddd, *J* = 14.0, 7.2, 3.2 Hz, 1H), 2.34 (ddd, *J* = 12.9, 10.0, 3.0 Hz, 1H), 2.06 (ddd, *J* = 13.7, 10.7, 6.7 Hz, 1H), 1.66 – 1.53 (m, 2H), 1.43 – 1.25 (m, 6H), 1.22 (s, 1H), 0.91 (t, *J* = 6.9 Hz, 3H); ^{13}C NMR (101 MHz, Chloroform-*d*) δ 137.12, 136.78, 133.93, 133.25 (d, *J* = 7.8 Hz), 116.10, 115.77, 98.34 (d, *J* = 173.7 Hz), 51.14 (d, *J* = 22.7 Hz), 38.65 (d, *J* = 22.6 Hz), 33.18 (d, *J* = 4.1 Hz), 25.12 (d, *J* = 4.4 Hz), 23.02, 21.11 (d, *J* = 25.0 Hz), 14.03; ^{19}F NMR (377 MHz, Chloroform-*d*) δ -149.77. **HRMS** (APCI) $[M-F]^+$, calculated for $C_{14}H_{23}$; 191.1794; found 191.1807.

(4*S,5*S**)-5-fluoro-5-methyl-4-((*E*)-prop-1-en-1-yl)non-1-ene (4n)**

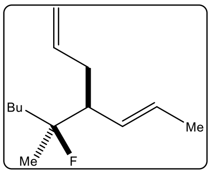

The title compound was prepared according to the general procedure C. The product was obtained as a colourless liquid (85%, 68 mg from 80 mg, *dr* = 88:12). R_f = 0.5 (petroleum ether /Et_2O = 9/1). ^{1}H NMR (400 MHz, Chloroform-*d*) δ 5.71 (ddt, *J* = 17.1, 10.1, 6.9 Hz, 1H), 5.44 (dddd, *J* = 15.9, 13.4, 9.2, 5.8 Hz, 1H), 5.13 (ddq, *J* = 15.0, 9.4, 1.7 Hz, 1H), 5.05 – 4.90 (m, 2H), 2.43 (dddt, *J* = 13.8, 7.1, 2.9, 1.3 Hz, 1H), 2.25 (qdd, *J* = 12.7, 9.3, 2.5 Hz, 1H), 2.00 (dddt, *J* = 13.6, 10.8, 6.8, 1.3 Hz, 1H), 1.72 – 1.64 (m, 3H), 1.63 – 1.51 (m, 2H), 1.46 – 1.26 (m, 5H), 1.25 (s, 1H), 1.20 (s, 1H), 0.91 (t, *J* = 7.2 Hz, 3H); ^{13}C NMR (101 MHz, Chloroform-*d*) δ 137.66, 129.96 (d, *J* = 8.1 Hz), 128.33, 115.30, 98.54 (d, *J* = 172.6 Hz), 51.07 (d, *J* = 22.3 Hz), 38.64 (d, *J* = 22.7 Hz), 33.22 (d, *J* = 4.3 Hz), 25.05 (d, *J* = 4.4 Hz), 23.06, 20.94 (d, *J* = 25.0 Hz), 18.02, 14.04; ^{19}F NMR (377 MHz, Chloroform-*d*) δ -149.94; **HRMS** (APCI) $[M-F]^+$, calculated for $C_{13}H_{23}$; 179.1800; found 179.1775.

(6*S,7*S**,*E*)-6-allyl-7-fluoro-2,7-dimethylundec-4-ene (4o)**

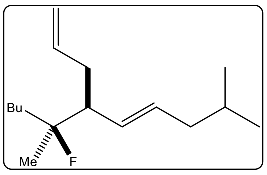

The title compound was prepared according to the general procedure C. The product was obtained as a colourless liquid (87%, 61 mg from 70 mg, *dr* = 90:10). R_f = 0.5 (petroleum ether /Et_2O = 9/1). ^{1}H NMR (400 MHz, Chloroform-*d*) δ 5.72 (ddt, *J* = 17.0, 10.3, 7.0 Hz, 1H), 5.43 (dt, *J* = 14.5, 7.0 Hz, 1H), 5.09 (dd, *J* = 15.3, 9.4 Hz, 1H), 5.02 – 4.87 (m, 2H), 2.53 – 2.36 (m, 1H), 2.27 (t, *J* = 11.6 Hz, 1H), 2.00 (td, *J* = 12.3, 11.5, 7.1 Hz, 1H), 1.90 (t, *J* = 7.0 Hz, 2H), 1.59 (ddt, *J* = 19.2, 11.9, 5.8 Hz, 3H), 1.41 – 1.24 (m, 6H), 1.21 (s, 1H), 0.89 (dd, *J* = 15.4, 7.0 Hz, 9H); ^{13}C NMR (101 MHz, Chloroform-*d*) δ 137.64, 132.82, 129.89 (d, *J* = 8.1 Hz), 115.39, 98.51 (d, *J* = 173.0 Hz), 51.05 (d, *J* = 22.2 Hz), 42.06, 38.83 (d, *J* = 22.8 Hz), 33.23 (d, *J* = 4.1 Hz), 28.45, 24.99 (d, *J* = 4.4 Hz), 23.07, 22.26 (d, *J* = 2.1 Hz), 20.98 (d, *J* = 25.0 Hz), 14.03; ^{19}F NMR (377 MHz, Chloroform-*d*) δ -150.16. **HRMS** (APCI) $[M-F]^+$, calculated for $C_{16}H_{29}$; 221.2264; found 221.2266.

((3*S,4*S**,*E*)-3-allyl-4-fluoro-4-methyloct-1-en-1-yl)cyclohexane (4p)**

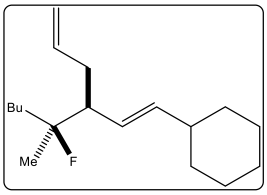

The title compound was prepared according to the general procedure C. The product was obtained as a colourless liquid (90%, 54 mg from 60 mg, *dr* = 90:10). R_f = 0.5 (petroleum ether /Et_2O = 9/1). ^{1}H NMR (400 MHz, Chloroform-*d*) δ 5.63 (ddt, *J* = 17.1, 10.1, 7.0 Hz, 1H), 5.31 (dd, *J* = 15.4, 6.8 Hz, 1H), 5.07 – 4.79 (m, 3H), 2.43 – 2.27 (m, 1H), 2.22 – 2.07 (m, 1H), 1.96 – 1.81 (m, 2H), 1.65 – 1.45 (m, 7H), 1.36 – 1.10 (m, 10H), 1.03 – 0.93 (m, 2H), 0.83 (t, *J* = 7.0 Hz, 3H); ^{13}C NMR (101 MHz, Chloroform-*d*) δ 140.04, 137.58, 126.09 (d, *J* = 8.3 Hz), 115.31, 98.56 (d, *J* = 172.8 Hz), 50.76 (d, *J* = 22.2 Hz), 40.83, 38.78 (d, *J* = 22.7 Hz), 33.19, 33.04, 26.16, 25.97, 24.95 (d, *J* = 4.4 Hz), 23.05, 20.97 (d, *J* = 25.0 Hz), 14.02; ^{19}F NMR (377 MHz, Chloroform-*d*) δ -150.21; **HRMS** (APCI) $[M-F]^+$, calculated for $C_{18}H_{31}$; 221.2266; found 247.2399.

((*S,*E*)-3-((*S**)-4-(benzyloxy)-2-fluorobutan-2-yl)hexa-1,5-dien-1-yl)benzene (4q)**

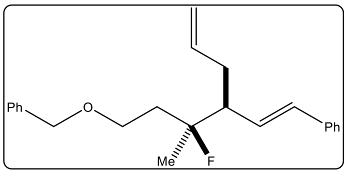

The title compound was prepared according to the general procedure C. The product was obtained as a colourless liquid (87%, 79 mg from 90 mg, *dr* = 88:12). R_f = 0.5 (petroleum ether /Et_2O = 9/1). ^{1}H NMR (400 MHz, Chloroform-*d*) δ 7.45 – 7.32 (m, 7H), 7.32 – 7.28 (m, 2H), 7.26 – 7.21 (m, 1H), 6.40 (d, *J* = 15.8 Hz, 1H), 5.94 (dd, *J* = 15.8, 9.5 Hz, 1H), 5.75 (ddt, *J* = 17.1, 10.1, 6.9 Hz, 1H), 5.09 – 4.94 (m, 2H), 4.52 (s, 2H), 3.69 (t, *J* = 7.0 Hz, 2H), 2.65 – 2.38 (m, 2H), 2.22 – 1.90 (m, 3H), 1.37 (d, *J* = 22.2 Hz, 3H); ^{13}C NMR (101 MHz, Chloroform-*d*) δ 138.26, 137.06, 136.84, 133.37, 128.49, 128.37, 127.46 (d, *J* = 22.1 Hz), 126.20, 116.02, 97.58 (d, *J* = 174.3 Hz), 73.12, 65.64 (d, *J* = 5.7 Hz), 52.23 (d, *J* = 22.4 Hz), 38.66 (d, *J* = 22.3 Hz), 33.20 (d, *J* = 4.3 Hz), 21.34 (d, *J* = 25.0 Hz); ^{19}F NMR (377 MHz, Chloroform-*d*) δ -148.76; **HRMS** (APCI) $[M-F]^+$, calculated for $C_{23}H_{27}O$; 319.2056; found 319.2066.

1-((*S,*E*)-3-((*S**)-4-(benzyloxy)-2-fluorobutan-2-yl)hexa-1,5-dien-1-yl)-4-methoxybenzene (4r)**

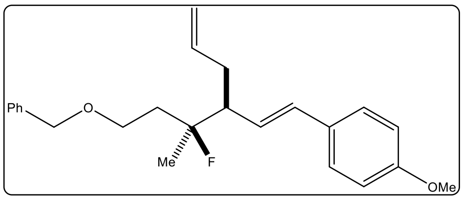

The title compound was prepared according to the general procedure C. The product was obtained as a colourless liquid (87%, 61 mg from 70 mg, *dr* > 95:05). R_f = 0.5 (petroleum ether /Et_2O = 9/1). ^{1}H NMR (400 MHz, Chloroform-*d*) δ 7.25 (d, *J* = 3.6 Hz, 4H), 7.23 – 7.16 (m, 3H), 6.76 (d, *J* = 8.7 Hz, 2H), 6.24 (d, *J* = 15.8 Hz, 1H), 5.66 (dddd, *J* = 17.1, 13.9, 9.8, 6.6 Hz, 2H), 5.02 – 4.81 (m, 2H), 4.43 (s, 2H), 3.73 (s, 3H), 3.59 (t, *J* = 7.1 Hz, 2H), 2.52 – 2.28 (m, 2H), 2.13 – 1.73 (m, 3H), 1.27 (d, *J* = 22.3 Hz, 3H); ^{13}C NMR (101 MHz, Chloroform-*d*) δ 159.03, 138.28, 137.00, 132.72, 129.91, 128.37, 127.67, 127.57, 127.33, 126.33 (d, *J* = 7.8 Hz), 115.90, 113.90, 97.69 (d, *J* = 174.0 Hz), 73.11, 65.68 (d, *J* = 5.6 Hz), 55.30, 52.25 (d, *J* = 22.3 Hz), 38.68 (d, *J* = 22.3 Hz), 33.27 (d, *J* = 4.4 Hz), 21.25 (d, *J* = 24.9 Hz); ^{19}F NMR (377 MHz, Chloroform-*d*) δ -148.69; **HRMS** (APCI) $[M+H]^+$, calculated for $C_{24}H_{30}FO_2$; 369.2224; found 369.2243.

(4*R,5*S**)-5-fluoro-4-(methoxymethyl)-2,5-dimethylnon-2-ene (4s)**

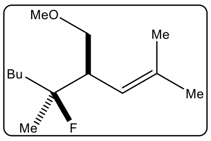

The title compound was prepared according to the general procedure C. The product was obtained as a colourless liquid (91%, 55 mg from 60 mg, *dr* = 88:12). R_f = 0.5 (petroleum ether /Et_2O = 9/1). ^{1}H NMR (400 MHz, Chloroform-*d*) δ 5.09 – 4.94 (m, 1H), 3.59 (dd, *J* = 9.3, 4.4 Hz, 1H), 3.36 (dd, *J* = 9.4, 7.5 Hz, 1H), 3.31 (s, 3H), 2.80 (dddd, *J* = 14.4, 10.2, 7.5, 4.4 Hz, 1H), 1.75 (d, *J* = 1.5 Hz, 3H), 1.66 (d, *J* = 1.4 Hz, 3H), 1.63 – 1.51 (m, 2H), 1.39 – 1.24 (m, 6H), 1.21 (s, 1H), 0.90 (t, *J* = 7.1 Hz, 3H); ^{13}C NMR (101 MHz, Chloroform-*d*) δ 134.94, 122.52 (d, *J* = 7.1 Hz), 98.89 (d, *J* = 172.1 Hz), 73.09 (d, *J* = 4.8 Hz), 58.83, 46.25 (d, *J* = 21.6 Hz), 38.65 (d, *J* = 22.7 Hz), 26.17, 25.23 (d, *J* = 4.8 Hz), 23.06, 21.47 (d, *J* = 25.1 Hz), 18.49, 14.02; ^{19}F NMR (377 MHz, Chloroform-*d*) δ -148.68; **HRMS** (APCI) $[M-F]^+$, calculated for $C_{13}H_{25}O$; 197.1905; found 197.1935.

((3*S,4*S**)-3-allyl-4-fluoro-4-methyloct-1-ene-1,1-diyl)dibenzene (4t)**

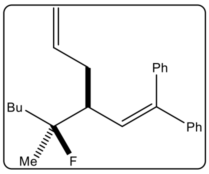

The title compound was prepared according to the general procedure C. The product was obtained as a colourless liquid (90%, 81 mg from 90 mg, *dr* = 90:10). R_f = 0.5 (petroleum ether /Et_2O = 9/1). ^1H NMR (400 MHz, Chloroform-*d*) δ 7.30 – 7.22 (m, 3H), 7.19 – 7.13 (m, 5H), 7.11 – 7.07 (m, 2H), 5.81 (d, *J* = 10.9 Hz, 1H), 5.66 (ddd, *J* = 17.1, 9.9, 7.2 Hz, 1H), 4.99 – 4.91 (m, 2H), 2.57 – 2.46 (m, 1H), 2.44 – 2.35 (m, 1H), 2.11 (ddd, *J* = 13.7, 10.0, 7.5 Hz, 1H), 1.56 – 1.40 (m, 3H), 1.27 (s, 1H), 1.22 (s, 2H), 1.15 – 1.02 (m, 3H), 0.75 (t, *J* = 7.1 Hz, 3H); ^{13}C NMR (101 MHz, Chloroform-*d*) δ 143.14 (d, *J* = 120.5 Hz), 139.91, 137.29, 129.90, 128.11, 127.20, 127.06 (d, *J* = 16.7 Hz), 115.99, 98.90 (d, *J* = 174.5 Hz), 46.80 (d, *J* = 22.2 Hz), 38.56 (d, *J* = 22.5 Hz), 34.31 (d, *J* = 4.7 Hz), 25.22 (d, *J* = 5.2 Hz), 23.02, 21.55 (d, *J* = 25.0 Hz), 13.92; ^{19}F NMR (377 MHz, Chloroform-*d*) δ -149.73; **HRMS** (APCI) $[M-F]^+$, calculated for $C_{24}H_{29}$; 317.2264; found 317.2249.

1-((3*S,4*S**,*E*)-3-benzyl-4-fluoro-4-methyloct-1-en-1-yl)-4-methoxybenzene (4u)**

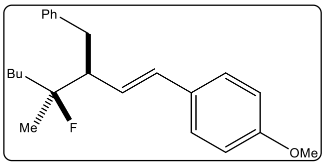

The title compound was prepared according to the general procedure C. The product was obtained as a colourless liquid (92%, 65 mg from 70 mg, *dr* = 93:07). R_f = 0.5 (petroleum ether /Et_2O = 9/1). ^1H NMR (400 MHz, Chloroform-*d*) δ 7.17 – 7.07 (m, 4H), 7.04 (d, *J* = 7.3 Hz, 3H), 6.78 – 6.70 (m, 2H), 5.90 (d, *J* = 15.7 Hz, 1H), 5.70 (dd, *J* = 15.7, 9.0 Hz, 1H), 3.69 (d, *J* = 1.9 Hz, 3H), 3.09 (d, *J* = 12.3 Hz, 1H), 2.50 (dq, *J* = 23.7, 11.4, 10.7 Hz, 2H), 1.60 (ddd, *J* = 21.5, 10.7, 6.5 Hz, 2H), 1.34 (d, *J* = 12.3 Hz, 3H), 1.30 – 1.15 (m, 4H), 0.87 – 0.79 (m, 3H); ^{13}C NMR (101 MHz, Chloroform-*d*) δ 158.90, 140.74, 132.54, 130.14, 129.40, 128.00, 127.19, 126.43 (d, *J* = 8.0 Hz), 125.67, 113.83, 98.59 (d, *J* = 174.0 Hz), 55.24, 53.99 (d, *J* = 22.7 Hz), 38.94 (d, *J* = 22.7 Hz), 35.51 (d, *J* = 4.2 Hz), 25.16 (d, *J* = 4.6 Hz), 23.03, 21.22 (d, *J* = 24.9 Hz), 14.06; ^{19}F NMR (377 MHz, Chloroform-*d*) δ -149.32; **HRMS** (APCI) $[M-F]^+$, calculated for $C_{23}H_{29}O$; 321.2213; found 321.2202.

1-((3*S,4*S**,*E*)-3-benzyl-7-chloro-4-fluoro-4-methylhept-1-en-1-yl)-4-methoxybenzene (4v)**

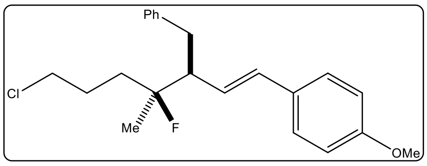

The title compound was prepared according to the general procedure C. The product was obtained as a colourless liquid (84%, 72 mg from 85 mg, *dr* > 95:05). R_f = 0.5 (petroleum ether /Et_2O = 9/1). ^1H NMR (400 MHz, Chloroform-*d*) δ 7.19 – 7.11 (m, 3H), 7.10 – 6.99 (m, 4H), 6.74 (d, *J* = 8.7 Hz, 2H), 5.94 (d, *J* = 15.8 Hz, 1H), 5.69 (dd, *J* = 15.8, 9.1 Hz, 1H), 3.71 (s, 3H), 3.47 (t, *J* = 6.4 Hz, 2H), 3.10 (dd, *J* = 12.5, 1.8 Hz, 1H), 2.62 – 2.42 (m, 2H), 1.96 – 1.82 (m, 2H), 1.81 – 1.67 (m, 2H), 1.33 (d, *J* = 21.9 Hz, 3H); ^{13}C NMR (101 MHz, Chloroform-*d*) δ 159.00, 140.44, 132.99, 129.88, 129.37, 128.06, 127.26, 125.83, 125.78, 113.85, 98.10 (d, *J* = 175.2 Hz), 55.27, 54.16 (d, *J* = 22.7 Hz), 45.21, 36.63 (d, *J* = 22.7 Hz), 35.49 (d, *J* = 4.1 Hz), 26.44 (d, *J* = 4.2 Hz), 20.91 (d, *J* = 24.8 Hz); ^{19}F NMR (377 MHz, Chloroform-*d*) δ -149.20; **HRMS** (APCI) $[M-F]^+$, calculated for $C_{22}H_{26}ClO$; 341.1667; found 341.1666.

1-((3*S,4*S**,*E*)-3-allyl-4-fluoro-4-methyloct-1-en-1-yl)-4-fluorobenzene (4w)**

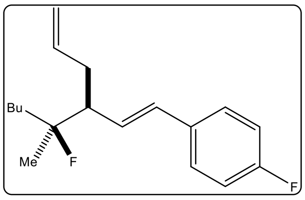

The title compound was prepared according to the general procedure C. The product was obtained as a colourless liquid (87%, 57 mg from 65 mg, *dr* = 88:12). R_f = 0.5 (petroleum ether /Et_2O = 9/1). ^{1}H NMR (400 MHz, Chloroform-*d*) δ 7.27 – 7.21 (m, 2H), 6.91 (t, *J* = 8.7 Hz, 2H), 6.27 (d, *J* = 15.8 Hz, 1H), 5.77 (dd, *J* = 15.8, 9.5 Hz, 1H), 5.66 (ddt, *J* = 17.1, 10.1, 6.9 Hz, 1H), 5.00 – 4.82 (m, 2H), 2.55 – 2.28 (m, 2H), 2.13 – 2.00 (m, 1H), 1.63 – 1.45 (m, 2H), 1.35 – 1.17 (m, 7H), 0.83 (t, *J* = 7.1 Hz, 3H); ^{13}C NMR (101 MHz, Chloroform-*d*) δ 162.13 (d, *J* = 246.4 Hz), 137.06, 133.38 (d, *J* = 3.3 Hz), 131.67, 128.86 (dd, *J* = 7.6, 2.4 Hz), 127.62 (d, *J* = 7.8 Hz), 115.93, 115.37 (d, *J* = 21.6 Hz), 98.38 (d, *J* = 173.7 Hz), 51.53 (d, *J* = 22.6 Hz), 38.63 (d, *J* = 22.7 Hz), 33.28 (d, *J* = 4.2 Hz), 25.21 (d, *J* = 4.7 Hz), 23.04, 21.38 (d, *J* = 25.0 Hz), 14.05; ^{19}F NMR (377 MHz, Chloroform-*d*) δ -114.94 (d, *J* = 1.9 Hz), -149.72; **HRMS** (APCI) $[M-F]^+$, calculated for $C_{18}H_{24}F$; 259.1857; found 259.1878.

1-((3*S,4*S**,*E*)-3-allyl-4-fluoro-4-methylhept-1-en-1-yl)-4-methoxybenzene (4x)**

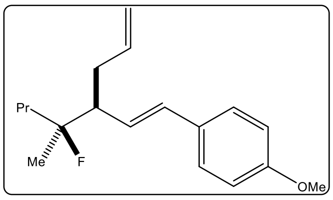

The title compound was prepared according to the general procedure C. The product was obtained as a colourless liquid (92%, 83 mg from 90 mg, *dr* = 91:09). R_f = 0.5 (petroleum ether /Et_2O = 9/1). ^{1}H NMR (400 MHz, Chloroform-*d*) δ 7.31 (d, *J* = 8.7 Hz, 2H), 6.87 (d, *J* = 8.7 Hz, 2H), 6.35 (d, *J* = 15.8 Hz, 1H), 5.98 – 5.58 (m, 2H), 5.16 – 4.82 (m, 2H), 3.82 (s, 3H), 2.61 – 2.35 (m, 2H), 2.23 – 2.07 (m, 1H), 1.69 – 1.57 (m, 2H), 1.53 – 1.40 (m, 2H), 1.34 (s, 1H), 1.28 (s, 2H), 0.93 (t, *J* = 7.2 Hz, 3H); ^{13}C NMR (101 MHz, Chloroform-*d*) δ 158.99, 137.25, 132.19, 130.05, 127.27, 126.87 (d, *J* = 7.9 Hz), 115.75, 113.92, 98.52 (d, *J* = 173.4 Hz), 55.29, 51.65 (d, *J* = 22.5 Hz), 41.27 (d, *J* = 22.7 Hz), 33.34 (d, *J* = 4.2 Hz), 21.09 (d, *J* = 25.0 Hz), 16.31 (d, *J* = 4.9 Hz), 14.46; ^{19}F NMR (377 MHz, Chloroform-*d*) δ -149.44; **HRMS** (APCI) $[M-F]^+$, calculated for $C_{18}H_{25}O$; 257.1905; found 257.1931.

((3*S,4*S**,*E*)-3-allyl-4-bromo-4-propyloct-1-en-1-yl)benzene (4y)**

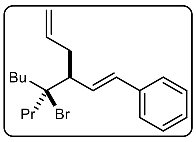

The title compound was prepared according to the general procedure C. The product was obtained as a colourless liquid (92%, 67 mg from 60 mg, *dr* = 95:05). R_f = 0.5 (petroleum ether /Et_2O = 9/1). ^{1}H NMR (400 MHz, Chloroform-*d*) δ 7.40 – 7.28 (m, 2H), 7.26 – 7.22 (m, 2H), 7.17 – 7.13 (m, 1H), 6.29 (d, *J* = 15.9 Hz, 1H), 6.08 (dd, *J* = 15.9, 8.7 Hz, 1H), 5.67 (ddt, *J* = 17.1, 10.3, 6.9 Hz, 1H), 5.00 – 4.85 (m, 2H), 2.55 – 2.45 (m, 1H), 2.29 – 2.18 (m, 2H), 1.96 – 1.77 (m, 4H), 1.37 – 1.20 (m, 6H), 0.87 – 0.78 (m, 6H); ^{13}C NMR (101 MHz, Chloroform-*d*) δ 137.26, 136.93, 132.68, 130.32, 128.50, 127.25, 126.25, 116.02, 52.72, 42.11, 40.32, 35.50, 27.17, 22.79, 18.26, 14.22, 14.04; **HRMS** (APCI) $[M-Br]^+$, calculated for $C_{20}H_{29}$; 269.2264; found 269.2273.

((3*S,4*R**,E)-3-allyl-4-bromo-4-propyloct-1-en-1-yl)benzene (4z)**

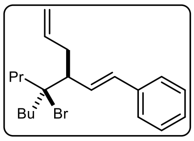

The title compound was prepared according to the general procedure C. The product was obtained as a colourless liquid (92%, 67 mg from 60 mg, *dr* = 95:05). R_f = 0.5 (petroleum ether /Et_2O = 9/1). ^{1}H NMR (400 MHz, Chloroform-*d*) δ 7.31 – 7.26 (m, 2H), 7.22 (dd, *J* = 8.4, 6.8 Hz, 2H), 7.15 – 7.10 (m, 1H), 6.27 (d, *J* = 15.9 Hz, 1H), 6.06 (dd, *J* = 15.9, 8.7 Hz,

1H), 5.72 – 5.56 (m, 1H), 4.99 – 4.82 (m, 2H), 2.53 – 2.44 (m, 1H), 2.27 – 2.17 (m, 2H), 1.94 – 1.81 (m, 3H), 1.71 – 1.60 (m, 1H), 1.39 – 1.23 (m, 6H), 0.84 – 0.77 (m, 6H); ^{13}C NMR (101 MHz, Chloroform-*d*) δ 137.25, 136.92, 132.67, 130.34, 128.50, 127.26, 126.25, 116.02, 81.99, 52.69, 42.78, 39.60, 35.46, 27.05, 22.83, 18.34, 14.17, 14.03; **HRMS** (APCI) [M+H]$^+$, calculated for $C_{20}H_{30}Br$; 349.1525; found 349.1500.

((3S,4S,E)-3-allyl-4-bromo-4-methyloct-1-en-1-yl)benzene (4aa)

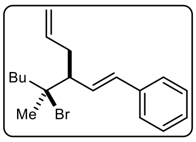

The title compound was prepared according to the general procedure C. The product was obtained as a colourless liquid (92%, 91 mg from 80 mg, *dr* = 95:05). R_f = 0.5 (petroleum ether /Et_2O = 9/1). ^{1}H NMR (400 MHz, Chloroform-*d*) δ 7.33 – 7.27 (m, 2H), 7.24 (dd, *J* = 8.5, 6.7 Hz, 2H), 7.19 – 7.13 (m, 1H), 6.31 (d, *J* = 15.8 Hz, 1H), 5.99 (dd, *J* = 15.8, 9.2 Hz, 1H), 5.67 (ddt, *J* = 17.0, 10.1, 6.9 Hz, 1H), 5.04 – 4.80 (m, 2H), 2.64 (dddt, *J* = 13.7, 7.2, 2.7, 1.3 Hz, 1H), 2.34 – 2.12 (m, 2H), 1.96 – 1.78 (m, 2H), 1.65 (s, 3H), 1.51 – 1.36 (m, 2H), 1.26 (q, *J* = 7.3 Hz, 2H), 0.86 (t, *J* = 7.2 Hz, 3H); ^{13}C NMR (101 MHz, Chloroform-*d*) δ 137.11, 136.82, 133.01, 129.75, 128.52, 127.34, 126.24, 116.08, 76.79, 54.53, 44.38, 35.93, 29.05, 27.62, 22.78, 14.04; **HRMS** (APCI) [M+H]$^+$, calculated for $C_{18}H_{26}Br$; 321.1220; found 321.1228.

((3*S,4*S**,*E*)-4-bromo-4-ethyl-3-propyloct-1-en-1-yl)benzene (4ab)**

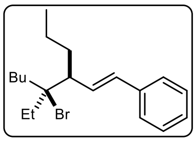

The title compound was prepared according to the general procedure C. The product was obtained as a colourless liquid (90%, 110 mg from 100 mg, *dr* = 95:05). R_f = 0.5 (petroleum ether /Et_2O = 9/1). ^{1}H NMR (400 MHz, Chloroform-*d*) δ 7.29 (dt, *J* = 6.1, 1.5 Hz, 2H), 7.22 (td, *J* = 7.5, 1.6 Hz, 2H), 7.14 (dq, *J* = 8.0, 1.9 Hz, 1H), 6.29 (d, *J* = 15.9 Hz, 1H), 6.06 (dd, *J* = 15.9, 9.3 Hz, 1H), 2.14 (td, *J* = 9.9, 9.4, 2.6 Hz, 1H), 2.02 – 1.84 (m, 3H), 1.73 (dt, *J* = 14.8, 7.4 Hz, 1H), 1.63 (dddd, *J* = 12.5, 9.3, 6.5, 2.5 Hz, 1H), 1.41 – 1.14 (m, 7H), 0.90 – 0.78 (m, 9H); ^{13}C NMR (101 MHz, Chloroform-*d*) δ 137.32, 132.24, 131.19, 128.52, 127.19, 126.17, 83.56, 52.10, 38.87, 33.42, 32.62, 26.98, 22.87, 20.78, 14.10, 14.04, 9.64; **HRMS** (APCI) [M-Br]$^+$, calculated for $C_{19}H_{29}$; 257.2264; found 257.2287.

Ethyl (2*R,3*S**)-3-bromo-3-methyl-2-((*E*)-styryl)heptanoate (4ac)**

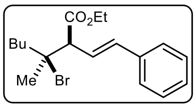

The title compound was prepared according to the general procedure C. The product was obtained as a colourless liquid (90%, 97 mg from 80 mg, *dr* > 95:05). R_f = 0.5 (petroleum ether /Et_2O = 8/2). ^{1}H NMR (400 MHz, Chloroform-*d*) δ 7.43 – 7.38 (m, 2H), 7.34 (dd, *J* = 8.3, 6.6 Hz, 2H), 7.28 (ddt, *J* = 7.2, 4.9, 2.4 Hz, 1H), 6.58 (d, *J* = 15.8 Hz, 1H), 6.33 (dd, *J* = 15.8, 9.6 Hz, 1H), 4.22 (dddd, *J* = 18.0, 10.8, 7.1, 3.7 Hz, 2H), 3.67 (d, *J* = 9.6 Hz, 1H), 1.91 (s, 3H), 1.88 (d, *J* = 2.8 Hz, 1H), 1.64 – 1.49 (m, 2H), 1.38 – 1.26 (m, 6H), 0.95 (t, *J* = 7.3 Hz, 3H); ^{13}C NMR (101 MHz, Chloroform-*d*) δ 170.92, 136.21, 135.03, 128.58, 128.01, 126.52, 123.83, 70.51, 61.34, 60.88, 43.25, 28.35, 27.65, 22.59, 14.13, 14.01; **HRMS** (APCI) [M+H]$^+$, calculated for $C_{18}H_{26}BrO_2$; 353.1116; found 353.1131.

1-((3*S,4*S**,*E*)-3-allyl-4-chloro-4-methyloct-1-en-1-yl)-4-methoxybenzene (4ad)**

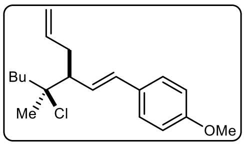

The title compound was prepared according to the general procedure C. The product was obtained as a colourless liquid (84%, 85 mg from 80 mg, *dr* > 95:05). R_f = 0.5 (petroleum ether /Et_2O = 9/1). ^1H NMR (400 MHz, Chloroform-*d*) δ 7.31 (d, *J* = 8.7 Hz, 2H), 6.86 (d, *J* = 8.7 Hz, 2H), 6.32 (d, *J* = 15.8 Hz, 1H), 5.88 (dd, *J* = 15.8, 9.5 Hz, 1H), 5.74 (ddt, *J* = 17.1, 10.1, 7.0 Hz, 1H), 5.08 – 4.93 (m, 2H), 3.81 (s, 3H), 2.72 – 2.62 (m, 1H), 2.43 (ddd, *J* = 12.1, 9.6, 2.7 Hz, 1H), 2.27 – 2.17 (m, 1H), 1.91 – 1.76 (m, 2H), 1.52 (s, 3H), 1.47 – 1.41 (m, 1H), 1.38 – 1.24 (m, 3H), 0.92 (t, *J* = 7.2 Hz, 3H); ^{13}C NMR (101 MHz, Chloroform-*d*) δ 158.98, 137.14, 132.39, 129.96, 127.32, 127.13, 115.89, 113.90, 77.42, 55.30, 53.91, 43.07, 34.59, 27.29, 26.51, 22.86, 14.06; **HRMS** (APCI) $[M+H]^+$, calculated for $C_{19}H_{28}ClO$; 307.1823; found 307.1827.

((1*E*,3*S,4*S**)-4-azido-4-methyl-3-propyloct-1-en-1-yl)benzene (4ae)**

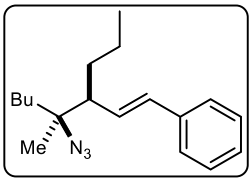

The title compound was prepared according to the general procedure E. The product was obtained as a colourless liquid (95%, 97 mg from 80 mg, *dr* = 86:14). R_f = 0.5 (petroleum ether /Et_2O = 9/1). ^1H NMR (400 MHz, $CDCl_3$) δ 7.29 (d, *J* = 7.2 Hz, 2H), 7.24 (t, *J* = 7.6 Hz, 2H), 7.14 (t, *J* = 7.0 Hz, 1H), 6.37 – 6.26 (m, 1H), 5.98 – 5.80 (m, 1H), 2.18 – 2.09 (m, 1H), 1.51 (dd, *J* = 9.3, 5.6 Hz, 3H), 1.37 – 1.22 (m, 5H), 1.21 (s, 1H), 1.15 (s, 3H), 1.13 – 1.05 (m, 1H), 0.88 – 0.80 (m, 6H); ^{13}C NMR (101 MHz, $CDCl_3$) δ 137.4, 132.9, 130.0, 128.7, 127.4, 126.3, 66.8, 51.6, 38.5, 31.3, 25.9, 23.2, 21.1, 20.7, 14.2, 14.2; **HRMS** (APCI) $[M-N_2+H]^+$,calculated for $C_{18}H_{28}N$: 258.2222; found 258.2236.

1-((1*E*,3*S,4*S**)-4-azido-4-methyl-3-propyloct-1-en-1-yl)-4-methoxybenzene (4af)**

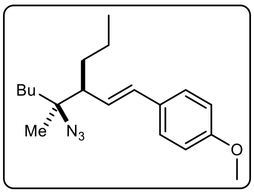

The title compound was prepared according to the general procedure E. The product was obtained as a colourless liquid (92%, 69 mg from 60 mg, *dr* > 95:05). R_f = 0.5 (petroleum ether /Et_2O = 9/1). ^1H NMR (400 MHz, $CDCl_3$) δ 7.38 – 7.28 (m, 2H), 6.92 – 6.83 (m, 2H), 6.33 (d, *J* = 15.8 Hz, 1H), 5.79 (dd, *J* = 15.7, 9.7 Hz, 1H), 3.81 (s, 3H), 2.29 – 2.11 (m, 1H), 1.67 – 1.55 (m, 3H), 1.47 – 1.25 (m, 7H), 1.23 (s, 3H), 1.20 – 1.10 (m, 1H), 0.91 (q, *J* = 7.0 Hz, 6H); ^{13}C NMR (101 MHz, $CDCl_3$) δ 159.1, 132.2, 130.3, 127.8, 127.4, 114.1, 66.9, 55.5, 51.6, 38.5, 31.4, 25.9, 23.2, 21.1, 20.7, 14.2; **HRMS** (APCI) $[M-N_2+H]^+$, calculated for $C_{19}H_{30}NO$: 288.2327; found: 288.2347.

1-((1*E*,3*S,4*R**)-4-azido-4-methyl-3-propyloct-1-en-1-yl)-4-methoxybenzene (4ag)**

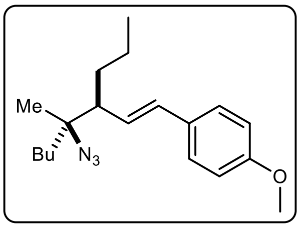

The title compound was prepared according to the general procedure E. The product was obtained as a colourless liquid (90%, 68 mg from 60 mg, *dr* > 95:05). R_f = 0.5 (petroleum ether /Et_2O = 9/1). ^1H NMR (400 MHz, $CDCl_3$) δ 7.31 (d, *J* = 8.7 Hz, 2H), 6.85 (d, *J* = 8.7 Hz, 2H), 6.33 (d, *J* = 15.8 Hz, 1H), 5.83 (dd, *J* = 15.8, 9.7 Hz, 1H), 3.81 (s, 3H), 2.20 (t, *J* = 11.5 Hz, 1H), 1.54 (d, *J* = 13.2 Hz, 4H), 1.38 (m, 5H), 1.27 (s, 3H), 1.19 (m, 1H), 0.90 (q, *J* = 6.8 Hz, 6H).; ^{13}C NMR (101 MHz, $CDCl_3$) δ 159.1, 132.2, 130.4, 127.9, 127.4, 114.1, 66.9, 55.5, 52.3, 37.6, 31.5, 26.0, 23.2, 21.4, 21.1, 14.2; **HRMS** (APCI) $[M-N_2+H]^+$, calculated for $C_{19}H_{30}NO$: 288.2327; found: 288.2331.

1-((1*E*,3*S,4*S**)-4-azido-4-methyl-3-propyloct-1-en-1-yl)-3-methoxybenzene (4ah)**

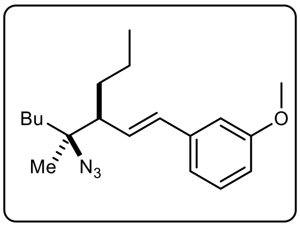

The title compound was prepared according to the general procedure E. The product was obtained as a colourless liquid (75%, 57 mg from 60 mg, *dr* = 89:11). R_f = 0.5 (petroleum ether /Et_2O = 9/1). ^{1}H NMR (400 MHz, $CDCl_3$) δ 7.26 – 7.08 (m, 1H), 6.89 (d, *J* = 7.7 Hz, 1H), 6.83 (d, *J* = 2.0 Hz, 1H), 6.71 (dd, *J* = 8.1, 2.1 Hz, 1H), 5.86 (dd, *J* = 15.8, 9.8 Hz, 1H), 3.75 (s, 3H), 2.18 – 2.08 (m, 1H), 1.57 – 1.46 (m, 3H), 1.40 – 1.23 (m, 7H), 1.15 (s, 3H), 0.83 (q, *J* = 7.0 Hz, 6H); ^{13}C NMR (101 MHz, $CDCl_3$) δ 160.0, 138.9, 132.7, 130.4, 129.7, 118.9, 112.9, 111.8, 66.8, 55.4, 51.6, 38.5, 31.3, 25.9, 23.2, 21.1, 20.8, 14.2; **HRMS** (APCI) $[M\text{-}N_2\text{+}H]^+$, calculated for $C_{19}H_{30}NO$: 288.2327; found: 288.2346.

1-((1,3*,4*)-4-azido-4-methyl-3-propyloct-1-en-1-yl)-2-methoxybenzene (4ai)

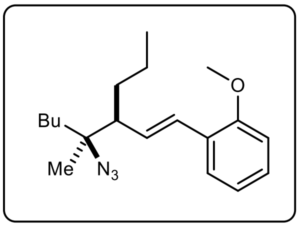

The title compound was prepared according to the general procedure E. The product was obtained as a colourless liquid (90%, 91 mg from 60 mg, *dr* = 92:08). R_f = 0.5 (petroleum ether /Et_2O = 9/1). ^{1}H NMR (400 MHz, $CDCl_3$) δ 7.45 (dd, *J* = 7.6, 1.6 Hz, 1H), 7.30 – 7.18 (m, 1H), 6.94 (t, *J* = 7.5 Hz, 1H), 6.88 (d, *J* = 8.2 Hz, 1H), 6.72 (d, *J* = 15.9 Hz, 1H), 6.05 – 5.88 (m, 1H), 3.85 (s, 3H), 2.31 – 2.19 (m, 1H), 1.67 – 1.58 (m, 3H), 1.50 – 1.27 (m, 7H), 1.25 (s, 3H), 0.97 – 0.86 (m, 6H); ^{13}C NMR (101 MHz, $CDCl_3$) δ 156.6, 130.4, 128.4, 127.5, 126.6, 120.7, 111.1, 66.9, 55.6, 51.8, 38.6, 31.4, 25.9, 23.2, 21.1, 20.7, 14.24, 14.21; **HRMS** (APCI) $[M\text{-}N_2\text{+}H]^+$,calculated for $C_{19}H_{30}NO$: 288.2327; found 288.2341.

((1*E*,3*S,4*S**)-4-azido-4-methyl-3-propyloct-1-en-1-yl)cyclohexane (4aj)**

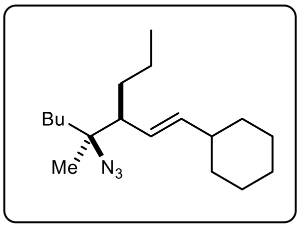

The title compound was prepared according to the general procedure E. The product was obtained as a colourless liquid (90%, 62 mg from 60 mg, *dr* = 83:17). R_f = 0.5 (petroleum ether /Et_2O = 9/1). ^{1}H NMR (400 MHz, $CDCl_3$) δ 5.47 – 5.30 (m, 1H), 5.17 – 4.98 (m, 1H), 2.03 – 1.91 (m, 2H), 1.68 (m, 6.4 Hz, 5H), 1.55 – 1.44 (m, 3H), 1.38 – 1.22 (m, 8H), 1.20 (s, 1H), 1.14 (s, 3H), 1.08 (m, 3H), 0.89 (dt, *J* = 14.2, 7.0 Hz, 6H); ^{13}C NMR (101 MHz, $CDCl_3$) δ 140.1, 126.7, 66.7, 50.9, 41.0, 38.4, 33.4, 33.2, 31.3, 26.3, 26.2, 25.7, 23.2, 20.9, 20.5, 14.2; **HRMS** (APCI) $[M\text{-}N_2\text{-}H_2]^+$,calculated for $C_{18}H_{31}N$: 264.4770; found: 261.2448.

1-((1*E*,3*S,4*S**)-4-azido-4-methyl-3-propyloct-1-en-1-yl)-4-fluorobenzene (4ak)**

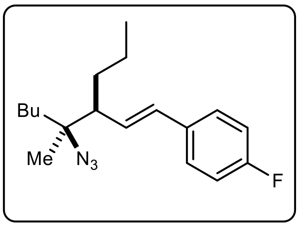

The title compound was prepared according to the general procedure E. The product was obtained as a colourless liquid (60%, 53 mg from 90 mg, *dr* = 81:19). R_f = 0.5 (petroleum ether /Et_2O = 9/1). ^{1}H NMR (400 MHz, Chloroform-*d*) δ 7.37 – 7.30 (m, 2H), 7.00 (t, *J* = 8.7 Hz, 2H), 6.35 (dd, *J* = 15.8, 5.0 Hz, 1H), 5.88 (ddd, *J* = 15.8, 9.7 Hz, 1H), 2.30 – 2.14 (m, 1H), 1.65 – 1.51 (m, 3H), 1.46 – 1.27 (m, 7H), 1.23 (s, 3H), 0.96 – 0.88 (m, 6H); ^{13}C NMR (101 MHz, $CDCl_3$) δ 163.5,

161.0, 133.6, 131.7, 129.8, 127.8, 127.7, 115.7, 115.4, 66.7, 51.7, 38.4, 31.3, 25.9, 23.2, 21.1, 20.9, 14.22, 14.19; ^{19}F NMR (377 MHz, $CDCl_3$) δ -114.99, -115.11; **HRMS** (APCI) $[M-N_2+H]^+$, calculated for $C_{18}H_{27}O_4FN$: 276.2128; found 276.2111.

1-((1*E*,3*S,4*S**)-4-azido-3-benzyl-4-methyloct-1-en-1-yl)-4-methoxybenzene (4al)**

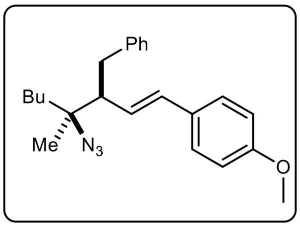

The title compound was prepared according to the general procedure E. The product was obtained as a colourless liquid (90%, 64 mg from 60 mg, *dr* > 95:05). R_f = 0.5 (petroleum ether /Et_2O = 9/1). ^{1}H NMR (400 MHz, $CDCl_3$) δ 7.25 – 7.10 (m, 7H), 6.82 (d, *J* = 8.7 Hz, 2H), 5.96 (d, *J* = 15.8 Hz, 1H), 5.82 (dd, *J* = 15.8, 9.2 Hz, 1H), 3.79 (s, 3H), 3.11 (dd, *J* = 13.0, 2.0 Hz, 1H), 2.53 (dd, *J* = 12.9, 10.9 Hz, 1H), 2.48 – 2.39 (m, 1H), 1.77 – 1.53 (m, 3H), 1.49 – 1.34 (m, 3H), 1.32 (s, 3H), 0.93 (t, *J* = 7.2 Hz, 3H); ^{13}C NMR (101 MHz, $CDCl_3$) δ 159.1, 140.7, 132.9, 130.2, 129.6, 128.2, 127.4, 126.3, 125.9, 114.0, 66.9, 55.4, 54.0, 38.7, 36.5, 25.9, 23.2, 20.7, 14.2; **HRMS** (APCI) $[M-N_2+H]^+$, calculated for $C_{23}H_{30}NO$: 336.2327; found: 336.2347.

Ethyl (2S,3S)-3-azido-2-((E)-2-methoxystyryl)-3-methylnonanoate (4am)

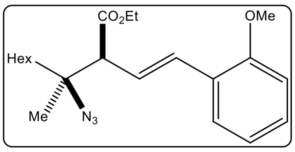

The title compound was prepared according to the general procedure E. The product was obtained as a colourless liquid (70%, 56 mg from 80 mg, *dr* = 88:12). R_f = 0.5 (petroleum ether /Et_2O = 9/1). ^{1}H NMR (400 MHz, Chloroform-*d*) δ 7.48 (dd, *J* = 7.6, 1.8 Hz, 1H), 7.25 (dt, *J* = 7.6, 2.0 Hz, 1H), 6.95 (t, *J* = 7.5 Hz, 1H), 6.89 (d, *J* = 8.2 Hz, 2H), 6.29 (dd, *J* = 16.0, 9.9 Hz, 1H), 4.20 (ddt, *J* = 11.6, 7.1, 4.1 Hz, 2H), 3.86 (s, 3H), 3.35 (d, *J* = 9.9 Hz, 1H), 1.64 (dd, *J* = 9.3, 6.0 Hz, 2H), 1.44 (d, *J* = 8.3 Hz, 2H), 1.40 (s, 3H), 1.31 (dq, *J* = 4.7, 3.1, 2.0 Hz, 9H), 0.89 (d, *J* = 6.8 Hz, 3H); ^{13}C NMR (101 MHz, Chloroform-*d*) δ 171.45, 156.63, 129.73, 129.00, 126.84, 125.37, 123.74, 120.59, 110.83, 65.31, 60.86, 58.39, 55.41, 38.26, 31.68, 29.43, 23.47, 22.54, 20.70, 14.12, 14.03; **HRMS** (APCI) $[M+H-N_2]^+$, calculated for $C_{21}H_{32}NO_3$; 346.2377; found 346.2391.

1-((3S,4S,E)-4-azido-3-(methoxymethyl)-4-methyloct-1-en-1-yl)-4-methoxybenzene (4an)

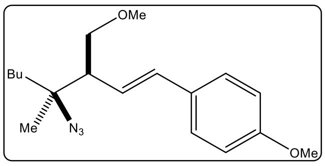

The title compound was prepared according to the general procedure E. The product was obtained as a colourless liquid (65%, 49 mg from 70 mg, *dr* = 87:13). R_f = 0.5 (petroleum ether /Et_2O = 9/1). ^{1}H NMR (400 MHz, Chloroform-*d*) δ 7.32 (d, *J* = 8.8 Hz, 2H), 6.85 (d, *J* = 8.7 Hz, 2H), 6.42 (d, *J* = 15.8 Hz, 1H), 5.95 (dd, *J* = 15.7, 9.5 Hz, 1H), 3.81 (s, 3H), 3.63 (dd, *J* = 9.4, 4.2 Hz, 1H), 3.50 – 3.43 (m, 1H), 3.33 (s, 3H), 2.51 (ddd, *J* = 9.3, 7.7, 4.1 Hz, 1H), 1.63 – 1.57 (m, 3H), 1.37 – 1.30 (m, 3H), 1.26 (s, 3H), 0.94 – 0.88 (m, 3H); ^{13}C NMR (101 MHz, Chloroform-*d*) δ 159.04, 132.84, 129.93, 127.44, 125.30, 113.88, 72.87, 65.65, 58.96, 55.30, 51.16, 38.46, 25.70, 22.97, 21.17, 14.06; **HRMS** (APCI) $[M+H-N_2]^+$, calculated for $C_{18}H_{28}NO_2$; 290.2115; found 290.2140.

2-((4S,5S)-5-azido-4-((E)-4-methoxystyryl)-5-methylnonyl)-4,4,5,5-tetramethyl-1,3,2-dioxaborolane (4ao)

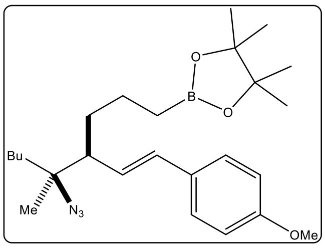

The title compound was prepared according to the general procedure E. The product was obtained as a colourless liquid (85%, 72 mg from 80 mg, *dr* > 95:05). R_f = 0.5 (petroleum ether /Et_2O = 9/1). ^{1}H NMR (400 MHz, Chloroform-*d*) δ 7.30 (d, *J* = 8.7 Hz, 2H), 6.85 (d, *J* = 8.7 Hz, 2H), 6.33 (d, *J* = 15.8 Hz, 1H), 5.78 (dd, *J* = 15.8, 9.7 Hz, 1H), 3.81 (s, 3H), 2.16 (td, *J* = 10.2, 2.2 Hz, 1H), 1.72 – 1.58 (m, 2H), 1.55 (d, *J* = 7.9 Hz, 2H), 1.43 – 1.26 (m, 6H), 1.23 (s, 12H), 1.20 (s, 3H), 0.90 (t, *J* = 6.9 Hz, 3H), 0.83 – 0.71 (m, 2H); ^{13}C NMR (101 MHz, Chloroform-*d*) δ 158.86, 132.02, 130.16, 127.63, 127.25, 113.87, 82.89, 66.76, 55.30, 51.48, 38.23, 31.74, 25.76, 24.77 (d, *J* = 1.5 Hz), 23.04, 22.18, 20.59, 14.09; ^{11}B NMR (128 MHz, Chloroform-*d*) δ 34.87; **HRMS** (APCI) $[M+H-N_2]^+$, calculated for $C_{25}H_{41}BNO_3$; 414.3174; found 414.3196.

((3*S,4*S**,*E*)-3-allyl-4-methyl-4-thiocyanatooct-1-en-1-yl)benzene (4ap)**

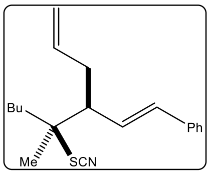

The title compound was prepared according to the general procedure F. The product was obtained as a colourless liquid (74%, 61 mg from 110 mg, *dr* > 95:05). R_f = 0.5 (petroleum ether /Et_2O = 9/1). ^{1}H NMR (400 MHz, Chloroform-*d*) δ 7.34 – 7.28 (m, 2H), 7.25 (dd, *J* = 8.5, 6.6 Hz, 2H), 7.21 – 7.14 (m, 1H), 6.36 (d, *J* = 15.7 Hz, 1H), 5.89 (dd, *J* = 15.7, 9.5 Hz, 1H), 5.71 – 5.55 (m, 1H), 5.04 – 4.88 (m, 2H), 2.57 – 2.39 (m, 2H), 2.19 – 2.05 (m, 1H), 1.75 (ddd, *J* = 9.2, 5.9, 2.3 Hz, 2H), 1.53 – 1.43 (m, 1H), 1.41 (s, 3H), 1.39 – 1.32 (m, 1H), 1.27 (h, *J* = 7.4, 6.8 Hz, 2H), 0.86 (t, *J* = 7.2 Hz, 3H); ^{13}C NMR (101 MHz, Chloroform-*d*) δ 136.62, 135.94, 134.31, 128.59, 127.68, 127.51, 126.32, 116.79, 111.89, 63.07, 52.43, 39.73, 34.59, 26.46, 23.59, 22.81, 13.96; **HRMS** (APCI) $[M+H]^+$, calculated for $C_{19}H_{26}NS$; 300.1780; found 300.1769.

1-((3*S,4*S**,*E*)-3-allyl-4-methyl-4-thiocyanatooct-1-en-1-yl)-4-methoxybenzene (4aq)**

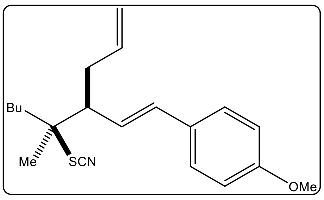

The title compound was prepared according to the general procedure F. The product was obtained as a colourless liquid (75%, 52 mg from 90 mg, *dr* > 95:05). R_f = 0.5 (petroleum ether /Et_2O = 9/1). ^{1}H NMR (400 MHz, Chloroform-*d*) δ 7.32 (d, *J* = 8.7 Hz, 2H), 6.87 (d, *J* = 8.7 Hz, 2H), 6.38 (d, *J* = 15.7 Hz, 1H), 5.82 (dd, *J* = 15.7, 9.5 Hz, 1H), 5.71 (ddt, *J* = 17.0, 10.1, 6.9 Hz, 1H), 5.12 – 4.95 (m, 2H), 3.81 (s, 3H), 2.58 (dddd, *J* = 12.3, 7.0, 2.8, 1.4 Hz, 1H), 2.49 (ddd, *J* = 12.1, 9.5, 2.7 Hz, 1H), 2.19 (dddt, *J* = 13.6, 11.0, 6.9, 1.2 Hz, 1H), 1.94 – 1.75 (m, 2H), 1.58 – 1.50 (m, 1H), 1.49 (s, 3H), 1.46 – 1.40 (m, 1H), 1.39 – 1.29 (m, 2H), 0.94 (t, *J* = 7.2 Hz, 3H); ^{13}C NMR (101 MHz, Chloroform-*d*) δ 159.25, 136.07, 133.66, 129.40, 127.46, 125.22, 116.62, 113.97, 111.96, 63.22, 55.27, 52.40, 39.67, 34.63, 26.43, 23.60, 22.79, 13.94; **HRMS** (APCI) $[M+H]^+$, calculated for $C_{20}H_{28}NOS$; 330.1892; found 330.1909.

1-((3*S,4*S**,*E*)-3-allyl-4-methyl-4-thiocyanatooct-1-en-1-yl)-3-methoxybenzene (4ar)**

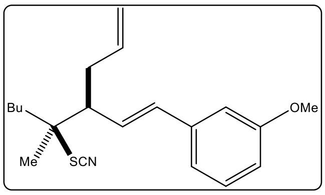

The title compound was prepared according to the general procedure F. The product was obtained as a colourless liquid (70%, 49 mg from 90 mg, *dr* > 95:05). R_f = 0.5 (petroleum ether /Et_2O = 9/1). ^{1}H NMR (400 MHz, Chloroform-*d*) δ 7.32 – 7.26 (m, 1H), 7.00 (dt, *J* = 7.7, 1.2 Hz, 1H), 6.94 (t, *J* = 2.0 Hz, 1H), 6.87 – 6.80 (m, 1H), 6.44 (d, *J* = 15.7 Hz, 1H), 5.99 (dd, *J* = 15.7, 9.6 Hz, 1H), 5.73 (ddt, *J* = 17.0, 10.1, 6.9 Hz, 1H), 5.14 – 4.98 (m, 2H), 3.86 (s, 3H), 2.60 (dd, *J* = 6.8, 1.4 Hz, 1H), 2.58 – 2.49 (m, 1H), 2.29 – 2.16 (m, 1H), 1.85 (ddd, *J* = 9.4, 5.8, 2.7 Hz, 2H), 1.60 – 1.52 (m, 1H), 1.51 (s, 3H), 1.49 – 1.42 (m, 1H), 1.42 – 1.32 (m, 2H), 0.96 (t, *J* = 7.2 Hz, 3H); ^{13}C NMR (101 MHz, Chloroform-*d*) δ 159.77, 138.04, 135.89, 134.16, 129.57, 127.85, 118.93, 116.81, 113.08, 111.90, 111.81, 63.03, 55.22, 52.40, 39.68, 34.56, 26.44, 23.56, 22.80, 13.97; **HRMS** (APCI) [M+H]$^+$, calculated for $C_{20}H_{28}NOS$; 330.1892; found 330.1921.

((3*S,4*S**,*E*)-3-allyl-4-ethyl-4-thiocyanatooct-1-en-1-yl)benzene (4as)**

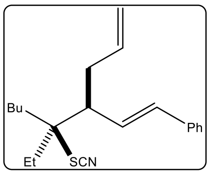

The title compound was prepared according to the general procedure F. The product was obtained as a colourless liquid (60%, 46 mg from 100 mg, *dr* = 86:14). R_f = 0.5 (petroleum ether /Et_2O = 9/1). ^{1}H NMR (400 MHz, Chloroform-*d*) δ 7.41 (d, *J* = 7.3 Hz, 2H), 7.35 (t, *J* = 7.5 Hz, 2H), 7.30 – 7.26 (m, 1H), 6.44 (d, *J* = 15.7 Hz, 1H), 6.10 (dd, *J* = 15.7, 9.6 Hz, 1H), 5.73 (ddt, *J* = 17.0, 10.2, 6.9 Hz, 1H), 5.12 – 4.98 (m, 2H), 2.66 – 2.54 (m, 1H), 2.50 (ddd, *J* = 14.0, 7.1, 2.7 Hz, 1H), 2.30 (ddd, *J* = 14.2, 11.0, 6.9 Hz, 1H), 2.01 – 1.77 (m, 4H), 1.51 – 1.36 (m, 4H), 1.08 (dt, *J* = 14.8, 7.3 Hz, 3H), 0.97 (dt, *J* = 13.5, 6.9 Hz, 3H); ^{13}C NMR (101 MHz, Chloroform-*d*) δ 136.63, 136.08, 134.28, 128.60, 127.81, 127.66, 126.37, 116.69, 112.48, 67.11, 51.38, 35.49, 34.19, 29.47, 26.05, 22.97, 13.97, 8.49; **HRMS** (APCI) [M+H]$^+$, calculated for $C_{20}H_{28}NS$; 314.1942; found 314.1931.

1-((3*S,4*S**,*E*)-3-allyl-4-methyl-4-thiocyanatooct-1-en-1-yl)-4-fluorobenzene (4at)**

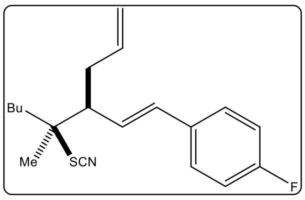

The title compound was prepared according to the general procedure F. The product was obtained as a colourless liquid (72%, 61 mg from 110 mg, *dr* = 95:05). R_f = 0.5 (petroleum ether /Et_2O = 9/1). ^{1}H NMR (400 MHz, Chloroform-*d*) δ 7.33 – 7.22 (m, 2H), 6.94 (t, *J* = 8.7 Hz, 2H), 6.32 (d, *J* = 15.8 Hz, 1H), 5.81 (dd, *J* = 15.7, 9.5 Hz, 1H), 5.62 (ddt, *J* = 17.0, 10.1, 6.9 Hz, 1H), 5.02 – 4.88 (m, 2H), 2.57 – 2.47 (m, 1H), 2.47 – 2.39 (m, 1H), 2.16 – 2.06 (m, 1H), 1.74 (ddd, *J* = 9.3, 5.8, 2.6 Hz, 2H), 1.54 – 1.43 (m, 1H), 1.41 (s, 3H), 1.36 (q, *J* = 4.1, 3.5 Hz, 1H), 1.28 (q, *J* = 7.1 Hz, 2H), 0.86 (t, *J* = 7.1 Hz, 3H); ^{13}C NMR (101 MHz, Chloroform-*d*) δ 162.33 (d, *J* = 246.9 Hz), 135.90, 133.10, 132.76 (d, *J* = 3.5 Hz), 127.84 (d, *J* = 8.0 Hz), 127.30 (d, *J* = 2.3 Hz), 116.85, 115.50 (d, *J* = 21.6 Hz), 111.91, 62.99, 52.50, 39.59, 34.56, 26.46, 23.70, 22.83, 13.98; ^{19}F NMR (377 MHz, Chloroform-*d*) δ -114.21; **HRMS** (APCI) [M+H]$^+$, calculated for $C_{19}H_{25}FNS$; 318.1692; found 318.1703.

(4*R,5*S**)-4-(methoxymethyl)-2,5-dimethyl-5-thiocyanatonon-2-ene (4au)**

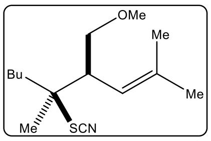

The title compound was prepared according to the general procedure F. The product was obtained as a colourless liquid (57%, 50 mg from 120 mg, *dr* = 88:12). R_f = 0.5 (petroleum ether /Et_2O = 9/1). ^{1}H NMR (400 MHz, Chloroform-*d*) δ 5.10 (dd, *J* = 10.3, 1.5 Hz, 1H),

3.54 (dd, J = 9.7, 5.1 Hz, 1H), 3.40 (dd, J = 9.7, 6.2 Hz, 1H), 3.30 (s, 3H), 2.84 (ddd, J = 10.2, 6.2, 5.1 Hz, 1H), 1.79 (dd, J = 6.4, 2.1 Hz, 2H), 1.76 (d, J = 1.4 Hz, 3H), 1.67 (d, J = 1.5 Hz, 3H), 1.45 (s, 3H), 1.43 – 1.23 (m, 4H), 0.92 (t, J = 7.1 Hz, 3H); ^{13}C NMR (101 MHz, Chloroform-*d*) δ 136.56, 121.07, 112.57, 73.58, 62.69, 58.86, 46.27, 39.74, 26.47, 26.14, 24.33, 22.84, 18.59, 13.94; **HRMS** (APCI) [M+H]$^+$, calculated for $C_{14}H_{26}NOS$; 256.1735; found 256.1736.

((4*R,5*S**,*E*)-4-(methoxymethyl)-5-methyl-5-thiocyanatonon-2-en-2-yl)benzene (4av)**

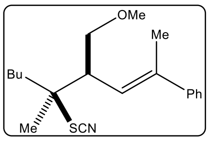

The title compound was prepared according to the general procedure F. The product was obtained as a colourless liquid (55%, 42 mg from 100 mg, *dr* = 86:14). R_f = 0.5 (petroleum ether /Et_2O = 9/1). ^{1}H NMR (400 MHz, Chloroform-*d*) δ 7.50 – 7.38 (m, 2H), 7.37 – 7.31 (m, 2H), 7.29 – 7.25 (m, 1H), 5.72 (dd, J = 10.4, 1.5 Hz, 1H), 3.66 (dd, J = 9.7, 5.1 Hz, 1H), 3.52 (dd, J = 9.7, 6.1 Hz, 1H), 3.33 (s, 3H), 3.07 (ddd, J = 10.3, 6.1, 5.0 Hz, 1H), 2.11 (d, J = 1.4 Hz, 3H), 1.94 – 1.79 (m, 2H), 1.54 (s, 3H), 1.51 – 1.43 (m, 2H), 1.39 – 1.31 (m, 2H), 0.93 (t, J = 7.3 Hz, 3H); ^{13}C NMR (101 MHz, Chloroform-*d*) δ 73.57, 69.42, 58.52, 57.51, 56.17, 54.80, 42.62, 3.65, -7.15, -10.77, -22.78, -29.79, -43.22, -45.31, -46.88, -52.91, -55.77; **HRMS** (APCI) [M+H]$^+$, calculated for $C_{29}H_{28}NOS$; 318.1892; found 318.1896.

4,4,5,5-tetramethyl-2-((4*S,5*S**)-5-methyl-4-((*E*)-styryl)-5-thiocyanatononyl)-1,3,2-dioxaborolane (4aw)**

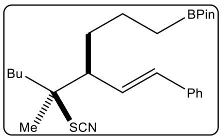

The title compound was prepared according to the general procedure F. The product was obtained as a colourless liquid (65%, 52 mg from 80 mg, *dr* > 95:05). R_f = 0.5 (petroleum ether /Et_2O = 8/2). ^{1}H NMR (400 MHz, Chloroform-*d*) δ 7.34 – 7.29 (m, 2H), 7.25 (t, J = 7.6 Hz, 2H), 7.20 – 7.16 (m, 1H), 6.38 (d, J = 15.7 Hz, 1H), 5.87 (dd, J = 15.7, 9.6 Hz, 1H), 2.34 (ddd, J = 11.4, 9.9, 2.3 Hz, 1H), 1.79 – 1.61 (m, 3H), 1.50 – 1.43 (m, 1H), 1.40 (s, 3H), 1.39 – 1.20 (m, 6H), 1.16 (s, 12H), 0.85 (t, J = 7.2 Hz, 3H), 0.77 – 0.64 (m, 2H); ^{13}C NMR (101 MHz, Chloroform-*d*) δ 136.79, 133.91, 128.56, 128.37, 127.54, 126.29, 112.17, 82.97, 63.56, 52.01, 39.76, 32.37, 26.47, 24.80, 23.98, 22.85, 22.20, 13.98; ^{11}B NMR (128 MHz, Chloroform-*d*) δ 34.6; **HRMS** (APCI) [M+H]$^+$, calculated for $C_{25}H_{39}BNO_2S$; 428.2789; found 428.2792.

(4*S,5*S**)-5-methyl-4-((*E*)-styryl)non-1-ene-5-thiol (6a)**

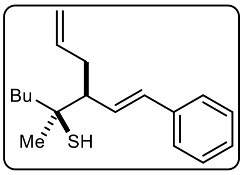

The title compound was prepared according to the general procedure F and our previous publication.[1e] The product was obtained as a colourless liquid (61%, 38 mg from 90 mg, *dr* > 95:05). R_f = 0.5 (petroleum ether /Et_2O = 9/1). ^{1}H NMR (400 MHz, Chloroform-*d*) δ 7.33 – 7.27 (m, 2H), 7.24 (t, J = 7.6 Hz, 2H), 7.19 – 7.13 (m, 1H), 6.28 (d, J = 15.8 Hz, 1H), 6.01 (dd, J = 15.8, 9.4 Hz, 1H), 5.66 (ddt, J = 17.0, 10.1, 6.9 Hz, 1H), 4.99 – 4.84 (m, 2H), 2.55 – 2.43 (m, 1H), 2.21 (td, J = 10.3, 9.5, 2.5 Hz, 1H), 2.15 – 2.03 (m, 1H), 1.58 (dd, J = 9.2, 7.0 Hz, 2H), 1.47 (s, 1H), 1.46 – 1.37 (m, 1H), 1.31 – 1.20 (m, 6H), 0.85 (t, J = 7.1 Hz, 3H); ^{13}C NMR (101 MHz, Chloroform-*d*) δ 137.41, 137.30, 132.65, 130.23, 128.49, 127.15, 126.16, 115.73, 53.57, 51.37, 43.15, 34.53, 27.69, 26.49, 23.09, 14.11; **HRMS** (APCI) [M+H]$^+$, calculated for $C_{18}H_{27}S$; 275.1833; found 275.1849.

Characterization spectra for starting materials

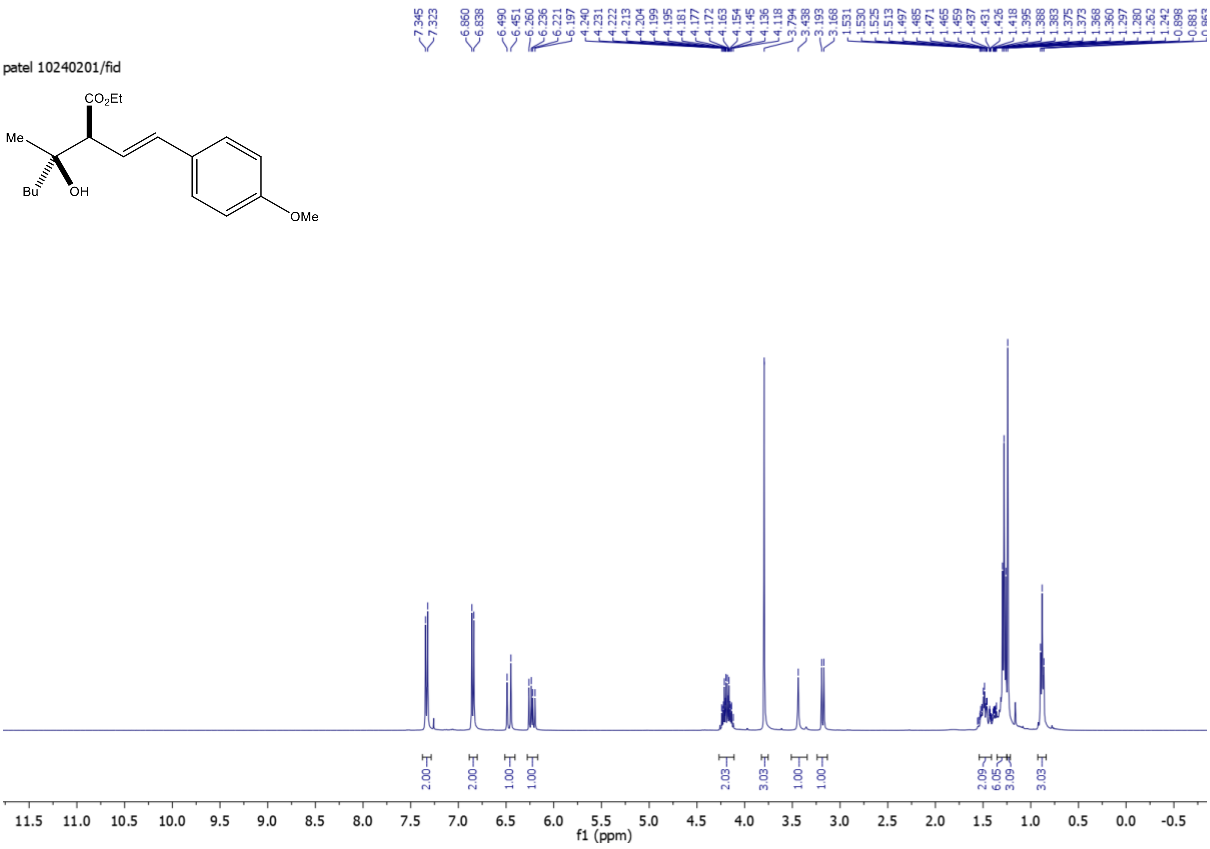

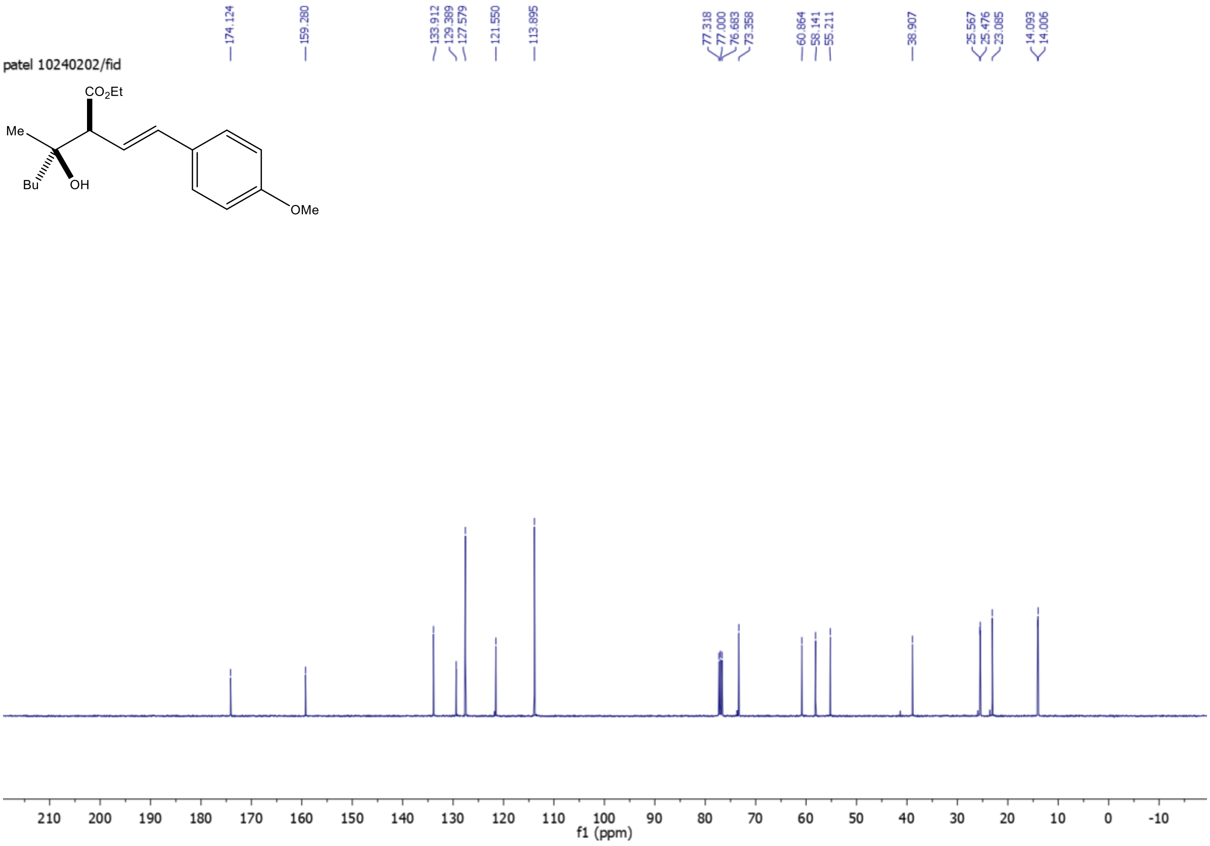

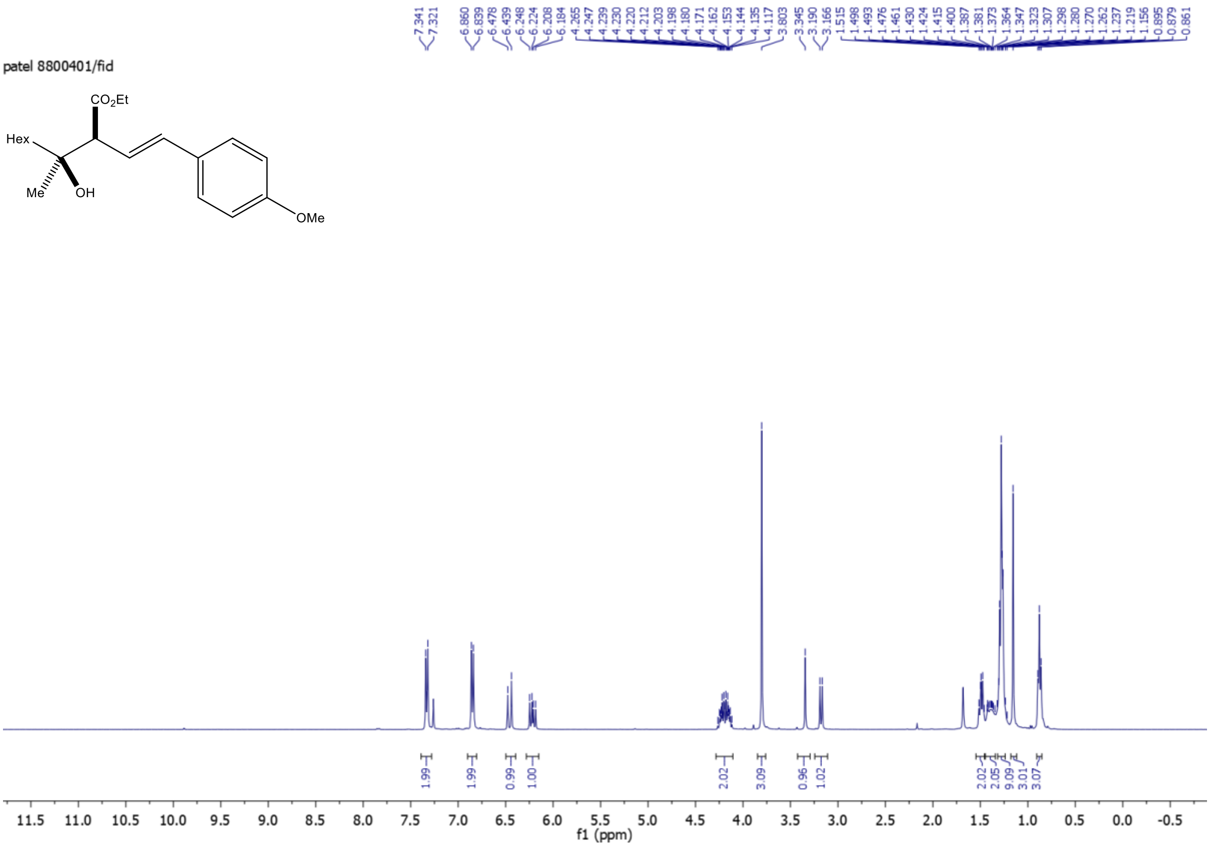
patel 8800401/fid
CO2Et
Hex
Me
OH
OMe
f1 (ppm)

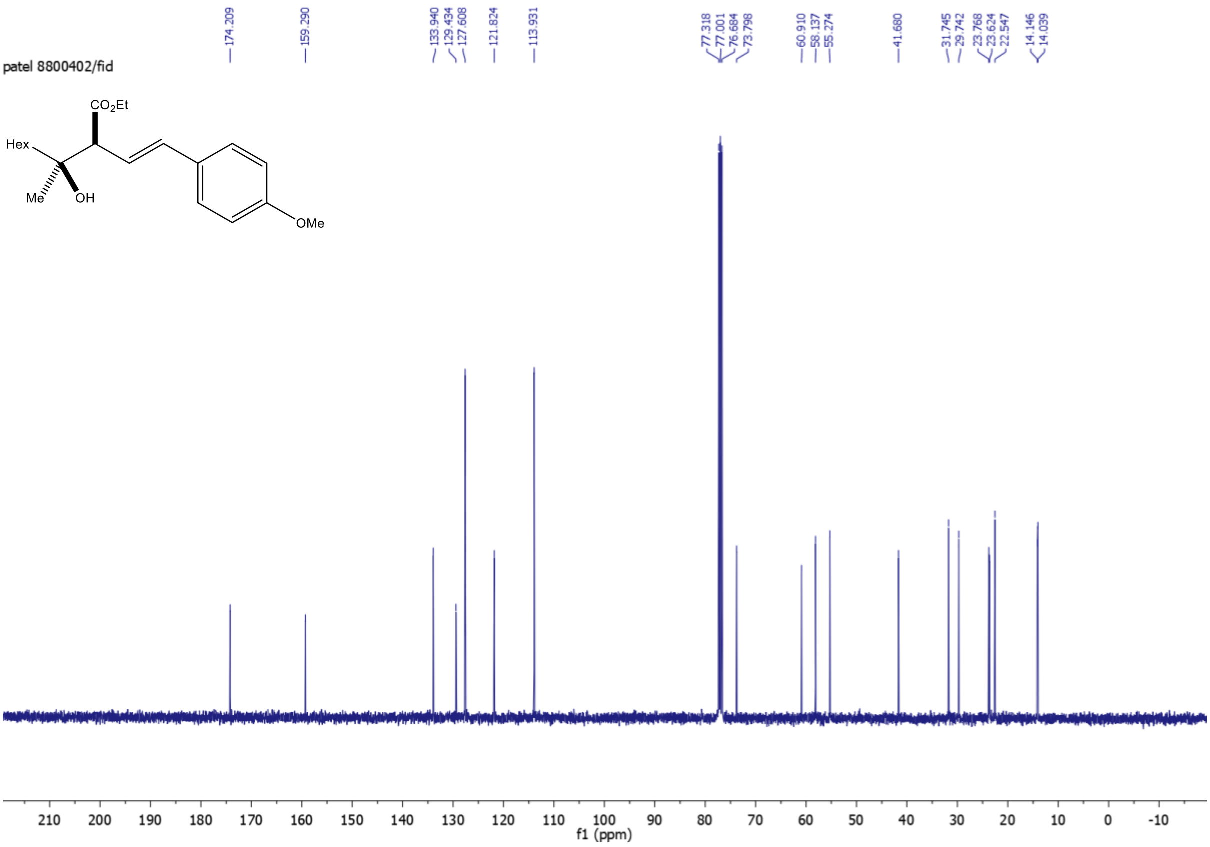
patel 8800402/fid
CO2Et
Hex
Me
OH
OMe
f1 (ppm)

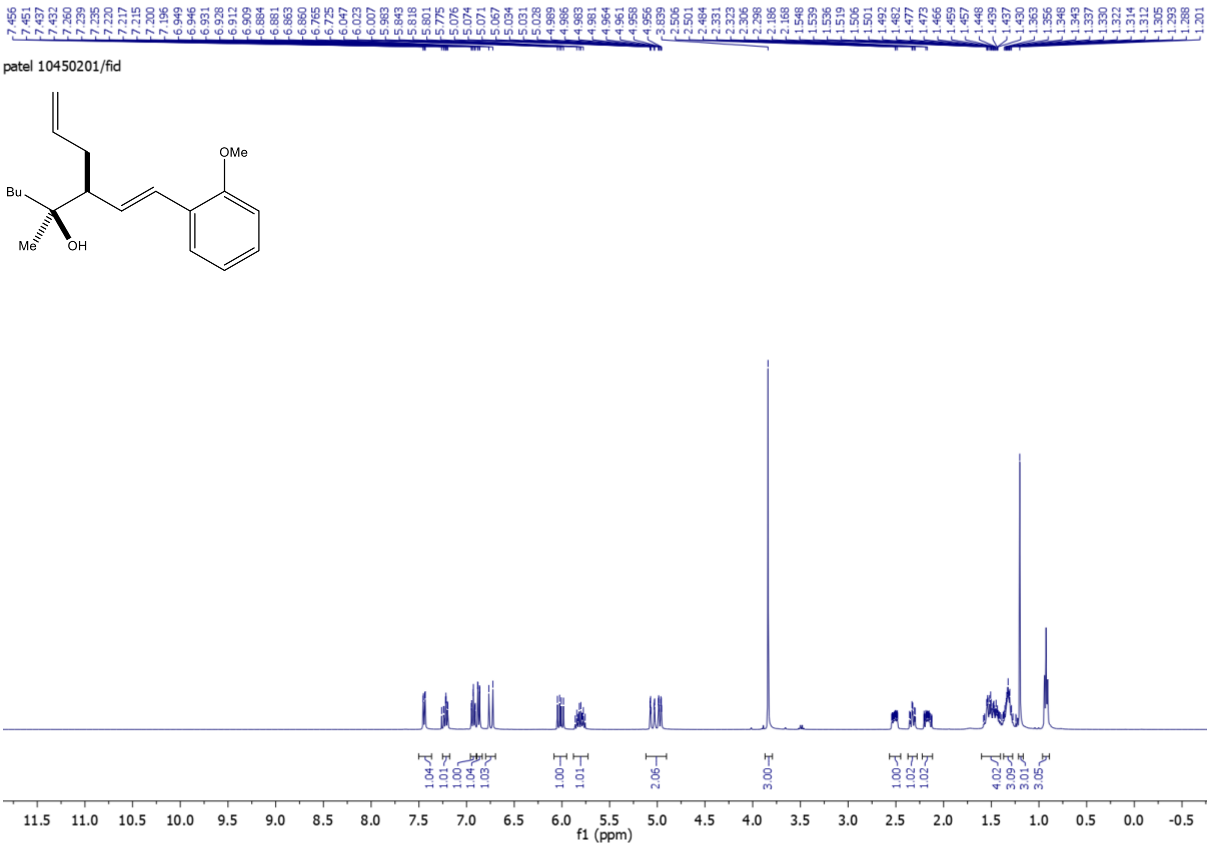
7.456 7.451 7.437 7.432 7.260 7.239 7.235 7.220 7.217 7.215 7.200 7.196 6.949 6.946 6.931 6.928 6.912 6.909 6.884 6.881 6.863 6.860 6.765 6.725 6.047 6.023 6.007 5.983 5.843 5.818 5.801 5.775 5.076 5.074 5.071 5.067 5.034 5.031 5.028 4.989 4.986 4.983 4.981 4.964 4.961 4.958 4.956 3.839 2.506 2.501 2.484 2.331 2.323 2.306 2.298 2.186 2.168 1.548 1.539 1.536 1.519 1.506 1.501 1.492 1.482 1.477 1.473 1.466 1.459 1.457 1.448 1.439 1.437 1.430 1.363 1.356 1.348 1.343 1.337 1.330 1.322 1.314 1.312 1.305 1.293 1.288 1.201
patel 10450201/fid
OMe
Bu
Me
OH
1.04 1.01 1.00 1.04 1.03 1.00 1.01 2.06 3.00 1.00 1.02 1.02 4.02 3.09 3.01 3.05
11.5 11.0 10.5 10.0 9.5 9.0 8.5 8.0 7.5 7.0 6.5 6.0 5.5 5.0 4.5 4.0 3.5 3.0 2.5 2.0 1.5 1.0 0.5 0.0 -0.5
f1 (ppm)

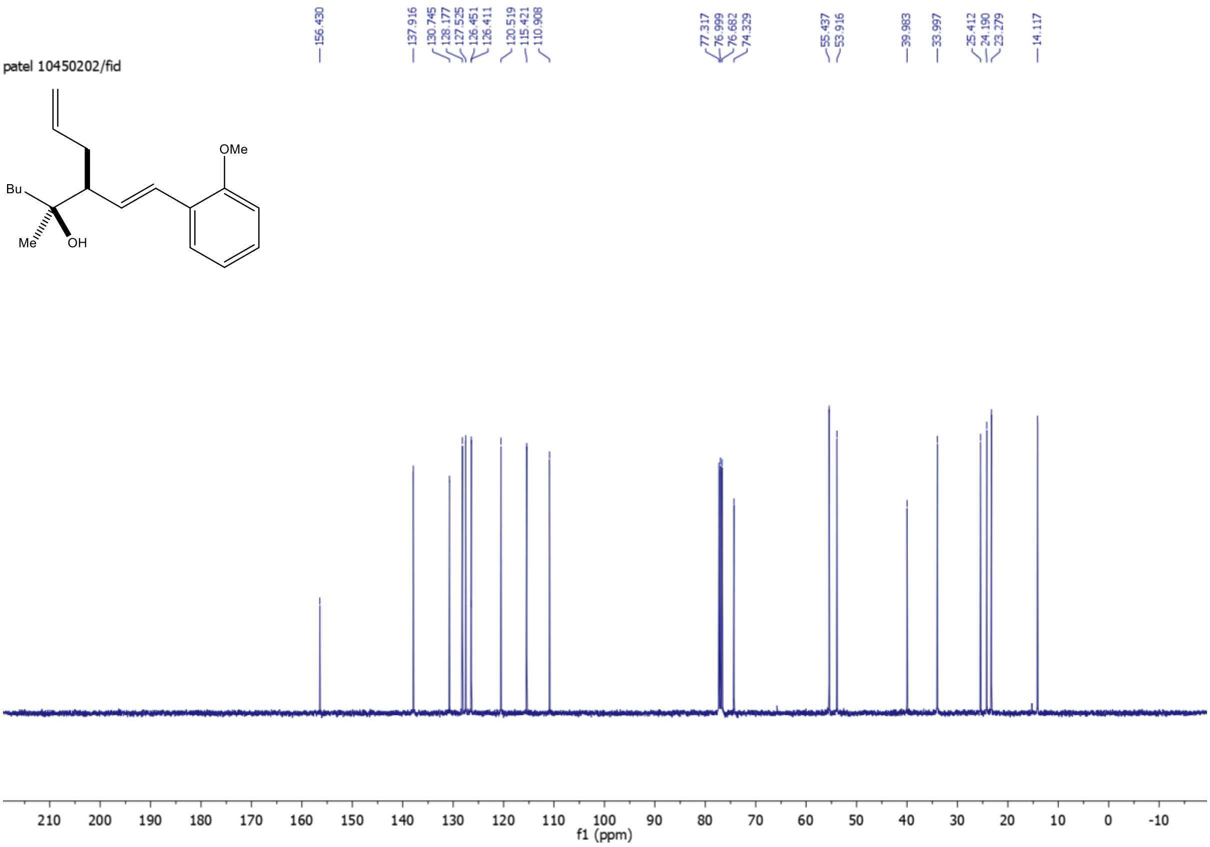
156.430 137.916 130.745 128.177 127.525 126.451 126.411 120.519 115.421 110.908 77.317 76.999 76.682 74.329 55.437 53.916 39.983 33.997 25.412 24.190 23.279 14.117
patel 10450202/fid
OMe
Bu
Me
OH
210 200 190 180 170 160 150 140 130 120 110 100 90 80 70 60 50 40 30 20 10 0 -10
f1 (ppm)

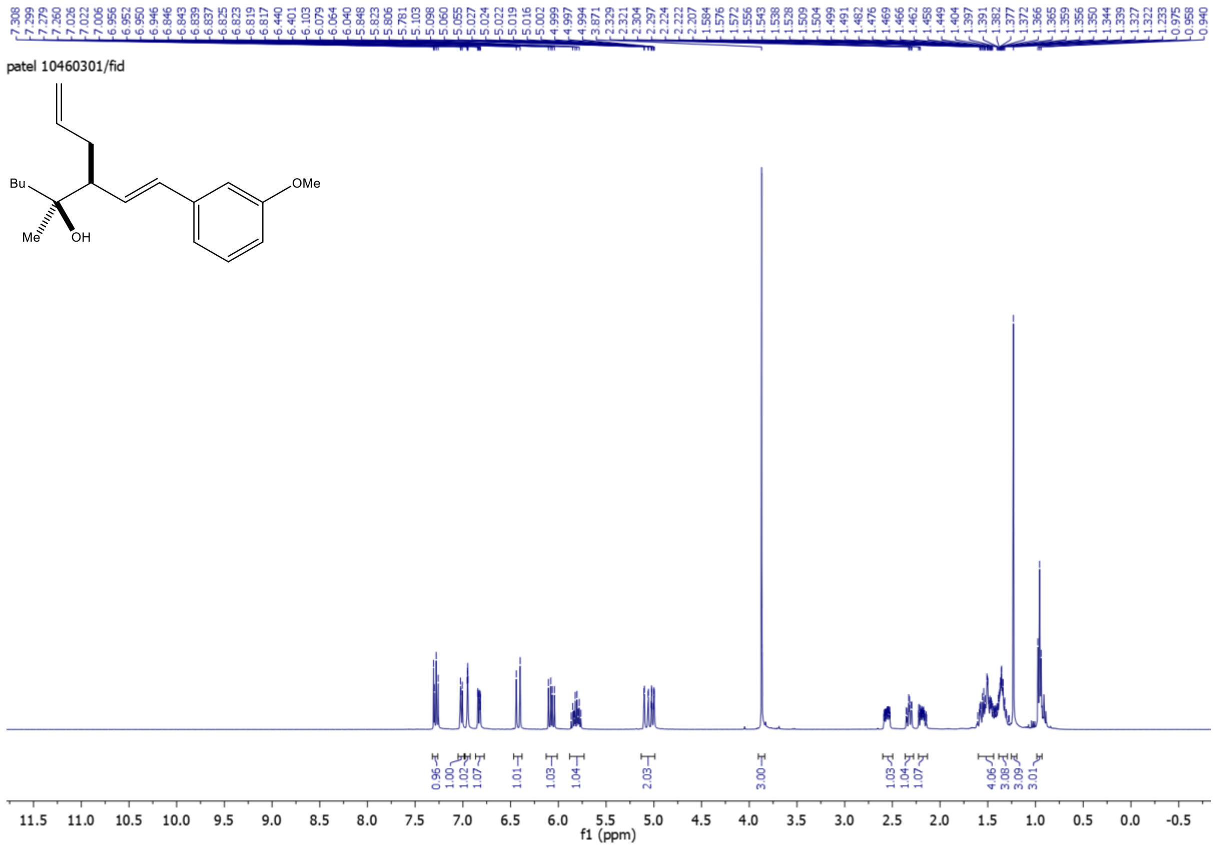
patel 10460301/fid
Bu
Me
OH
OMe
f1 (ppm)

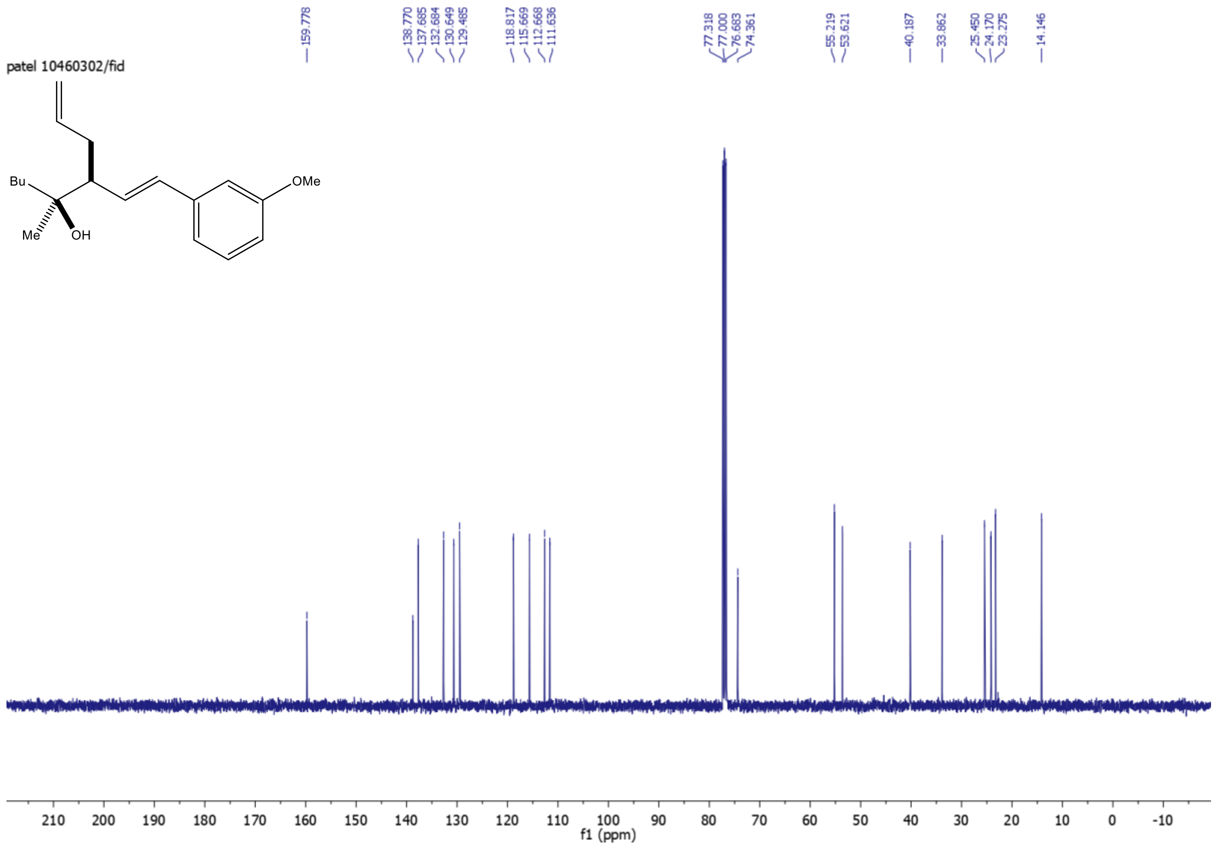
patel 10460302/fid
Bu
Me
OH
OMe
f1 (ppm)

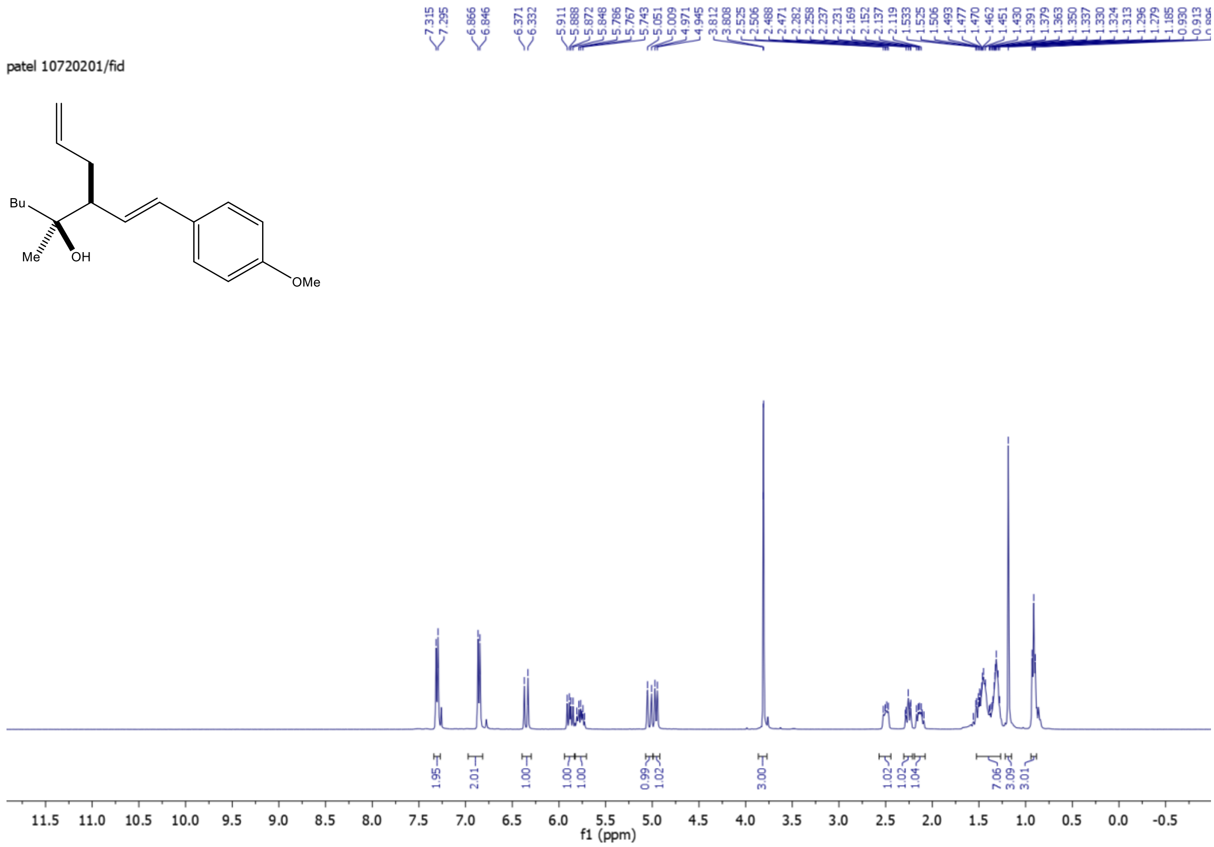
patel 10720201/fid
Bu
Me
OH
OMe
f1 (ppm)

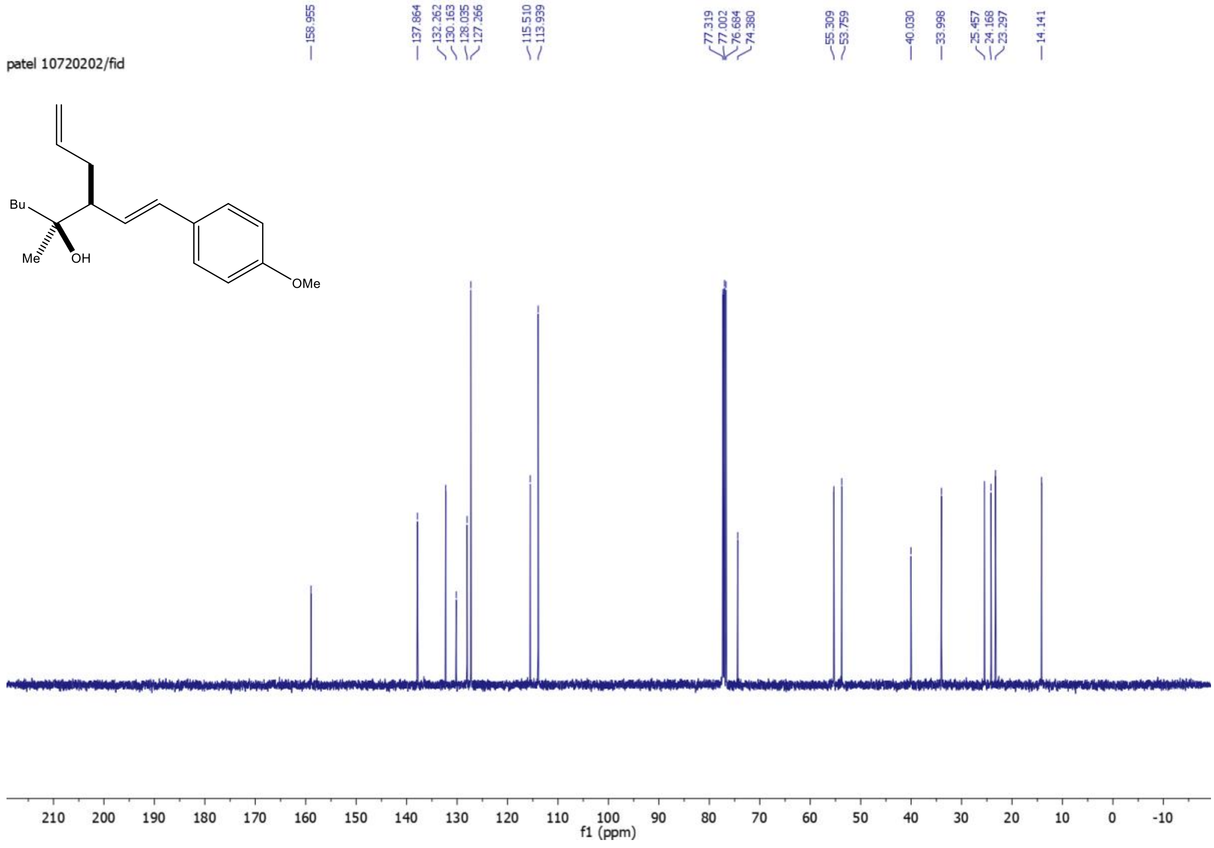
patel 10720202/fid
Bu
Me
OH
OMe
f1 (ppm)

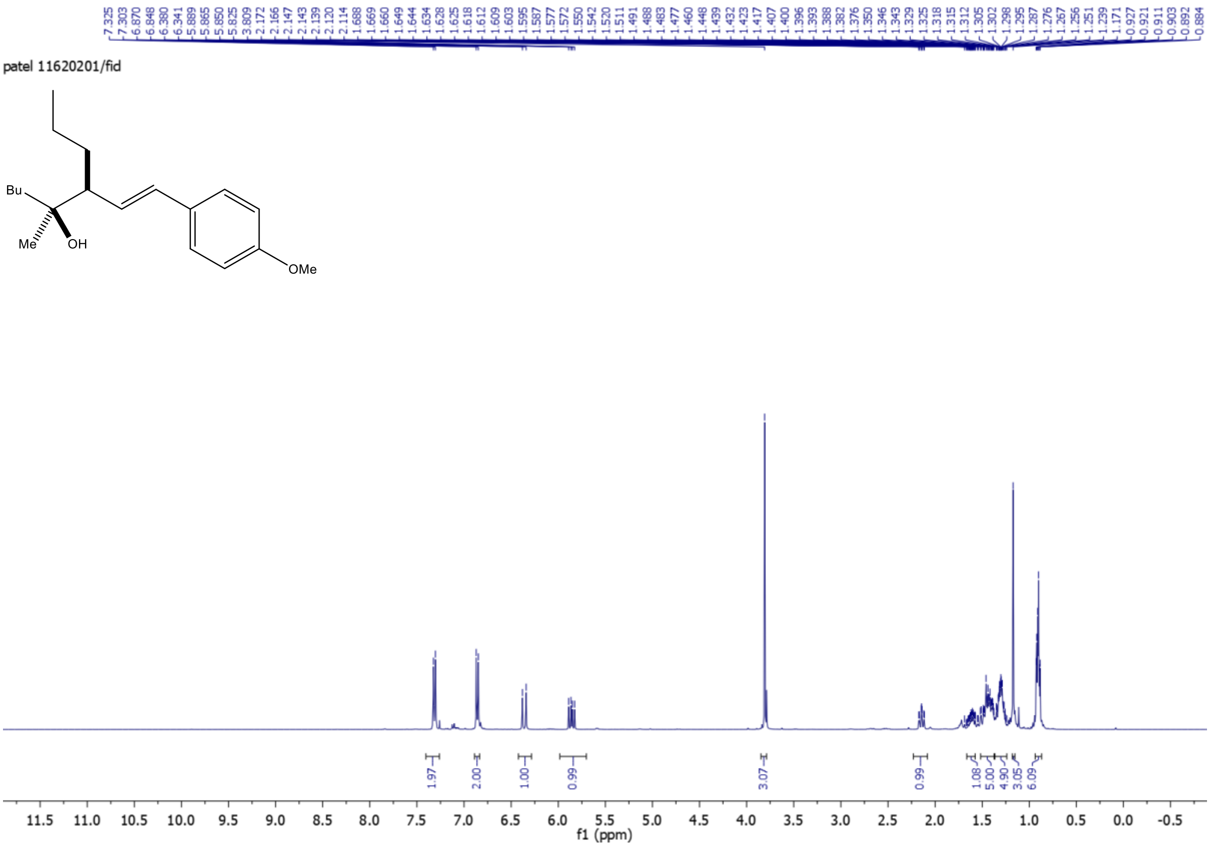
7.325 7.303 6.870 6.848 6.380 6.341 5.889 5.865 5.850 5.825 3.809 2.172 2.166 2.147 2.143 2.139 2.120 2.114 1.688 1.669 1.660 1.649 1.644 1.634 1.628 1.625 1.618 1.612 1.609 1.603 1.595 1.587 1.577 1.572 1.550 1.542 1.520 1.511 1.491 1.488 1.483 1.477 1.460 1.448 1.439 1.432 1.423 1.417 1.407 1.400 1.396 1.393 1.388 1.382 1.376 1.350 1.346 1.343 1.329 1.325 1.318 1.315 1.312 1.305 1.302 1.298 1.295 1.287 1.276 1.267 1.256 1.251 1.239 1.171 0.927 0.921 0.911 0.903 0.892 0.884
patel 11620201/fid
Bu
Me
OH
OMe
1.97
2.00
1.00
0.99
3.07
0.99
1.08
5.00
4.90
3.05
6.09
11.5 11.0 10.5 10.0 9.5 9.0 8.5 8.0 7.5 7.0 6.5 6.0 5.5 5.0 4.5 4.0 3.5 3.0 2.5 2.0 1.5 1.0 0.5 0.0 -0.5
f1 (ppm)

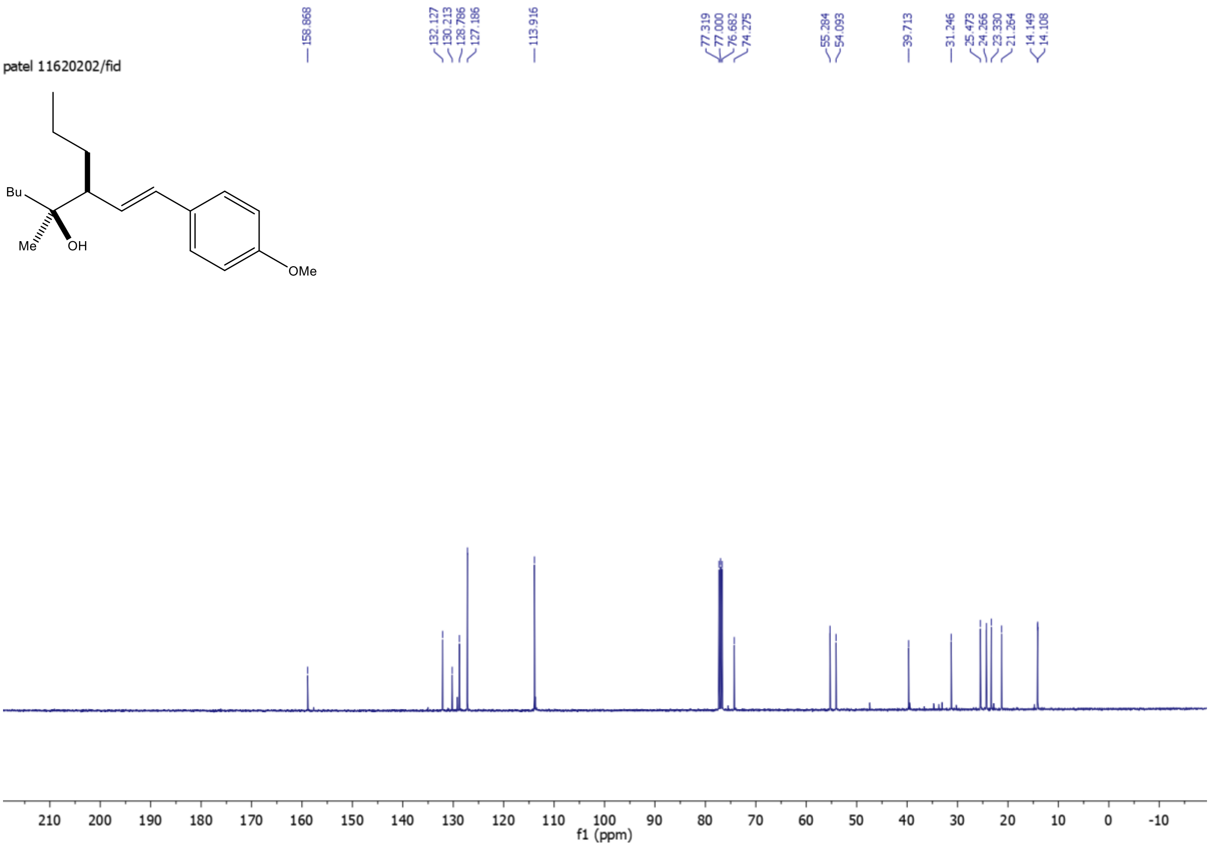
158.868
132.127 130.213 128.786 127.186
113.916
77.319 77.000 76.682 74.275
55.284 54.093
39.713
31.246 25.473 24.266 23.330 21.264
14.149 14.108
patel 11620202/fid
Bu
Me
OH
OMe
210 200 190 180 170 160 150 140 130 120 110 100 90 80 70 60 50 40 30 20 10 0 -10
f1 (ppm)

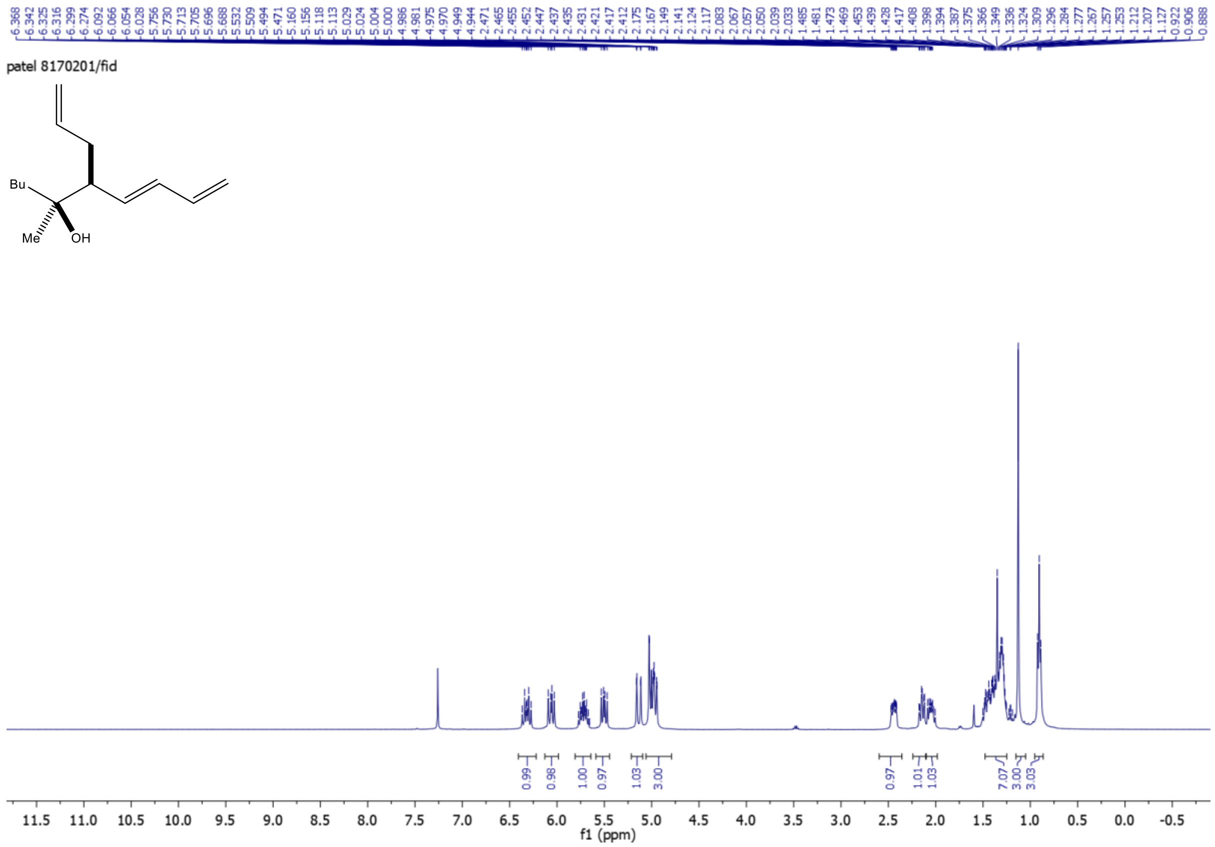
patel 8170201/fid
Bu
Me
OH
6.368 6.342 6.325 6.316 6.299 6.274 6.092 6.066 6.054 6.028 5.756 5.730 5.713 5.705 5.696 5.688 5.532 5.509 5.494 5.471 5.160 5.156 5.118 5.113 5.029 5.024 5.004 5.000 4.986 4.981 4.975 4.970 4.949 4.944 2.471 2.465 2.455 2.452 2.447 2.437 2.435 2.431 2.421 2.417 2.412 2.175 2.167 2.149 2.141 2.124 2.117 2.083 2.067 2.057 2.050 2.039 2.033 1.485 1.481 1.473 1.469 1.453 1.439 1.428 1.417 1.408 1.398 1.394 1.387 1.375 1.366 1.349 1.336 1.324 1.309 1.296 1.284 1.277 1.267 1.257 1.253 1.212 1.207 1.127 0.922 0.906 0.888
0.99 0.98 1.00 0.97 1.03 3.00 0.97 1.01 1.03 7.07 3.00 3.03
f1 (ppm)

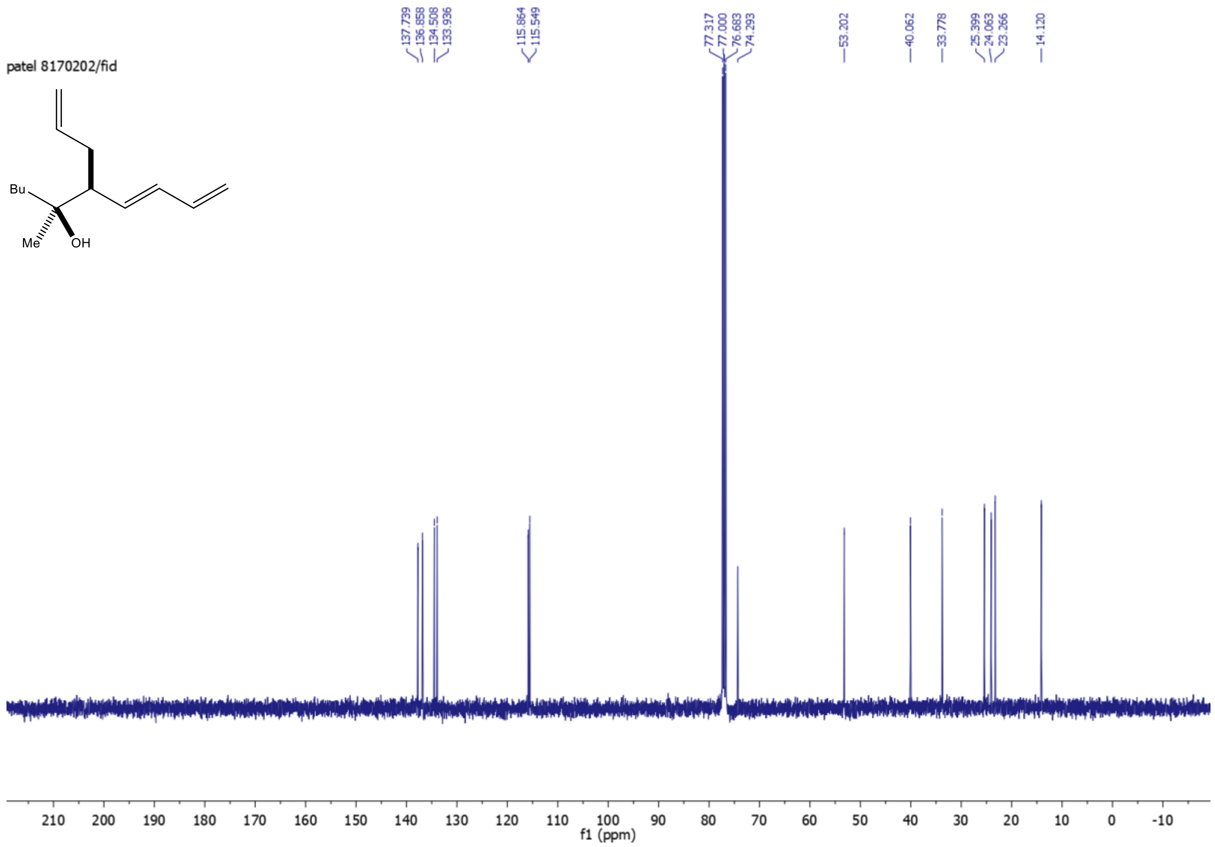
patel 8170202/fid
Bu
Me
OH
137.739 136.858 134.508 133.936 115.864 115.549 77.317 77.000 76.683 74.293 53.202 40.062 33.778 25.399 24.063 23.266 14.120
f1 (ppm)

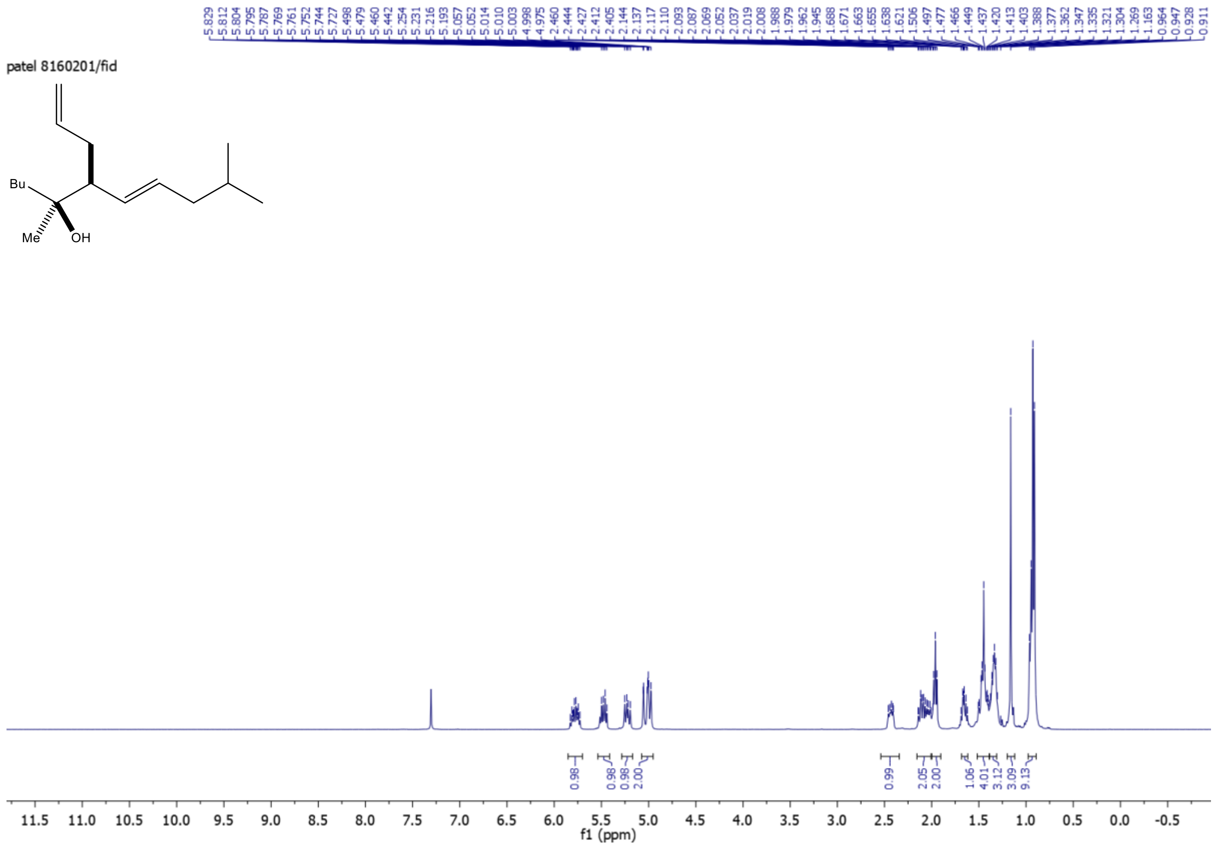
5.829 5.812 5.804 5.795 5.787 5.769 5.761 5.752 5.744 5.727 5.498 5.479 5.460 5.442 5.254 5.231 5.216 5.193 5.057 5.052 5.014 5.010 5.003 4.998 4.975 2.460 2.444 2.427 2.412 2.405 2.144 2.137 2.117 2.110 2.093 2.087 2.069 2.052 2.037 2.019 2.008 1.988 1.979 1.962 1.945 1.688 1.671 1.663 1.655 1.638 1.621 1.506 1.497 1.477 1.466 1.449 1.437 1.420 1.413 1.403 1.388 1.377 1.362 1.347 1.335 1.321 1.304 1.269 1.163 0.964 0.947 0.928 0.911
patel 8160201/fid
Bu
Me
OH
0.98
0.98
0.98
2.00
0.99
2.05
2.00
1.06
4.01
3.12
3.09
9.13
11.5 11.0 10.5 10.0 9.5 9.0 8.5 8.0 7.5 7.0 6.5 6.0 5.5 5.0 4.5 4.0 3.5 3.0 2.5 2.0 1.5 1.0 0.5 0.0 -0.5
f1 (ppm)

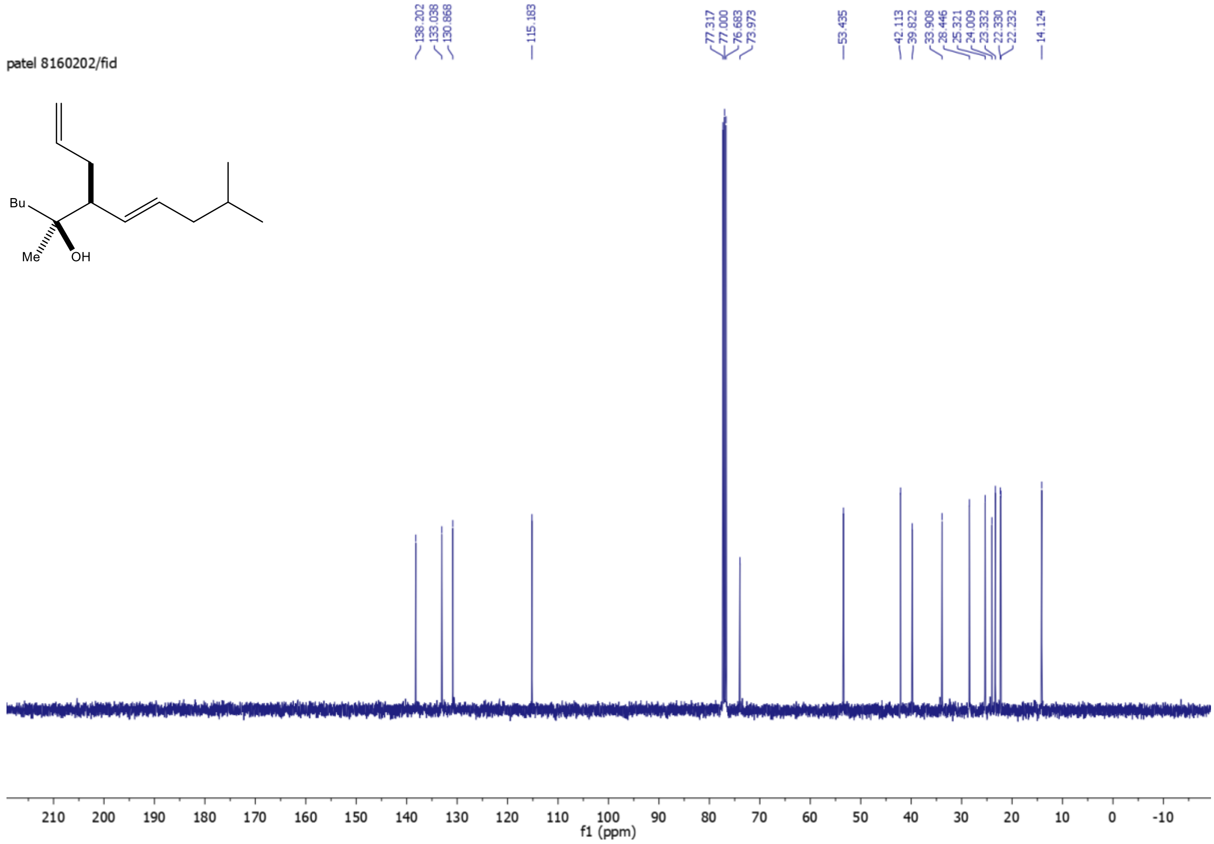
138.202 133.038 130.868
115.183
77.317 77.000 76.683 73.973
53.435
42.113 39.822 33.908 28.446 25.321 24.009 23.332 22.330 22.232
14.124
patel 8160202/fid
Bu
Me
OH
210 200 190 180 170 160 150 140 130 120 110 100 90 80 70 60 50 40 30 20 10 0 -10
f1 (ppm)

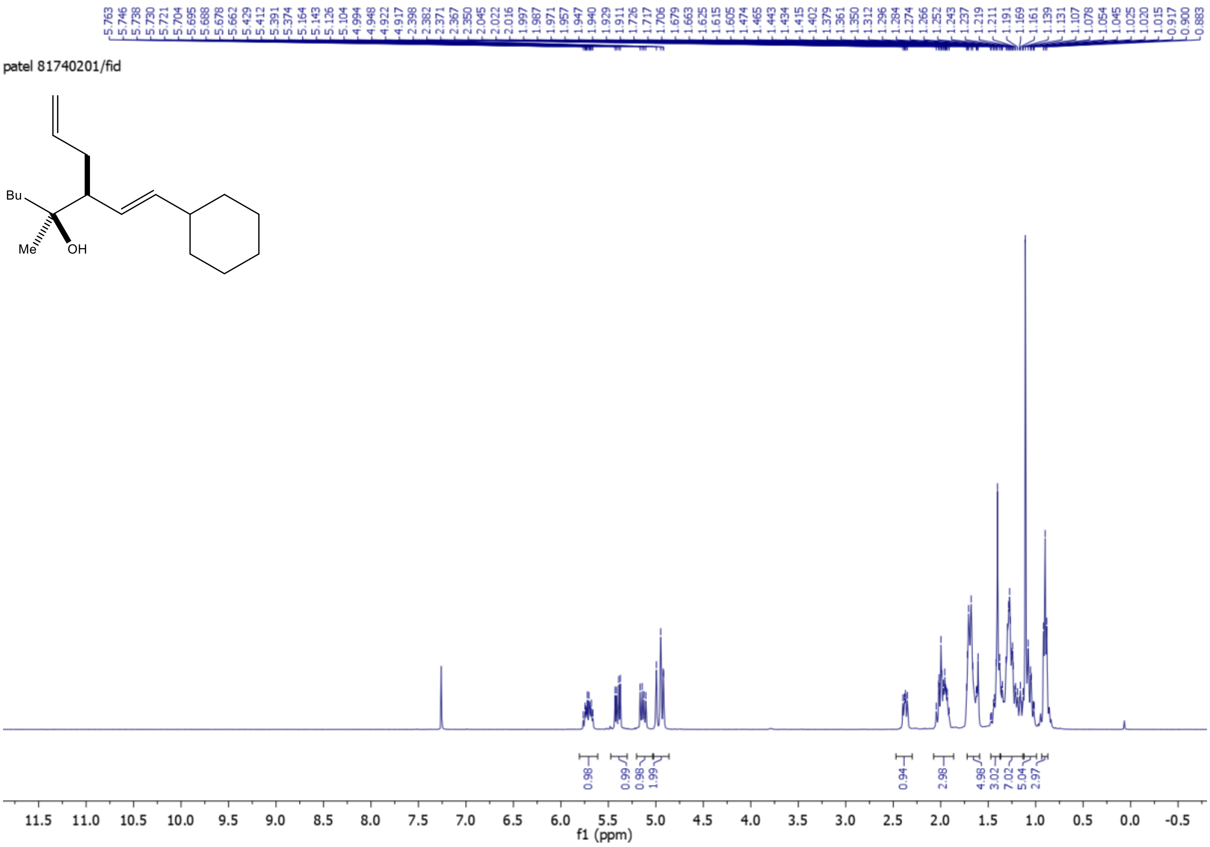
patel 81740201/fid
Bu
Me
OH
5.763 5.746 5.738 5.730 5.721 5.704 5.695 5.688 5.678 5.662 5.429 5.412 5.391 5.374 5.164 5.143 5.126 5.104 4.994 4.948 4.922 4.917 2.398 2.382 2.371 2.367 2.350 2.045 2.022 2.016 1.997 1.987 1.971 1.957 1.947 1.940 1.929 1.911 1.726 1.717 1.706 1.679 1.663 1.625 1.615 1.605 1.474 1.465 1.443 1.434 1.415 1.402 1.379 1.361 1.350 1.312 1.296 1.284 1.274 1.266 1.252 1.243 1.237 1.219 1.211 1.191 1.169 1.161 1.139 1.131 1.107 1.078 1.054 1.045 1.025 1.020 1.015 0.917 0.900 0.883
0.98 0.99 0.98 1.99 0.94 2.98 4.98 3.02 7.02 5.04 2.97
f1 (ppm)

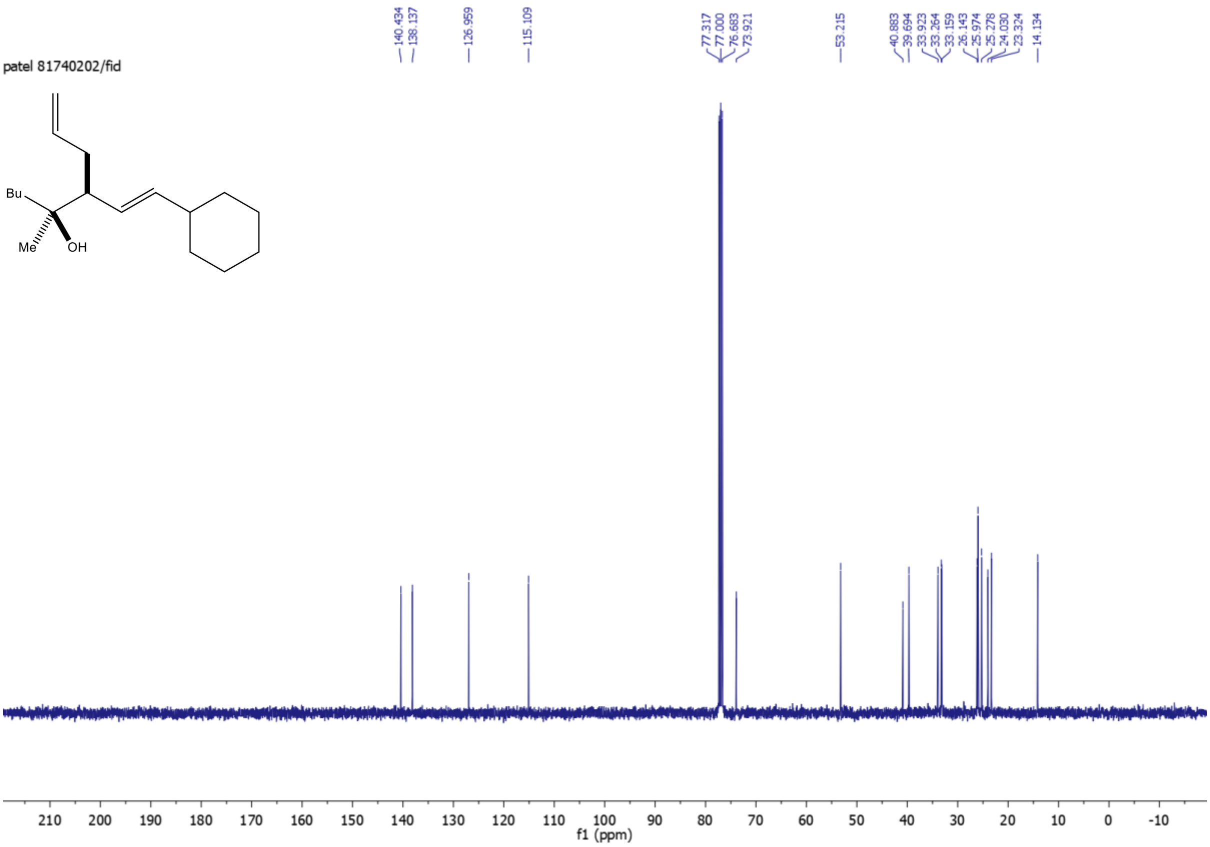
patel 81740202/fid
Bu
Me
OH
140.434 138.137 126.959 115.109 77.317 77.000 76.683 73.921 53.215 40.883 39.694 33.923 33.264 33.159 26.143 25.974 25.278 24.030 23.324 14.134
f1 (ppm)

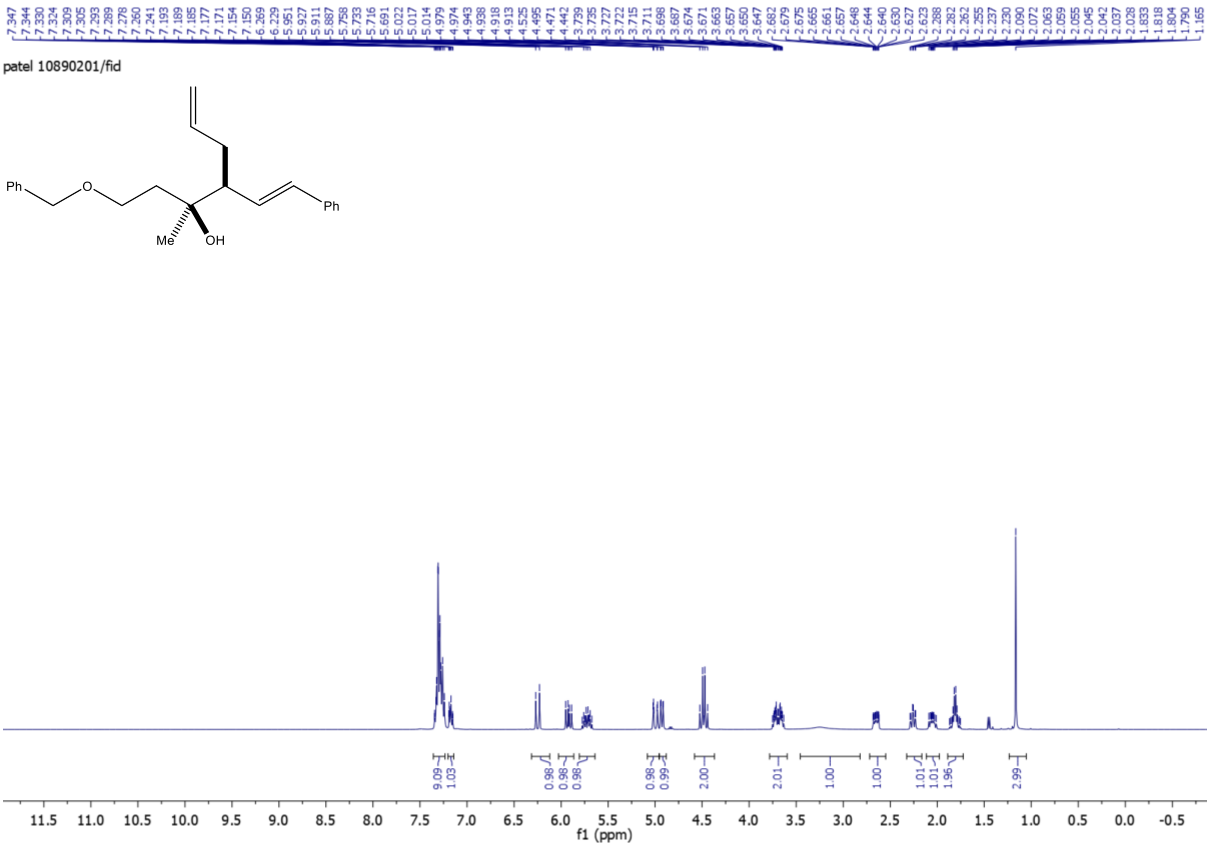
7.347 7.344 7.330 7.324 7.309 7.305 7.293 7.289 7.278 7.260 7.241 7.193 7.189 7.185 7.177 7.171 7.154 7.150 6.269 6.229 5.951 5.927 5.911 5.887 5.758 5.733 5.716 5.691 5.022 5.017 5.014 4.979 4.974 4.943 4.938 4.918 4.913 4.525 4.495 4.471 4.442 3.739 3.735 3.727 3.722 3.715 3.711 3.698 3.687 3.674 3.671 3.663 3.657 3.650 3.647 2.682 2.679 2.675 2.665 2.661 2.657 2.648 2.644 2.640 2.630 2.627 2.623 2.288 2.282 2.262 2.255 2.237 2.230 2.090 2.072 2.063 2.059 2.055 2.045 2.042 2.037 2.028 1.833 1.818 1.804 1.790 1.165
patel 10890201/fid
Ph
O
Me
OH
Ph
9.09
1.03
0.98
0.98
0.98
0.98
0.99
2.00
2.01
1.00
1.00
1.01
1.01
1.96
2.99
f1 (ppm)

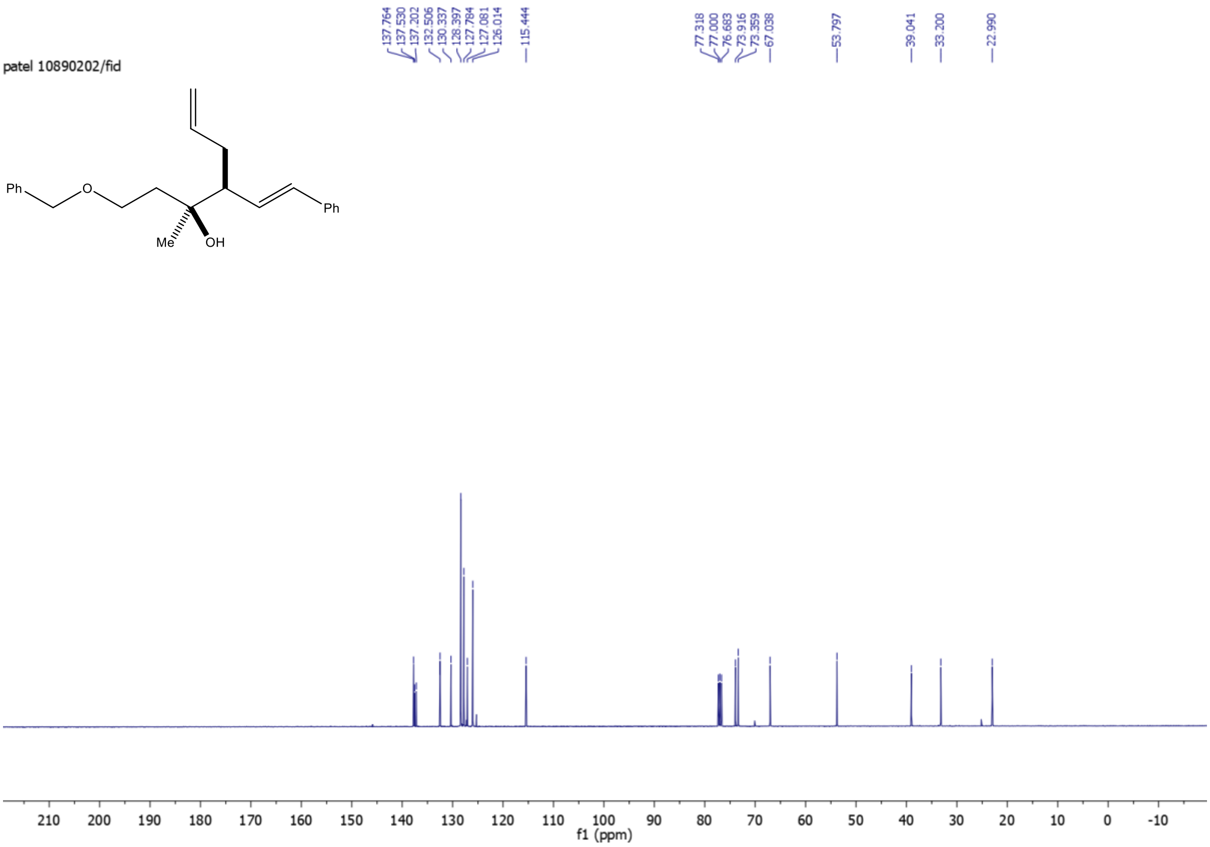
137.764 137.530 137.202 132.506 130.337 128.397 127.784 127.081 126.014 115.444
77.318 77.000 76.683 73.916 73.359 67.038 53.797 39.041 33.200 22.990
patel 10890202/fid
Ph
O
Me
OH
Ph
f1 (ppm)

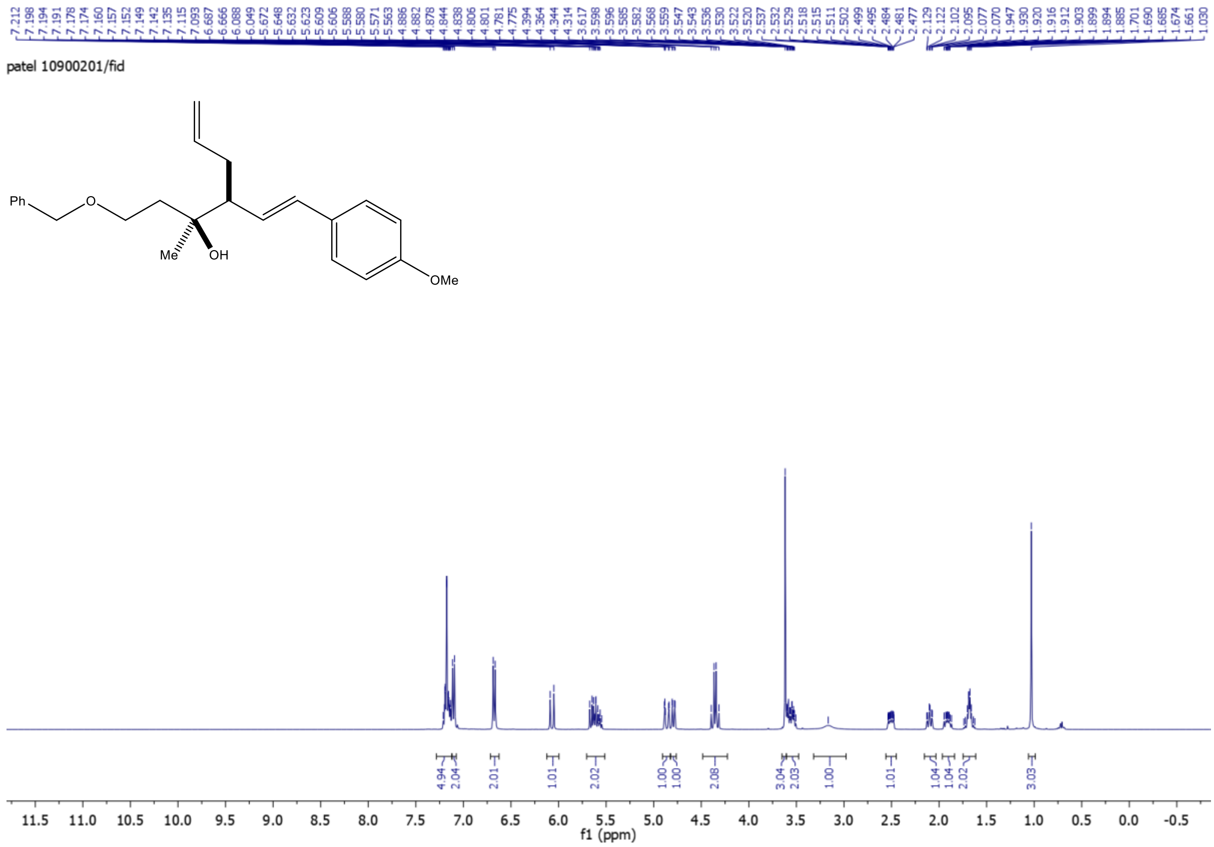
patel 10900201/fid
Ph
O
Me
OH
OMe
f1 (ppm)

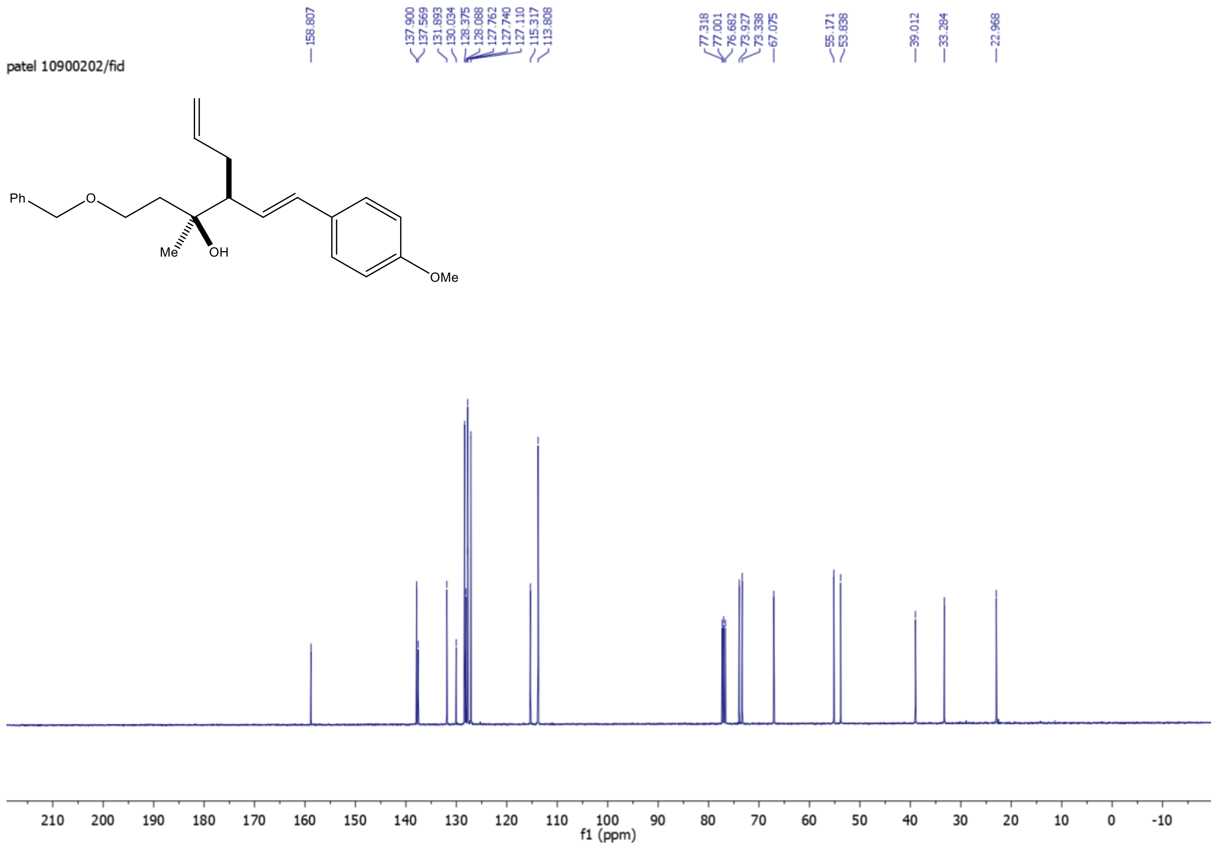
patel 10900202/fid
Ph
O
Me
OH
OMe
f1 (ppm)

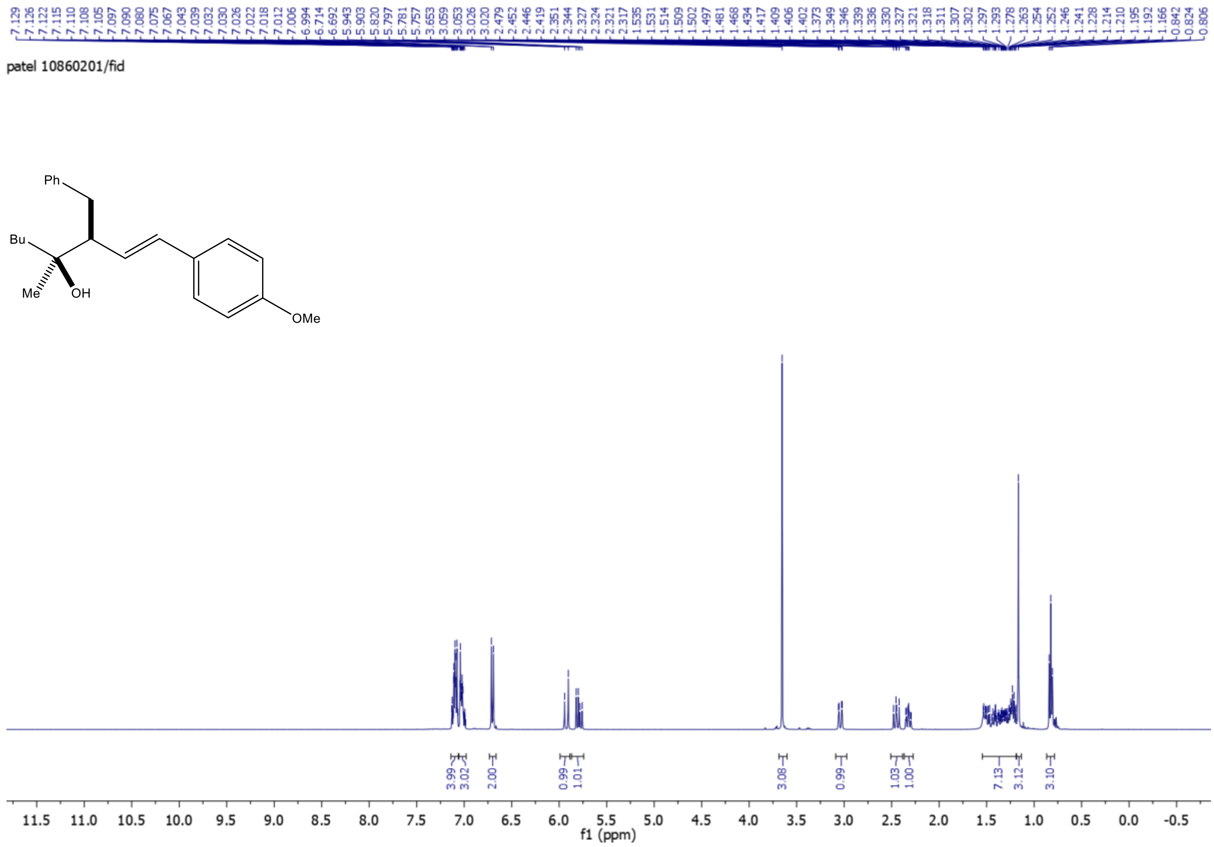
patel 10860201/fid
Ph
Bu
Me
OH
OMe
f1 (ppm)

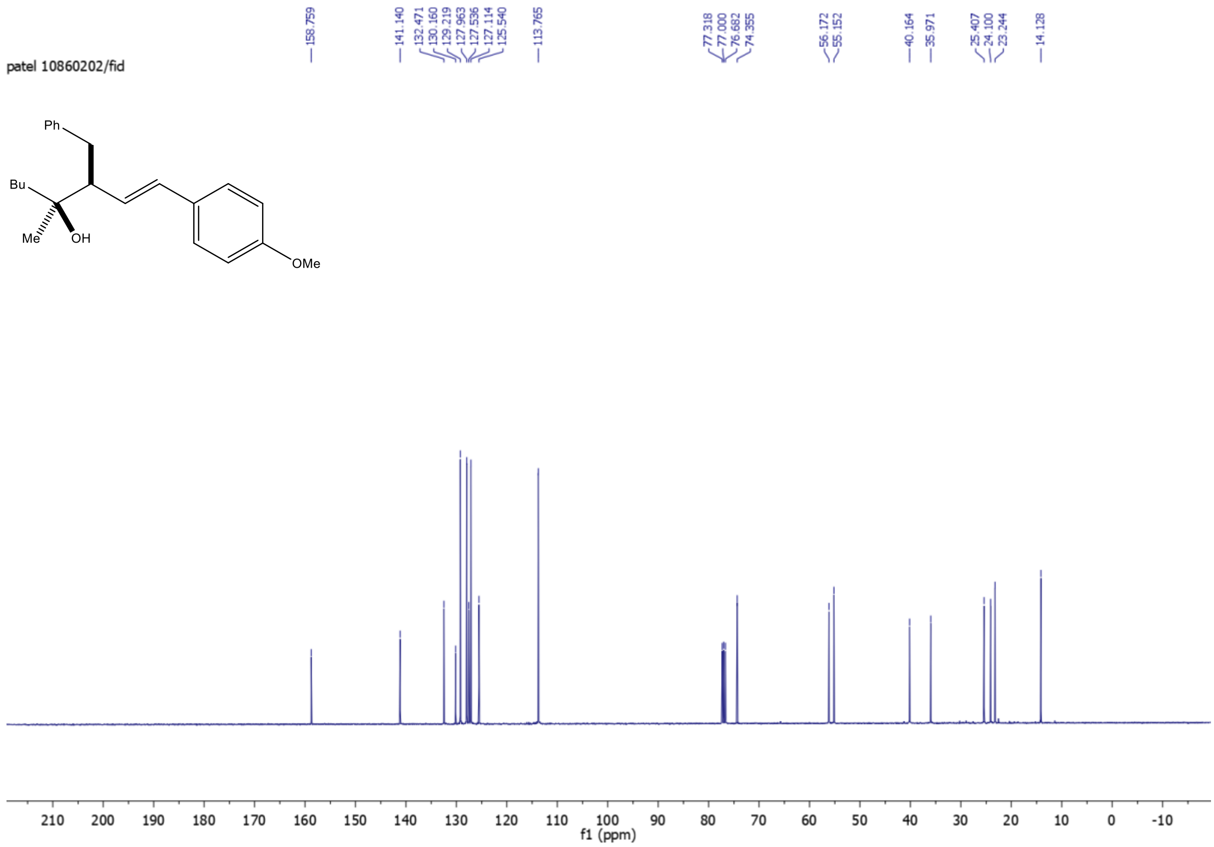
158.759
141.140
132.471
130.160
129.219
127.963
127.536
127.114
125.540
113.765
77.318
77.000
76.682
74.355
56.172
55.152
40.164
35.971
25.407
24.100
23.244
14.128
patel 10860202/fid
Ph
Bu
Me
OH
OMe
f1 (ppm)

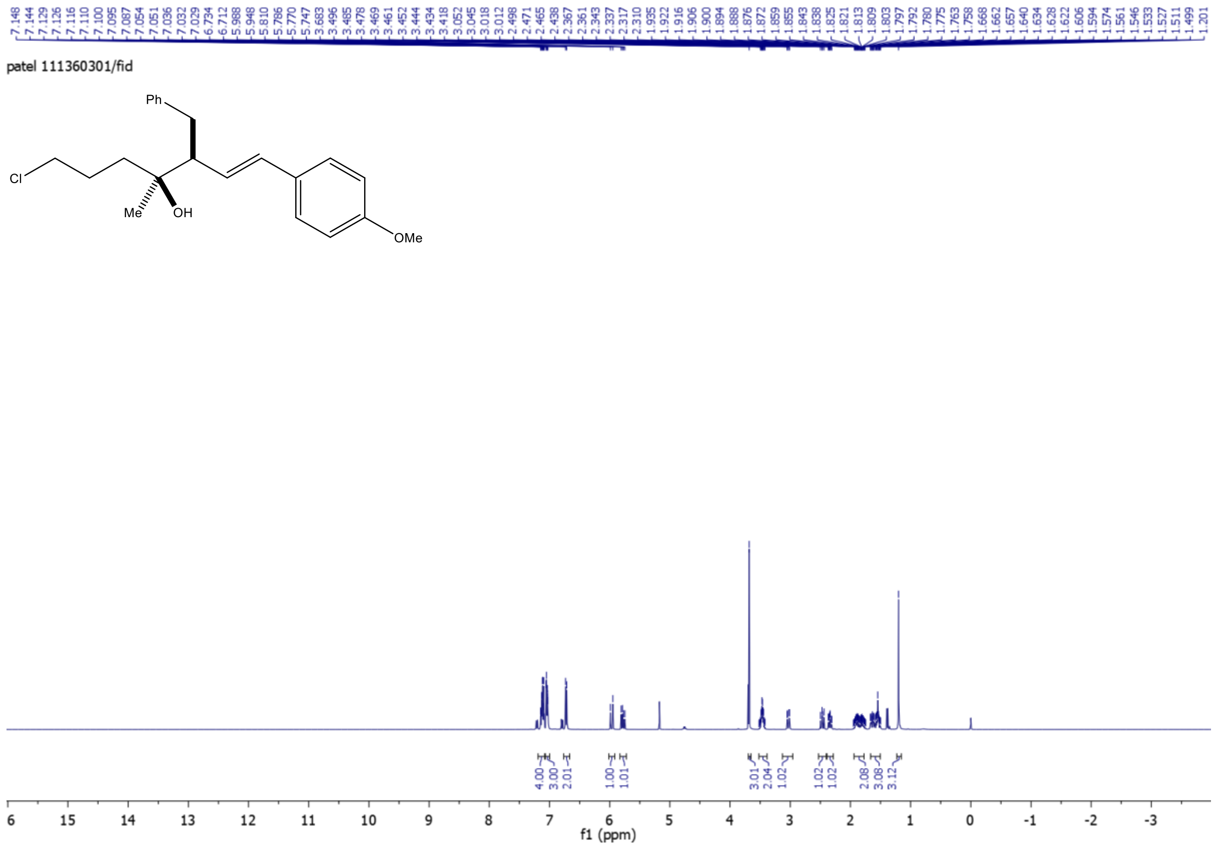
7.148 7.144 7.129 7.126 7.116 7.110 7.100 7.095 7.087 7.054 7.051 7.036 7.032 7.029 6.734 6.712 5.988 5.948 5.810 5.786 5.770 5.747 3.683 3.496 3.485 3.478 3.469 3.461 3.452 3.444 3.434 3.418 3.052 3.045 3.018 3.012 2.498 2.471 2.465 2.438 2.367 2.361 2.343 2.337 2.317 2.310 1.935 1.922 1.916 1.906 1.900 1.894 1.888 1.876 1.872 1.859 1.855 1.843 1.838 1.825 1.821 1.813 1.809 1.803 1.797 1.792 1.780 1.775 1.763 1.758 1.668 1.662 1.657 1.640 1.634 1.628 1.622 1.606 1.594 1.574 1.561 1.546 1.533 1.527 1.511 1.499 1.201
patel 111360301/fid
Ph
Cl
Me
OH
OMe
4.00 3.00 2.01 1.00 1.01 3.01 2.04 1.02 1.02 1.02 2.08 3.08 3.12
f1 (ppm)

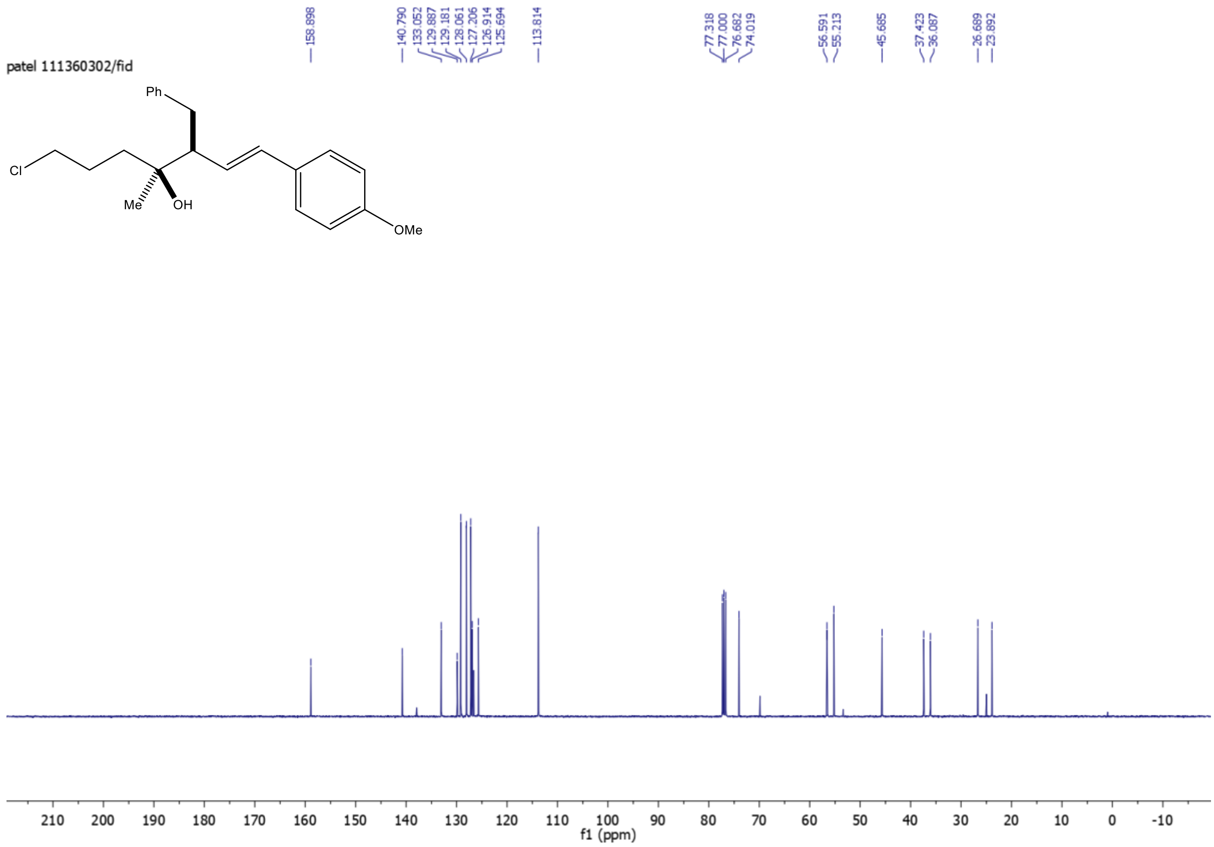
158.898 140.790 133.052 129.887 129.181 128.061 127.206 126.914 125.694 113.814 77.318 77.000 76.682 74.019 56.591 55.213 45.685 37.423 36.087 26.689 23.892
patel 111360302/fid
Ph
Cl
Me
OH
OMe
f1 (ppm)

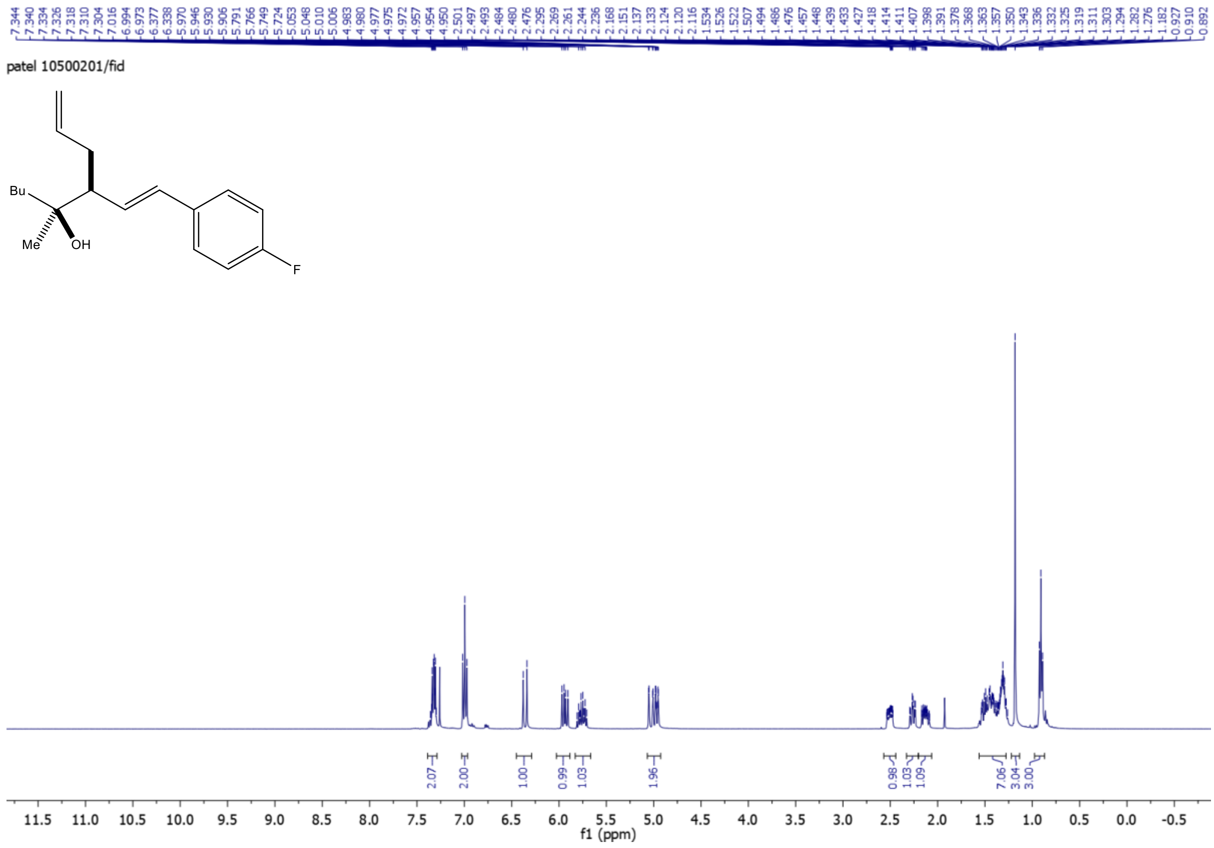
7.344 7.340 7.334 7.326 7.318 7.310 7.304 7.016 6.994 6.973 6.377 6.338 5.970 5.946 5.930 5.906 5.791 5.766 5.749 5.724 5.053 5.048 5.010 5.006 4.983 4.980 4.977 4.975 4.972 4.957 4.954 4.950 2.501 2.497 2.493 2.484 2.480 2.476 2.295 2.269 2.261 2.244 2.236 2.168 2.151 2.137 2.133 2.124 2.120 2.116 1.534 1.526 1.522 1.507 1.494 1.486 1.476 1.457 1.448 1.439 1.433 1.427 1.418 1.414 1.411 1.407 1.398 1.391 1.378 1.368 1.363 1.357 1.350 1.343 1.336 1.332 1.325 1.319 1.311 1.303 1.294 1.282 1.276 1.182 0.927 0.910 0.892
patel 10500201/fid
Bu
Me
OH
F
2.07 2.00 1.00 0.99 1.03 1.96 0.98 1.03 1.09 7.06 3.04 3.00
11.5 11.0 10.5 10.0 9.5 9.0 8.5 8.0 7.5 7.0 6.5 6.0 5.5 5.0 4.5 4.0 3.5 3.0 2.5 2.0 1.5 1.0 0.5 0.0 -0.5
f1 (ppm)

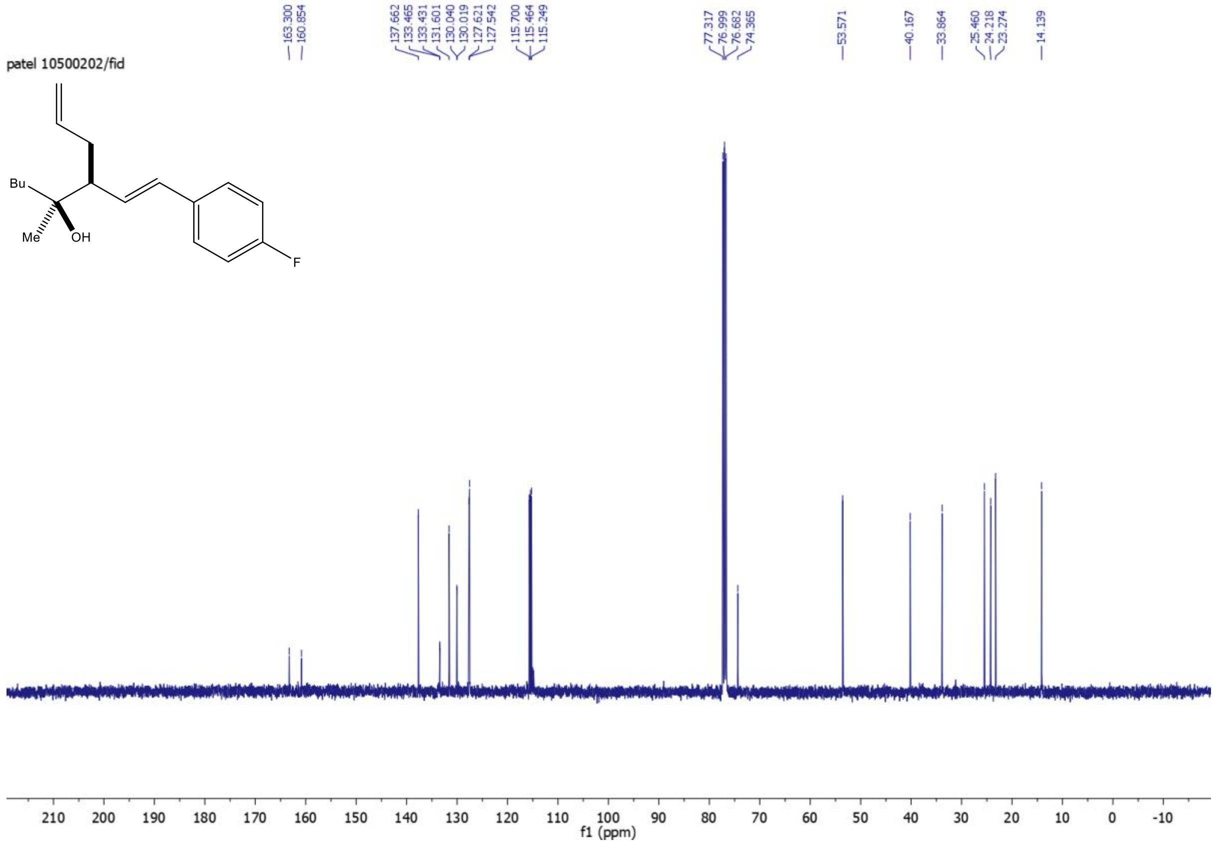
163.300 160.854 137.662 133.465 133.431 131.601 130.040 130.019 127.621 127.542 115.700 115.464 115.249 77.317 76.999 76.682 74.365 53.571 40.167 33.864 25.460 24.218 23.274 14.139
patel 10500202/fid
Bu
Me
OH
F
210 200 190 180 170 160 150 140 130 120 110 100 90 80 70 60 50 40 30 20 10 0 -10
f1 (ppm)

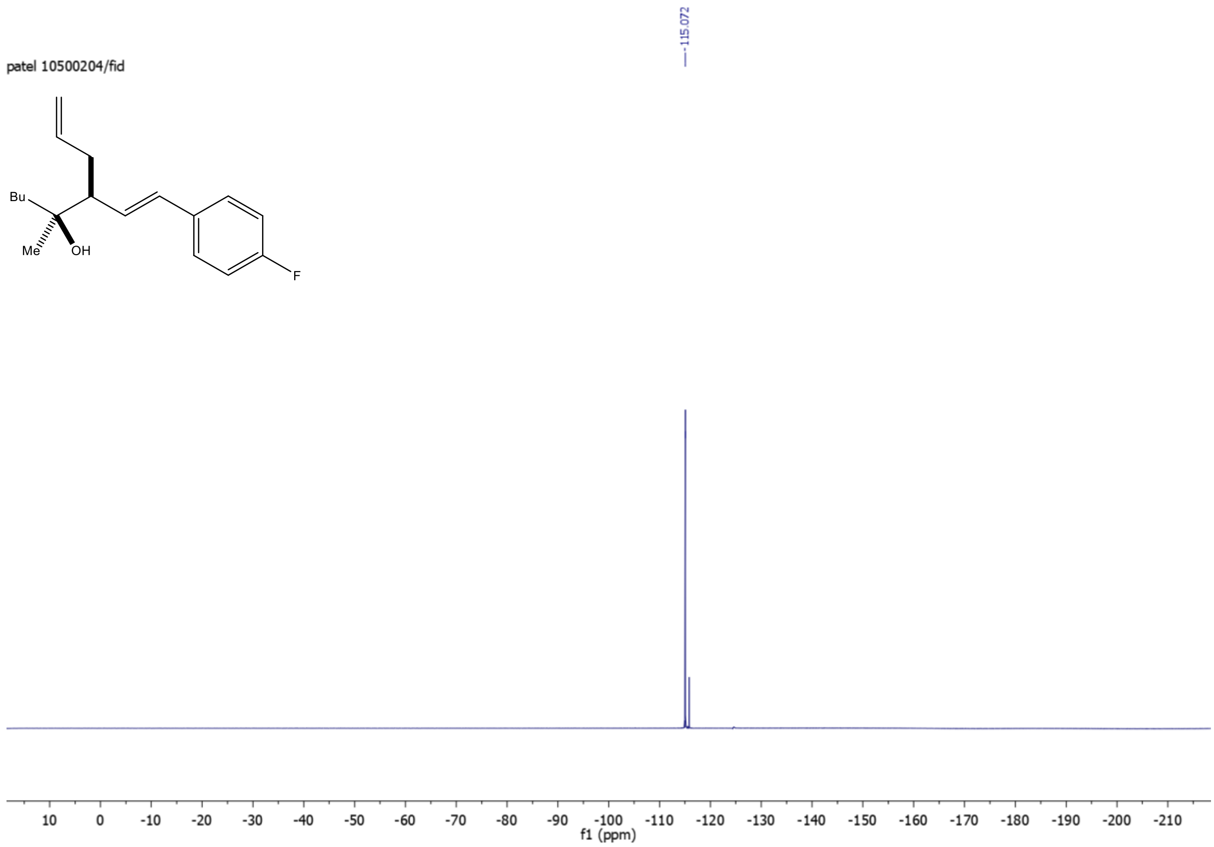
patel 10500204/fid
-115.072
Bu
Me
OH
F
f1 (ppm)

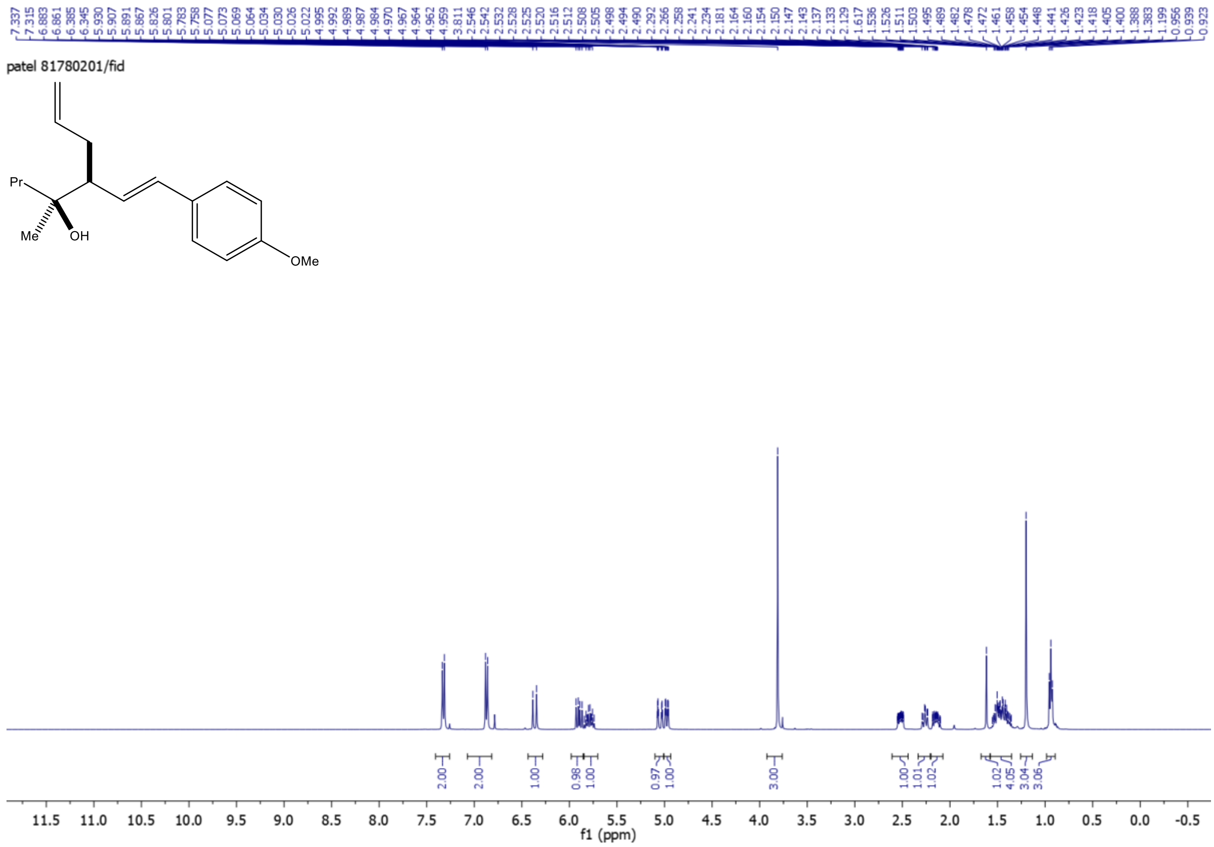
patel 81780201/fid
7.337
7.315
6.883
6.861
6.385
6.345
5.930
5.907
5.891
5.867
5.826
5.801
5.783
5.758
5.077
5.073
5.069
5.064
5.034
5.030
5.026
5.022
4.995
4.992
4.989
4.987
4.984
4.970
4.967
4.964
4.962
4.959
3.811
2.546
2.542
2.532
2.528
2.525
2.520
2.516
2.512
2.508
2.505
2.498
2.494
2.490
2.292
2.266
2.258
2.241
2.234
2.181
2.164
2.160
2.154
2.150
2.147
2.143
2.137
2.133
2.129
1.617
1.536
1.526
1.511
1.503
1.495
1.489
1.482
1.478
1.472
1.461
1.458
1.454
1.448
1.441
1.426
1.423
1.418
1.405
1.400
1.388
1.383
1.199
0.956
0.939
0.923
Pr
Me
OH
OMe
2.00
2.00
1.00
0.98
1.00
0.97
1.00
3.00
1.00
1.01
1.02
1.02
4.05
3.04
3.06
f1 (ppm)

patel 81780202/fid

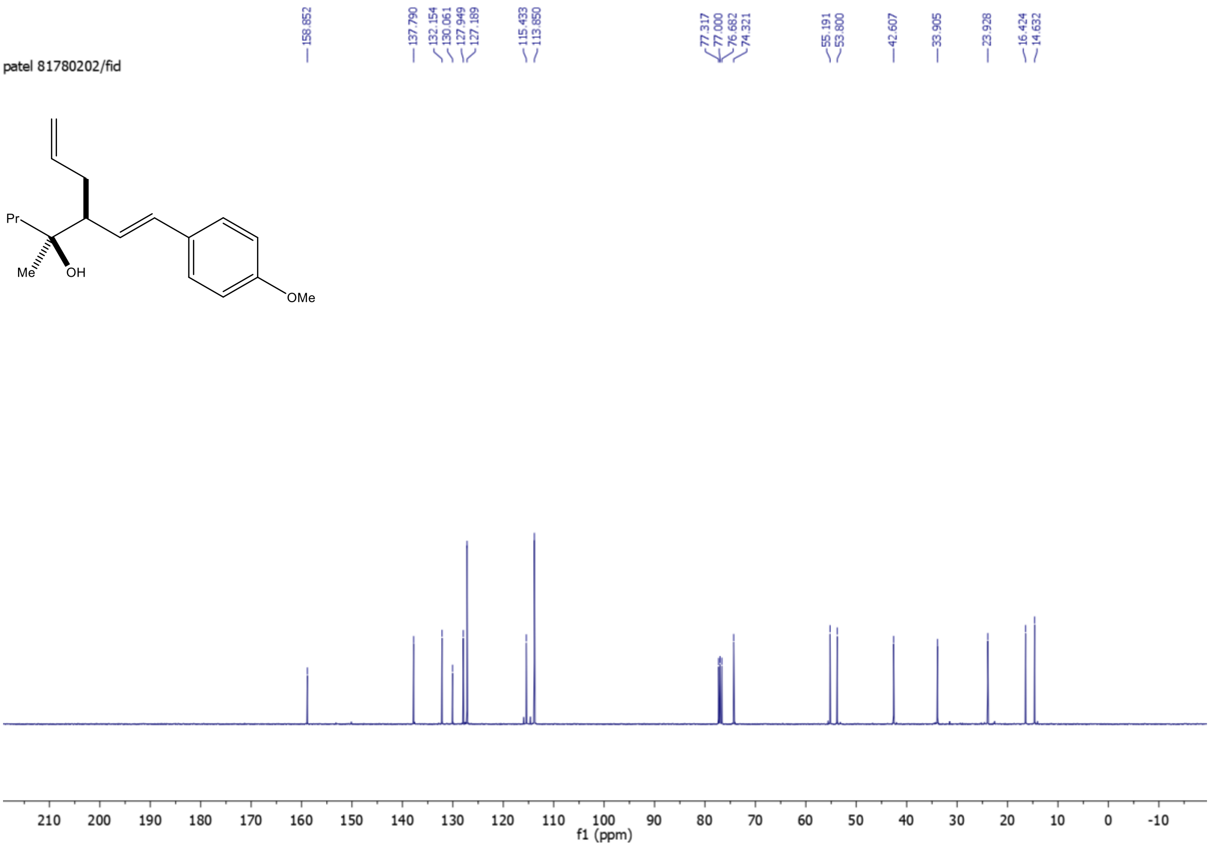

Leonie400-2023.101290101.fid

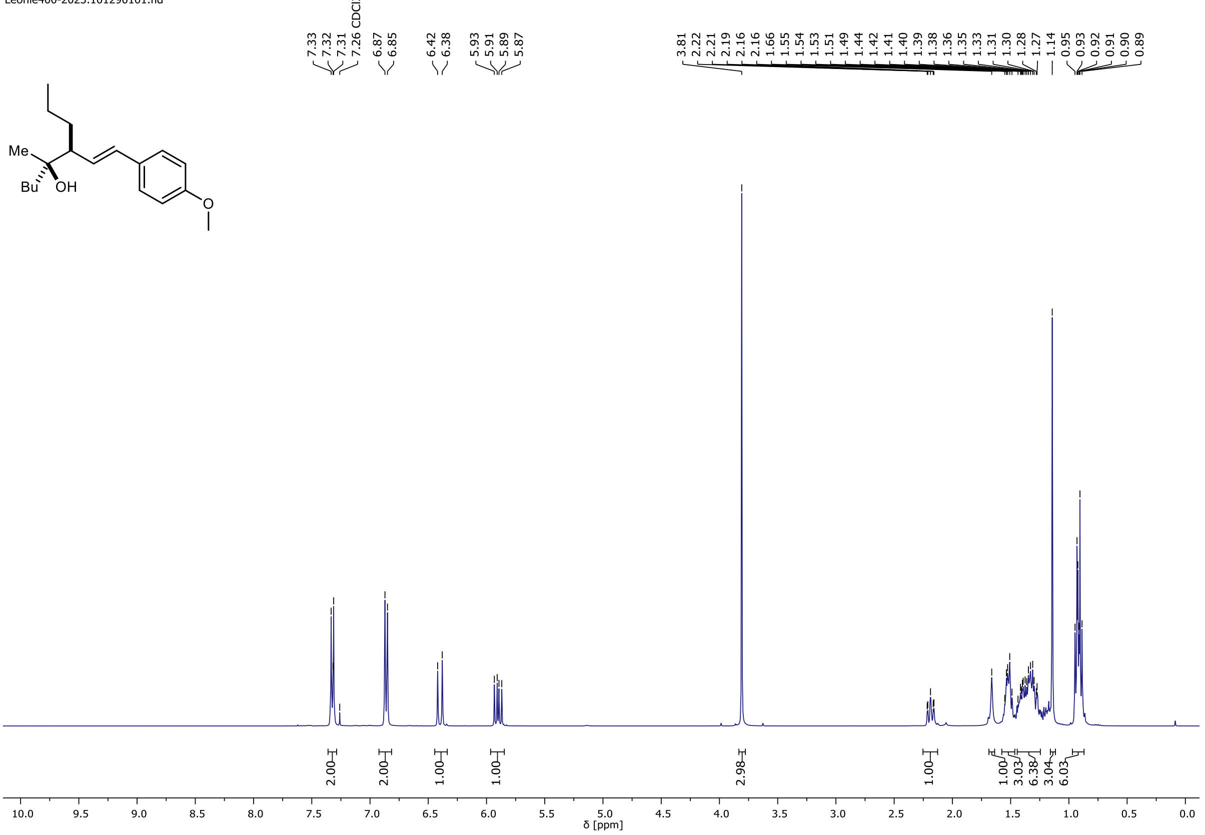

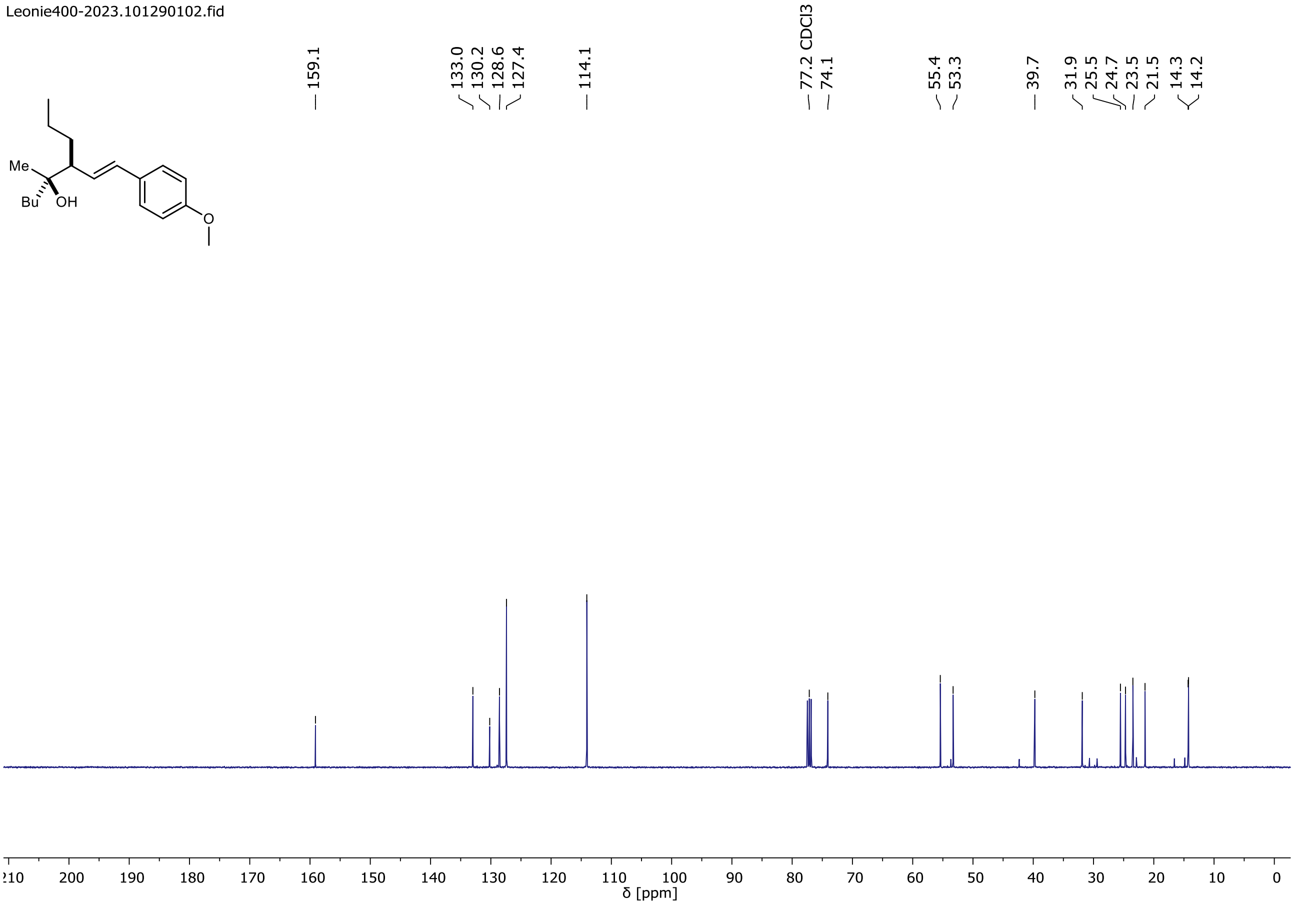
Leonie400-2023.101290102.fid
Me
Bu
OH
O
159.1
133.0
130.2
128.6
127.4
114.1
77.2 CDCl3
74.1
55.4
53.3
39.7
31.9
25.5
24.7
23.5
21.5
14.3
14.2
δ [ppm]

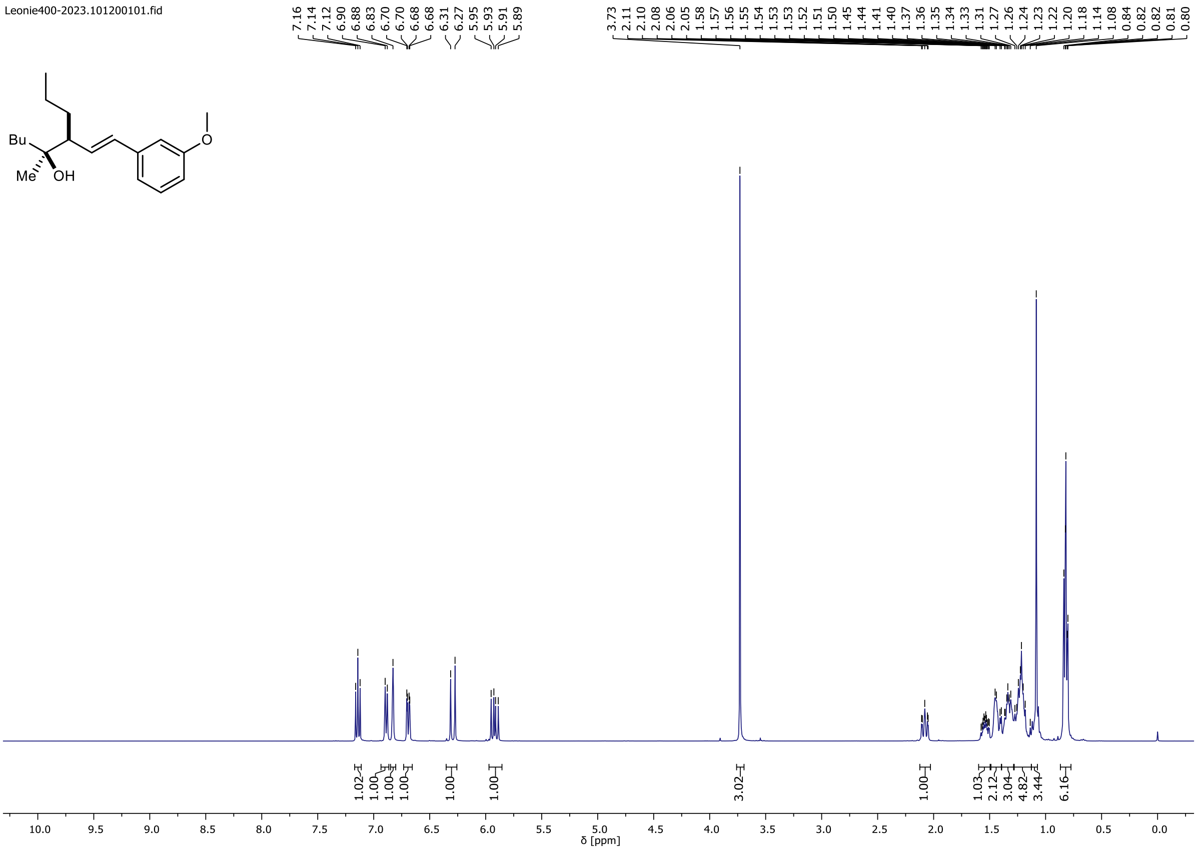
Leonie400-2023.101200101.fid
Bu
Me
OH
O
7.16 7.14 7.12 6.90 6.88 6.83 6.70 6.70 6.68 6.68 6.31 6.27 5.95 5.93 5.91 5.89
3.73 2.11 2.10 2.08 2.06 2.05 1.58 1.57 1.56 1.55 1.54 1.53 1.53 1.52 1.51 1.50 1.45 1.44 1.41 1.40 1.37 1.36 1.35 1.34 1.33 1.31 1.27 1.26 1.24 1.23 1.22 1.20 1.18 1.14 1.08 0.84 0.82 0.82 0.81 0.80
1.02 1.00 1.00 1.00 1.00 1.00 3.02 1.00 1.03 2.12 3.04 4.82 3.44 6.16
δ [ppm]

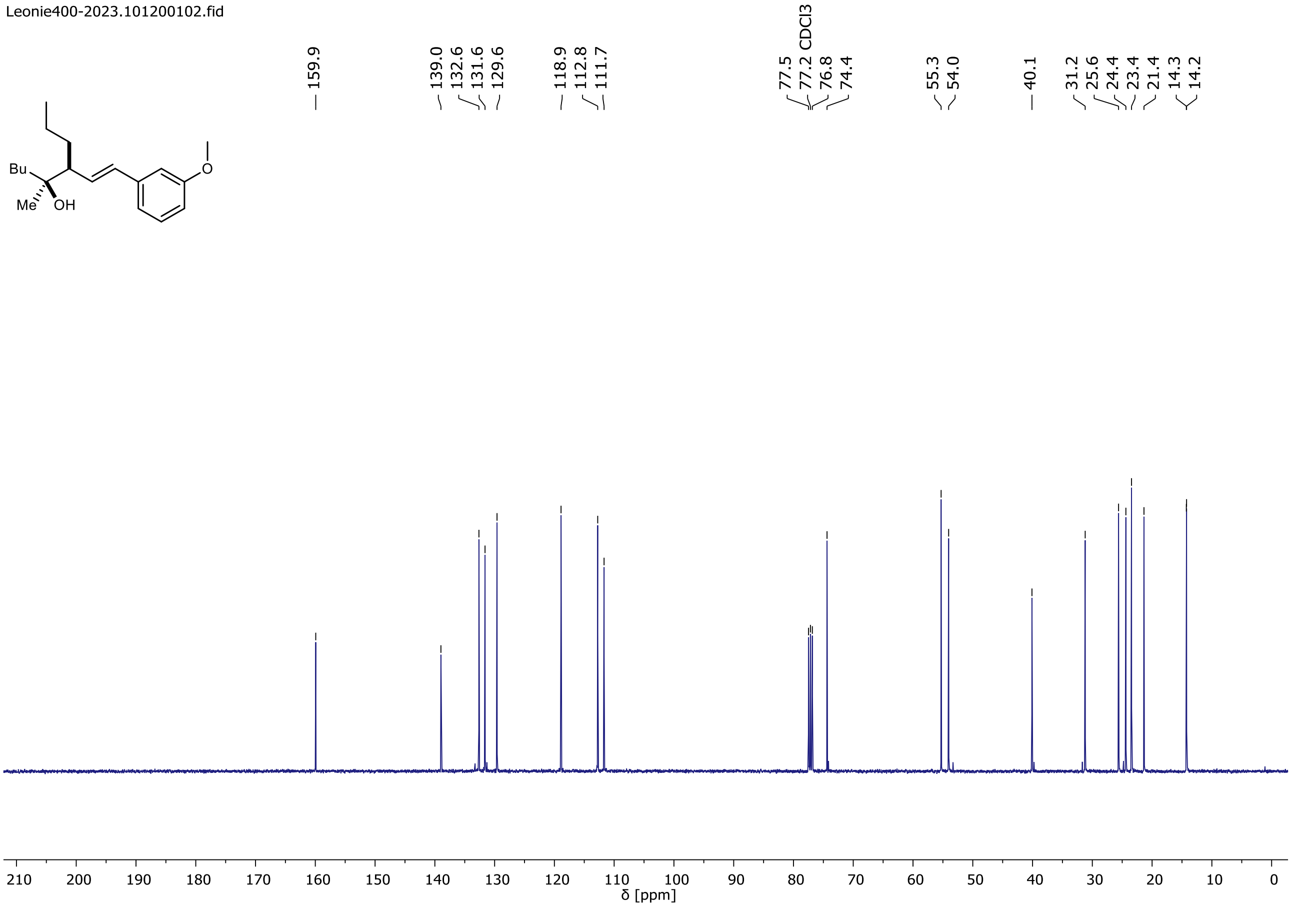
Leonie400-2023.101200102.fid
159.9
139.0
132.6
131.6
129.6
118.9
112.8
111.7
77.5
77.2 CDCl3
76.8
74.4
55.3
54.0
40.1
31.2
25.6
24.4
23.4
21.4
14.3
14.2
Bu
Me
OH
O
210 200 190 180 170 160 150 140 130 120 110 100 90 80 70 60 50 40 30 20 10 0
δ [ppm]

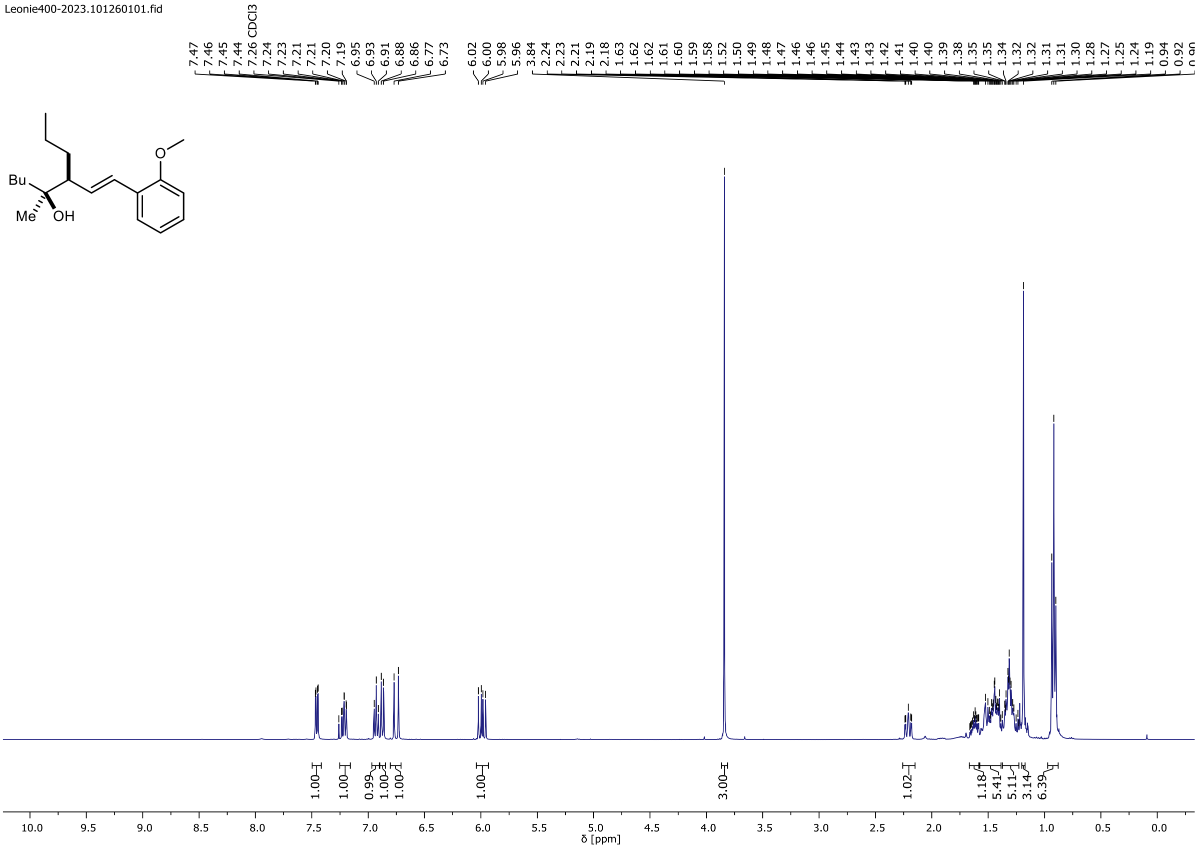
Leonie400-2023.101260101.fid
7.47 7.46 7.45 7.44 7.26 CDCl3 7.24 7.23 7.21 7.21 7.20 7.19 6.95 6.93 6.91 6.88 6.86 6.77 6.73
6.02 6.00 5.98 5.96 3.84 2.24 2.23 2.21 2.19 2.18 1.63 1.62 1.62 1.61 1.60 1.59 1.58 1.52 1.50 1.49 1.48 1.47 1.46 1.46 1.45 1.44 1.43 1.43 1.42 1.41 1.40 1.40 1.39 1.38 1.35 1.35 1.34 1.32 1.32 1.31 1.31 1.30 1.28 1.27 1.25 1.24 1.19 0.94 0.92
Bu
Me
OH
O
1.00
1.00
0.99
1.00
1.00
1.00
3.00
1.02
1.18
5.41
5.11
3.14
6.39
10.0 9.5 9.0 8.5 8.0 7.5 7.0 6.5 6.0 5.5 5.0 4.5 4.0 3.5 3.0 2.5 2.0 1.5 1.0 0.5 0.0
δ [ppm]

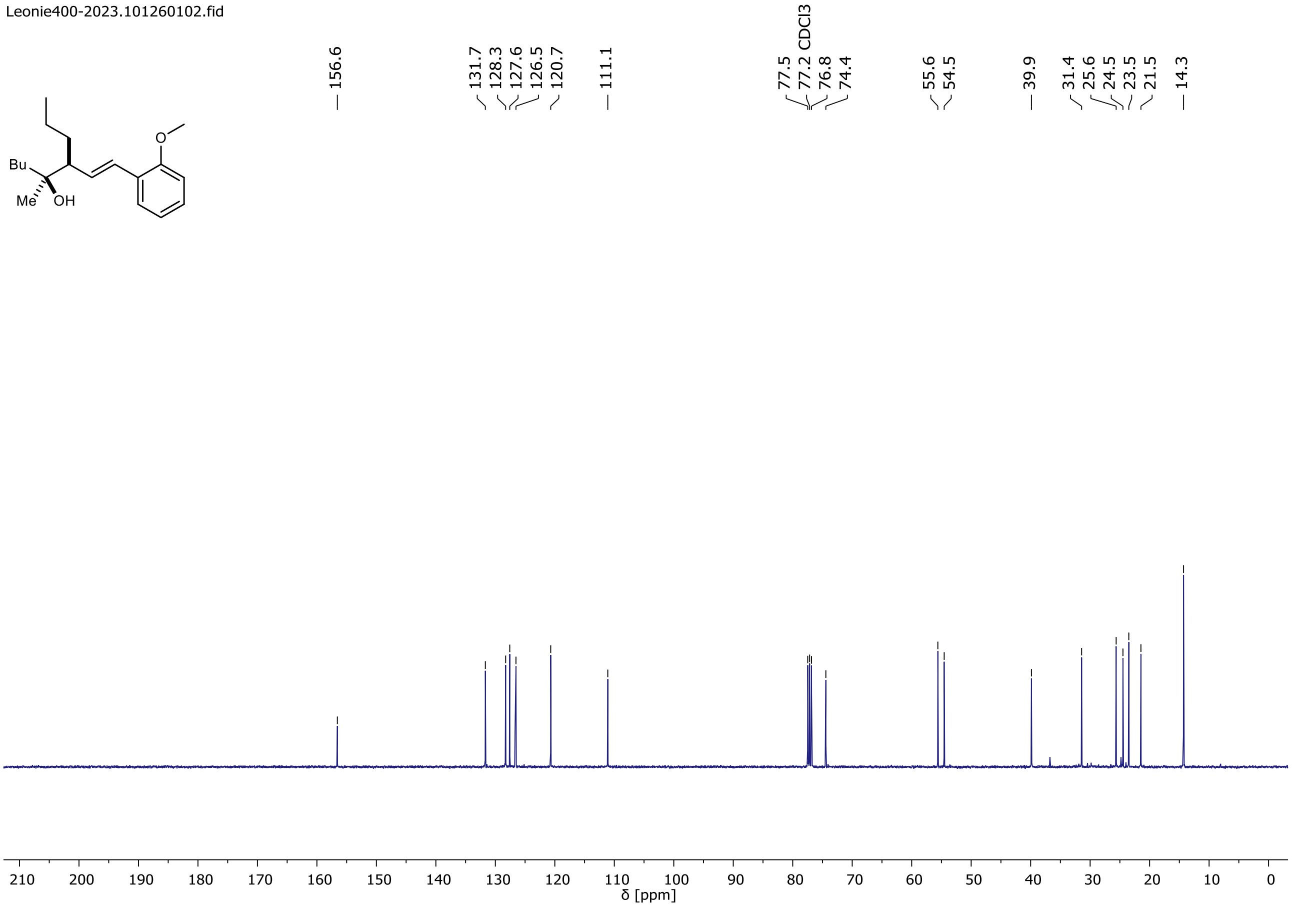
Leonie400-2023.101260102.fid
Bu
Me
OH
O
156.6
131.7
128.3
127.6
126.5
120.7
111.1
77.5
77.2 CDCl3
76.8
74.4
55.6
54.5
39.9
31.4
25.6
24.5
23.5
21.5
14.3
210 200 190 180 170 160 150 140 130 120 110 100 90 80 70 60 50 40 30 20 10 0
δ [ppm]

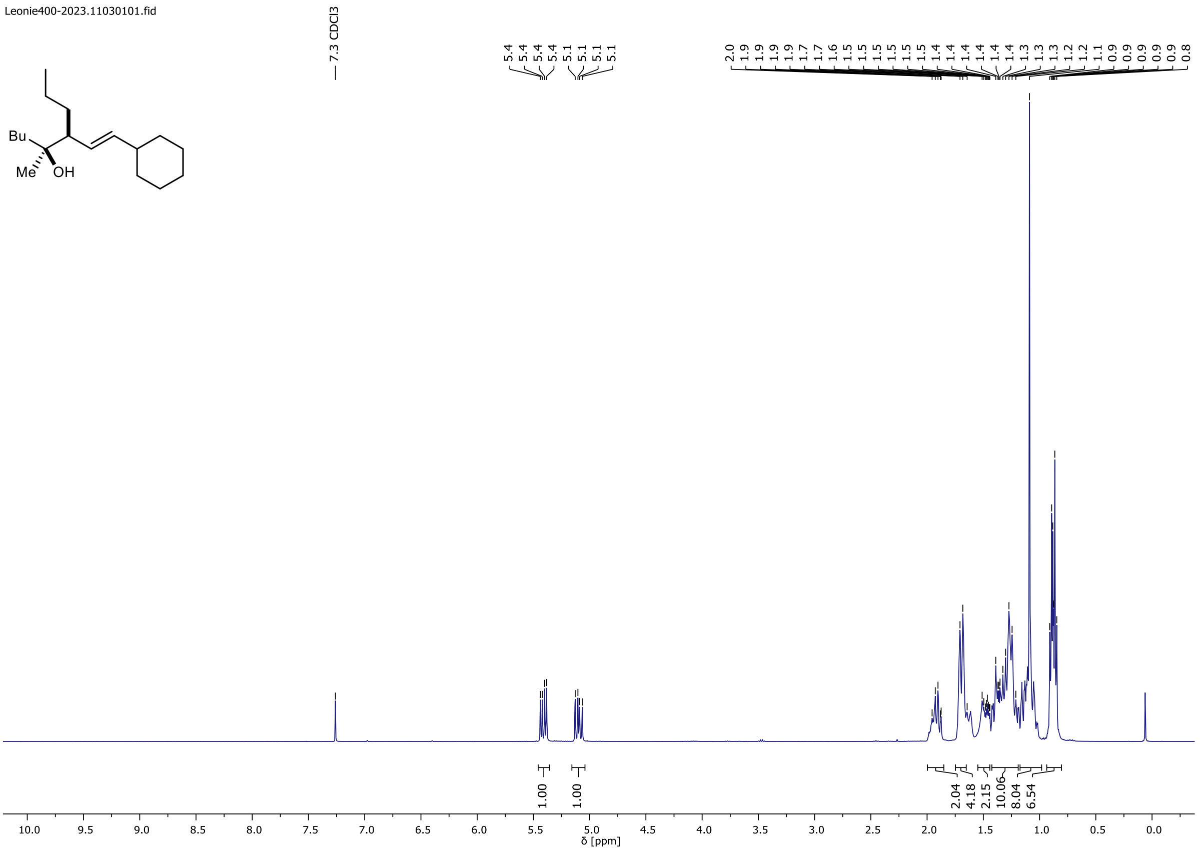
Leonie400-2023.11030101.fid
Bu
Me
OH
7.3 CDCl3
5.4 5.4 5.4 5.4 5.1 5.1 5.1 5.1
2.0 1.9 1.9 1.9 1.7 1.7 1.6 1.5 1.5 1.5 1.5 1.5 1.4 1.4 1.4 1.4 1.3 1.3 1.3 1.2 1.1 0.9 0.9 0.9 0.9 0.9 0.8
1.00
1.00
2.04
4.18
2.15
10.06
8.04
6.54
10.0 9.5 9.0 8.5 8.0 7.5 7.0 6.5 6.0 5.5 5.0 4.5 4.0 3.5 3.0 2.5 2.0 1.5 1.0 0.5 0.0
δ [ppm]

Leonie400-2023.11030102.fid

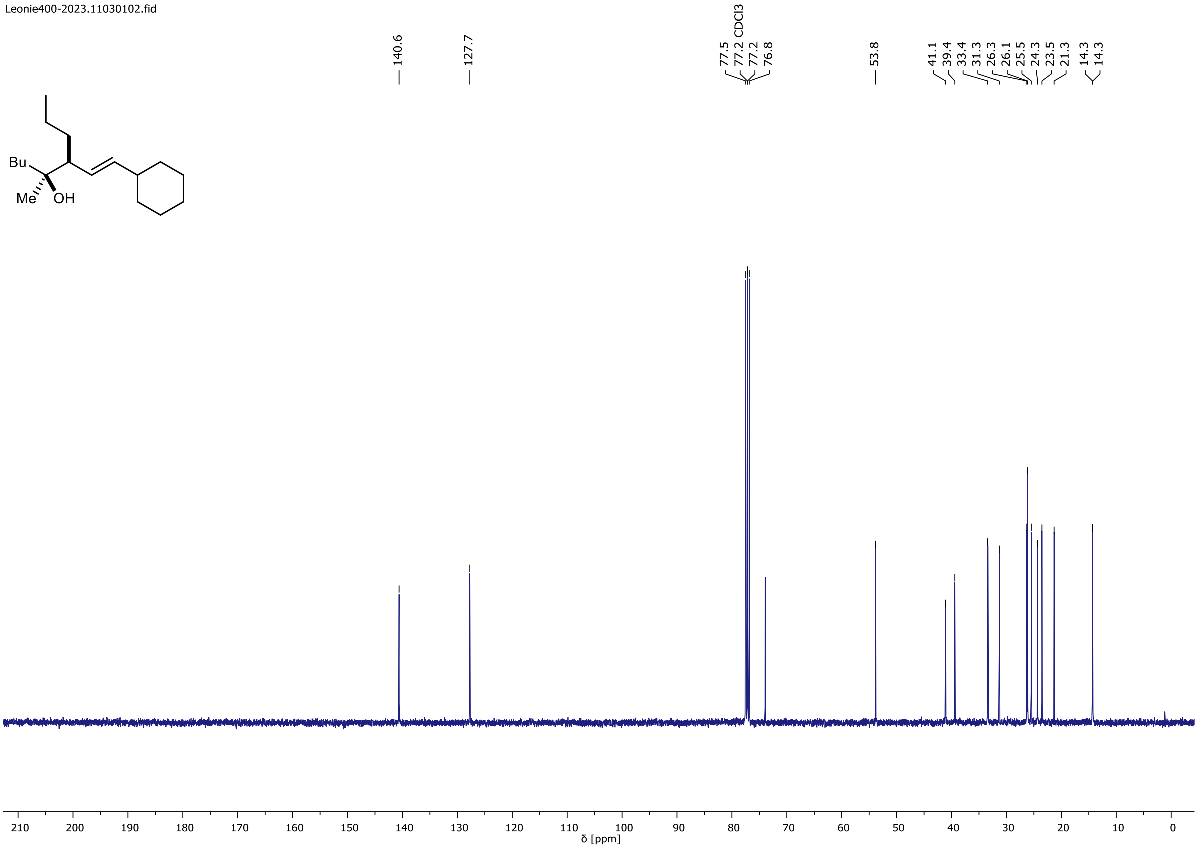

Leonie400-2023.101190101.fid

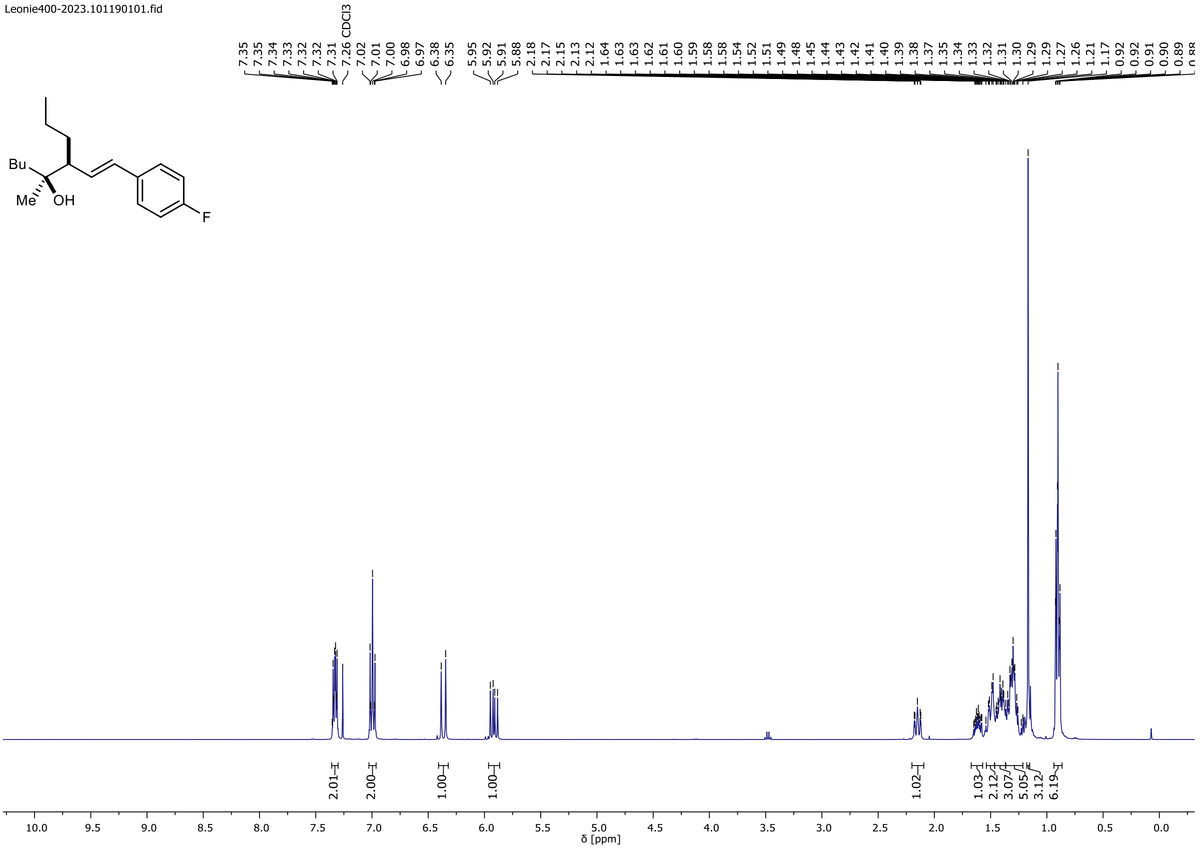

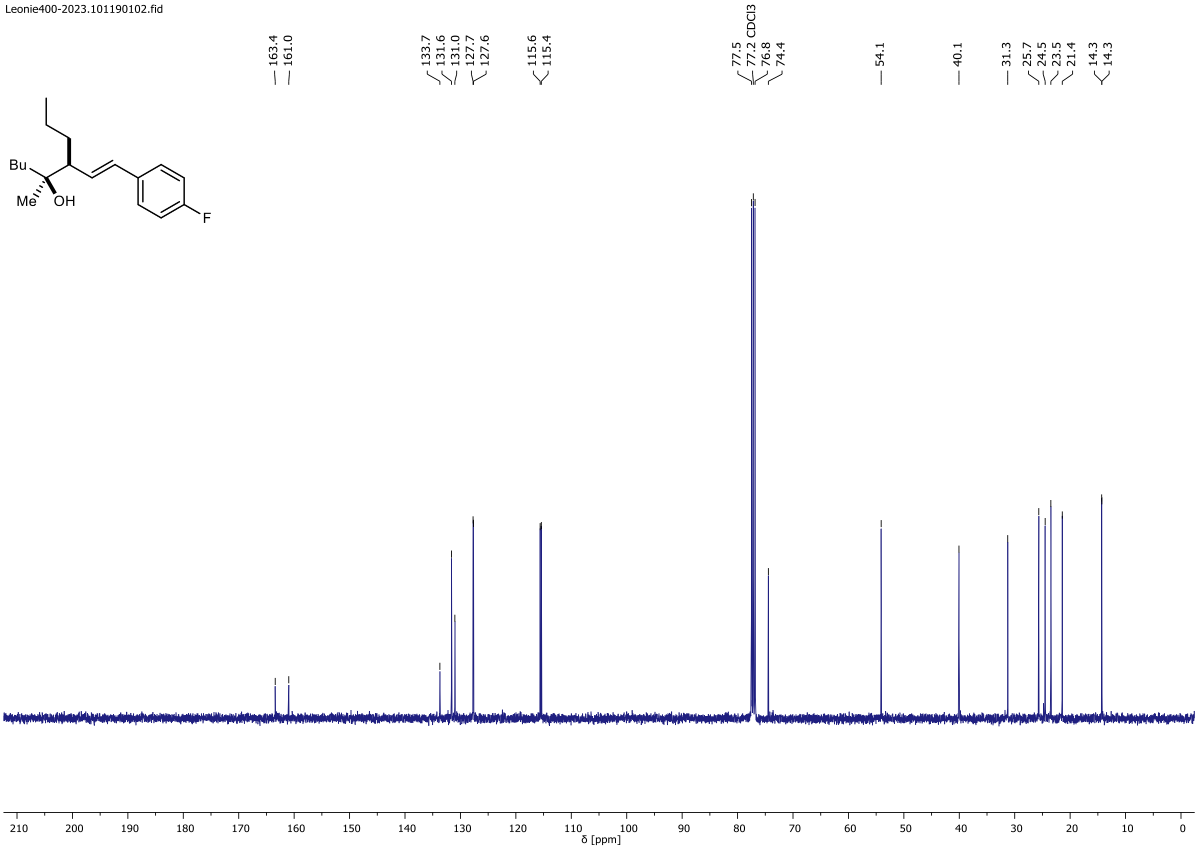
Leonie400-2023.101190102.fid
163.4
161.0
133.7
131.6
131.0
127.7
127.6
115.6
115.4
77.5
77.2 CDCl3
76.8
74.4
54.1
40.1
31.3
25.7
24.5
23.5
21.4
14.3
14.3
Bu
Me
OH
F
δ [ppm]

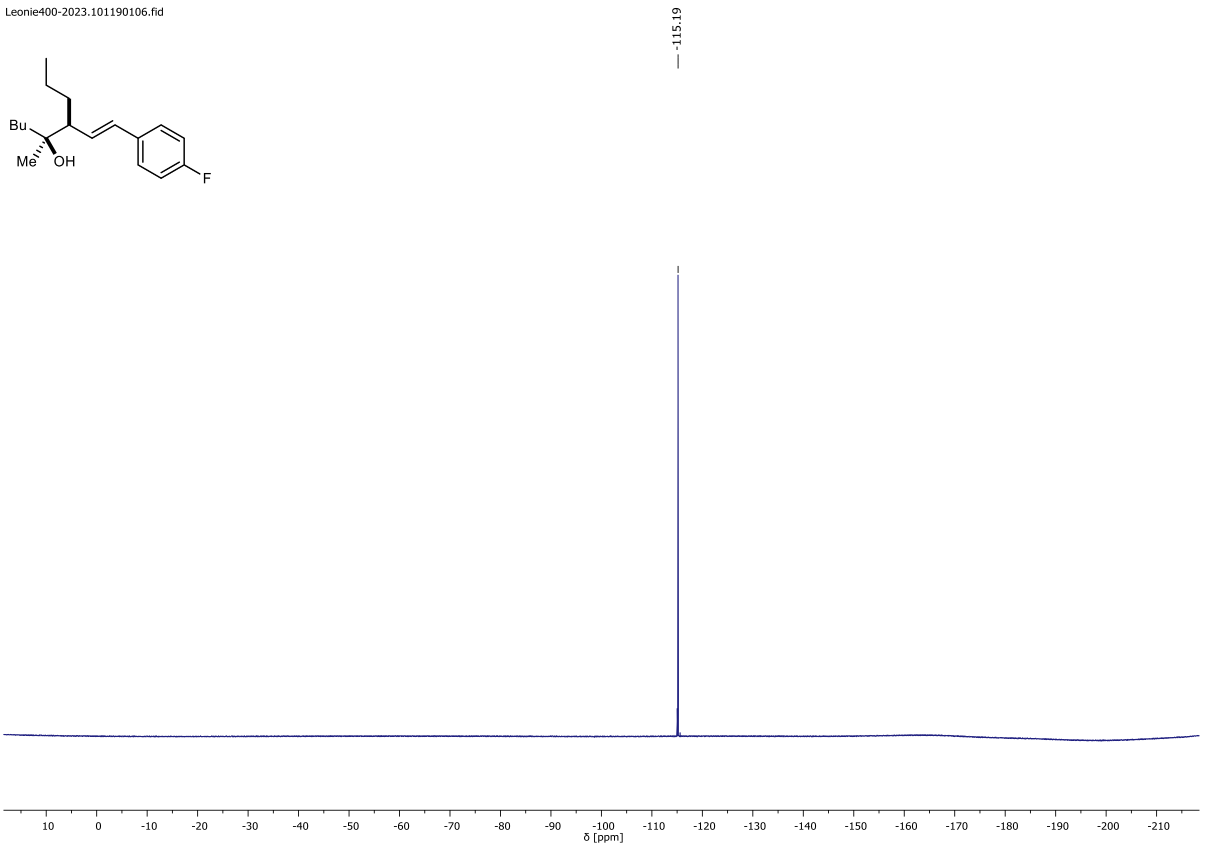
Leonie400-2023.101190106.fid
-115.19
Bu
Me
OH
F
δ [ppm]

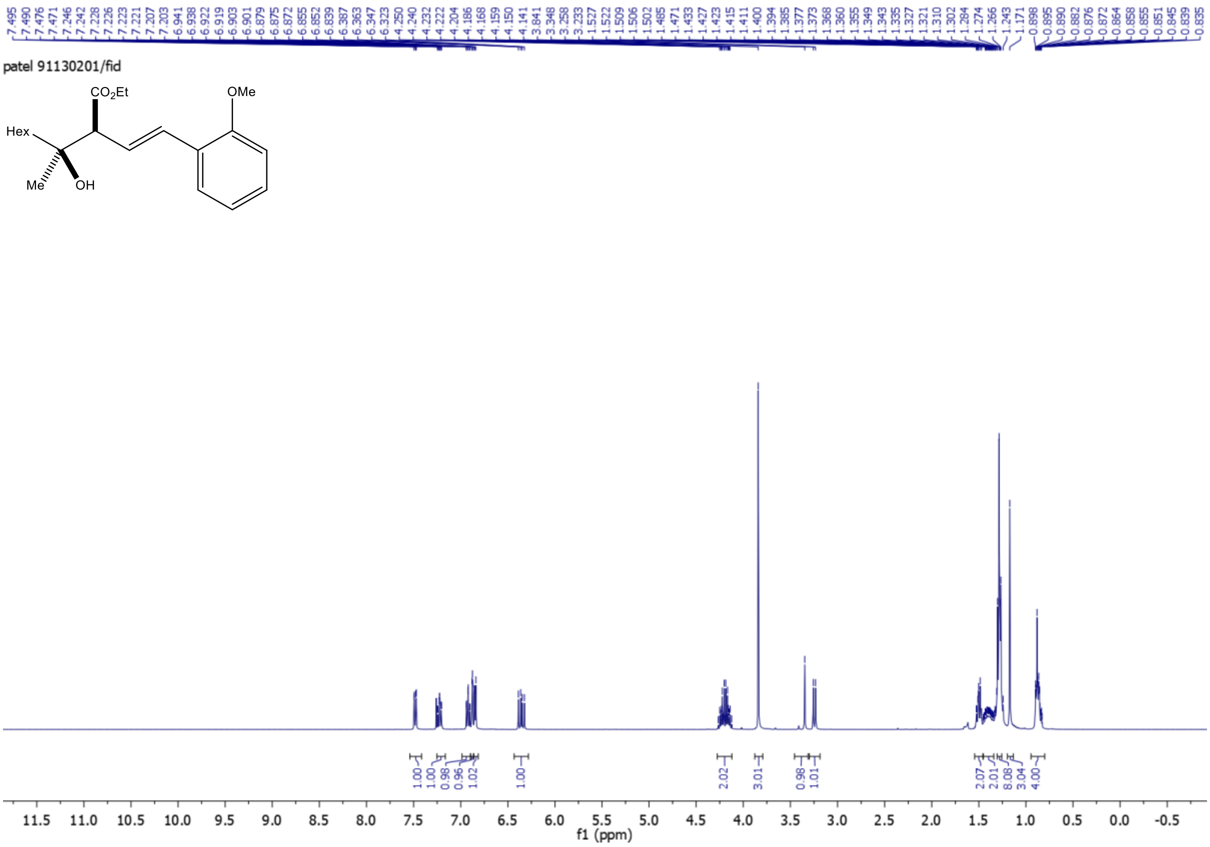

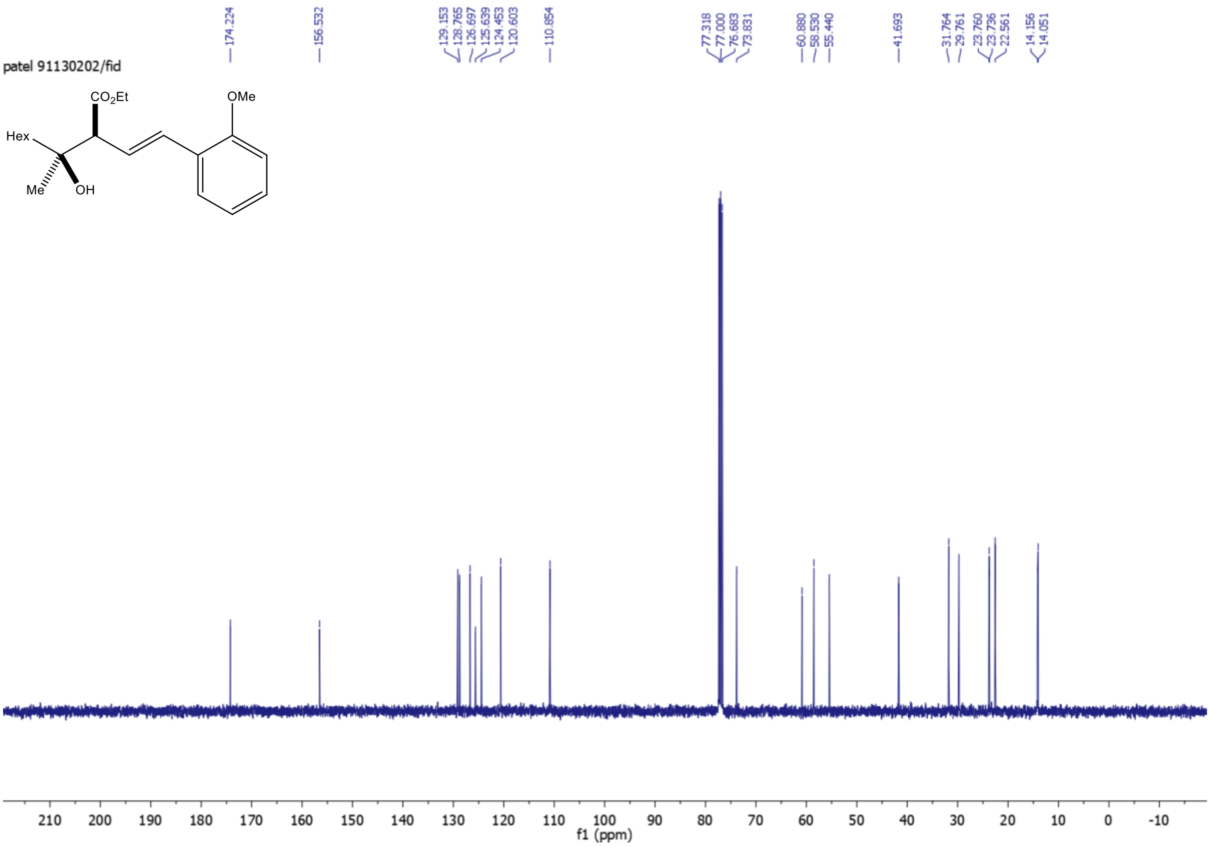

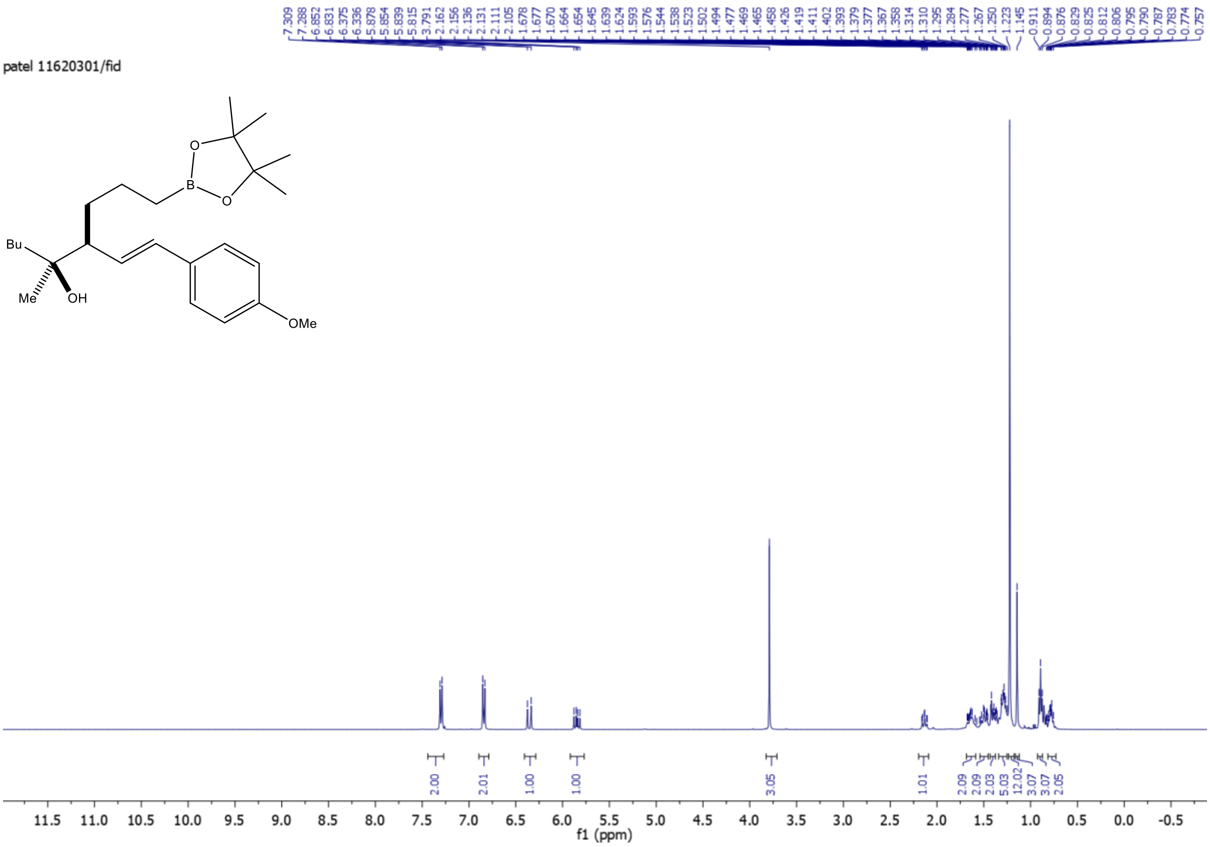
patel 11620301/fid
7.309
7.288
6.852
6.831
6.375
6.336
5.878
5.854
5.839
5.815
3.791
2.162
2.156
2.136
2.131
2.111
2.105
1.678
1.677
1.670
1.664
1.654
1.645
1.639
1.624
1.593
1.576
1.544
1.538
1.523
1.502
1.494
1.477
1.469
1.465
1.458
1.426
1.419
1.411
1.402
1.393
1.379
1.377
1.367
1.358
1.314
1.310
1.295
1.284
1.277
1.267
1.250
1.223
1.145
0.911
0.894
0.876
0.829
0.825
0.812
0.806
0.795
0.790
0.787
0.783
0.774
0.757
Bu
Me
OH
O
B
O
OMe
2.00
2.01
1.00
1.00
3.05
1.01
2.09
2.09
2.03
5.03
12.02
3.07
3.07
2.05
f1 (ppm)

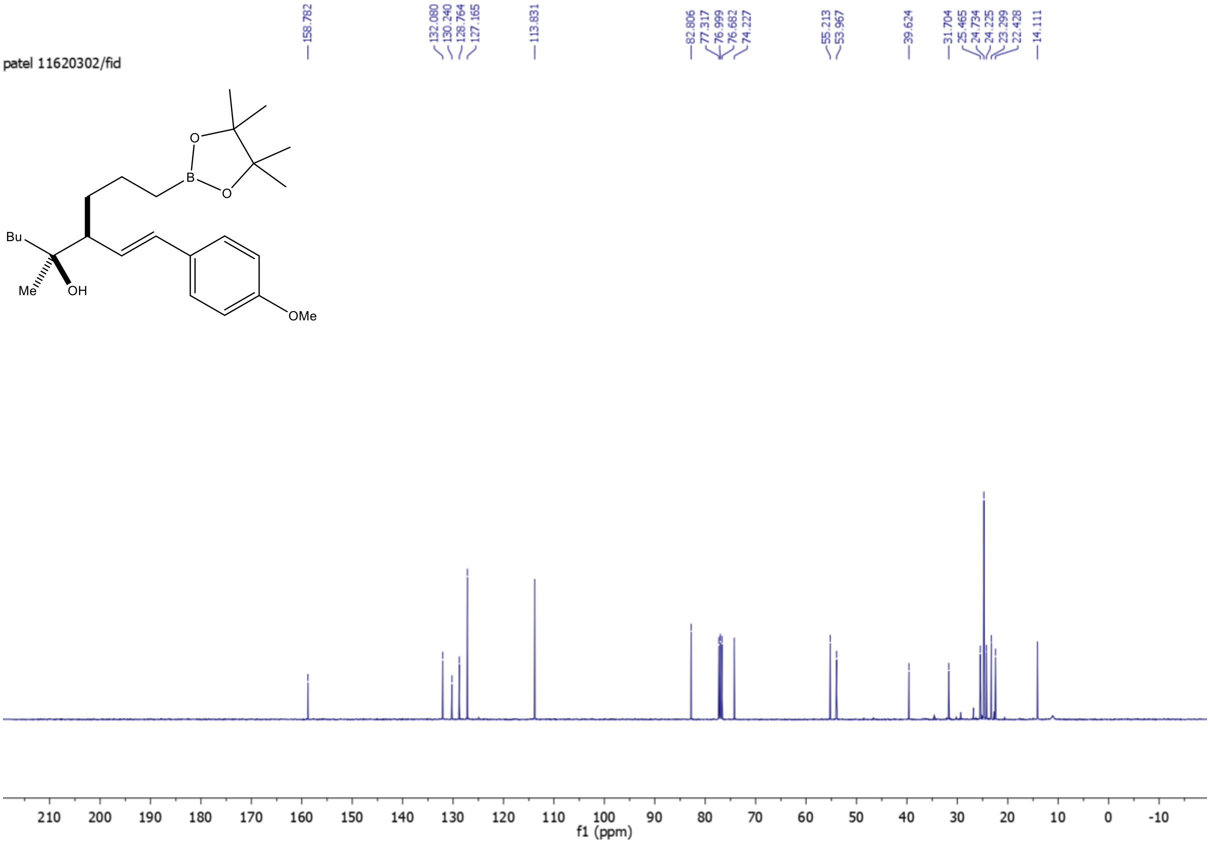
patel 11620302/fid
158.782
132.080
130.240
128.764
127.165
113.831
82.806
77.317
76.999
76.682
74.227
55.213
53.967
39.624
31.704
25.465
24.734
24.225
23.299
22.428
14.111
Bu
Me
OH
O
B
O
OMe
f1 (ppm)

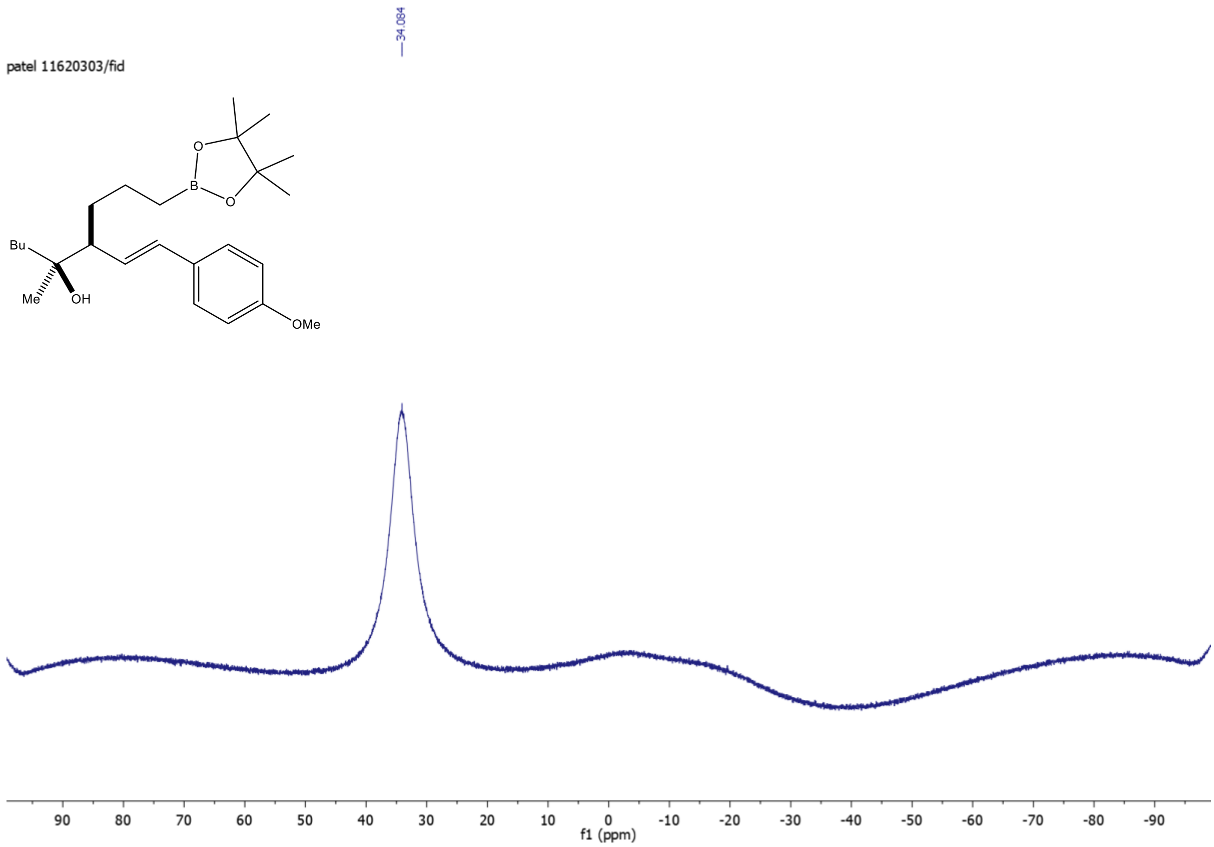
34.084
patel 11620303/fid
O
B
O
Bu
Me
OH
OMe
90 80 70 60 50 40 30 20 10 0 -10 -20 -30 -40 -50 -60 -70 -80 -90
f1 (ppm)

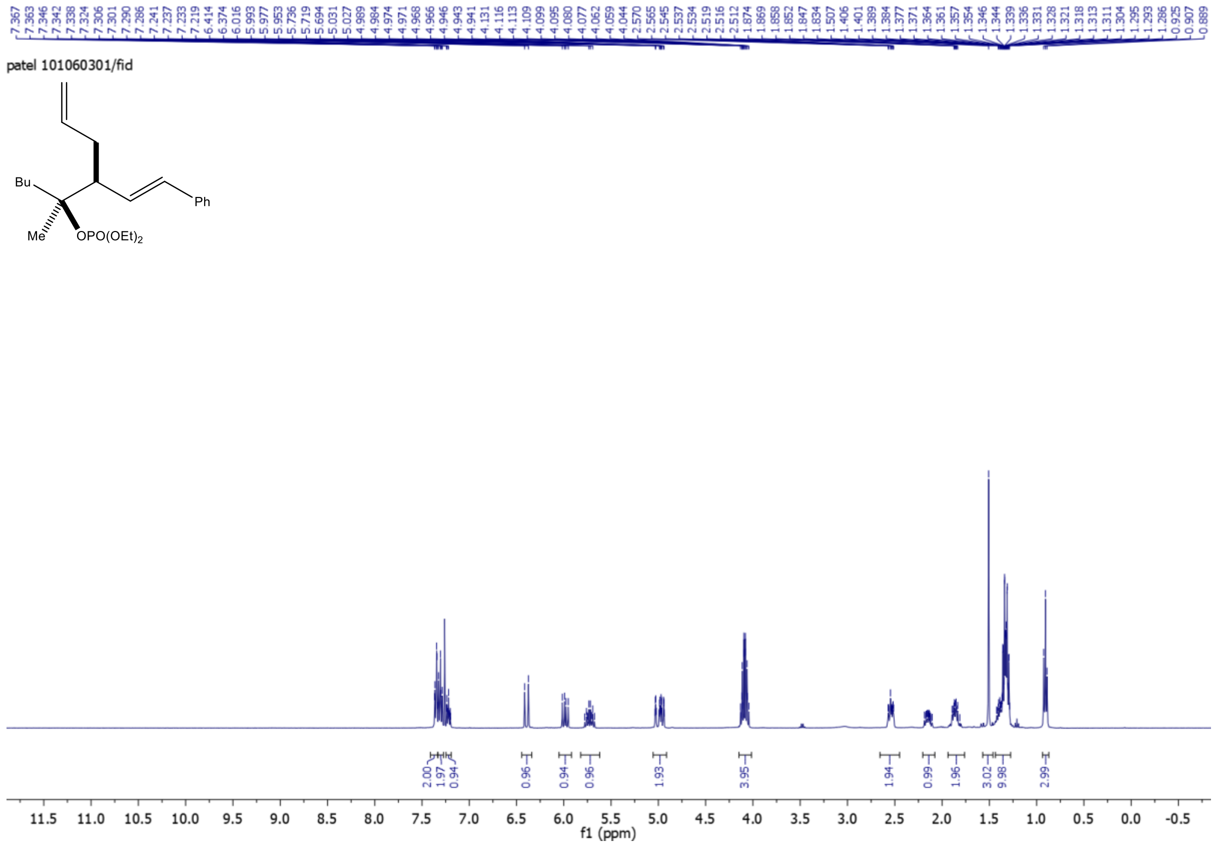
7.367 7.363 7.346 7.342 7.338 7.324 7.306 7.301 7.290 7.286 7.241 7.237 7.233 7.219 6.414 6.374 6.016 5.993 5.977 5.953 5.736 5.719 5.694 5.031 5.027 4.989 4.984 4.974 4.971 4.968 4.966 4.946 4.943 4.941 4.131 4.116 4.113 4.109 4.099 4.095 4.080 4.077 4.062 4.059 4.044 2.570 2.565 2.545 2.537 2.534 2.519 2.516 2.512 1.874 1.869 1.858 1.852 1.847 1.834 1.507 1.406 1.401 1.389 1.384 1.377 1.371 1.364 1.361 1.357 1.354 1.346 1.344 1.339 1.336 1.331 1.328 1.321 1.318 1.313 1.311 1.304 1.295 1.293 1.286 0.925 0.907 0.889
patel 101060301/fid
Bu
Ph
Me
OPO(OEt)2
2.00 1.97 0.94 0.96 0.94 0.96 1.93 3.95 1.94 0.99 1.96 3.02 9.98 2.99
11.5 11.0 10.5 10.0 9.5 9.0 8.5 8.0 7.5 7.0 6.5 6.0 5.5 5.0 4.5 4.0 3.5 3.0 2.5 2.0 1.5 1.0 0.5 0.0 -0.5
f1 (ppm)

patel 101060302/fid

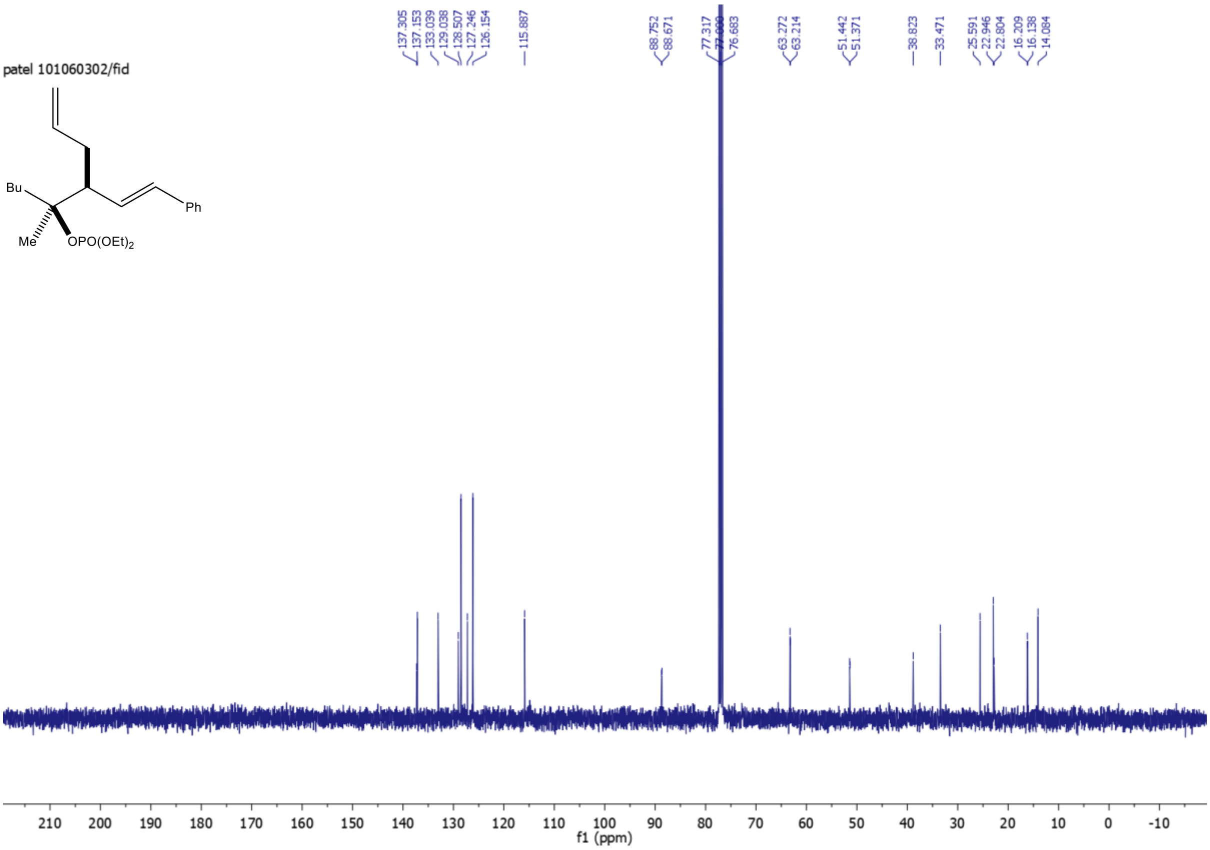

patel 101060303/fid

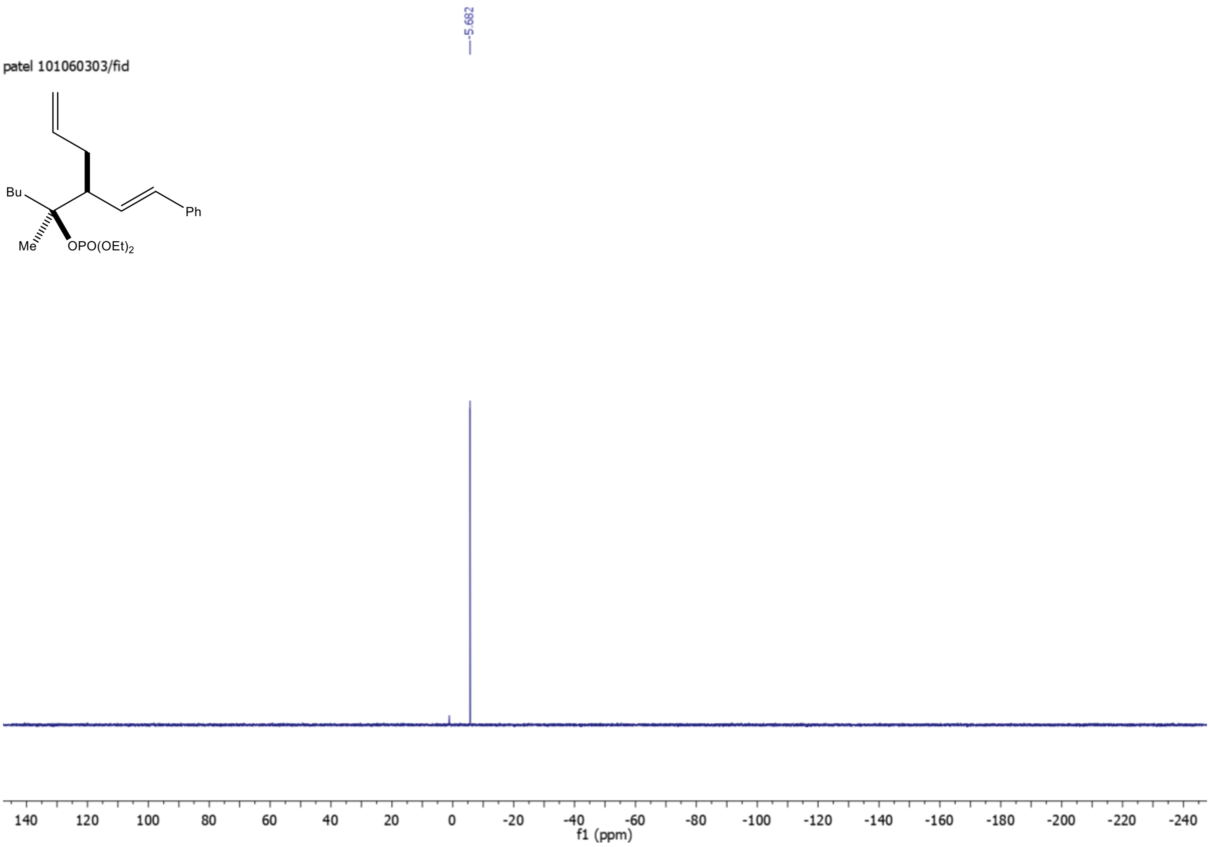

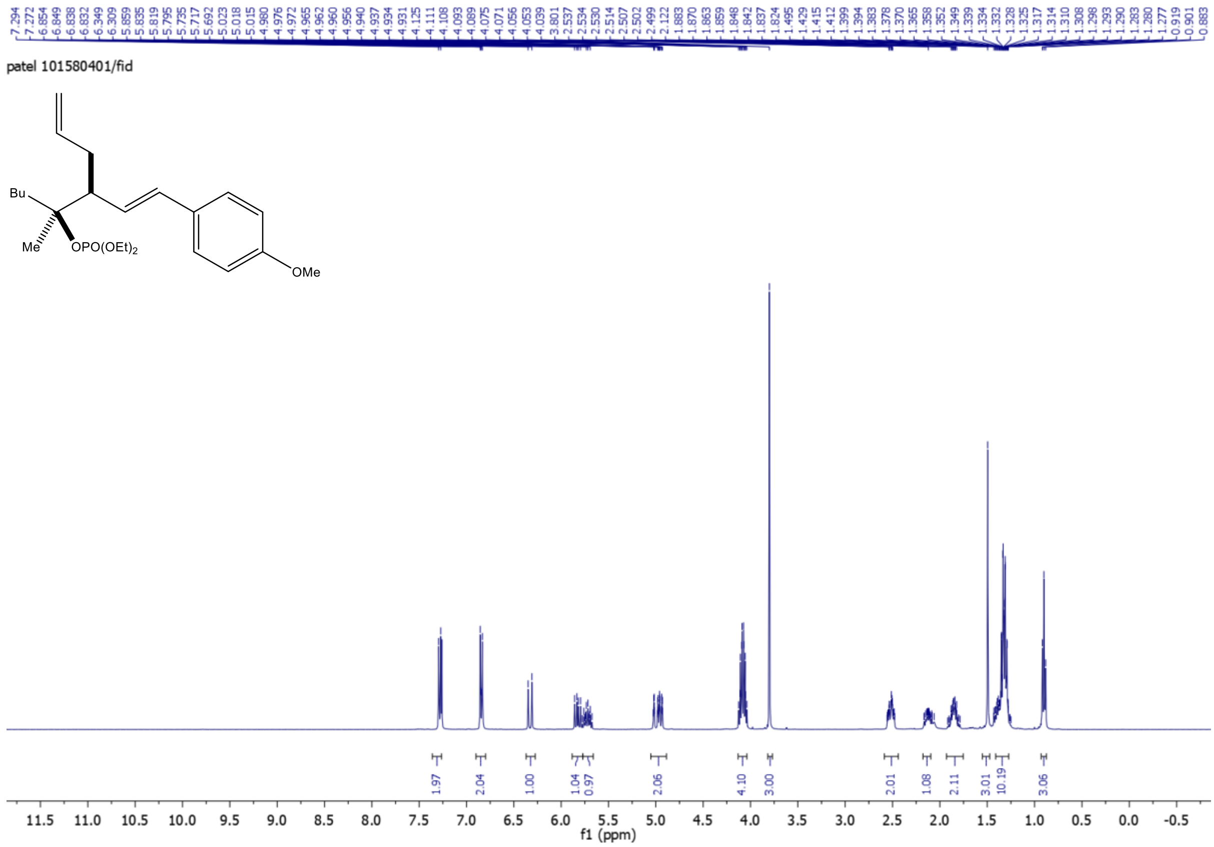
7.294
7.272
6.854
6.849
6.838
6.832
6.349
6.309
5.859
5.835
5.819
5.795
5.735
5.717
5.692
5.023
5.018
5.015
4.980
4.976
4.972
4.965
4.962
4.960
4.956
4.940
4.937
4.934
4.931
4.125
4.111
4.108
4.093
4.089
4.075
4.071
4.056
4.053
4.039
3.801
2.537
2.534
2.530
2.514
2.507
2.502
2.499
2.122
1.883
1.870
1.863
1.859
1.848
1.842
1.837
1.824
1.495
1.429
1.415
1.412
1.399
1.394
1.383
1.378
1.370
1.365
1.358
1.352
1.349
1.339
1.334
1.332
1.328
1.325
1.317
1.314
1.310
1.308
1.298
1.293
1.290
1.283
1.280
1.277
0.919
0.901
0.883
patel 101580401/fid
Bu
Me
OPO(OEt)2
OMe
1.97
2.04
1.00
1.04
0.97
2.06
4.10
3.00
2.01
1.08
2.11
3.01
10.19
3.06
11.5 11.0 10.5 10.0 9.5 9.0 8.5 8.0 7.5 7.0 6.5 6.0 5.5 5.0 4.5 4.0 3.5 3.0 2.5 2.0 1.5 1.0 0.5 0.0 -0.5
f1 (ppm)

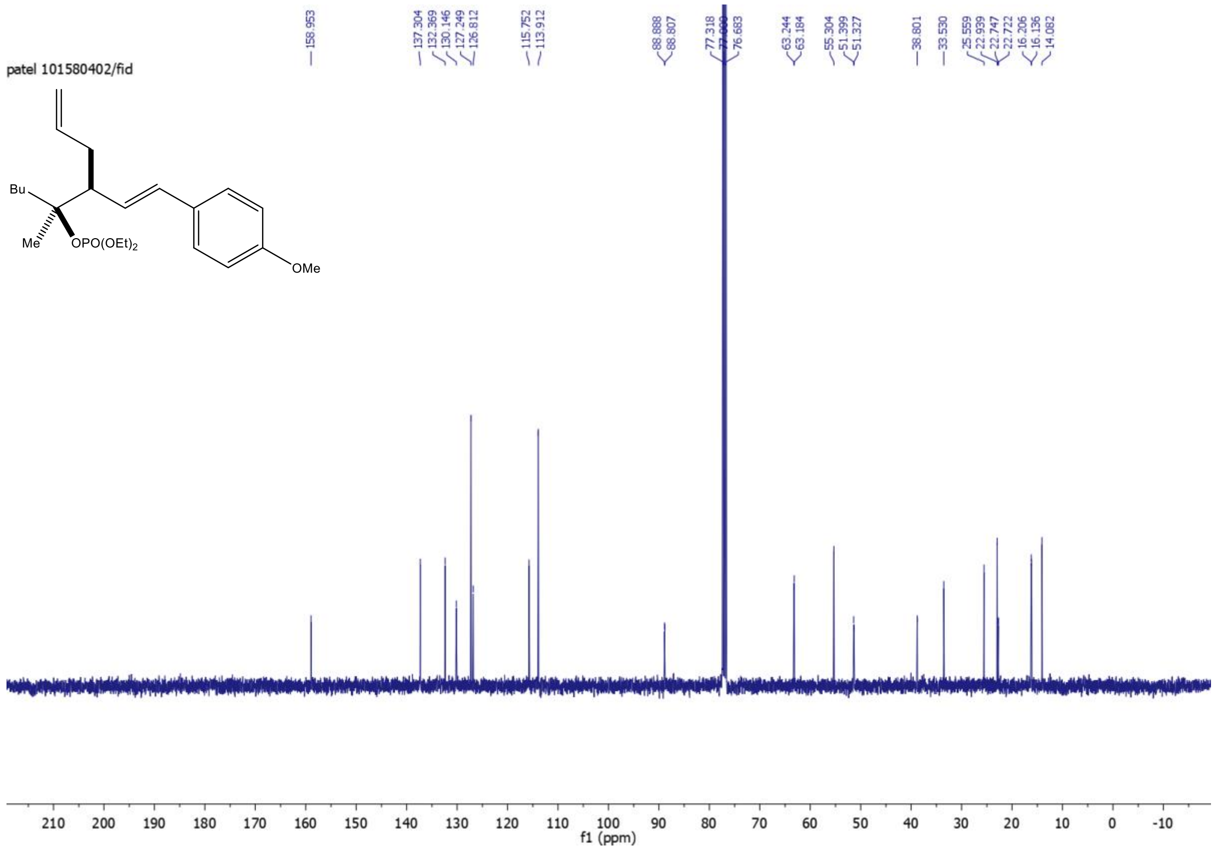
158.953
137.304
132.369
130.146
127.249
126.812
115.752
113.912
88.888
88.807
77.318
77.000
76.683
63.244
63.184
55.304
51.399
51.327
38.801
33.530
25.559
22.939
22.747
22.722
16.206
16.136
14.082
patel 101580402/fid
Bu
Me
OPO(OEt)2
OMe
210 200 190 180 170 160 150 140 130 120 110 100 90 80 70 60 50 40 30 20 10 0 -10
f1 (ppm)

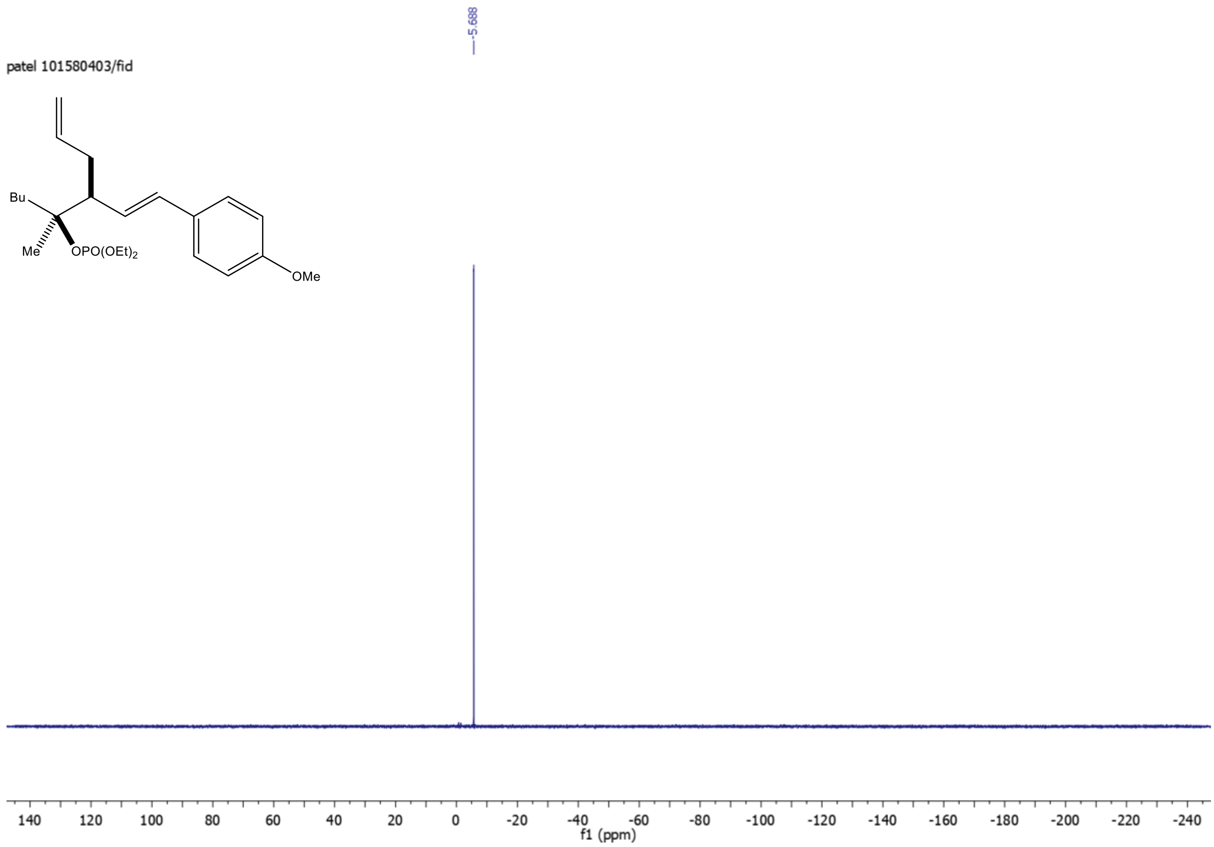
-5.688
patel 101580403/fid
Bu
Me
OPO(OEt)2
OMe
f1 (ppm)

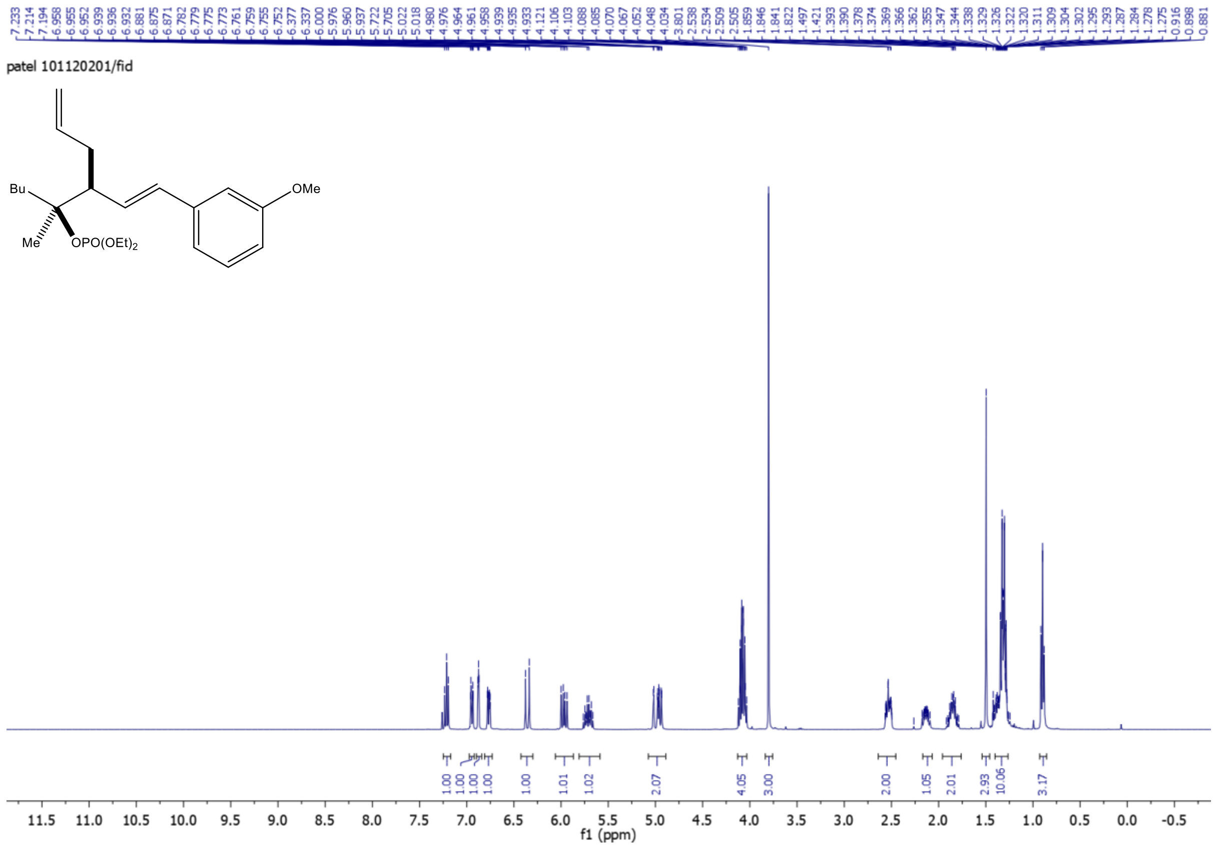
7.233
7.214
7.194
6.958
6.955
6.952
6.939
6.936
6.932
6.881
6.875
6.871
6.782
6.779
6.775
6.773
6.761
6.759
6.755
6.752
6.377
6.337
6.000
5.976
5.960
5.937
5.722
5.705
5.022
5.018
4.980
4.976
4.964
4.961
4.958
4.939
4.935
4.933
4.121
4.106
4.103
4.088
4.085
4.070
4.067
4.052
4.048
4.034
3.801
2.538
2.534
2.509
2.505
1.859
1.846
1.841
1.822
1.497
1.421
1.393
1.390
1.378
1.374
1.369
1.366
1.362
1.355
1.347
1.344
1.338
1.329
1.326
1.322
1.320
1.311
1.309
1.304
1.302
1.295
1.293
1.287
1.284
1.278
1.275
0.916
0.898
0.881
patel 101120201/fid
Bu
Me
OPO(OEt)2
OMe
1.00
1.00
1.00
1.00
1.00
1.01
1.02
2.07
4.05
3.00
2.00
1.05
2.01
2.93
10.06
3.17
f1 (ppm)

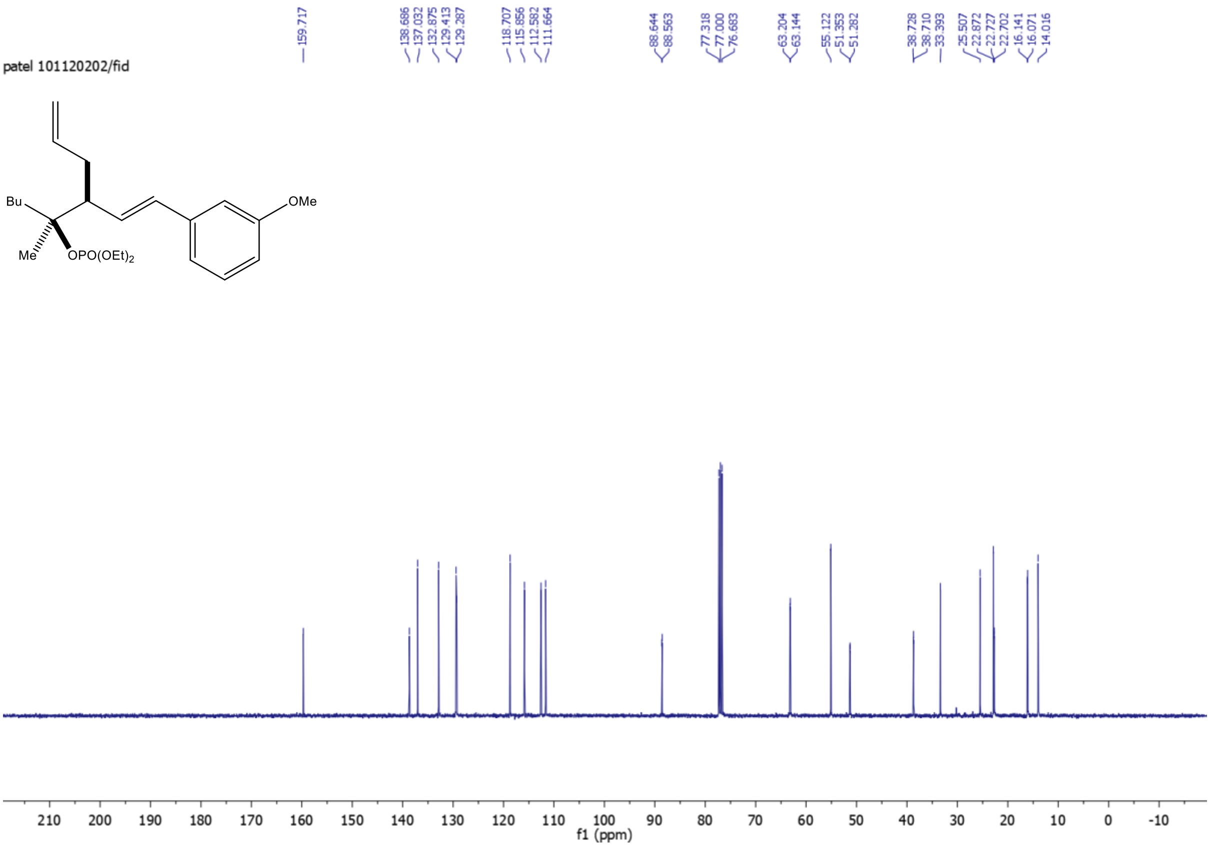
patel 101120202/fid
159.717
138.686
137.032
132.875
129.413
129.287
118.707
115.856
112.582
111.664
88.644
88.563
77.318
77.000
76.683
63.204
63.144
55.122
51.353
51.282
38.728
38.710
33.393
25.507
22.872
22.727
22.702
16.141
16.071
14.016
Bu
Me
OPO(OEt)2
OMe
f1 (ppm)

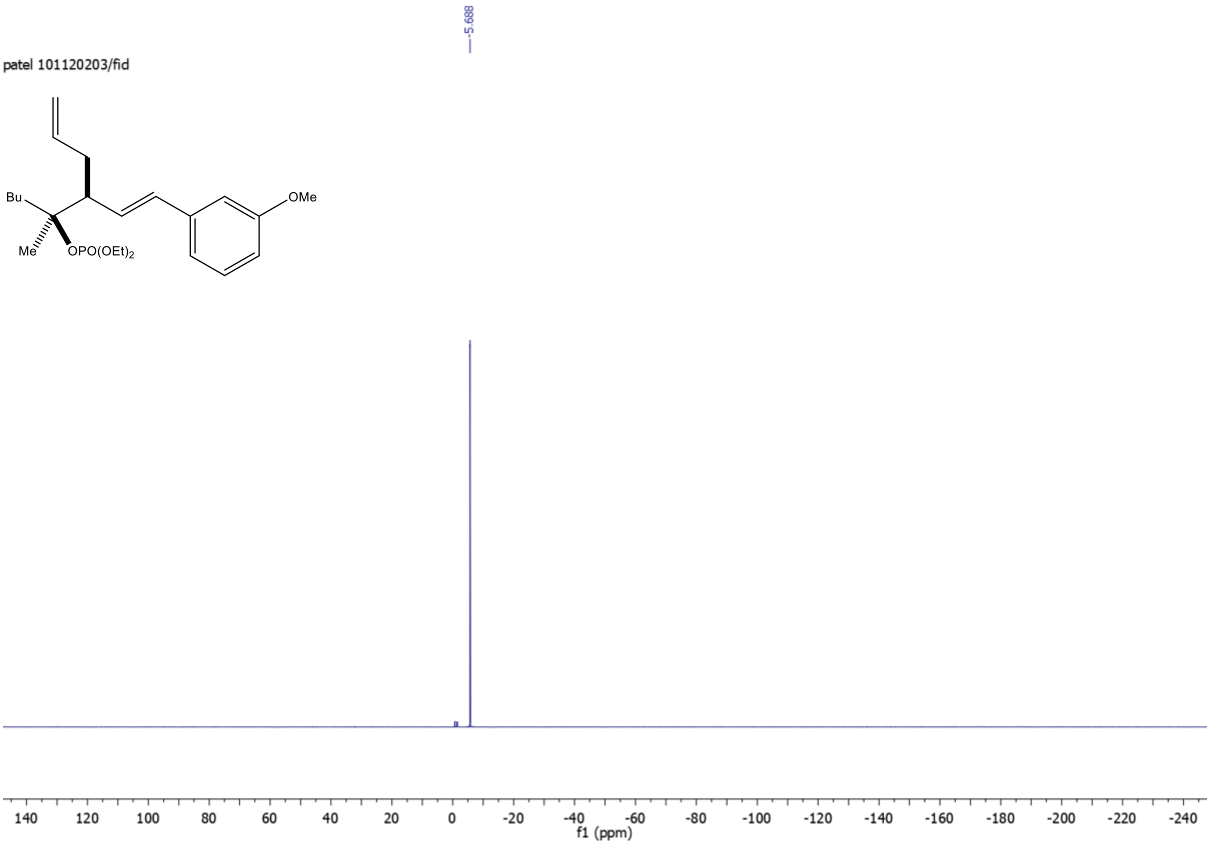
patel 101120203/fid
-5.688
Bu
Me
OPO(OEt)2
OMe
f1 (ppm)

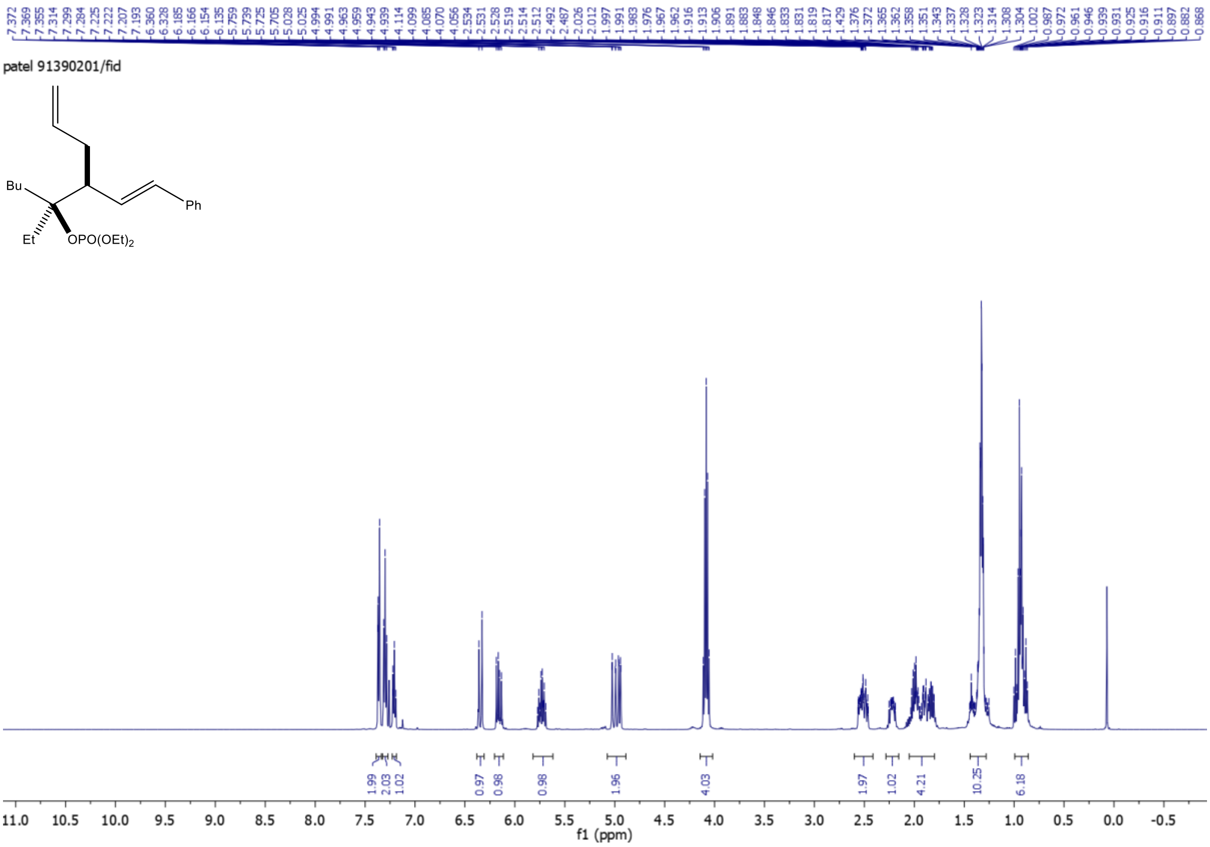
patel 91390201/fid
Bu
Et
OPO(OEt)2
Ph
f1 (ppm)

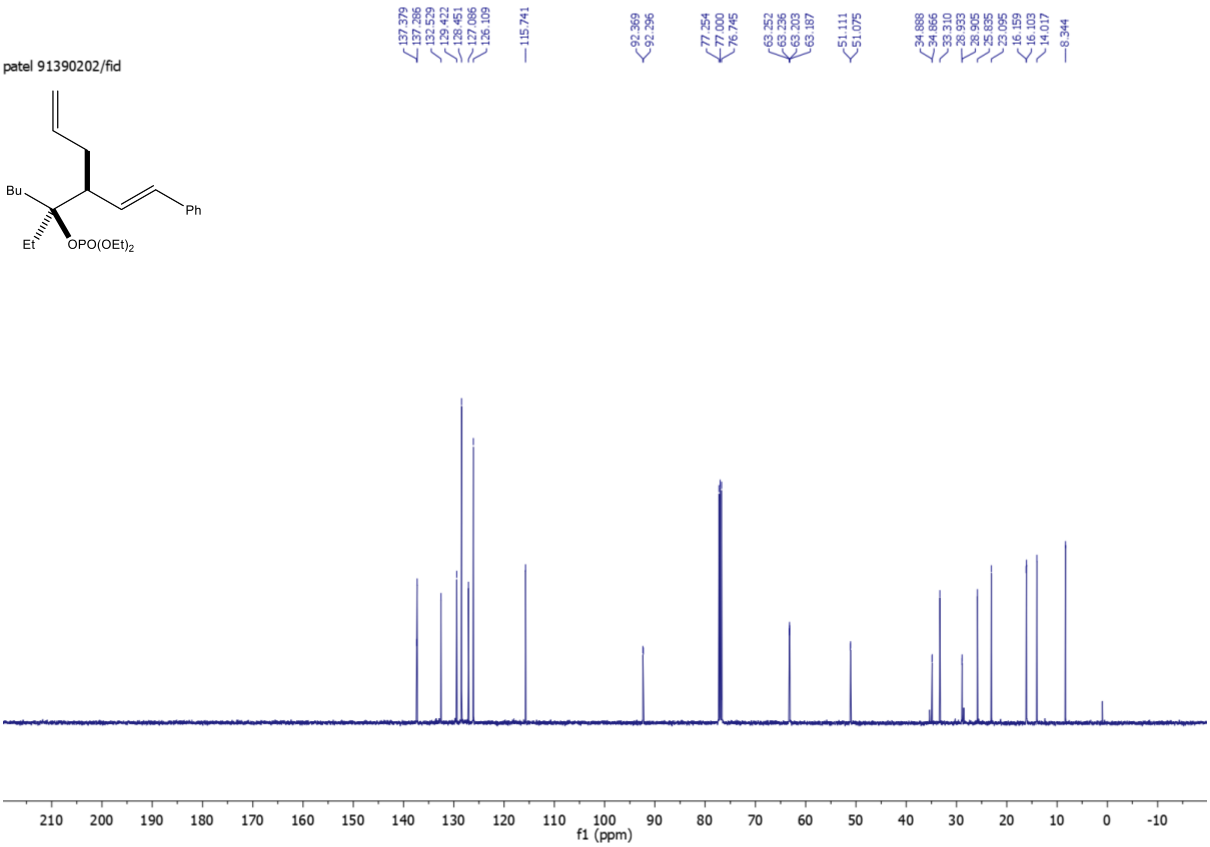
patel 91390202/fid
Bu
Et
OPO(OEt)2
Ph
f1 (ppm)

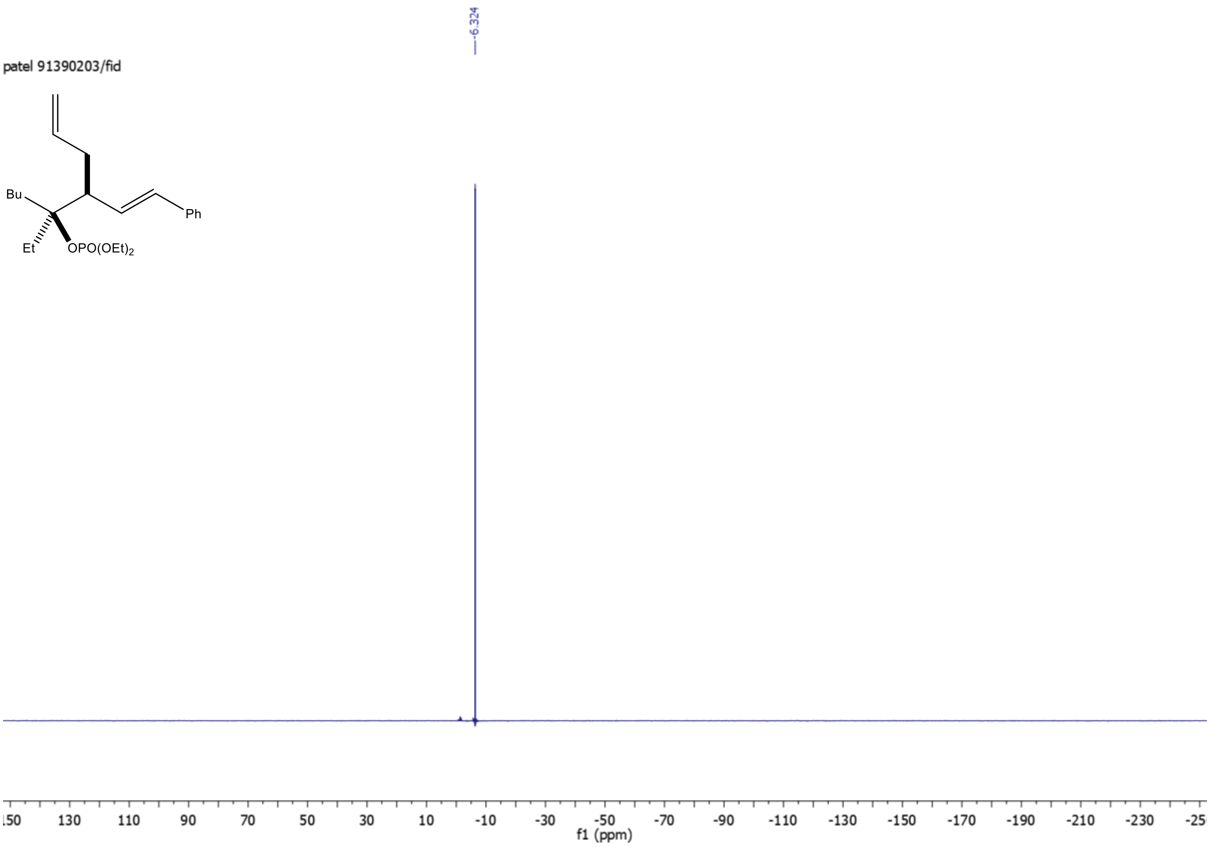
patel 91390203/fid
-6.324
Bu
Et
OPO(OEt)2
Ph
f1 (ppm)

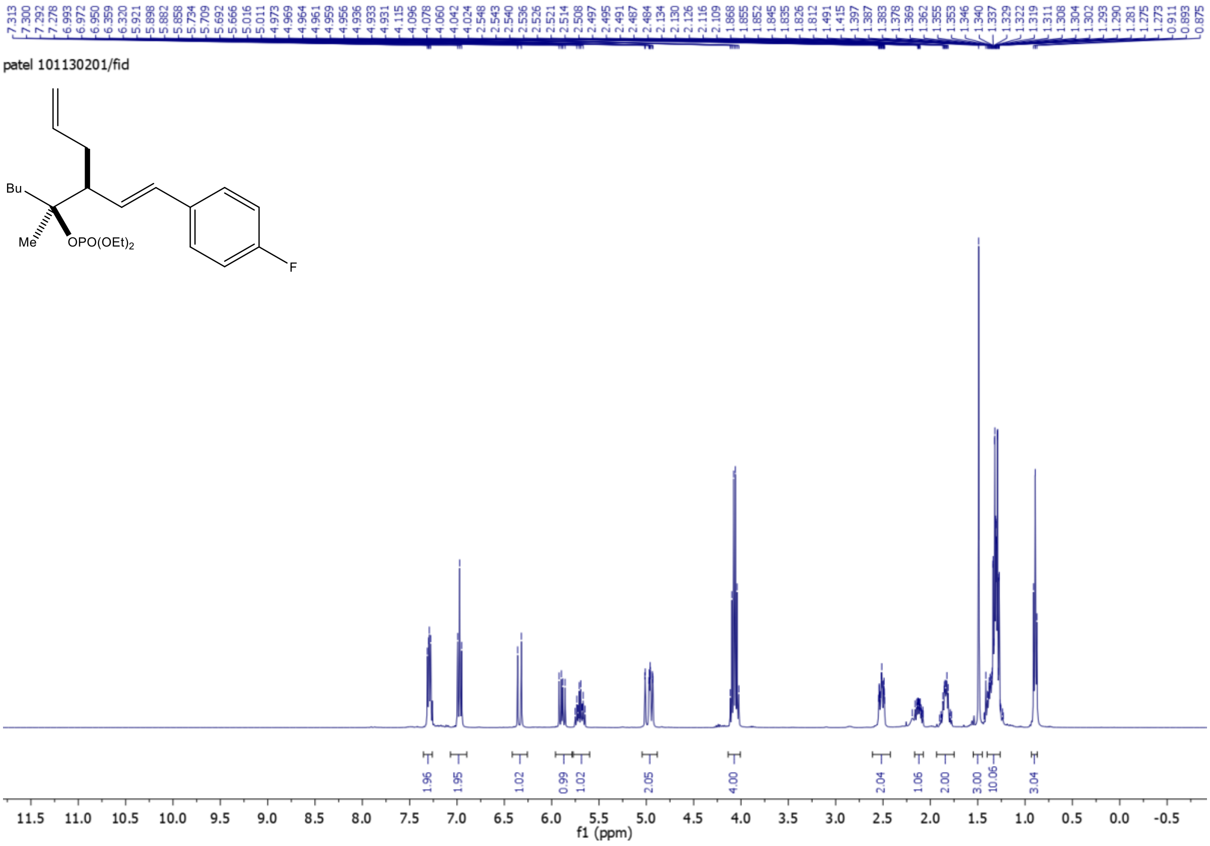
7.313 7.300 7.292 7.278 6.993 6.972 6.950 6.359 6.320 5.921 5.898 5.882 5.858 5.734 5.709 5.692 5.666 5.016 5.011 4.973 4.969 4.964 4.961 4.959 4.956 4.936 4.933 4.931 4.115 4.096 4.078 4.060 4.042 4.024 2.548 2.543 2.540 2.536 2.526 2.521 2.514 2.508 2.497 2.495 2.491 2.487 2.484 2.134 2.130 2.126 2.116 2.109 1.868 1.855 1.852 1.845 1.835 1.826 1.812 1.491 1.415 1.397 1.387 1.383 1.378 1.369 1.362 1.355 1.353 1.346 1.340 1.337 1.329 1.322 1.319 1.311 1.308 1.304 1.302 1.293 1.290 1.281 1.275 1.273 0.911 0.893 0.875
patel 101130201/fid
Bu
Me
OPO(OEt)2
F
1.96 1.95 1.02 0.99 1.02 2.05 4.00 2.04 1.06 2.00 3.00 10.06 3.04
f1 (ppm)

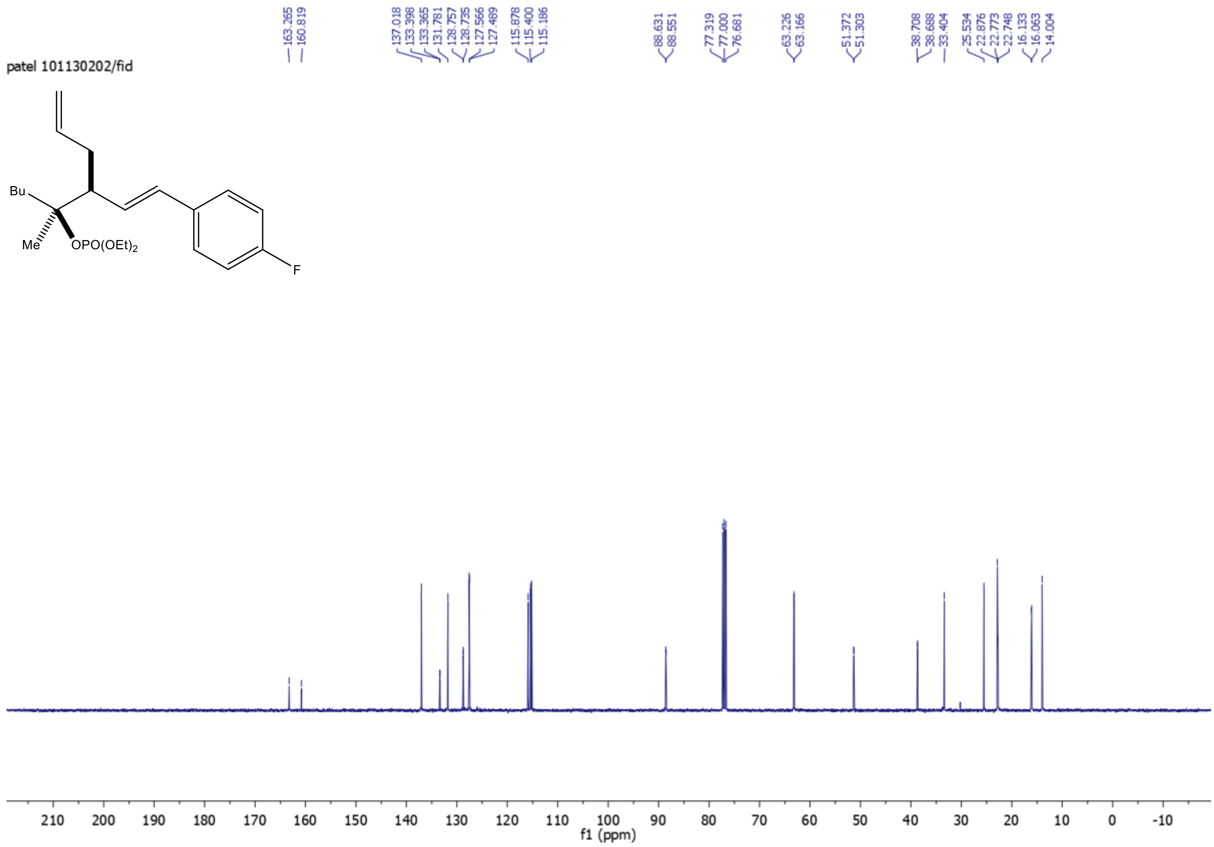
patel 101130202/fid
163.265
160.819
137.018
133.398
133.365
131.781
128.757
128.735
127.566
127.489
115.878
115.400
115.186
88.631
88.551
77.319
77.000
76.681
63.226
63.166
51.372
51.303
38.708
38.688
33.404
25.534
22.876
22.773
22.748
16.133
16.063
14.004
Bu
Me
OPO(OEt)2
F
f1 (ppm)

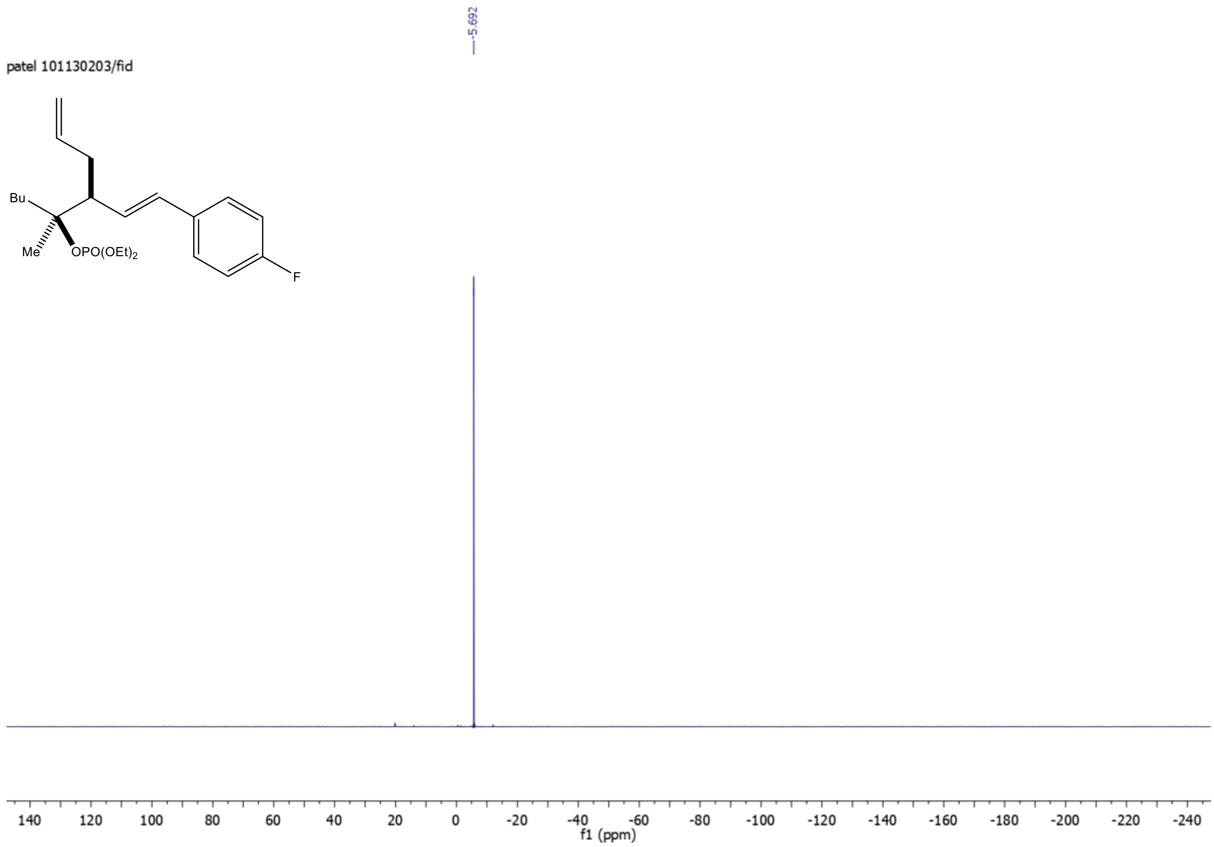
patel 101130203/fid
-5.692
Bu
Me
OPO(OEt)2
F
f1 (ppm)

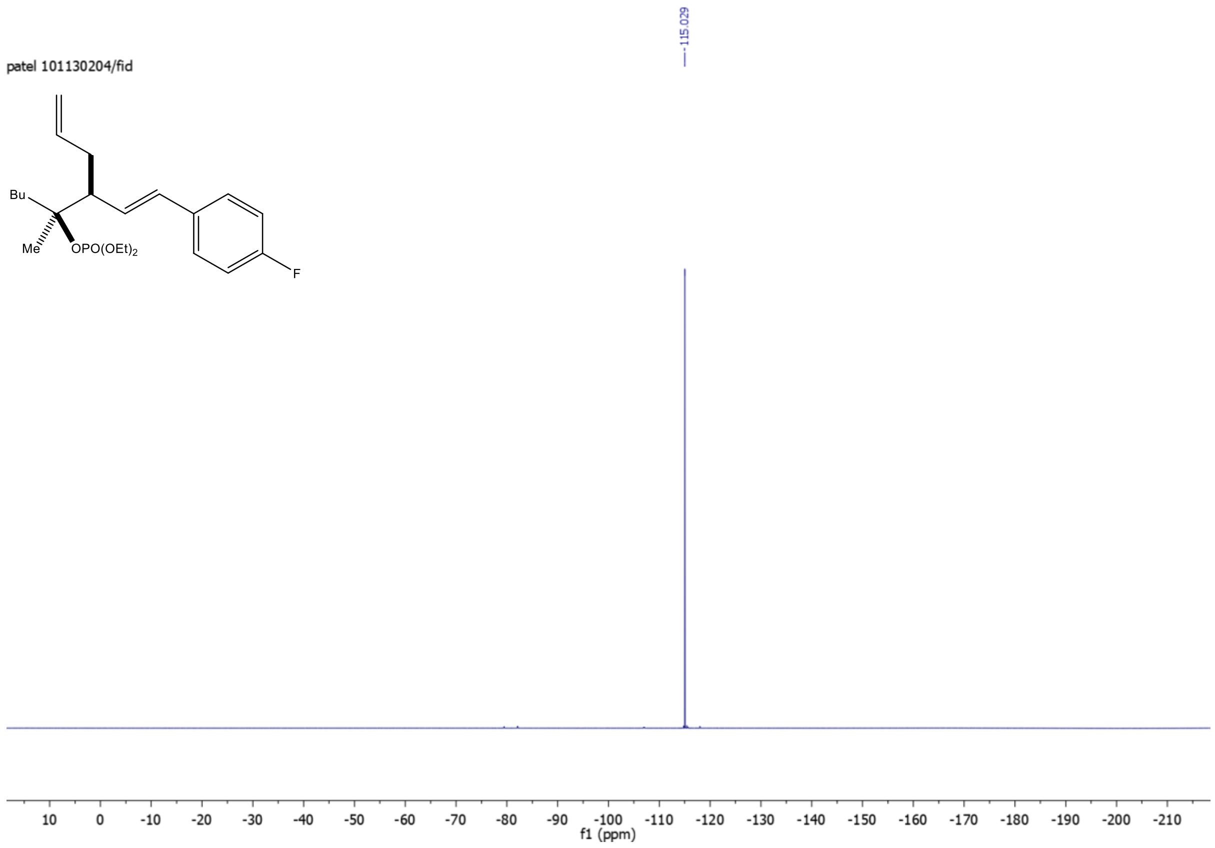
patel 101130204/fid
-115.029
Bu
Me
OPO(OEt)2
F
10 0 -10 -20 -30 -40 -50 -60 -70 -80 -90 -100 -110 -120 -130 -140 -150 -160 -170 -180 -190 -200 -210
f1 (ppm)

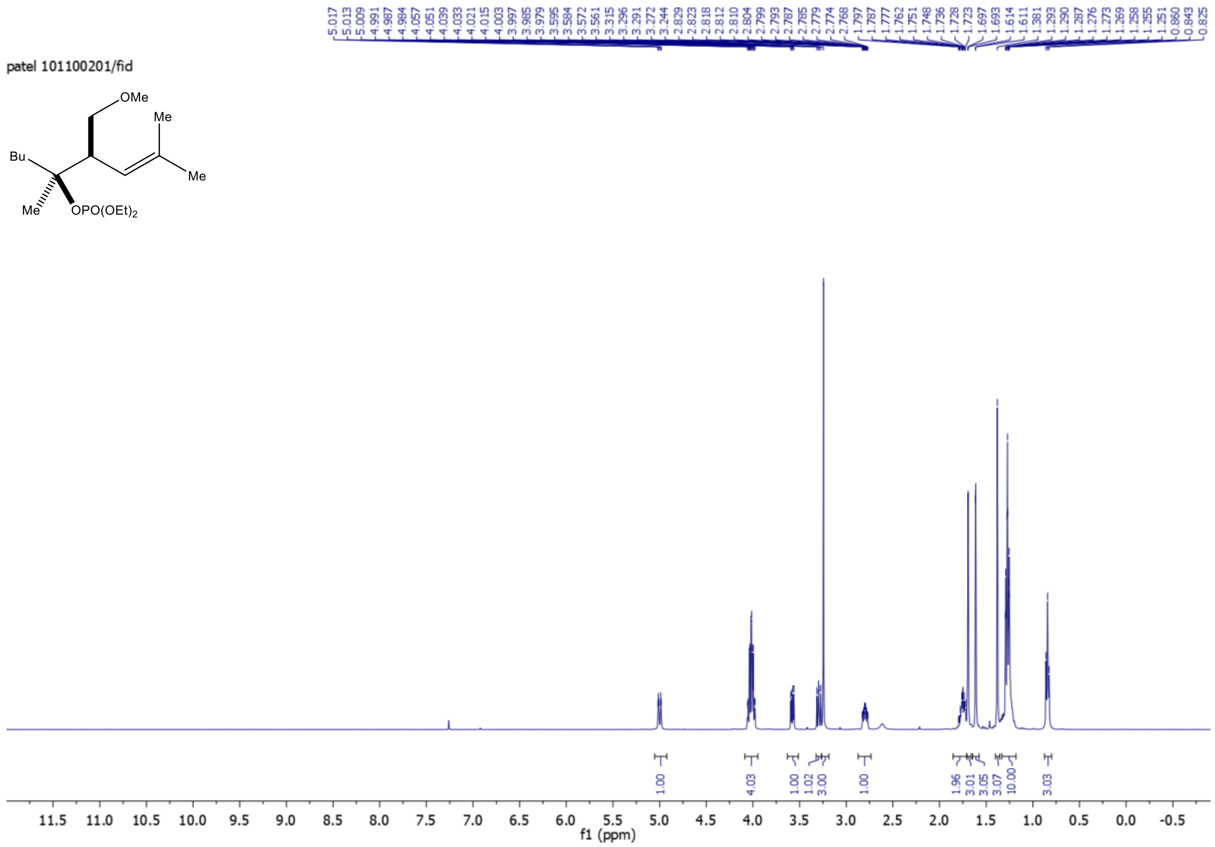
patel 101100201/fid
5.017 5.013 5.009 4.991 4.987 4.984 4.057 4.051 4.039 4.033 4.021 4.015 4.003 3.997 3.985 3.979 3.595 3.584 3.572 3.561 3.315 3.296 3.291 3.272 3.244 2.829 2.823 2.818 2.812 2.810 2.804 2.799 2.793 2.787 2.785 2.779 2.774 2.768 1.797 1.787 1.777 1.762 1.751 1.748 1.736 1.728 1.723 1.697 1.693 1.614 1.611 1.381 1.293 1.290 1.287 1.276 1.273 1.269 1.258 1.255 1.251 0.860 0.843 0.825
OMe
Me
Bu
Me
Me
OPO(OEt)2
1.00 4.03 1.00 1.02 3.00 1.00 1.96 3.01 3.05 3.07 10.00 3.03
11.5 11.0 10.5 10.0 9.5 9.0 8.5 8.0 7.5 7.0 6.5 6.0 5.5 5.0 4.5 4.0 3.5 3.0 2.5 2.0 1.5 1.0 0.5 0.0 -0.5
f1 (ppm)

patel 101100202/fid

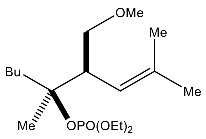

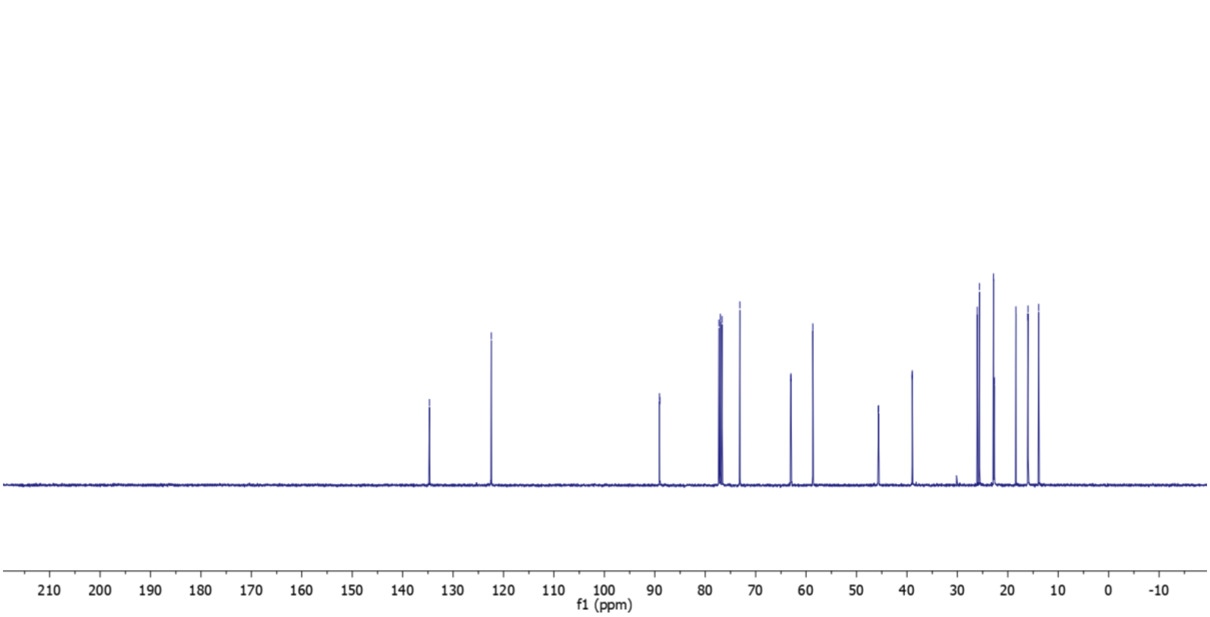

patel 101100203/fid

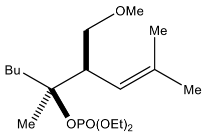

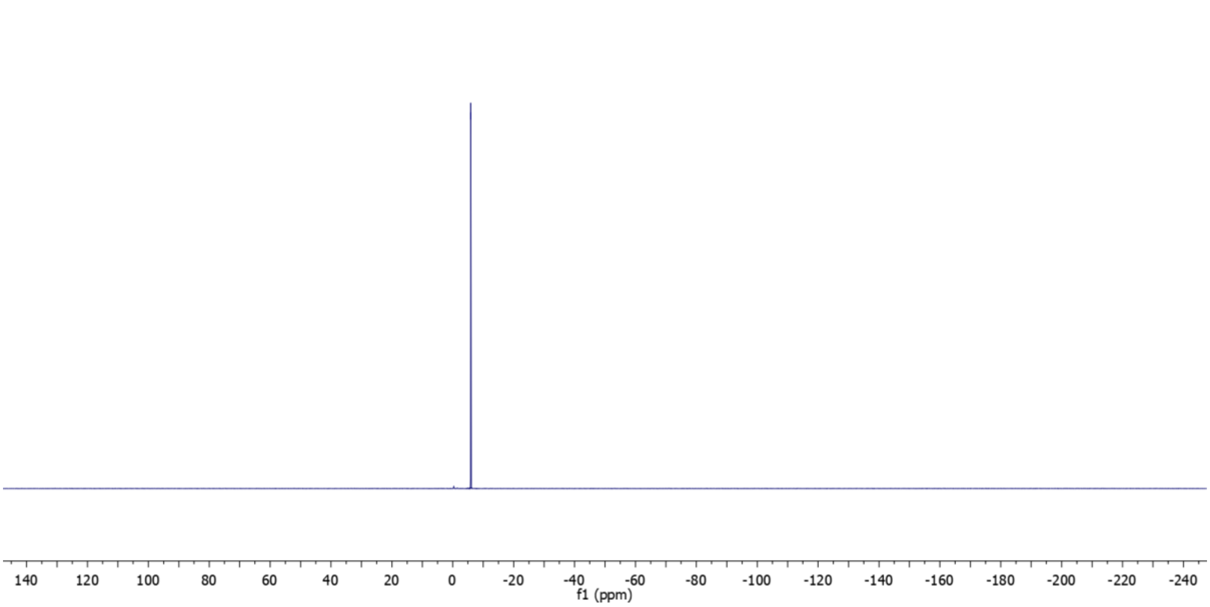

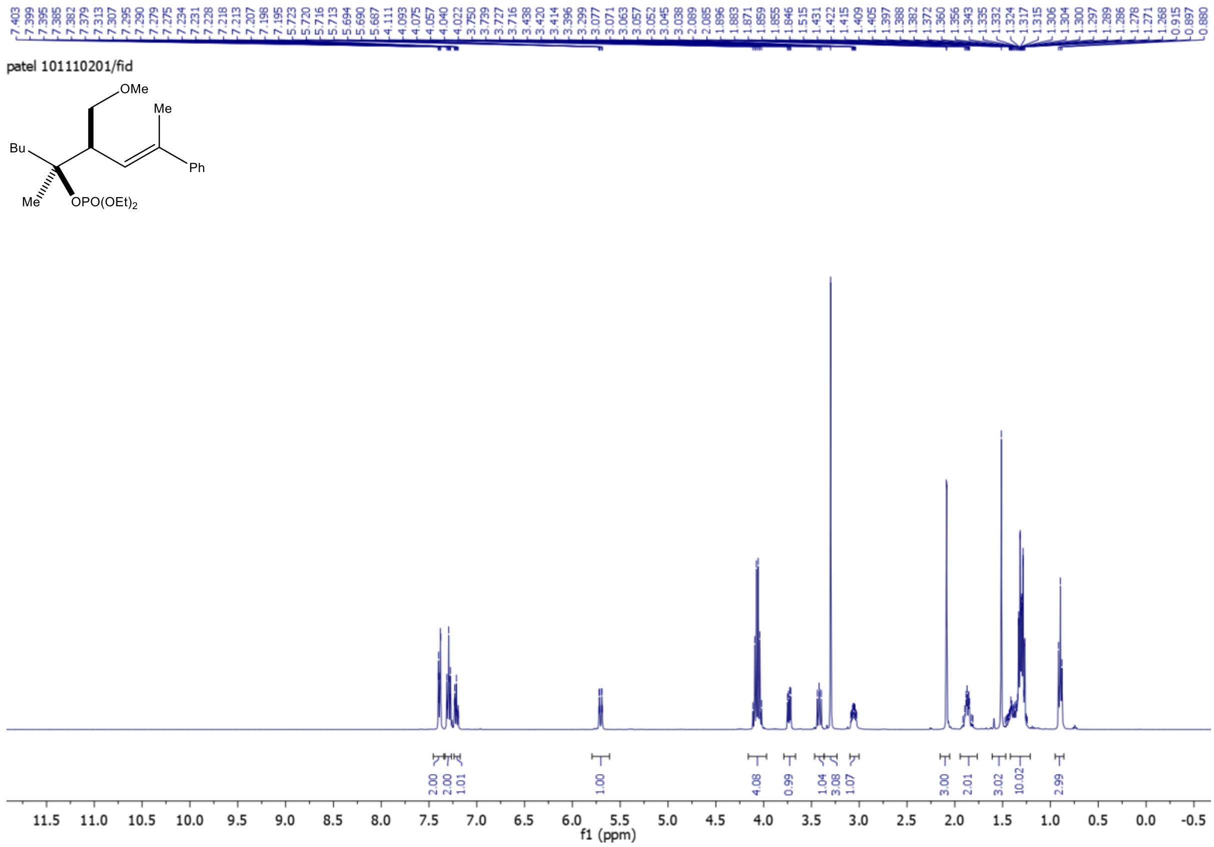
patel 101110201/fid
7.403 7.399 7.395 7.385 7.382 7.379 7.313 7.307 7.295 7.290 7.279 7.275 7.234 7.231 7.228 7.218 7.213 7.207 7.198 7.195 5.723 5.720 5.716 5.713 5.694 5.690 5.687 4.111 4.093 4.075 4.057 4.040 4.022 3.750 3.739 3.727 3.716 3.438 3.420 3.414 3.396 3.299 3.077 3.071 3.063 3.057 3.052 3.045 3.038 2.089 2.085 1.896 1.883 1.871 1.859 1.855 1.846 1.515 1.431 1.422 1.415 1.409 1.405 1.397 1.388 1.382 1.372 1.360 1.356 1.343 1.335 1.332 1.324 1.317 1.315 1.306 1.304 1.300 1.297 1.289 1.286 1.278 1.271 1.268 0.915 0.897 0.880
OMe
Me
Bu
Ph
Me
OPO(OEt)2
2.00 2.00 1.01 1.00 4.08 0.99 1.04 3.08 1.07 3.00 2.01 3.02 10.02 2.99
f1 (ppm)

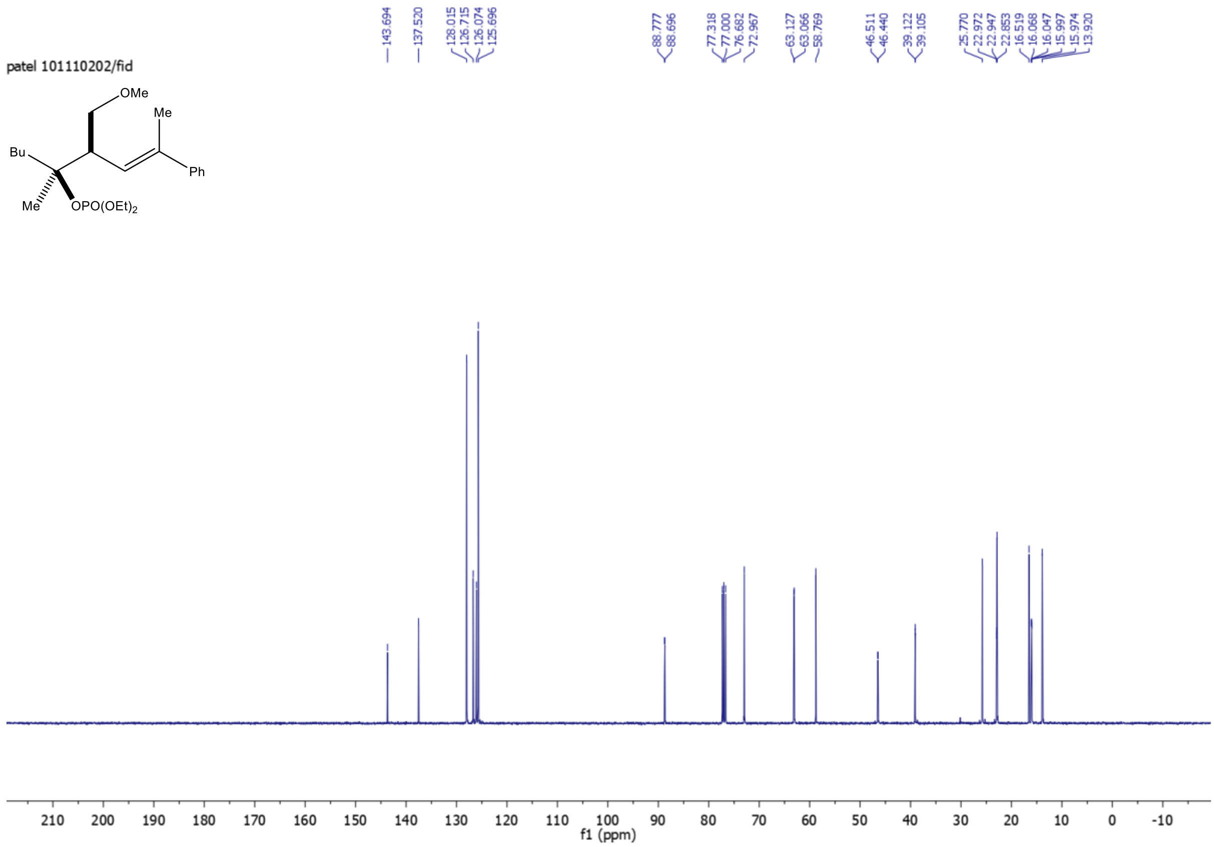
patel 101110202/fid
143.694 137.520 128.015 126.715 126.074 125.696 88.777 88.696 77.318 77.000 76.682 72.967 63.127 63.066 58.769 46.511 46.440 39.122 39.105 25.770 22.972 22.947 22.853 16.519 16.068 16.047 15.997 15.974 13.920
OMe
Me
Bu
Ph
Me
OPO(OEt)2
f1 (ppm)

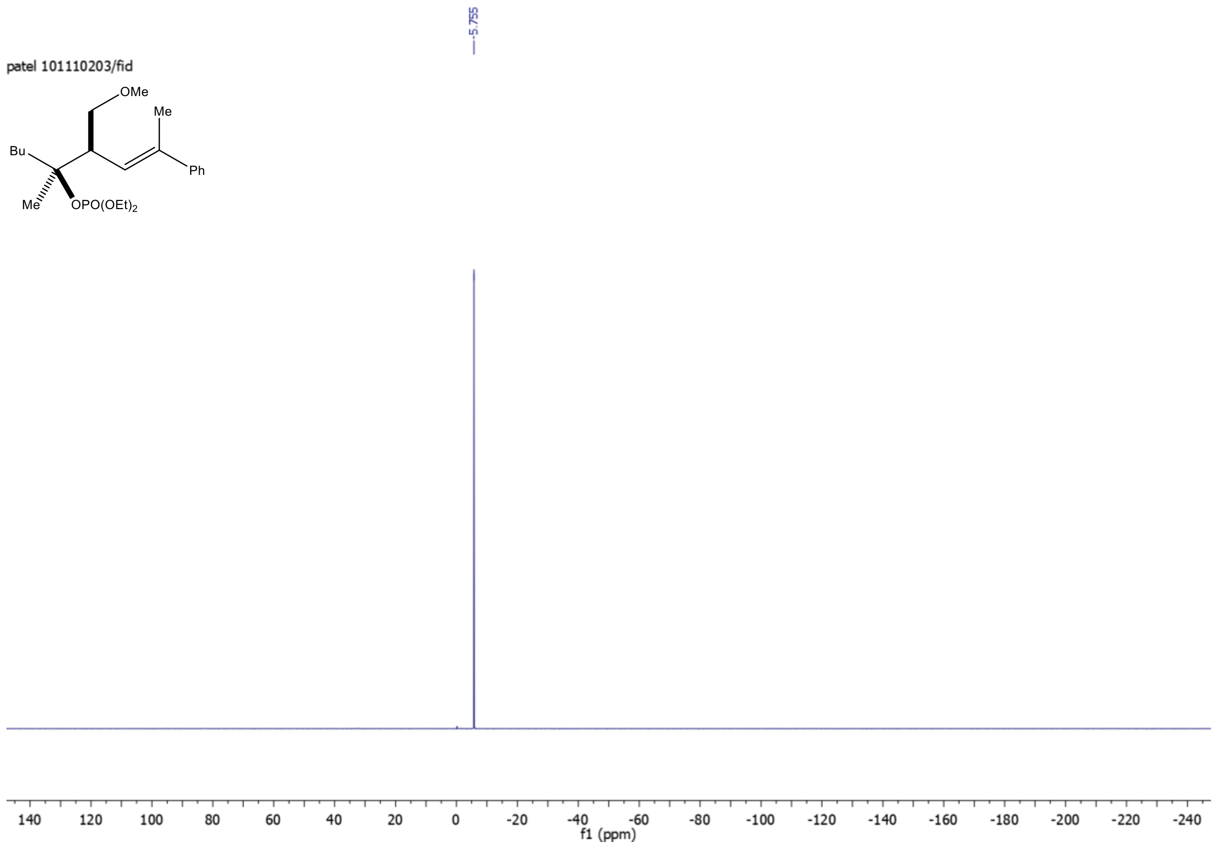
patel 101110203/fid
OMe
Me
Bu
Ph
Me
OPO(OEt)2
-5.755
140 120 100 80 60 40 20 0 -20 -40 -60 -80 -100 -120 -140 -160 -180 -200 -220 -240
f1 (ppm)

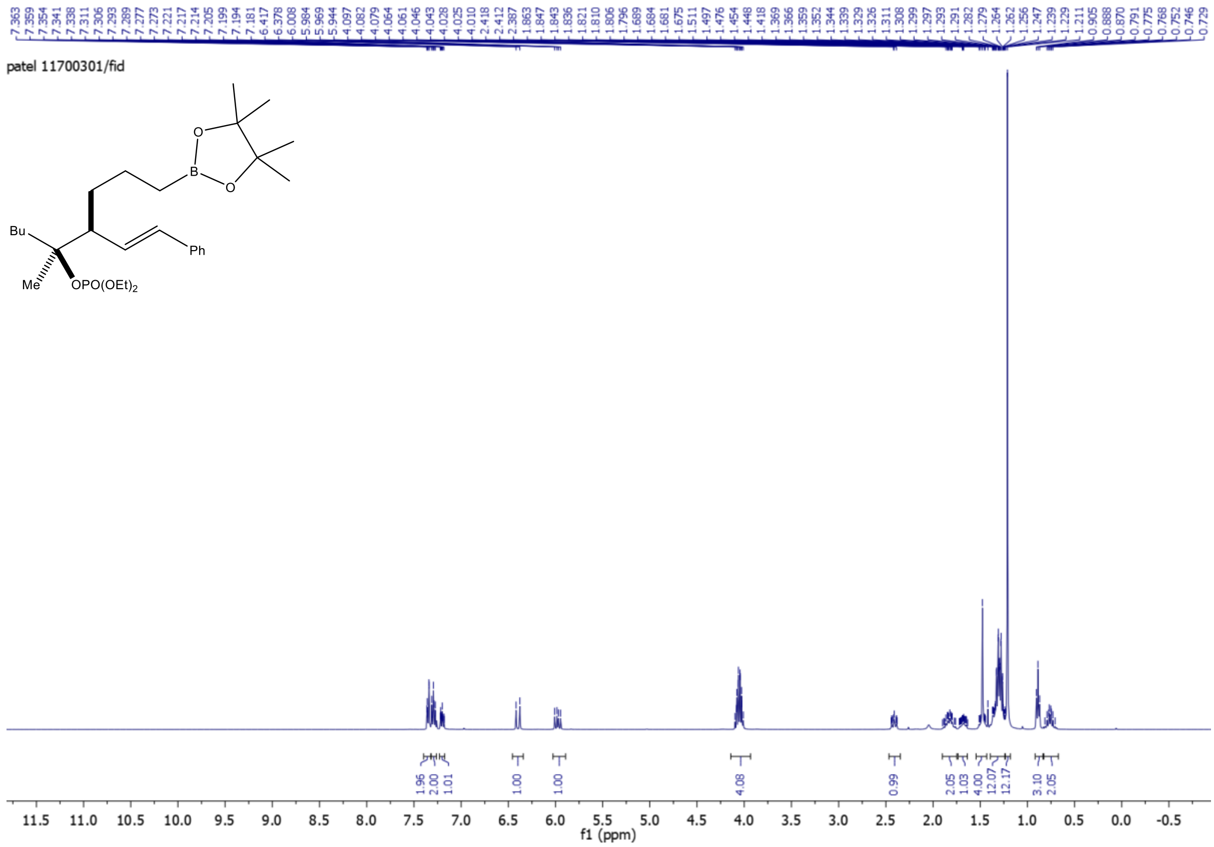
7.363 7.359 7.354 7.341 7.338 7.311 7.306 7.293 7.289 7.277 7.273 7.221 7.217 7.214 7.205 7.199 7.194 7.181 6.417 6.378 6.008 5.984 5.969 5.944 4.097 4.082 4.079 4.064 4.061 4.046 4.043 4.028 4.025 4.010 2.418 2.412 2.387 1.863 1.847 1.843 1.836 1.821 1.810 1.806 1.796 1.689 1.684 1.681 1.675 1.511 1.497 1.476 1.454 1.448 1.418 1.369 1.366 1.359 1.352 1.344 1.339 1.329 1.326 1.311 1.308 1.299 1.297 1.293 1.291 1.282 1.279 1.264 1.262 1.256 1.247 1.239 1.229 1.211 0.905 0.888 0.870 0.791 0.775 0.768 0.752 0.746 0.729
patel 11700301/fid
O
B
O
Bu
Ph
Me
OPO(OEt)2
1.96 2.00 1.01 1.00 1.00 4.08 0.99 2.05 1.03 4.00 12.07 12.17 3.10 2.05
11.5 11.0 10.5 10.0 9.5 9.0 8.5 8.0 7.5 7.0 6.5 6.0 5.5 5.0 4.5 4.0 3.5 3.0 2.5 2.0 1.5 1.0 0.5 0.0 -0.5
f1 (ppm)

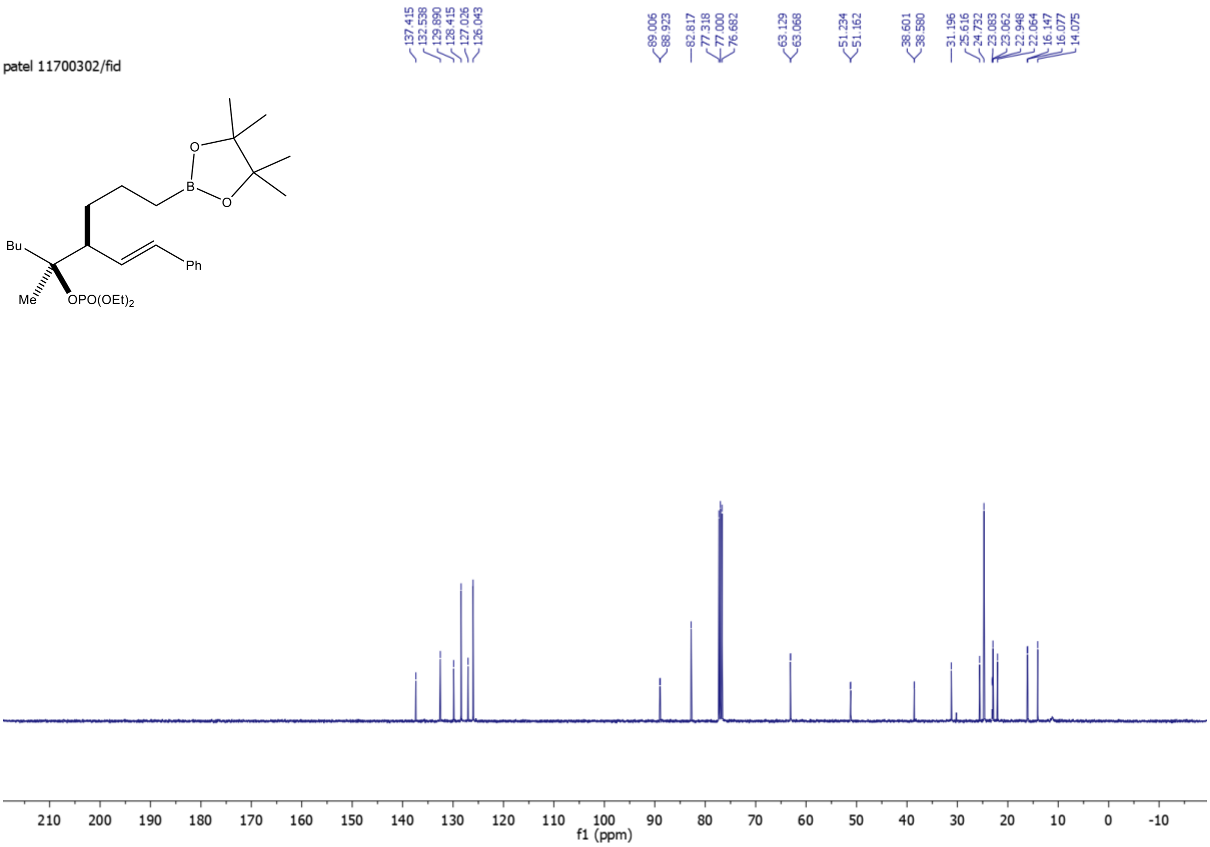
patel 11700302/fid
137.415
132.538
129.890
128.415
127.026
126.043
89.006
88.923
82.817
77.318
77.000
76.682
63.129
63.068
51.234
51.162
38.601
38.580
31.196
25.616
24.732
23.083
23.062
22.948
22.064
16.147
16.077
14.075
O
B
O
Bu
Ph
Me
OPO(OEt)2
f1 (ppm)

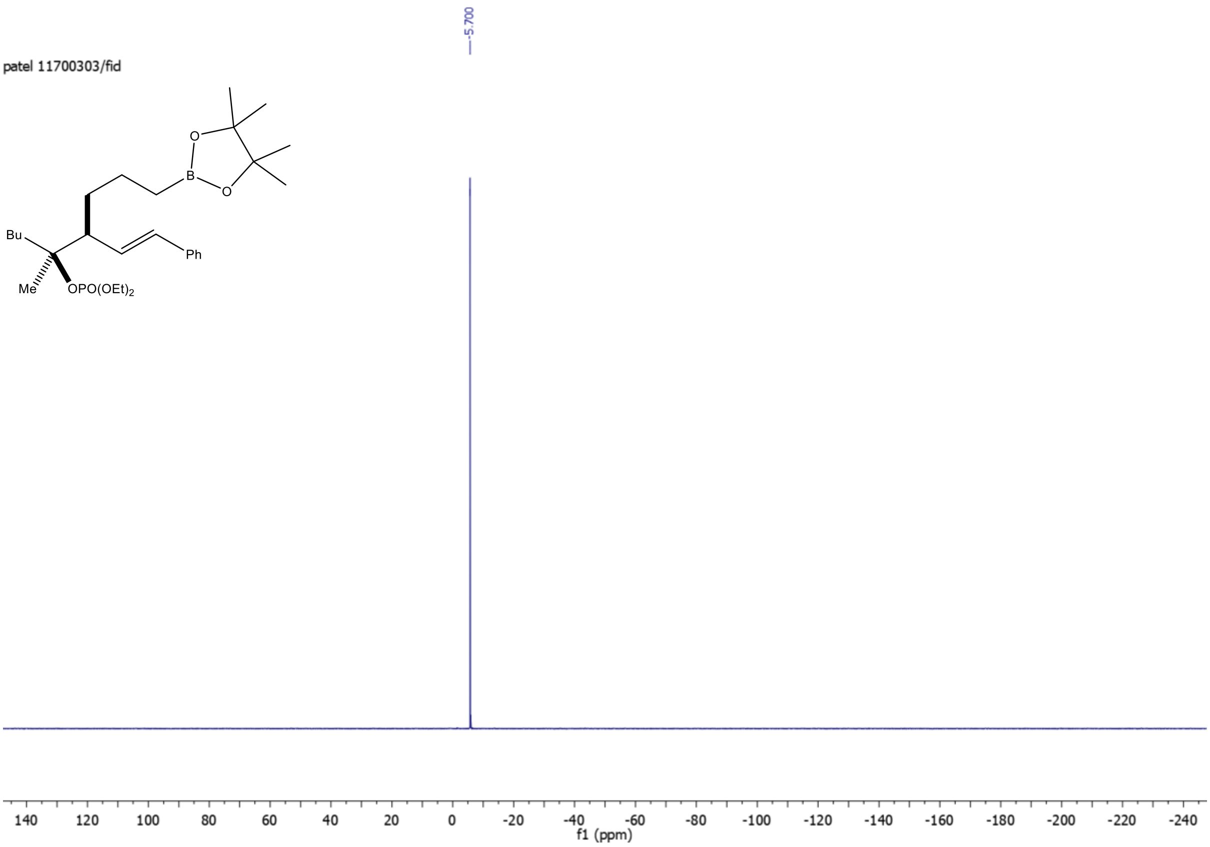
patel 11700303/fid
-5.700
O
B
O
Bu
Ph
Me
OPO(OEt)2
f1 (ppm)

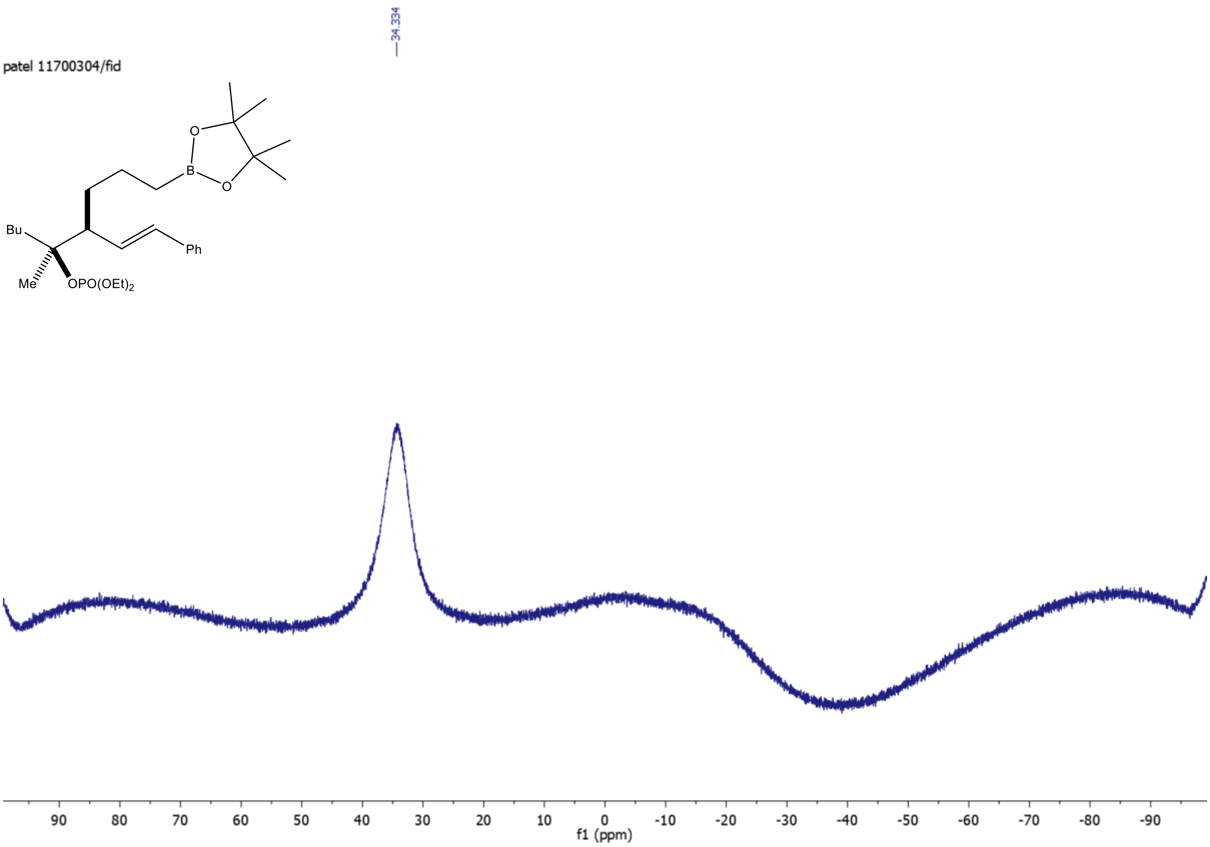

Characterization spectra for products

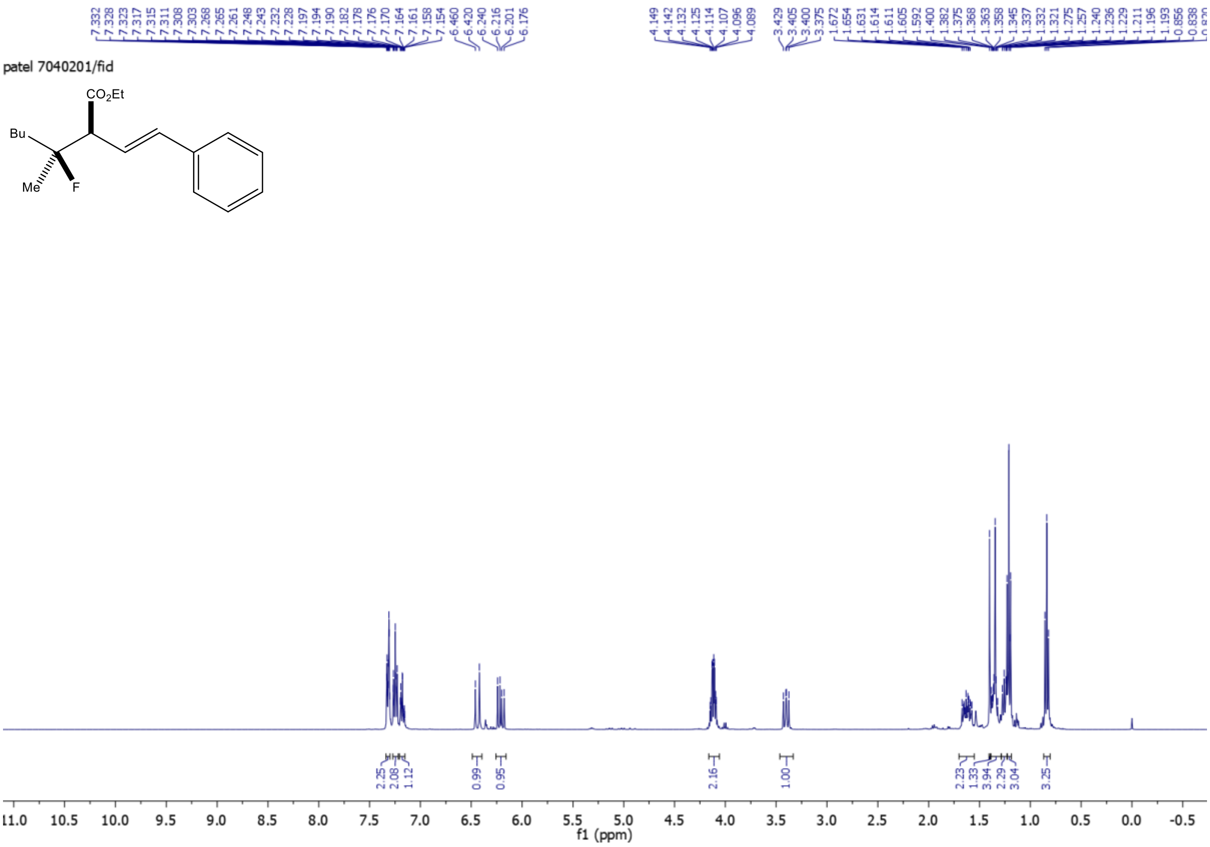

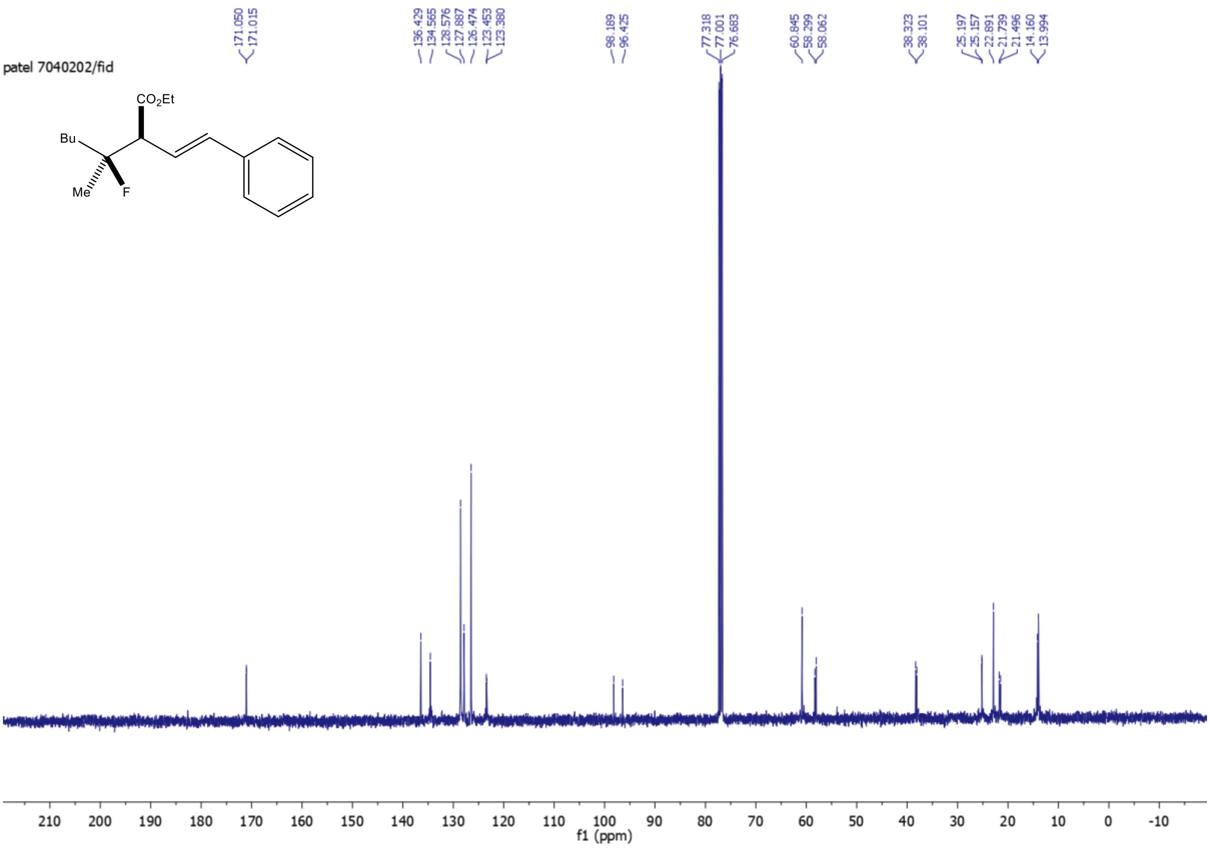
patel 7040202/fid
CO2Et
Bu
Me
F
171.050
171.015
136.429
134.565
128.576
127.887
126.474
123.453
123.380
98.189
96.425
77.318
77.001
76.683
60.845
58.299
58.062
38.323
38.101
25.197
25.157
22.891
21.739
21.496
14.160
13.994
f1 (ppm)

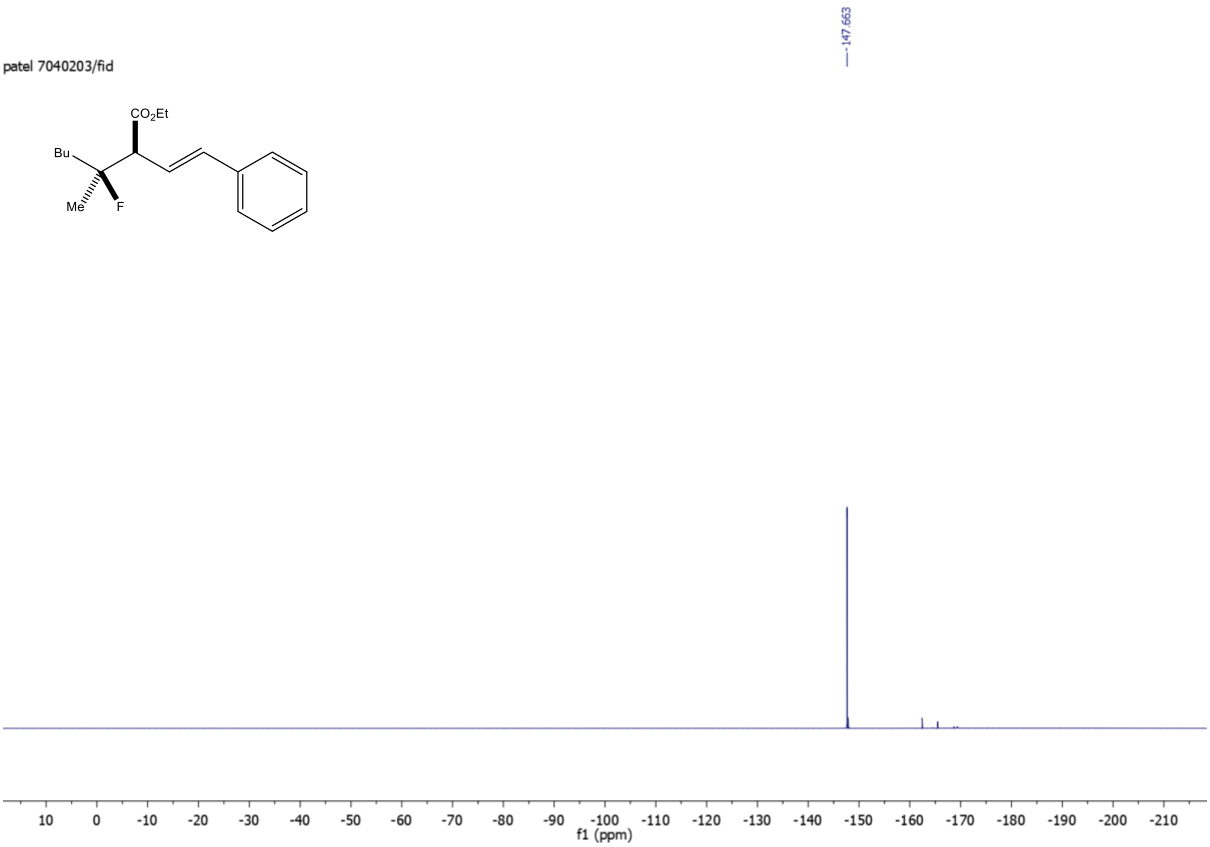
patel 7040203/fid
CO2Et
Bu
Me
F
-147.663
f1 (ppm)

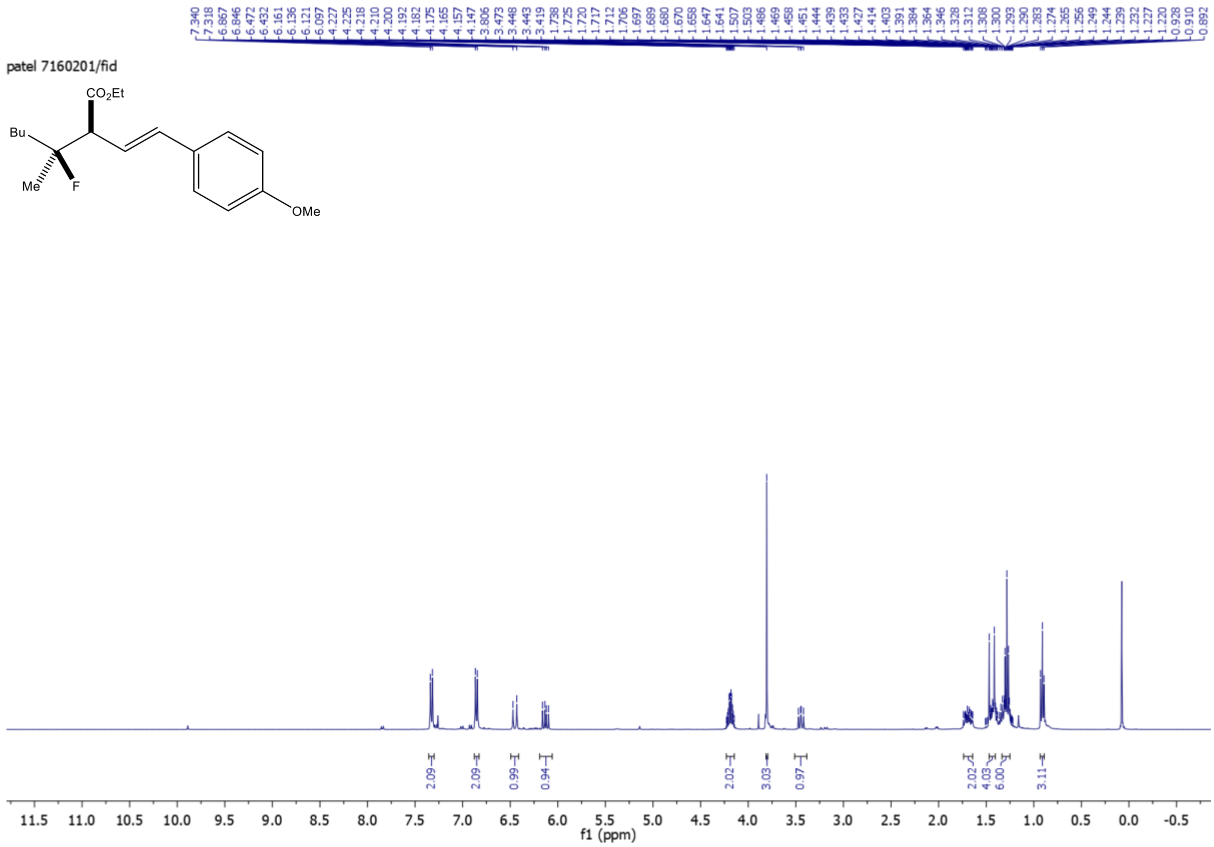
patel 7160201/fid
CO2Et
Bu
Me
F
OMe
7.340 7.318 6.867 6.846 6.472 6.432 6.161 6.136 6.121 6.097 4.227 4.225 4.218 4.210 4.200 4.192 4.182 4.175 4.165 4.157 4.147 3.806 3.473 3.448 3.443 3.419 1.738 1.725 1.720 1.717 1.712 1.706 1.697 1.689 1.680 1.670 1.658 1.647 1.641 1.507 1.503 1.486 1.469 1.458 1.451 1.444 1.439 1.433 1.427 1.414 1.403 1.391 1.384 1.364 1.346 1.328 1.312 1.308 1.300 1.293 1.290 1.283 1.274 1.265 1.256 1.249 1.244 1.239 1.232 1.227 1.220 0.928 0.910 0.892
2.09 2.09 0.99 0.94 2.02 3.03 0.97 2.02 4.03 6.00 3.11
f1 (ppm)

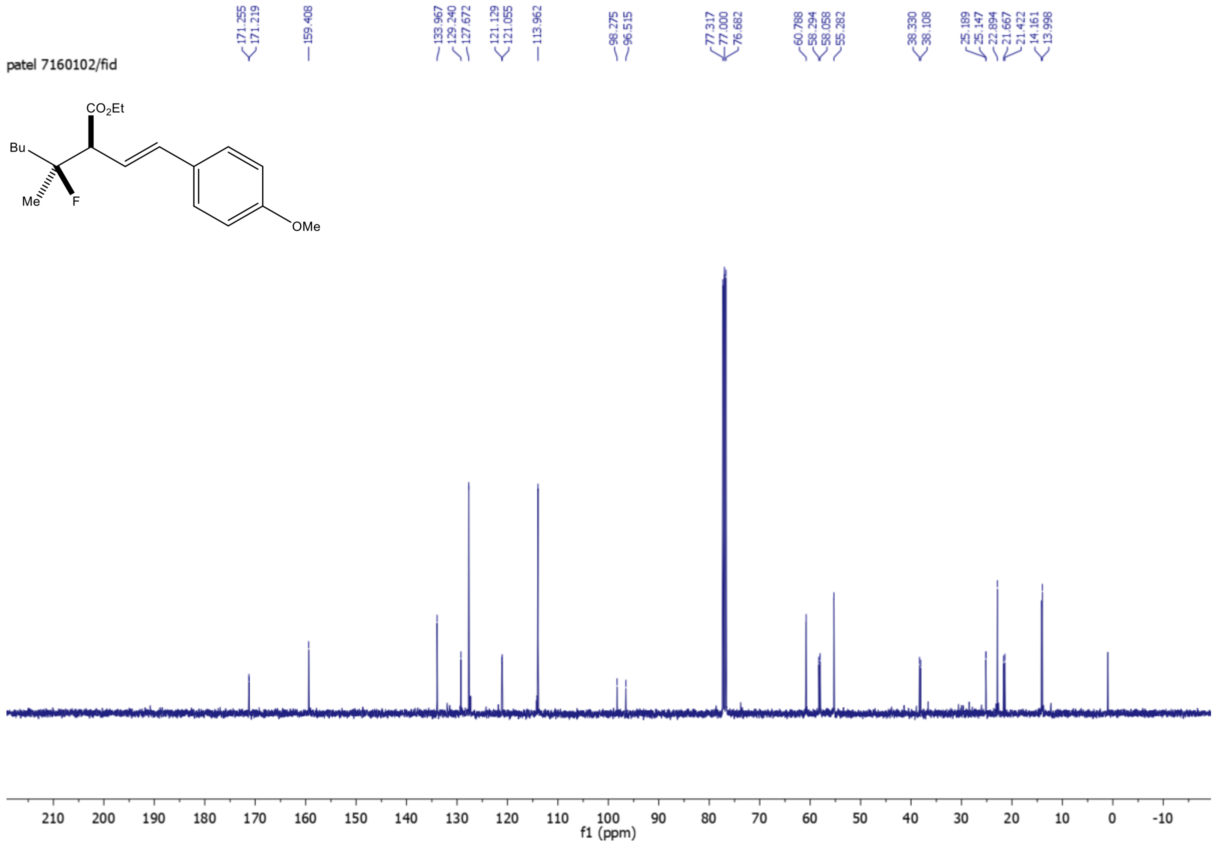
patel 7160102/fid
CO2Et
Bu
Me
F
OMe
171.255 171.219 159.408 133.967 129.240 127.672 121.129 121.055 113.962 98.275 96.515 77.317 77.000 76.682 60.788 58.294 58.058 55.282 38.330 38.108 25.189 25.147 22.894 21.667 21.422 14.161 13.998
f1 (ppm)

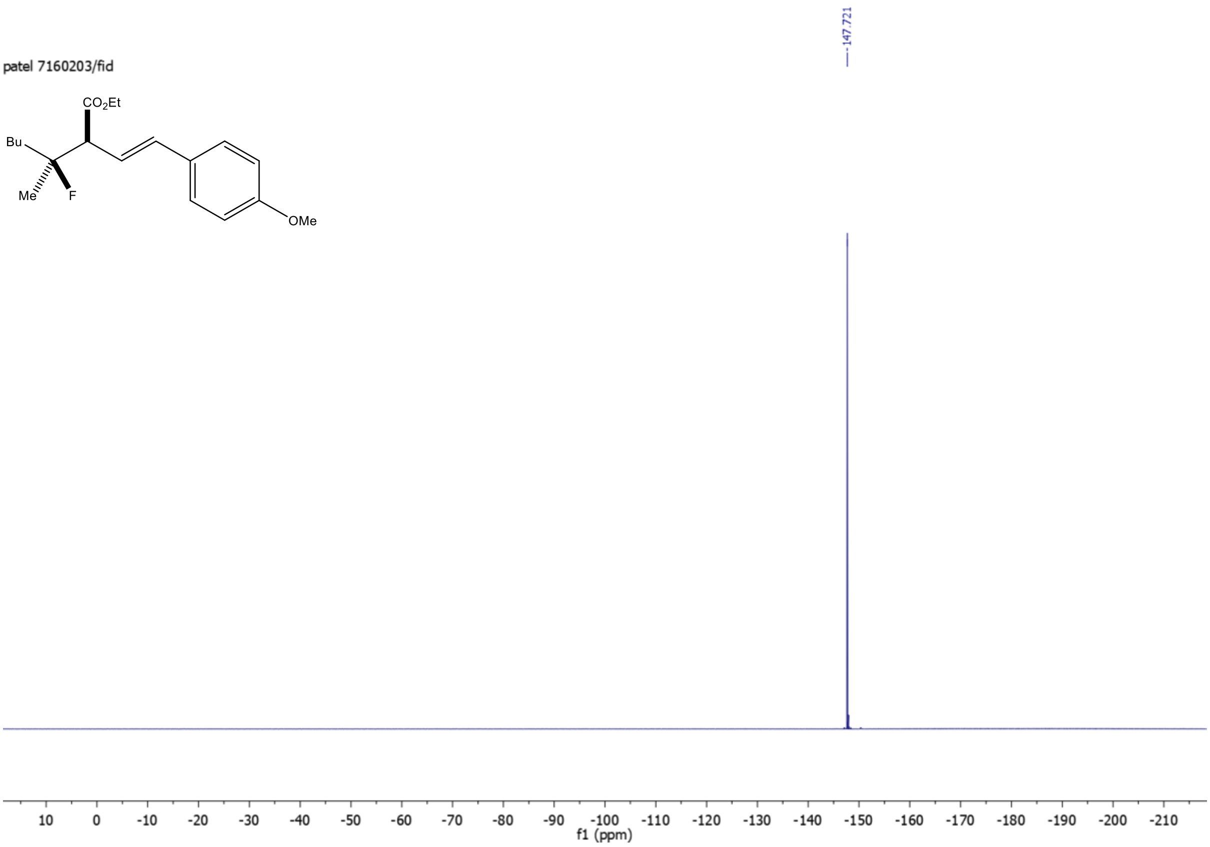
patel 7160203/fid
-147.721
CO2Et
Bu
Me
F
OMe
10 0 -10 -20 -30 -40 -50 -60 -70 -80 -90 -100 -110 -120 -130 -140 -150 -160 -170 -180 -190 -200 -210
f1 (ppm)

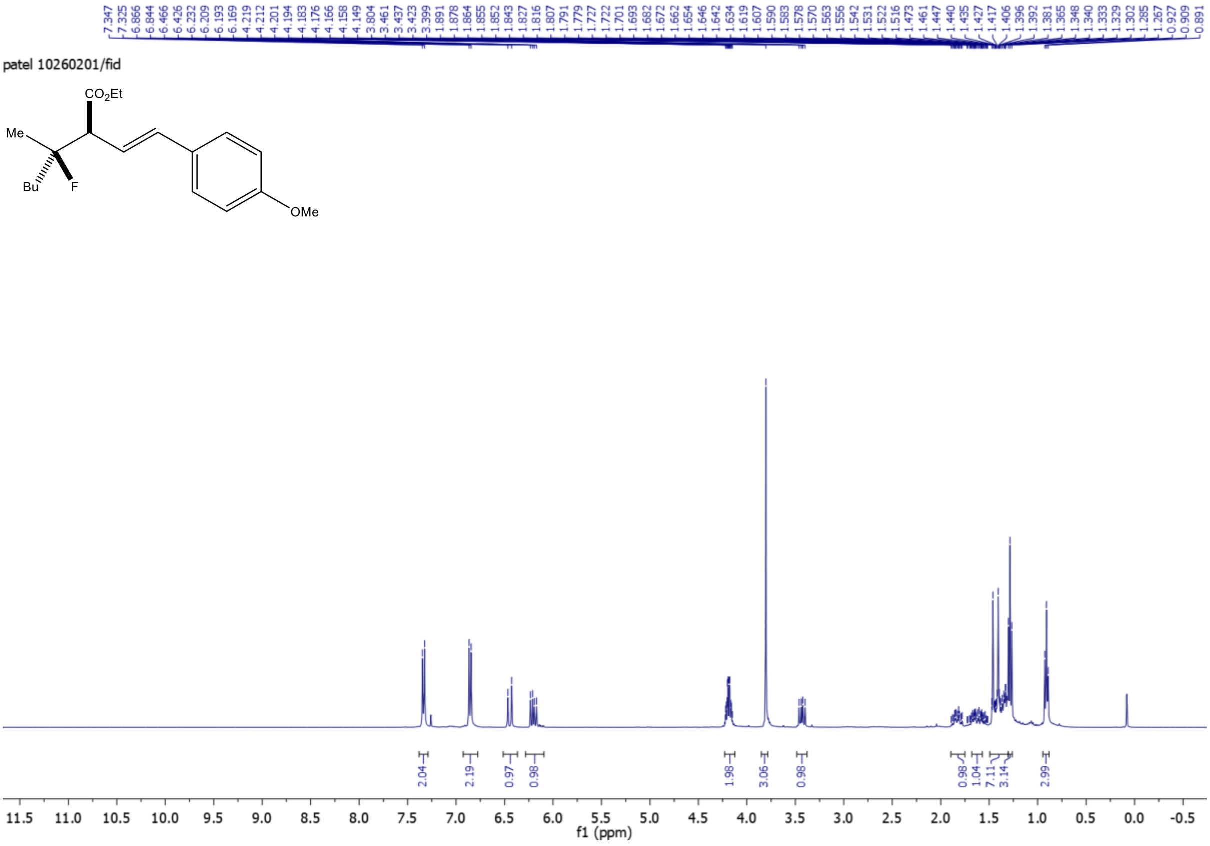
7.347 7.325 6.866 6.844 6.466 6.426 6.232 6.209 6.193 6.169 4.219 4.212 4.201 4.194 4.183 4.176 4.166 4.158 4.149 3.804 3.461 3.437 3.423 3.399 1.891 1.878 1.864 1.855 1.852 1.843 1.827 1.816 1.807 1.791 1.779 1.727 1.722 1.701 1.693 1.682 1.672 1.662 1.654 1.646 1.642 1.634 1.619 1.607 1.590 1.583 1.578 1.570 1.563 1.556 1.542 1.531 1.522 1.516 1.473 1.461 1.447 1.440 1.435 1.427 1.417 1.406 1.396 1.392 1.381 1.365 1.348 1.340 1.333 1.329 1.302 1.285 1.267 0.927 0.909 0.891
patel 10260201/fid
CO2Et
Me
Bu
F
OMe
2.04 2.19 0.97 0.98 1.98 3.06 0.98 0.98 1.04 7.11 3.14 2.99
11.5 11.0 10.5 10.0 9.5 9.0 8.5 8.0 7.5 7.0 6.5 6.0 5.5 5.0 4.5 4.0 3.5 3.0 2.5 2.0 1.5 1.0 0.5 0.0 -0.5
f1 (ppm)

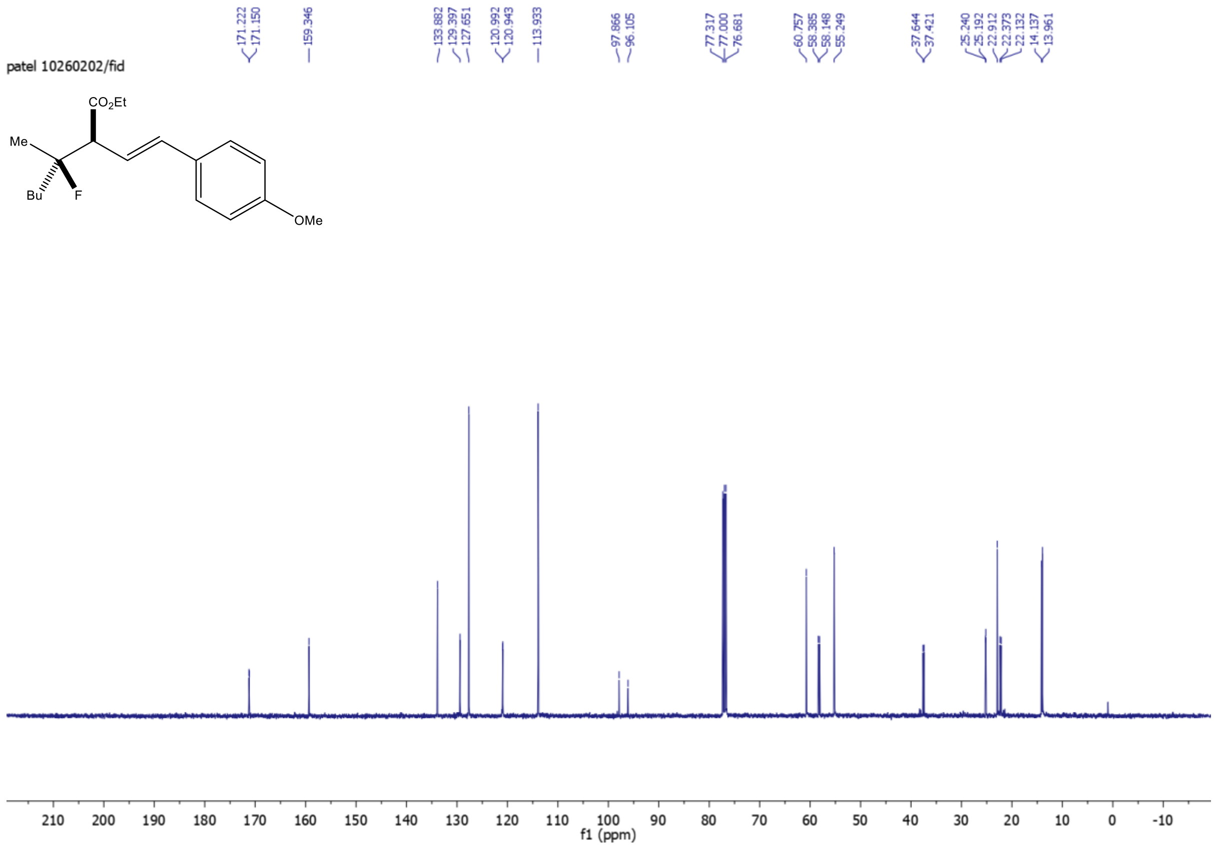

patel 10260202/fid
CO2Et
Me
Bu
F
OMe
171.222
171.150
159.346
133.882
129.397
127.651
120.992
120.943
113.933
97.866
96.105
77.317
77.000
76.681
60.757
58.385
58.148
55.249
37.644
37.421
25.240
25.192
22.912
22.373
22.132
14.137
13.961
f1 (ppm)

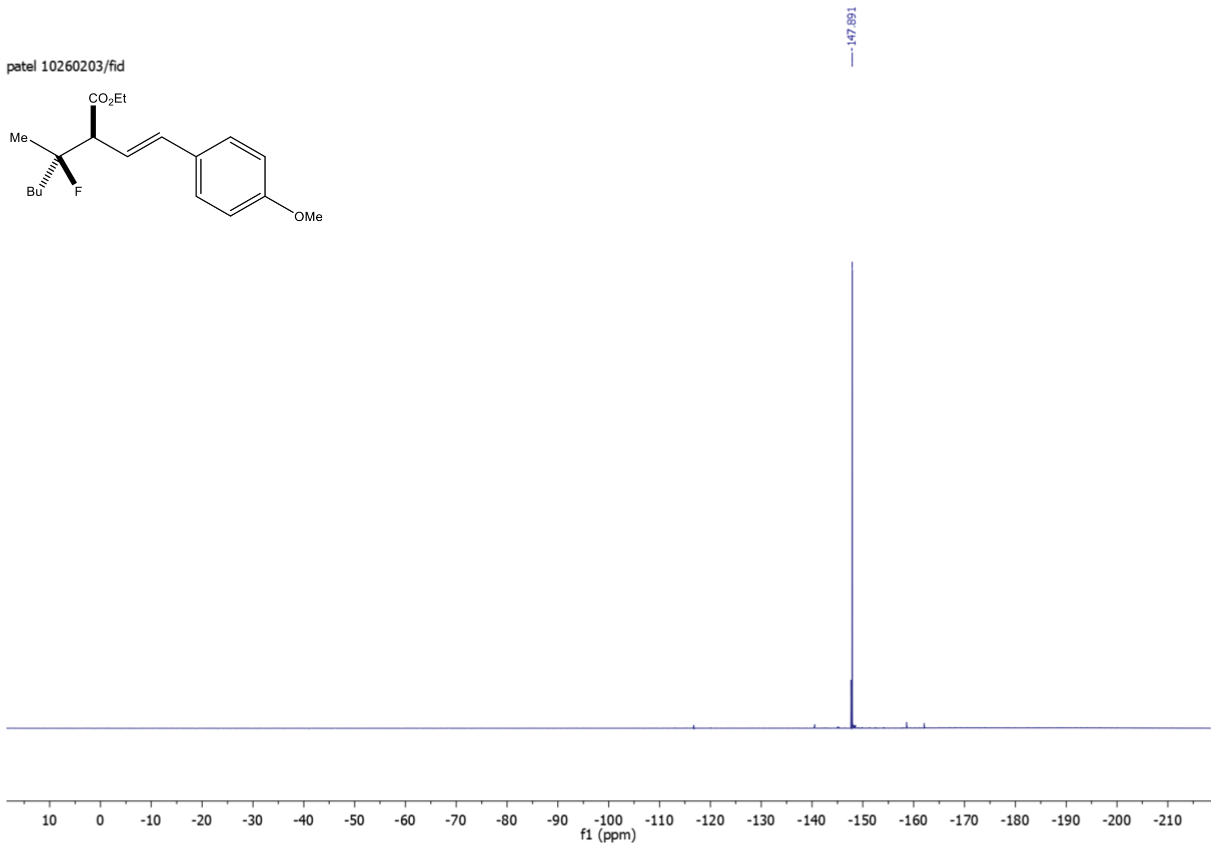

patel 10260203/fid
CO2Et
Me
Bu
F
OMe
-147.891
f1 (ppm)

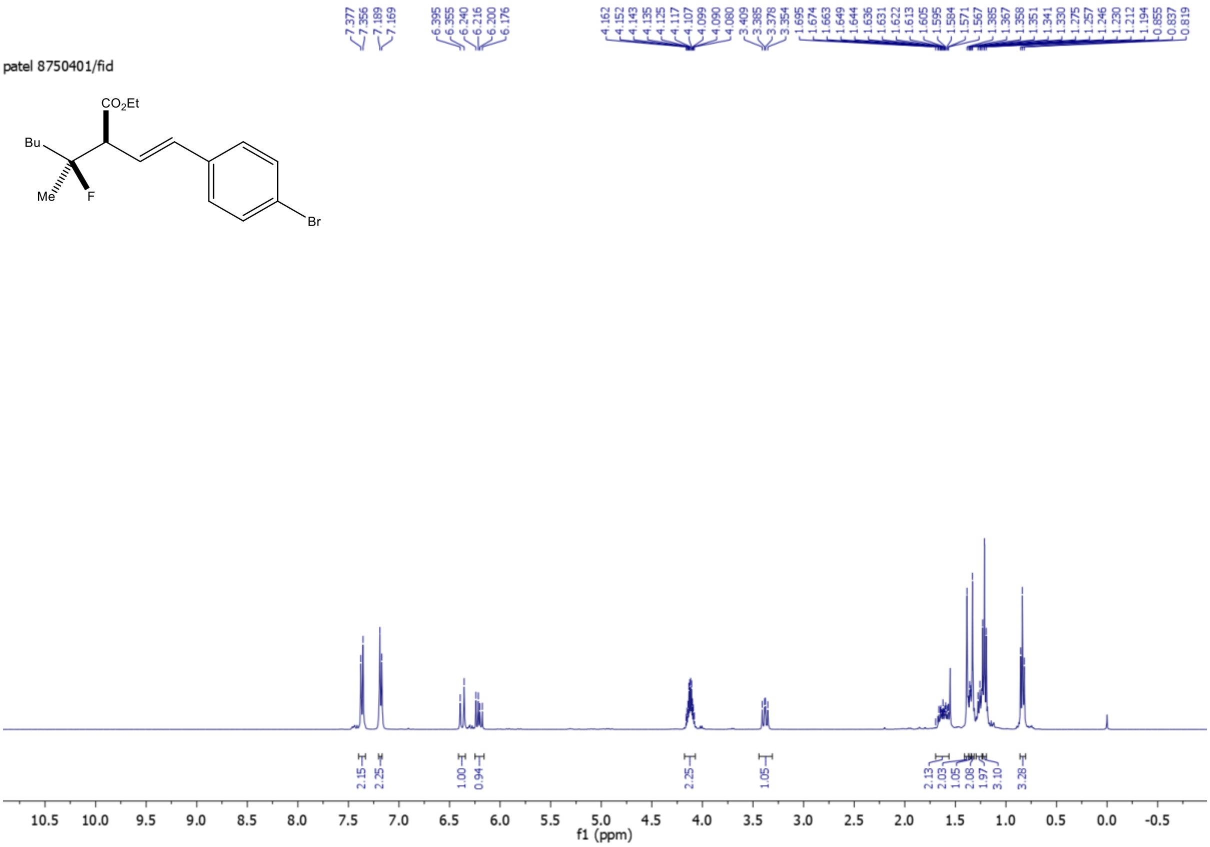
patel 8750401/fid
CO2Et
Bu
Me
F
Br
7.377 7.356 7.189 7.169
6.395 6.355 6.240 6.216 6.200 6.176
4.162 4.152 4.143 4.135 4.125 4.117 4.107 4.099 4.090 4.080 3.409 3.385 3.378 3.354 1.695 1.674 1.663 1.649 1.644 1.636 1.631 1.622 1.613 1.605 1.595 1.584 1.571 1.567 1.385 1.367 1.358 1.351 1.341 1.330 1.275 1.257 1.246 1.230 1.212 1.194 0.855 0.837 0.819
2.15 2.25 1.00 0.94 2.25 1.05 2.13 2.03 1.05 2.08 1.97 3.10 3.28
10.5 10.0 9.5 9.0 8.5 8.0 7.5 7.0 6.5 6.0 5.5 5.0 4.5 4.0 3.5 3.0 2.5 2.0 1.5 1.0 0.5 0.0 -0.5
f1 (ppm)

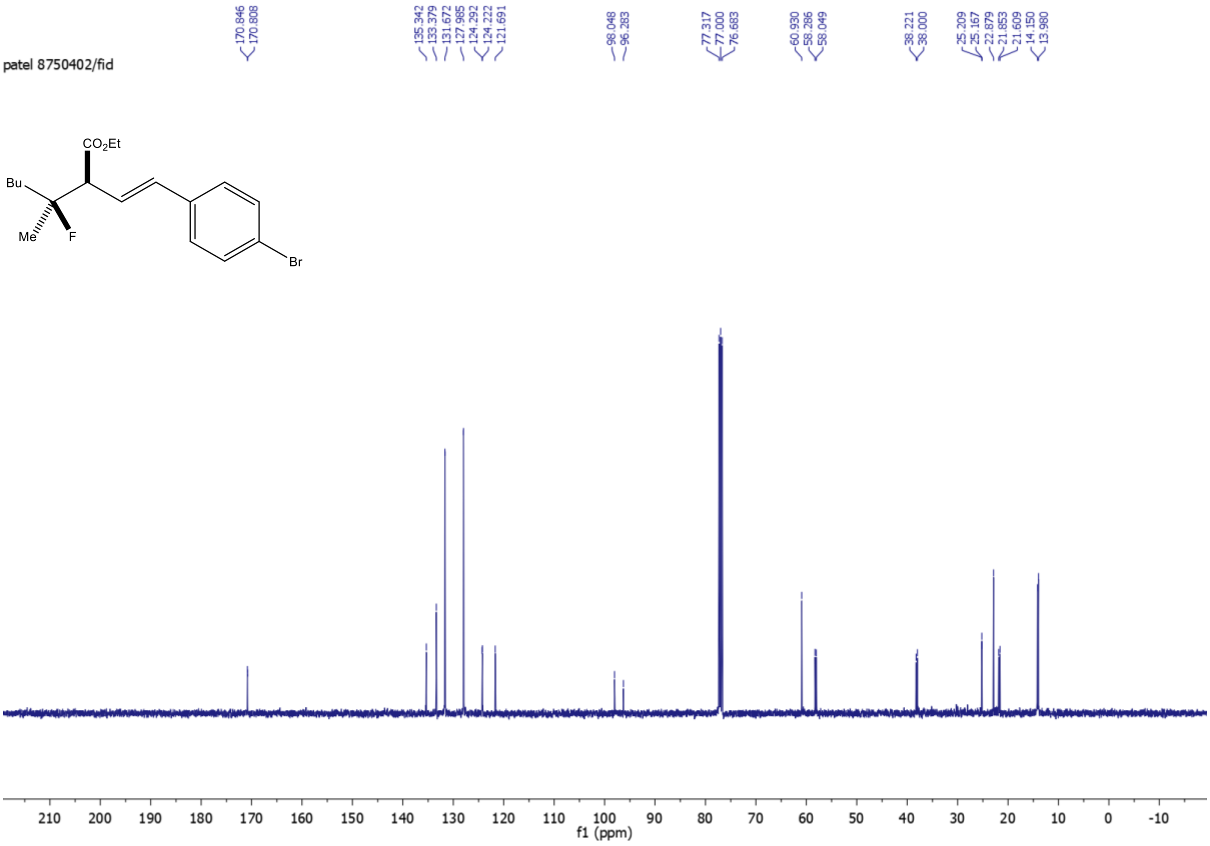
patel 8750402/fid
CO2Et
Bu
Me
F
Br
170.846 170.808
135.342 133.379 131.672 127.985 124.292 124.222 121.691
98.048 96.283
77.317 77.000 76.683
60.930 58.286 58.049
38.221 38.000
25.209 25.167 22.879 21.853 21.609 14.150 13.980
210 200 190 180 170 160 150 140 130 120 110 100 90 80 70 60 50 40 30 20 10 0 -10
f1 (ppm)

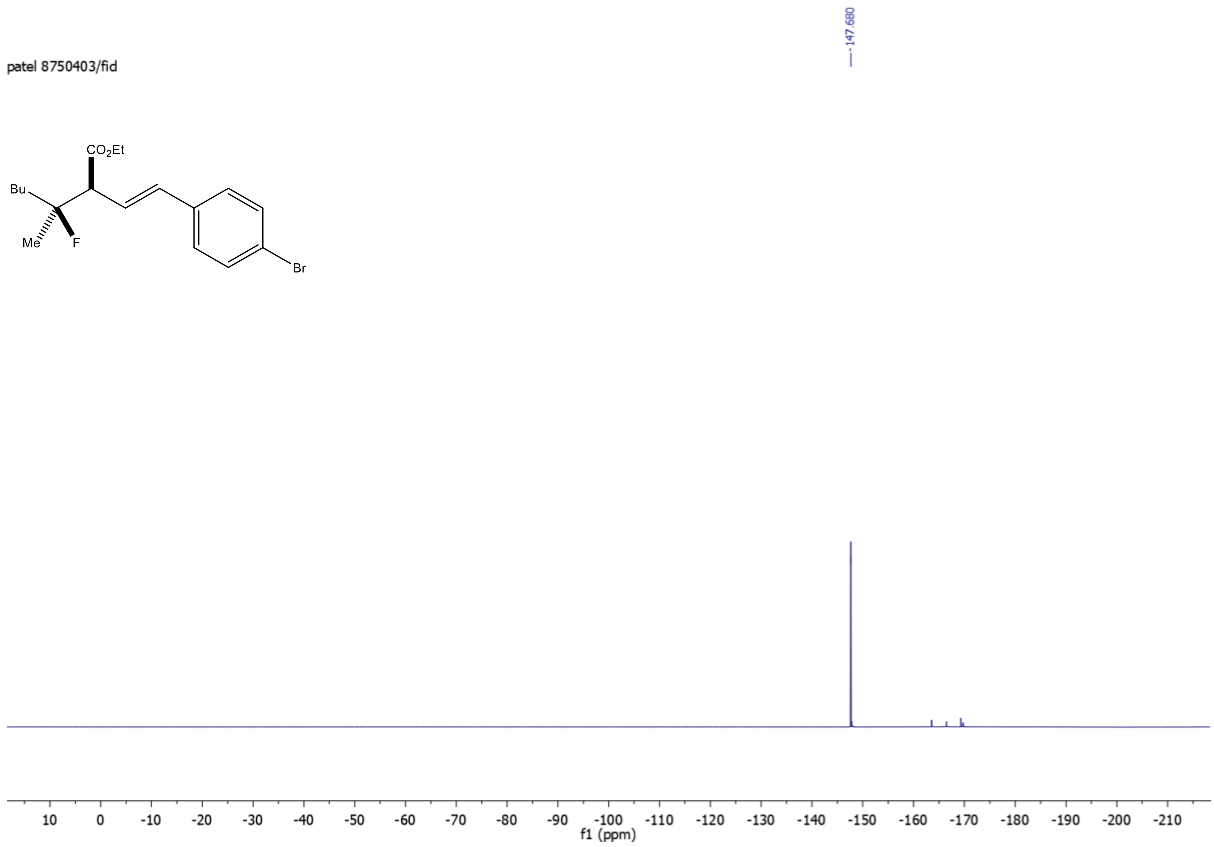
-147.680
patel 8750403/fid
CO2Et
Bu
Me
F
Br
10 0 -10 -20 -30 -40 -50 -60 -70 -80 -90 -100 -110 -120 -130 -140 -150 -160 -170 -180 -190 -200 -210
f1 (ppm)

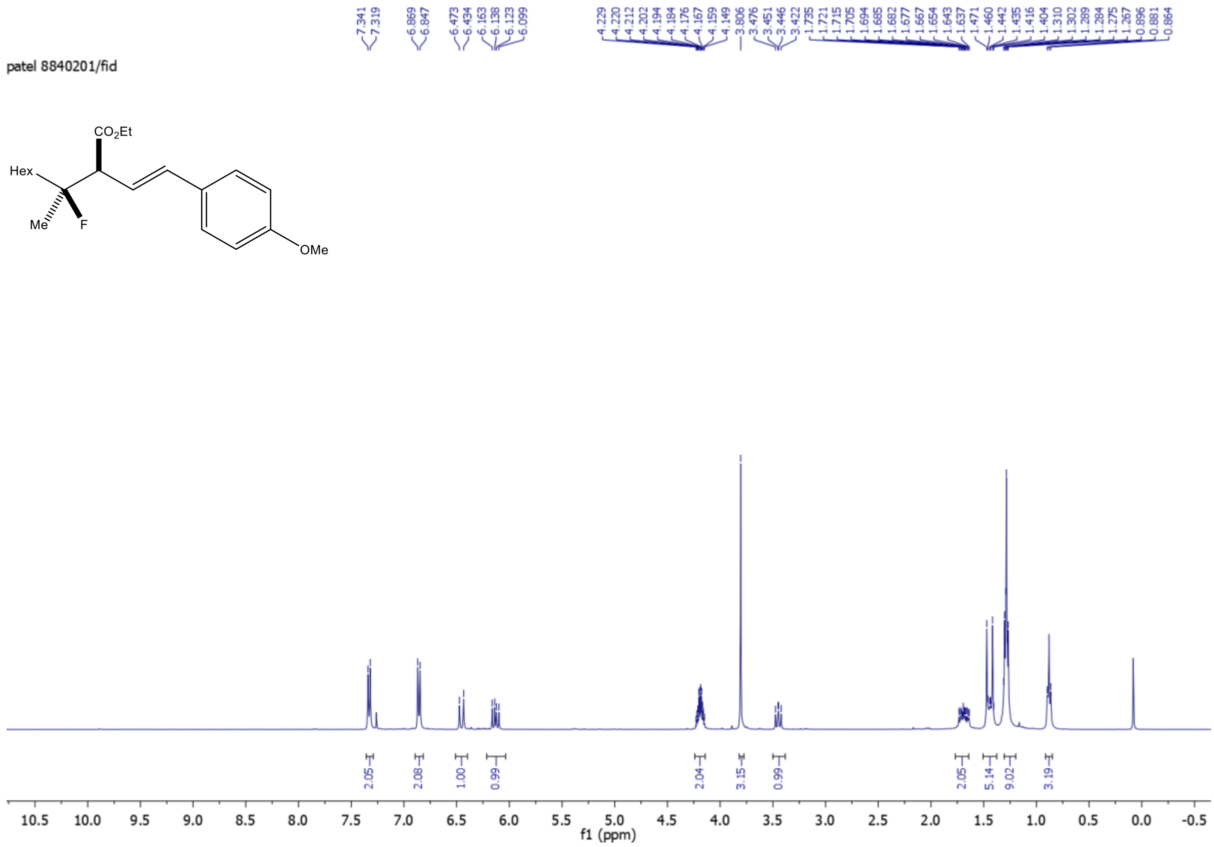
7.341 7.319 6.869 6.847 6.473 6.434 6.163 6.138 6.123 6.099 4.229 4.220 4.212 4.202 4.194 4.184 4.176 4.167 4.159 4.149 3.806 3.476 3.451 3.446 3.422 1.735 1.721 1.715 1.705 1.694 1.685 1.682 1.677 1.667 1.654 1.643 1.637 1.471 1.460 1.442 1.435 1.416 1.404 1.310 1.302 1.289 1.284 1.275 1.267 0.896 0.881 0.864
patel 8840201/fid
CO2Et
Hex
Me
F
OMe
2.05 2.08 1.00 0.99 2.04 3.15 0.99 2.05 5.14 9.02 3.19
10.5 10.0 9.5 9.0 8.5 8.0 7.5 7.0 6.5 6.0 5.5 5.0 4.5 4.0 3.5 3.0 2.5 2.0 1.5 1.0 0.5 0.0 -0.5
f1 (ppm)

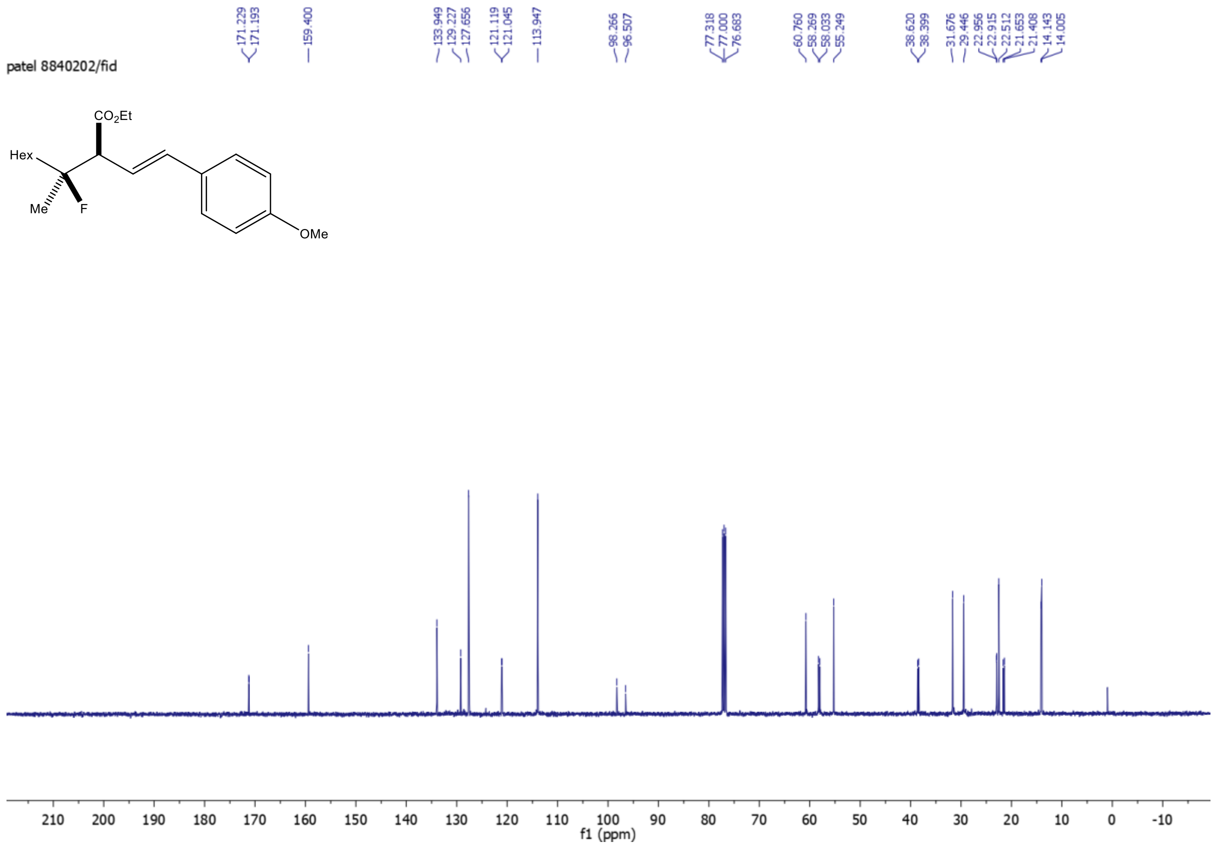
patel 8840202/fid
171.229
171.193
159.400
133.949
129.227
127.656
121.119
121.045
113.947
98.266
96.507
77.318
77.000
76.683
60.760
58.269
58.033
55.249
38.620
38.399
31.676
29.446
22.956
22.915
22.512
21.653
21.408
14.143
14.005
CO_2Et
Hex
Me
F
OMe
210 200 190 180 170 160 150 140 130 120 110 100 90 80 70 60 50 40 30 20 10 0 -10
f1 (ppm)

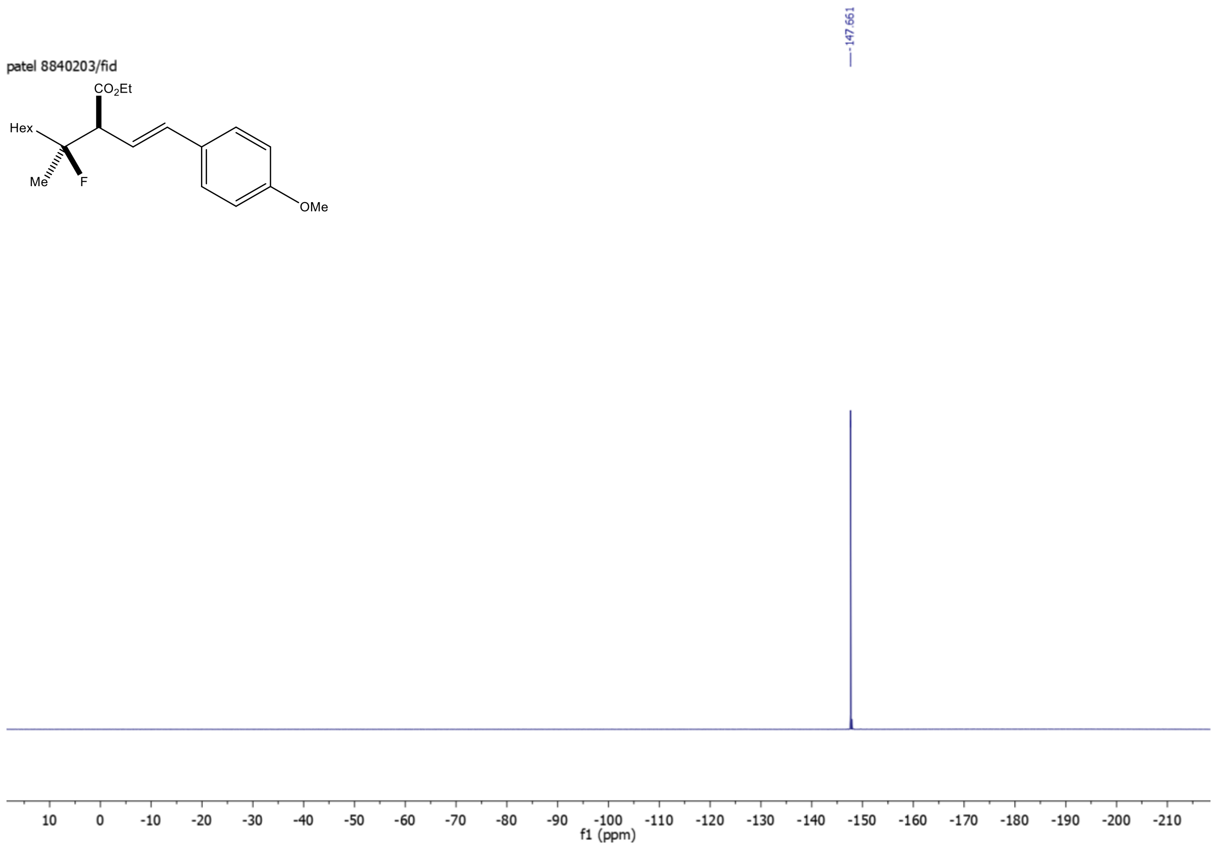
patel 8840203/fid
-147.661
CO_2Et
Hex
Me
F
OMe
10 0 -10 -20 -30 -40 -50 -60 -70 -80 -90 -100 -110 -120 -130 -140 -150 -160 -170 -180 -190 -200 -210
f1 (ppm)

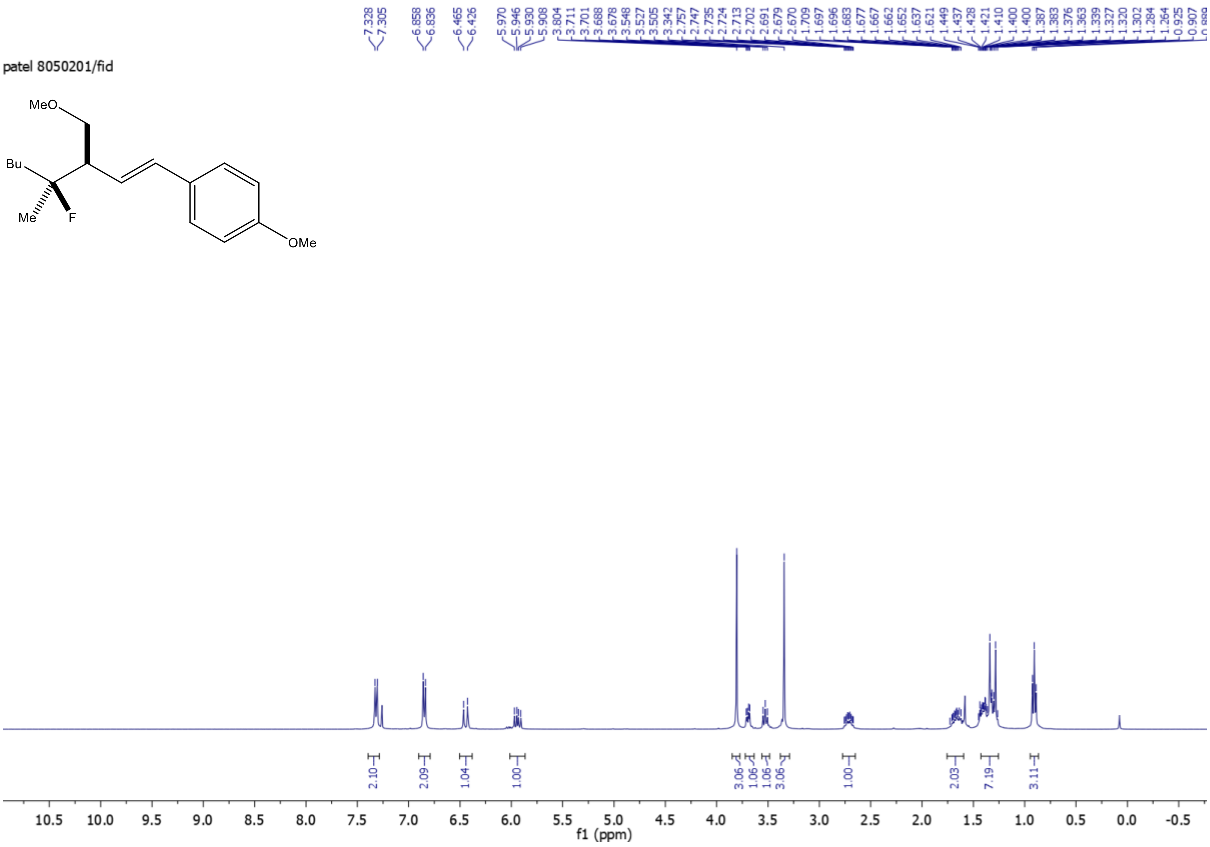
7.328
7.305
6.858
6.836
6.465
6.426
5.970
5.946
5.930
5.908
3.804
3.711
3.701
3.688
3.678
3.548
3.527
3.505
3.342
2.757
2.747
2.735
2.724
2.713
2.702
2.691
2.679
2.670
1.709
1.697
1.696
1.683
1.677
1.667
1.662
1.652
1.637
1.621
1.449
1.437
1.428
1.421
1.410
1.400
1.387
1.383
1.376
1.363
1.339
1.327
1.320
1.302
1.284
1.264
0.925
0.907
[illegible]
patel 8050201/fid
MeO
Bu
Me
F
OMe
2.10
2.09
1.04
1.00
3.06
1.06
1.06
3.06
1.00
2.03
7.19
3.11
10.5 10.0 9.5 9.0 8.5 8.0 7.5 7.0 6.5 6.0 5.5 5.0 4.5 4.0 3.5 3.0 2.5 2.0 1.5 1.0 0.5 0.0 -0.5
f1 (ppm)

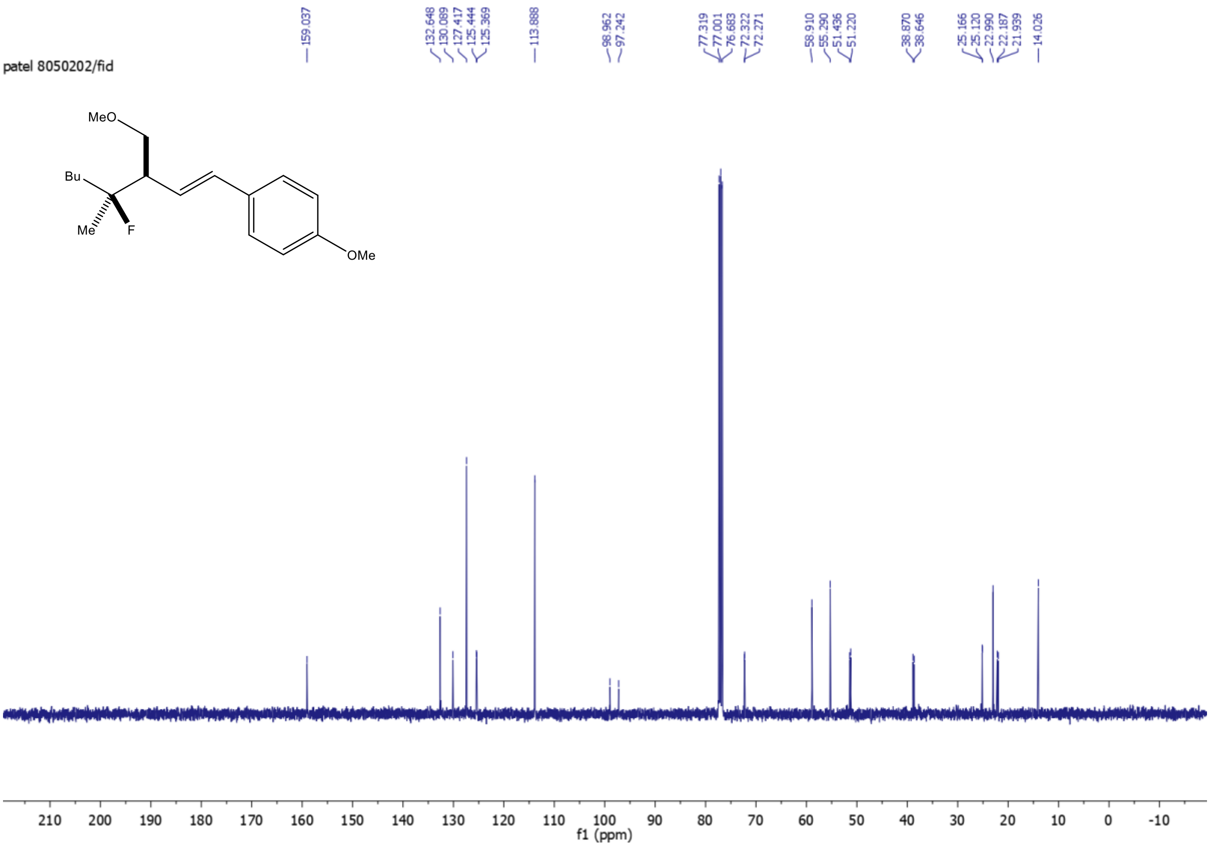
159.037
132.648
130.089
127.417
125.444
125.369
113.888
98.962
97.242
77.319
77.001
76.683
72.322
72.271
58.910
55.290
51.436
51.220
38.870
38.646
25.166
25.120
22.990
22.187
21.939
14.026
patel 8050202/fid
MeO
Bu
Me
F
OMe
210 200 190 180 170 160 150 140 130 120 110 100 90 80 70 60 50 40 30 20 10 0 -10
f1 (ppm)

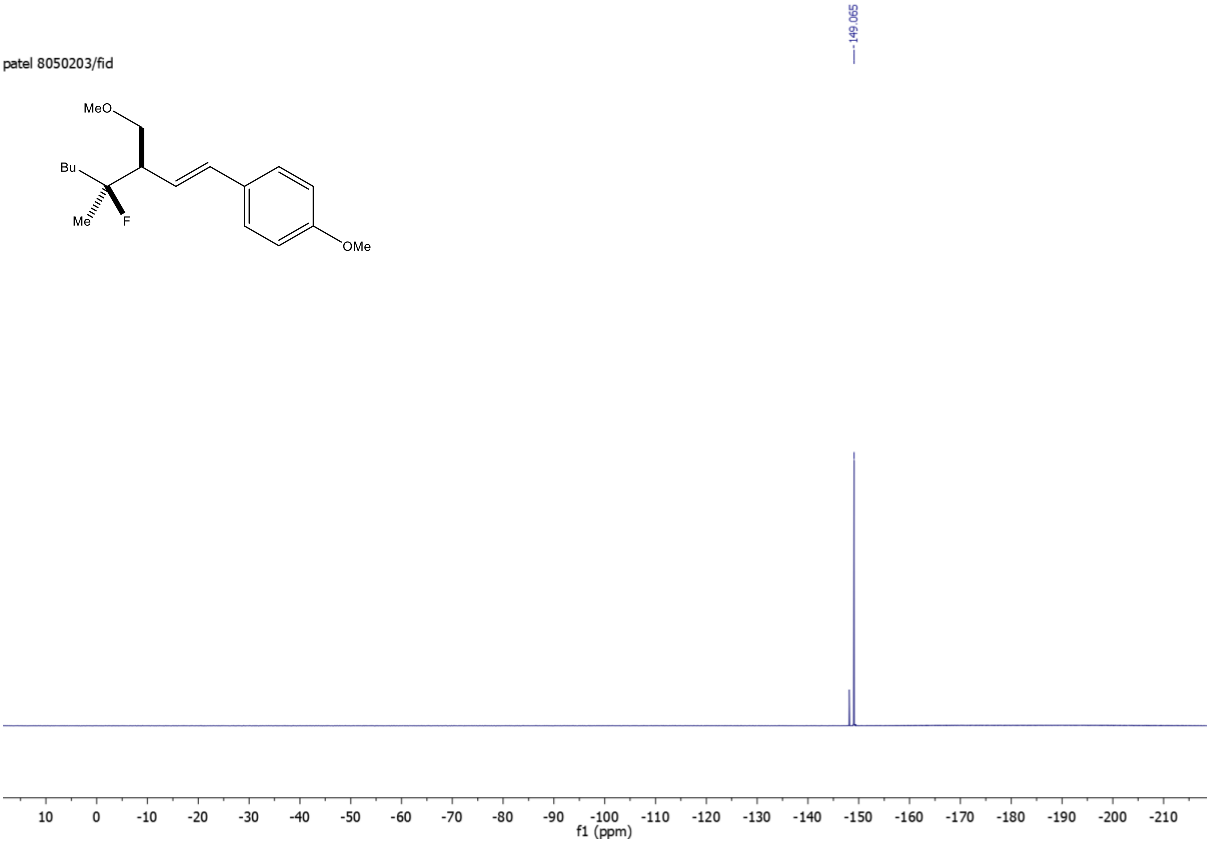
-149.065
patel 8050203/fid
MeO
Bu
Me
F
OMe
10 0 -10 -20 -30 -40 -50 -60 -70 -80 -90 -100 -110 -120 -130 -140 -150 -160 -170 -180 -190 -200 -210
f1 (ppm)

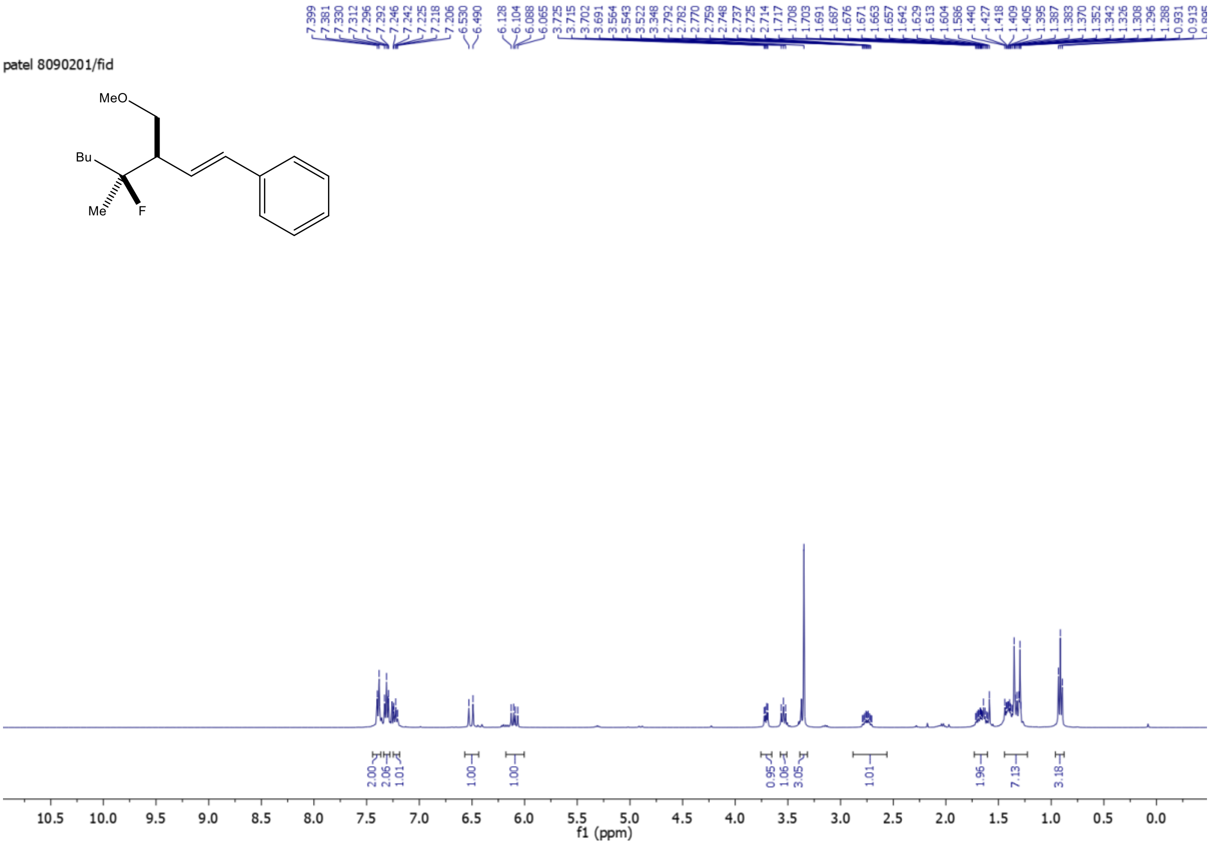
7.399 7.381 7.330 7.312 7.296 7.292 7.246 7.242 7.225 7.218 7.206 6.530 6.490 6.128 6.104 6.088 6.065 3.725 3.715 3.702 3.691 3.564 3.543 3.522 3.348 2.792 2.782 2.770 2.759 2.748 2.737 2.725 2.714 1.717 1.708 1.703 1.691 1.687 1.676 1.671 1.663 1.657 1.642 1.629 1.613 1.604 1.586 1.440 1.427 1.418 1.409 1.405 1.395 1.387 1.383 1.370 1.352 1.342 1.326 1.308 1.296 1.288 0.931 0.913
patel 8090201/fid
MeO
Bu
Me
F
2.00 2.06 1.01 1.00 1.00 0.95 1.06 3.05 1.01 1.96 7.13 3.18
10.5 10.0 9.5 9.0 8.5 8.0 7.5 7.0 6.5 6.0 5.5 5.0 4.5 4.0 3.5 3.0 2.5 2.0 1.5 1.0 0.5 0.0
f1 (ppm)

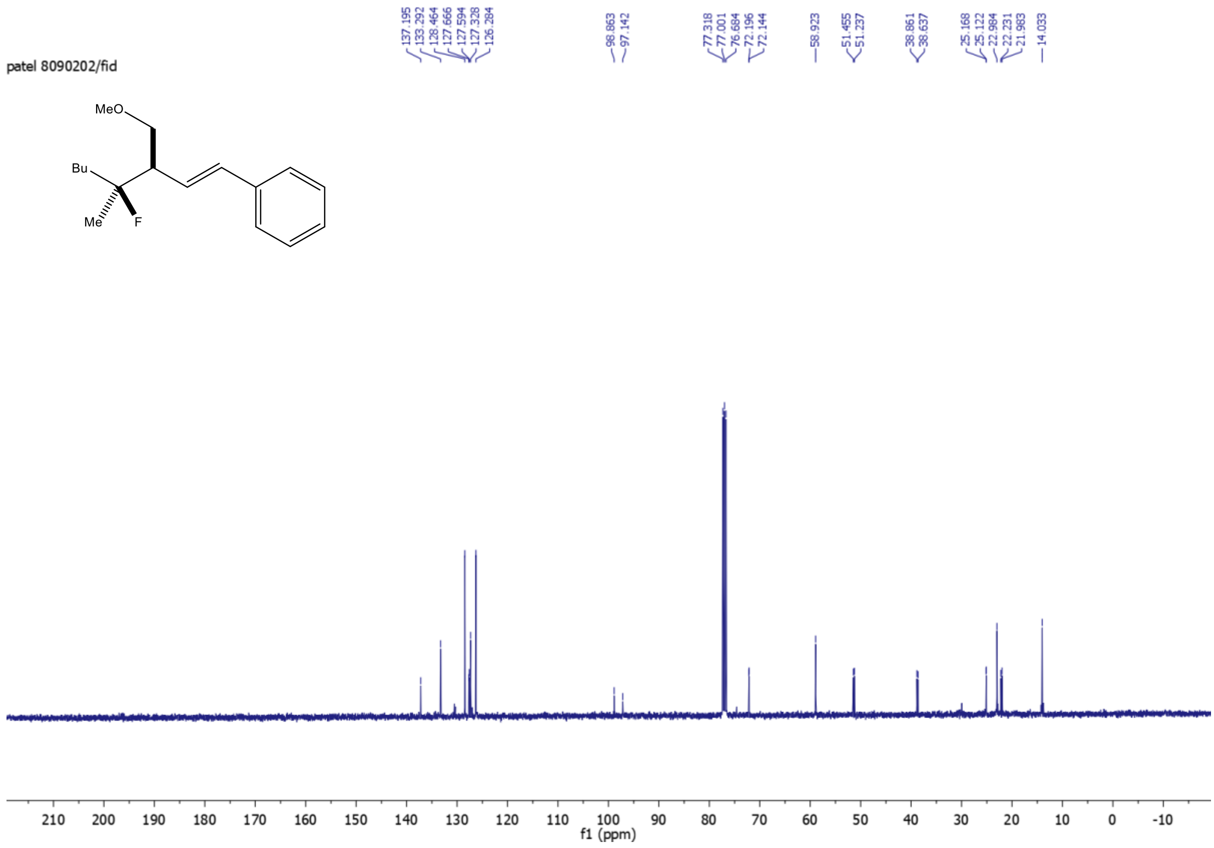
patel 8090202/fid
MeO
Bu
Me
F
137.195
133.292
128.464
127.666
127.594
127.328
126.284
98.863
97.142
77.318
77.001
76.684
72.196
72.144
58.923
51.455
51.237
38.861
38.637
25.168
25.122
22.984
22.231
21.983
14.033
f1 (ppm)

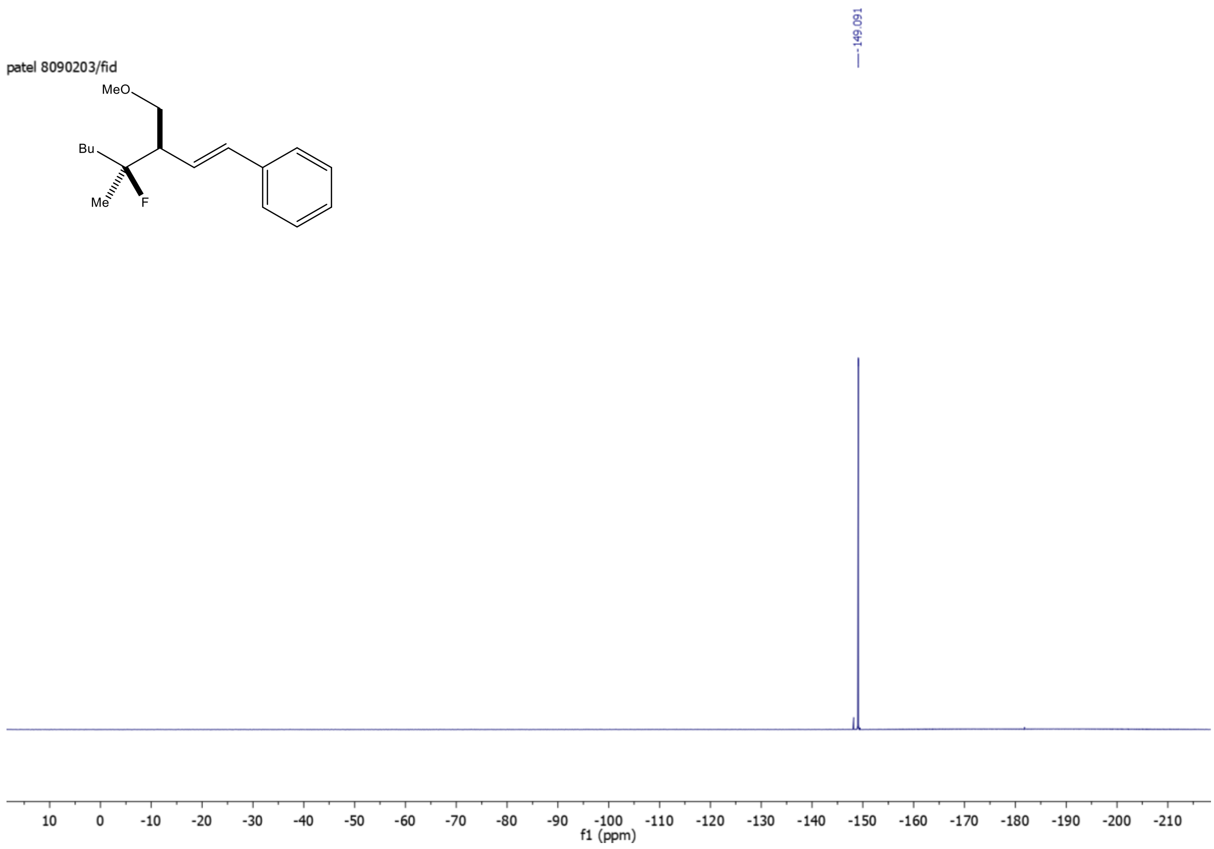
patel 8090203/fid
MeO
Bu
Me
F
-149.091
f1 (ppm)

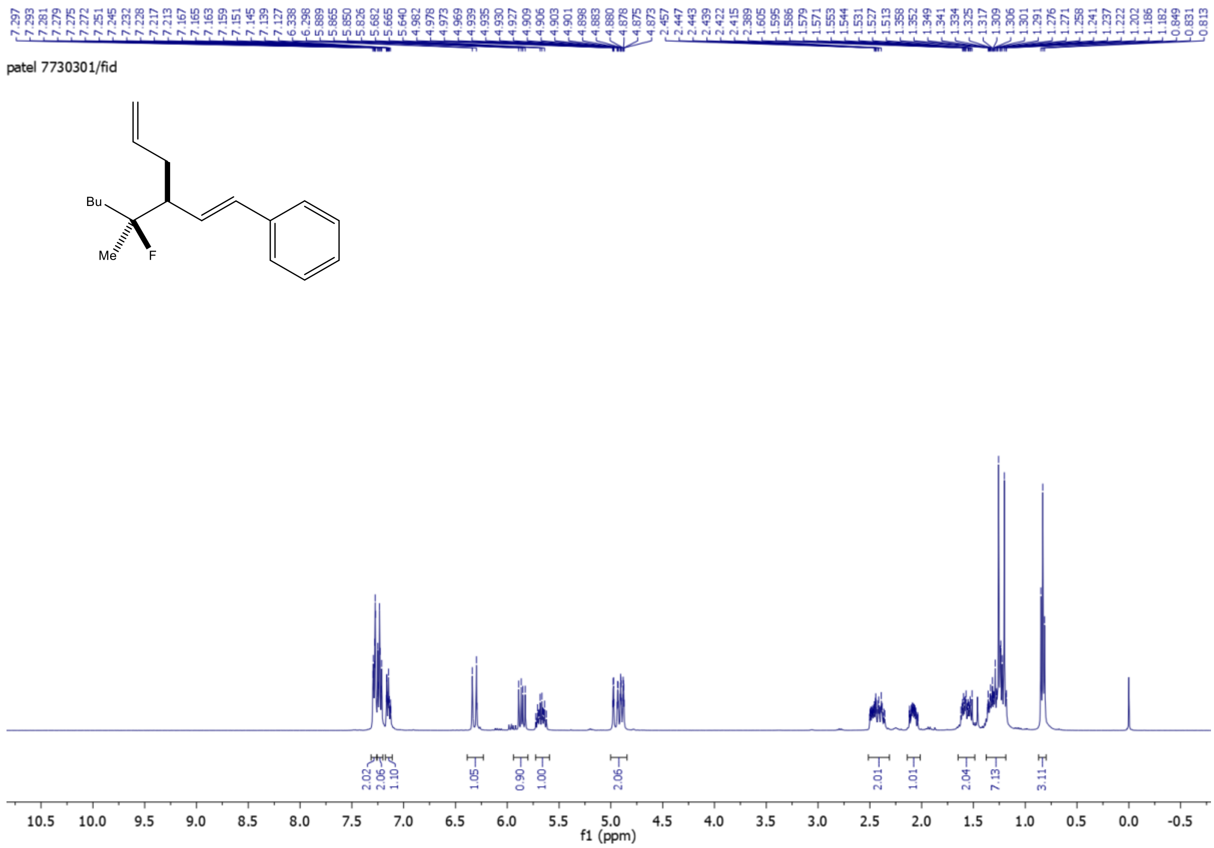
patel 7730301/fid
Bu
Me
F
f1 (ppm)

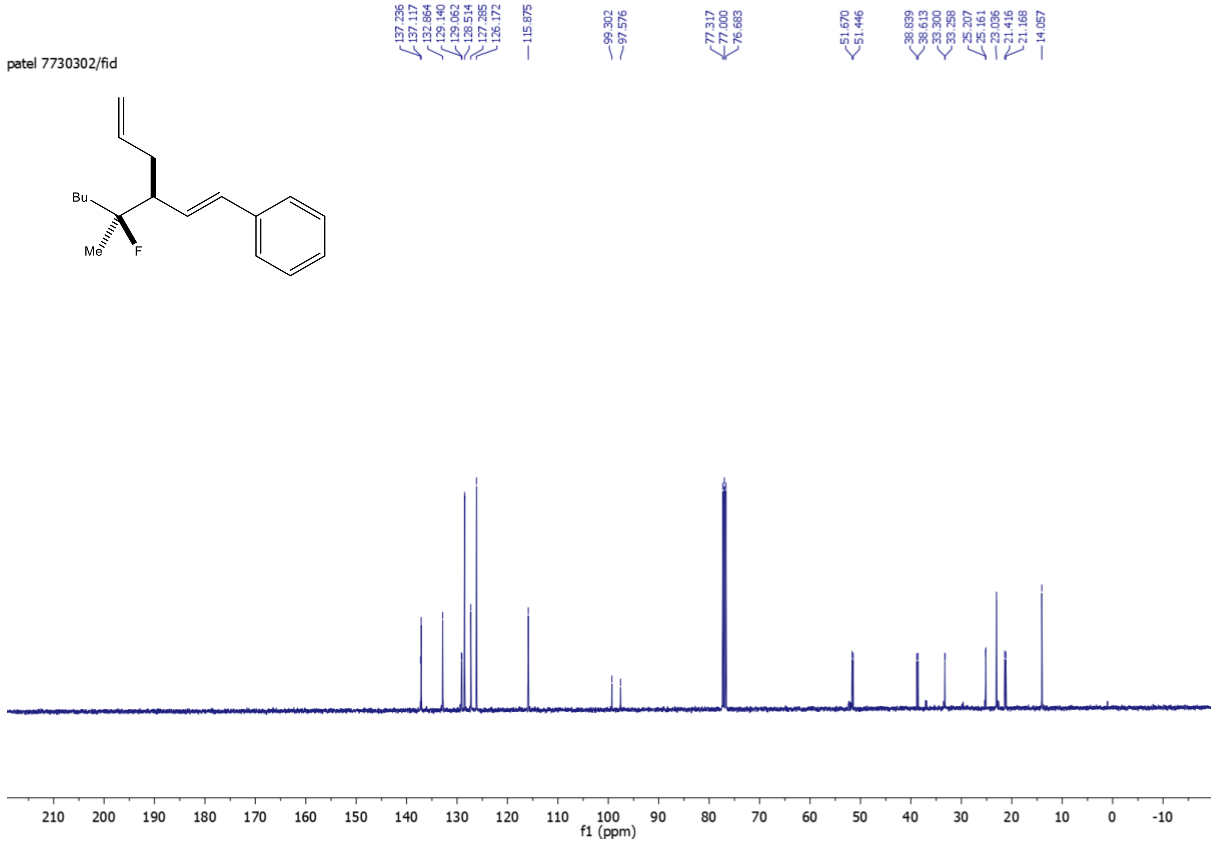
patel 7730302/fid
Bu
Me
F
f1 (ppm)

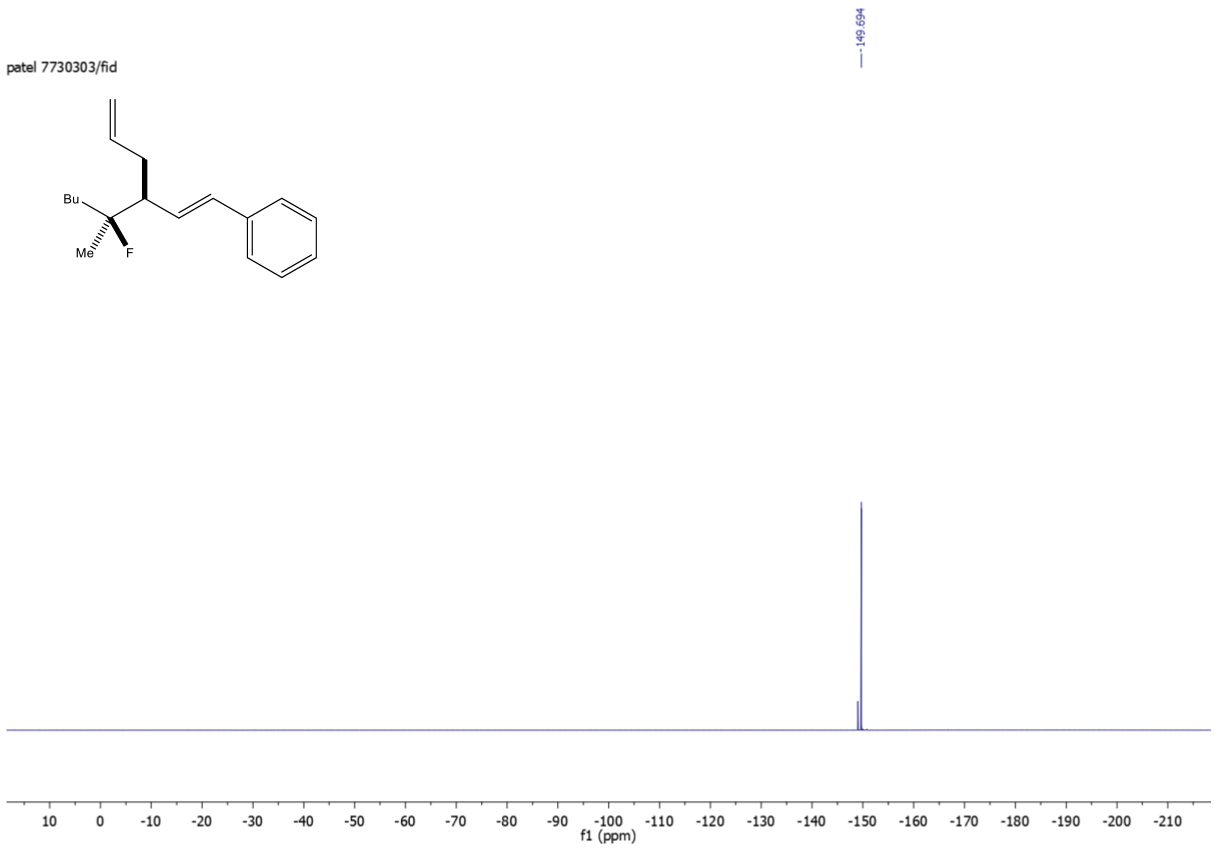

patel 7730303/fid
Bu
Me
F
-149.694
f1 (ppm)

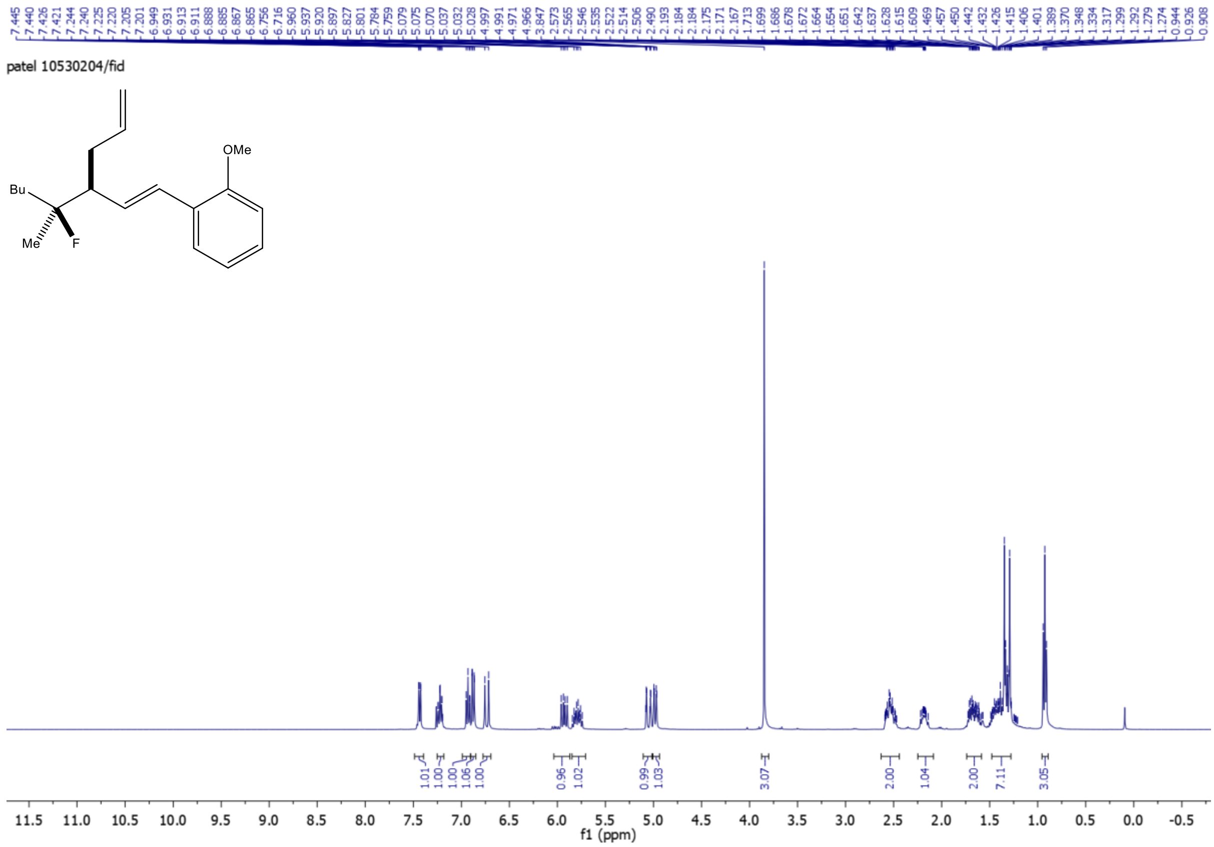

patel 10530204/fid
OMe
Bu
Me
F
f1 (ppm)

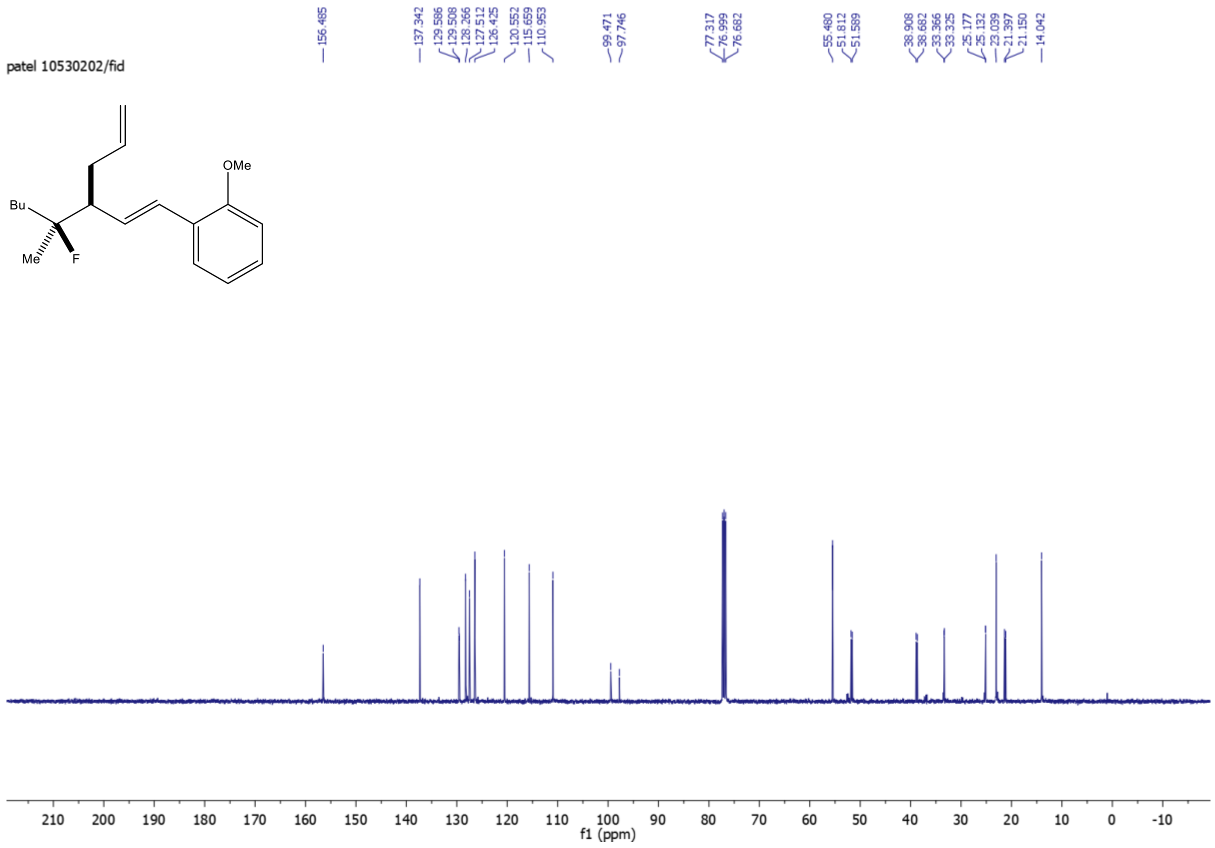
patel 10530202/fid
156.485
137.342
129.586
129.508
128.266
127.512
126.425
120.552
115.659
110.953
99.471
97.746
77.317
76.999
76.682
55.480
51.812
51.589
38.908
38.682
33.366
33.325
25.177
25.132
23.039
21.397
21.150
14.042
OMe
Bu
Me
F
210 200 190 180 170 160 150 140 130 120 110 100 90 80 70 60 50 40 30 20 10 0 -10
f1 (ppm)

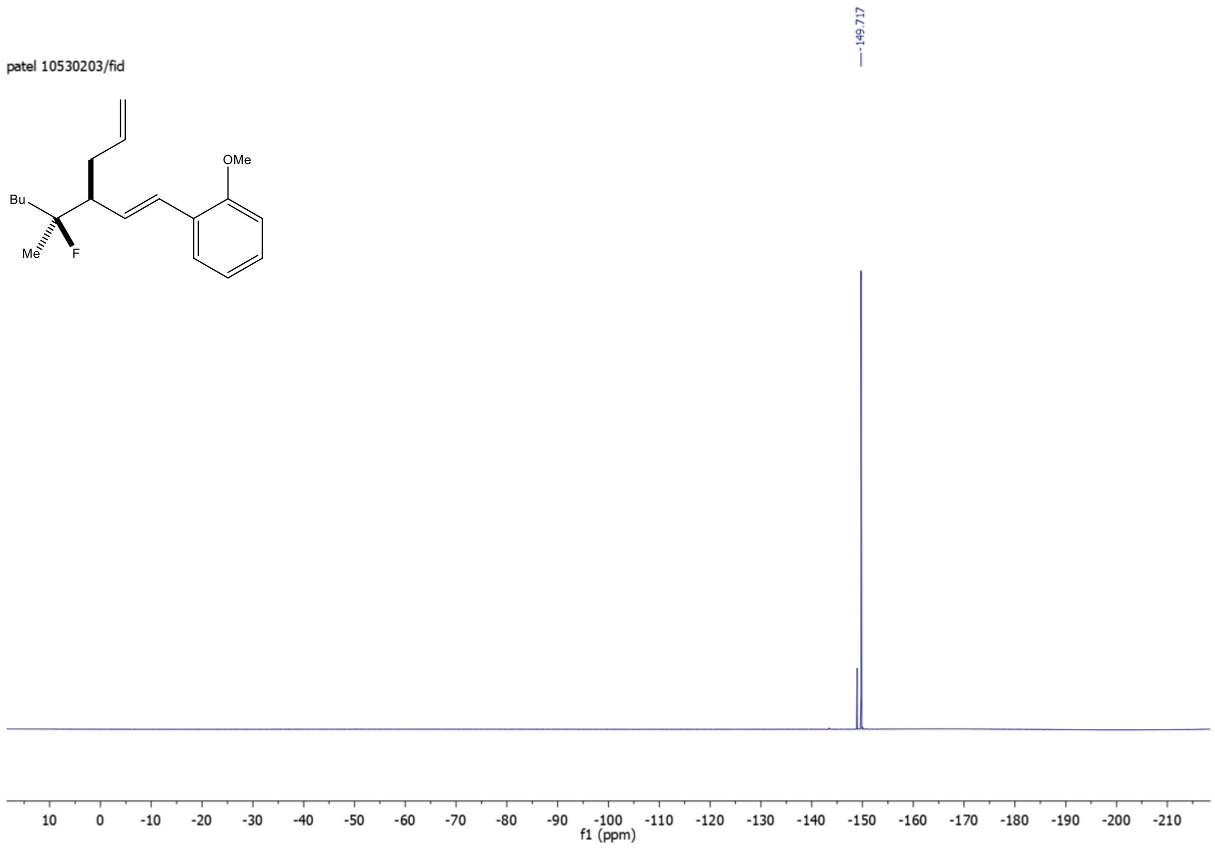
patel 10530203/fid
-149.717
OMe
Bu
Me
F
10 0 -10 -20 -30 -40 -50 -60 -70 -80 -90 -100 -110 -120 -130 -140 -150 -160 -170 -180 -190 -200 -210
f1 (ppm)

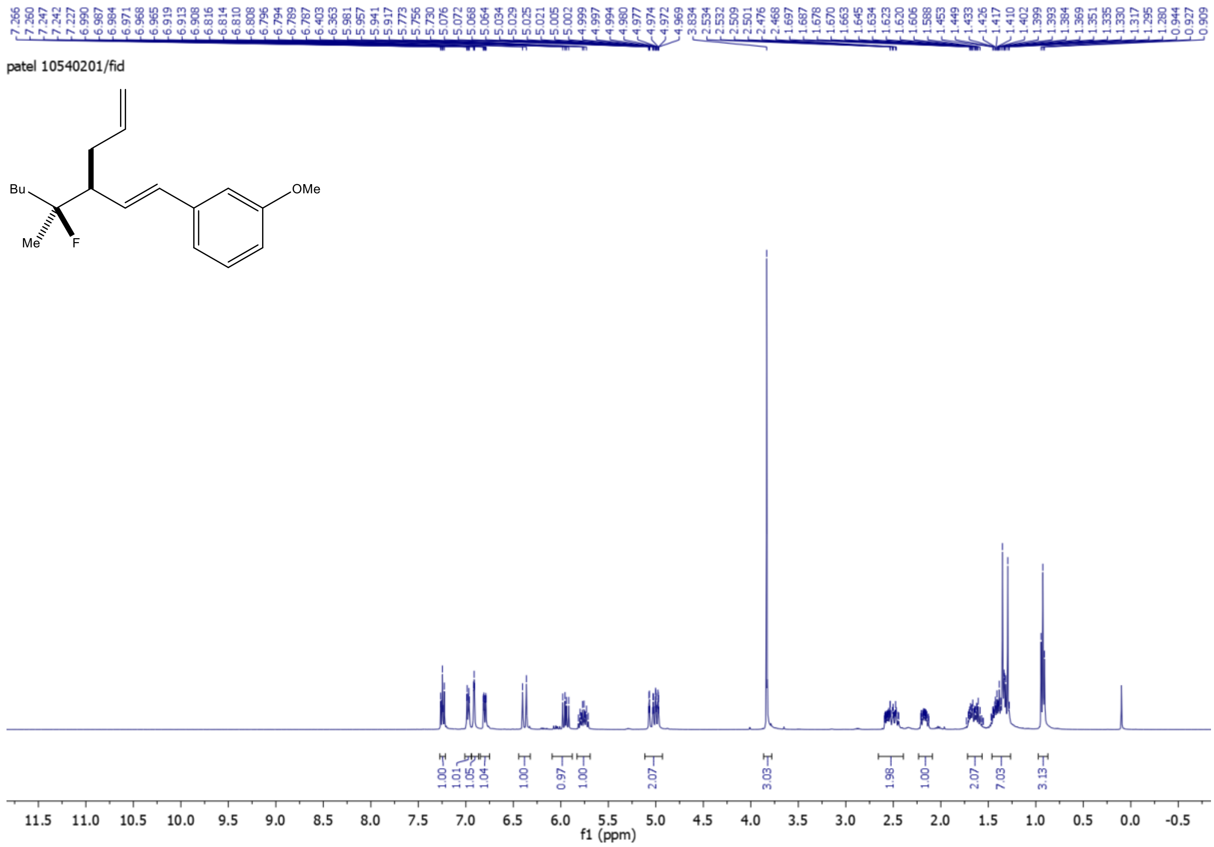
7.266 7.260 7.247 7.242 7.227 6.990 6.987 6.984 6.971 6.968 6.965 6.919 6.913 6.908 6.816 6.814 6.810 6.808 6.796 6.794 6.789 6.787 6.403 6.363 5.981 5.957 5.941 5.917 5.773 5.756 5.730 5.076 5.072 5.068 5.064 5.034 5.029 5.025 5.021 5.005 5.002 4.999 4.997 4.994 4.980 4.977 4.974 4.972 4.969 3.834 2.534 2.532 2.509 2.501 2.476 2.468 1.697 1.687 1.678 1.670 1.663 1.645 1.634 1.623 1.620 1.606 1.588 1.453 1.449 1.433 1.426 1.417 1.410 1.402 1.399 1.393 1.384 1.369 1.351 1.335 1.330 1.317 1.295 1.280 0.944 0.927 0.909
patel 10540201/fid
Bu
Me
F
OMe
1.00 1.01 1.05 1.04 1.00 0.97 1.00 2.07 3.03 1.98 1.00 2.07 7.03 3.13
11.5 11.0 10.5 10.0 9.5 9.0 8.5 8.0 7.5 7.0 6.5 6.0 5.5 5.0 4.5 4.0 3.5 3.0 2.5 2.0 1.5 1.0 0.5 0.0 -0.5
f1 (ppm)

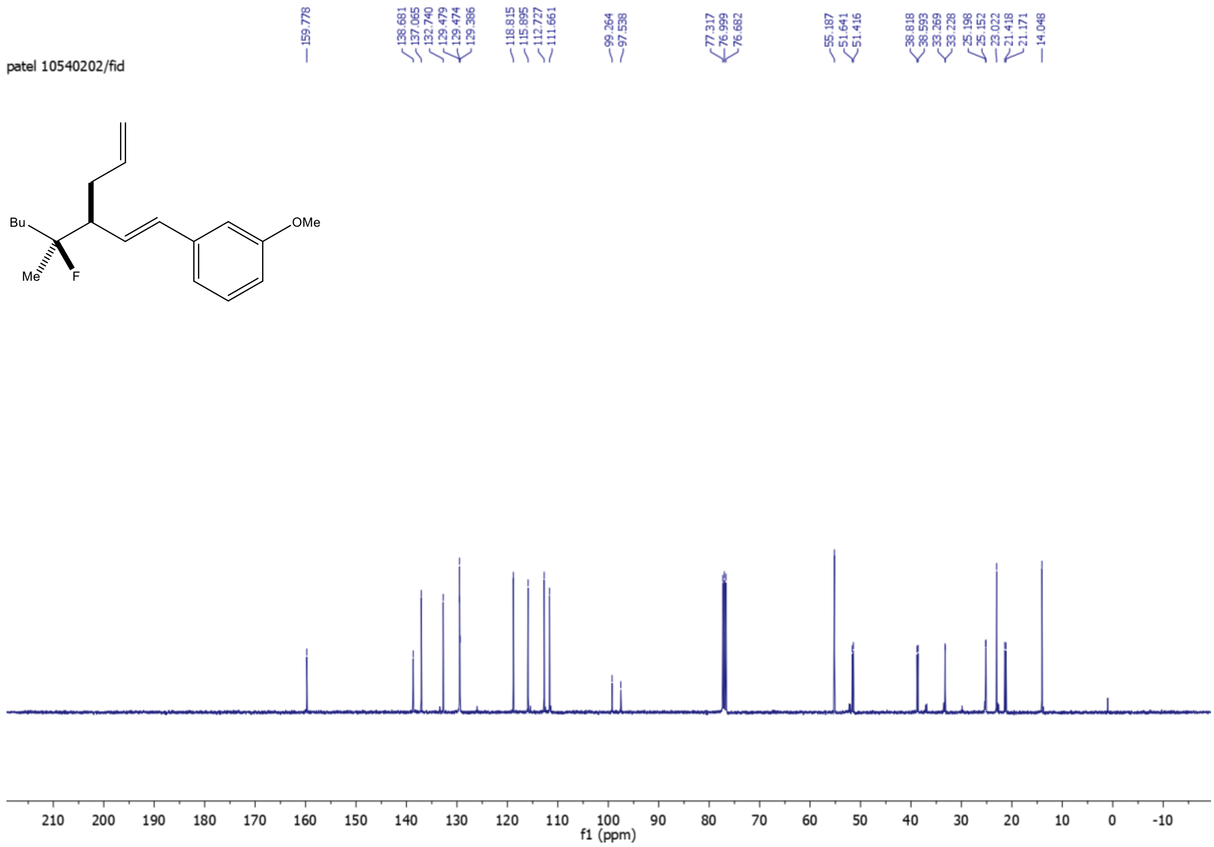
159.778
138.681 137.065 132.740 129.479 129.474 129.386
118.815 115.895 112.727 111.661
99.264 97.538
77.317 76.999 76.682
55.187 51.641 51.416
38.818 38.593 33.269 33.228 25.198 25.152 23.022 21.418 21.171 14.048
patel 10540202/fid
Bu
Me
F
OMe
210 200 190 180 170 160 150 140 130 120 110 100 90 80 70 60 50 40 30 20 10 0 -10
f1 (ppm)

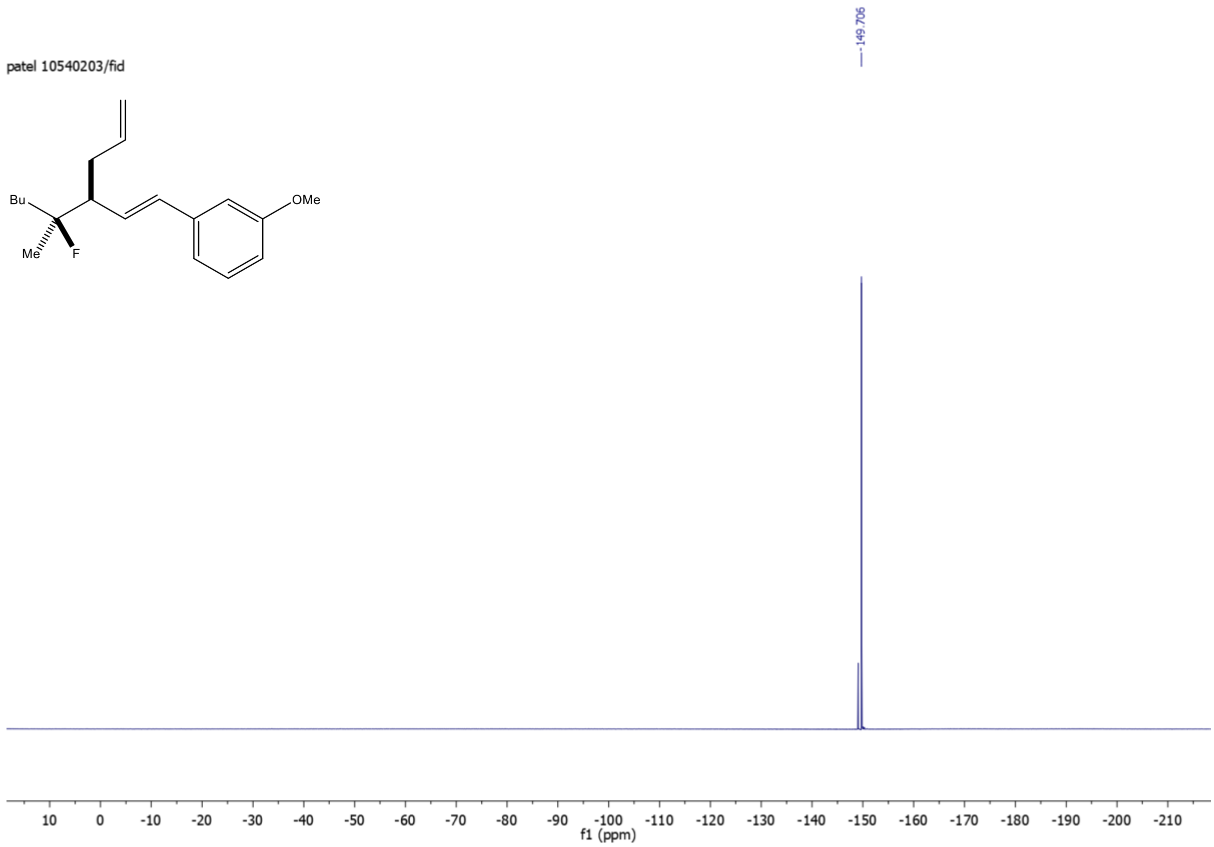

patel 10540203/fid
-149.706
Bu
Me
F
OMe
10 0 -10 -20 -30 -40 -50 -60 -70 -80 -90 -100 -110 -120 -130 -140 -150 -160 -170 -180 -190 -200 -210
f1 (ppm)

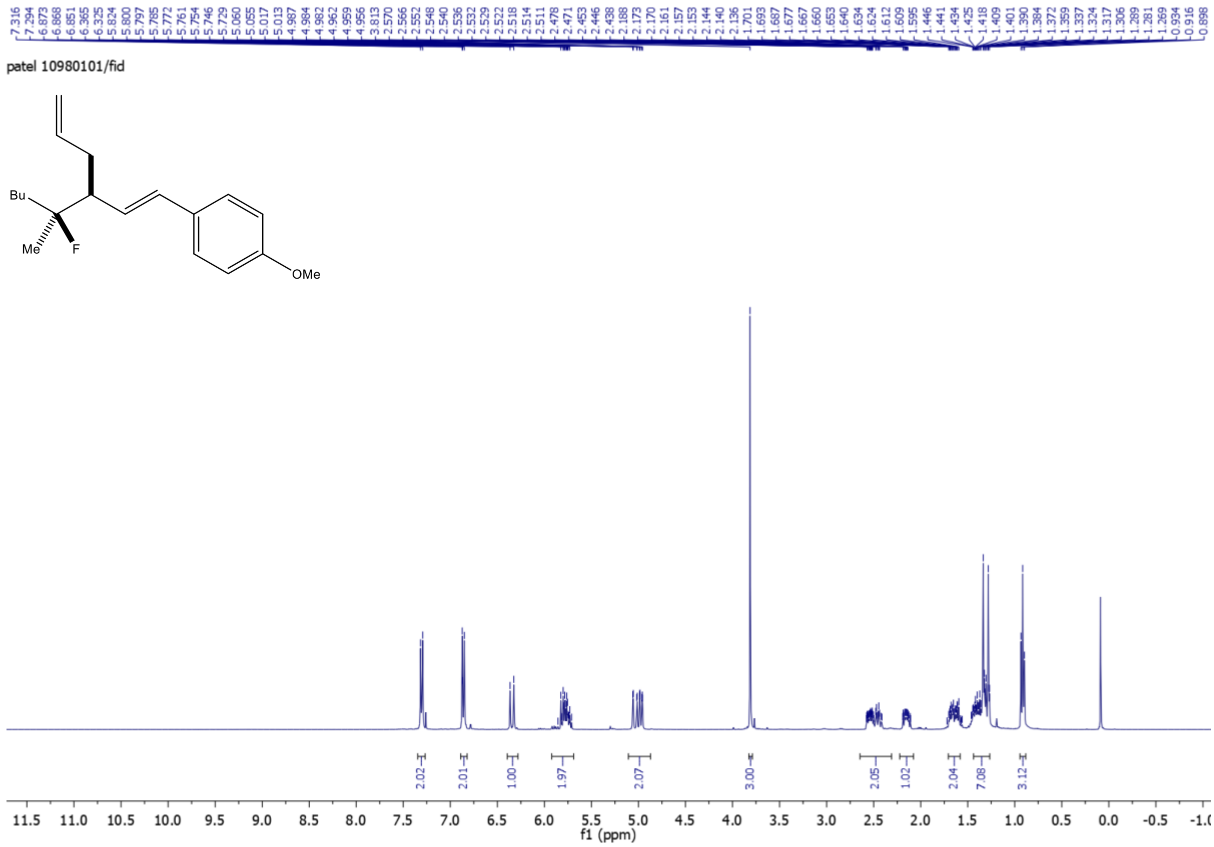

7.316 7.294 6.873 6.868 6.851 6.365 6.325 5.824 5.800 5.797 5.785 5.772 5.761 5.754 5.746 5.729 5.060 5.055 5.017 5.013 4.987 4.984 4.982 4.962 4.959 4.956 3.813 2.570 2.566 2.552 2.548 2.540 2.536 2.532 2.529 2.522 2.518 2.514 2.511 2.478 2.471 2.453 2.446 2.438 2.188 2.173 2.170 2.161 2.157 2.153 2.144 2.140 2.136 1.701 1.693 1.687 1.677 1.667 1.660 1.653 1.640 1.634 1.624 1.612 1.609 1.595 1.446 1.441 1.434 1.425 1.418 1.409 1.401 1.390 1.384 1.372 1.359 1.337 1.324 1.317 1.306 1.289 1.281 1.269 0.934 0.916 0.898
patel 10980101/fid
Bu
Me
F
OMe
2.02 2.01 1.00 1.97 2.07 3.00 2.05 1.02 2.04 7.08 3.12
11.5 11.0 10.5 10.0 9.5 9.0 8.5 8.0 7.5 7.0 6.5 6.0 5.5 5.0 4.5 4.0 3.5 3.0 2.5 2.0 1.5 1.0 0.5 0.0 -0.5
f1 (ppm)

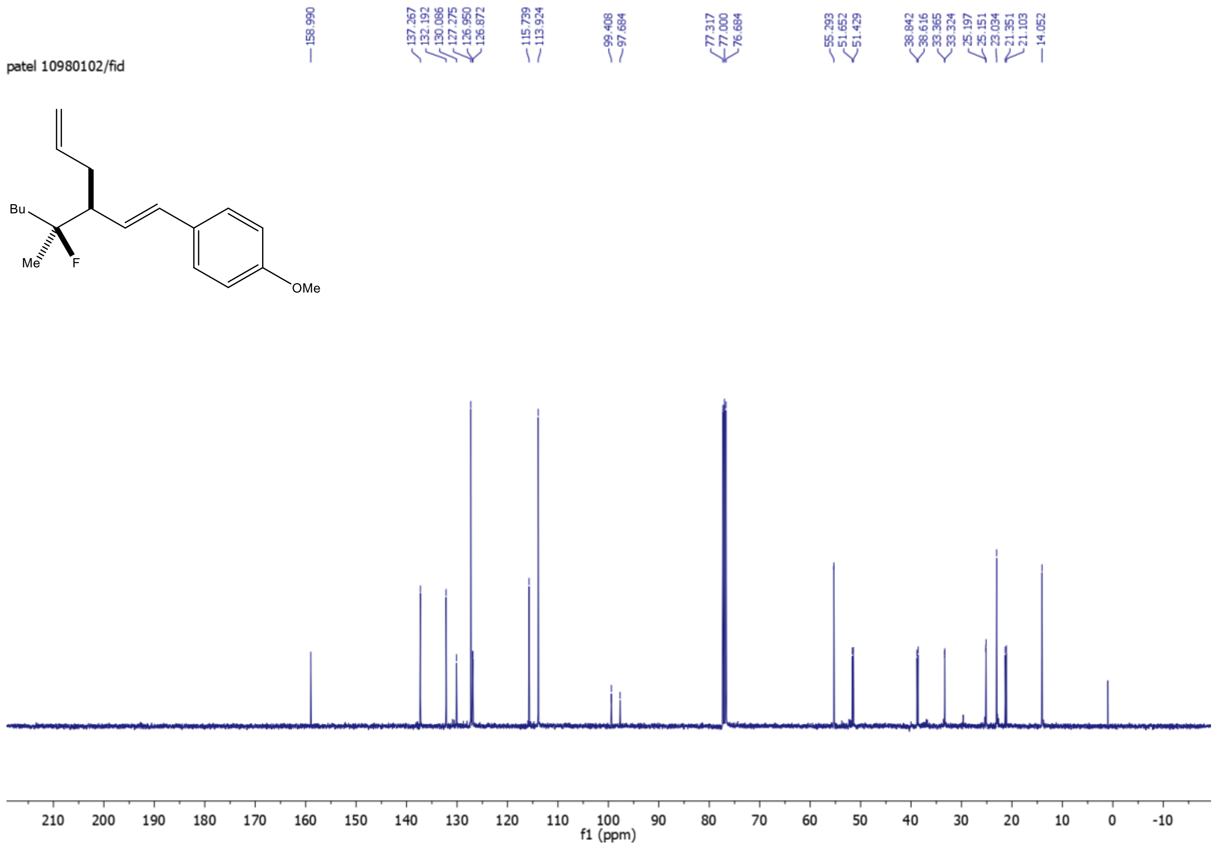
patel 10980102/fid
158.990
137.267
132.192
130.086
127.275
126.950
126.872
115.739
113.924
99.408
97.684
77.317
77.000
76.684
55.293
51.652
51.429
38.842
38.616
33.365
33.324
25.197
25.151
23.034
21.351
21.103
14.052
Bu
Me
F
OMe
f1 (ppm)

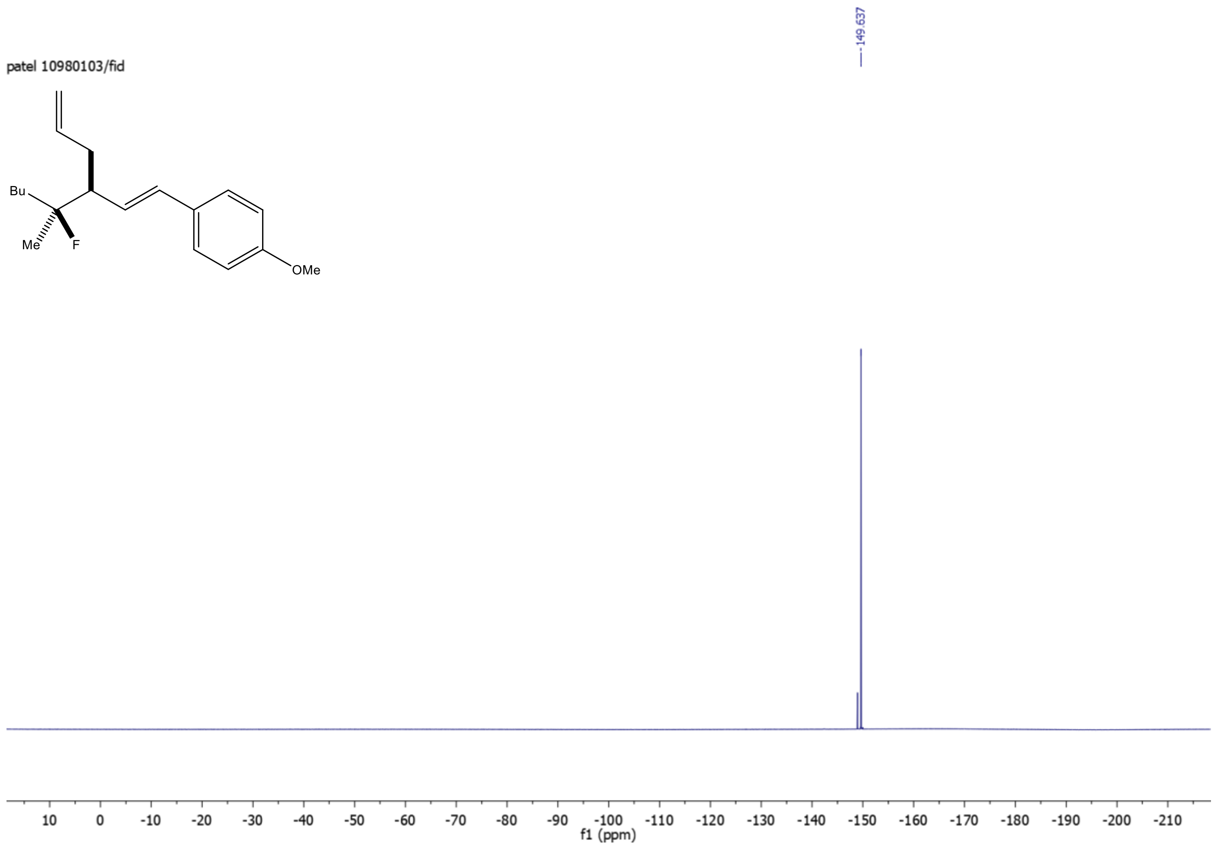
patel 10980103/fid
-149.637
Bu
Me
F
OMe
f1 (ppm)

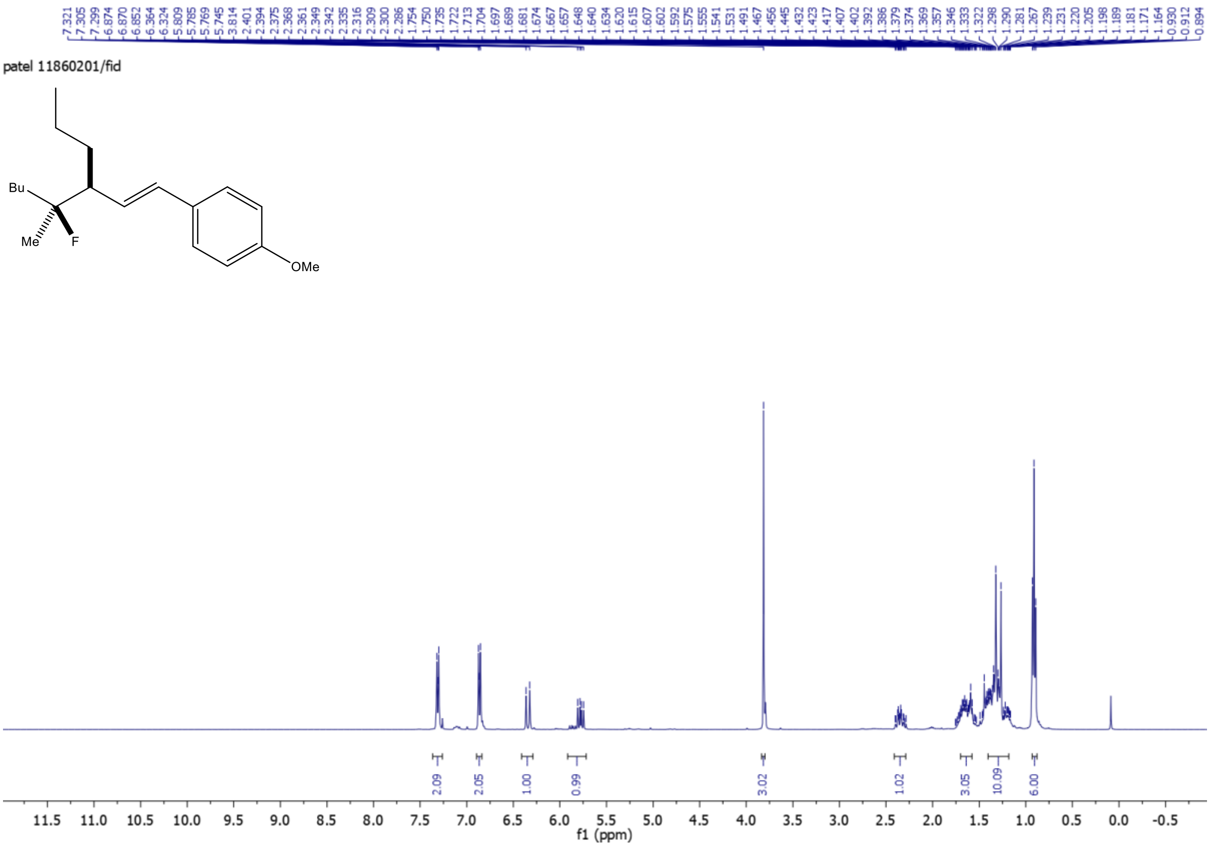
7.321 7.305 7.299 6.874 6.870 6.852 6.364 6.324 5.809 5.785 5.769 5.745 3.814 2.401 2.394 2.375 2.368 2.361 2.349 2.342 2.335 2.316 2.309 2.300 2.286 1.754 1.750 1.735 1.722 1.713 1.704 1.697 1.689 1.681 1.674 1.667 1.657 1.648 1.640 1.634 1.620 1.615 1.607 1.602 1.592 1.575 1.555 1.541 1.531 1.491 1.467 1.456 1.445 1.432 1.423 1.417 1.407 1.402 1.392 1.386 1.379 1.374 1.369 1.357 1.346 1.333 1.322 1.298 1.290 1.281 1.267 1.239 1.231 1.220 1.205 1.198 1.189 1.181 1.171 1.164 0.930 0.912 0.894
patel 11860201/fid
Bu
Me
F
OMe
2.09
2.05
1.00
0.99
3.02
1.02
3.05
10.09
6.00
11.5 11.0 10.5 10.0 9.5 9.0 8.5 8.0 7.5 7.0 6.5 6.0 5.5 5.0 4.5 4.0 3.5 3.0 2.5 2.0 1.5 1.0 0.5 0.0 -0.5
f1 (ppm)

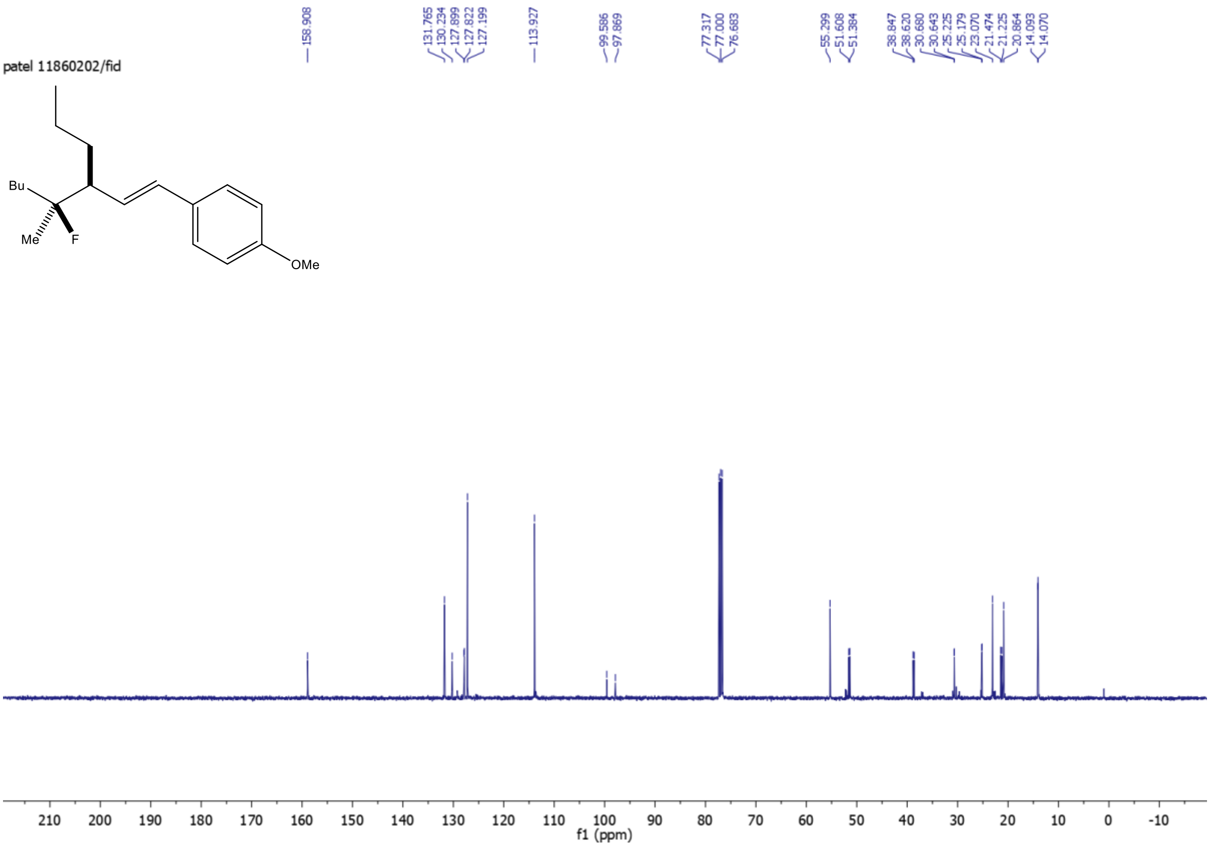
158.908
131.765 130.234 127.899 127.822 127.199
113.927
99.586 97.869
77.317 77.000 76.683
55.299 51.608 51.384
38.847 38.620 30.680 30.643 25.225 25.179 23.070 21.474 21.225 20.864 14.093 14.070
patel 11860202/fid
Bu
Me
F
OMe
210 200 190 180 170 160 150 140 130 120 110 100 90 80 70 60 50 40 30 20 10 0 -10
f1 (ppm)

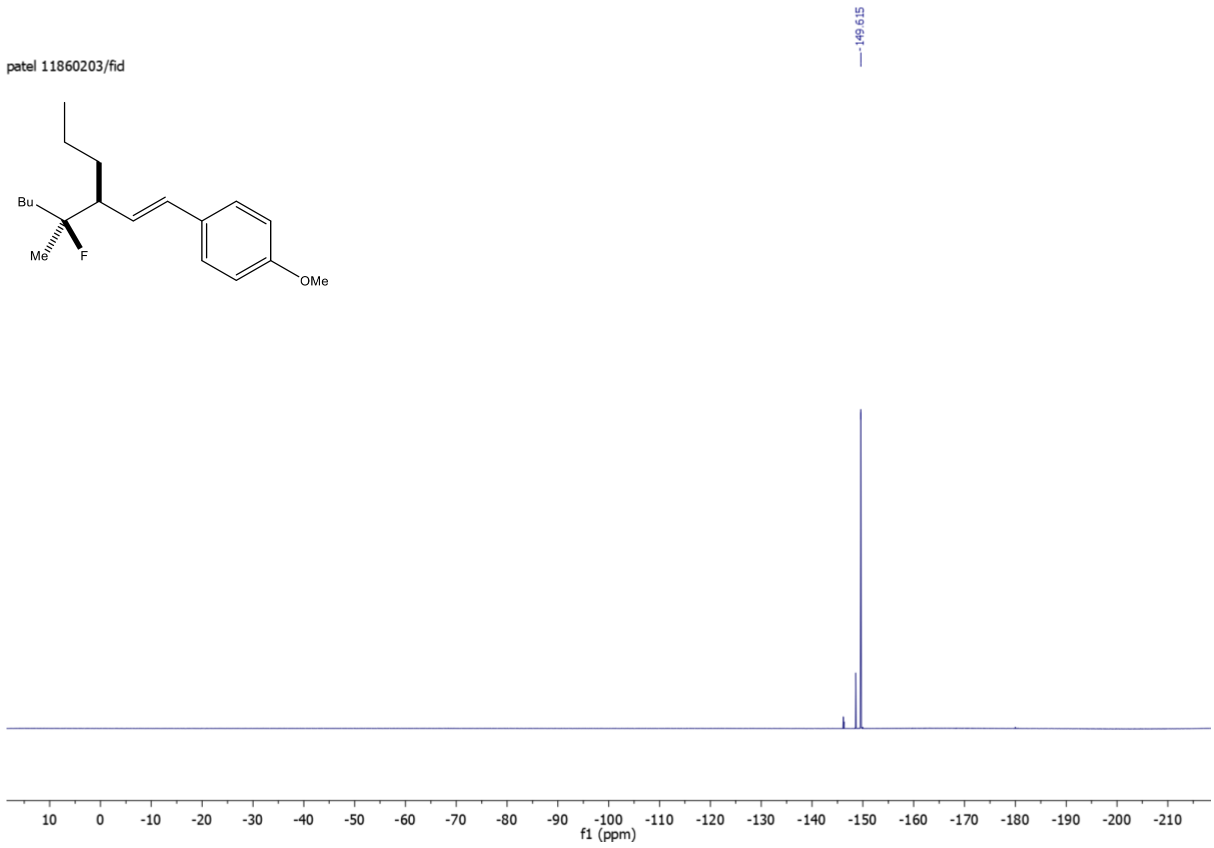
patel 11860203/fid
-149.615
Bu
Me
F
OMe
f1 (ppm)

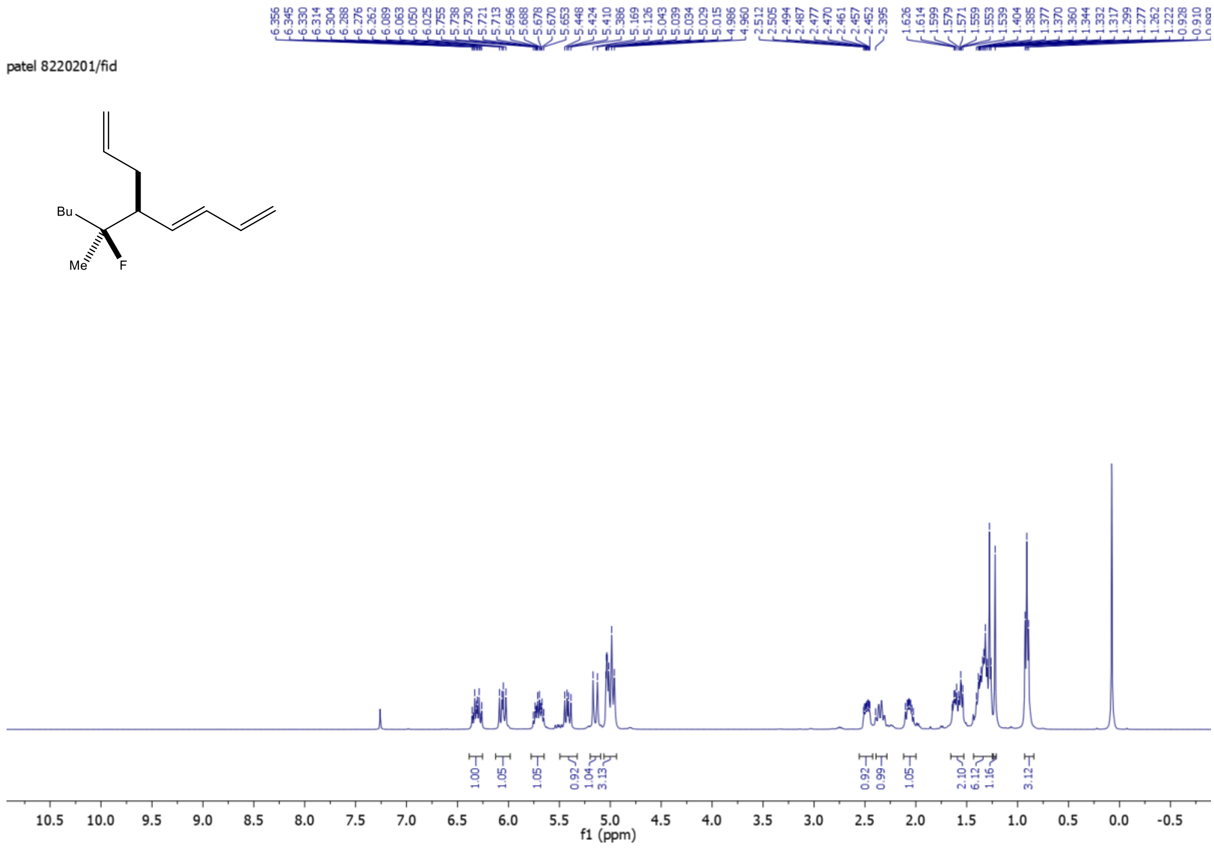
patel 8220201/fid
Bu
Me
F
f1 (ppm)

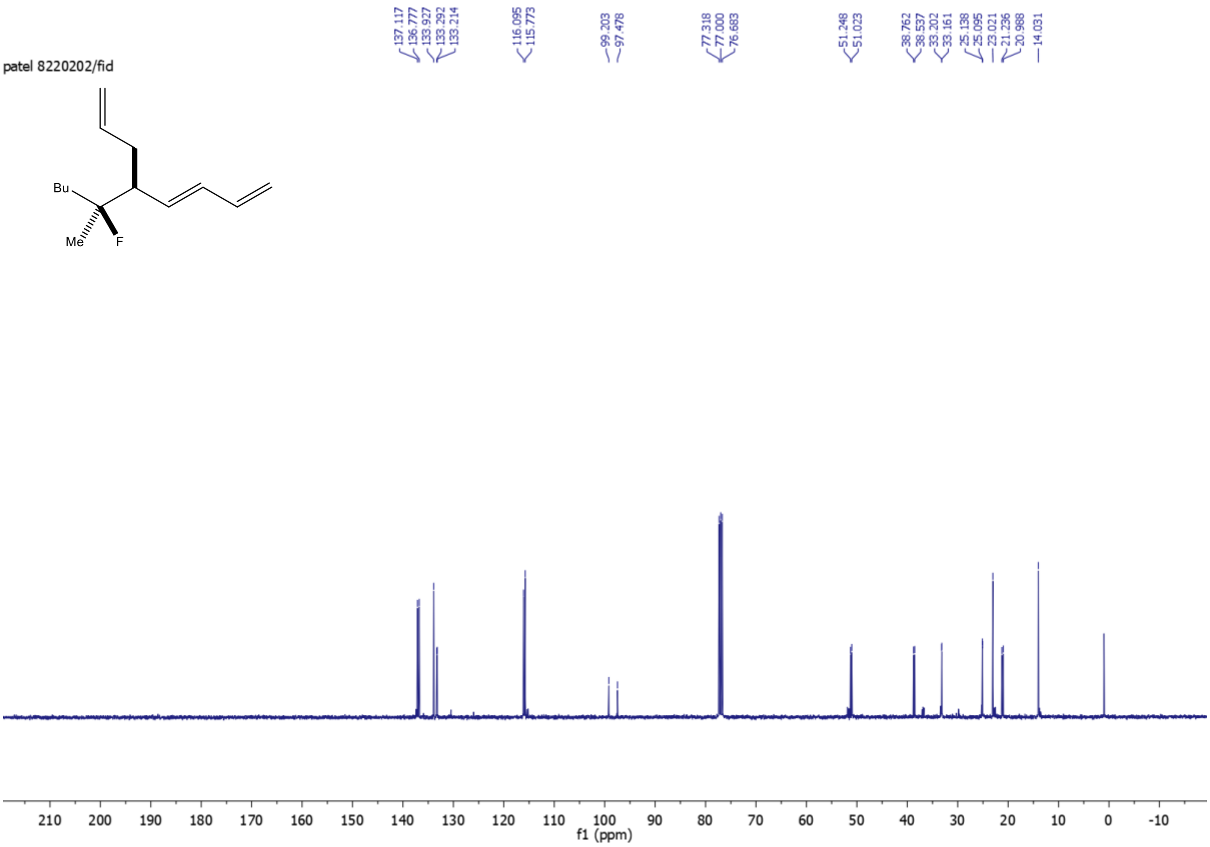
patel 8220202/fid
Bu
Me
F
137.117
136.777
133.927
133.292
133.214
116.095
115.773
99.203
97.478
77.318
77.000
76.683
51.248
51.023
38.762
38.537
33.202
33.161
25.138
25.095
23.021
21.236
20.988
14.031
210 200 190 180 170 160 150 140 130 120 110 100 90 80 70 60 50 40 30 20 10 0 -10
f1 (ppm)

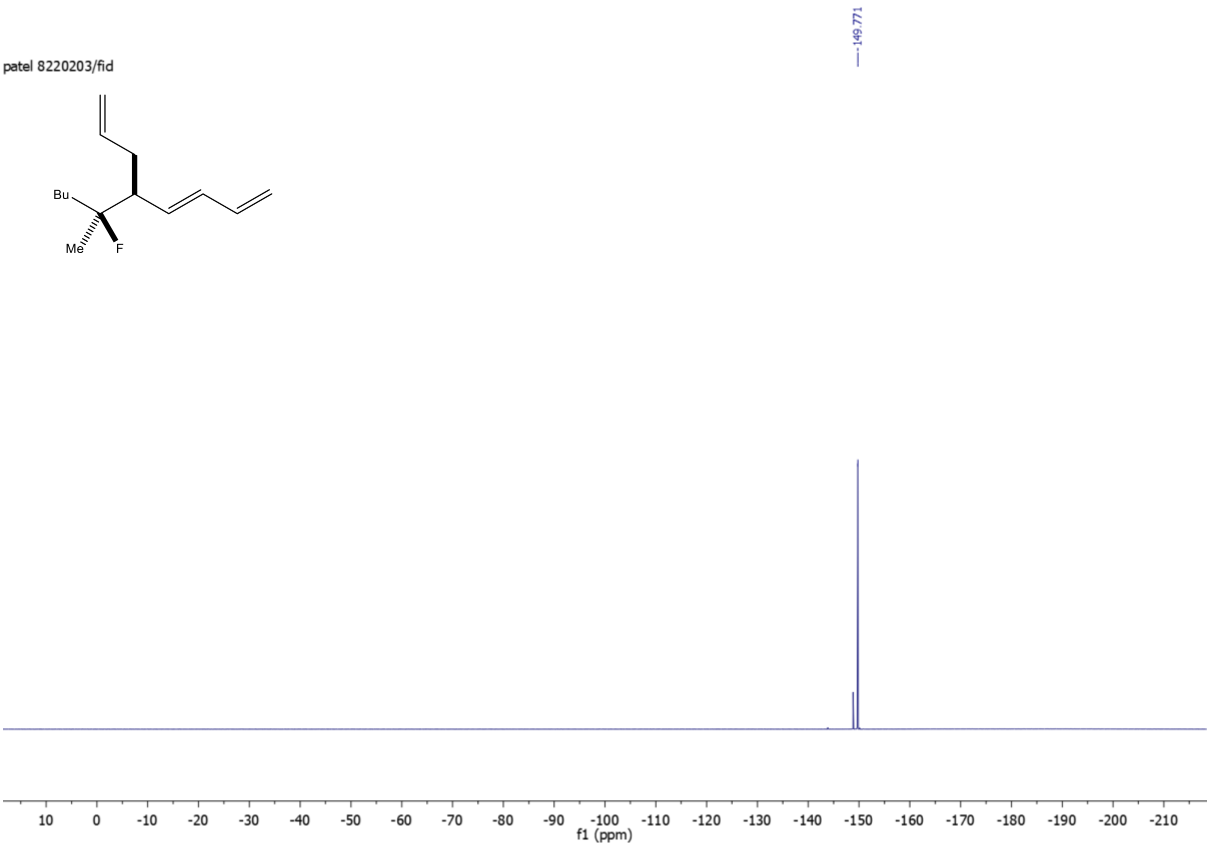
patel 8220203/fid
Bu
Me
F
-149.771
10 0 -10 -20 -30 -40 -50 -60 -70 -80 -90 -100 -110 -120 -130 -140 -150 -160 -170 -180 -190 -200 -210
f1 (ppm)

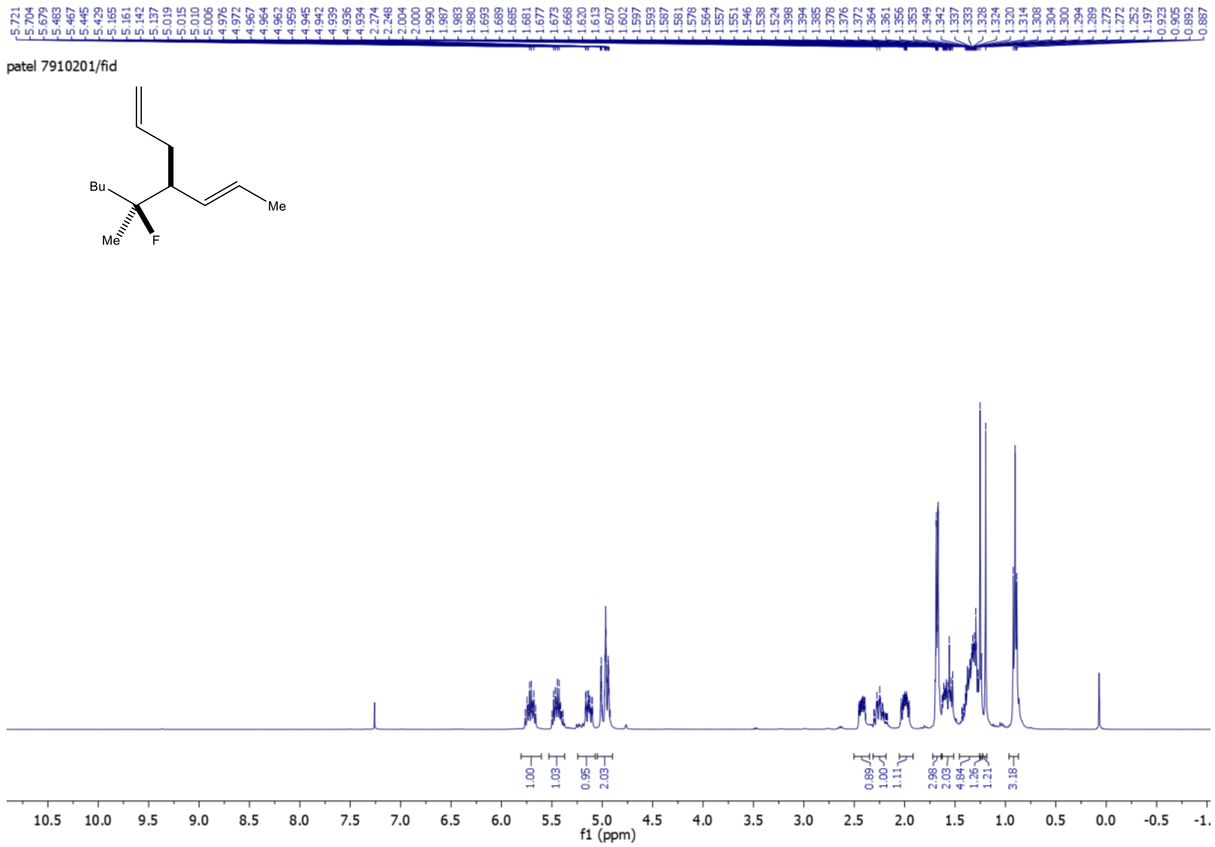
5.721 5.704 5.679 5.483 5.467 5.445 5.429 5.165 5.161 5.142 5.137 5.019 5.015 5.010 5.006 4.976 4.972 4.967 4.964 4.962 4.959 4.945 4.942 4.939 4.936 4.934 2.274 2.248 2.004 2.000 1.990 1.987 1.983 1.980 1.693 1.689 1.685 1.681 1.677 1.673 1.668 1.620 1.613 1.607 1.602 1.597 1.593 1.587 1.581 1.578 1.564 1.557 1.551 1.546 1.538 1.524 1.398 1.394 1.385 1.378 1.376 1.372 1.364 1.361 1.356 1.353 1.349 1.342 1.337 1.333 1.328 1.324 1.320 1.314 1.308 1.304 1.300 1.294 1.289 1.273 1.272 1.252 1.197 0.923 0.905 0.892 0.887
patel 7910201/fid
Bu
Me
F
Me
1.00 1.03 0.95 2.03 0.89 1.00 1.11 2.98 2.03 4.84 1.26 1.21 3.18
f1 (ppm)

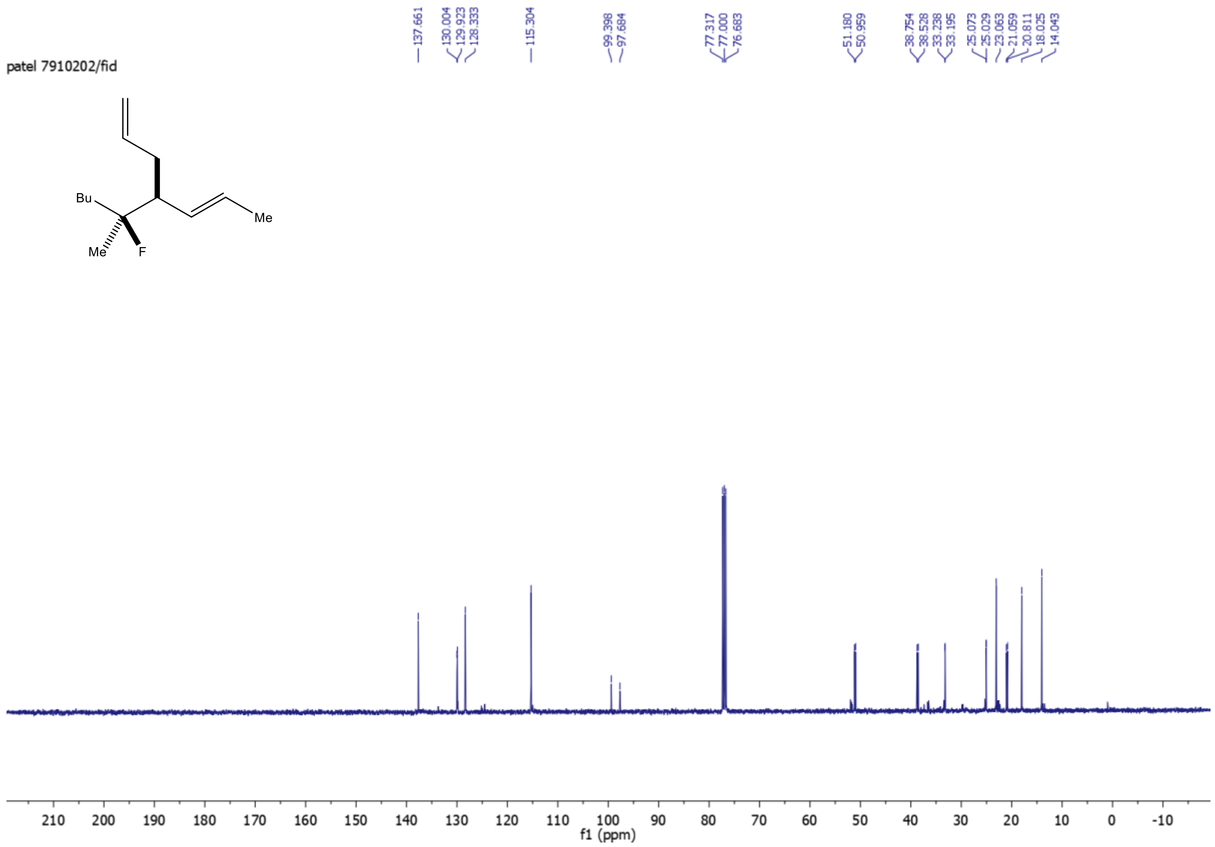
137.661 130.004 129.923 128.333 115.304 99.398 97.684 77.317 77.000 76.683 51.180 50.959 38.754 38.528 33.238 33.195 25.073 25.029 23.063 21.059 20.811 18.025 14.043
patel 7910202/fid
Bu
Me
F
Me
f1 (ppm)

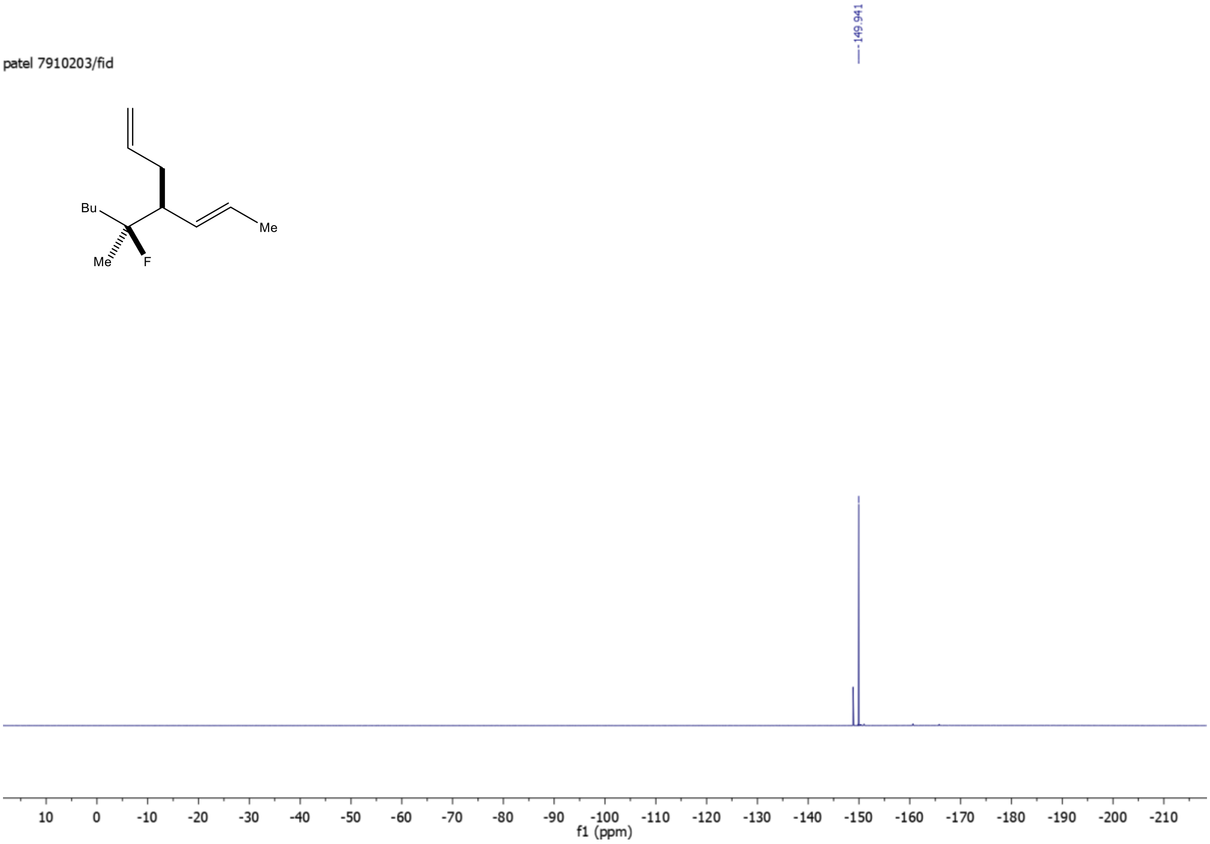
-149.941
patel 7910203/fid
Bu
Me
F
Me
10 0 -10 -20 -30 -40 -50 -60 -70 -80 -90 -100 -110 -120 -130 -140 -150 -160 -170 -180 -190 -200 -210
f1 (ppm)

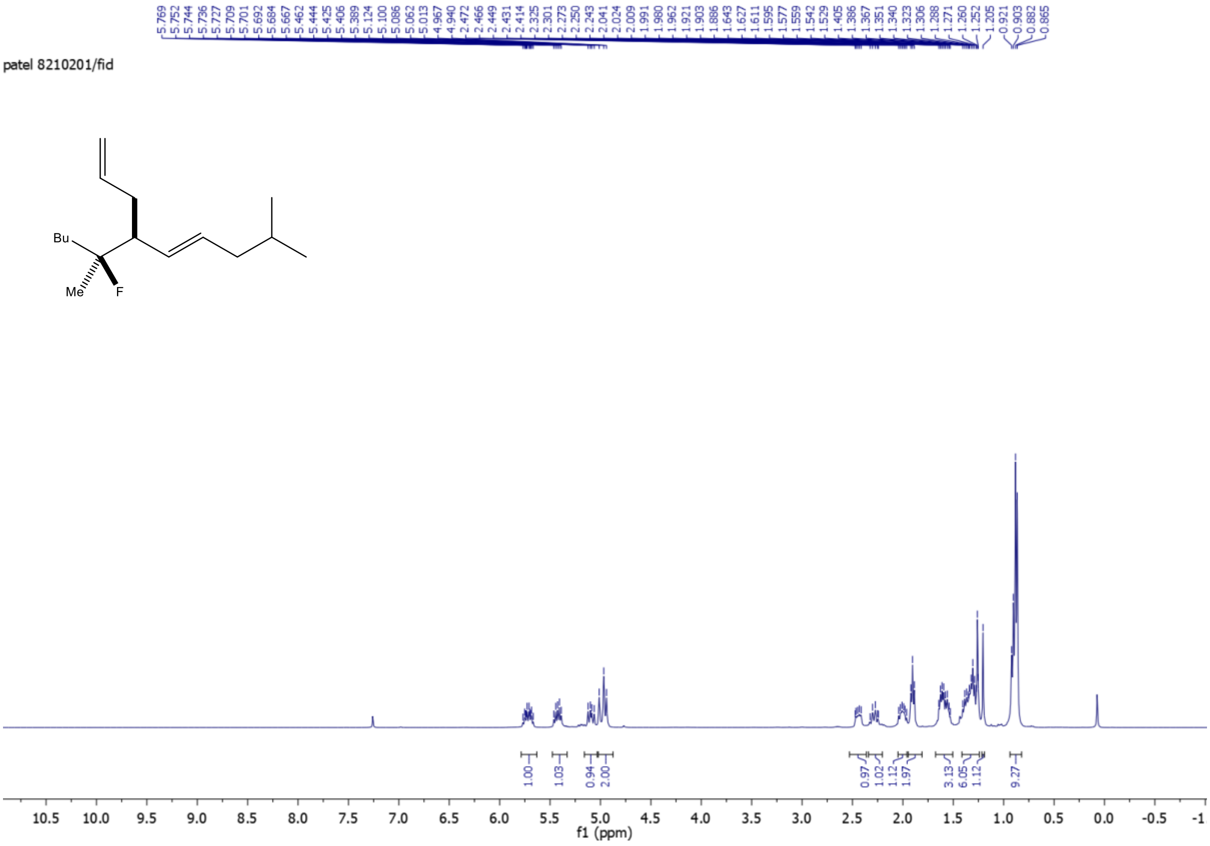
5.769 5.752 5.744 5.736 5.727 5.709 5.701 5.692 5.684 5.667 5.462 5.444 5.425 5.406 5.389 5.124 5.100 5.086 5.062 5.013 4.967 4.940 2.472 2.466 2.449 2.431 2.414 2.325 2.301 2.273 2.250 2.243 2.041 2.024 2.009 1.991 1.980 1.962 1.921 1.903 1.886 1.643 1.627 1.611 1.595 1.577 1.559 1.542 1.529 1.405 1.386 1.367 1.351 1.340 1.323 1.306 1.288 1.271 1.260 1.252 1.205 0.921 0.903 0.882 0.865
patel 8210201/fid
Bu
Me
F
1.00 1.03 0.94 2.00 0.97 1.02 1.12 1.97 3.13 6.05 1.12 9.27
10.5 10.0 9.5 9.0 8.5 8.0 7.5 7.0 6.5 6.0 5.5 5.0 4.5 4.0 3.5 3.0 2.5 2.0 1.5 1.0 0.5 0.0 -0.5 -1
f1 (ppm)

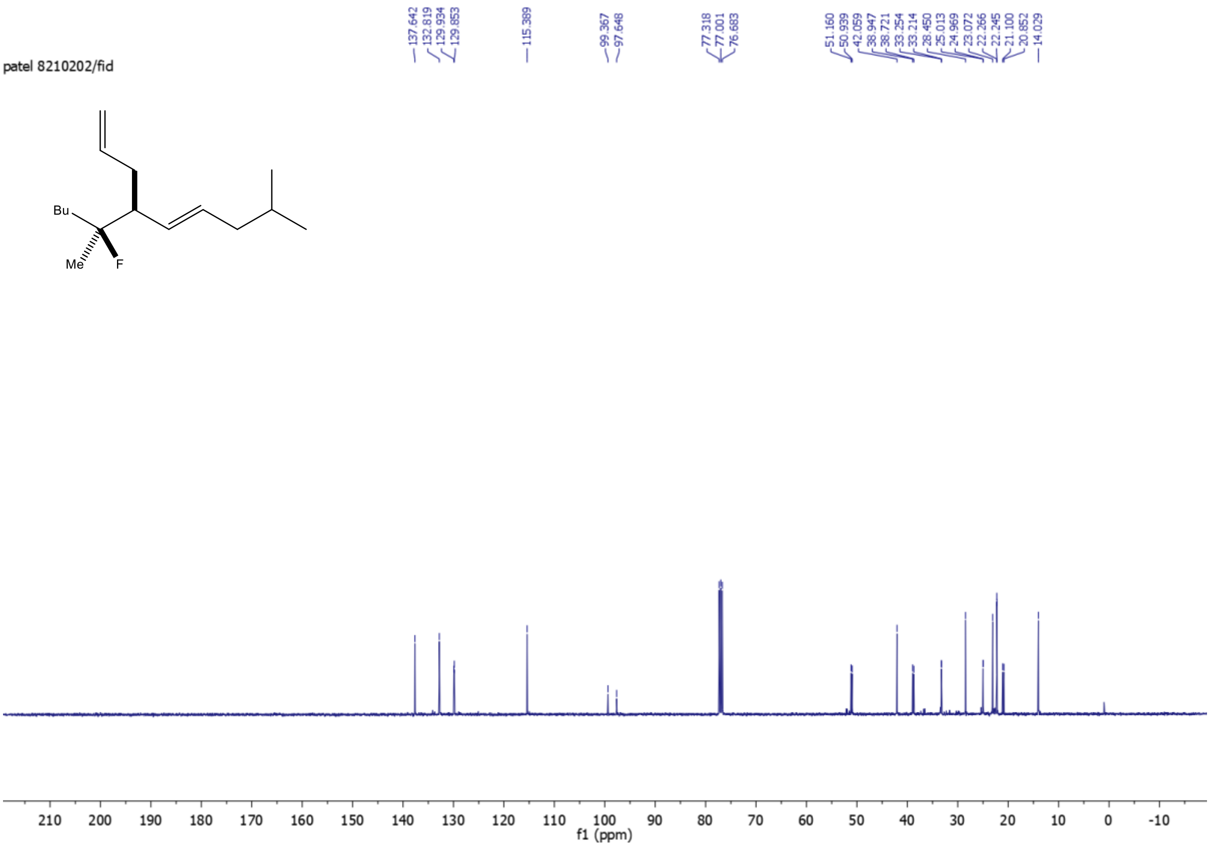
patel 8210202/fid
137.642
132.819
129.934
129.853
115.389
99.367
97.648
77.318
77.001
76.683
51.160
50.939
42.059
38.947
38.721
33.254
33.214
28.450
25.013
24.969
23.072
22.266
22.245
21.100
20.852
14.029
Bu
Me
F
210 200 190 180 170 160 150 140 130 120 110 100 90 80 70 60 50 40 30 20 10 0 -10
f1 (ppm)

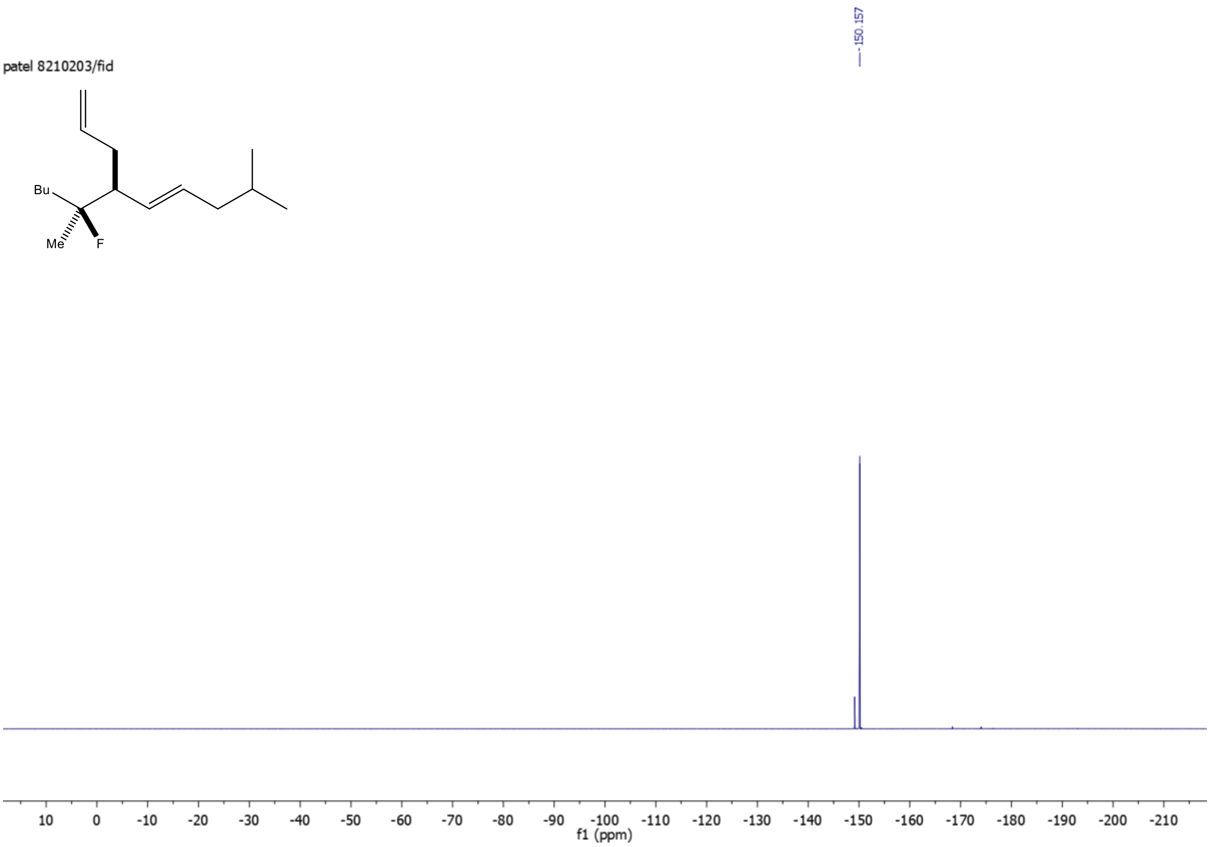
patel 8210203/fid
-150.157
Bu
Me
F
10 0 -10 -20 -30 -40 -50 -60 -70 -80 -90 -100 -110 -120 -130 -140 -150 -160 -170 -180 -190 -200 -210
f1 (ppm)

patel 81790301/fid

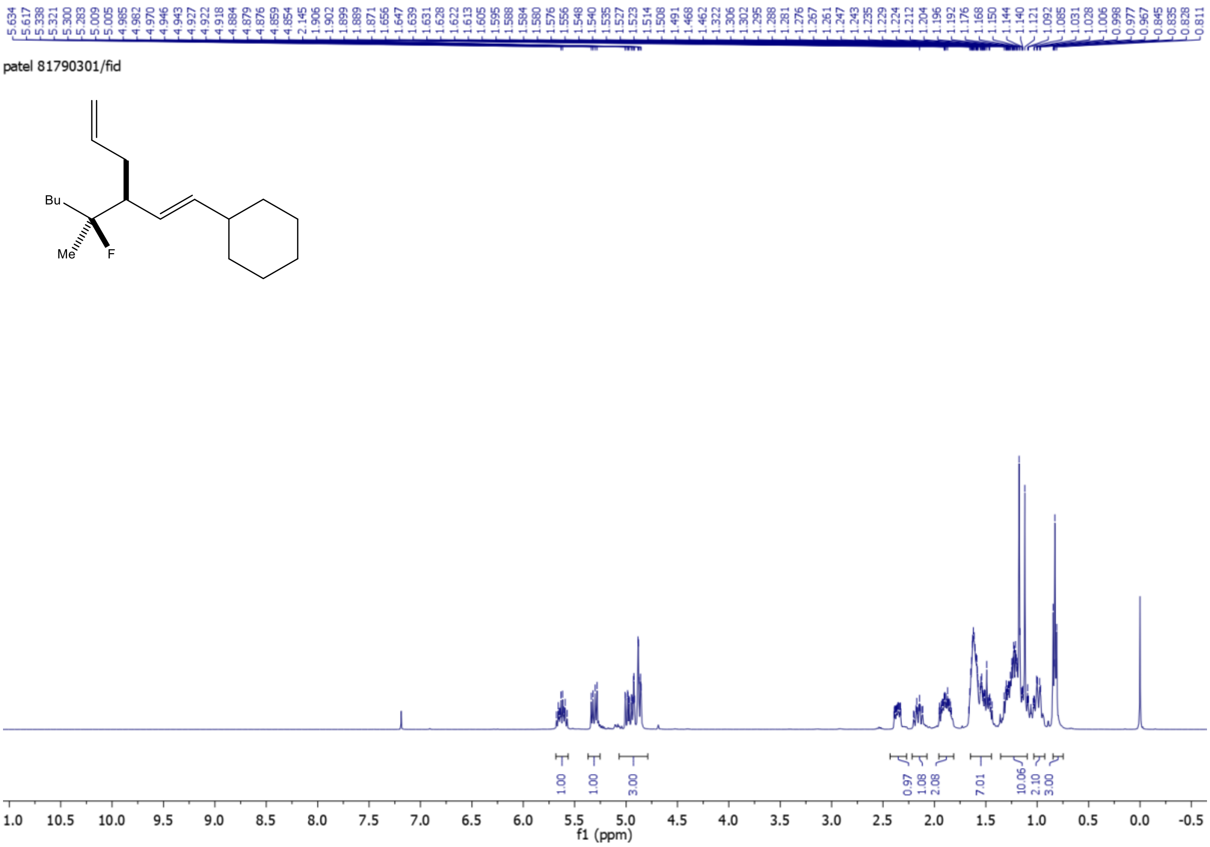

patel 81790302/fid

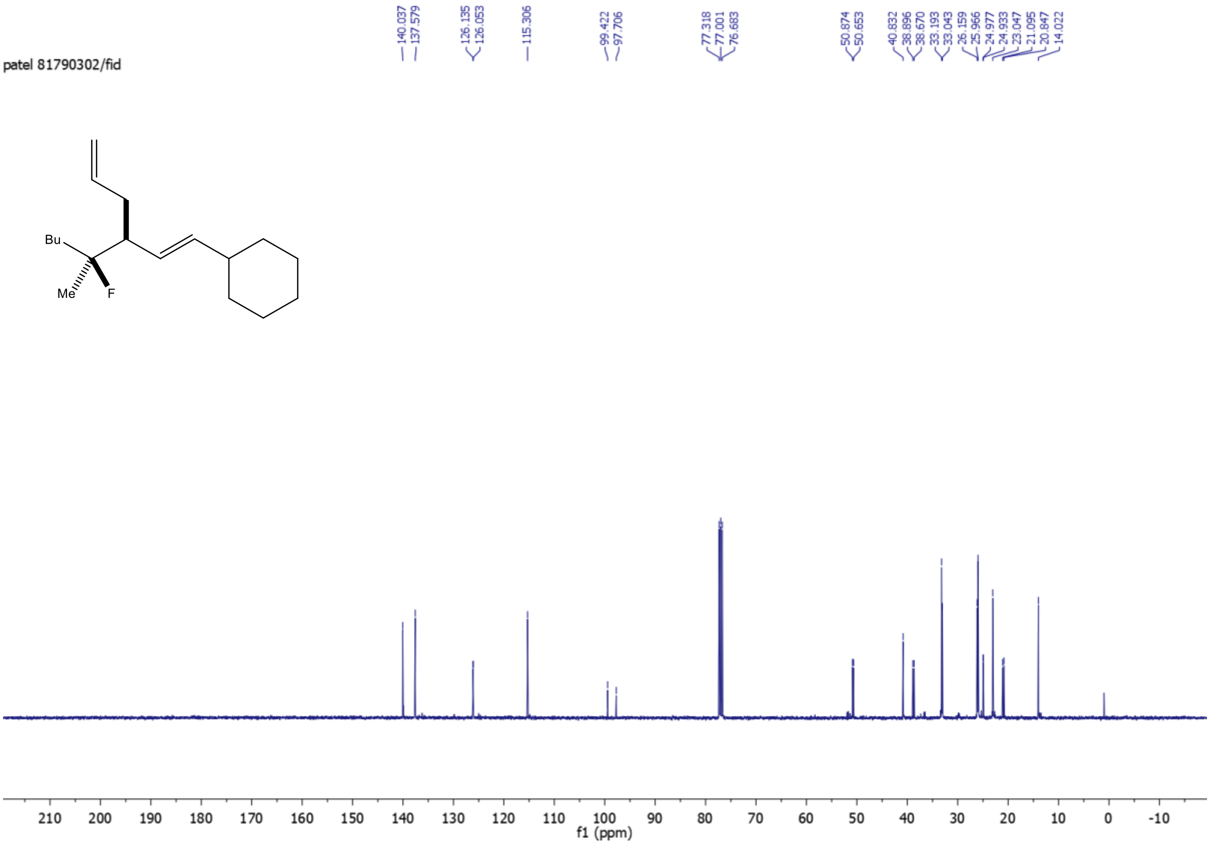

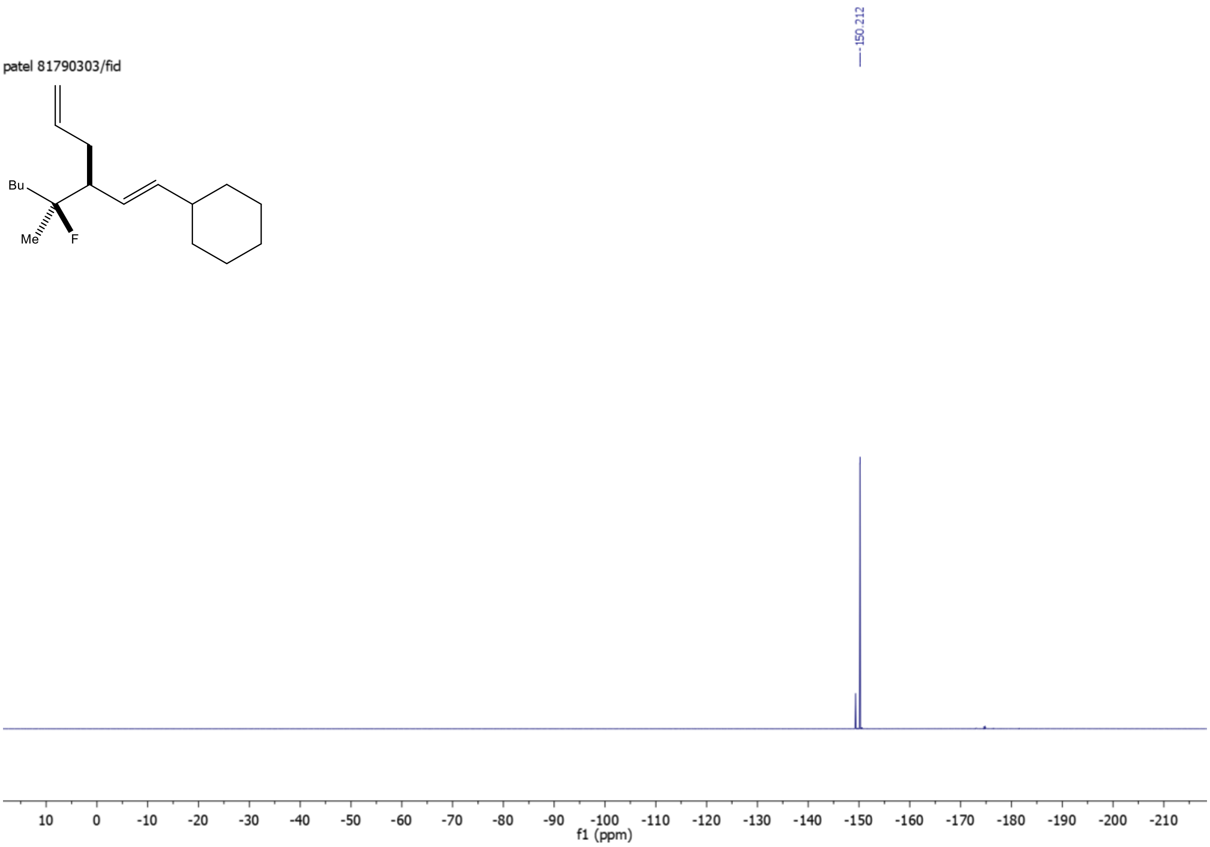
-150.212
patel 81790303/fid
Bu
Me
F
f1 (ppm)

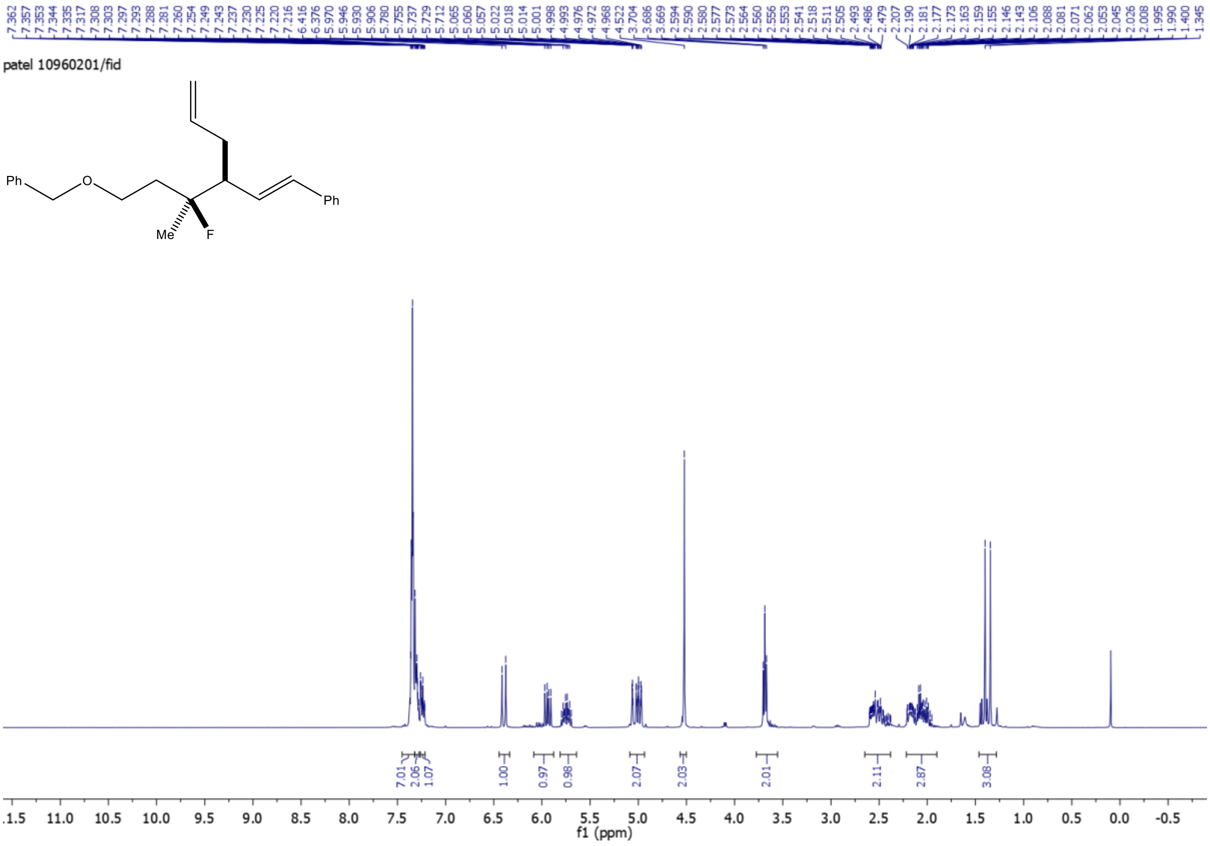
patel 10960201/fid
Ph
O
Ph
Me
F
7.01
2.06
1.07
1.00
0.97
0.98
2.07
2.03
2.01
2.11
2.87
3.08
f1 (ppm)

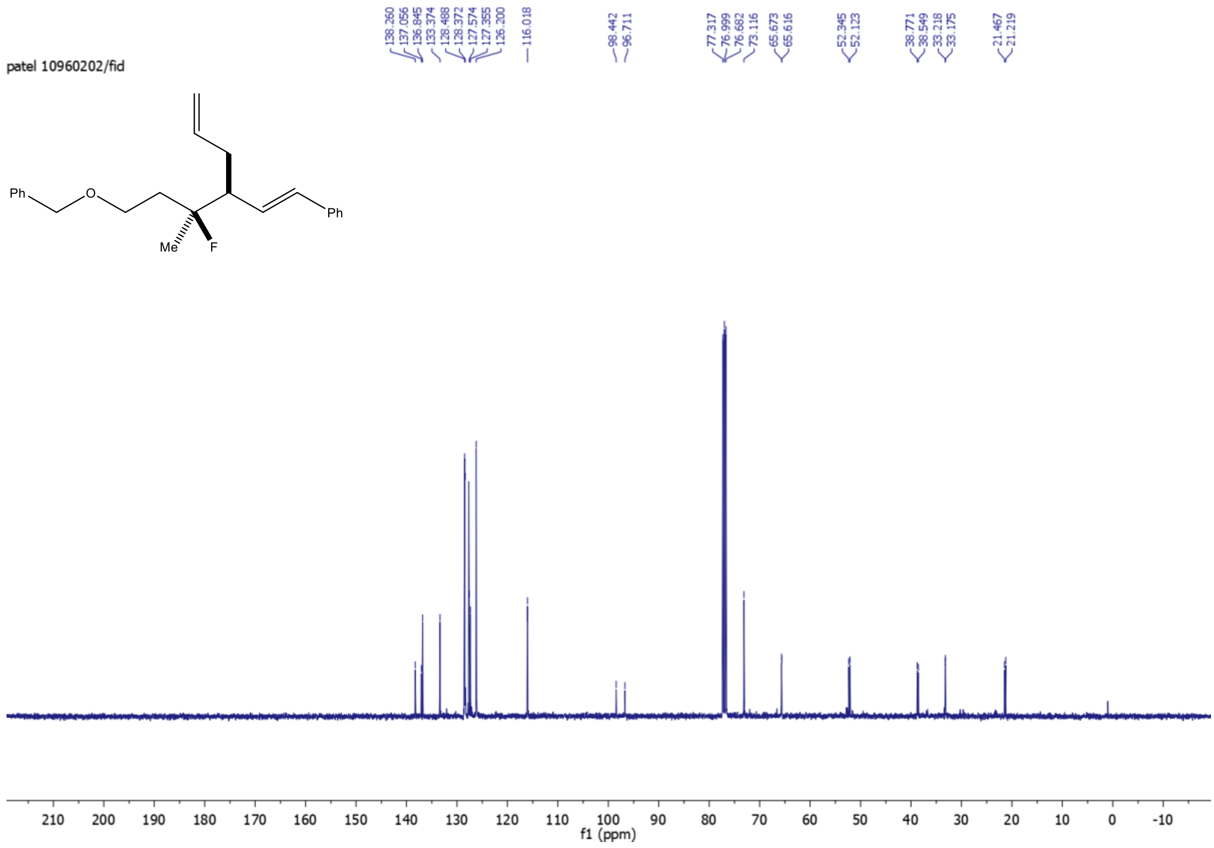
patel 10960202/fid
138.260
137.056
136.845
133.374
128.488
128.372
127.574
127.355
126.200
116.018
98.442
96.711
77.317
76.999
76.682
73.116
65.673
65.616
52.345
52.123
38.771
38.549
33.218
33.175
21.467
21.219
Ph
O
Me
F
Ph
210 200 190 180 170 160 150 140 130 120 110 100 90 80 70 60 50 40 30 20 10 0 -10
f1 (ppm)

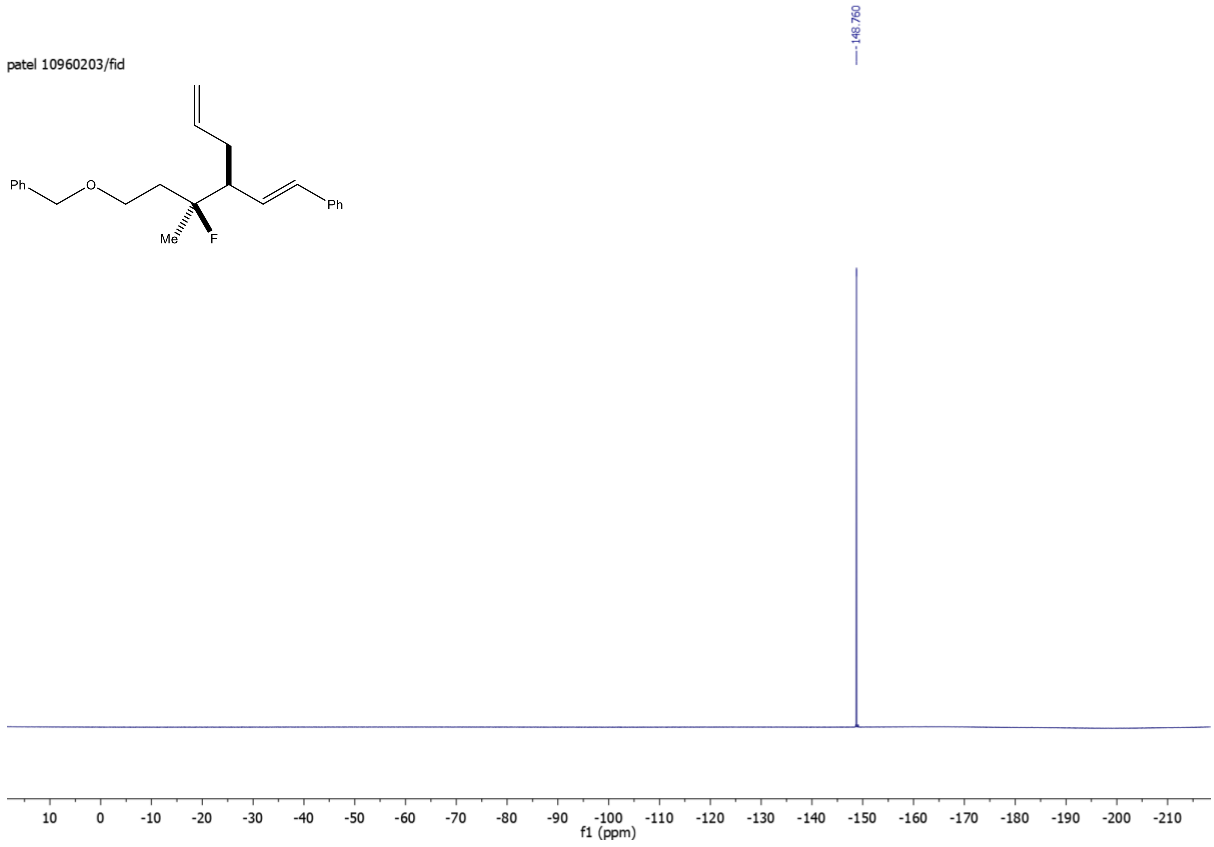
patel 10960203/fid
-148.760
Ph
O
Me
F
Ph
10 0 -10 -20 -30 -40 -50 -60 -70 -80 -90 -100 -110 -120 -130 -140 -150 -160 -170 -180 -190 -200 -210
f1 (ppm)

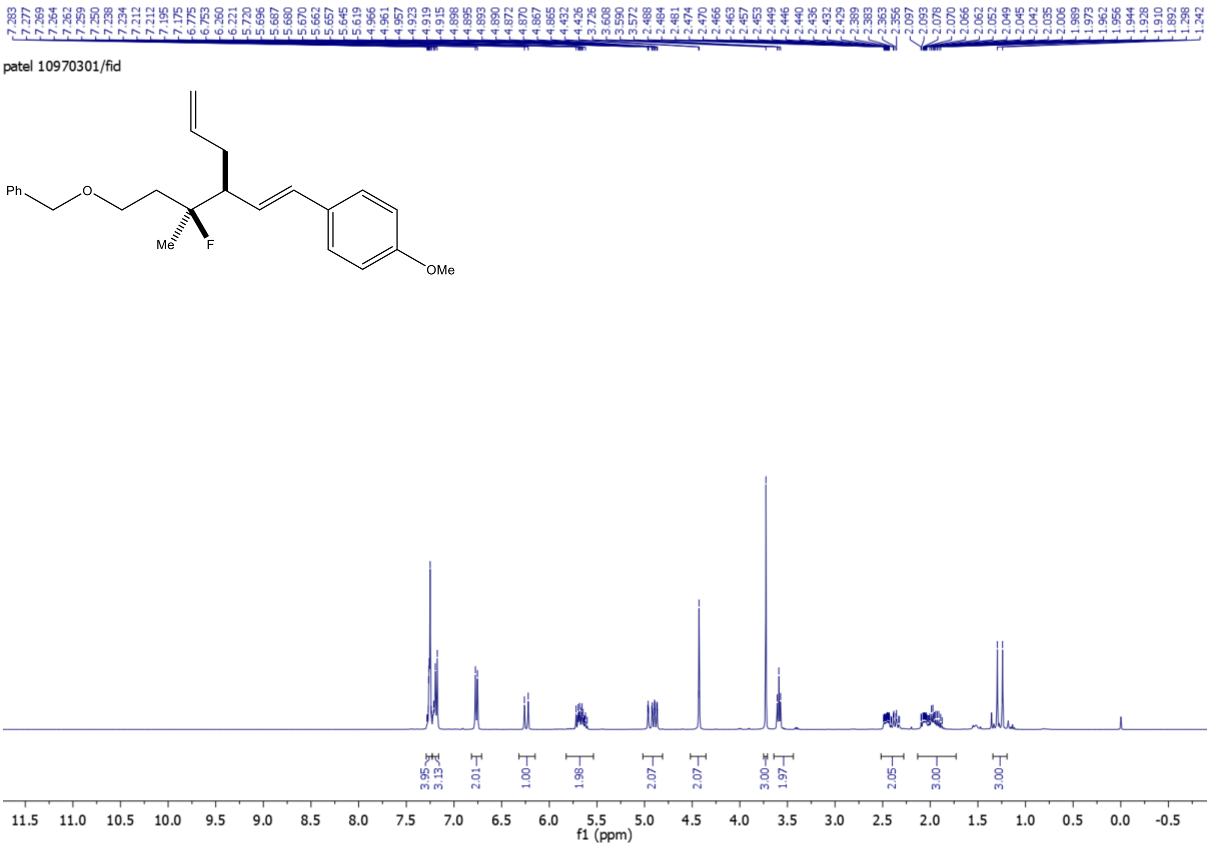
7.283 7.277 7.269 7.264 7.262 7.259 7.250 7.238 7.234 7.212 7.212 7.195 7.175 6.775 6.753 6.260 6.221 5.720 5.696 5.687 5.680 5.670 5.662 5.657 5.645 5.619 4.966 4.961 4.957 4.923 4.919 4.915 4.898 4.895 4.893 4.890 4.872 4.870 4.867 4.865 4.432 4.426 3.726 3.608 3.590 3.572 2.488 2.484 2.481 2.474 2.470 2.466 2.463 2.457 2.453 2.449 2.446 2.440 2.436 2.432 2.429 2.389 2.383 2.363 2.356 2.097 2.093 2.078 2.070 2.066 2.062 2.052 2.049 2.045 2.042 2.035 2.006 1.989 1.973 1.962 1.956 1.944 1.928 1.910 1.892 1.298 1.242
patel 10970301/fid
Ph
O
Me
F
OMe
3.95 3.13 2.01 1.00 1.98 2.07 2.07 3.00 1.97 2.05 3.00 3.00
11.5 11.0 10.5 10.0 9.5 9.0 8.5 8.0 7.5 7.0 6.5 6.0 5.5 5.0 4.5 4.0 3.5 3.0 2.5 2.0 1.5 1.0 0.5 0.0 -0.5
f1 (ppm)

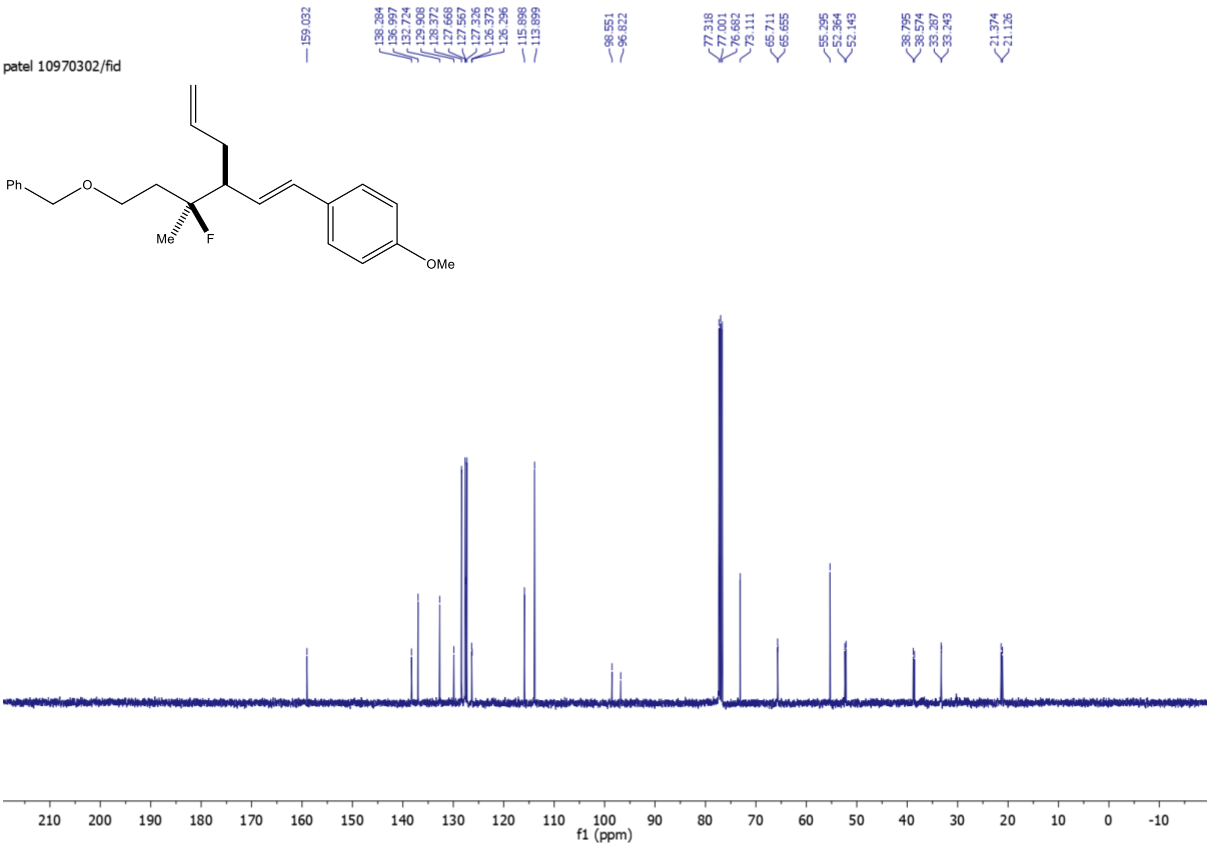
159.032
138.284 136.997 132.724 129.908 128.372 127.668 127.567 127.326 126.373 126.296 115.898 113.899
98.551 96.822
77.318 77.001 76.682 73.111 65.711 65.655
55.295 52.364 52.143
38.795 38.574 33.287 33.243
21.374 21.126
patel 10970302/fid
Ph
O
Me
F
OMe
210 200 190 180 170 160 150 140 130 120 110 100 90 80 70 60 50 40 30 20 10 0 -10
f1 (ppm)

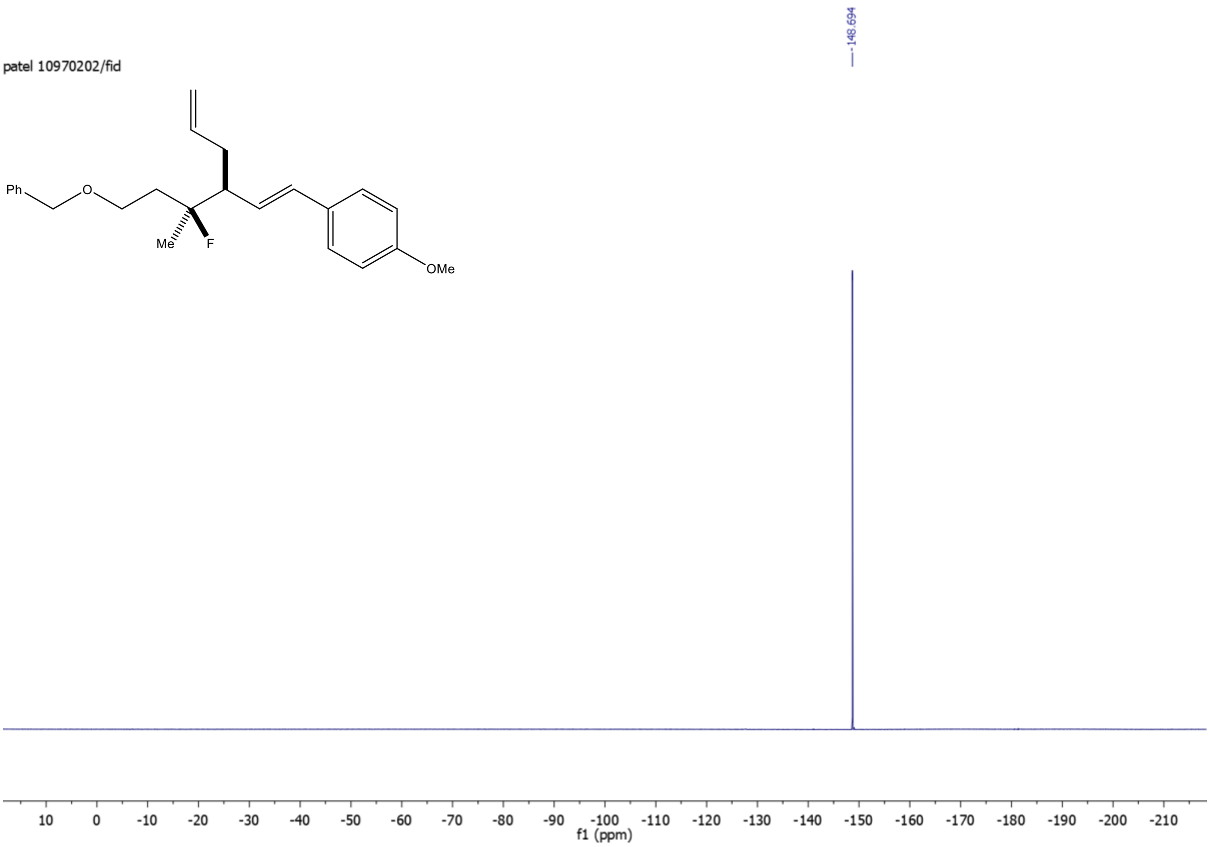
patel 10970202/fid
Ph
O
Me
F
OMe
-148.694
10 0 -10 -20 -30 -40 -50 -60 -70 -80 -90 -100 -110 -120 -130 -140 -150 -160 -170 -180 -190 -200 -210
f1 (ppm)

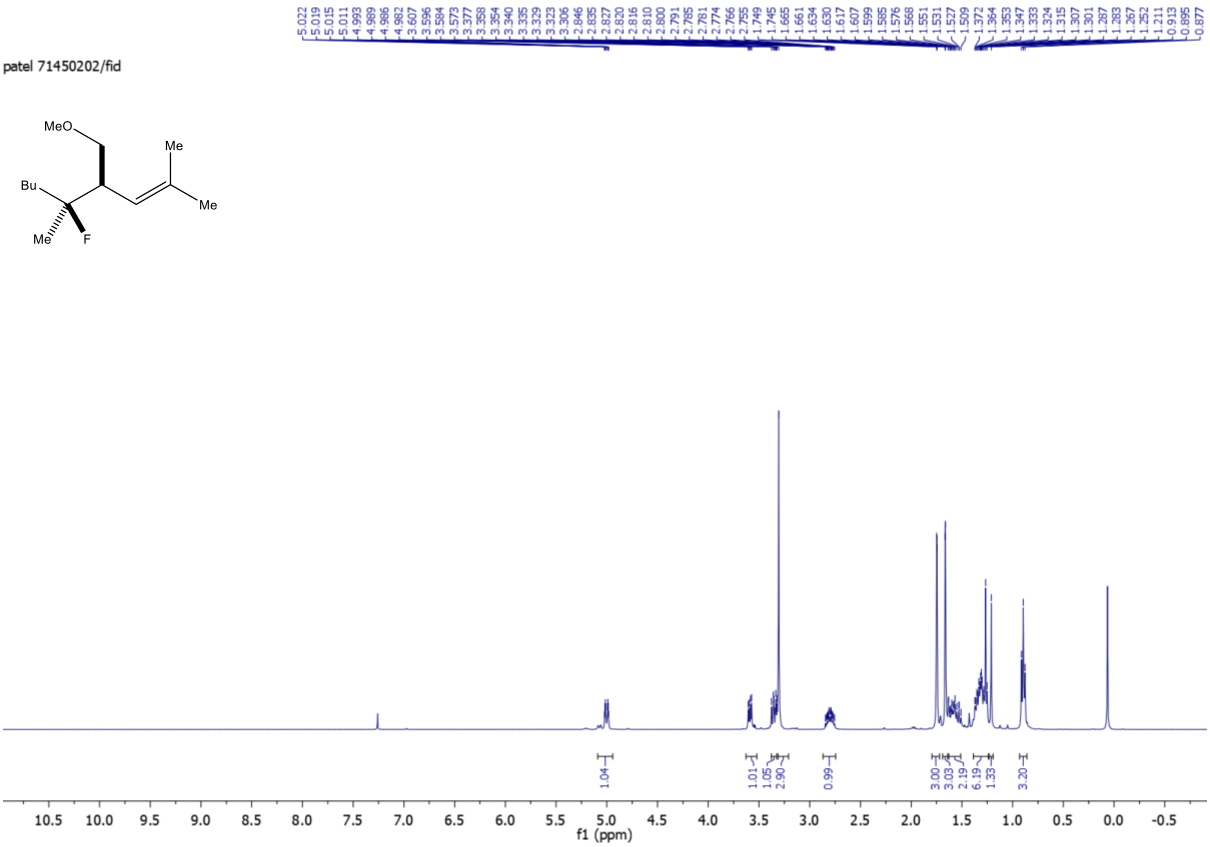
patel 71450202/fid
MeO
Me
Bu
Me
Me
F
5.022 5.019 5.015 5.011 4.993 4.989 4.986 4.982 3.607 3.596 3.584 3.573 3.377 3.358 3.354 3.340 3.335 3.329 3.323 3.306 2.846 2.835 2.827 2.820 2.816 2.810 2.800 2.791 2.785 2.781 2.774 2.766 2.755 1.749 1.745 1.665 1.661 1.634 1.630 1.617 1.607 1.599 1.585 1.576 1.568 1.551 1.531 1.527 1.509 1.372 1.364 1.353 1.347 1.333 1.324 1.315 1.307 1.301 1.287 1.283 1.267 1.252 1.211 0.913 0.895 0.877
1.04
1.01
1.05
2.90
0.99
3.00
3.03
2.19
6.19
1.33
3.20
10.5 10.0 9.5 9.0 8.5 8.0 7.5 7.0 6.5 6.0 5.5 5.0 4.5 4.0 3.5 3.0 2.5 2.0 1.5 1.0 0.5 0.0 -0.5
f1 (ppm)

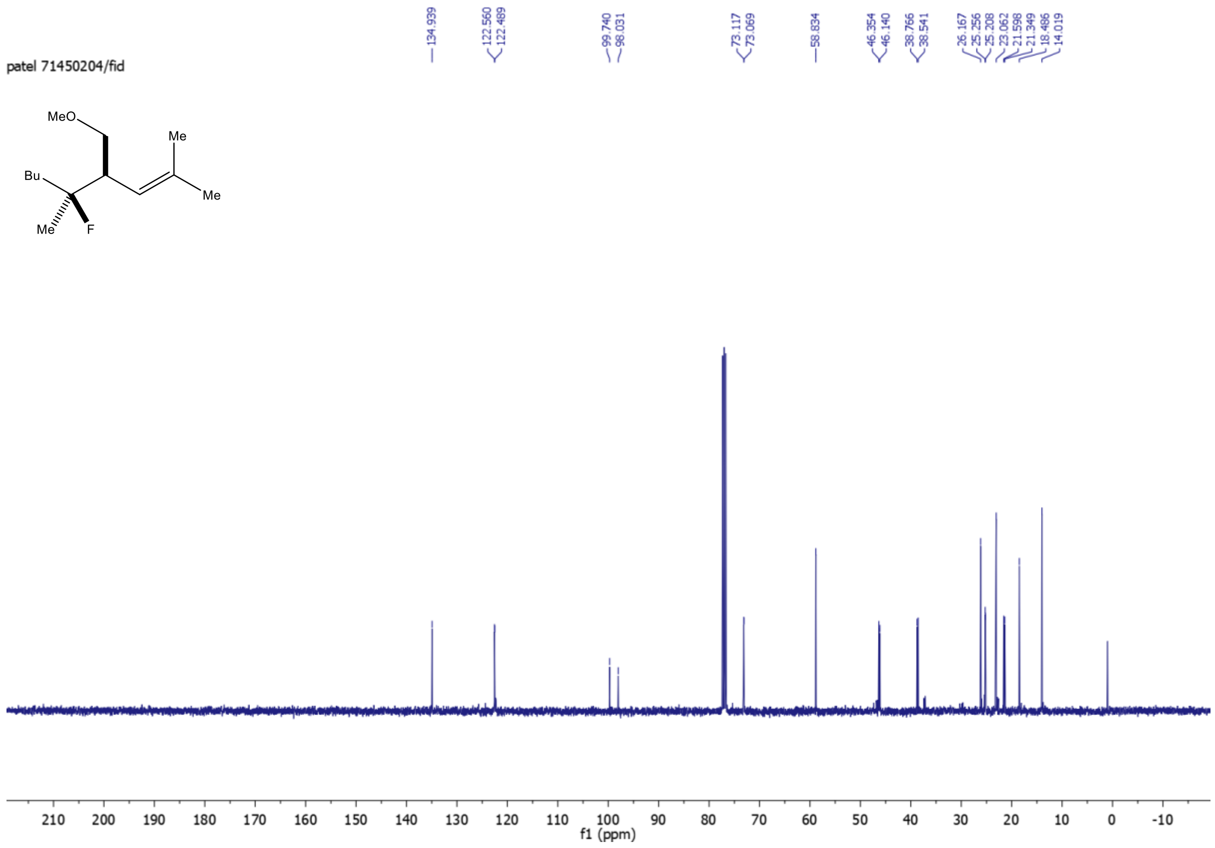
patel 71450204/fid
MeO
Me
Bu
Me
Me
F
134.939
122.560
122.489
99.740
98.031
73.117
73.069
58.834
46.354
46.140
38.766
38.541
26.167
25.256
25.208
23.062
21.598
21.349
18.486
14.019
f1 (ppm)

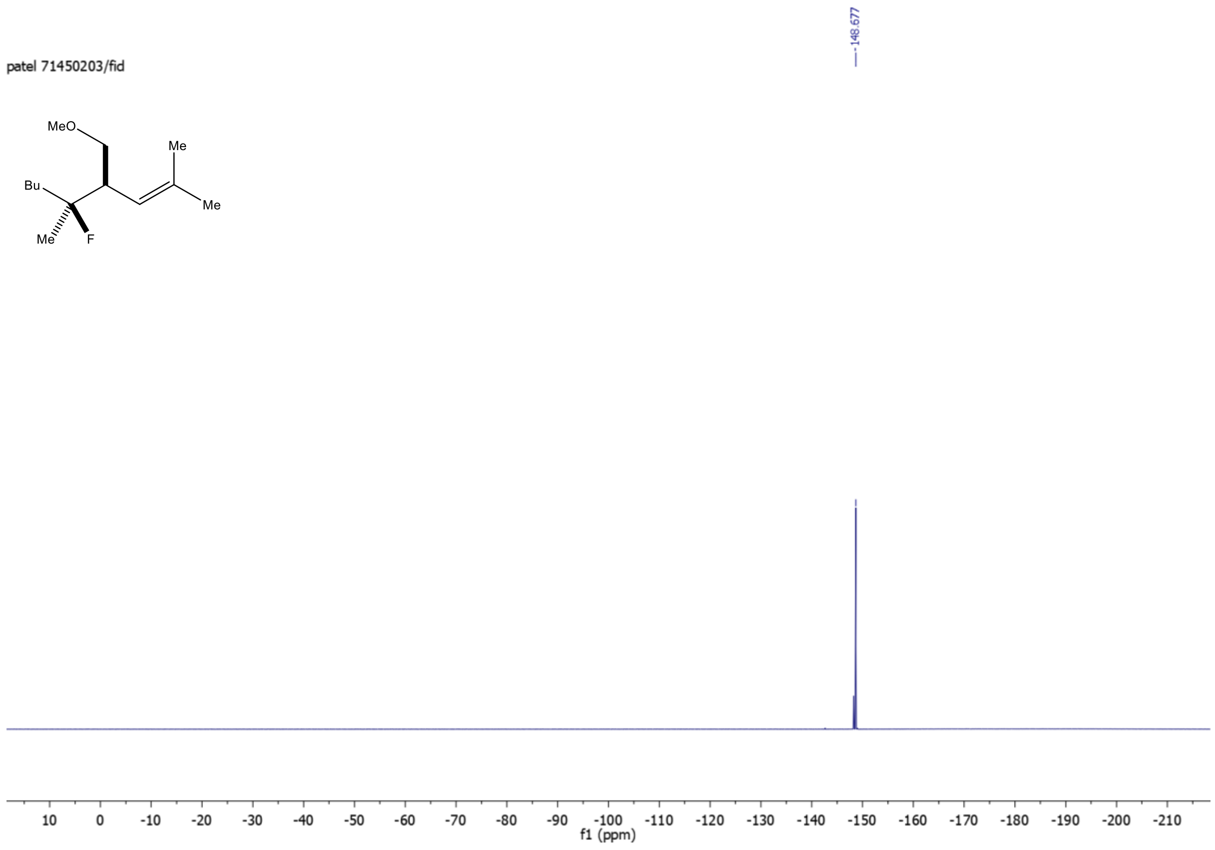
patel 71450203/fid
MeO
Me
Bu
Me
Me
F
-148.677
f1 (ppm)

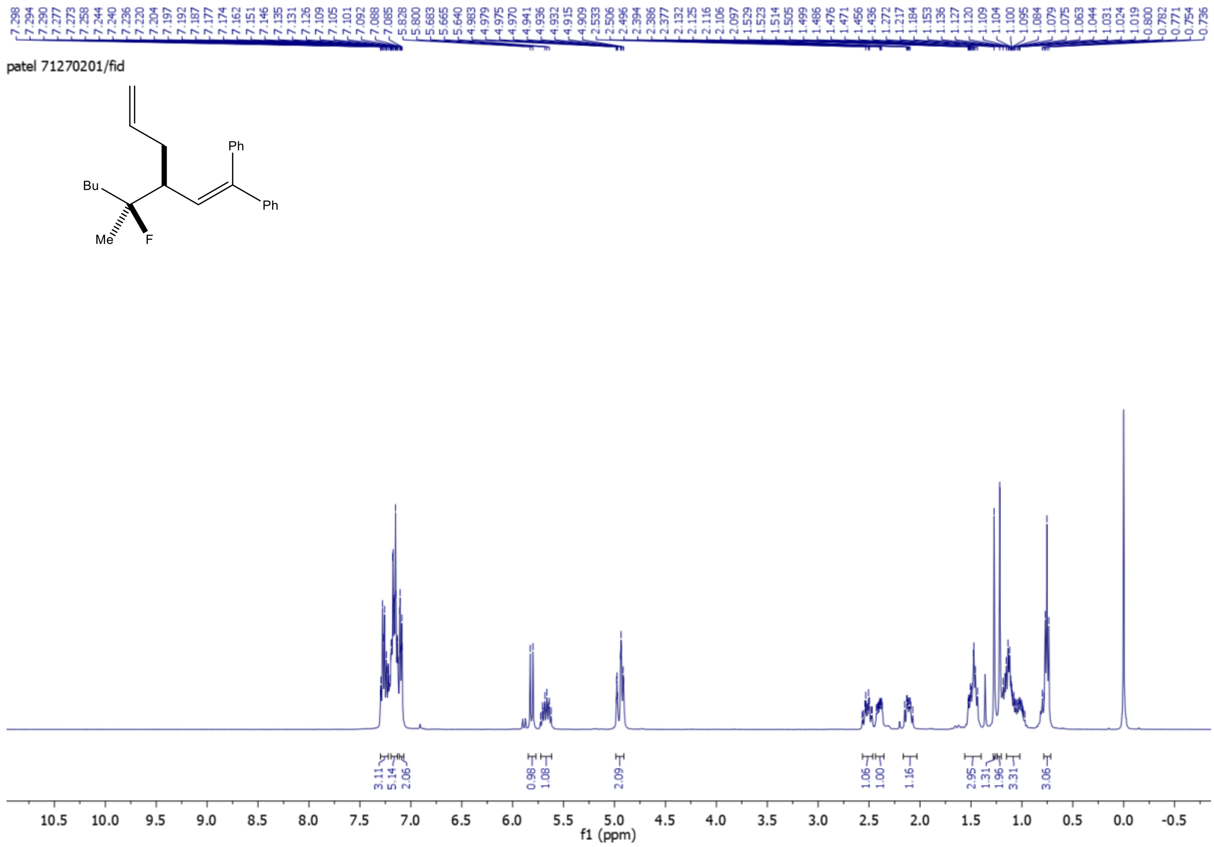
7.298 7.294 7.290 7.277 7.273 7.258 7.244 7.240 7.236 7.220 7.204 7.197 7.192 7.187 7.177 7.174 7.162 7.151 7.146 7.135 7.131 7.126 7.109 7.105 7.101 7.092 7.088 7.085 5.828 5.800 5.683 5.665 5.640 4.983 4.979 4.975 4.970 4.941 4.936 4.932 4.915 4.909 2.533 2.506 2.496 2.394 2.386 2.377 2.132 2.125 2.116 2.106 2.097 1.529 1.523 1.514 1.505 1.499 1.486 1.476 1.471 1.456 1.436 1.272 1.217 1.184 1.153 1.136 1.127 1.120 1.109 1.104 1.100 1.095 1.084 1.079 1.075 1.063 1.044 1.031 1.024 1.019 0.800 0.782 0.771 0.754 0.736
patel 71270201/fid
Bu
Me
F
Ph
Ph
3.11 5.14 2.06 0.98 1.08 2.09 1.06 1.00 1.16 2.95 1.31 1.96 3.31 3.06
10.5 10.0 9.5 9.0 8.5 8.0 7.5 7.0 6.5 6.0 5.5 5.0 4.5 4.0 3.5 3.0 2.5 2.0 1.5 1.0 0.5 0.0 -0.5
f1 (ppm)

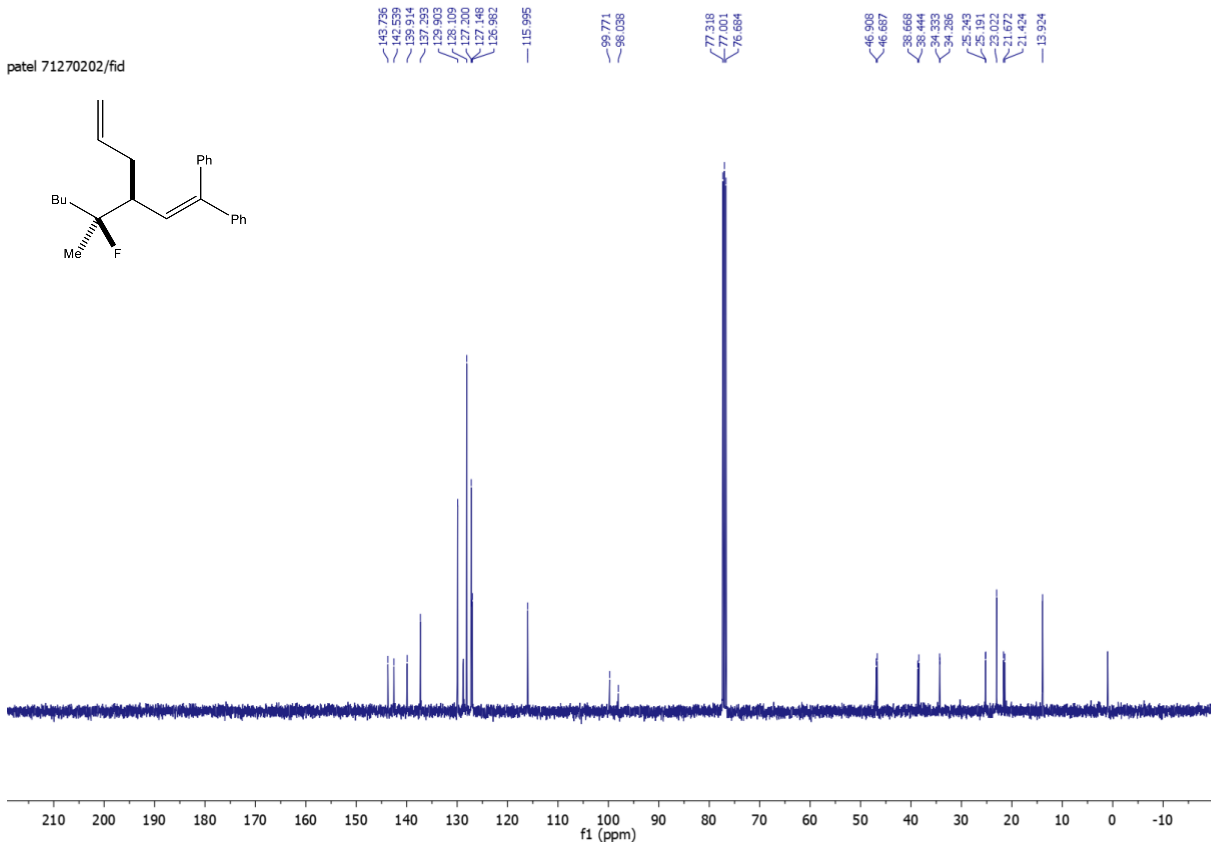
143.736 142.539 139.914 137.293 129.903 128.109 127.200 127.148 126.982 115.995 99.771 98.038 77.318 77.001 76.684 46.908 46.687 38.668 38.444 34.333 34.286 25.243 25.191 23.022 21.672 21.424 13.924
patel 71270202/fid
Bu
Me
F
Ph
Ph
210 200 190 180 170 160 150 140 130 120 110 100 90 80 70 60 50 40 30 20 10 0 -10
f1 (ppm)

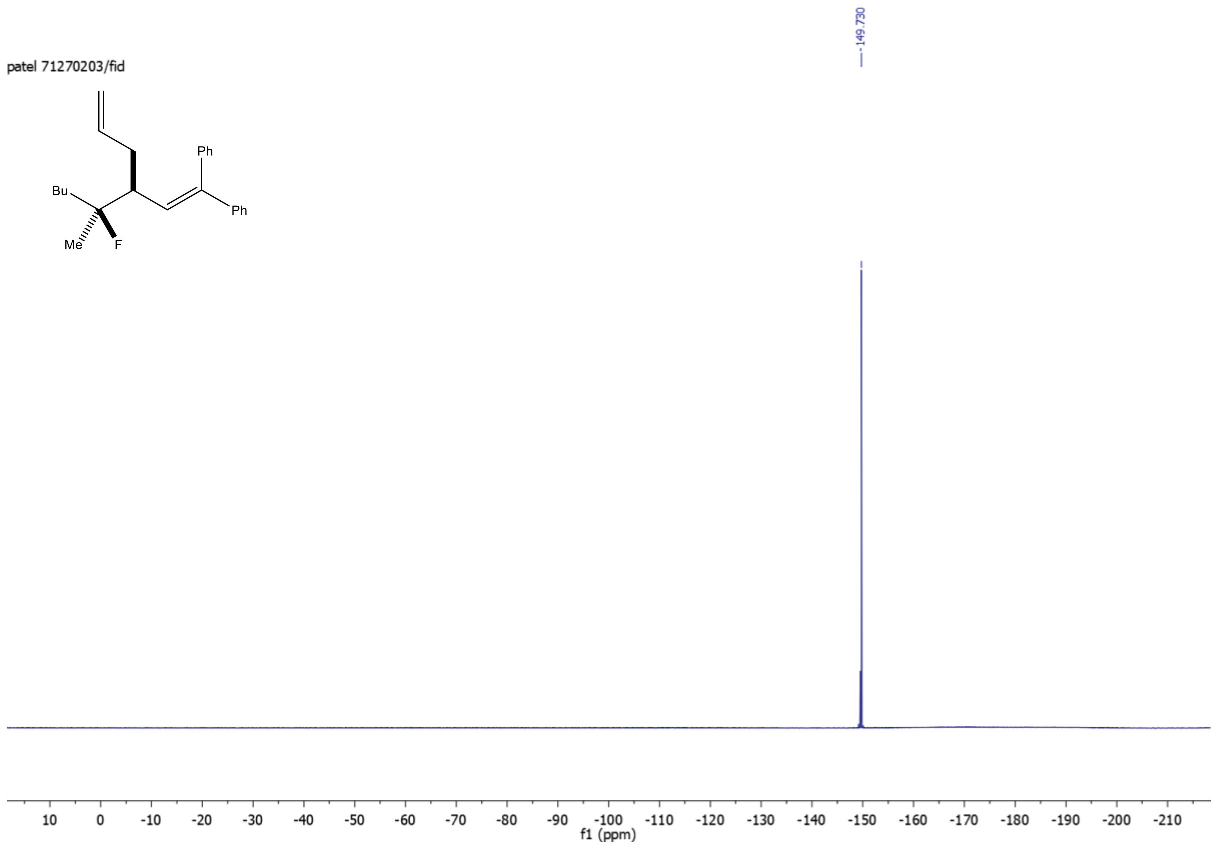

patel 71270203/fid
-149.730
Ph
Ph
Bu
Me
F
f1 (ppm)

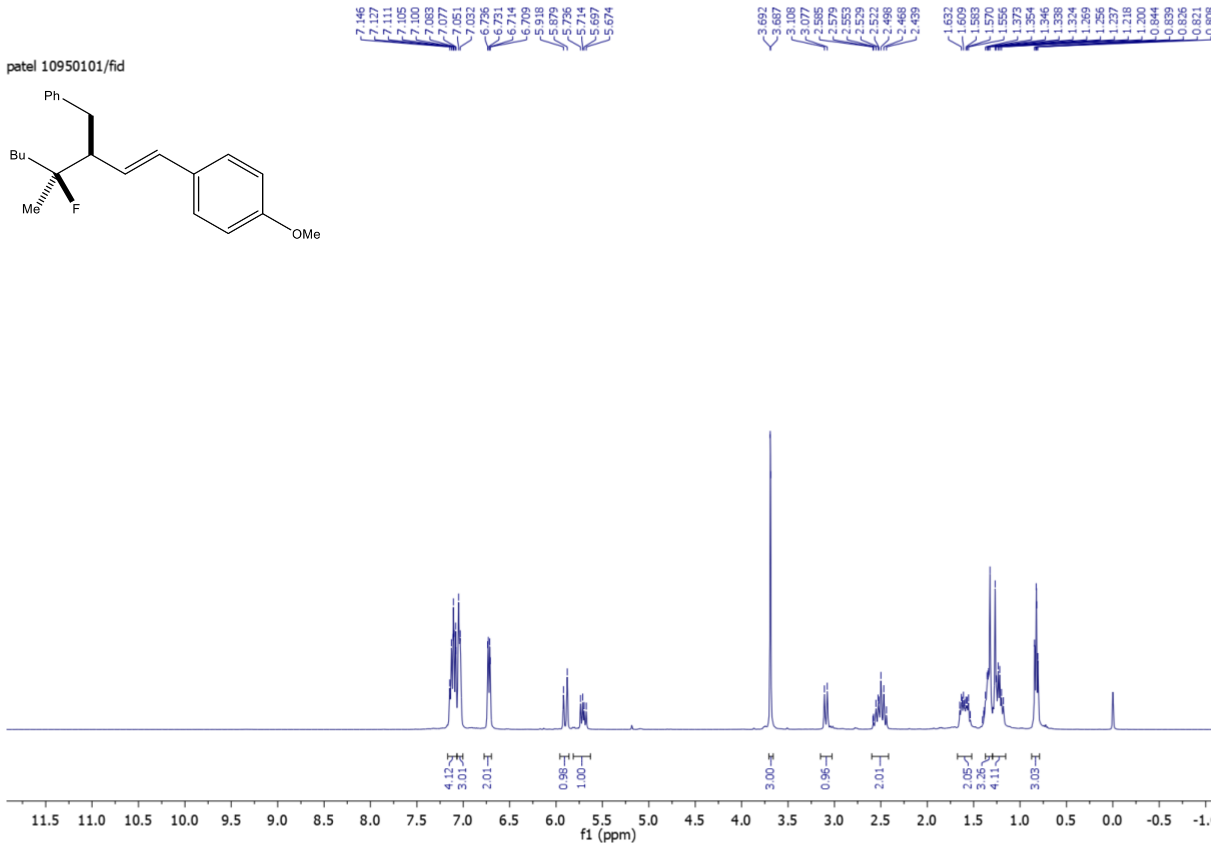

patel 10950101/fid
7.146
7.127
7.111
7.105
7.100
7.083
7.077
7.051
7.032
6.736
6.731
6.714
6.709
5.918
5.879
5.736
5.714
5.697
5.674
3.692
3.687
3.108
3.077
2.585
2.579
2.553
2.529
2.522
2.498
2.468
2.439
1.632
1.609
1.583
1.570
1.556
1.373
1.354
1.346
1.338
1.324
1.269
1.256
1.237
1.218
1.200
0.844
0.839
0.826
0.821
Ph
Bu
Me
F
OMe
4.12
3.01
2.01
0.98
1.00
3.00
0.96
2.01
2.05
3.26
4.11
3.03
f1 (ppm)

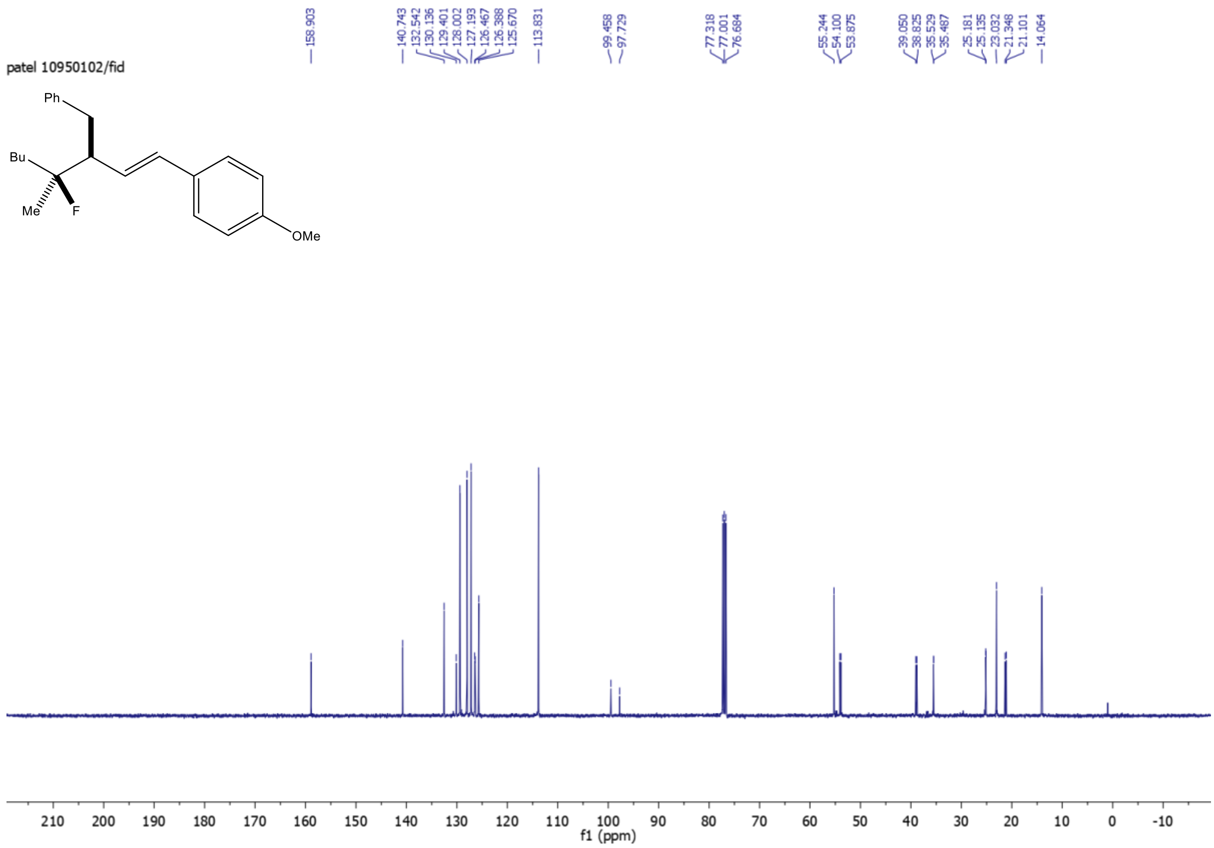
patel 10950102/fid
158.903
140.743
132.542
130.136
129.401
128.002
127.193
126.467
126.388
125.670
113.831
99.458
97.729
77.318
77.001
76.684
55.244
54.100
53.875
39.050
38.825
35.529
35.487
25.181
25.135
23.032
21.348
21.101
14.064
Ph
Bu
Me
F
OMe
f1 (ppm)

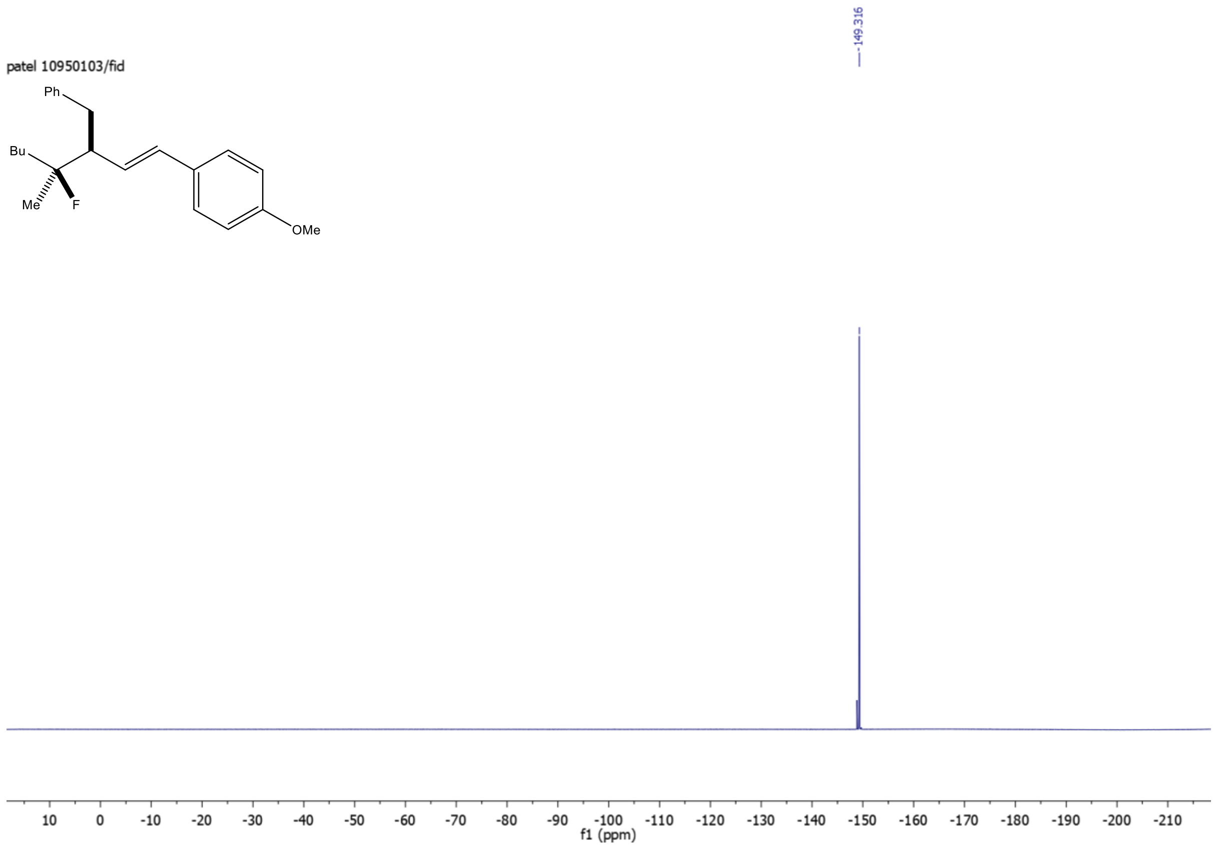
patel 10950103/fid
-149.316
Ph
Bu
Me
F
OMe
f1 (ppm)

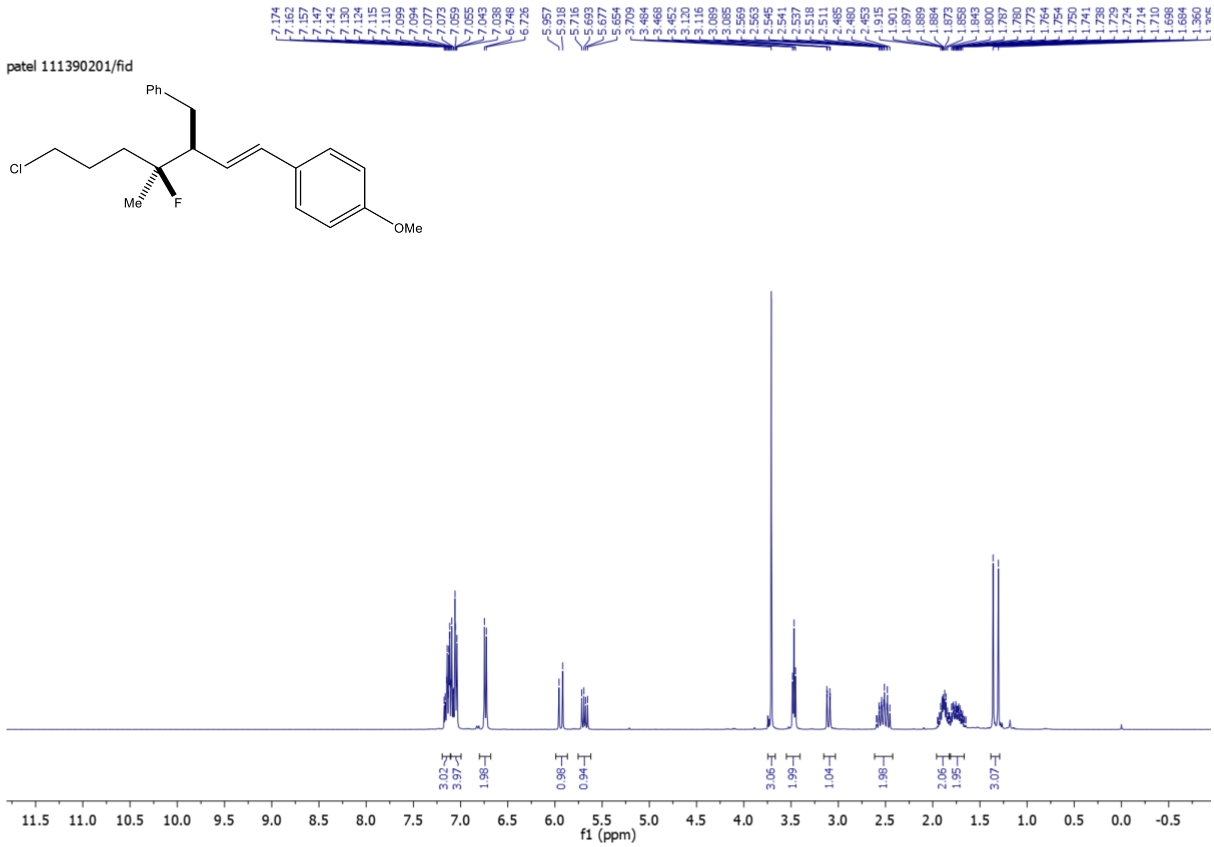
7.174
7.162
7.157
7.147
7.142
7.130
7.124
7.115
7.110
7.099
7.094
7.077
7.073
7.059
7.055
7.043
7.038
6.748
6.726
5.957
5.918
5.716
5.693
5.677
5.654
3.709
3.484
3.468
3.452
3.120
3.116
3.089
3.085
2.569
2.563
2.545
2.541
2.537
2.518
2.511
2.485
2.480
2.453
1.915
1.901
1.897
1.889
1.884
1.873
1.858
1.843
1.800
1.787
1.780
1.773
1.764
1.754
1.750
1.741
1.738
1.729
1.724
1.714
1.710
1.698
1.684
1.360
patel 111390201/fid
Ph
Cl
Me
F
OMe
3.02
3.97
1.98
0.98
0.94
3.06
1.99
1.04
1.98
2.06
1.95
3.07
f1 (ppm)

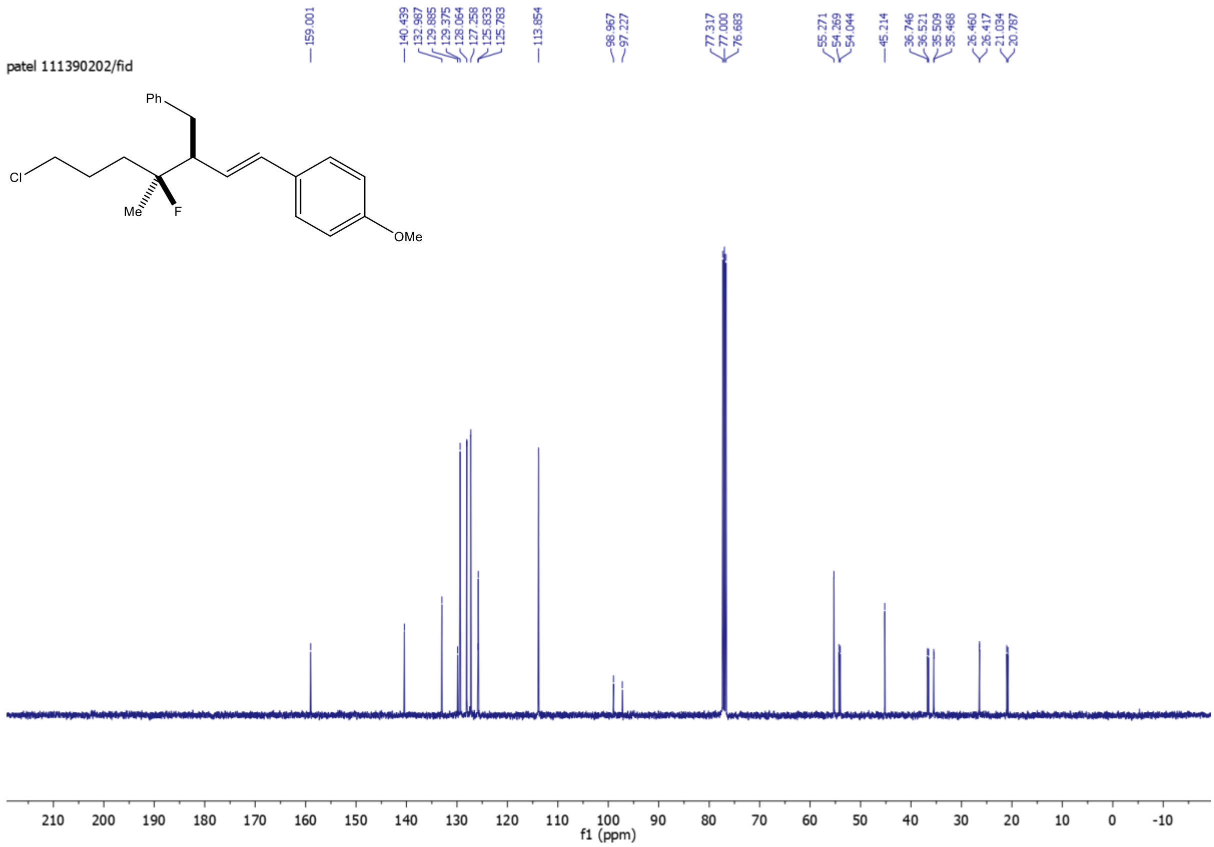
159.001
140.439
132.987
129.885
129.375
128.064
127.258
125.833
125.783
113.854
98.967
97.227
77.317
77.000
76.683
55.271
54.269
54.044
45.214
36.746
36.521
35.509
35.468
26.460
26.417
21.034
20.787
patel 111390202/fid
Ph
Cl
Me
F
OMe
f1 (ppm)

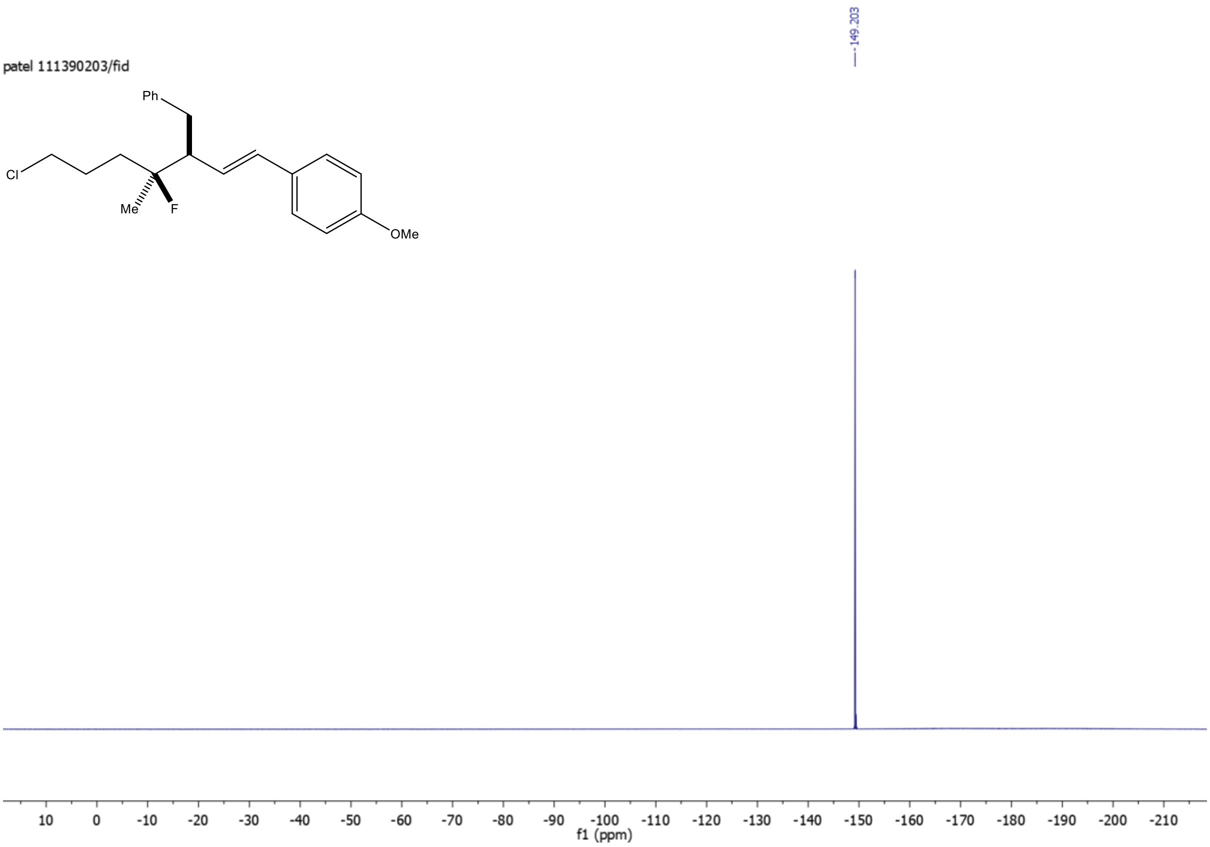
patel 111390203/fid
Ph
Cl
Me
F
OMe
-149.203
10 0 -10 -20 -30 -40 -50 -60 -70 -80 -90 -100 -110 -120 -130 -140 -150 -160 -170 -180 -190 -200 -210
f1 (ppm)

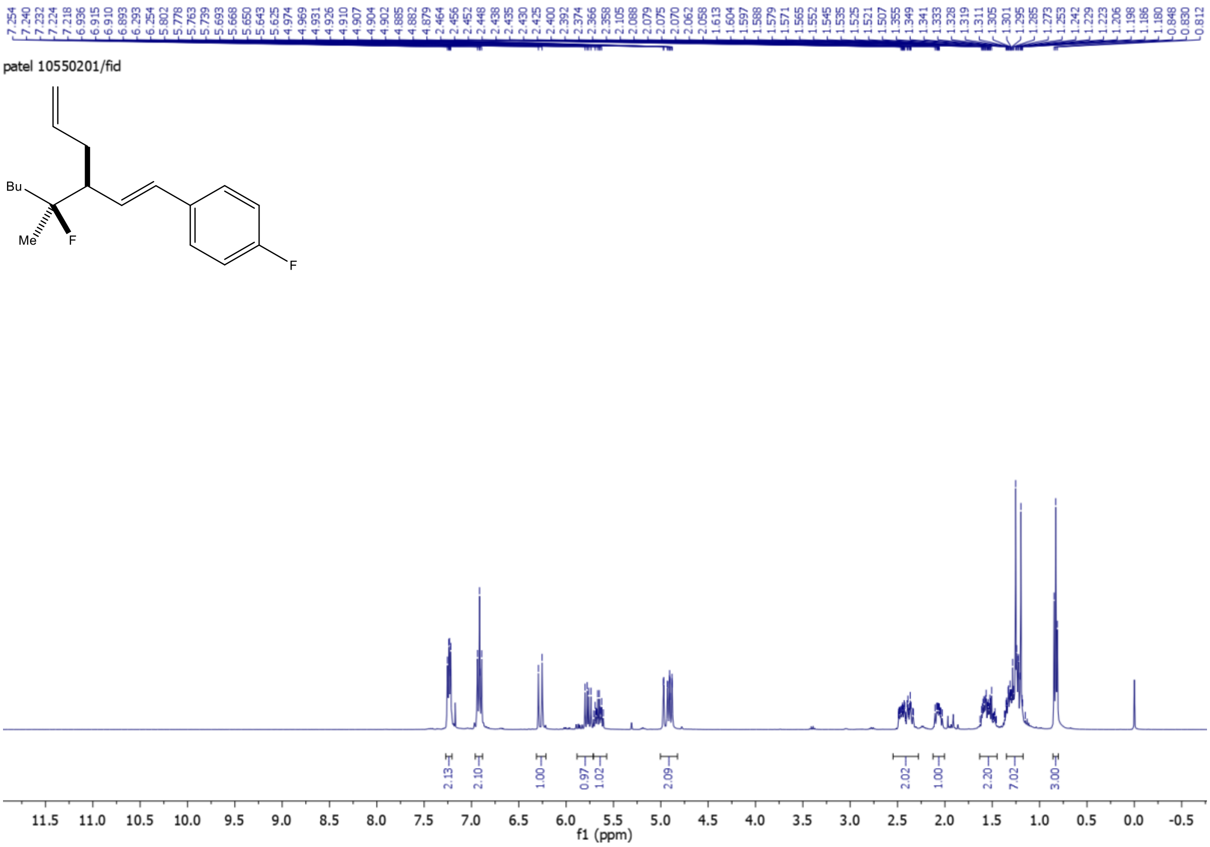
7.254 7.240 7.232 7.224 7.218 6.936 6.915 6.910 6.893 6.293 6.254 5.802 5.778 5.763 5.739 5.693 5.668 5.650 5.643 5.625 4.974 4.969 4.931 4.926 4.910 4.907 4.904 4.902 4.885 4.882 4.879 2.464 2.456 2.452 2.448 2.438 2.435 2.430 2.425 2.400 2.392 2.374 2.366 2.358 2.105 2.088 2.079 2.075 2.070 2.062 2.058 1.613 1.604 1.597 1.588 1.579 1.571 1.565 1.552 1.545 1.535 1.525 1.521 1.507 1.355 1.349 1.341 1.333 1.328 1.319 1.311 1.305 1.301 1.295 1.285 1.273 1.253 1.242 1.229 1.223 1.206 1.198 1.186 1.180 0.848 0.830 0.812
patel 10550201/fid
Bu
Me
F
F
2.13 2.10 1.00 0.97 1.02 2.09 2.02 1.00 2.20 7.02 3.00
11.5 11.0 10.5 10.0 9.5 9.0 8.5 8.0 7.5 7.0 6.5 6.0 5.5 5.0 4.5 4.0 3.5 3.0 2.5 2.0 1.5 1.0 0.5 0.0 -0.5
f1 (ppm)

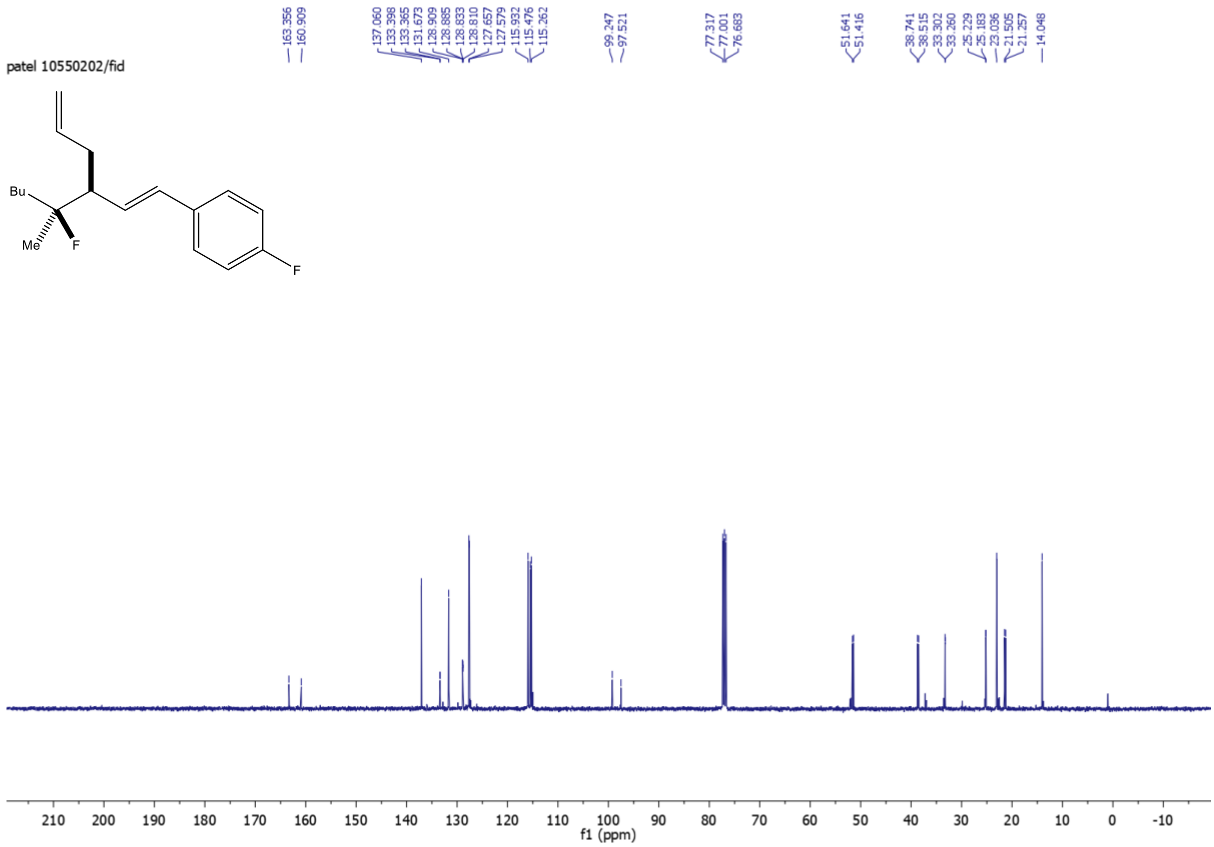
patel 10550202/fid
Bu
Me
F
F
163.356
160.909
137.060
133.398
133.365
131.673
128.909
128.885
128.833
128.810
127.657
127.579
115.932
115.476
115.262
99.247
97.521
77.317
77.001
76.683
51.641
51.416
38.741
38.515
33.302
33.260
25.229
25.183
23.036
21.505
21.257
14.048
210
200
190
180
170
160
150
140
130
120
110
100
90
80
70
60
50
40
30
20
10
0
-10
f1 (ppm)

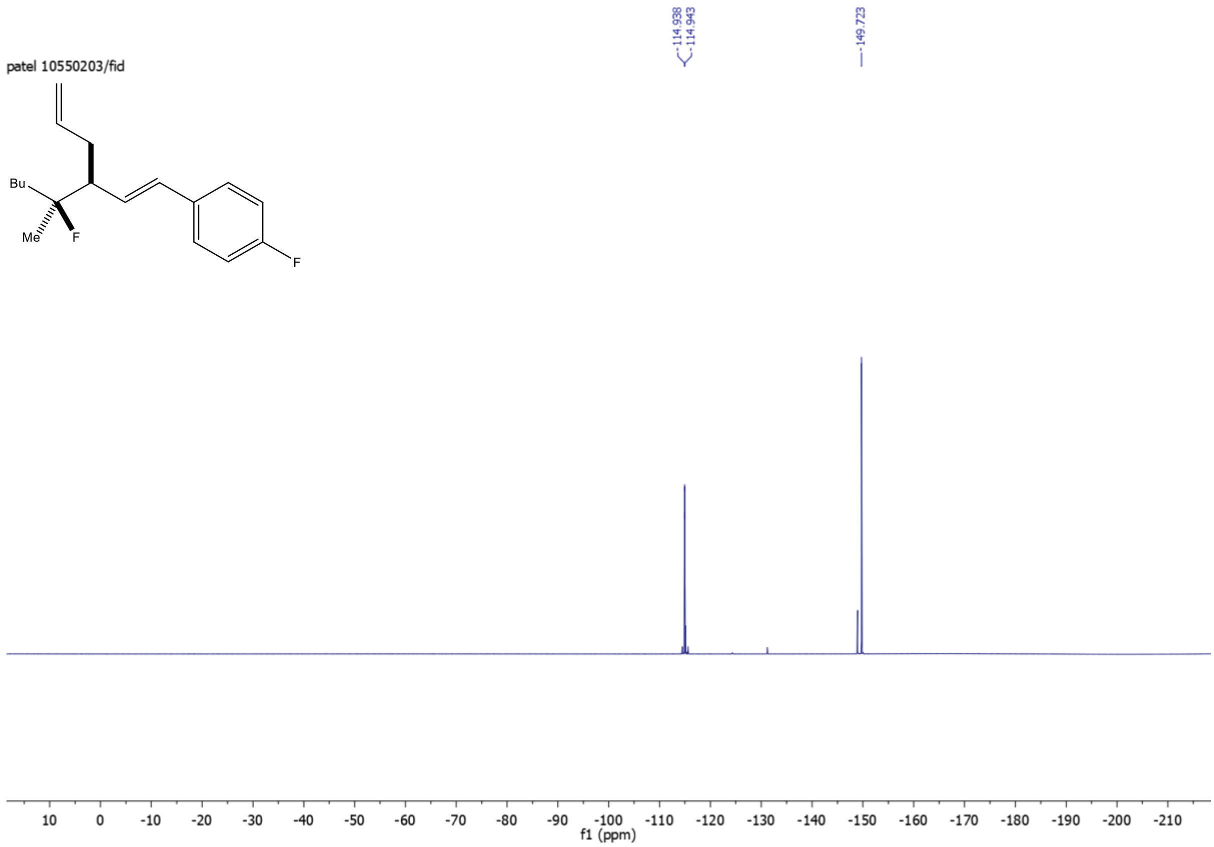
patel 10550203/fid
Bu
Me
F
F
-114.938
-114.943
-149.723
10
0
-10
-20
-30
-40
-50
-60
-70
-80
-90
-100
-110
-120
-130
-140
-150
-160
-170
-180
-190
-200
-210
f1 (ppm)

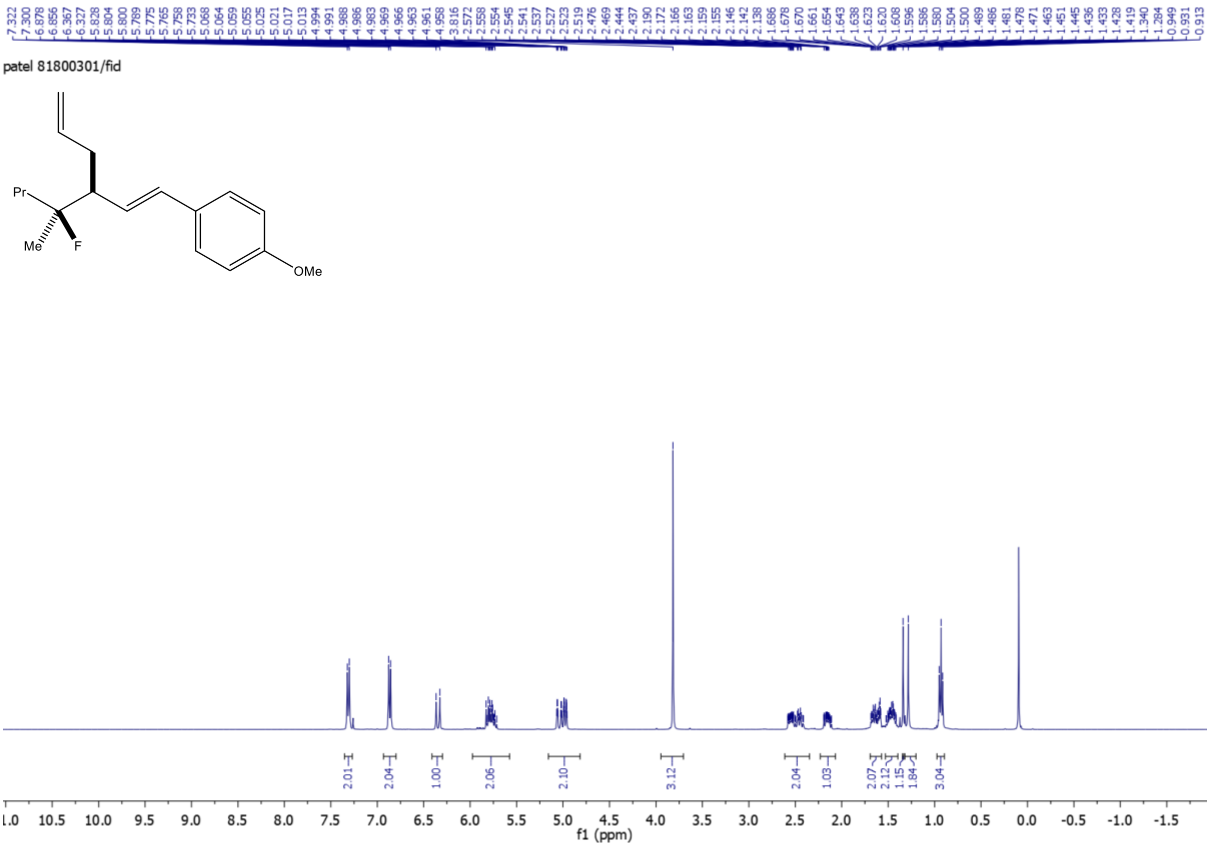
7.322
7.300
6.878
6.856
6.367
6.327
5.828
5.804
5.800
5.789
5.775
5.765
5.758
5.733
5.068
5.064
5.059
5.055
5.025
5.021
5.017
5.013
4.994
4.991
4.988
4.986
4.983
4.969
4.966
4.963
4.961
4.958
3.816
2.572
2.558
2.554
2.545
2.541
2.537
2.527
2.523
2.519
2.476
2.469
2.444
2.437
2.190
2.172
2.166
2.163
2.159
2.155
2.146
2.142
2.138
1.686
1.678
1.670
1.661
1.654
1.643
1.638
1.623
1.620
1.608
1.596
1.586
1.580
1.504
1.500
1.489
1.486
1.481
1.478
1.471
1.463
1.451
1.445
1.436
1.433
1.428
1.419
1.340
1.284
0.949
0.931
0.913
patel 81800301/fid
Pr
Me
F
OMe
2.01
2.04
1.00
2.06
2.10
3.12
2.04
1.03
2.07
2.12
1.15
1.84
3.04
f1 (ppm)

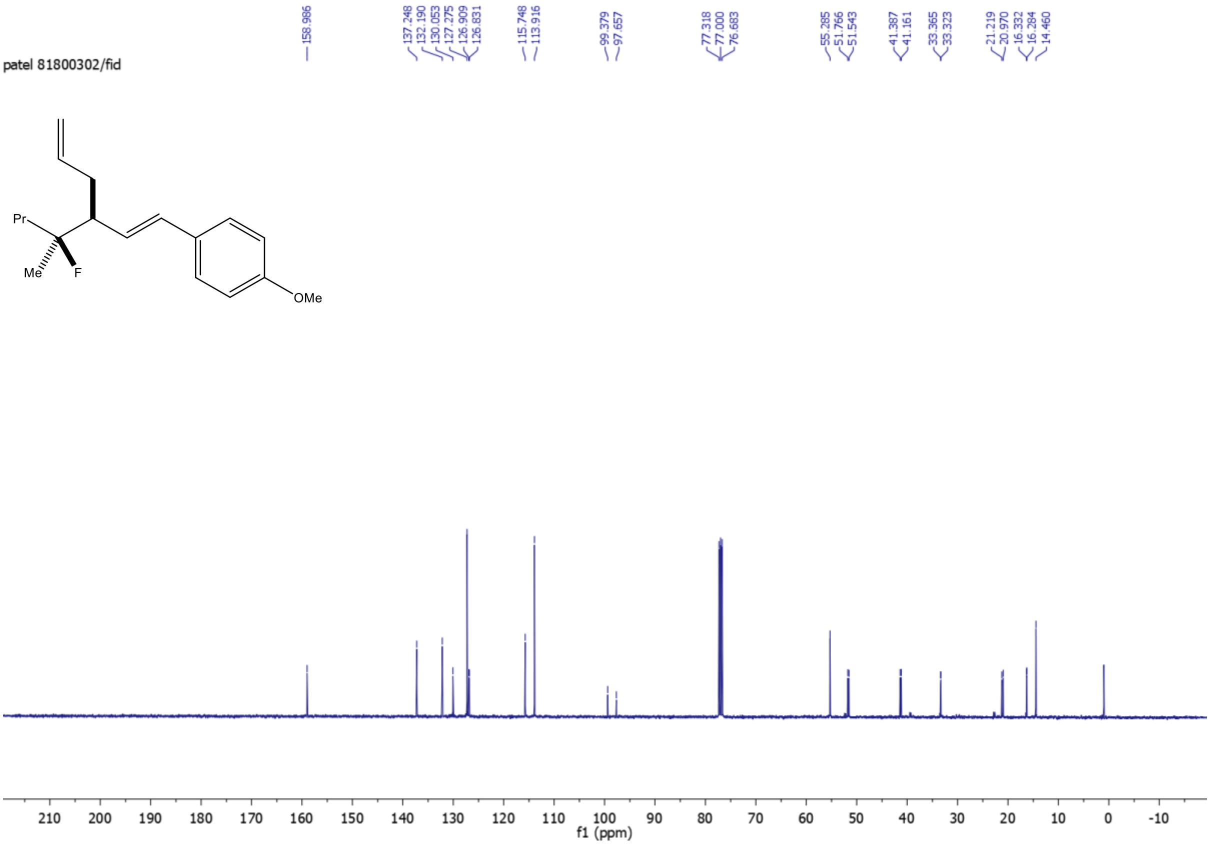
158.986
137.248
132.190
130.053
127.275
126.909
126.831
115.748
113.916
99.379
97.657
77.318
77.000
76.683
55.285
51.766
51.543
41.387
41.161
33.365
33.323
21.219
20.970
16.332
16.284
14.460
patel 81800302/fid
Pr
Me
F
OMe
f1 (ppm)

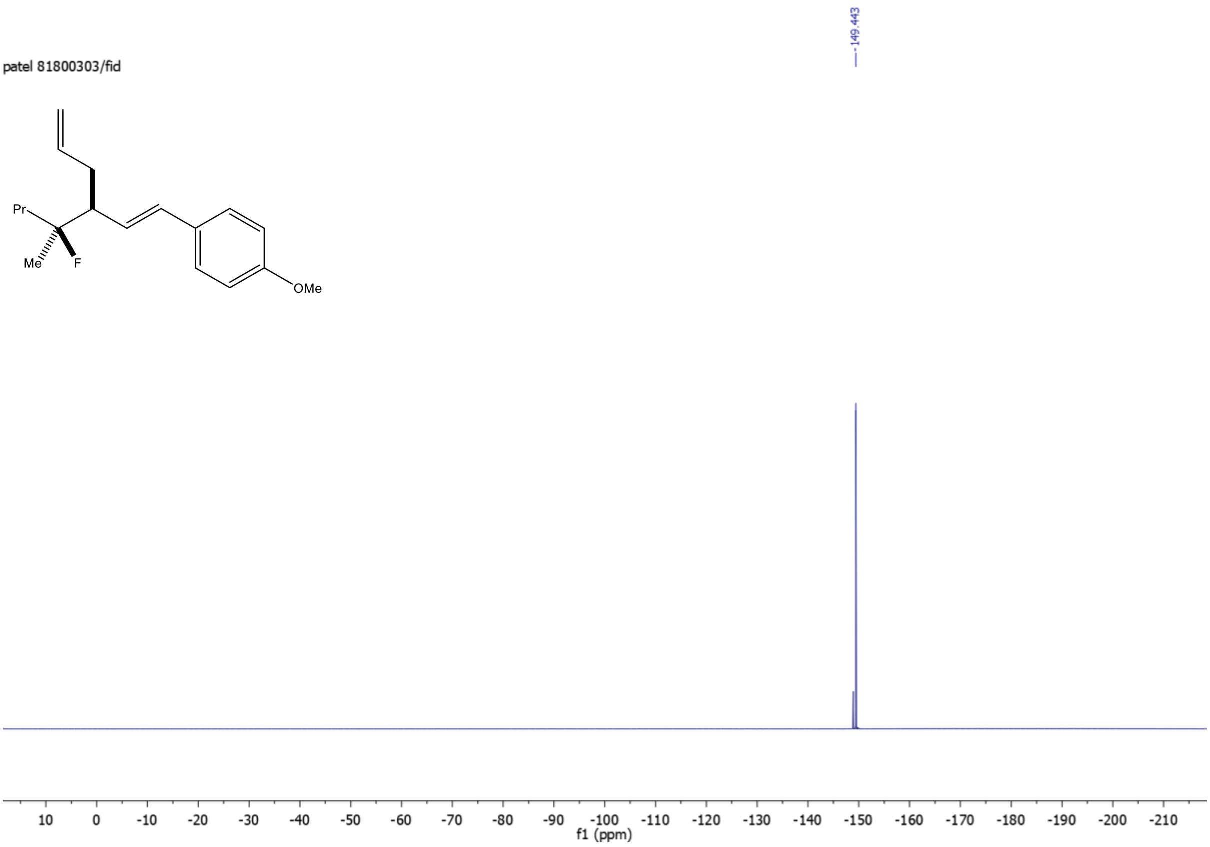
patel 81800303/fid
-149.443
Pr
Me
F
OMe
f1 (ppm)

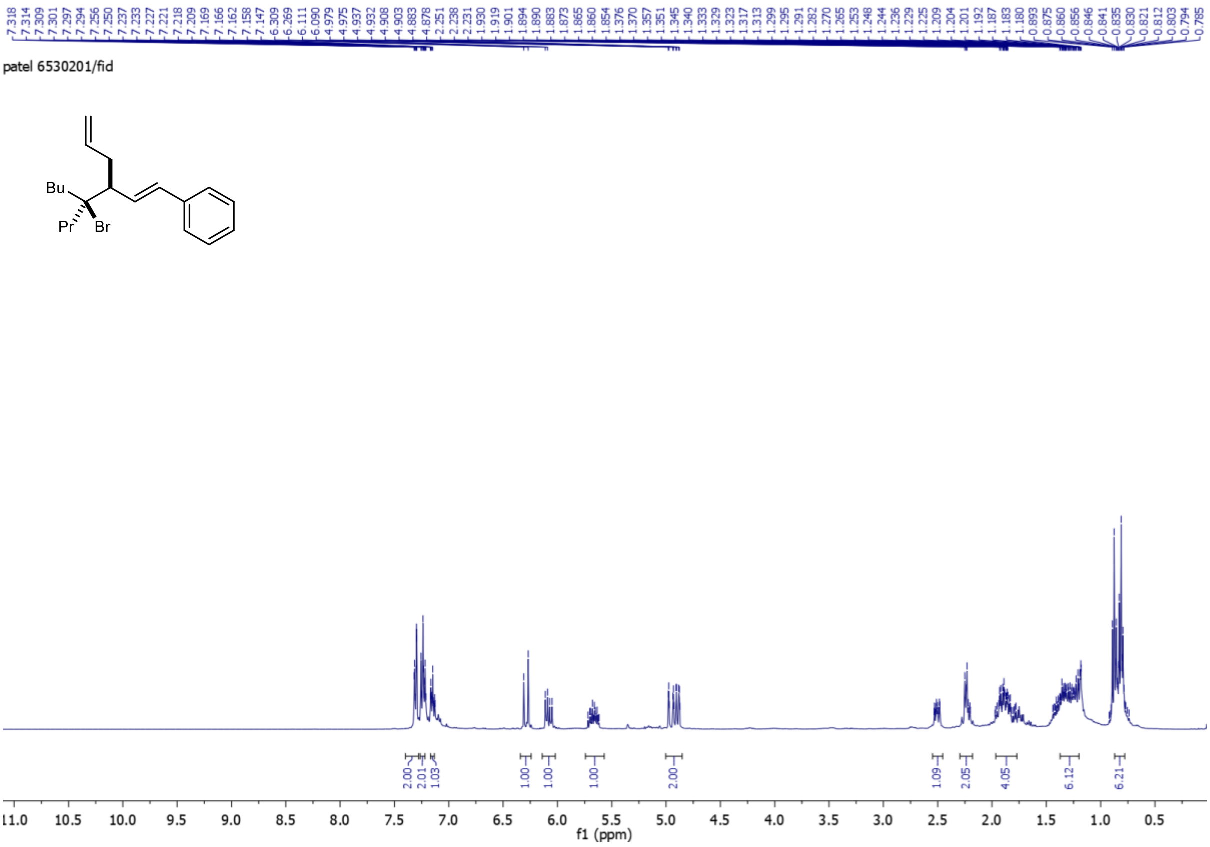
patel 6530201/fid
Bu
Pr
Br
f1 (ppm)

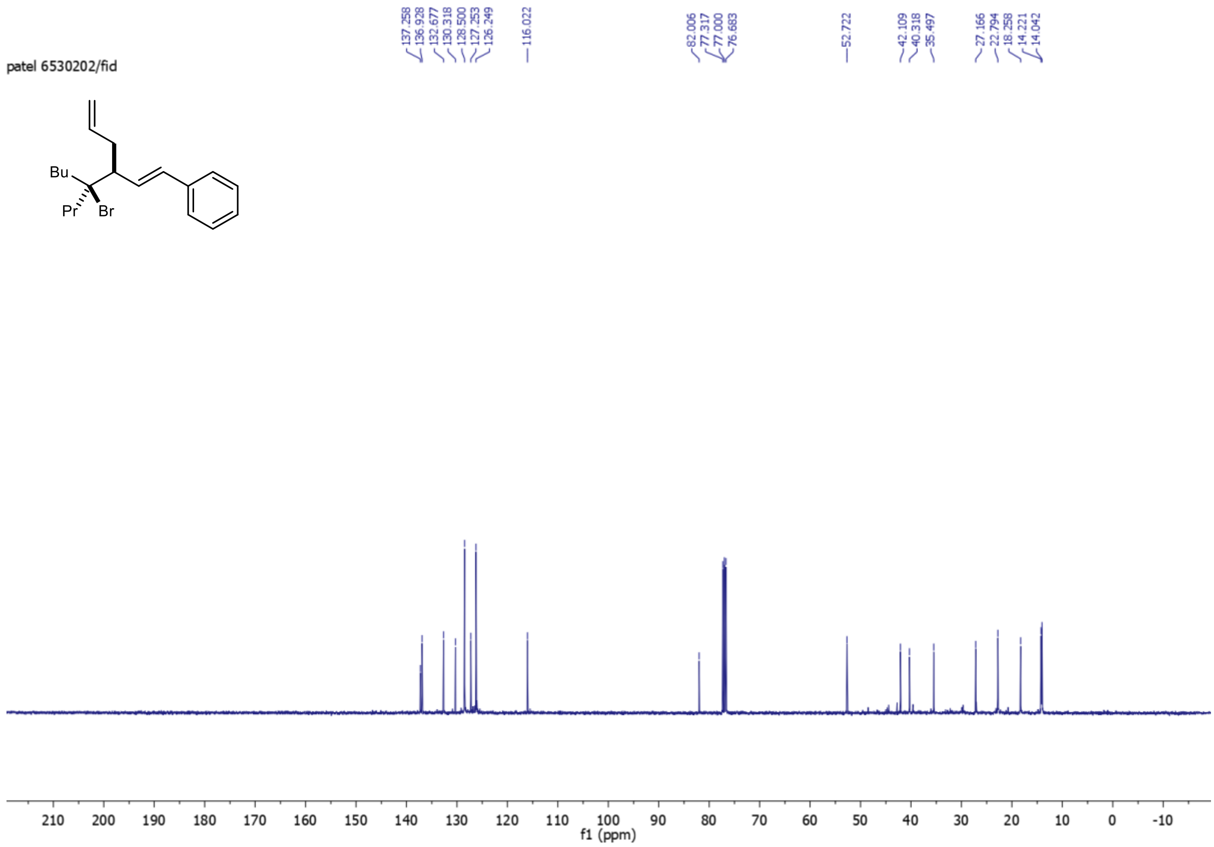
patel 6530202/fid
Bu
Pr
Br
137.258
136.928
132.677
130.318
128.500
127.253
126.249
116.022
82.006
77.317
77.000
76.683
52.722
42.109
40.318
35.497
27.166
22.794
18.258
14.221
14.042
210 200 190 180 170 160 150 140 130 120 110 100 90 80 70 60 50 40 30 20 10 0 -10
f1 (ppm)

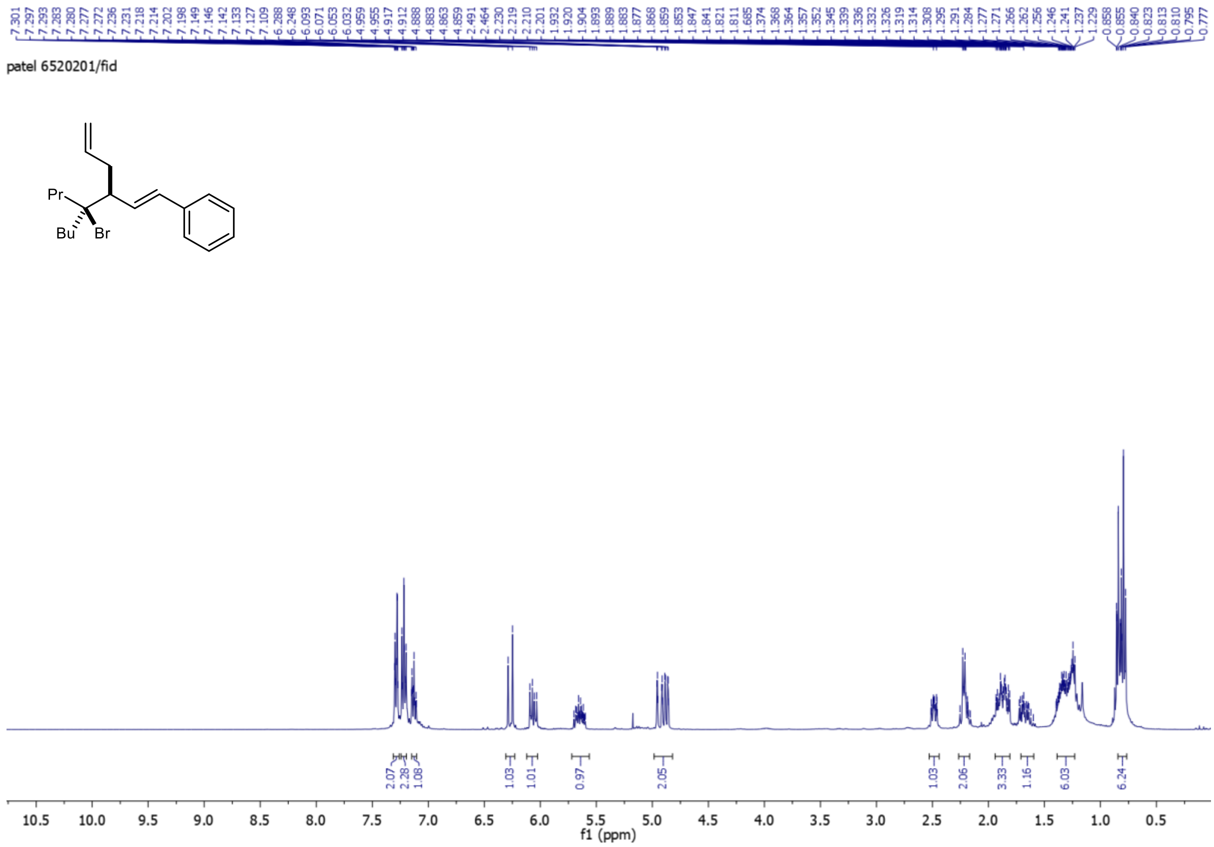
patel 6520201/fid
Pr
Bu
Br
7.301 7.297 7.293 7.283 7.280 7.277 7.272 7.236 7.231 7.218 7.214 7.202 7.198 7.149 7.146 7.142 7.133 7.127 7.109 6.288 6.248 6.093 6.071 6.053 6.032 4.959 4.955 4.917 4.912 4.888 4.883 4.863 4.859 2.491 2.464 2.230 2.219 2.210 2.201 1.932 1.920 1.904 1.893 1.889 1.883 1.877 1.868 1.859 1.853 1.847 1.841 1.821 1.811 1.685 1.374 1.368 1.364 1.357 1.352 1.345 1.339 1.336 1.332 1.326 1.319 1.314 1.308 1.295 1.291 1.284 1.277 1.271 1.266 1.262 1.256 1.246 1.241 1.237 1.229 0.858 0.855 0.840 0.823 0.813 0.810 0.795 0.777
2.07 2.28 1.08 1.03 1.01 0.97 2.05 1.03 2.06 3.33 1.16 6.03 6.24
10.5 10.0 9.5 9.0 8.5 8.0 7.5 7.0 6.5 6.0 5.5 5.0 4.5 4.0 3.5 3.0 2.5 2.0 1.5 1.0 0.5
f1 (ppm)

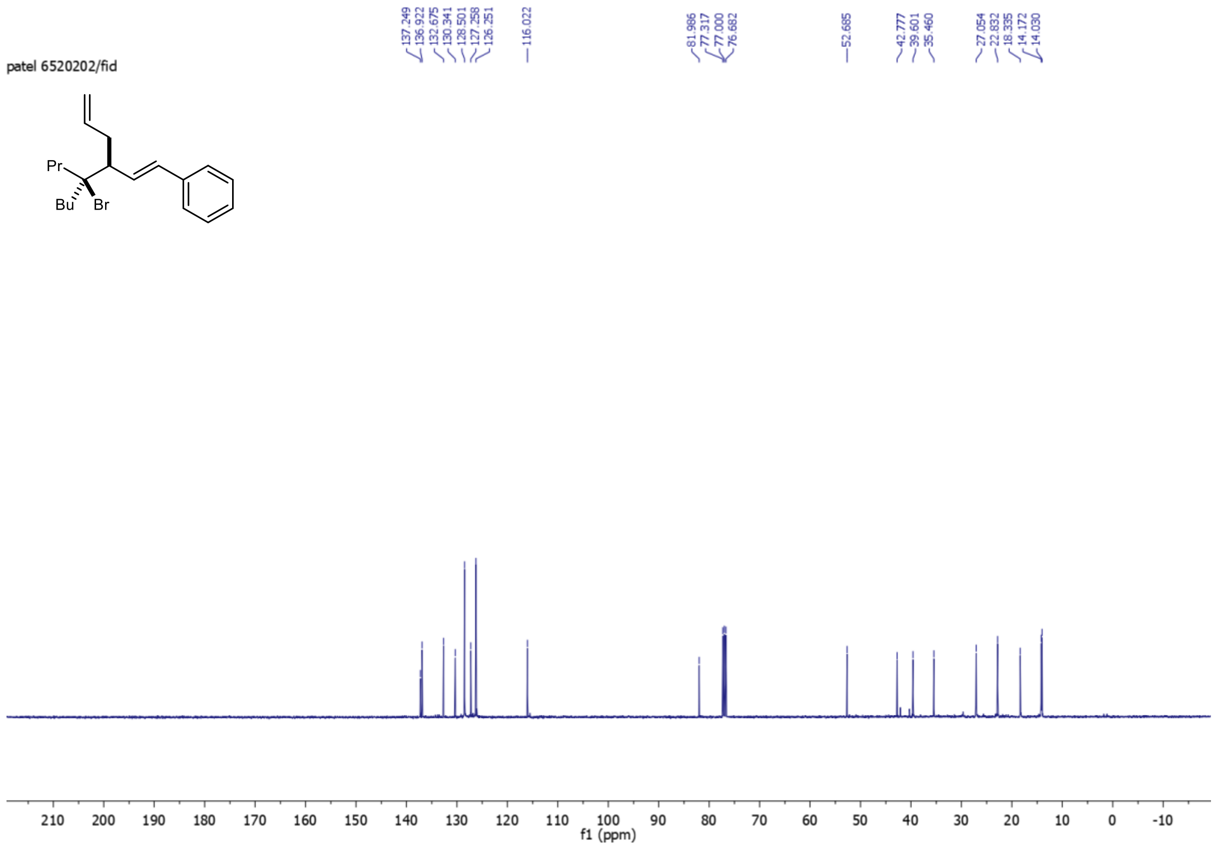
patel 6520202/fid
137.249
136.922
132.675
130.341
128.501
127.258
126.251
116.022
81.986
77.317
77.000
76.682
52.685
42.777
39.601
35.460
27.054
22.832
18.335
14.172
14.030
Pr
Bu
Br
f1 (ppm)

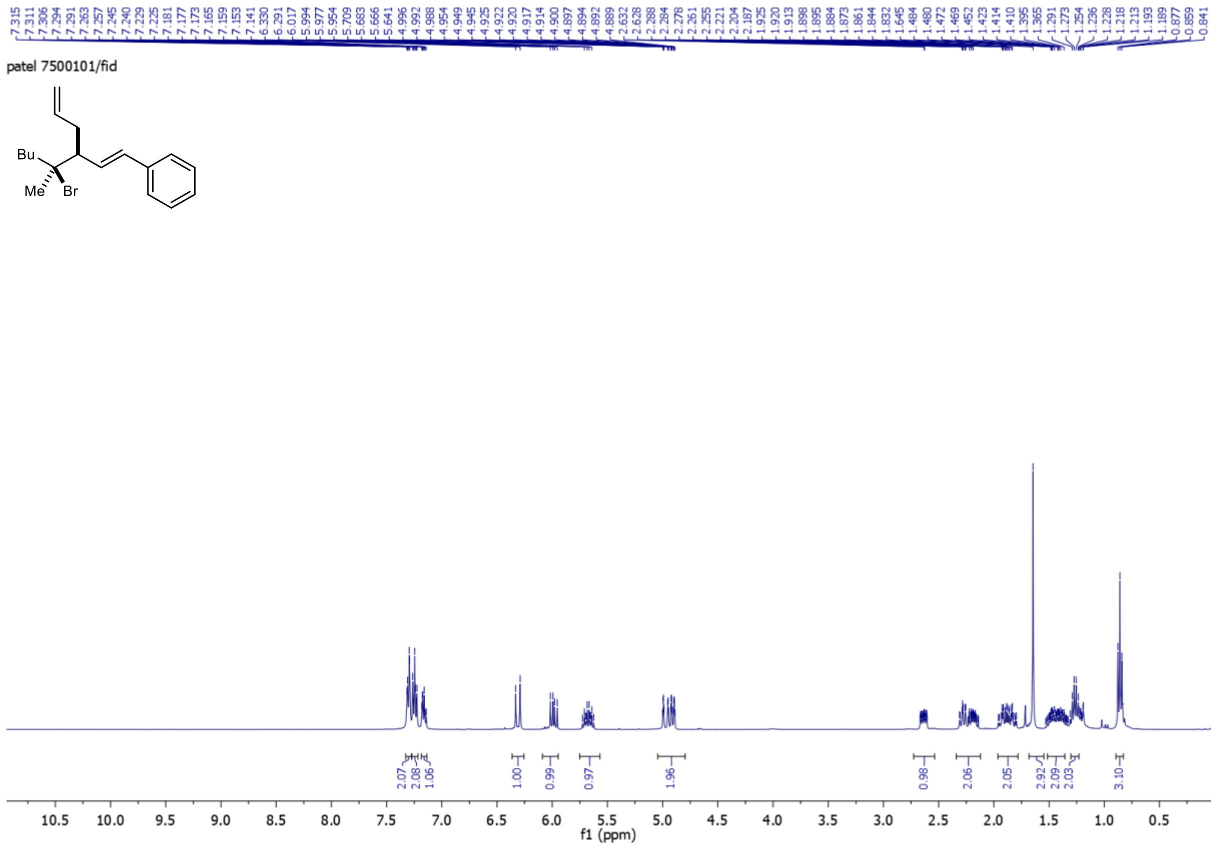
patel 7500101/fid
Bu
Me
Br
2.07
2.08
1.06
1.00
0.99
0.97
1.96
0.98
2.06
2.05
2.92
2.09
2.03
3.10
f1 (ppm)

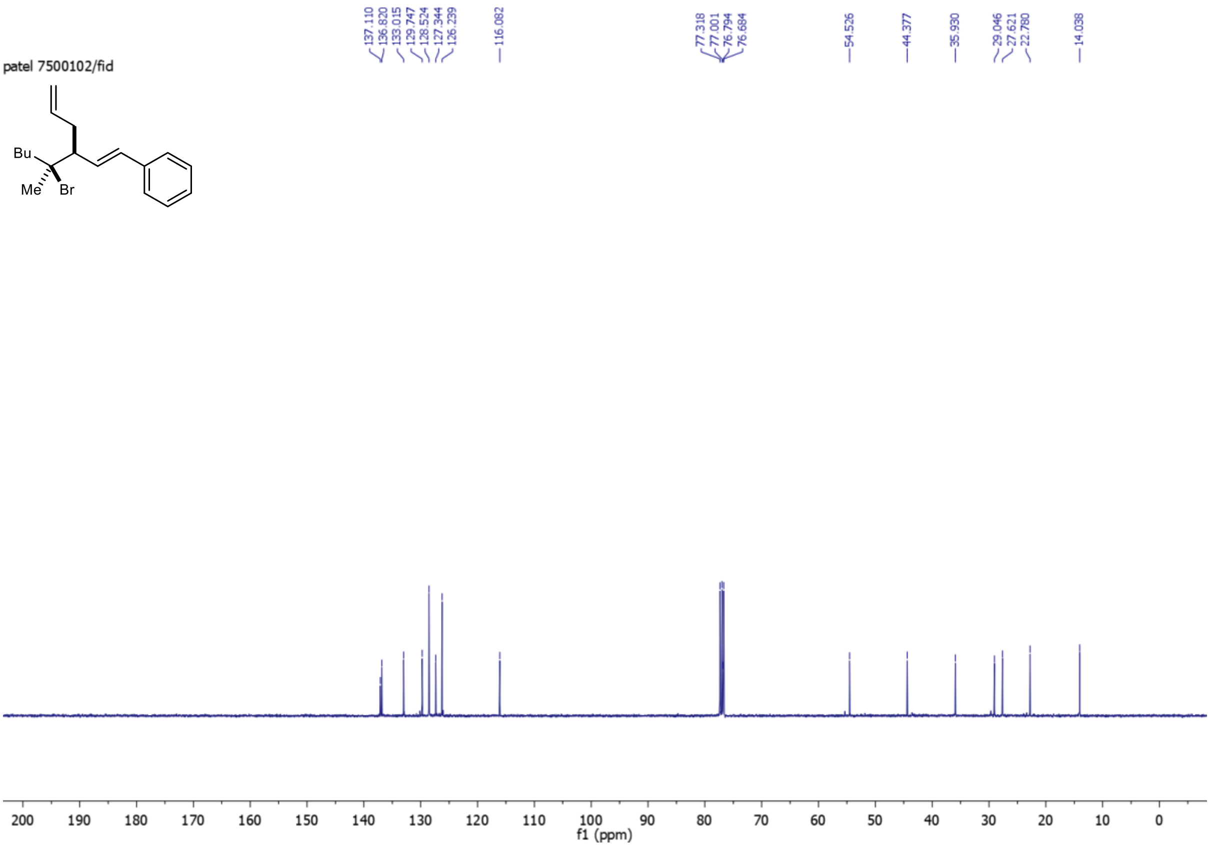
patel 7500102/fid
Bu
Me
Br
137.110
136.820
133.015
129.747
128.524
127.344
126.239
116.082
77.318
77.001
76.794
76.684
54.526
44.377
35.930
29.046
27.621
22.780
14.038
f1 (ppm)

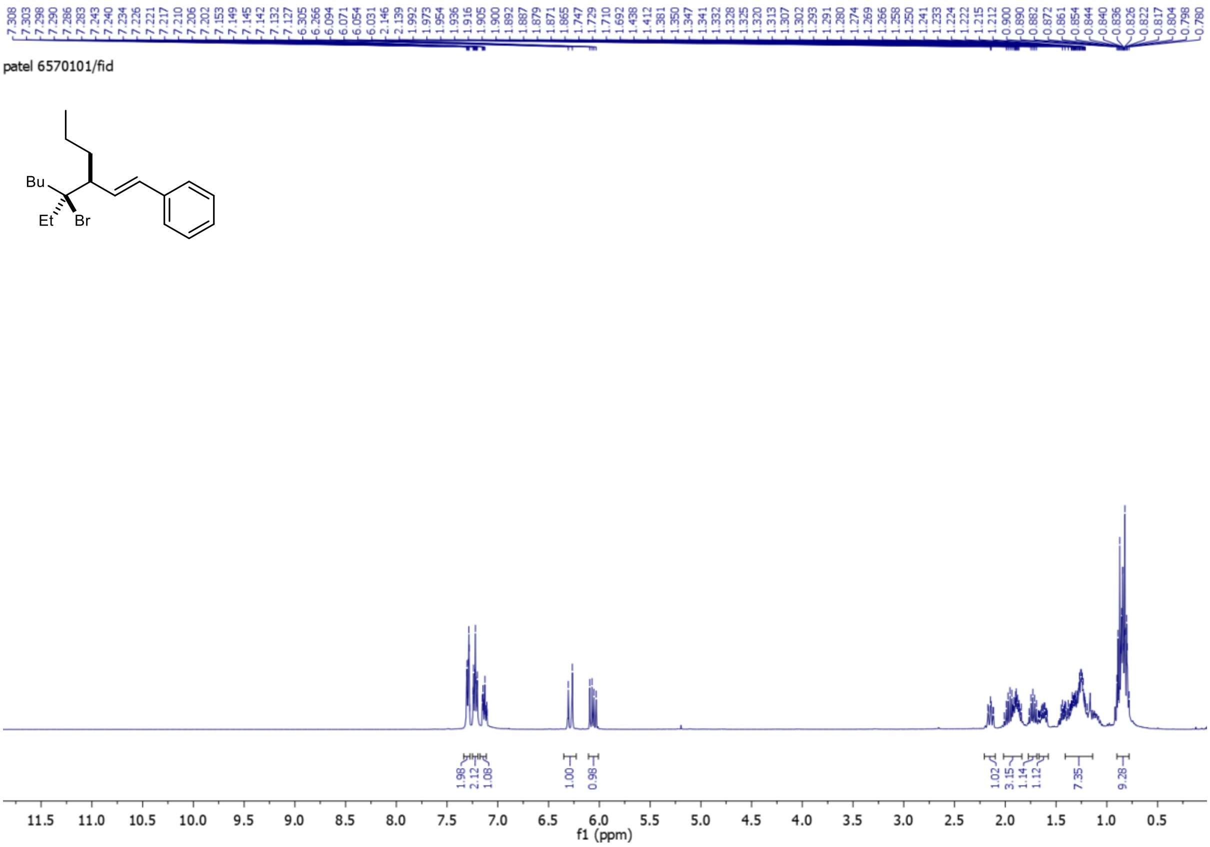
patel 6570101/fid
Bu
Et
Br
7.308
7.303
7.298
7.290
7.286
7.283
7.243
7.240
7.234
7.226
7.221
7.217
7.210
7.206
7.202
7.153
7.149
7.145
7.142
7.132
7.127
6.305
6.266
6.094
6.071
6.054
6.031
2.146
2.139
1.992
1.973
1.954
1.936
1.916
1.905
1.900
1.892
1.887
1.879
1.871
1.865
1.747
1.729
1.710
1.692
1.438
1.412
1.381
1.350
1.347
1.341
1.332
1.328
1.325
1.320
1.313
1.307
1.302
1.293
1.291
1.280
1.274
1.269
1.266
1.258
1.250
1.241
1.233
1.224
1.222
1.215
1.212
0.900
0.890
0.882
0.872
0.861
0.854
0.844
0.840
0.836
0.826
0.822
0.817
0.804
0.798
0.780
1.98
2.12
1.08
1.00
0.98
1.02
3.15
1.14
1.12
7.35
9.28
f1 (ppm)

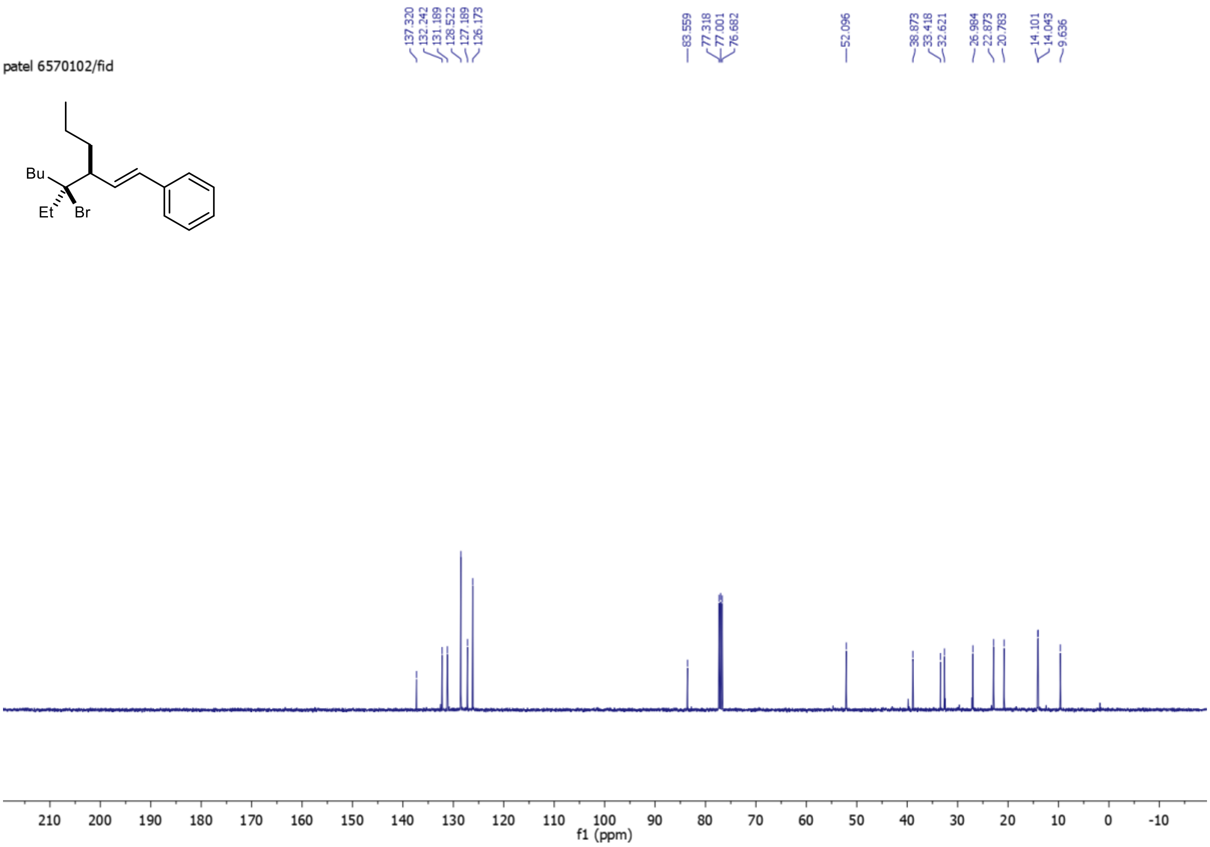

patel 6570102/fid
Bu
Et
Br
137.320
132.242
131.189
128.522
127.189
126.173
83.559
77.318
77.001
76.682
52.096
38.873
33.418
32.621
26.984
22.873
20.783
14.101
14.043
9.636
210 200 190 180 170 160 150 140 130 120 110 100 90 80 70 60 50 40 30 20 10 0 -10
f1 (ppm)

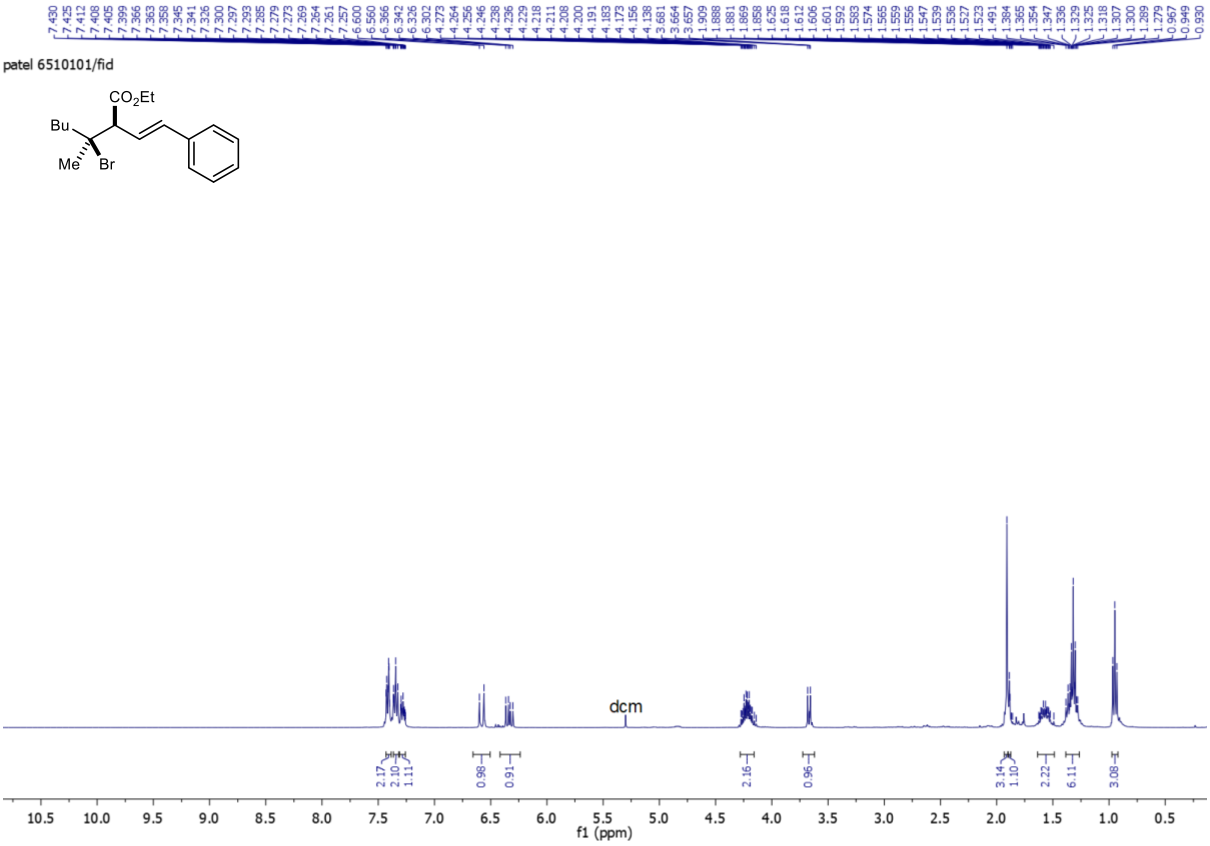

patel 6510101/fid
CO_2Et
Bu
Me
Br
7.430 7.425 7.412 7.408 7.405 7.399 7.366 7.363 7.358 7.345 7.341 7.326 7.300 7.297 7.293 7.285 7.279 7.273 7.269 7.264 7.261 7.257 6.600 6.560 6.366 6.342 6.326 6.302 4.273 4.264 4.256 4.246 4.238 4.236 4.229 4.218 4.211 4.208 4.200 4.191 4.183 4.173 4.156 4.138 3.681 3.664 3.657 1.909 1.888 1.881 1.869 1.858 1.625 1.618 1.612 1.606 1.601 1.592 1.583 1.574 1.565 1.559 1.556 1.547 1.539 1.536 1.527 1.523 1.491 1.384 1.365 1.354 1.347 1.336 1.329 1.325 1.318 1.307 1.300 1.289 1.279 0.967 0.949 0.930
dcm
2.17 2.10 1.11 0.98 0.91 2.16 0.96 3.14 1.10 2.22 6.11 3.08
10.5 10.0 9.5 9.0 8.5 8.0 7.5 7.0 6.5 6.0 5.5 5.0 4.5 4.0 3.5 3.0 2.5 2.0 1.5 1.0 0.5
f1 (ppm)

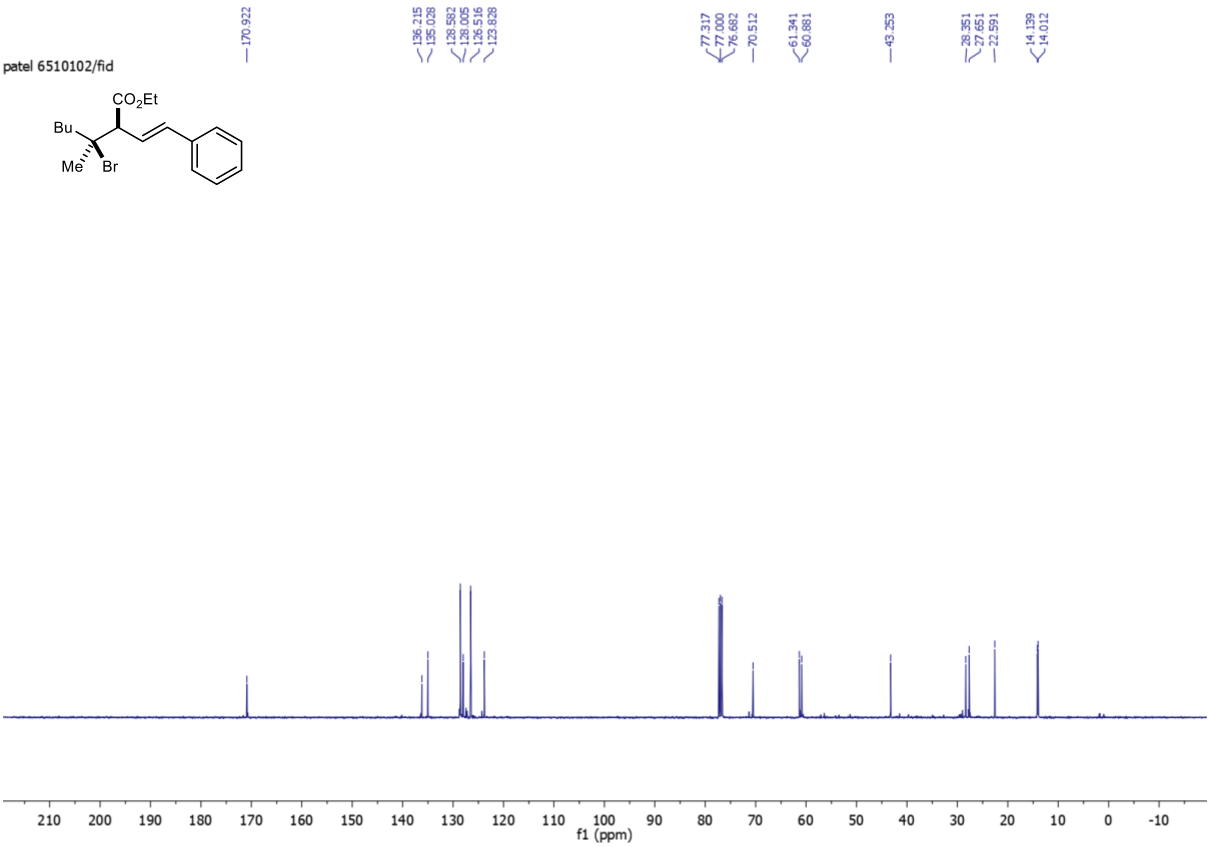
170.922
136.215
135.028
128.582
128.005
126.516
123.828
77.317
77.000
76.682
70.512
61.341
60.881
43.253
28.351
27.651
22.591
14.139
14.012
patel 6510102/fid
CO_2Et
Bu
Me
Br
210 200 190 180 170 160 150 140 130 120 110 100 90 80 70 60 50 40 30 20 10 0 -10
f1 (ppm)

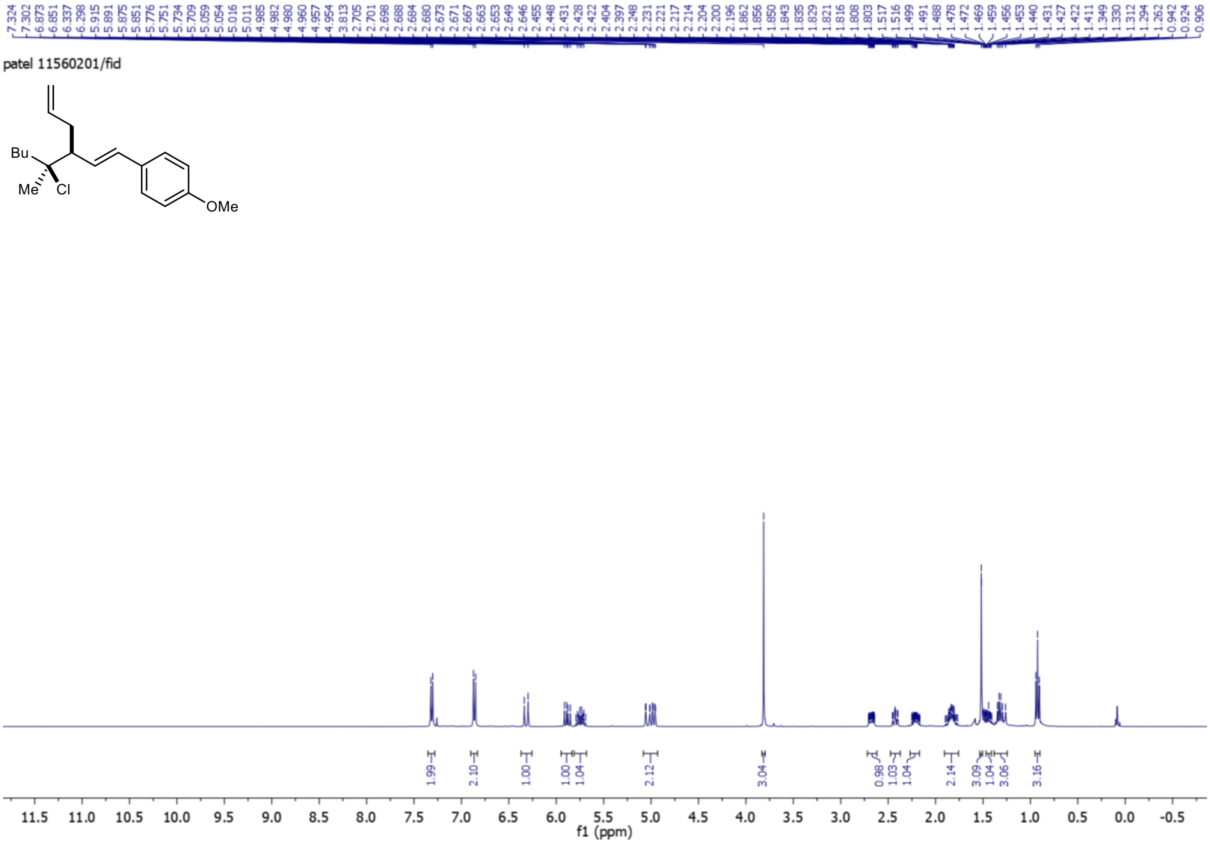
7.324 7.302 6.873 6.851 6.337 6.298 5.915 5.891 5.875 5.851 5.776 5.751 5.734 5.709 5.059 5.054 5.016 5.011 4.985 4.982 4.980 4.960 4.957 4.954 3.813 2.705 2.701 2.698 2.688 2.684 2.680 2.673 2.671 2.667 2.663 2.653 2.649 2.646 2.455 2.448 2.431 2.428 2.422 2.404 2.397 2.248 2.231 2.221 2.217 2.214 2.204 2.200 2.196 1.862 1.856 1.850 1.843 1.835 1.829 1.821 1.816 1.808 1.803 1.517 1.516 1.499 1.491 1.488 1.478 1.472 1.469 1.459 1.456 1.453 1.440 1.431 1.427 1.422 1.411 1.349 1.330 1.312 1.294 1.262 0.942 0.924 0.906
patel 11560201/fid
Bu
Me
Cl
OMe
1.99 2.10 1.00 1.00 1.04 2.12 3.04 0.98 1.03 1.04 2.14 3.09 1.04 3.06 3.16
11.5 11.0 10.5 10.0 9.5 9.0 8.5 8.0 7.5 7.0 6.5 6.0 5.5 5.0 4.5 4.0 3.5 3.0 2.5 2.0 1.5 1.0 0.5 0.0 -0.5
f1 (ppm)

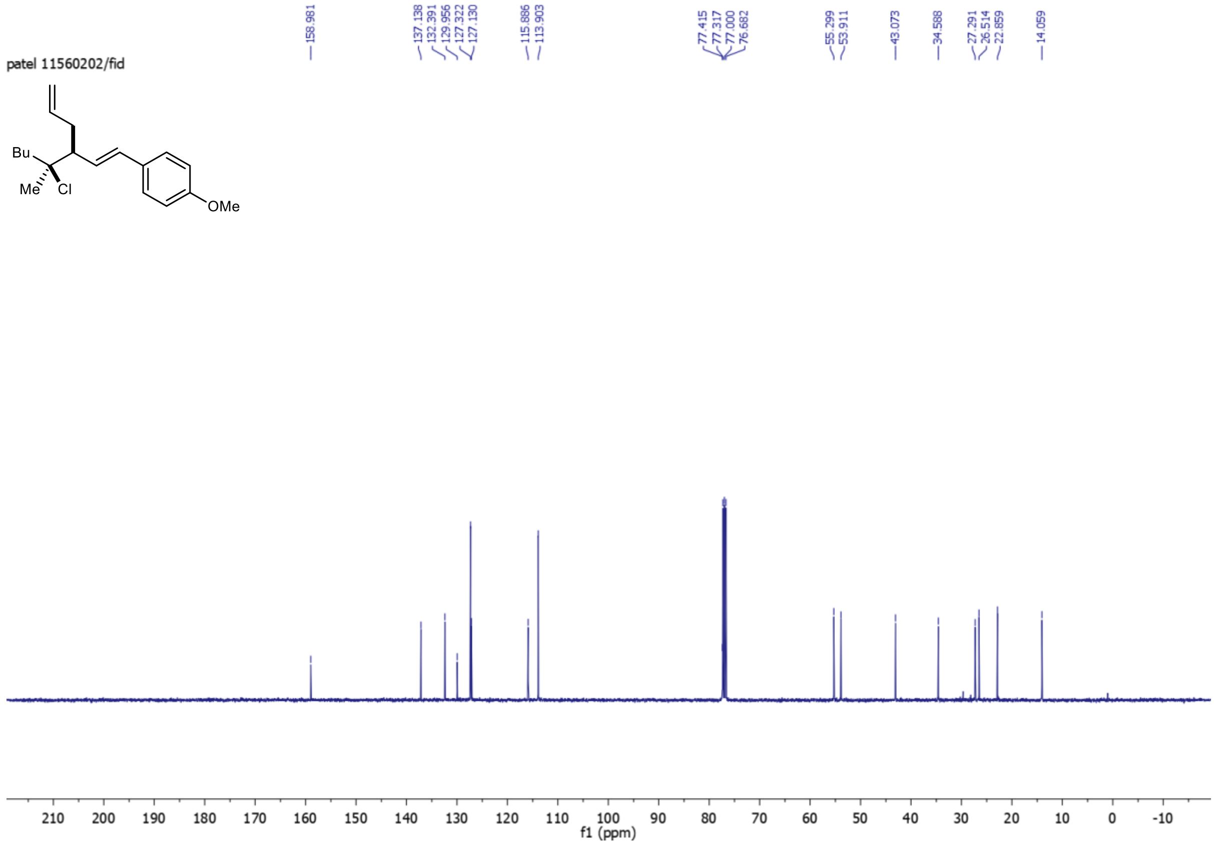
patel 11560202/fid
Bu
Me
Cl
OMe
158.981
137.138
132.391
129.956
127.322
127.130
115.886
113.903
77.415
77.317
77.000
76.682
55.299
53.911
43.073
34.588
27.291
26.514
22.859
14.059
f1 (ppm)

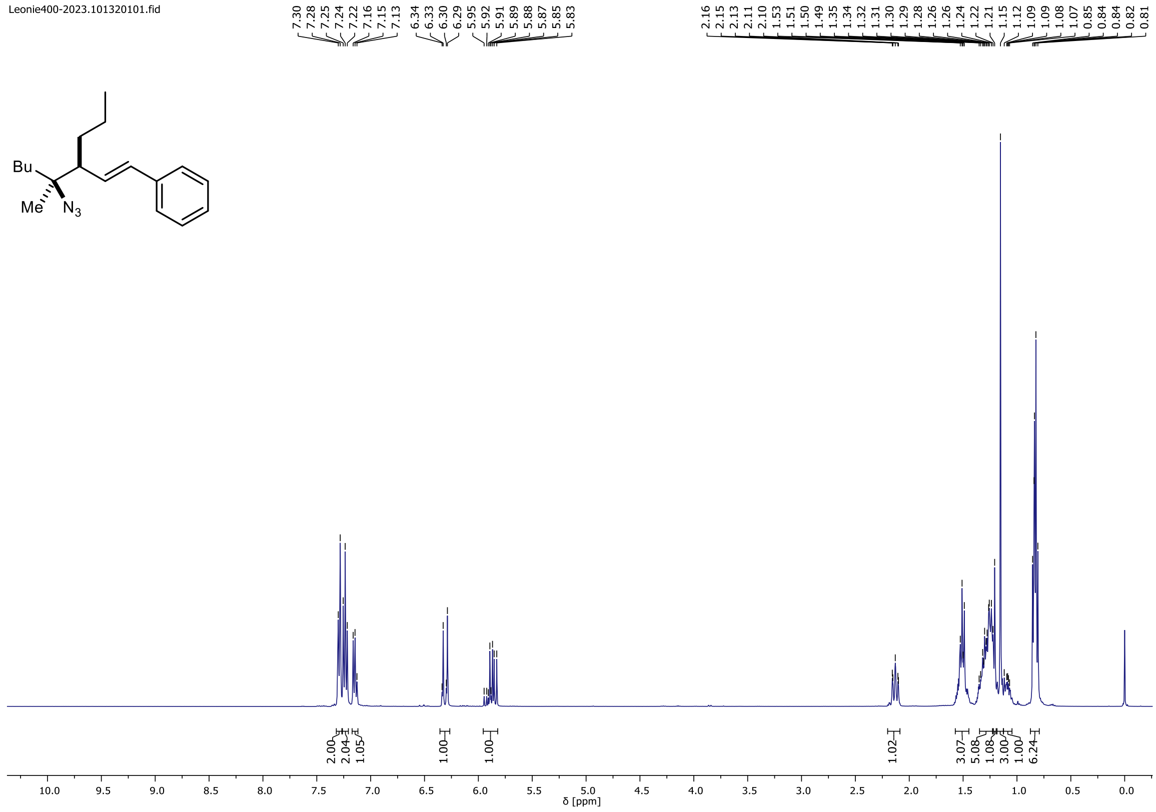
Leonie400-2023.101320101.fid
Bu
Me
N_3
7.30 7.28 7.25 7.24 7.22 7.16 7.15 7.13 6.34 6.33 6.30 6.29 5.95 5.92 5.91 5.89 5.88 5.87 5.85 5.83
2.16 2.15 2.13 2.11 2.10 1.53 1.51 1.50 1.49 1.35 1.34 1.32 1.31 1.30 1.29 1.28 1.26 1.24 1.22 1.21 1.15 1.12 1.09 1.09 1.08 1.07 0.85 0.84 0.84 0.82 0.81
2.00 2.04 1.05 1.00 1.00 1.02 3.07 5.08 1.08 3.00 1.00 6.24
δ [ppm]

Leonie400-2023.101320102.fid

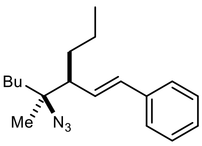

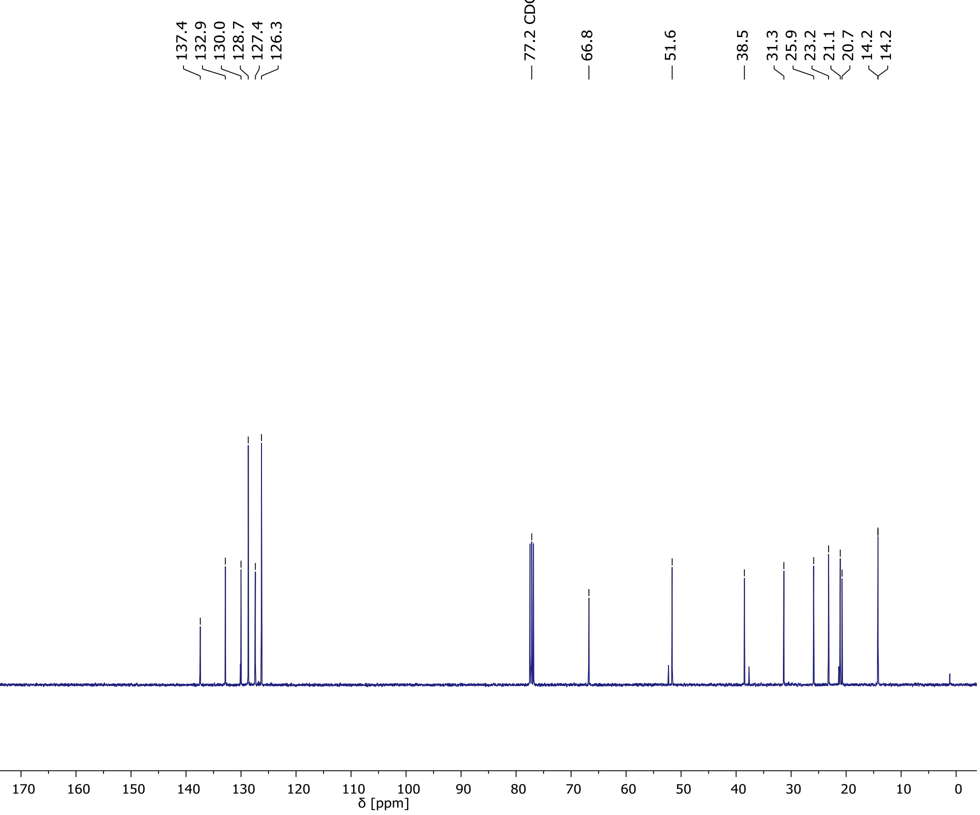

Leonie400-2023.101120101.fid

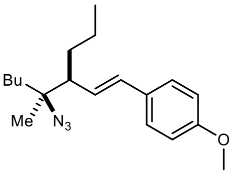

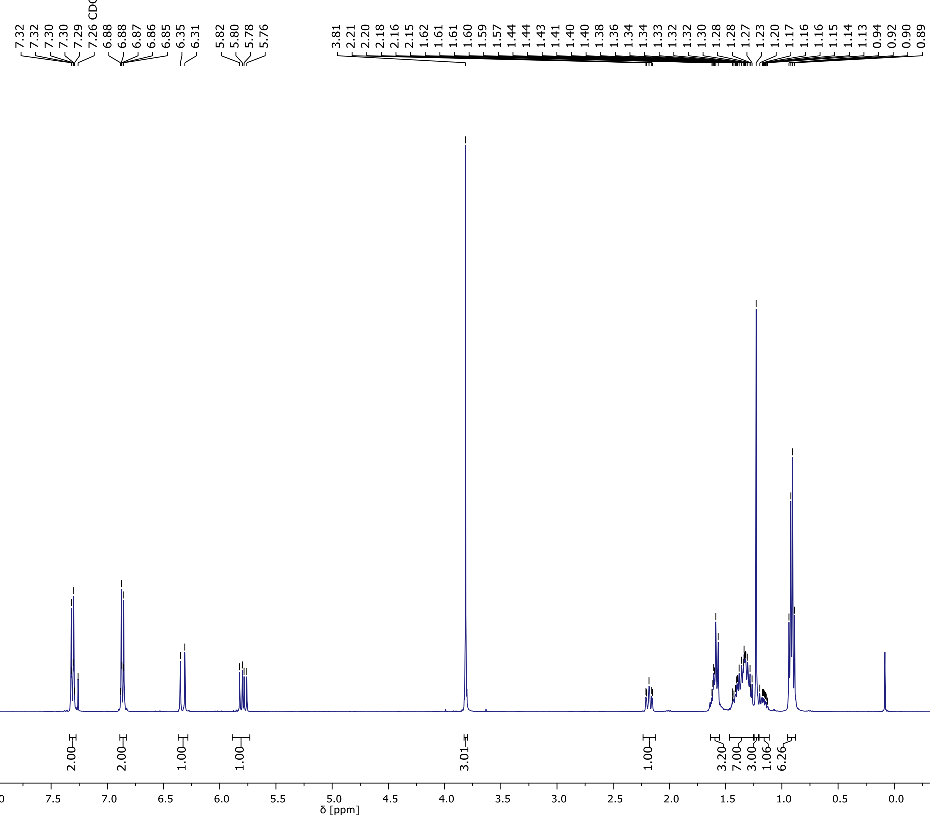

Leonie400-2023.101120102.fid

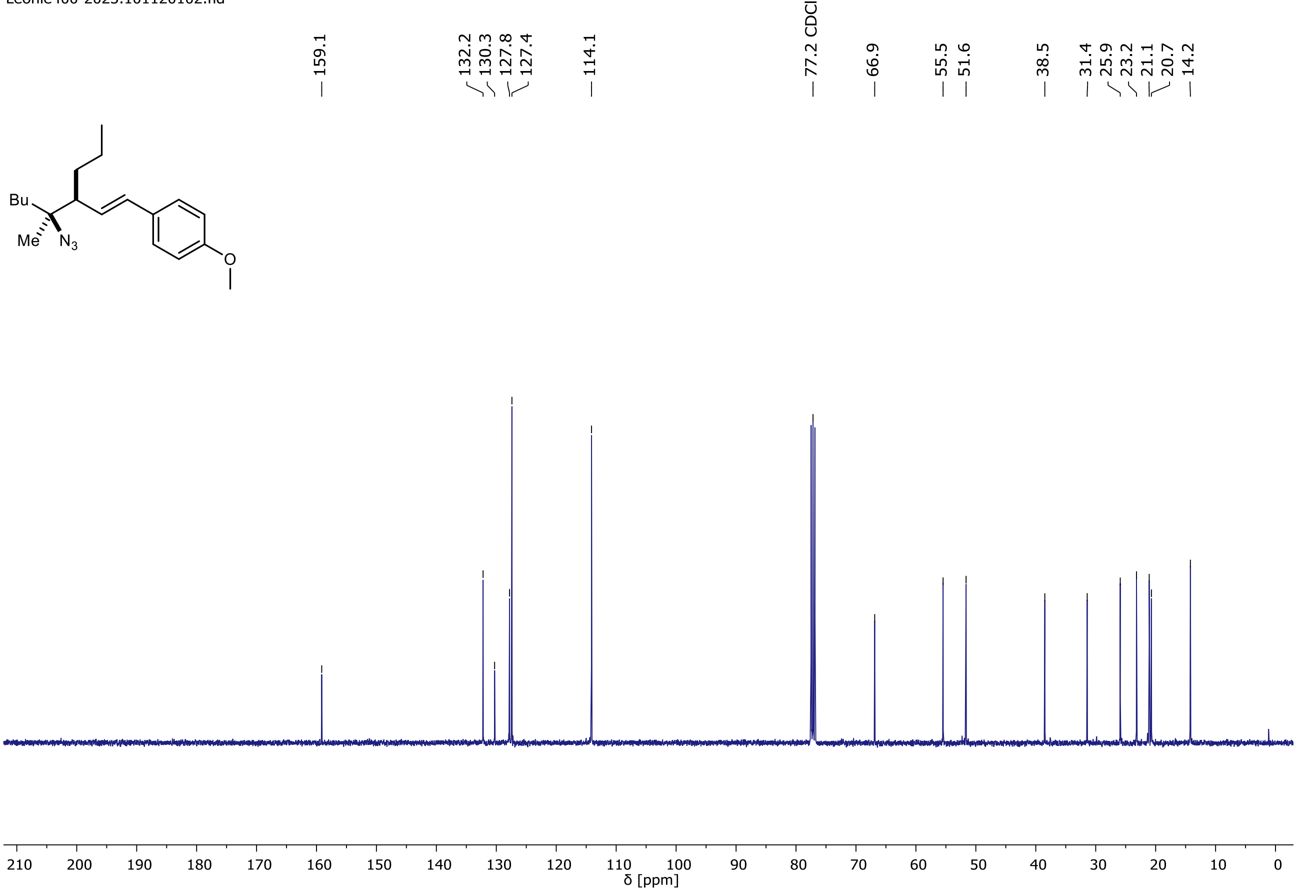

Leonie400-2023.101330201.fid

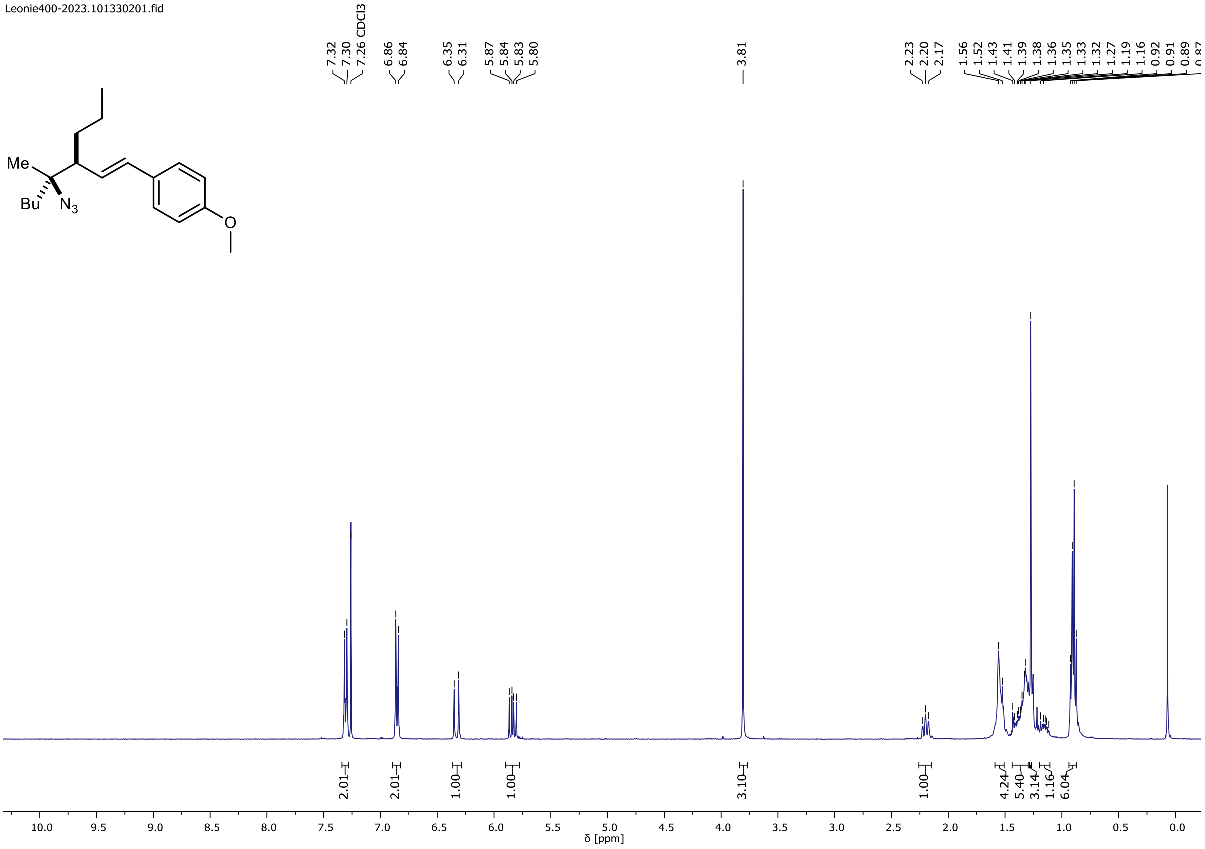

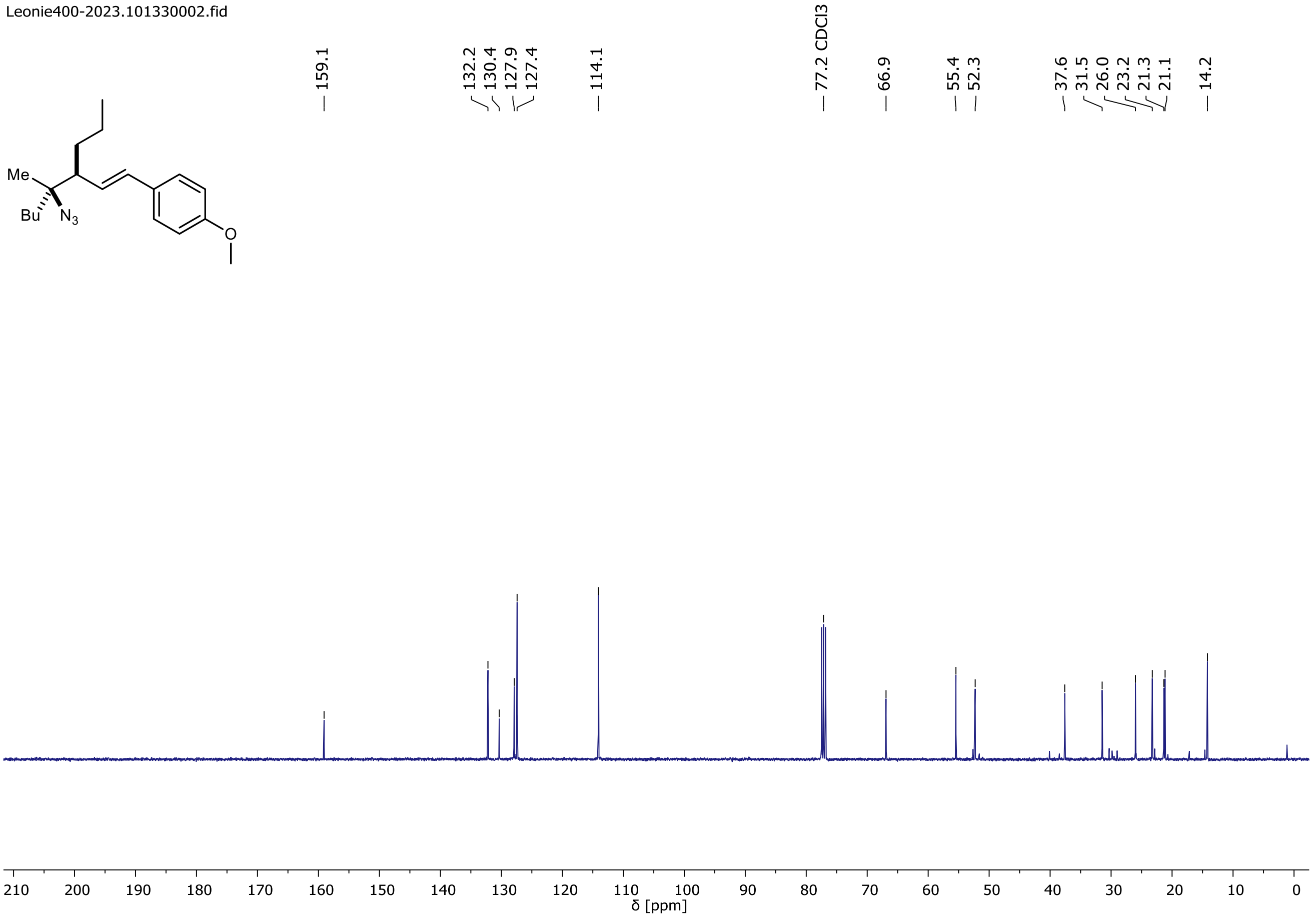
Leonie400-2023.101330002.fid
159.1
132.2
130.4
127.9
127.4
114.1
77.2 CDCl3
66.9
55.4
52.3
37.6
31.5
26.0
23.2
21.3
21.1
14.2
Me
Bu
N3
O
δ [ppm]
210 200 190 180 170 160 150 140 130 120 110 100 90 80 70 60 50 40 30 20 10 0

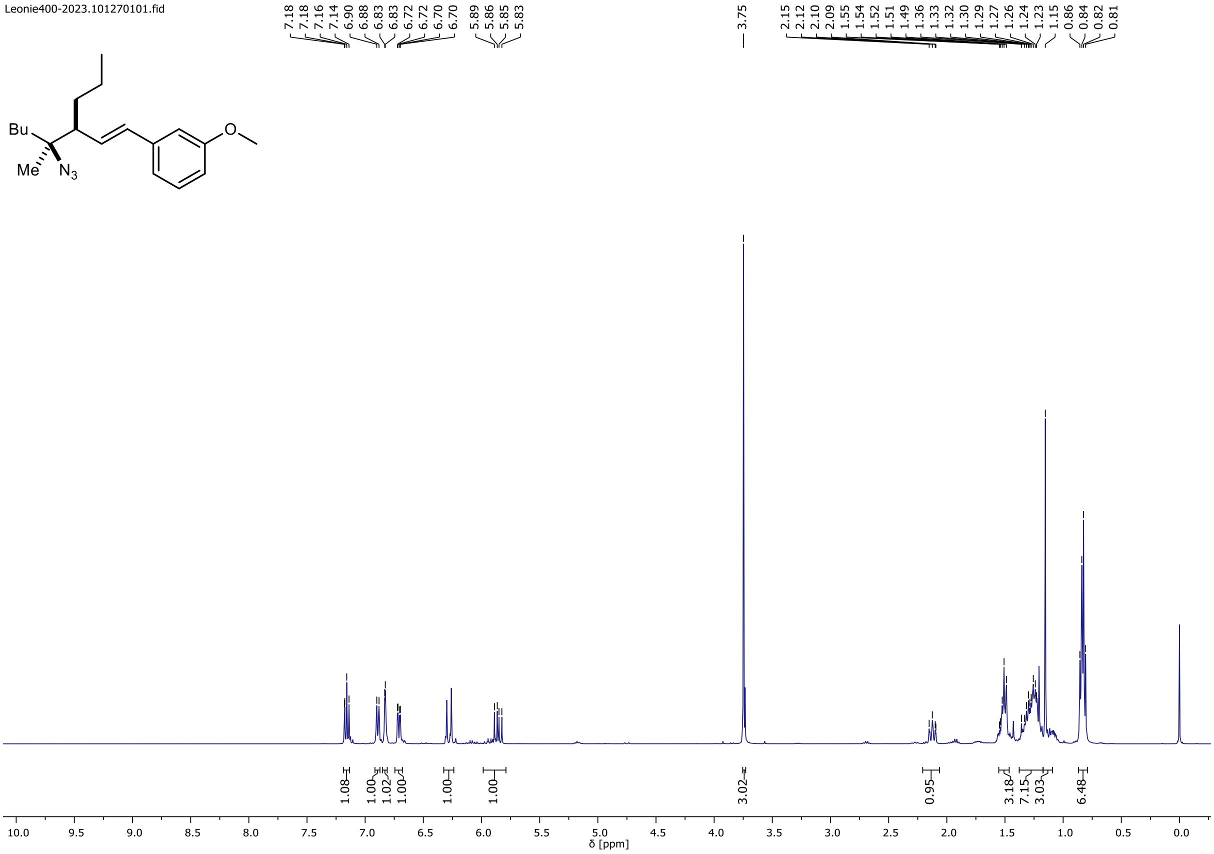
Leonie400-2023.101270101.fid
7.18 7.18 7.16 7.14 6.90 6.88 6.83 6.83 6.72 6.72 6.70 6.70
5.89 5.86 5.85 5.83
3.75
2.15 2.12 2.10 2.09 1.55 1.54 1.52 1.51 1.49 1.36 1.33 1.32 1.30 1.29 1.27 1.26 1.24 1.23 1.15 0.86 0.84 0.82 0.81
Bu
Me
N3
O
1.08 1.00 1.02 1.00 1.00 1.00 3.02 0.95 3.18 7.15 3.03 6.48
δ [ppm]
10.0 9.5 9.0 8.5 8.0 7.5 7.0 6.5 6.0 5.5 5.0 4.5 4.0 3.5 3.0 2.5 2.0 1.5 1.0 0.5 0.0

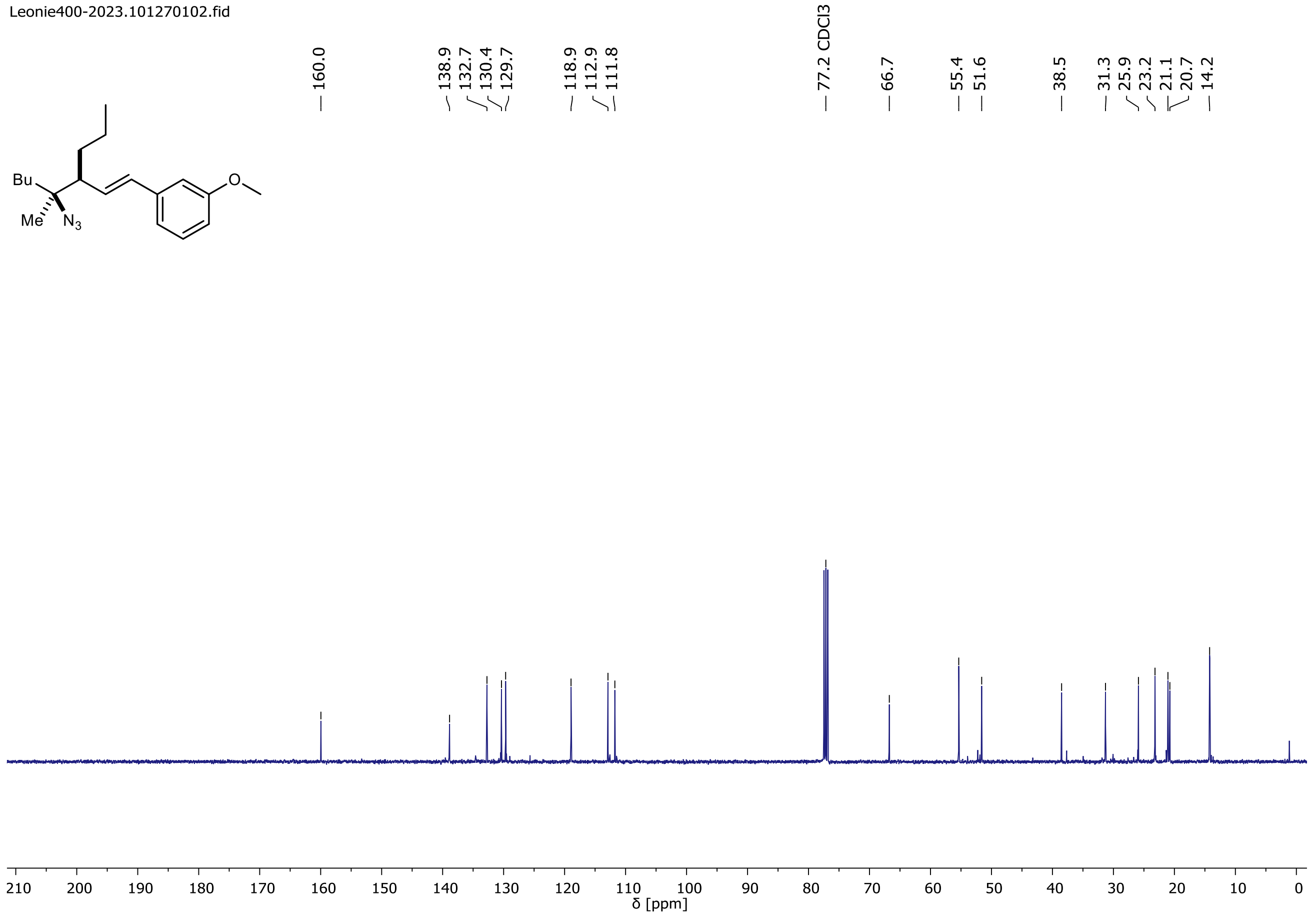
Leonie400-2023.101270102.fid
160.0
138.9
132.7
130.4
129.7
118.9
112.9
111.8
77.2 CDCl3
66.7
55.4
51.6
38.5
31.3
25.9
23.2
21.1
20.7
14.2
Bu
Me
N_3
O
δ [ppm]

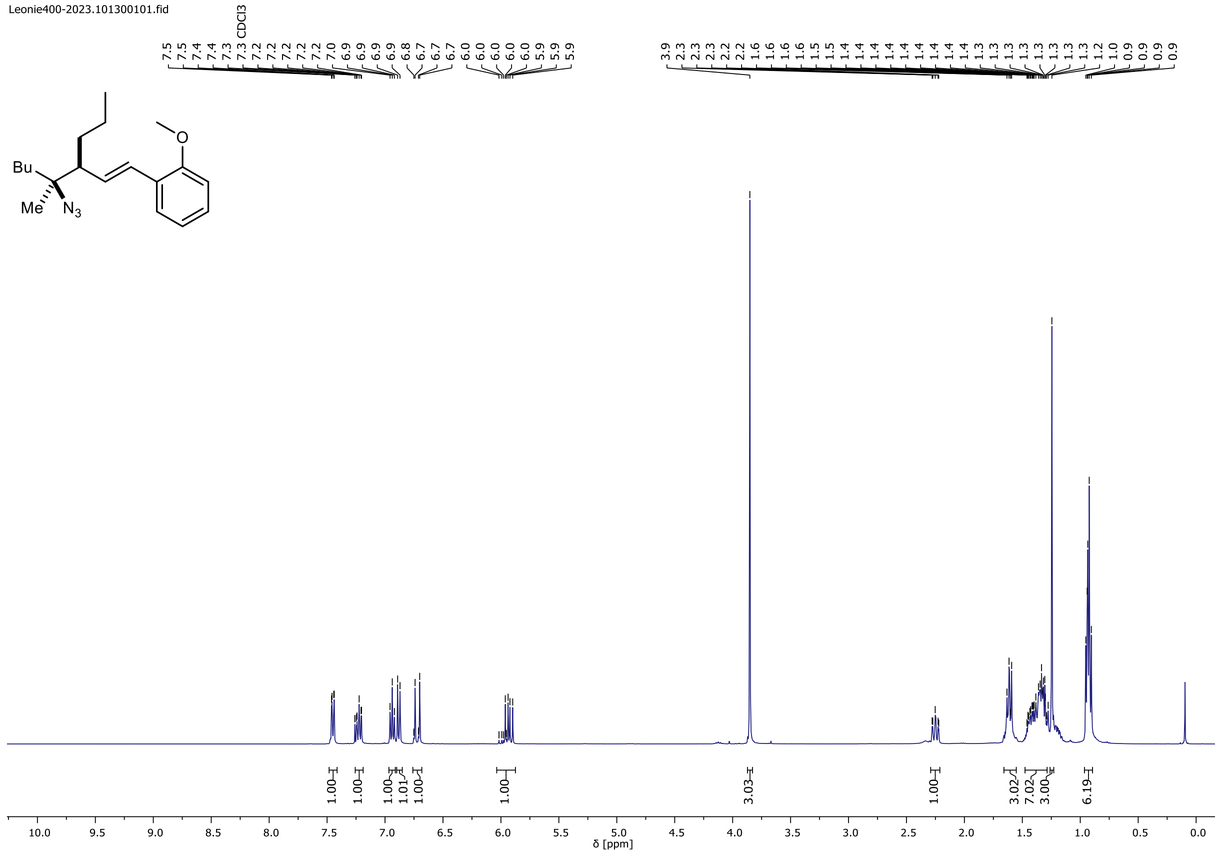
Leonie400-2023.101300101.fid
CDCl3
Bu
Me
N_3
O
1.00
1.00
1.00
1.01
1.00
1.00
3.03
1.00
3.02
7.02
3.00
6.19
δ [ppm]

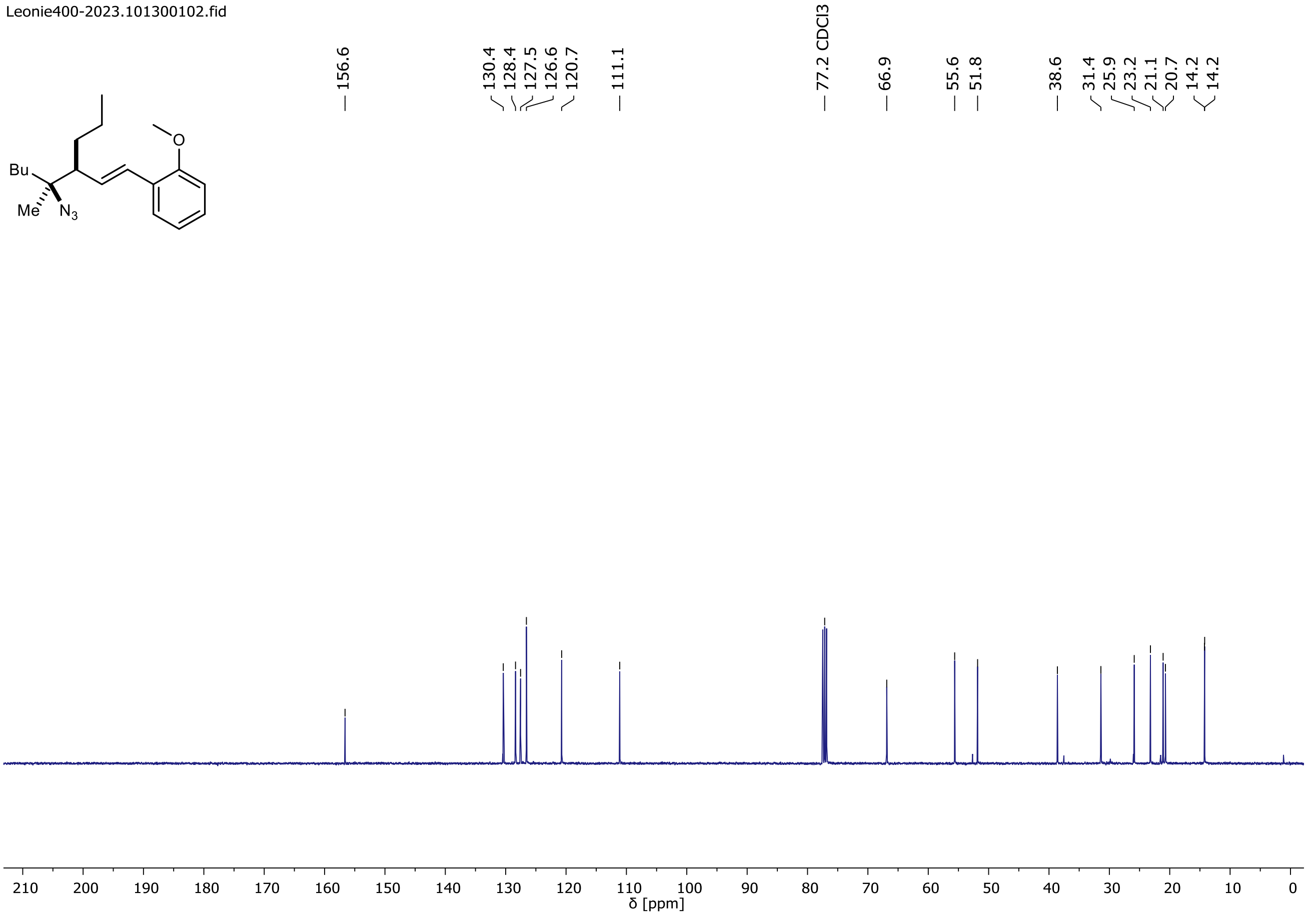
Leonie400-2023.101300102.fid
Bu
Me
N3
O
156.6
130.4
128.4
127.5
126.6
120.7
111.1
77.2 CDCl3
66.9
55.6
51.8
38.6
31.4
25.9
23.2
21.1
20.7
14.2
14.2
210 200 190 180 170 160 150 140 130 120 110 100 90 80 70 60 50 40 30 20 10 0
δ [ppm]

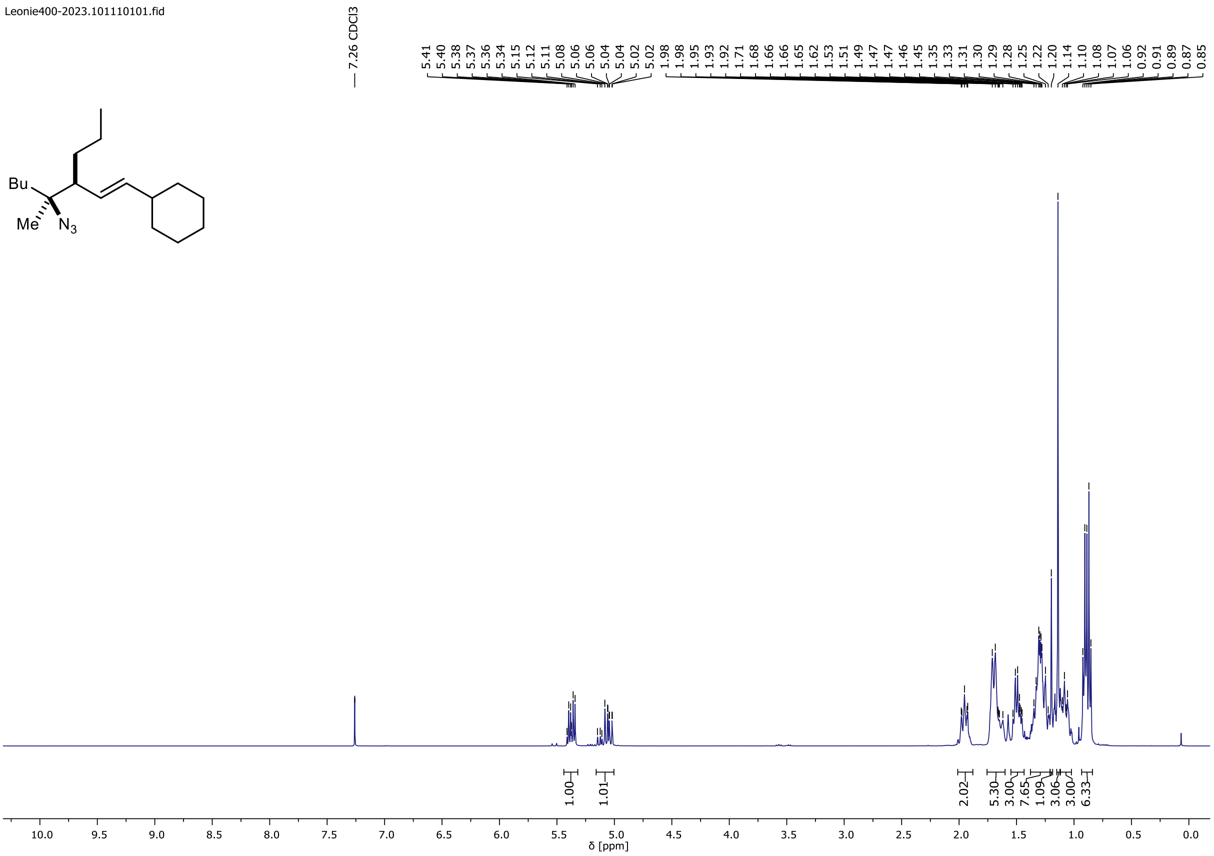
Leonie400-2023.101110101.fid
Bu
Me
N3
7.26 CDCl3
5.41 5.40 5.38 5.37 5.36 5.34 5.15 5.12 5.11 5.08 5.06 5.06 5.04 5.04 5.02 5.02 1.98 1.95 1.93 1.92 1.71 1.68 1.66 1.66 1.65 1.62 1.53 1.51 1.49 1.47 1.47 1.46 1.45 1.35 1.33 1.31 1.30 1.29 1.28 1.25 1.22 1.20 1.14 1.10 1.08 1.07 1.06 0.92 0.91 0.89 0.87 0.85
1.00
1.01
2.02
5.30
3.00
7.65
1.09
3.06
3.00
6.33
10.0 9.5 9.0 8.5 8.0 7.5 7.0 6.5 6.0 5.5 5.0 4.5 4.0 3.5 3.0 2.5 2.0 1.5 1.0 0.5 0.0
δ [ppm]

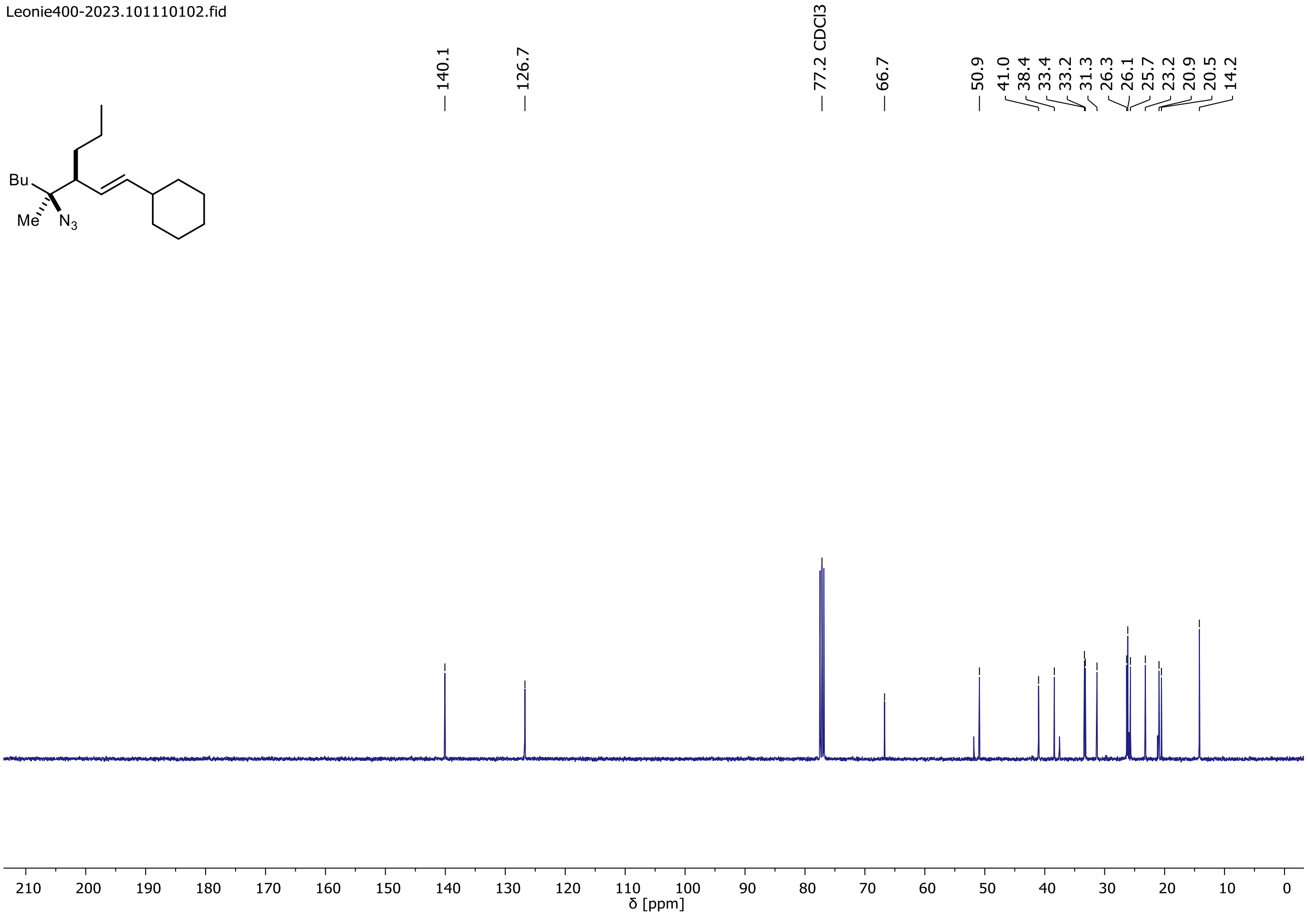
Leonie400-2023.101110102.fid
140.1
126.7
77.2 CDCl3
66.7
50.9
41.0
38.4
33.4
33.2
31.3
26.3
26.1
25.7
23.2
20.9
20.5
14.2
Bu
Me
N3
210 200 190 180 170 160 150 140 130 120 110 100 90 80 70 60 50 40 30 20 10 0
δ [ppm]

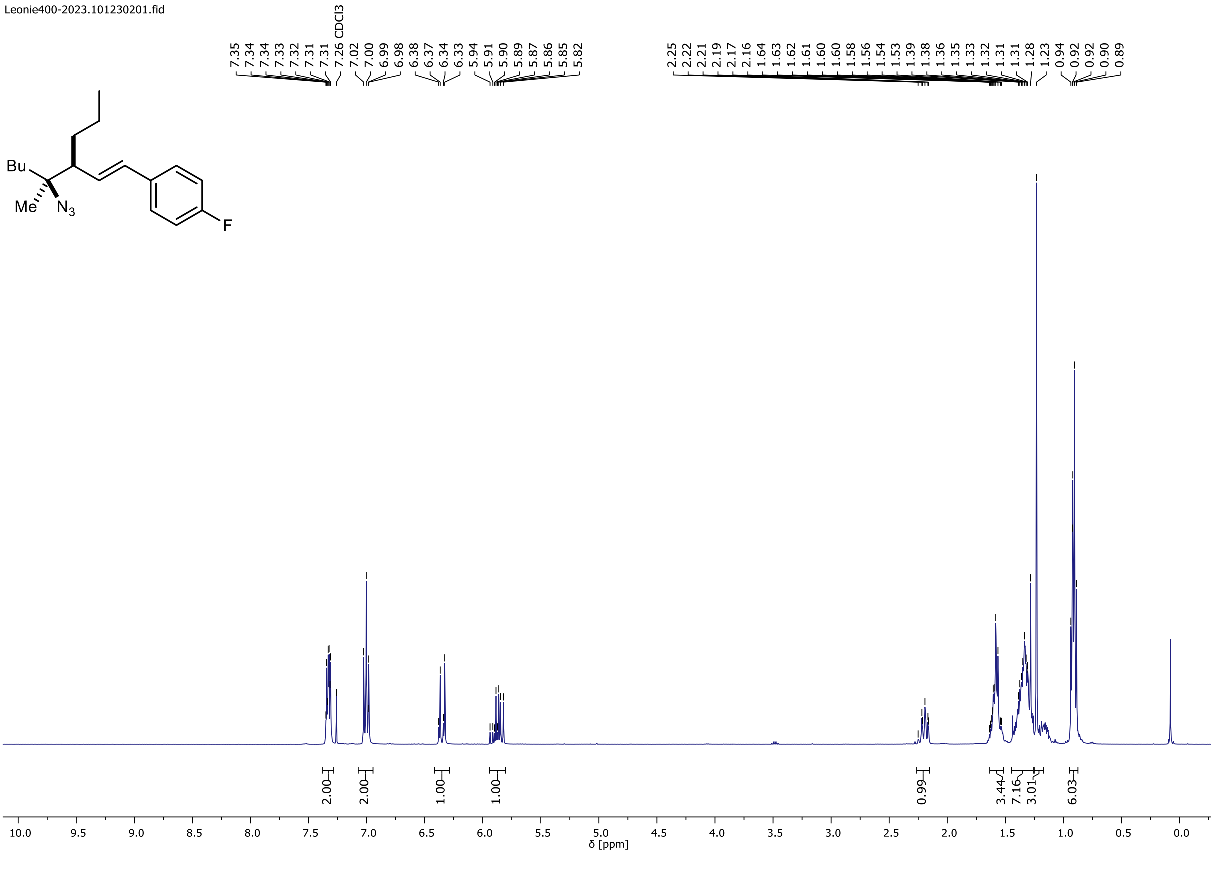
Leonie400-2023.101230201.fid
7.35 7.34 7.34 7.33 7.32 7.31 7.31 7.26 CDCl3 7.02 7.00 6.99 6.98 6.38 6.37 6.34 6.33 5.94 5.91 5.90 5.89 5.87 5.86 5.85 5.82
2.25 2.22 2.21 2.19 2.17 2.16 1.64 1.63 1.62 1.61 1.60 1.60 1.58 1.56 1.54 1.53 1.39 1.38 1.36 1.35 1.33 1.32 1.31 1.31 1.28 1.23 0.94 0.92 0.92 0.90 0.89
Bu
Me
N3
F
2.00
2.00
1.00
1.00
0.99
3.44
7.16
3.01
6.03
10.0 9.5 9.0 8.5 8.0 7.5 7.0 6.5 6.0 5.5 5.0 4.5 4.0 3.5 3.0 2.5 2.0 1.5 1.0 0.5 0.0
δ [ppm]

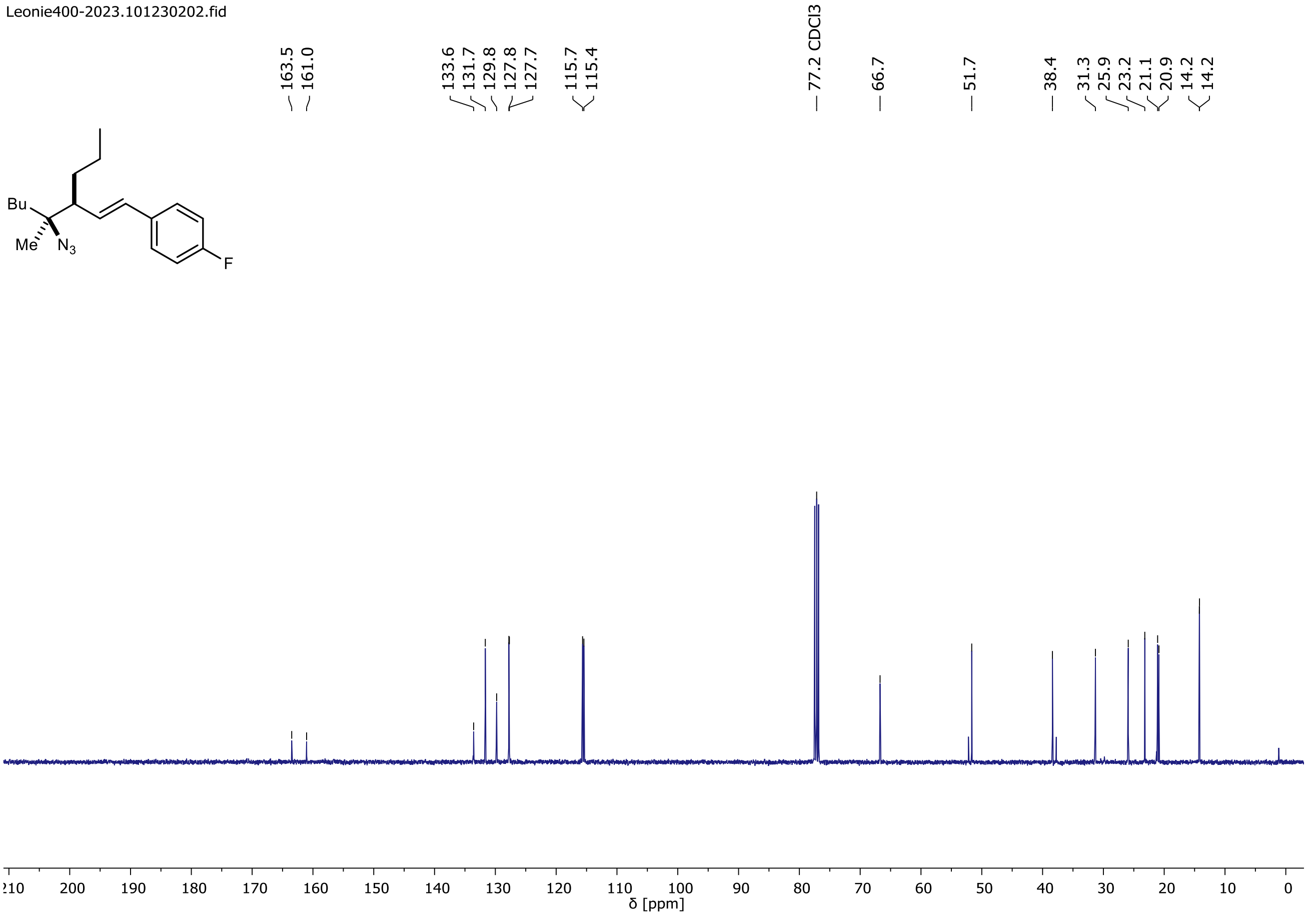
Leonie400-2023.101230202.fid
163.5
161.0
133.6
131.7
129.8
127.8
127.7
115.7
115.4
77.2 CDCl3
66.7
51.7
38.4
31.3
25.9
23.2
21.1
20.9
14.2
14.2
Bu
Me
N3
F
δ [ppm]

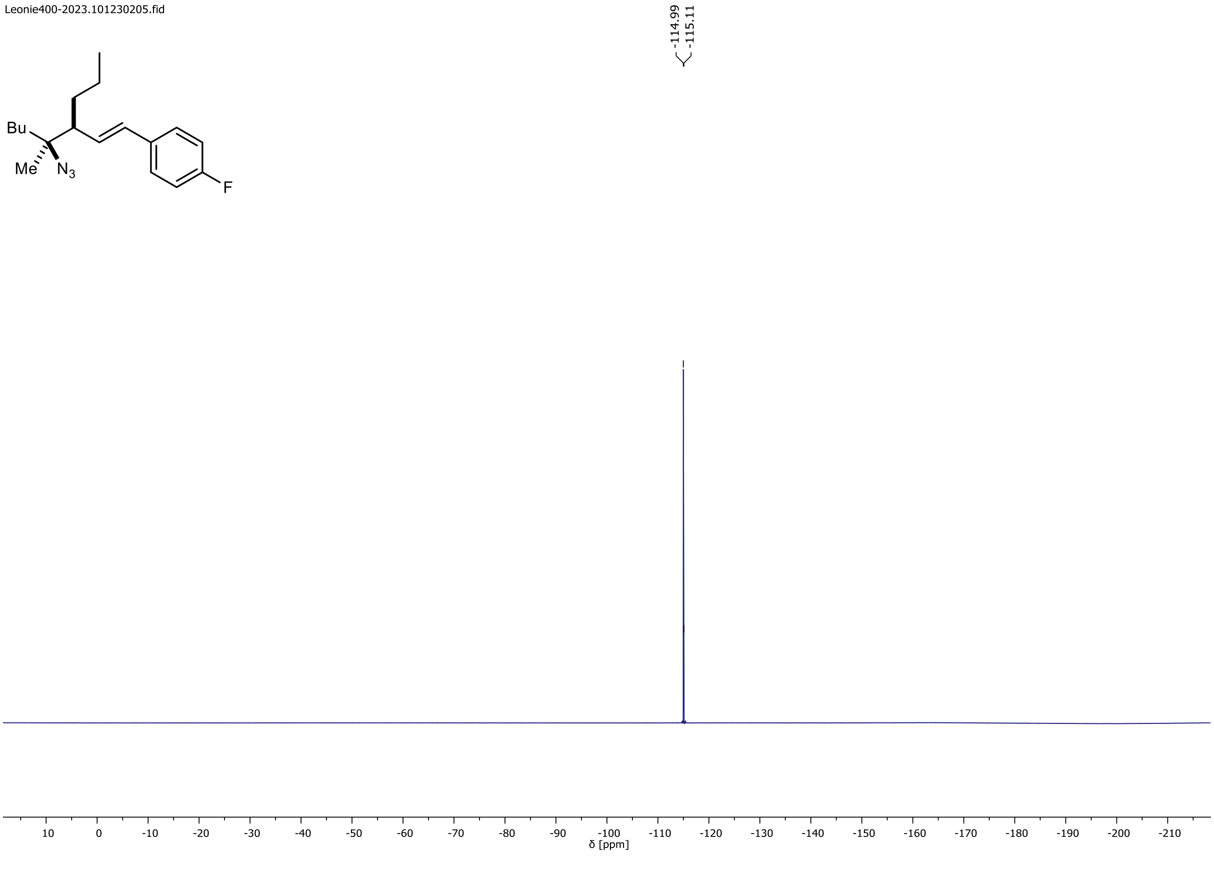
Leonie400-2023.101230205.fid
-114.99
-115.11
Bu
Me
N3
F
δ [ppm]

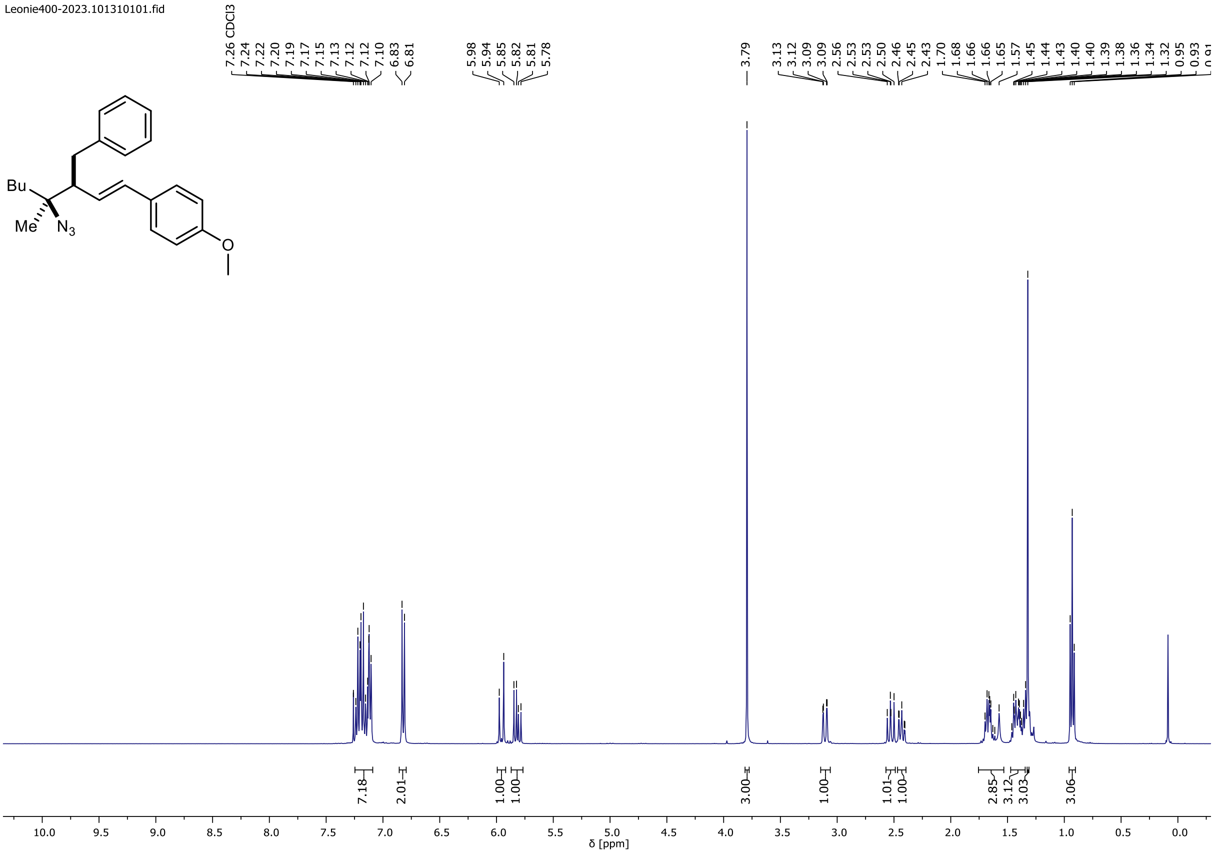
Leonie400-2023.101310101.fid
7.26 CDCl3
7.24
7.22
7.20
7.19
7.17
7.15
7.13
7.12
7.12
7.10
6.83
6.81
5.98
5.94
5.85
5.82
5.81
5.78
3.79
3.13
3.12
3.09
3.09
2.56
2.53
2.53
2.50
2.46
2.45
2.43
1.70
1.68
1.66
1.66
1.65
1.57
1.45
1.45
1.44
1.43
1.40
1.40
1.39
1.38
1.36
1.34
1.32
0.95
0.93
Bu
Me
N3
O
7.18
2.01
1.00
1.00
3.00
1.00
1.01
1.00
2.85
3.12
3.03
3.06
10.0
9.5
9.0
8.5
8.0
7.5
7.0
6.5
6.0
5.5
5.0
4.5
4.0
3.5
3.0
2.5
2.0
1.5
1.0
0.5
0.0
δ [ppm]

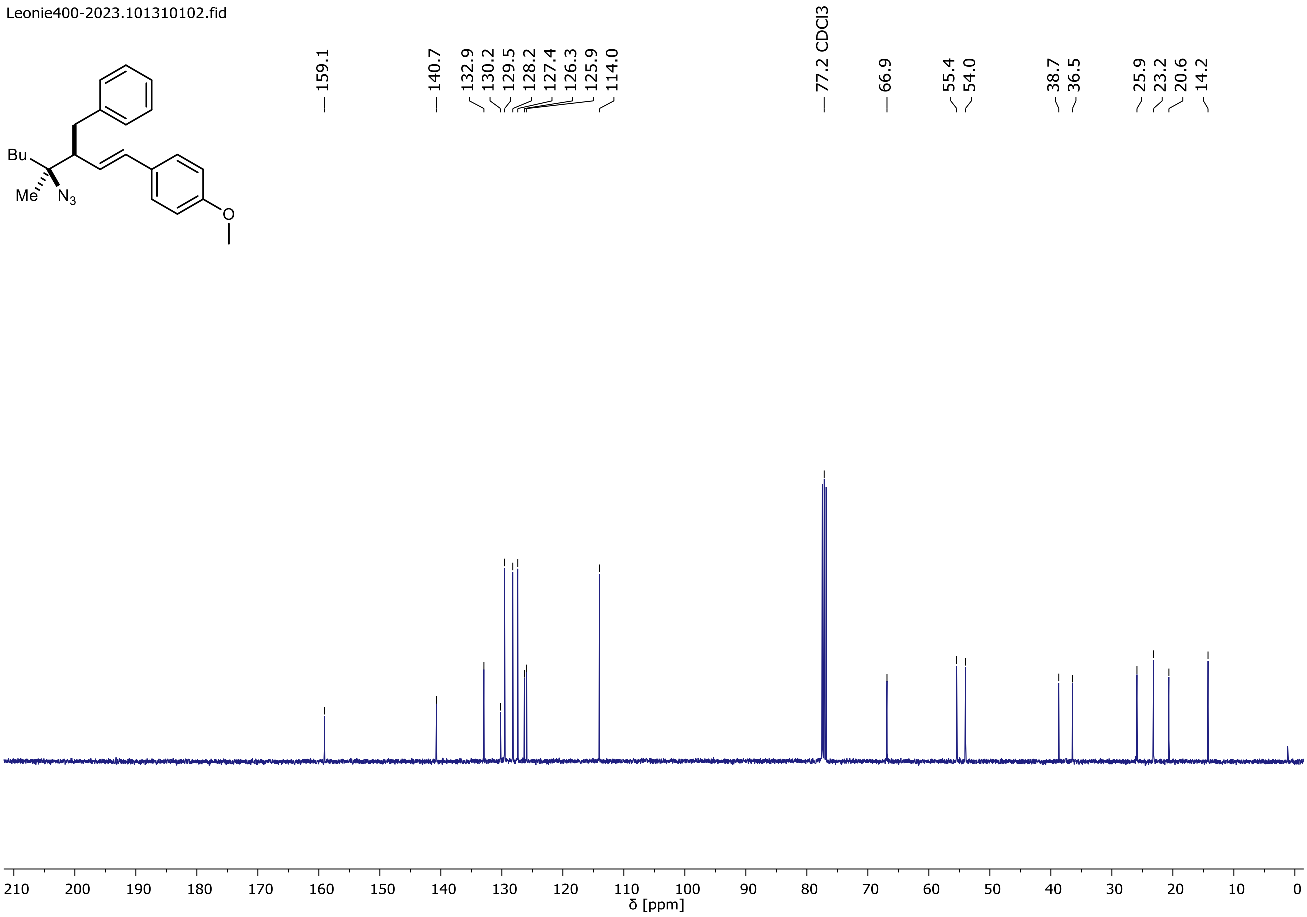
Leonie400-2023.101310102.fid
159.1
140.7
132.9
130.2
129.5
128.2
127.4
126.3
125.9
114.0
77.2 CDCl3
66.9
55.4
54.0
38.7
36.5
25.9
23.2
20.6
14.2
Bu
Me
N3
O
210
200
190
180
170
160
150
140
130
120
110
100
90
80
70
60
50
40
30
20
10
0
δ [ppm]

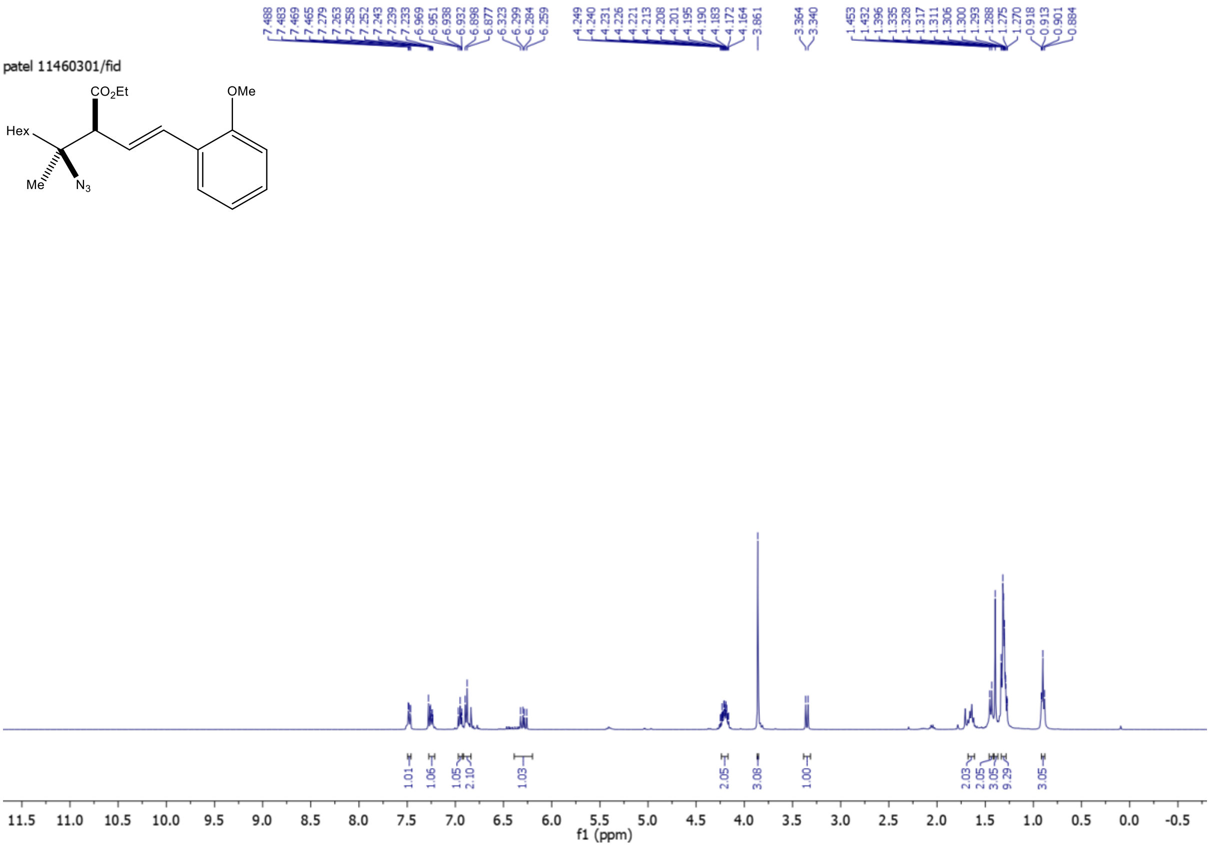
7.488 7.483 7.469 7.465 7.279 7.263 7.258 7.252 7.243 7.239 7.233 6.969 6.951 6.938 6.932 6.898 6.877 6.323 6.299 6.284 6.259
4.249 4.240 4.231 4.226 4.221 4.213 4.208 4.201 4.195 4.190 4.183 4.172 4.164 3.861
3.364 3.340
1.453 1.432 1.396 1.335 1.328 1.317 1.311 1.306 1.300 1.293 1.288 1.275 1.270 0.918 0.913 0.901 0.884
patel 11460301/fid
CO2Et
OMe
Hex
Me
N3
1.01 1.06 1.05 2.10 1.03 2.05 3.08 1.00 2.03 2.05 3.05 9.29 3.05
f1 (ppm)

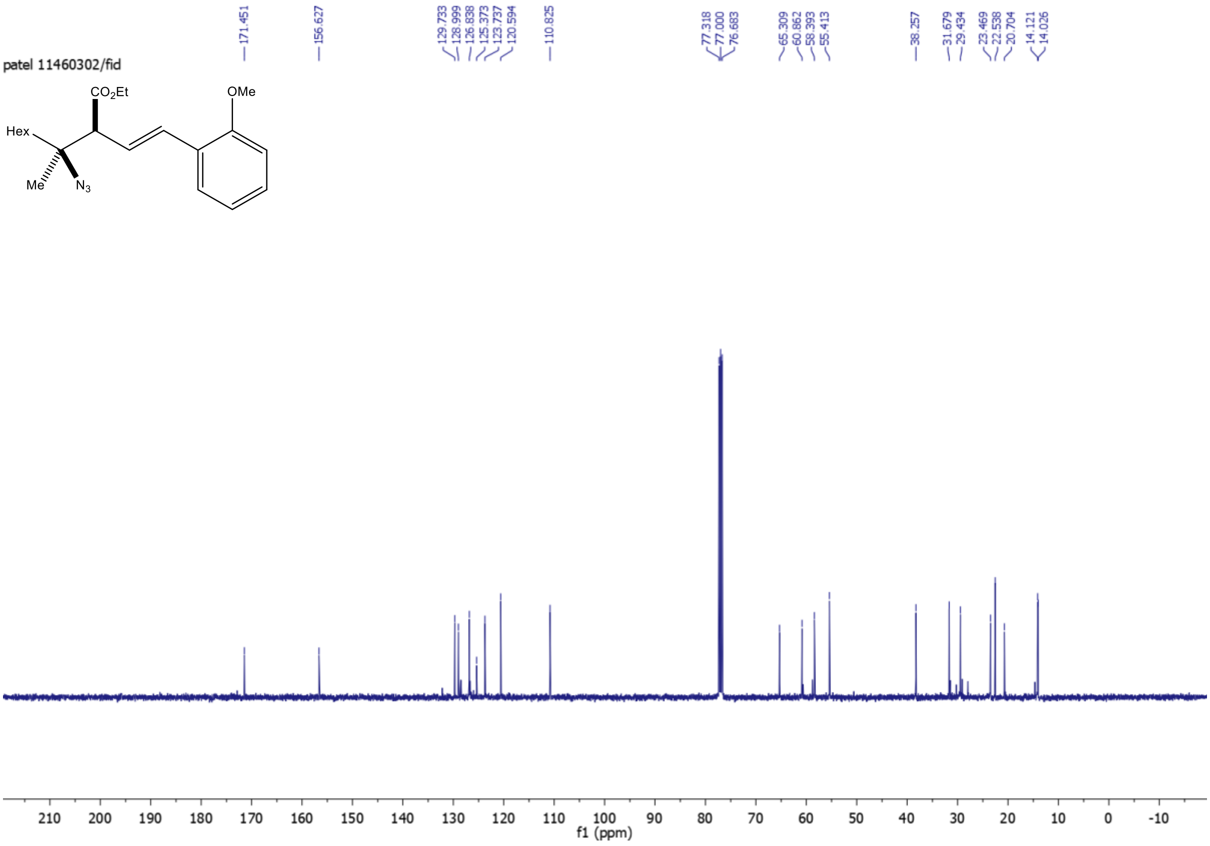
171.451
156.627
129.733 128.999 126.838 125.373 123.737 120.594
110.825
77.318 77.000 76.683
65.309 60.862 58.393 55.413
38.257
31.679 29.434
23.469 22.538 20.704
14.121 14.026
patel 11460302/fid
CO2Et
OMe
Hex
Me
N3
f1 (ppm)

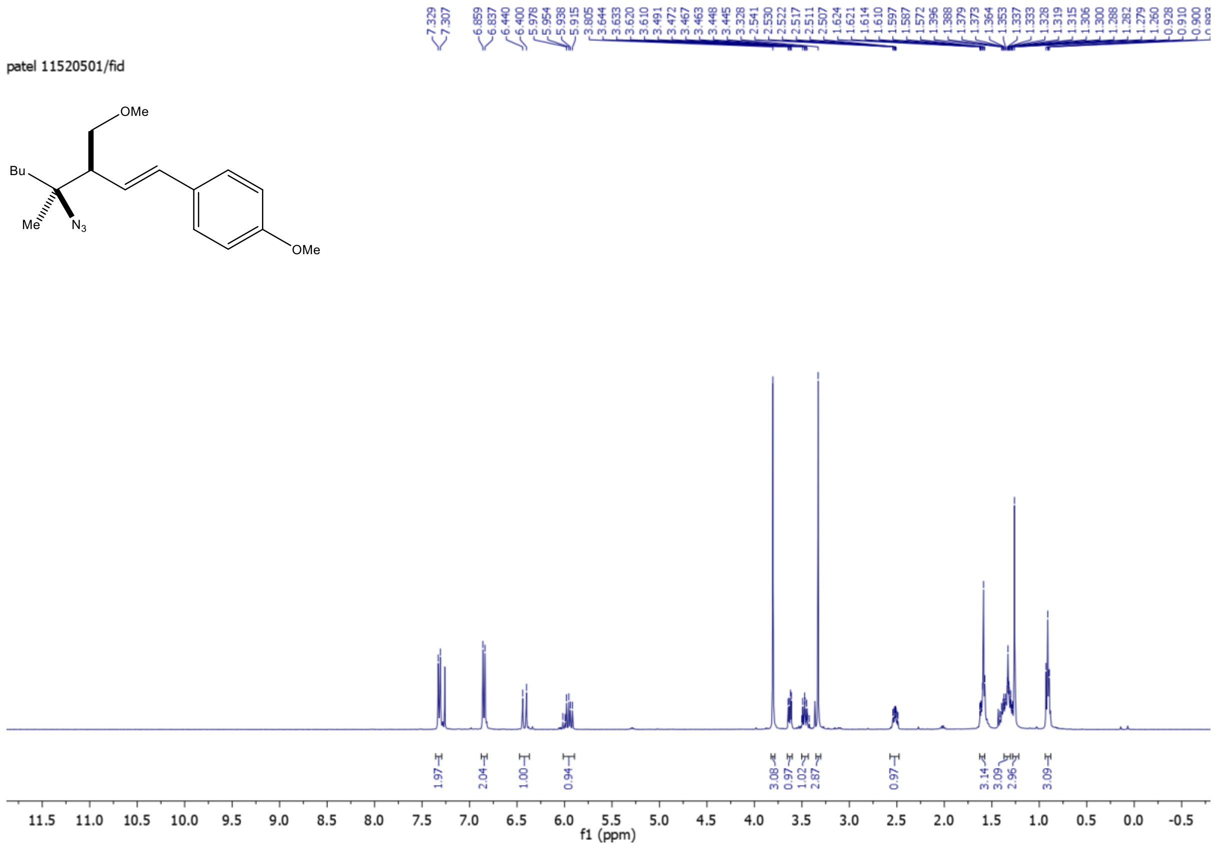
patel 11520501/fid
OMe
Bu
Me
N3
OMe
f1 (ppm)

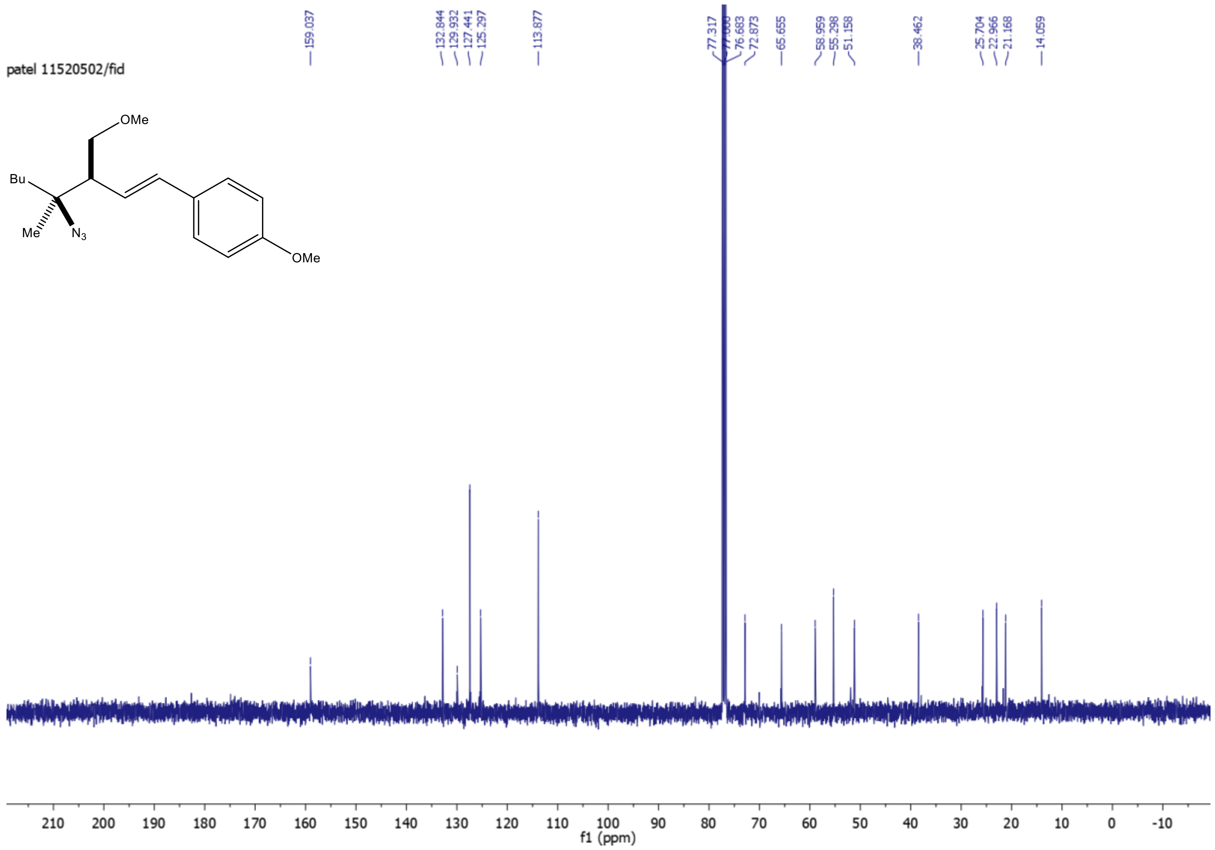
patel 11520502/fid
OMe
Bu
Me
N3
OMe
f1 (ppm)

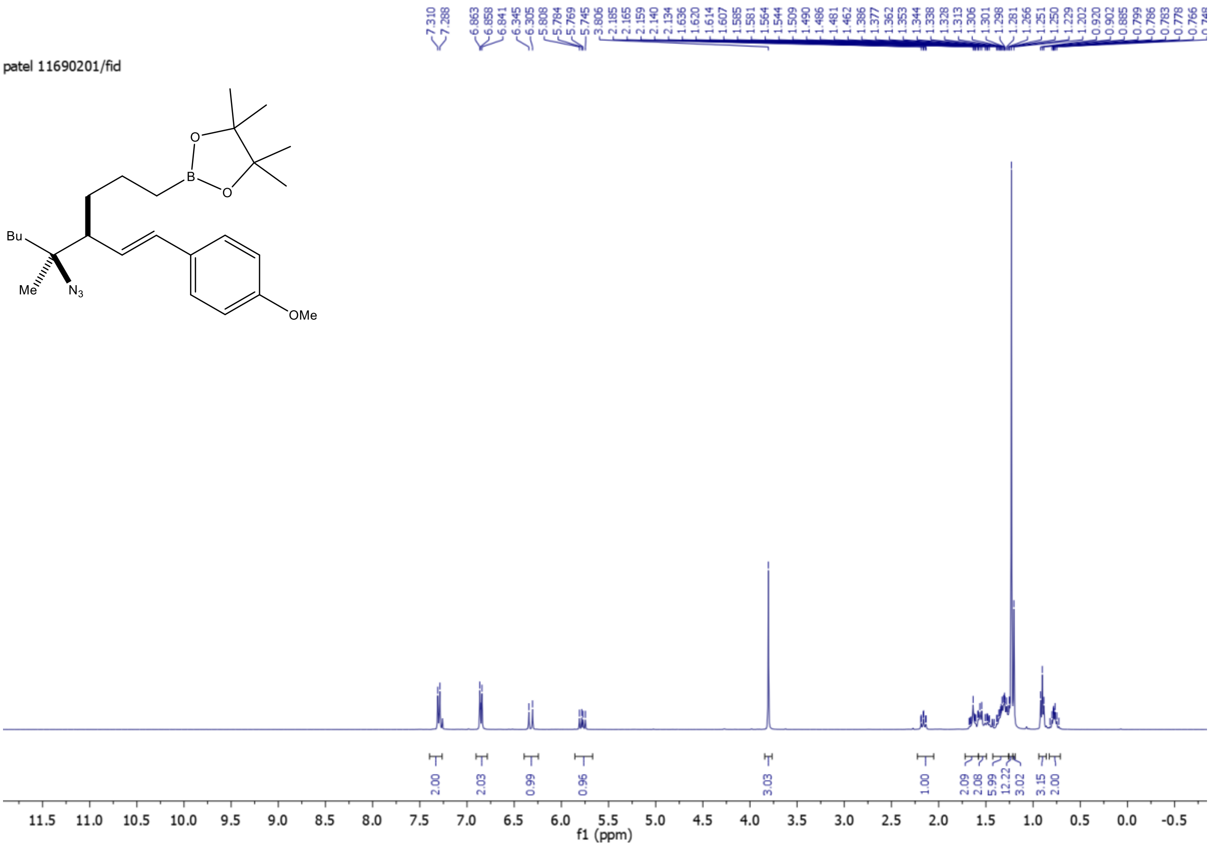
7.310
7.288
6.863
6.858
6.841
6.345
6.305
5.808
5.784
5.769
5.745
3.806
2.185
2.165
2.159
2.140
2.134
1.636
1.620
1.614
1.607
1.585
1.581
1.564
1.544
1.509
1.490
1.486
1.481
1.462
1.386
1.377
1.362
1.353
1.344
1.338
1.328
1.313
1.306
1.301
1.298
1.281
1.266
1.251
1.250
1.229
1.202
0.920
0.902
0.885
0.799
0.786
0.783
0.778
0.766
patel 11690201/fid
O
B
O
Bu
Me
N3
OMe
2.00
2.03
0.99
0.96
3.03
1.00
2.09
2.08
5.99
12.22
3.02
3.15
2.00
11.5
11.0
10.5
10.0
9.5
9.0
8.5
8.0
7.5
7.0
6.5
6.0
5.5
5.0
4.5
4.0
3.5
3.0
2.5
2.0
1.5
1.0
0.5
0.0
-0.5
f1 (ppm)

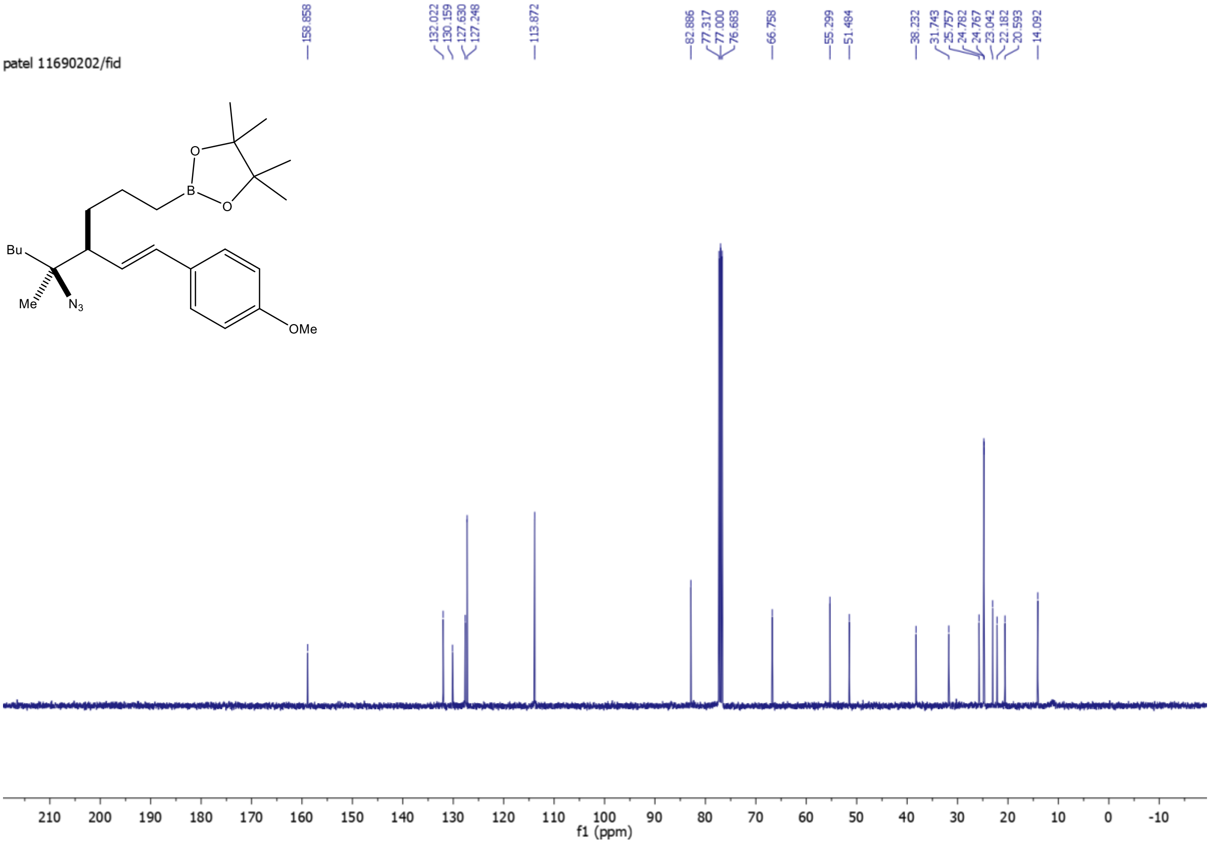
158.858
132.022
130.159
127.630
127.248
113.872
82.886
77.317
77.000
76.683
66.758
55.299
51.484
38.232
31.743
25.757
24.782
24.767
23.042
22.182
20.593
14.092
patel 11690202/fid
O
B
O
Bu
Me
N3
OMe
210
200
190
180
170
160
150
140
130
120
110
100
90
80
70
60
50
40
30
20
10
0
-10
f1 (ppm)

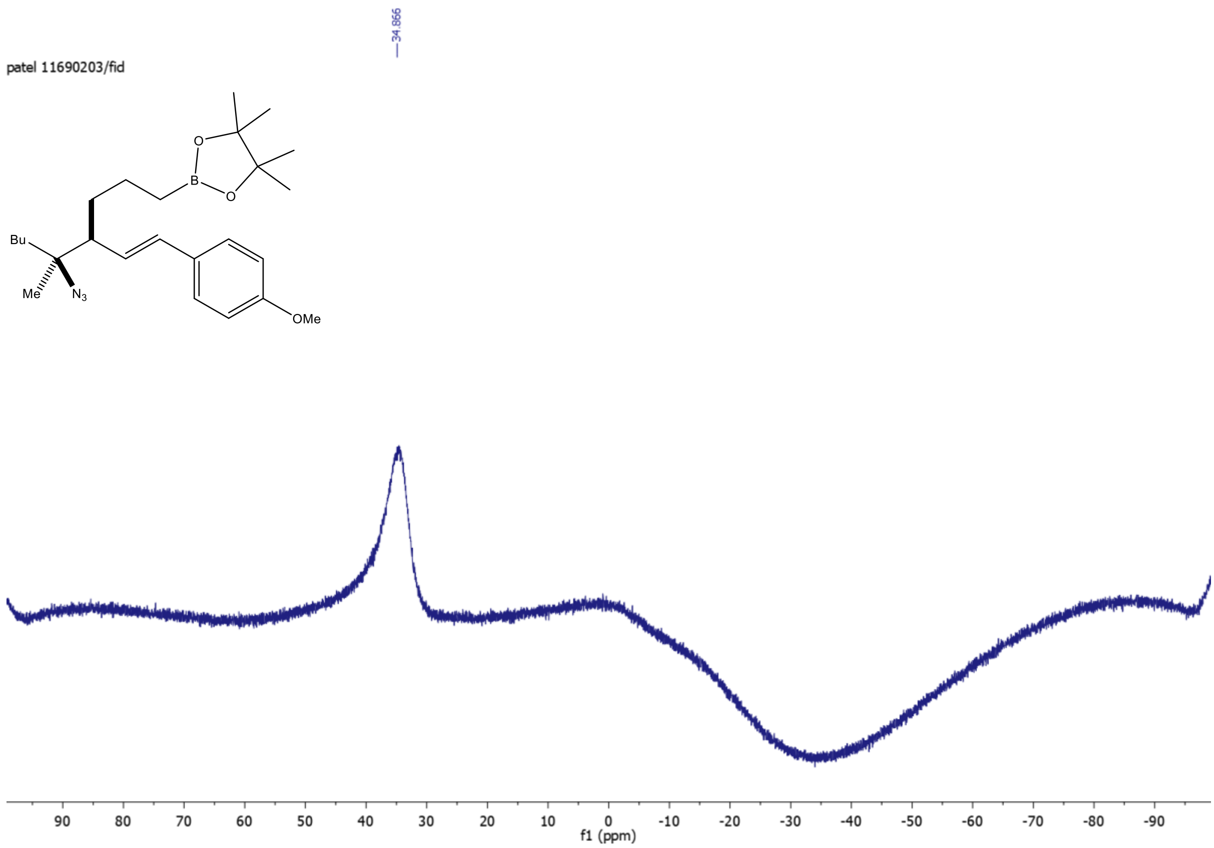
patel 11690203/fid
34.866
Bu
Me
N3
O
B
O
OMe
90 80 70 60 50 40 30 20 10 0 -10 -20 -30 -40 -50 -60 -70 -80 -90
f1 (ppm)

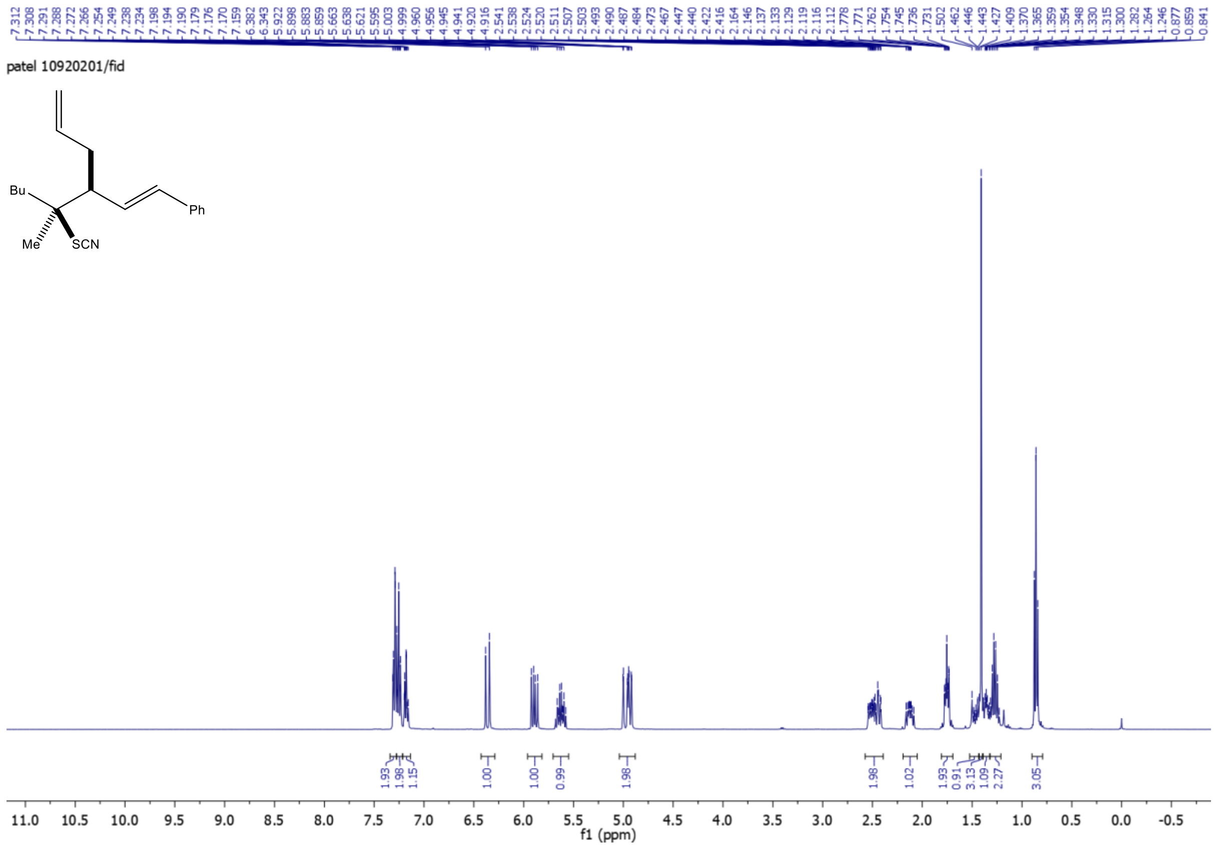
patel 10920201/fid
Bu
Me
SCN
Ph
1.93
1.98
1.15
1.00
1.00
0.99
1.98
1.98
1.02
1.93
0.91
3.13
1.09
2.27
3.05
11.0 10.5 10.0 9.5 9.0 8.5 8.0 7.5 7.0 6.5 6.0 5.5 5.0 4.5 4.0 3.5 3.0 2.5 2.0 1.5 1.0 0.5 0.0 -0.5
f1 (ppm)

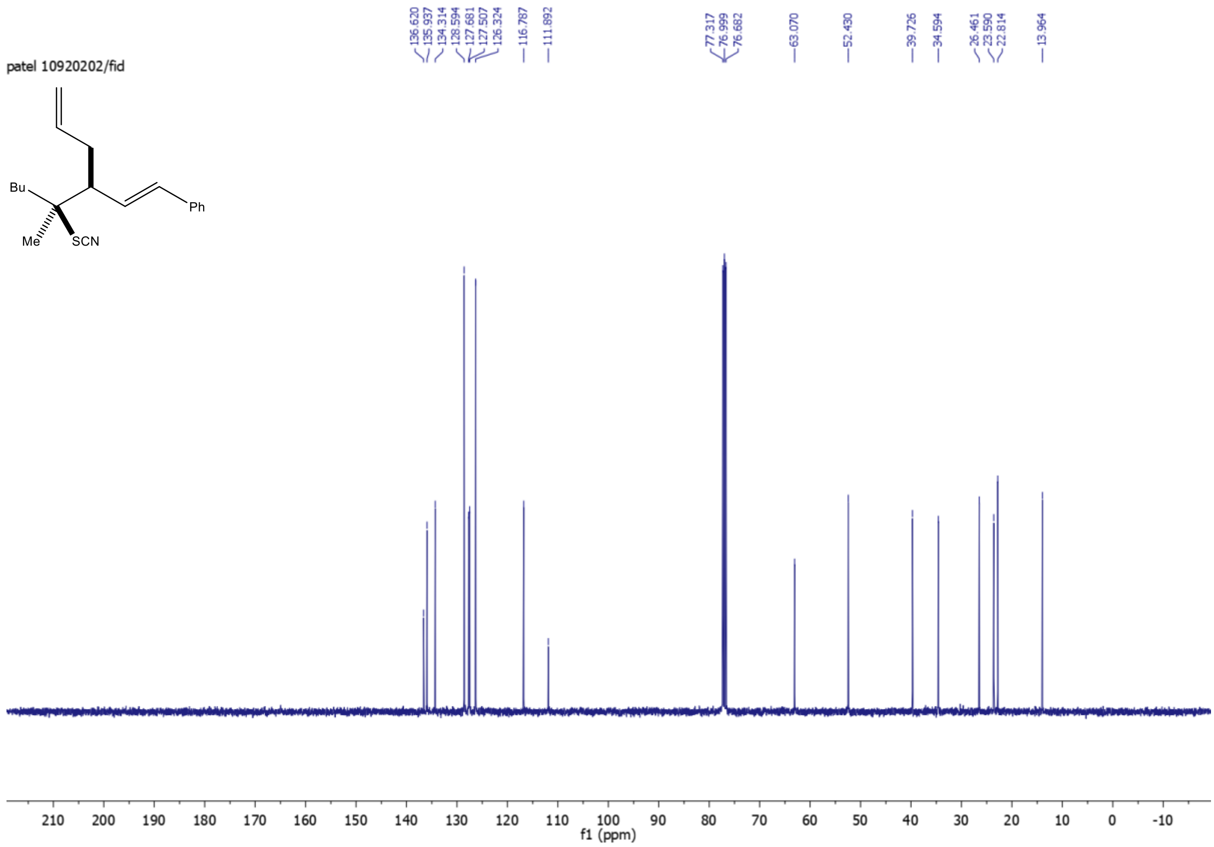
patel 10920202/fid
Bu
Me
SCN
Ph
136.620
135.937
134.314
128.594
127.681
127.507
126.324
116.787
111.892
77.317
76.999
76.682
63.070
52.430
39.726
34.594
26.461
23.590
22.814
13.964
210 200 190 180 170 160 150 140 130 120 110 100 90 80 70 60 50 40 30 20 10 0 -10
f1 (ppm)

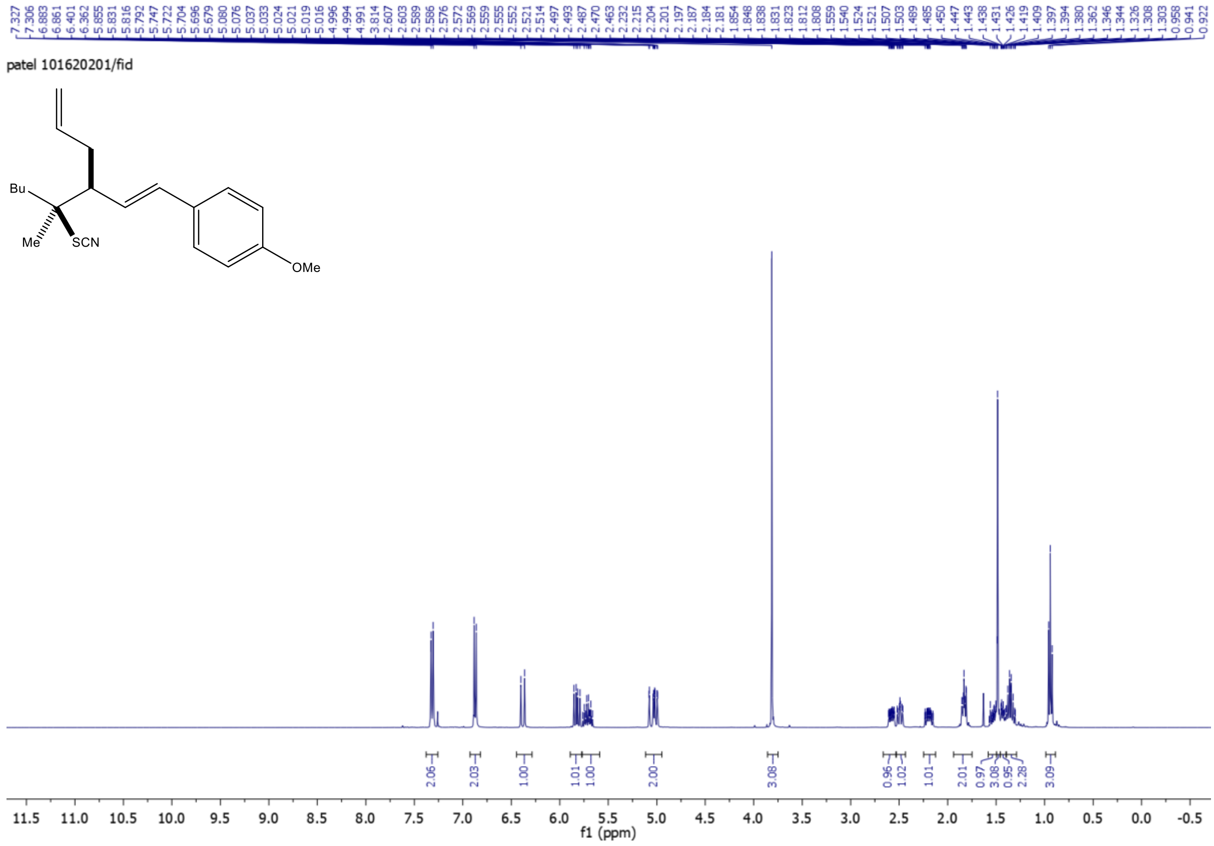
7.327
7.306
6.883
6.861
6.401
6.362
5.855
5.831
5.816
5.792
5.747
5.722
5.704
5.696
5.679
5.080
5.076
5.037
5.033
5.024
5.021
5.019
5.016
4.996
4.994
4.991
3.814
2.607
2.603
2.589
2.586
2.576
2.572
2.569
2.559
2.555
2.552
2.521
2.514
2.497
2.493
2.487
2.470
2.463
2.232
2.215
2.204
2.201
2.197
2.187
2.184
2.181
1.854
1.848
1.838
1.831
1.823
1.812
1.808
1.559
1.540
1.524
1.521
1.507
1.503
1.489
1.485
1.450
1.447
1.443
1.438
1.431
1.426
1.419
1.409
1.397
1.394
1.380
1.362
1.346
1.344
1.326
1.308
1.303
0.958
0.941
0.922
patel 101620201/fid
Bu
Me
SCN
OMe
2.06
2.03
1.00
1.01
1.00
2.00
3.08
0.96
1.02
1.01
2.01
0.97
3.08
0.95
2.28
3.09
11.5 11.0 10.5 10.0 9.5 9.0 8.5 8.0 7.5 7.0 6.5 6.0 5.5 5.0 4.5 4.0 3.5 3.0 2.5 2.0 1.5 1.0 0.5 0.0 -0.5
f1 (ppm)

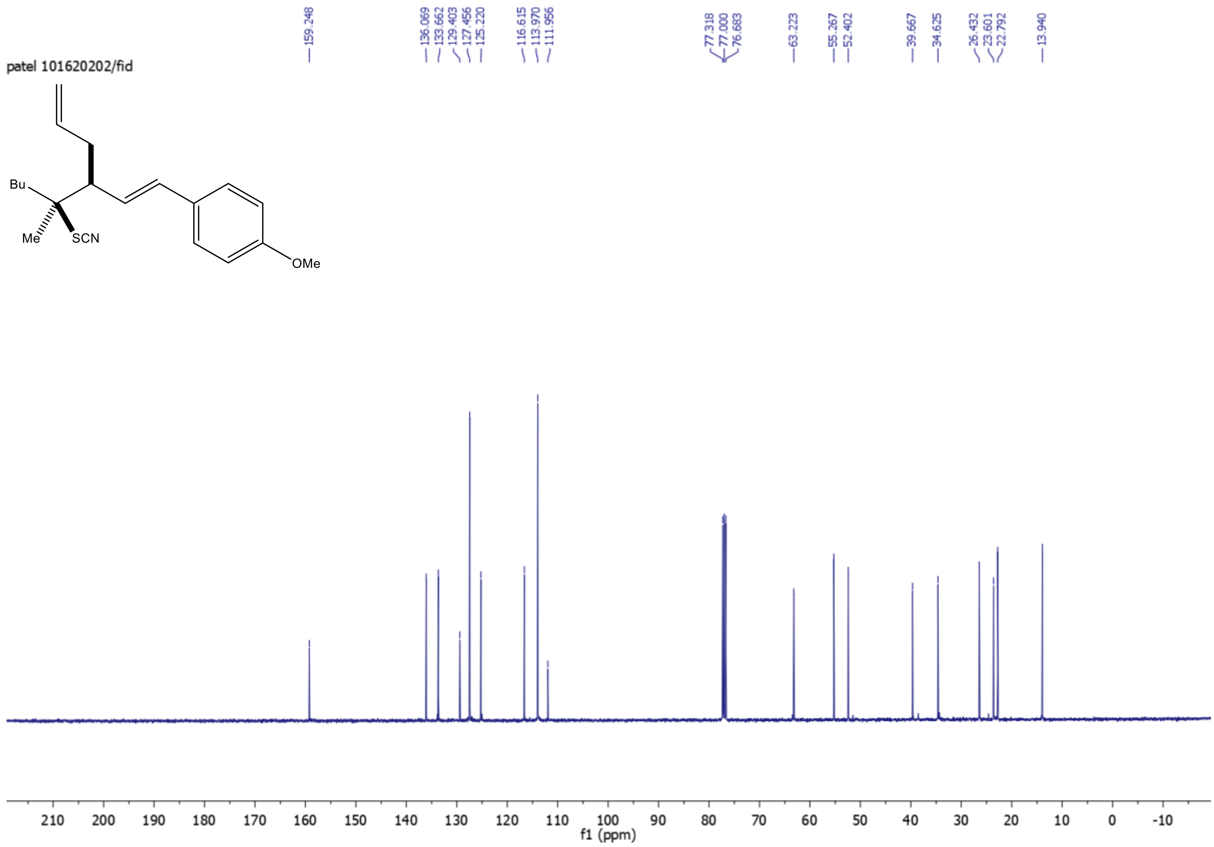
patel 101620202/fid
159.248
136.069
133.662
129.403
127.456
125.220
116.615
113.970
111.956
77.318
77.000
76.683
63.223
55.267
52.402
39.667
34.625
26.432
23.601
22.792
13.940
Bu
Me
SCN
OMe
f1 (ppm)

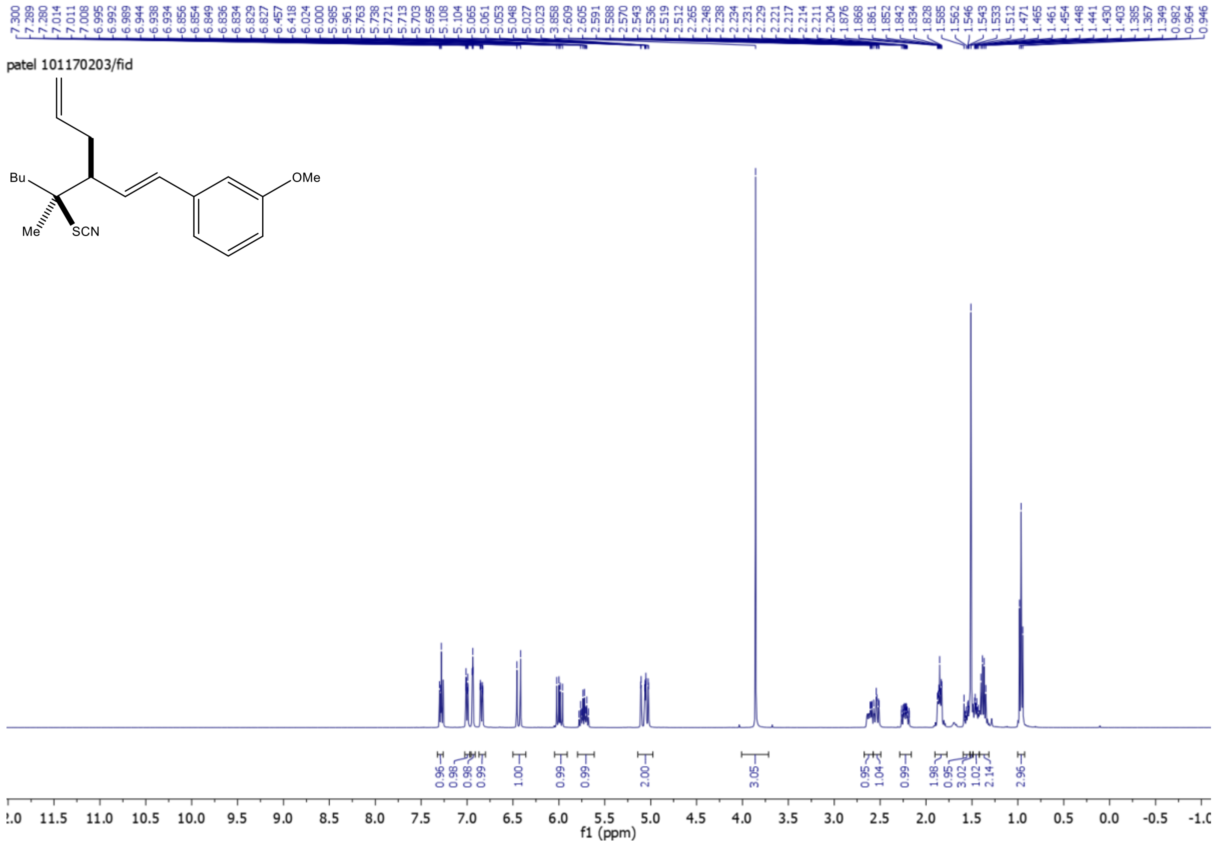
7.300
7.289
7.280
7.014
7.011
7.008
6.995
6.992
6.989
6.944
6.938
6.934
6.856
6.854
6.849
6.836
6.834
6.829
6.827
6.457
6.418
6.024
6.000
5.985
5.961
5.763
5.738
5.721
5.713
5.703
5.695
5.108
5.104
5.065
5.061
5.053
5.048
5.027
5.023
3.858
2.609
2.605
2.591
2.588
2.570
2.543
2.536
2.519
2.512
2.265
2.248
2.238
2.234
2.231
2.229
2.221
2.217
2.214
2.211
2.204
1.876
1.868
1.861
1.852
1.842
1.834
1.828
1.585
1.562
1.546
1.543
1.533
1.512
1.471
1.465
1.461
1.454
1.448
1.441
1.430
1.403
1.385
1.367
1.349
0.982
0.964
0.946
patel 101170203/fid
Bu
Me
SCN
OMe
0.96
0.98
0.98
0.99
1.00
0.99
0.99
2.00
3.05
0.95
1.04
0.99
1.98
0.95
3.02
1.02
2.14
2.96
f1 (ppm)

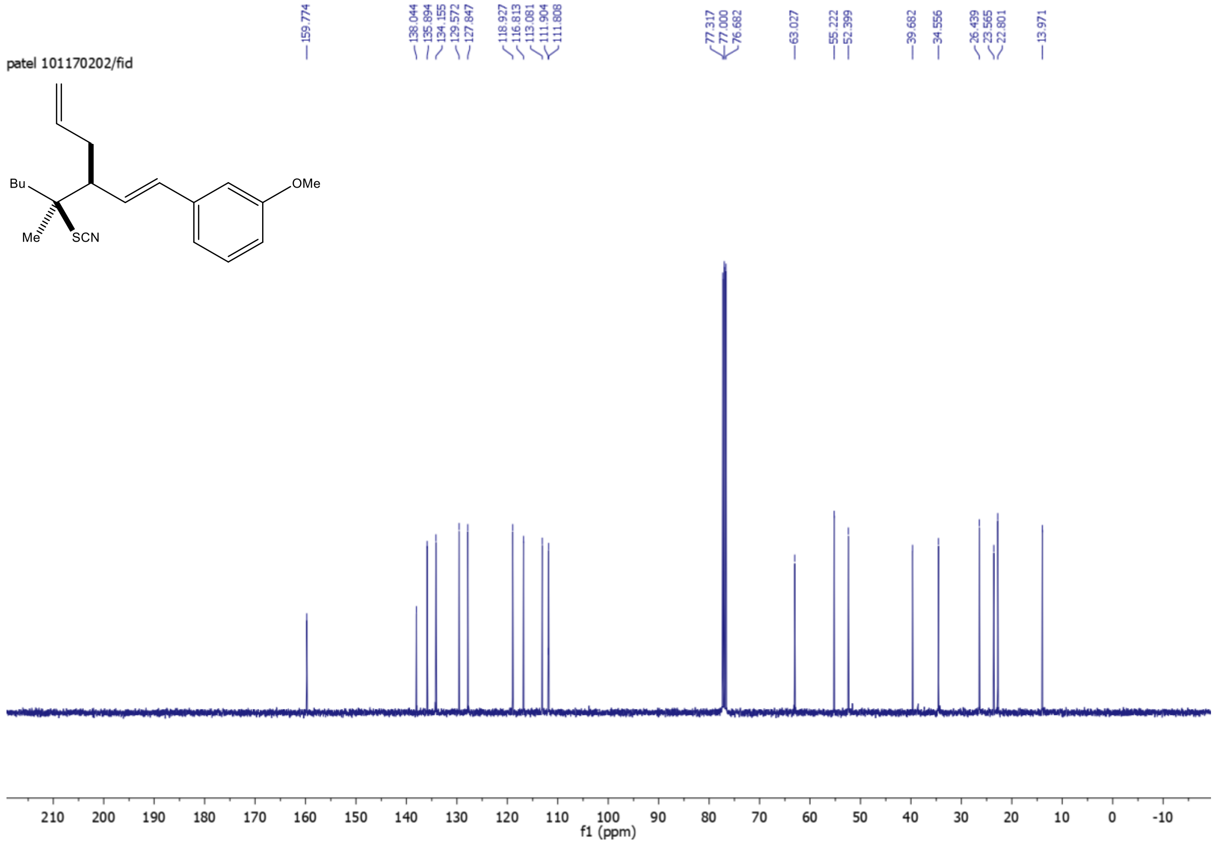
patel 101170202/fid
Bu
Me
SCN
OMe
159.774
138.044
135.894
134.155
129.572
127.847
118.927
116.813
113.081
111.904
111.808
77.317
77.000
76.682
63.027
55.222
52.399
39.682
34.556
26.439
23.565
22.801
13.971
210 200 190 180 170 160 150 140 130 120 110 100 90 80 70 60 50 40 30 20 10 0 -10
f1 (ppm)

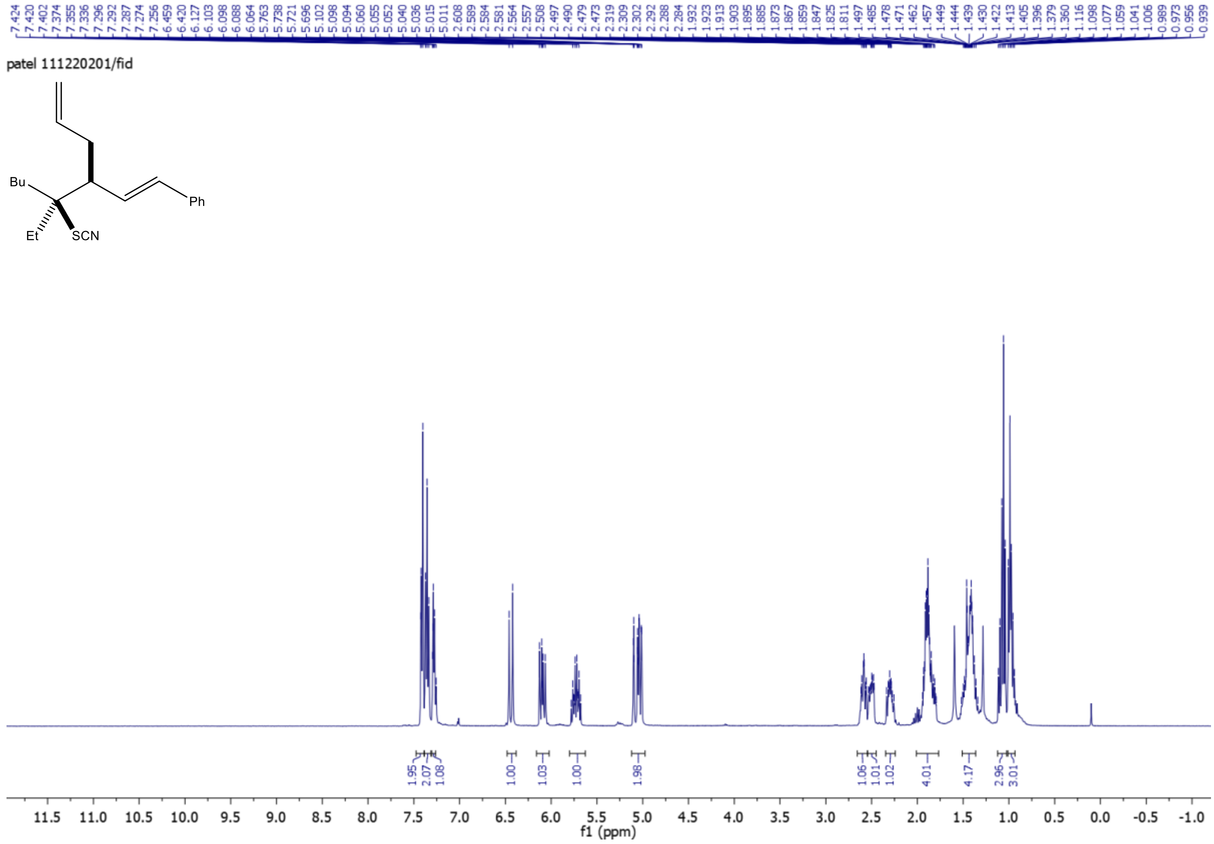
7.424 7.420 7.402 7.374 7.355 7.336 7.296 7.292 7.287 7.274 7.256 6.459 6.420 6.127 6.103 6.098 6.088 6.064 5.763 5.738 5.721 5.696 5.102 5.098 5.094 5.060 5.055 5.052 5.040 5.036 5.015 5.011 2.608 2.589 2.584 2.581 2.564 2.557 2.508 2.497 2.490 2.479 2.473 2.319 2.309 2.302 2.292 2.288 2.284 1.932 1.923 1.913 1.903 1.895 1.885 1.873 1.867 1.859 1.847 1.825 1.811 1.497 1.485 1.478 1.471 1.462 1.457 1.449 1.444 1.439 1.430 1.422 1.413 1.405 1.396 1.379 1.360 1.116 1.098 1.077 1.059 1.041 1.006 0.989 0.973 0.956 0.939
patel 111220201/fid
Bu
Et
SCN
Ph
1.95
2.07
1.08
1.00
1.03
1.00
1.98
1.06
1.01
1.02
4.01
4.17
2.96
3.01
11.5 11.0 10.5 10.0 9.5 9.0 8.5 8.0 7.5 7.0 6.5 6.0 5.5 5.0 4.5 4.0 3.5 3.0 2.5 2.0 1.5 1.0 0.5 0.0 -0.5 -1.0
f1 (ppm)

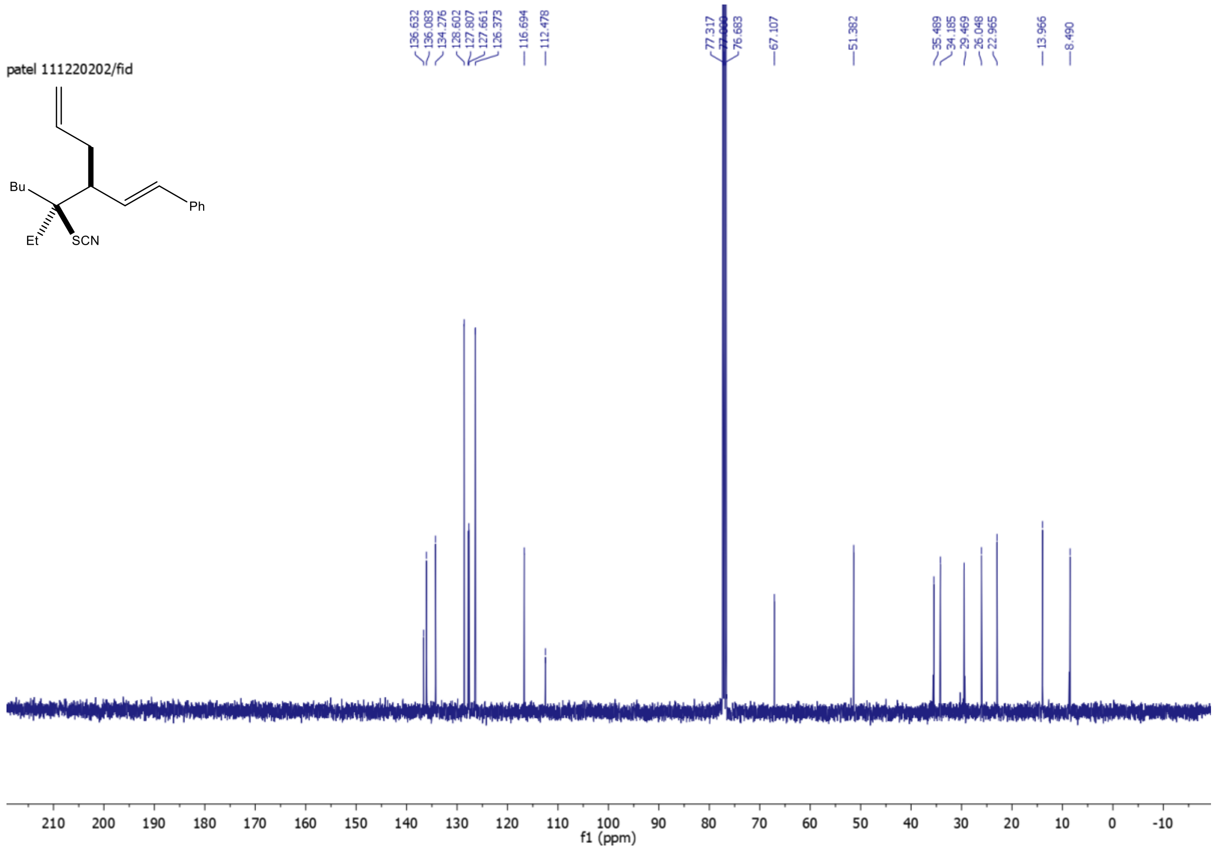
patel 111220202/fid
Bu
Et
SCN
Ph
136.632
136.083
134.276
128.602
127.807
127.661
126.373
116.694
112.478
77.317
76.683
67.107
51.382
35.489
34.185
29.469
26.048
22.965
13.966
8.490
f1 (ppm)

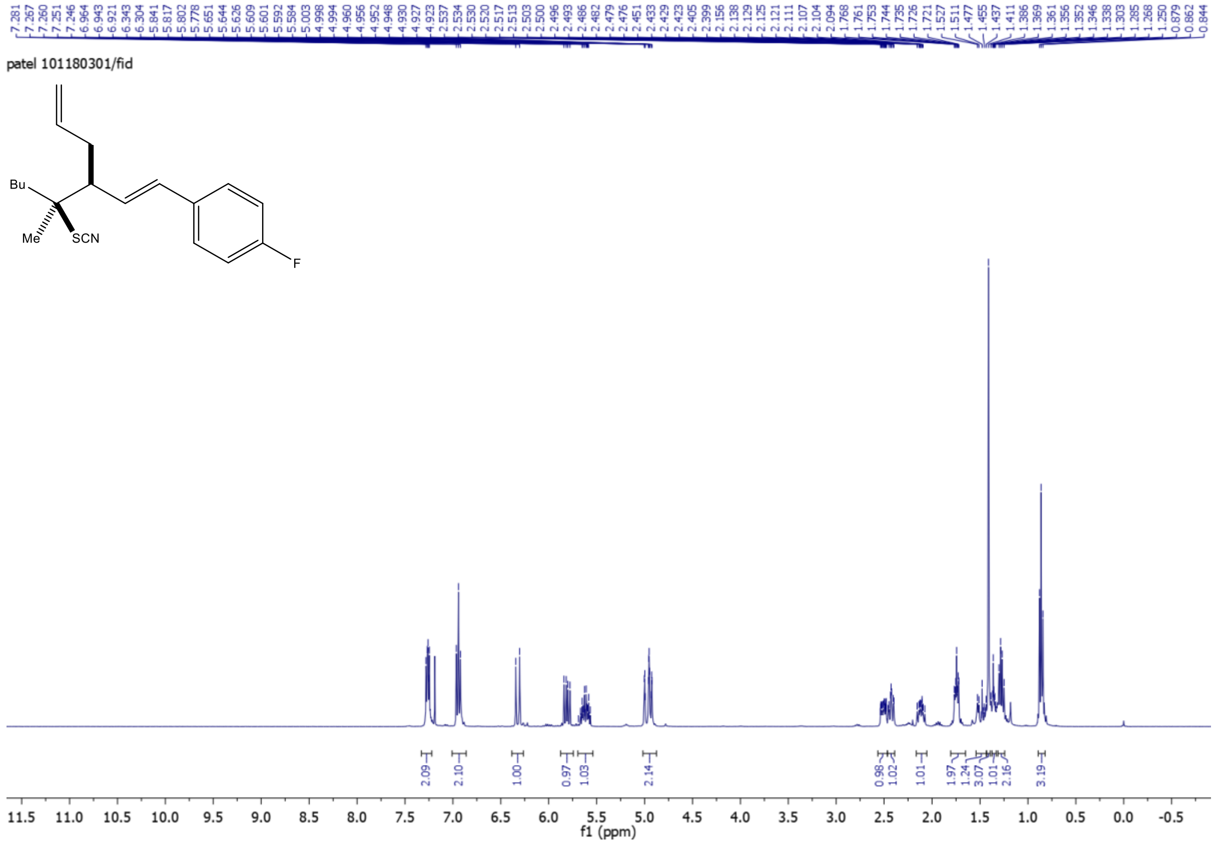
patel 101180301/fid
Bu
Me
SCN
F
7.281
7.267
7.260
7.251
7.246
6.964
6.943
6.921
6.343
6.304
5.841
5.817
5.802
5.778
5.651
5.644
5.626
5.609
5.601
5.592
5.584
5.003
4.998
4.994
4.960
4.956
4.952
4.948
4.930
4.927
4.923
2.537
2.534
2.530
2.520
2.517
2.513
2.503
2.500
2.496
2.493
2.486
2.482
2.479
2.476
2.451
2.433
2.429
2.423
2.405
2.399
2.156
2.138
2.129
2.125
2.121
2.111
2.107
2.104
2.094
1.768
1.761
1.753
1.744
1.735
1.726
1.721
1.527
1.511
1.477
1.455
1.437
1.411
1.386
1.369
1.361
1.356
1.352
1.346
1.338
1.303
1.285
1.268
1.250
0.879
0.862
0.844
2.09
2.10
1.00
0.97
1.03
2.14
0.98
1.02
1.01
1.97
1.24
3.07
1.01
2.16
3.19
f1 (ppm)

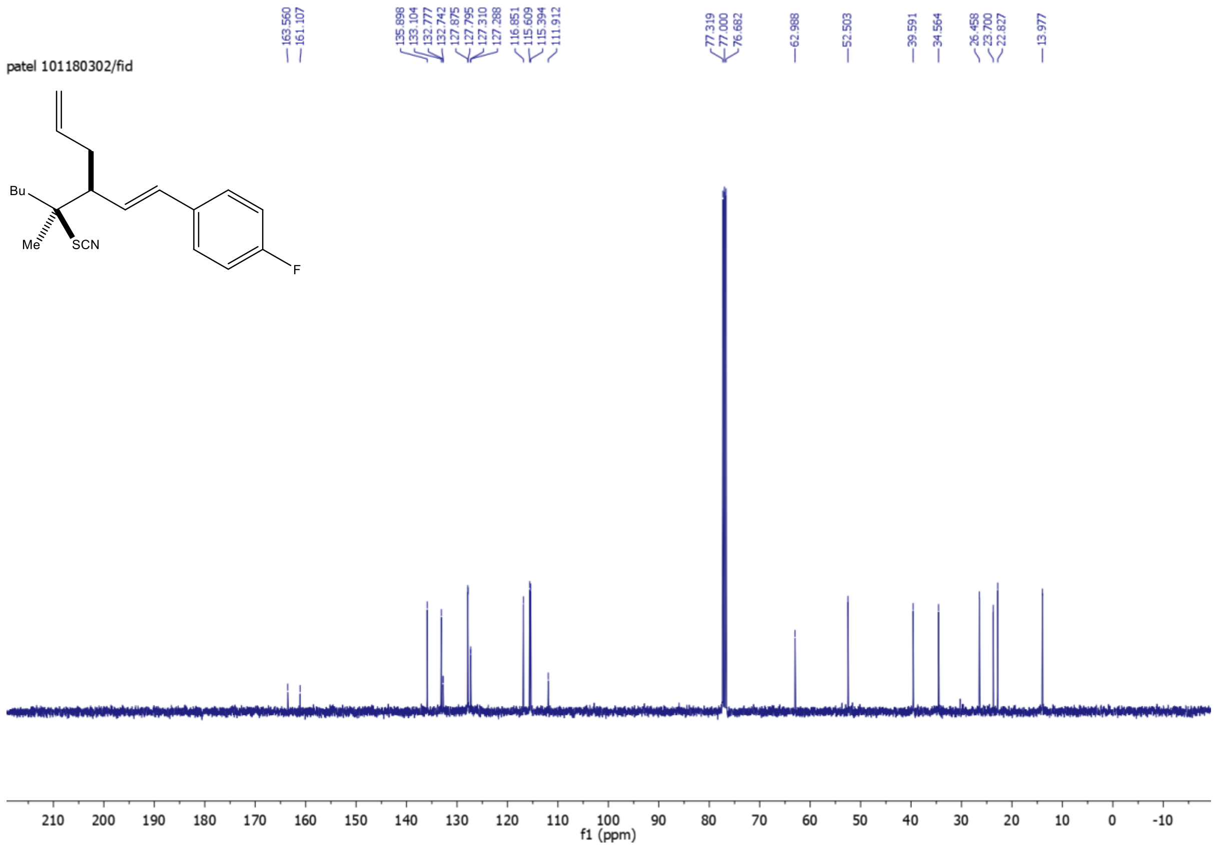
patel 101180302/fid
163.560
161.107
135.898
133.104
132.777
132.742
127.875
127.795
127.310
127.288
116.851
115.609
115.394
111.912
77.319
77.000
76.682
62.988
52.503
39.591
34.564
26.458
23.700
22.827
13.977
Bu
Me
SCN
F
f1 (ppm)

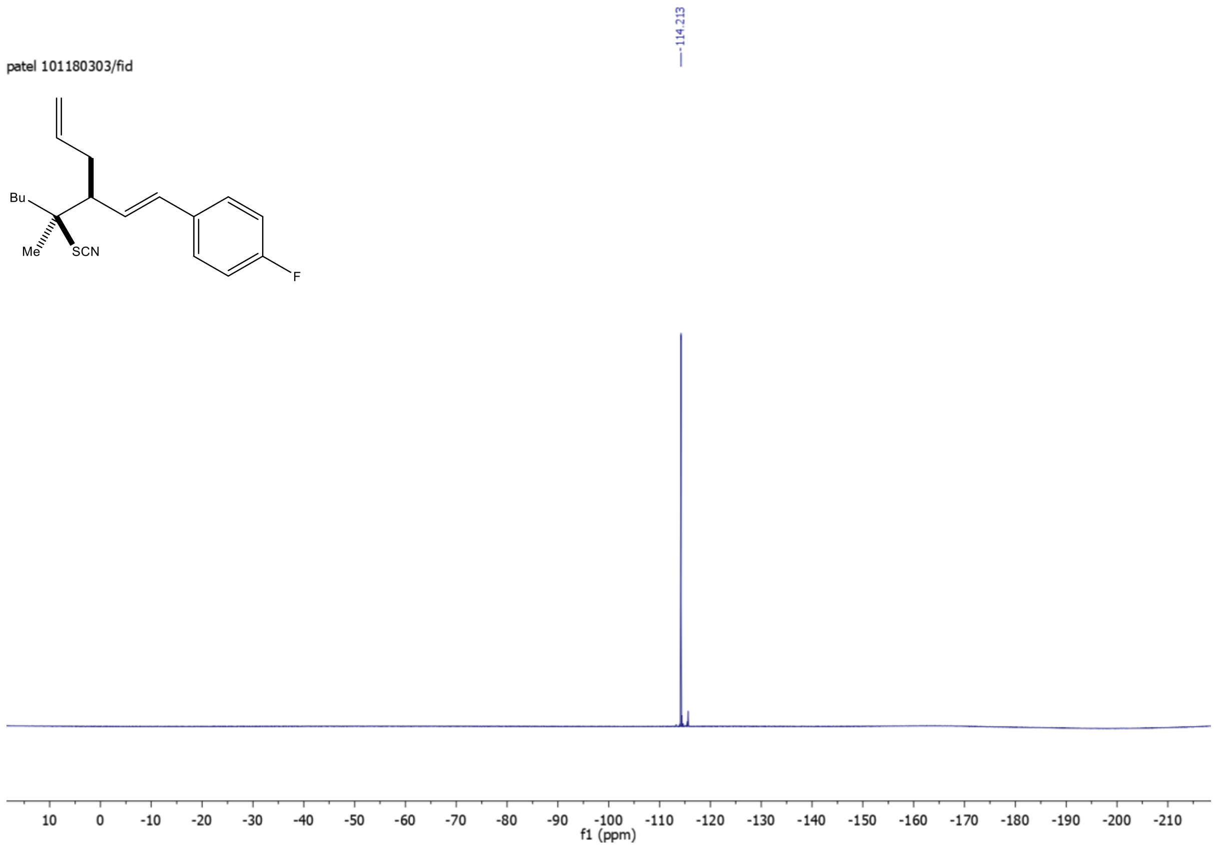
patel 101180303/fid
-114.213
Bu
Me
SCN
F
f1 (ppm)

patel 101150301/fid

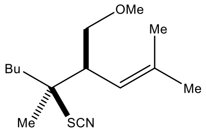

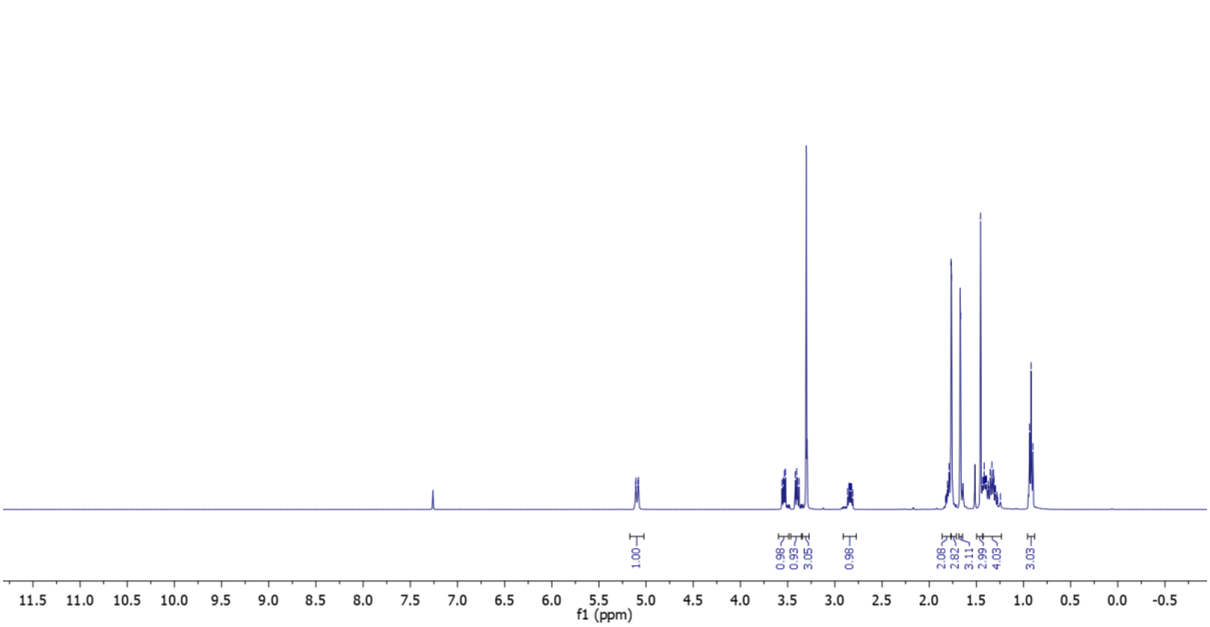

patel 101150302/fid

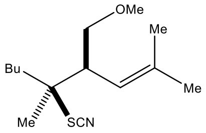

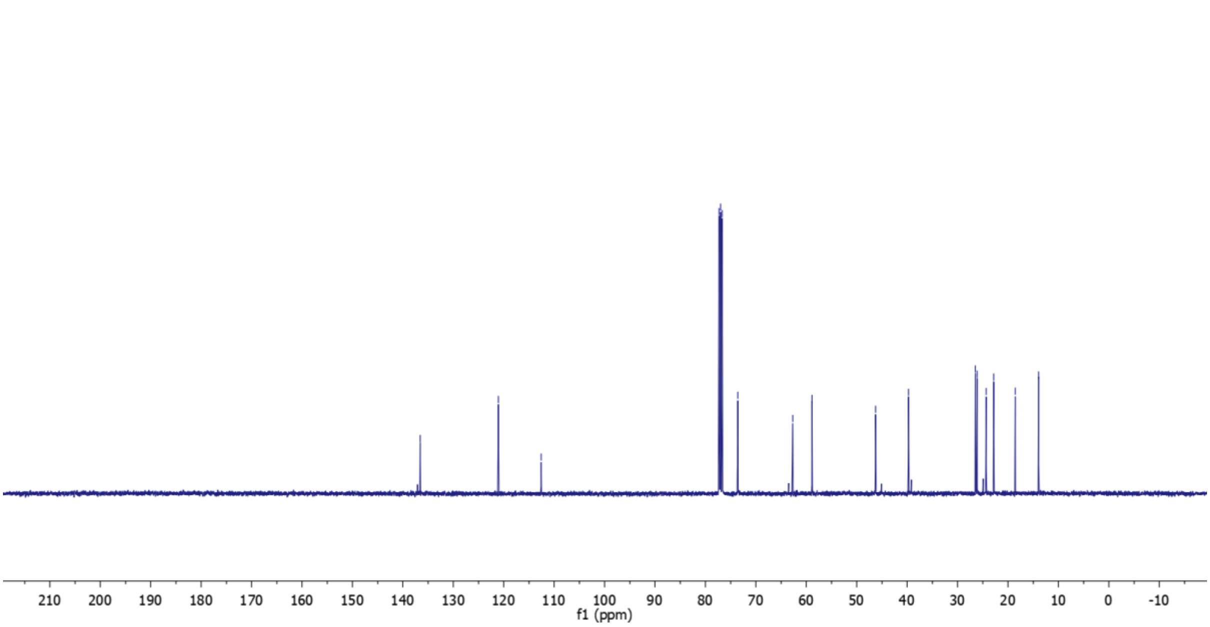

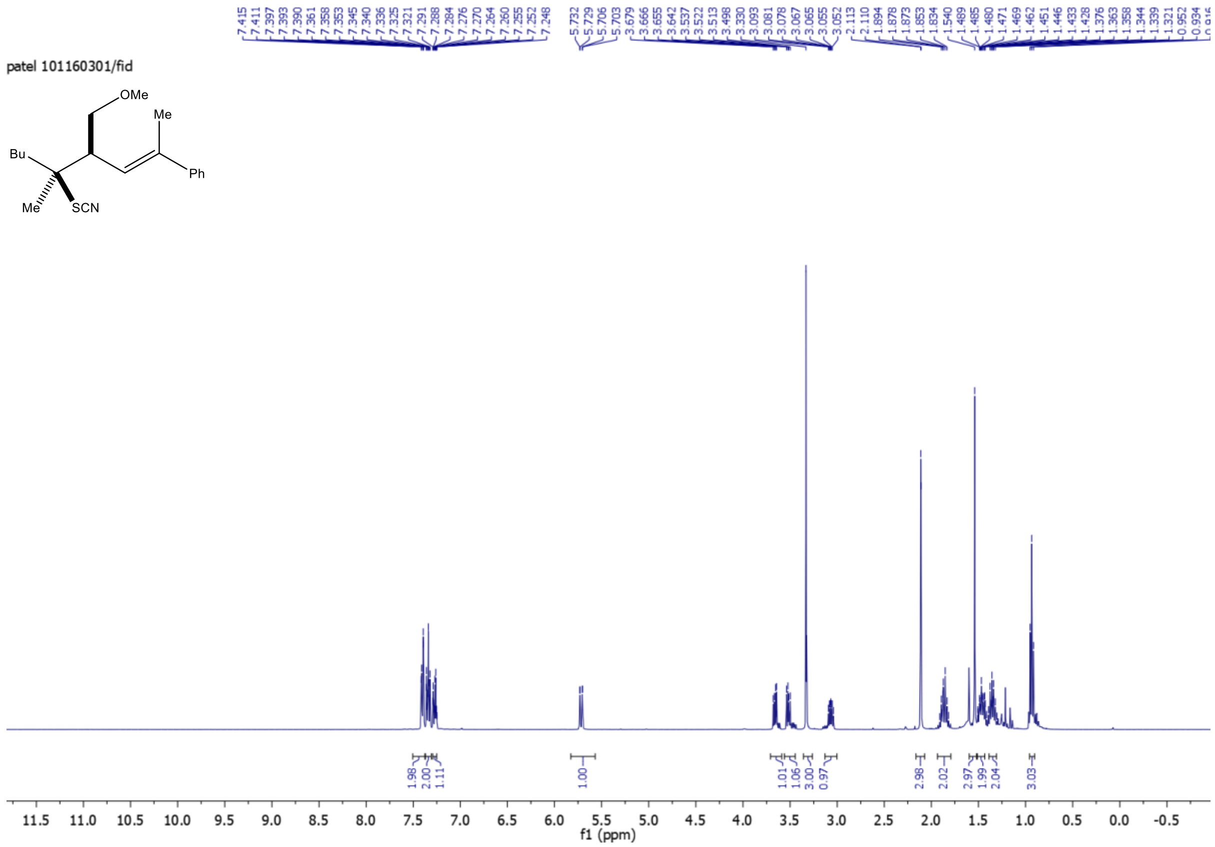
patel 101160301/fid
OMe
Me
Bu
Ph
Me
SCN
f1 (ppm)

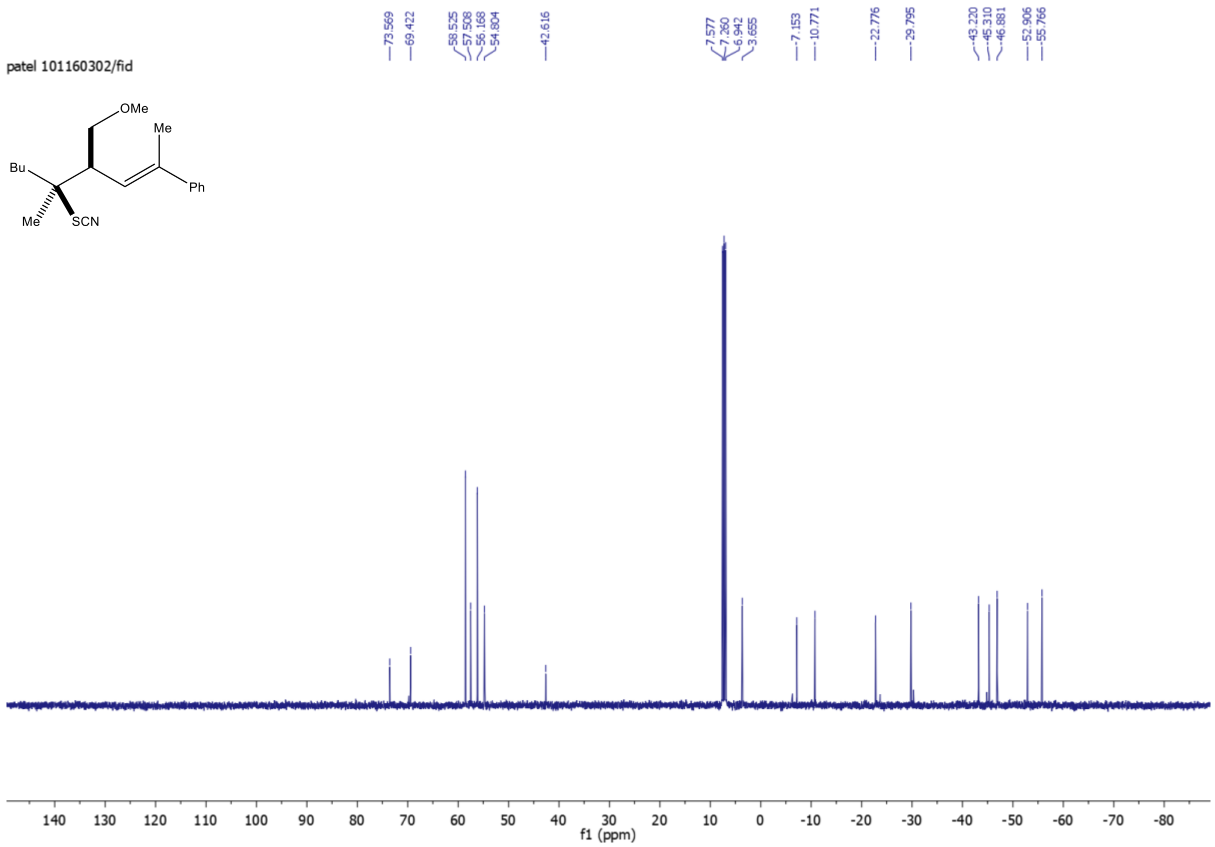
patel 101160302/fid
OMe
Me
Bu
Ph
Me
SCN
f1 (ppm)

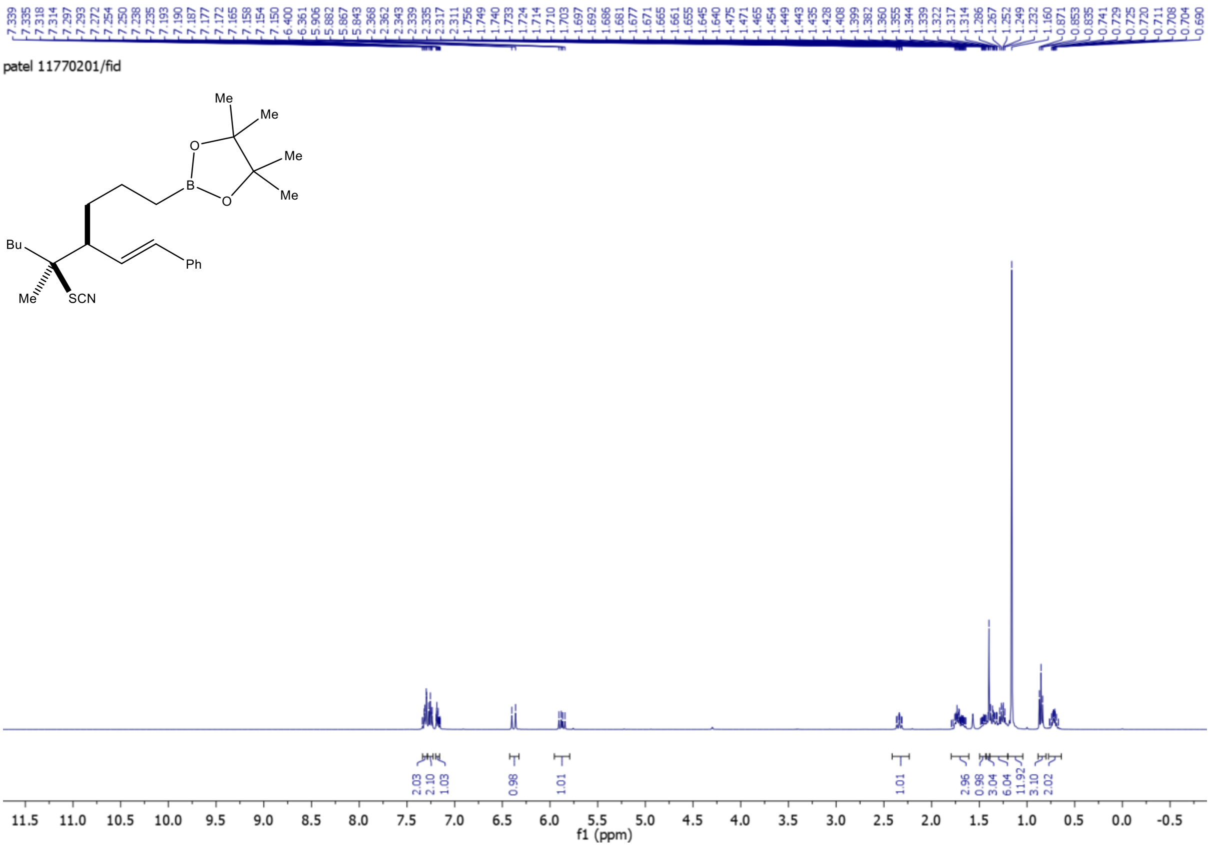
7.339
7.335
7.318
7.314
7.297
7.293
7.272
7.254
7.250
7.238
7.235
7.193
7.190
7.187
7.177
7.172
7.165
7.158
7.154
7.150
6.400
6.361
5.906
5.882
5.867
5.843
2.368
2.362
2.343
2.339
2.335
2.317
2.311
1.756
1.749
1.740
1.733
1.724
1.714
1.710
1.703
1.697
1.692
1.686
1.681
1.677
1.671
1.665
1.661
1.655
1.645
1.640
1.475
1.471
1.465
1.454
1.449
1.443
1.435
1.428
1.408
1.399
1.382
1.360
1.355
1.344
1.339
1.322
1.317
1.314
1.286
1.267
1.252
1.249
1.232
1.160
0.871
0.853
0.835
0.741
0.729
0.725
0.720
0.711
0.708
0.704
0.690
patel 11770201/fid
Me
Me
O
Me
B
O
Me
Bu
Ph
Me
SCN
2.03
2.10
1.03
0.98
1.01
1.01
2.96
0.98
3.04
6.04
11.92
3.10
2.02
f1 (ppm)

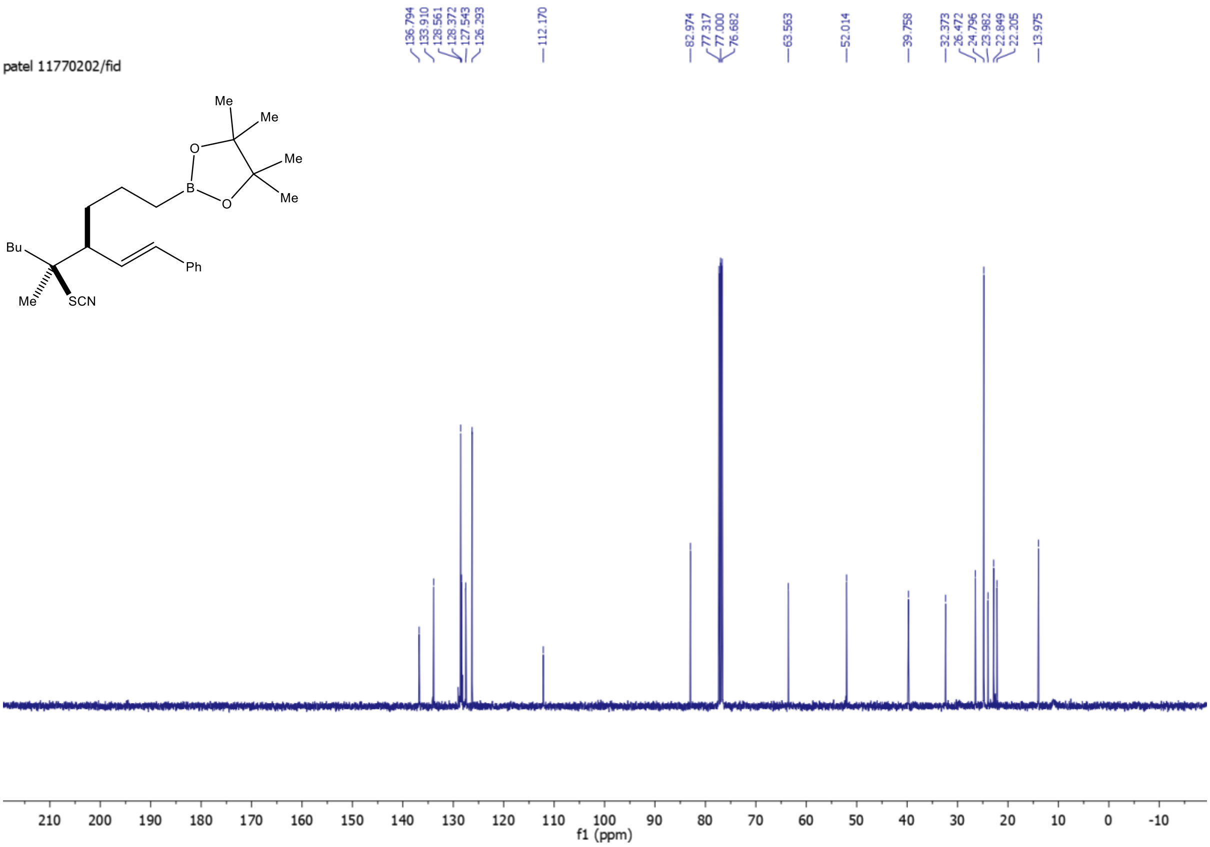
136.794
133.910
128.561
128.372
127.543
126.293
112.170
82.974
77.317
77.000
76.682
63.563
52.014
39.758
32.373
26.472
24.796
23.982
22.849
22.205
13.975
patel 11770202/fid
Me
Me
O
Me
B
O
Me
Bu
Ph
Me
SCN
f1 (ppm)

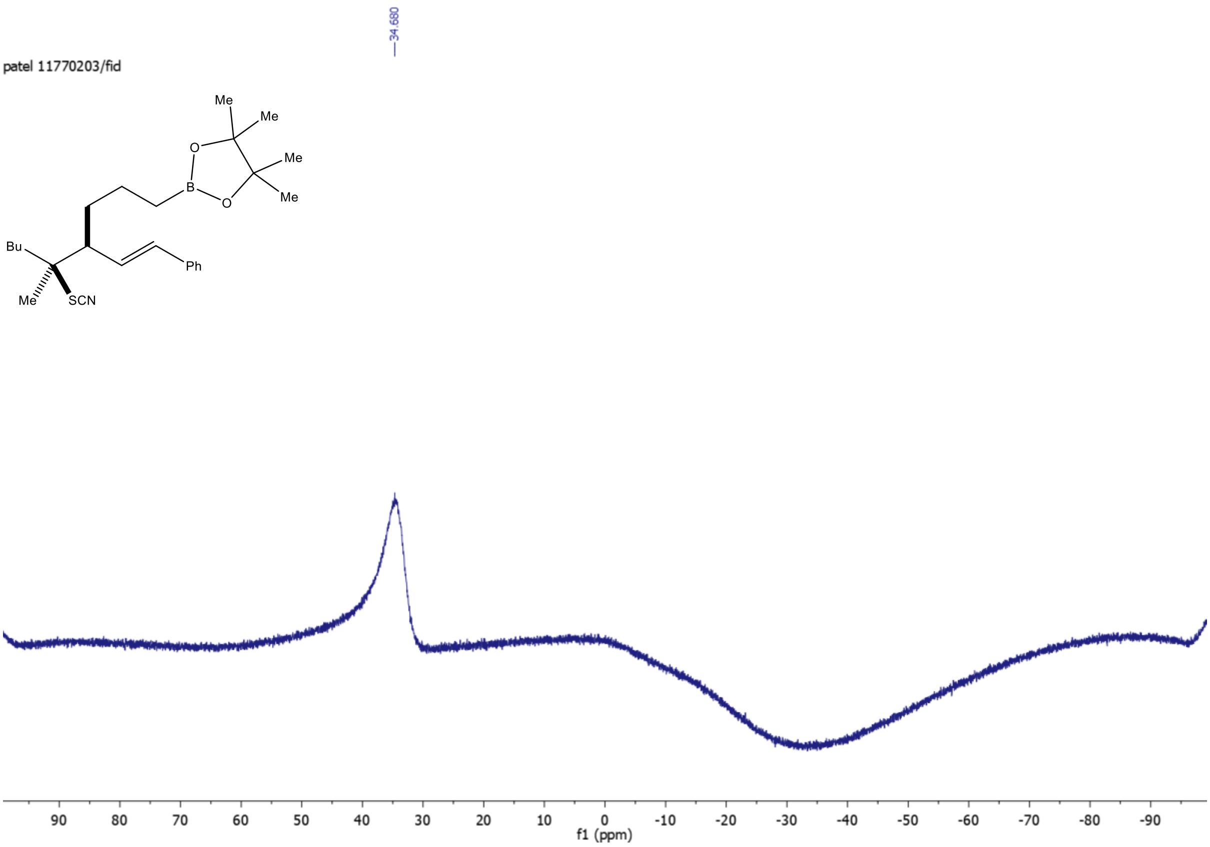
34.680
patel 11770203/fid
Me
Me
Me
Me
O
B
O
Bu
Ph
Me
SCN
90 80 70 60 50 40 30 20 10 0 -10 -20 -30 -40 -50 -60 -70 -80 -90
f1 (ppm)

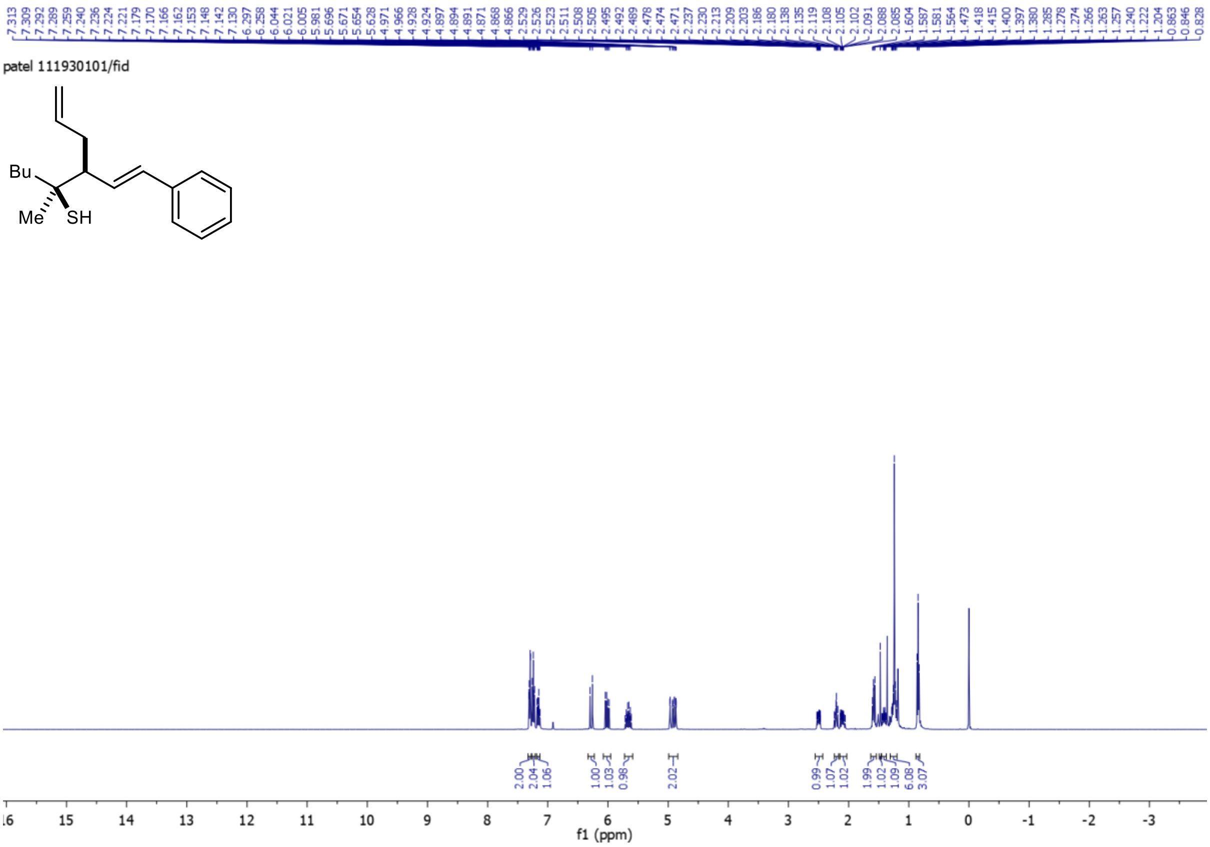
7.313 7.309 7.292 7.289 7.259 7.240 7.236 7.224 7.221 7.179 7.170 7.166 7.162 7.153 7.148 7.142 7.130 6.297 6.258 6.044 6.021 6.005 5.981 5.696 5.671 5.654 5.628 4.971 4.966 4.928 4.924 4.897 4.894 4.891 4.871 4.868 4.866 2.529 2.526 2.523 2.511 2.508 2.505 2.495 2.492 2.489 2.478 2.474 2.471 2.237 2.230 2.213 2.209 2.203 2.186 2.180 2.138 2.135 2.119 2.108 2.105 2.102 2.091 2.088 2.085 1.604 1.587 1.581 1.564 1.473 1.418 1.415 1.400 1.397 1.380 1.285 1.278 1.274 1.266 1.263 1.257 1.240 1.222 1.204 0.863 0.846 0.828
patel 111930101/fid
Bu
Me
SH
2.00 2.04 1.06 1.00 1.03 0.98 2.02 0.99 1.07 1.02 1.99 1.02 1.09 6.08 3.07
16 15 14 13 12 11 10 9 8 7 6 5 4 3 2 1 0 -1 -2 -3
f1 (ppm)

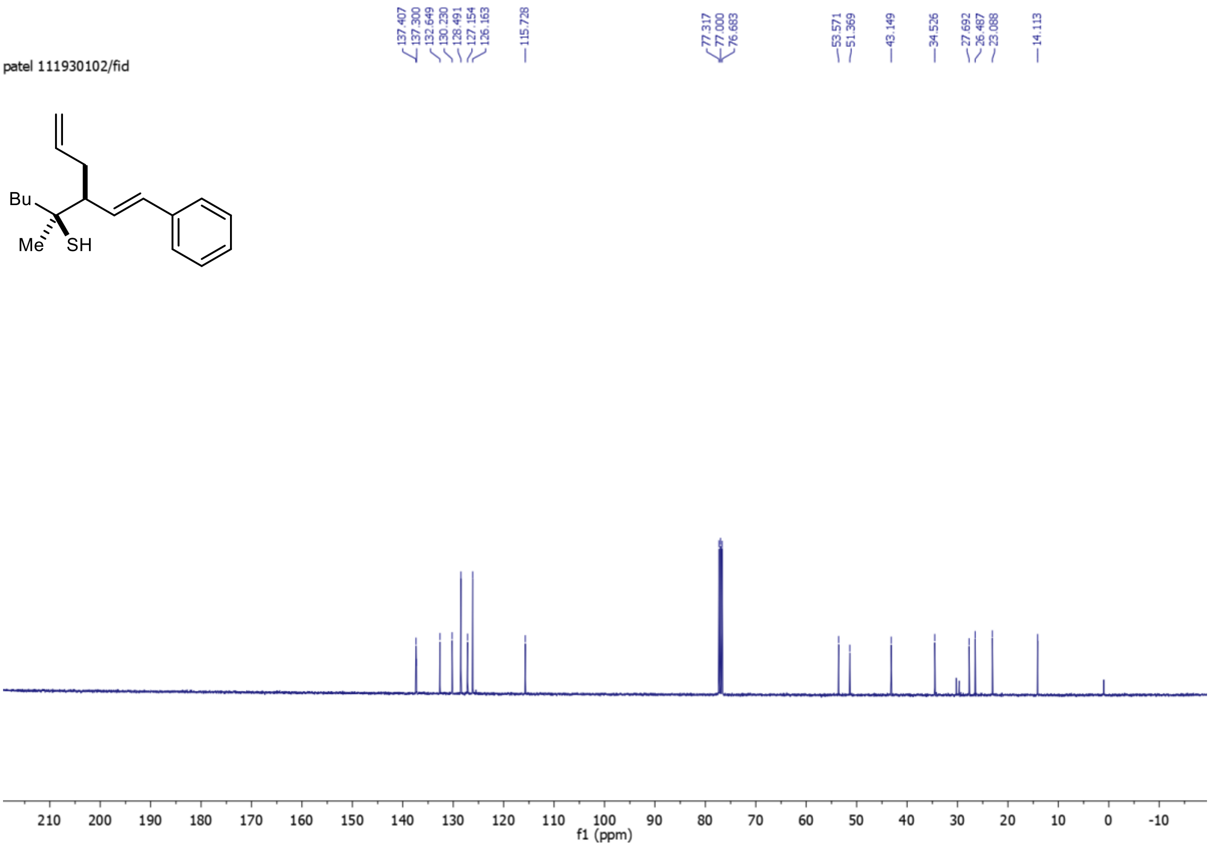

References

(1) (a) Chen, X.; Patel, K.; Marek, I. *Angew. Chem. Int. Ed.* **2023**, *62*, e202212425. (b) Patel, K.; Marek, I. *Nat. Chem*. **2025**, https://doi.org/10.1038/s41557-025-01783-2. (c) Lanke, V.; Marek, I. *J. Am. Chem. Soc*. **2020**, *142*, 5543-554 (correction: *J. Am. Chem. Soc*. **2020**, *142*, 7710-7712). (d) Chen, X.; Marek, I. *Angew. Chem. Int. Ed.* **2022**, *61*, e202203673. (e) Patel, K.; Oginetz, L.; Marek, I. *Org. Lett*. **2023**, *25*, 8474-8477.

(2) Goh, S. S.; Champagne, P. A.; Guduguntla, S.; Kikuchi, T.; Fujita, M.; Houk, K. N.; Feringa, B. L. *J. Am. Chem. Soc.* **2018**, *140*, 4986-4990.

(3) Yamamoto, Y.; Fujikawa, R.; Umemoto, T.; Miyaura, N. *Tetrahedron* **2004**, *60*, 10695-10700.

(4) Patel, K.; Lanke, V.; Marek, I. *J. Am. Chem. Soc.* **2022**, *144*, 7066-7071.
